# Supplementary material for: Absence of heterosis in hybrid crested newts
Source: PeerJ. 2018 Jul 24;6:e5317. doi: 10.7717/peerj.5317 (PMC6063215; doi:10.7717/peerj.5317)

Suppl. Inf. 2. Raw output of the KASP genotyping protocol

KBiosciences grid report
Grid version 1.03
More information is available in the Genotyping-007.004-01.csv file. This file lists only the calls for eac
Project nur 7,004
Order number
Plates Vlasi-1 Vlasi-2 Vlasi-3 Vlasi-4

| DNA \ Assa | arh_var1 | clasp2_var1 | col18_var1 | ddx17_var1 | dnaj_var1 | fam178_var1 | gak_var1 |
|---|---|---|---|---|---|---|---|
| 1808 | C:C | -:- | C:C | C:C | G:G | A:A | C:C |
| 1809 | C:C | -:- | C:C | C:C | G:G | A:A | C:C |
| 1812 | C:C | -:- | C:C | C:C | G:G | A:A | C:C |
| 1879 | C:C | -:- | C:C | C:C | G:G | A:A | C:C |
| 1880 | C:C | -:- | C:C | C:C | G:G | A:A | C:C |
| 1881 | C:C | -:- | C:C | C:C | G:G | A:A | C:C |
| 2360 | C:C | -:- | C:C | C:C | G:G | A:A | C:C |
| 2361 | C:C | -:- | C:C | C:C | G:G | A:A | C:C |
| 2362 | C:C | -:- | C:C | C:C | G:G | A:A | C:C |
| 2492 | C:C | -:- | C:C | C:C | G:G | A:A | C:C |
| 2493 | C:C | -:- | C:C | C:C | G:G | A:A | C:C |
| 2494 | C:C | -:- | C:C | C:C | G:G | A:A | C:C |
| 2579 | C:C | -:- | C:C | C:C | G:G | A:A | C:C |
| 2580 | C:C | -:- | C:C | C:C | G:G | A:A | C:C |
| 2581 | C:C | -:- | C:C | C:C | G:G | A:A | C:C |
| 2602 | C:C | -:- | C:C | C:C | G:G | A:A | C:C |
| 2603 | C:C | -:- | C:C | C:C | G:G | A:A | C:C |
| 2604 | C:C | -:- | C:C | C:C | G:G | A:A | C:C |
| 2815 | G:G | C:C | G:G | T:T | A:A | C:C | G:G |
| 2816 | G:G | C:C | G:G | T:T | A:A | C:C | G:G |
| 2817 | G:G | C:C | G:G | T:T | A:A | C:C | G:G |
| 2820 | G:G | C:C | G:G | T:T | A:A | C:C | G:G |
| 2821 | G:G | C:C | G:G | T:T | A:A | C:C | G:G |
| 2822 | G:G | C:C | G:G | T:T | A:A | C:C | G:G |
| 2846 | C:C | -:- | ? | C:C | G:G | A:A | C:C |
| 2847 | C:C | -:- | C:C | C:C | G:G | A:A | C:C |
| 2848 | C:C | -:- | C:C | C:C | G:G | A:A | C:C |
| 3245 | G:G | C:C | G:G | T:T | A:A | C:C | G:G |
| 3246 | G:G | C:C | G:G | T:T | A:A | C:C | G:G |
| 3247 | G:G | C:C | G:G | T:T | A:A | C:C | G:G |
| 3327 | G:G | C:C | G:G | T:T | A:A | C:C | G:G |
| 3472 | G:G | C:C | G:G | T:T | A:A | C:C | G:G |
| 3473 | G:G | C:C | G:G | T:T | A:A | C:C | G:G |
| 3474 | G:G | C:C | G:G | T:T | A:A | C:C | G:G |
| 3583 | G:G | C:C | G:G | T:T | A:A | C:C | G:G |
| 3584 | G:G | C:C | G:G | T:T | A:A | C:C | G:G |
| 3601 | G:G | C:C | G:G | T:T | A:A | C:C | G:G |
| 3602 | G:G | C:C | C:G | T:T | A:A | C:C | G:G |
| 3603 | G:G | C:C | G:G | T:T | A:A | C:C | G:G |
| 3775 | G:G | C:C | G:G | T:T | A:A | C:C | G:G |

| | | | | | | | |
|---|---|---|---|---|---|---|---|
| 3776 | G:G | C:C | G:G | T:T | A:A | C:C | G:G |
| 3777 | G:G | C:C | G:G | T:T | A:A | C:C | G:G |
| 6284 | C:G | -:C | G:G | C:C | ? | A:A | C:C |
| 6285 | C:G | -:C | C:C | C:T | G:A | A:C | C:G |
| 6286 | C:G | -:C | C:G | C:T | G:A | A:C | G:G |
| 6287 | G:G | -:- | C:G | C:T | G:A | A:A | C:G |
| 6288 | C:G | -:- | C:G | C:C | G:G | A:C | C:G |
| 6289 | C:G | -:- | C:G | C:T | G:A | A:C | C:G |
| 6290 | C:G | -:C | C:G | C:T | G:G | C:C | C:G |
| 6291 | C:C | -:C | G:G | C:C | G:G | A:A | C:G |
| 6292 | C:C | -:C | C:C | C:C | G:G | A:C | G:G |
| 6293 | C:C | -:- | G:G | C:T | G:G | A:C | C:C |
| 6294 | C:C | C:C | C:G | C:C | G:G | A:C | G:G |
| 6295 | C:G | -:C | G:G | C:C | G:A | A:C | C:G |
| 6296 | C:G | -:C | G:G | C:T | G:G | A:C | C:G |
| 6297 | C:C | C:C | C:G | T:T | G:G | A:C | C:G |
| 6298 | C:C | -:C | C:G | C:C | G:A | A:A | C:G |
| 6299 | C:G | -:- | C:G | C:T | A:A | C:C | C:G |
| 6300 | C:G | -:- | C:G | T:T | G:A | A:C | C:G |
| 6301 | G:G | C:C | C:G | T:T | G:A | A:C | G:G |
| 6302 | C:G | -:C | C:C | C:C | G:G | A:A | C:G |
| 6303 | G:G | -:- | G:G | C:T | G:A | A:C | C:G |
| 6304 | C:G | -:C | C:G | C:T | G:A | A:A | C:C |
| 6305 | C:G | C:C | C:G | C:C | G:G | A:A | C:G |
| 6306 | C:G | -:C | G:G | C:T | G:A | A:A | C:G |
| 6307 | C:G | -:- | C:G | C:T | G:G | C:C | G:G |
| 6308 | C:G | C:C | C:G | C:T | G:A | A:C | C:G |
| 6309 | C:C | -:- | G:G | C:C | G:A | A:C | C:C |
| 6310 | C:C | -:C | C:G | C:C | G:G | A:C | C:G |
| 6311 | G:G | -:- | C:G | C:T | G:A | A:A | G:G |
| 6312 | C:G | -:- | C:G | C:T | G:G | A:A | C:G |
| 6313 | C:G | -:- | C:G | C:T | G:A | A:A | ? |
| 6314 | C:C | -:C | C:G | C:C | G:A | C:C | C:G |
| 6315 | C:C | -:C | C:G | C:C | G:G | A:C | G:G |
| 6316 | C:C | -:- | C:C | C:C | G:G | A:A | G:G |
| 6317 | C:G | -:- | G:G | C:T | G:A | A:A | C:G |
| 6318 | C:G | -:- | C:G | C:T | G:A | C:C | C:C |
| 6319 | C:G | -:- | C:G | C:T | A:A | A:C | C:G |
| 6320 | C:G | -:- | C:C | C:C | G:G | A:A | C:G |
| 6321 | C:C | -:C | G:G | C:C | G:G | A:C | C:G |
| 6322 | C:C | -:- | G:G | C:C | G:A | A:A | C:G |
| 6323 | C:C | C:C | C:G | C:T | G:G | A:A | C:G |
| 6324 | C:G | -:C | G:G | C:T | A:A | A:A | C:G |
| 6325 | C:C | C:C | C:G | C:T | G:G | A:A | G:G |
| 6326 | C:C | -:- | G:G | C:T | G:A | A:C | C:G |
| 6327 | C:G | C:C | G:G | C:C | G:G | A:C | G:G |
| 6328 | C:C | -:C | C:C | C:T | G:G | A:A | G:G |
| 6329 | C:G | -:C | G:G | C:T | G:A | A:A | C:G |
| 6330 | C:C | C:C | G:G | C:T | G:G | A:C | C:G |
| 6331 | C:C | -:- | G:G | C:C | G:G | C:C | C:C |

| | | | | | | | |
|---|---|---|---|---|---|---|---|
| 6332 | G:G | -:- | C:G | T:T | G:G | A:A | G:G |
| 6333 | C:C | -:- | C:C | C:T | G:G | A:C | C:C |
| 6334 | C:G | C:C | G:G | C:T | G:G | C:C | C:C |
| 6335 | C:G | -:C | C:G | C:C | G:G | A:C | G:G |
| 6336 | C:C | -:C | C:C | C:C | G:G | A:A | C:G |
| 6337 | C:G | C:C | C:G | C:C | G:G | A:C | C:G |
| 6338 | C:G | -:- | C:G | C:C | G:A | C:C | G:G |
| 6339 | C:C | -:C | C:G | C:C | G:G | A:A | G:G |
| 6340 | C:G | -:C | C:G | C:T | G:A | A:A | C:G |
| 6341 | C:C | -:C | G:G | C:C | G:G | C:C | C:G |
| 6342 | C:C | -:- | C:G | C:C | A:A | A:C | C:G |
| 6343 | C:G | -:C | C:G | T:T | G:A | A:A | C:C |
| 6344 | C:G | -:C | C:G | T:T | G:A | A:A | C:C |
| 6345 | C:G | C:C | C:G | C:T | G:A | A:A | C:G |
| 6346 | C:C | -:C | C:G | C:C | G:G | C:C | G:G |
| 6347 | C:C | C:C | C:G | C:T | G:A | C:C | C:G |
| 6348 | C:G | -:C | C:G | C:T | G:G | A:C | C:G |
| 6349 | C:C | C:C | G:G | C:C | G:G | A:A | C:G |
| 6350 | C:C | -:- | C:C | C:T | A:A | A:A | C:G |
| 6351 | C:G | -:- | G:G | C:C | G:G | A:A | C:C |
| 6352 | C:G | -:- | ? | C:T | G:A | A:A | C:G |
| 6353 | C:G | -:- | G:G | C:T | G:A | A:C | C:G |
| 6354 | C:C | -:- | C:G | T:T | G:G | A:C | C:C |
| 6355 | C:G | -:C | C:G | C:C | G:G | A:C | C:G |
| 6356 | G:G | -:C | C:G | C:T | G:A | A:C | G:G |
| 6357 | C:G | -:C | C:G | C:T | G:A | A:C | C:G |
| 6358 | C:C | -:C | C:G | C:C | G:G | A:A | C:G |
| 6359 | G:G | -:- | C:C | C:T | G:A | A:C | C:C |
| 6360 | G:G | C:C | C:G | T:T | G:A | A:A | G:G |
| 6361 | G:G | C:C | G:G | T:T | G:A | C:C | G:G |
| 6362 | C:G | -:C | C:G | C:T | A:A | A:A | C:C |
| 6363 | C:C | -:C | C:C | C:C | G:G | A:A | G:G |
| 6364 | C:C | -:C | G:G | C:C | G:G | C:C | C:C |
| 6365 | C:G | -:- | C:G | C:T | G:A | A:C | G:G |
| 6366 | C:C | -:C | G:G | C:C | G:A | A:C | C:G |
| 6367 | C:G | -:- | C:G | C:T | G:G | A:C | C:G |
| 6368 | G:G | C:C | C:G | C:T | G:G | A:A | G:G |
| 6369 | G:G | C:C | C:G | T:T | A:A | A:A | C:G |
| 6370 | C:C | -:- | G:G | C:C | G:A | A:A | C:C |
| 6371 | C:G | -:- | C:C | C:C | G:G | A:C | C:G |
| 6372 | C:C | -:- | C:G | C:C | G:A | C:C | C:G |
| 6373 | C:G | -:- | C:G | C:C | G:G | A:C | C:G |
| 6374 | C:G | -:C | C:C | C:T | G:A | A:C | C:C |
| 6375 | C:G | -:C | G:G | C:T | G:G | A:C | C:G |
| 6376 | C:G | C:C | C:G | C:T | G:A | C:C | C:G |
| 6377 | C:C | -:- | C:G | C:C | G:G | A:C | C:G |
| 6378 | C:G | -:- | C:G | C:C | G:G | A:C | C:G |
| 6379 | C:C | -:- | G:G | C:C | G:G | A:A | G:G |
| 6380 | C:G | -:C | C:C | T:T | G:A | A:C | C:G |
| 6381 | C:G | -:C | C:G | T:T | G:A | A:A | C:G |

| | | | | | | | |
|---|---|---|---|---|---|---|---|
| 6382 | C:C | C:C | C:C | C:T | G:A | A:A | G:G |
| 6383 | C:C | -:C | G:G | C:C | G:A | A:C | C:G |
| 6384 | C:C | C:C | C:G | C:C | G:G | A:A | C:C |
| 6385 | C:G | -:C | C:C | C:T | G:A | A:C | C:G |
| 6386 | C:G | -:- | C:G | C:T | G:A | A:C | C:C |
| 6387 | C:C | -:- | C:C | C:C | G:G | A:C | C:C |
| 6388 | C:G | -:C | G:G | C:C | G:A | A:C | C:G |
| 6389 | C:G | -:- | C:G | C:T | G:A | A:A | C:G |
| 6390 | C:G | -:C | C:C | C:T | G:A | A:A | C:G |
| 6391 | C:C | -:C | G:G | C:C | G:G | A:C | C:G |
| 6392 | C:C | -:C | C:G | C:C | G:G | A:A | C:G |
| 6393 | C:C | C:C | C:C | C:C | G:G | A:A | C:G |
| 6394 | C:C | -:- | C:C | C:T | G:G | A:C | C:G |
| 6395 | C:G | -:- | G:G | C:C | G:A | A:A | C:G |
| 6396 | C:C | -:C | C:C | C:T | G:A | A:A | C:C |
| 6397 | C:G | -:C | C:C | C:T | G:A | A:C | G:G |
| 6398 | C:G | -:- | C:C | C:T | A:A | A:A | G:G |
| 6399 | C:G | -:C | C:C | C:T | G:A | A:C | C:G |
| 6400 | C:C | -:C | C:G | C:T | G:A | A:A | C:G |
| 6401 | C:G | -:- | C:G | C:T | G:A | A:A | C:C |
| 6402 | C:C | -:C | C:C | C:T | G:G | C:C | C:G |
| 6403 | C:G | C:C | G:G | C:T | G:G | A:A | C:G |
| 6404 | C:C | -:C | C:G | C:T | G:G | A:A | C:G |
| 6405 | G:G | -:C | C:G | C:T | G:A | A:A | C:G |
| 6406 | C:C | -:- | C:G | C:C | G:A | A:C | C:C |
| 6407 | C:G | -:C | C:C | C:T | G:A | A:A | C:C |
| 6408 | C:G | -:- | C:C | C:T | G:A | C:C | G:G |
| 6409 | C:G | -:- | C:G | T:T | G:G | A:A | C:G |
| 6410 | C:C | -:- | C:G | C:C | G:G | A:C | C:C |
| 6411 | C:G | C:C | C:C | T:T | A:A | A:A | G:G |
| 6412 | G:G | C:C | G:G | ? | G:G | A:A | C:G |
| 6413 | G:G | -:C | C:C | T:T | A:A | A:A | C:C |
| 6414 | C:C | -:- | C:C | C:C | G:G | A:C | C:G |
| 6415 | C:C | -:C | C:C | C:C | G:G | A:C | C:G |
| 6416 | C:G | -:- | C:G | C:T | A:A | A:A | C:G |
| 6417 | C:G | -:- | C:G | C:T | G:A | A:A | G:G |
| 6418 | C:C | -:C | C:G | C:C | G:A | A:C | G:G |
| 6419 | C:C | -:C | C:G | C:C | G:G | A:A | C:G |
| 6421 | G:G | -:- | C:G | C:T | A:A | A:C | G:G |
| 6422 | C:G | -:C | C:G | C:T | G:A | A:A | C:G |
| 6423 | C:C | -:C | C:G | C:C | G:G | A:A | G:G |
| 6424 | C:G | -:C | C:G | C:C | G:A | A:C | C:G |
| 6425 | C:G | C:C | C:C | C:T | G:A | A:C | C:G |
| 6426 | C:G | -:C | C:C | ? | G:G | A:A | C:G |
| 6427 | C:C | -:- | C:G | C:C | G:A | A:C | C:G |
| 6428 | C:G | -:- | G:G | C:T | A:A | A:A | C:G |
| 6429 | ? | C:C | G:G | C:C | G:G | A:A | C:C |
| 6430 | C:G | C:C | C:G | C:T | G:G | A:A | C:C |
| 6431 | C:G | C:C | C:G | C:C | G:G | A:C | C:G |
| 6432 | C:G | -:C | C:C | T:T | G:A | A:A | C:C |

| | | | | | | | |
|---|---|---|---|---|---|---|---|
| 6433 | C:C | -:- | G:G | C:C | G:G | C:C | C:G |
| 6434 | C:C | C:C | G:G | C:T | G:A | A:C | C:G |
| 6435 | C:G | -:- | C:G | C:T | G:A | A:C | C:C |
| 6436 | G:G | C:C | C:G | T:T | A:A | A:C | G:G |
| 6437 | G:G | C:C | G:G | C:T | G:A | A:A | C:C |
| 6438 | C:G | -:C | C:G | C:C | G:G | A:C | C:G |
| 6439 | C:G | -:- | C:G | T:T | G:G | A:A | C:C |
| 6440 | C:G | -:C | G:G | C:T | G:A | A:A | C:C |
| 6441 | C:G | -:C | C:C | C:T | A:A | A:A | C:C |
| 6442 | C:G | -:C | C:C | C:T | G:G | C:C | C:G |
| 6443 | C:C | -:- | G:G | C:T | G:A | A:A | C:G |
| 6444 | C:C | -:- | G:G | C:C | G:A | A:C | C:G |
| 6445 | C:C | -:- | C:G | C:C | G:A | A:A | C:G |
| 6446 | C:G | -:- | C:G | C:T | A:A | C:C | G:G |
| 6447 | C:C | -:C | C:C | C:C | G:A | A:A | C:G |
| 6448 | C:G | -:- | G:G | C:C | G:A | A:A | C:C |
| 6449 | C:G | -:C | C:G | T:T | G:G | A:A | G:G |
| 6450 | C:G | -:C | C:C | C:T | G:G | A:C | ? |
| 6451 | C:C | -:- | G:G | C:T | G:G | A:C | C:G |
| 6452 | C:C | -:- | C:C | C:C | G:G | A:C | C:C |
| 6453 | C:C | -:- | C:G | C:C | G:G | A:A | C:G |
| 6454 | C:C | -:C | C:G | C:C | G:G | A:C | G:G |
| 6455 | C:C | -:C | G:G | C:C | G:G | A:A | C:G |
| 6456 | C:C | -:C | C:C | C:C | G:G | C:C | C:G |
| 6457 | G:G | -:C | C:G | C:T | G:A | A:C | C:G |
| 6458 | C:C | -:C | C:C | C:T | G:G | A:A | C:G |
| 6459 | G:G | -:- | C:G | C:T | G:G | A:A | C:C |
| 6460 | C:G | -:- | C:G | C:C | G:G | A:A | C:G |
| 6461 | C:G | -:C | C:G | C:C | G:A | C:C | G:G |
| 6462 | C:G | -:C | C:C | C:T | A:A | A:A | C:G |
| 6463 | C:C | -:- | C:G | C:C | G:G | A:C | C:G |
| 6464 | C:G | -:- | C:C | C:T | G:A | A:A | C:G |
| 6465 | C:C | -:- | C:G | C:C | G:G | A:A | G:G |
| 6466 | G:G | -:- | C:G | C:T | G:A | A:A | G:G |
| 6467 | C:C | -:C | C:G | C:C | G:G | A:C | C:C |
| 6468 | C:C | -:C | C:G | C:T | G:G | A:A | C:G |
| 6469 | C:G | C:C | ? | C:T | G:G | A:A | C:G |
| 6470 | C:G | -:C | C:G | C:T | G:A | A:A | C:G |
| 6471 | C:C | -:C | C:G | C:C | G:G | A:C | C:G |
| 6472 | C:C | -:- | C:G | C:C | G:A | A:A | C:G |
| 6473 | C:G | -:C | C:C | C:T | G:A | A:A | G:G |
| 6474 | C:G | -:- | G:G | C:C | G:G | A:A | G:G |
| 6475 | C:C | -:C | G:G | C:C | G:A | A:C | C:G |
| 6476 | C:G | -:- | C:G | C:C | G:A | C:C | C:G |
| 6477 | C:C | -:C | C:G | C:T | G:G | A:C | G:G |
| 6478 | C:C | -:- | C:G | C:C | G:G | A:C | C:C |
| 6479 | C:G | -:C | C:C | C:C | G:G | C:C | C:G |
| 6480 | C:C | -:C | C:G | C:C | G:G | A:A | G:G |
| 6481 | C:C | C:C | C:C | C:C | G:G | A:C | C:C |
| 6482 | C:C | -:- | C:G | C:C | G:A | A:C | G:G |

| | | | | | | |
|---|---|---|---|---|---|---|
| 6483 C:G | -:C | C:G | C:T | A:A | A:A | C:C |
| 6484 G:G | ? | C:G | C:C | G:G | C:C | C:G |
| 6485 C:C | -:- | C:C | C:C | G:A | A:A | G:G |
| 6486 C:C | -:C | C:G | C:C | G:G | C:C | C:C |
| 6487 C:C | -:- | G:G | C:C | G:G | A:C | C:C |
| 6488 C:C | -:- | C:C | C:C | A:A | A:A | C:C |
| 6489 C:G | -:C | G:G | C:T | G:A | A:A | C:G |
| 6490 C:C | C:C | C:C | C:C | G:A | A:A | C:C |
| 6491 C:C | -:C | G:G | C:C | G:A | A:C | C:G |
| 6492 C:G | -:C | C:G | C:T | G:A | A:A | C:C |
| 6493 G:G | C:C | C:C | T:T | G:A | A:A | C:C |
| 6494 C:C | -:C | G:G | C:C | G:A | A:A | G:G |
| 6495 C:G | -:- | C:C | C:T | G:A | C:C | C:G |
| 6496 C:C | -:C | G:G | C:C | A:A | A:A | C:G |
| 6497 C:G | -:C | C:C | T:T | G:A | A:A | G:G |
| 6498 C:G | -:- | C:G | C:C | G:G | A:A | G:G |
| 6499 C:G | -:C | C:G | C:T | G:A | A:A | C:C |
| 6500 C:G | -:C | C:C | C:T | G:A | A:A | C:C |
| 6501 C:C | C:C | G:G | C:C | G:G | A:C | C:C |
| 6502 C:G | C:C | C:G | C:C | A:A | A:A | C:G |
| 6503 C:C | -:- | G:G | C:T | G:G | A:C | C:G |
| 6504 C:C | -:C | C:C | C:C | G:G | A:C | C:C |
| 6505 G:G | -:- | C:G | C:T | A:A | A:C | ? |
| 6506 C:C | -:C | C:G | C:C | G:A | A:C | C:C |
| 6507 C:G | -:C | C:G | C:T | G:A | A:C | C:G |
| 6508 C:G | -:C | C:G | C:C | G:G | A:A | C:G |
| 6509 G:G | -:C | C:G | C:T | G:A | A:A | C:G |
| 6510 G:G | -:- | C:G | C:T | G:A | A:A | C:G |
| 6511 C:C | -:C | G:G | C:T | G:A | A:A | C:G |
| 6512 C:C | -:C | G:G | C:C | G:G | A:A | C:C |
| 6513 C:C | -:C | C:G | T:T | G:A | A:C | C:G |
| 6514 C:G | C:C | C:G | C:C | G:G | A:A | C:G |
| 6515 C:G | -:C | C:C | C:T | A:A | A:C | C:C |
| 6516 C:G | -:C | C:G | C:C | G:G | A:A | C:G |
| 6517 C:C | -:C | C:G | C:C | G:G | A:A | C:G |
| 6518 G:G | -:C | G:G | T:T | ? | A:A | C:G |
| 6519 C:G | -:- | C:C | C:T | G:A | A:C | G:G |
| 6520 C:C | -:C | C:G | C:T | G:A | A:C | C:G |
| 6521 C:G | -:C | G:G | C:T | G:A | C:C | C:G |
| 6522 C:C | -:- | C:C | C:C | G:G | A:A | G:G |
| 6523 C:C | -:C | C:G | C:C | G:G | C:C | C:G |
| 6524 C:C | -:- | C:G | C:C | A:A | A:C | C:G |
| 6525 C:G | -:C | C:G | C:T | A:A | A:A | C:G |
| 6526 C:G | -:- | C:C | C:T | G:A | A:A | C:G |
| 6527 C:C | -:- | G:G | C:C | G:A | A:A | C:G |
| 6528 C:C | -:- | G:G | C:C | G:G | A:A | C:G |
| 6529 G:G | -:C | C:C | C:T | G:A | A:C | C:G |
| 6530 C:G | -:C | G:G | T:T | G:G | A:C | G:G |
| 6531 C:G | -:C | C:G | C:C | G:G | A:A | C:G |
| 6532 C:C | -:C | C:C | C:C | G:G | A:C | C:C |

| | | | | | | | |
|---|---|---|---|---|---|---|---|
| 6533 | C:C | -:- | C:C | C:T | G:G | A:C | C:G |
| 6534 | G:G | -:- | C:C | C:T | G:A | A:C | C:G |
| 6535 | C:G | C:C | G:G | C:T | G:G | C:C | G:G |
| 6536 | C:G | -:C | C:G | T:T | G:A | A:A | C:G |
| 6537 | C:C | -:C | C:C | T:T | G:A | A:A | G:G |
| 6538 | C:G | -:C | C:C | C:C | G:A | A:C | G:G |
| 6539 | C:C | -:C | C:C | C:T | G:A | A:A | C:C |
| 6540 | C:G | C:C | C:C | C:C | G:A | A:C | C:G |
| 6541 | C:G | -:- | G:G | C:T | G:A | C:C | C:G |
| 6542 | C:C | -:- | C:G | C:T | G:G | A:C | G:G |
| 6543 | C:G | -:C | C:C | C:T | G:A | A:C | C:G |
| 6544 | C:C | -:C | G:G | C:C | G:A | C:C | C:G |
| 6545 | C:G | -:C | G:G | C:T | G:G | A:C | C:G |
| 6546 | C:G | -:- | C:G | C:T | G:A | A:C | G:G |
| 6547 | C:C | -:C | C:C | C:T | G:G | A:C | C:G |
| 6548 | C:G | -:- | C:G | C:T | G:A | A:A | C:G |
| 6549 | C:G | C:C | C:C | C:T | A:A | A:A | C:G |
| 6550 | G:G | -:- | G:G | C:C | G:A | A:C | C:G |
| 6551 | C:C | -:C | C:G | C:C | G:G | A:C | C:G |
| 6552 | C:C | -:- | C:G | C:T | G:G | A:A | G:G |
| 6553 | C:C | -:- | C:G | C:C | G:G | A:A | C:G |
| 6554 | G:G | -:C | C:G | C:T | G:A | A:C | C:G |
| 6555 | C:C | -:C | C:G | C:C | G:G | A:C | G:G |
| 6556 | C:G | -:C | C:G | T:T | G:A | A:A | C:C |
| 6557 | C:G | -:C | C:G | C:T | G:G | A:A | C:C |
| 6558 | C:G | -:C | C:G | C:C | G:G | A:A | G:G |
| 6559 | C:C | -:C | G:G | C:C | G:G | A:C | C:C |
| 6560 | C:G | -:- | C:G | ? | G:A | A:C | G:G |
| 6561 | C:C | -:C | C:C | C:T | G:A | A:C | C:G |
| 6562 | C:C | -:C | C:G | C:T | G:A | A:A | C:C |
| 6563 | C:C | -:C | G:G | T:T | G:G | A:C | C:G |
| 6564 | G:G | -:- | ? | T:T | A:A | A:C | C:G |
| 6565 | G:G | -:- | C:G | T:T | A:A | C:C | C:C |
| 6566 | C:C | -:C | G:G | C:C | G:G | C:C | C:C |
| 6567 | C:C | -:C | C:C | C:C | G:G | A:C | C:G |
| 6568 | C:G | -:- | C:G | C:T | G:A | A:C | C:G |
| 6569 | G:G | C:C | C:C | T:T | G:A | A:A | C:G |
| 6570 | C:G | -:- | C:G | C:T | G:G | A:C | C:G |
| 6571 | C:C | -:C | C:G | C:T | G:G | A:A | ? |
| 6572 | C:G | -:C | C:C | C:C | G:A | A:C | C:G |
| 6573 | G:G | -:- | C:G | T:T | G:A | A:A | C:C |
| 6574 | C:G | -:C | G:G | ? | G:G | A:A | C:C |
| 6575 | C:G | C:C | C:G | C:C | G:G | A:A | C:G |
| 6576 | C:C | -:C | C:G | C:T | G:A | A:A | C:C |
| 6577 | C:G | -:C | C:G | C:T | G:A | C:C | C:G |
| 6578 | C:C | -:C | C:C | C:C | G:A | A:A | C:C |
| 6579 | C:G | -:C | C:G | C:C | G:G | A:C | G:G |
| 6580 | G:G | -:- | C:C | T:T | G:A | A:C | G:G |
| 6581 | C:G | C:C | C:G | C:T | G:A | A:A | C:C |
| 6582 | C:G | -:- | C:C | C:C | G:G | A:A | C:G |

| | | | | | | | |
|---|---|---|---|---|---|---|---|
| 6583 | C:G | -:- | C:G | C:T | G:A | A:A | G:G |
| 6584 | C:G | -:C | G:G | C:T | G:A | A:C | C:G |
| 6585 | G:G | C:C | C:G | C:C | G:A | C:C | G:G |
| 6586 | C:G | -:C | C:G | C:T | G:A | A:C | C:G |
| 6587 | C:C | -:- | C:G | C:C | G:A | A:C | C:C |
| 6588 | C:G | -:- | C:C | C:C | G:A | A:C | C:G |
| 6589 | C:G | -:C | C:C | C:T | A:A | A:C | C:C |
| 6590 | G:G | C:C | C:G | C:T | G:A | A:C | G:G |
| 6591 | C:C | -:- | C:C | C:C | G:A | A:C | C:G |
| 6592 | C:G | -:C | G:G | C:T | G:A | A:C | C:G |
| 6593 | C:C | -:C | C:G | ? | G:G | A:C | C:C |
| 6594 | C:G | -:C | C:G | C:T | G:A | C:C | C:C |
| 6595 | C:C | -:- | C:G | C:T | G:G | C:C | C:G |
| 6596 | C:C | -:C | C:G | C:C | G:G | A:A | C:G |
| 6597 | C:G | -:C | C:G | C:C | G:A | A:C | C:G |
| 6598 | C:G | -:- | G:G | C:T | G:A | A:A | C:G |
| 6599 | C:G | C:C | C:C | C:T | G:A | A:C | C:C |
| 6600 | C:C | -:- | G:G | C:C | G:G | A:C | C:G |
| 6601 | G:G | -:- | C:G | C:T | G:A | A:A | C:G |
| 6602 | C:C | -:C | C:C | C:C | G:G | C:C | C:G |
| 6603 | C:G | -:C | C:C | C:C | G:A | A:C | C:G |
| 6604 | C:C | C:C | C:G | C:C | G:G | A:A | C:G |
| 6605 | C:G | -:- | C:G | C:C | G:G | C:C | G:G |
| 6606 | C:G | -:C | C:G | C:T | G:G | A:C | G:G |
| 6607 | C:C | C:C | G:G | C:T | G:G | A:A | C:G |
| 6608 | C:G | C:C | C:C | C:T | G:A | A:A | C:G |
| 6609 | G:G | -:C | G:G | C:C | G:A | A:C | C:G |
| 6610 | C:G | -:- | C:C | C:C | G:G | A:A | C:G |
| 6611 | G:G | -:C | C:G | T:T | A:A | A:A | C:G |
| 6612 | C:G | -:- | C:C | C:T | G:A | C:C | C:G |
| 6613 | C:G | -:C | C:G | T:T | G:A | A:C | C:G |
| 6614 | C:G | -:C | C:G | C:T | G:G | A:C | G:G |
| 6615 | C:G | -:C | C:G | C:T | G:A | C:C | G:G |
| 6616 | C:G | C:C | C:G | C:T | G:A | C:C | C:G |
| 6617 | C:C | -:- | C:G | C:C | G:A | A:C | C:C |
| 6618 | C:C | -:- | G:G | C:C | G:G | A:C | C:G |
| 6619 | C:C | -:C | C:G | C:C | G:G | A:C | G:G |
| 6620 | G:G | -:- | C:G | C:T | G:A | A:C | C:G |
| 6621 | ? | ? | G:G | C:C | ? | ? | G:G |
| 6622 | ? | ? | ? | ? | ? | ? | G:G |
| 6623 | C:C | -:- | ? | C:C | ? | ? | G:G |
| 6624 | ? | -:- | ? | ? | ? | ? | G:G |
| 6625 | ? | ? | ? | ? | ? | ? | G:G |
| NTC | NTC | NTC | NTC | NTC | NTC | NTC | NTC |

:h SNP on each well with a subject ID. When a subject ID is duplicated and the calls don't match t

| ganab_var1 | hmp19_var1 | msantd4_var1 | opa_var1 | plekhg1_var1 | slc25_var1 | sre_var1 |
|---|---|---|---|---|---|---|
| C:C | G:G | T:T | T:T | T:T | ? | T:T |
| C:C | G:G | T:T | T:T | T:T | ? | T:T |
| C:C | G:G | T:T | T:T | T:T | ? | T:T |
| C:C | G:G | T:T | T:T | T:T | ? | T:T |
| C:C | G:G | T:T | T:T | T:T | ? | T:T |
| C:C | G:G | T:T | T:T | T:T | ? | T:T |
| C:C | G:G | T:T | T:T | T:T | ? | T:T |
| C:C | G:G | T:T | T:T | T:T | A:A | T:T |
| C:C | G:G | T:T | T:T | T:T | ? | T:T |
| C:C | G:G | T:T | T:T | T:T | A:A | T:T |
| C:C | G:G | T:T | T:T | T:T | ? | T:T |
| C:C | G:G | T:T | T:T | T:T | ? | T:T |
| C:C | G:G | T:T | T:T | T:T | A:A | T:T |
| C:C | G:G | T:T | T:T | T:T | A:A | T:T |
| C:C | G:G | T:T | T:T | T:T | ? | T:T |
| C:C | G:G | T:T | T:T | T:T | ? | T:T |
| C:C | G:G | T:T | T:T | T:T | ? | T:T |
| C:C | G:G | T:T | T:T | T:T | ? | T:T |
| A:A | A:A | G:G | C:C | C:C | G:G | A:A |
| A:A | A:A | G:G | C:C | C:C | G:G | A:A |
| A:A | A:A | G:G | C:C | C:C | G:G | A:A |
| A:A | A:A | G:G | C:C | C:C | G:G | A:A |
| A:A | A:A | G:G | C:C | C:C | G:G | A:A |
| A:A | A:A | G:G | C:C | C:C | G:G | A:A |
| C:C | G:G | T:T | T:T | T:T | ? | T:T |
| C:C | G:G | T:T | T:T | T:T | ? | T:T |
| C:C | G:G | T:T | T:T | T:T | ? | T:T |
| A:A | A:A | G:G | C:C | C:C | G:G | A:A |
| A:A | A:A | G:G | C:C | C:C | G:G | A:A |
| A:A | A:A | G:G | C:C | C:C | G:G | A:A |
| A:A | A:A | G:G | C:C | C:C | G:G | A:A |
| A:A | A:A | G:G | C:C | C:C | G:G | A:A |
| A:A | A:A | G:G | C:C | C:C | G:G | A:A |
| A:A | A:A | G:G | C:C | C:C | G:G | A:A |
| A:A | A:A | G:G | C:C | C:C | G:G | A:A |
| A:A | A:A | G:G | C:C | C:C | G:G | A:A |
| A:A | A:A | G:G | C:C | C:C | G:G | A:A |
| A:A | A:A | G:G | C:C | C:C | G:G | A:A |
| A:A | A:A | G:G | C:C | C:C | G:G | A:A |
| A:A | A:A | G:G | C:C | C:C | G:G | A:A |

| | | | | | | |
|---|---|---|---|---|---|---|
| A:A | A:A | G:G | C:C | C:C | G:G | A:A |
| A:A | A:A | G:G | C:C | C:C | G:G | A:A |
| C:C | G:A | T:T | T:T | T:C | ? | T:T |
| C:A | G:G | T:G | T:T | T:C | A:A | T:A |
| C:C | G:G | T:T | T:C | T:T | A:G | T:A |
| A:A | G:G | T:T | T:C | T:C | A:G | T:A |
| C:A | G:G | T:G | T:C | T:C | A:G | T:T |
| C:C | G:A | G:G | T:C | T:T | A:A | T:A |
| C:C | G:G | G:G | C:C | T:T | A:G | T:A |
| C:C | G:G | G:G | T:C | T:C | A:G | T:T |
| C:C | G:A | G:G | T:C | T:C | ? | T:T |
| C:A | G:G | G:G | T:C | T:T | A:G | T:A |
| C:A | G:A | T:G | T:T | T:C | A:G | T:T |
| C:A | G:G | G:G | T:C | T:T | A:G | T:T |
| C:A | G:G | T:G | T:T | T:C | A:A | T:A |
| C:C | G:G | T:T | T:C | T:C | A:A | T:A |
| C:A | G:G | T:T | T:C | T:T | A:A | T:T |
| C:A | G:G | T:G | C:C | T:C | A:G | T:A |
| C:A | G:A | T:T | T:C | T:T | ? | A:A |
| C:A | G:G | T:T | T:C | ? | A:G | A:A |
| C:A | G:A | T:T | T:C | T:T | A:A | T:T |
| C:A | G:A | T:T | T:T | T:T | A:G | T:A |
| C:C | G:G | T:G | T:T | T:C | A:G | T:A |
| C:C | G:G | G:G | T:T | ? | A:A | T:T |
| C:C | A:A | G:G | T:C | C:C | A:G | T:A |
| C:A | G:G | T:G | T:T | T:C | A:A | T:A |
| A:A | G:G | T:T | T:T | C:C | A:A | T:A |
| C:C | G:A | T:G | C:C | T:C | A:G | T:T |
| C:C | G:G | T:T | T:C | T:C | G:G | T:T |
| C:A | G:G | T:T | T:C | T:T | A:A | T:A |
| C:A | G:A | T:G | T:T | C:C | A:G | T:A |
| C:C | G:G | T:G | C:C | T:C | A:A | T:A |
| C:A | G:A | G:G | T:C | T:C | A:A | T:A |
| C:A | G:G | T:T | T:C | T:C | G:G | T:T |
| C:A | G:A | G:G | T:T | T:C | ? | T:T |
| C:C | G:G | T:T | T:T | ? | ? | T:A |
| C:C | G:A | T:G | C:C | T:T | A:A | T:A |
| C:C | G:G | G:G | C:C | T:C | ? | T:A |
| C:A | G:G | T:T | C:C | T:C | ? | T:T |
| C:A | A:A | G:G | T:C | ? | ? | ? |
| A:A | G:G | T:G | C:C | T:C | ? | T:A |
| C:A | G:G | G:G | T:T | C:C | A:A | T:T |
| C:C | G:G | T:G | T:T | T:T | A:A | A:A |
| C:A | G:G | T:G | T:T | T:C | A:G | T:A |
| C:C | G:A | T:T | T:C | T:T | ? | T:A |
| C:C | G:G | T:G | C:C | C:C | ? | T:T |
| C:A | G:A | T:G | T:C | T:C | A:G | T:T |
| C:C | G:A | G:G | T:C | T:T | ? | T:A |
| C:A | G:A | G:G | T:C | T:C | A:G | A:A |
| C:C | G:G | T:T | T:T | T:T | A:G | T:T |

| | | | | | | |
|---|---|---|---|---|---|---|
| C:A | G:G | T:G | C:C | C:C | A:A | A:A |
| C:C | A:A | T:G | T:C | T:T | G:G | T:T |
| C:C | G:G | G:G | T:C | C:C | ? | T:A |
| C:C | G:A | T:G | C:C | ? | ? | T:T |
| C:C | G:A | T:G | T:C | T:T | ? | T:T |
| C:A | G:A | T:G | C:C | T:C | A:A | T:T |
| C:A | G:A | T:T | C:C | T:T | G:G | T:A |
| C:A | G:A | T:G | T:T | T:T | A:A | T:T |
| C:C | G:A | G:G | T:C | T:T | G:G | T:A |
| C:A | G:A | G:G | T:C | T:T | A:A | T:T |
| C:A | G:G | T:T | C:C | T:T | A:G | T:T |
| C:C | G:A | T:T | T:C | T:T | A:A | T:A |
| C:C | G:A | T:T | T:C | T:T | A:A | T:A |
| C:A | G:G | T:G | T:T | T:C | G:G | T:A |
| C:C | G:A | T:G | T:T | T:T | G:G | T:T |
| C:A | G:G | T:G | T:C | T:T | A:G | T:A |
| C:C | G:A | T:G | T:C | T:T | A:A | T:A |
| C:A | G:G | G:G | T:T | T:T | A:A | T:T |
| A:A | G:A | T:G | C:C | T:T | A:G | T:A |
| C:A | G:A | T:G | C:C | T:C | A:A | T:T |
| C:A | G:G | T:G | C:C | T:T | A:A | T:A |
| C:C | G:A | T:T | T:T | T:T | A:G | T:A |
| C:A | G:A | T:G | T:C | C:C | A:G | A:A |
| C:A | G:G | G:G | C:C | T:C | A:G | T:T |
| C:C | G:A | T:G | C:C | T:T | ? | T:A |
| C:C | G:G | T:G | T:C | T:C | A:G | T:A |
| C:C | G:A | T:G | T:C | T:T | A:A | T:A |
| C:A | G:G | T:G | C:C | T:C | A:G | A:A |
| C:A | G:A | T:G | C:C | T:T | A:A | A:A |
| C:C | G:A | T:G | C:C | T:T | A:A | A:A |
| C:A | G:A | T:T | T:T | T:C | G:G | T:A |
| C:A | G:A | T:G | C:C | T:C | A:G | T:T |
| C:C | A:A | T:G | T:C | T:C | A:A | T:T |
| C:A | G:A | G:G | T:C | T:T | G:G | T:A |
| C:C | G:G | T:T | T:C | T:T | A:G | T:T |
| C:C | G:G | G:G | T:C | T:T | A:A | T:A |
| C:C | G:A | T:G | T:C | C:C | A:G | T:T |
| C:C | G:A | T:T | T:T | T:C | A:A | T:A |
| C:A | G:A | T:T | T:T | T:C | A:A | T:T |
| C:C | G:G | T:G | T:C | T:T | A:A | T:T |
| C:A | G:G | T:G | T:C | T:C | A:A | T:T |
| C:A | G:A | T:G | T:T | T:C | A:G | T:T |
| C:C | G:G | G:G | T:C | C:C | G:G | T:A |
| C:C | G:G | T:G | T:T | T:C | A:G | T:A |
| C:C | G:G | T:G | T:T | T:T | G:G | T:A |
| C:A | G:G | G:G | T:C | T:T | A:A | T:T |
| C:C | G:G | T:G | T:T | T:T | A:G | T:T |
| C:A | G:G | G:G | T:C | T:C | G:G | T:T |
| ? | G:A | T:T | T:T | T:T | A:A | A:A |
| C:A | G:A | T:G | C:C | T:T | A:A | A:A |

| | | | | | | |
|---|---|---|---|---|---|---|
| C:C | G:A | T:T | T:C | T:C | A:A | T:A |
| C:A | G:A | T:T | C:C | ? | ? | T:T |
| C:C | G:A | T:T | C:C | C:C | A:A | T:T |
| C:A | G:G | T:G | C:C | T:T | A:A | T:A |
| C:C | G:A | T:G | T:C | C:C | A:G | T:A |
| C:C | G:A | G:G | T:C | T:C | A:G | T:T |
| C:C | G:G | T:G | T:T | T:T | A:G | T:T |
| C:C | G:A | G:G | C:C | T:T | G:G | T:A |
| C:C | G:G | T:G | T:T | T:T | G:G | T:A |
| C:C | G:G | T:T | T:C | T:C | A:A | T:T |
| C:C | G:G | T:T | C:C | T:C | A:G | T:T |
| C:A | A:A | T:G | T:T | T:C | G:G | T:T |
| C:C | G:A | T:G | T:T | T:T | A:A | T:A |
| C:A | G:G | G:G | T:C | T:T | A:A | T:T |
| C:C | G:G | G:G | C:C | T:T | A:A | T:A |
| C:A | G:G | T:T | C:C | C:C | G:G | T:A |
| C:A | G:A | G:G | T:C | T:T | A:G | A:A |
| C:A | G:A | T:G | T:C | T:T | ? | T:A |
| A:A | G:G | G:G | T:T | T:C | A:G | T:A |
| C:C | G:A | T:T | T:C | T:C | A:A | T:A |
| C:C | G:A | G:G | T:C | T:C | A:G | T:A |
| C:C | G:A | T:T | T:C | T:C | A:G | T:A |
| C:C | G:G | T:T | T:C | T:T | A:A | T:A |
| C:C | G:G | T:G | T:C | T:C | A:A | T:A |
| C:C | A:A | T:G | T:T | T:C | ? | T:T |
| C:C | G:A | T:G | T:C | C:C | A:G | T:A |
| ? | G:A | G:G | T:T | ? | ? | ? |
| C:A | G:A | G:G | T:C | ? | ? | A:A |
| C:A | G:A | T:G | T:T | ? | ? | T:T |
| C:A | G:G | T:G | T:T | T:C | A:G | T:A |
| A:A | G:G | T:G | T:C | T:T | A:G | T:A |
| C:A | G:G | T:G | T:C | T:T | A:A | A:A |
| C:C | G:A | T:G | T:C | T:T | A:A | T:T |
| C:A | G:A | T:T | T:C | T:T | ? | T:T |
| C:A | G:G | T:G | T:T | T:T | A:A | T:A |
| A:A | A:A | T:G | T:C | T:C | A:A | T:A |
| C:A | A:A | T:G | C:C | T:C | ? | T:T |
| C:C | G:G | T:G | T:C | T:T | A:G | T:T |
| C:A | G:G | G:G | T:T | T:T | A:G | A:A |
| C:C | G:A | T:G | T:C | T:T | A:G | T:A |
| C:C | A:A | T:T | T:C | ? | A:A | T:T |
| C:C | G:G | T:G | T:C | T:T | A:G | T:T |
| C:C | G:G | G:G | T:T | T:C | A:G | T:A |
| C:C | G:G | T:G | T:T | T:T | A:G | T:A |
| C:A | G:G | T:G | T:C | T:T | A:A | T:T |
| C:C | G:G | T:G | T:C | T:T | A:A | T:A |
| C:A | G:A | T:G | T:T | T:T | A:G | T:T |
| ? | G:A | T:G | T:C | ? | ? | T:A |
| C:C | G:G | T:G | T:T | C:C | G:G | T:A |
| C:C | G:G | T:T | C:C | ? | ? | A:A |

| | | | | | | |
|---|---|---|---|---|---|---|
| C:C | A:A | T:G | T:C | T:T | ? | T:T |
| C:C | G:G | T:G | C:C | T:T | A:G | T:A |
| C:A | G:A | T:G | T:C | T:C | A:G | T:A |
| C:C | G:G | T:G | T:T | T:T | A:G | A:A |
| C:C | G:G | T:T | C:C | T:C | A:A | T:A |
| C:C | G:A | T:G | T:C | T:T | A:A | T:T |
| A:A | G:G | T:T | T:T | ? | A:A | A:A |
| C:A | G:A | T:G | T:C | T:C | A:G | T:A |
| C:C | G:A | T:T | C:C | T:T | G:G | A:A |
| C:A | G:A | G:G | T:C | T:T | A:G | T:A |
| C:C | G:G | T:G | T:T | T:T | A:A | T:T |
| C:C | G:A | G:G | T:T | T:C | A:G | T:T |
| C:C | G:A | T:T | T:C | ? | ? | T:A |
| A:A | G:A | T:G | T:T | C:C | ? | T:A |
| ? | A:A | T:G | T:T | ? | ? | ? |
| C:C | G:G | T:G | T:C | T:T | ? | T:A |
| C:A | G:A | T:T | T:C | ? | A:A | A:A |
| ? | G:G | T:G | T:T | T:T | ? | T:A |
| C:A | A:A | T:G | T:T | C:C | A:A | T:T |
| C:C | A:A | T:T | C:C | T:C | A:A | T:T |
| C:C | A:A | T:G | T:C | T:C | G:G | T:T |
| C:C | G:A | T:G | C:C | T:C | A:G | T:T |
| C:C | G:G | G:G | T:C | T:C | G:G | T:T |
| C:C | G:A | T:G | T:C | T:T | A:G | T:A |
| C:C | G:G | G:G | T:C | T:C | A:G | A:A |
| C:A | G:A | T:G | C:C | T:T | A:A | T:A |
| C:C | G:G | G:G | T:T | C:C | G:G | T:A |
| C:C | G:G | T:T | T:C | T:C | A:G | T:T |
| C:A | G:G | T:G | ? | T:C | A:A | T:T |
| A:A | G:A | T:G | T:T | C:C | ? | T:A |
| C:C | G:G | T:G | T:T | T:C | A:G | T:T |
| C:C | G:G | T:G | T:C | T:C | A:A | T:A |
| A:A | G:A | G:G | T:C | T:T | G:G | T:T |
| C:C | G:G | T:G | C:C | T:T | A:A | T:A |
| C:A | G:G | G:G | C:C | T:T | A:G | T:T |
| C:A | A:A | T:G | T:C | T:T | A:A | T:A |
| A:A | G:A | G:G | T:C | T:C | G:G | T:T |
| C:C | G:A | T:T | C:C | T:C | G:G | T:A |
| C:A | G:A | G:G | T:T | T:C | A:A | T:T |
| C:C | G:A | T:G | C:C | T:C | A:G | T:T |
| C:A | G:G | T:G | T:C | T:T | A:A | T:A |
| C:C | G:G | T:T | T:T | T:T | A:A | T:T |
| C:C | G:G | G:G | T:C | T:T | A:G | T:T |
| C:A | G:A | G:G | T:C | T:T | A:A | T:A |
| C:C | G:A | T:G | T:T | T:C | G:G | T:A |
| C:A | G:G | G:G | T:C | T:C | G:G | T:T |
| A:A | G:G | T:T | T:T | T:C | G:G | T:T |
| C:A | G:A | G:G | T:C | T:T | A:G | T:T |
| C:C | G:A | G:G | T:C | C:C | A:A | T:T |
| C:A | G:A | T:G | T:T | T:T | A:A | T:T |

| | | | | | | |
|---|---|---|---|---|---|---|
| C:C | A:A | T:T | T:C | T:T | G:G | T:T |
| C:A | G:A | T:G | T:C | T:T | A:G | T:T |
| C:A | G:G | G:G | T:C | T:T | A:G | T:T |
| C:C | G:G | T:G | T:C | T:C | A:A | T:A |
| C:C | G:G | T:G | T:C | T:C | A:G | T:T |
| C:C | G:A | T:G | T:C | T:T | A:G | T:T |
| C:A | G:G | T:G | T:C | T:T | A:A | T:A |
| C:A | G:G | T:T | T:C | T:C | A:A | T:T |
| C:A | G:A | T:G | T:T | T:C | A:A | T:T |
| C:C | G:G | T:G | T:T | T:T | A:G | T:A |
| C:C | G:G | T:T | T:C | T:C | A:G | A:A |
| C:A | A:A | T:T | T:C | T:T | A:A | T:T |
| A:A | G:G | T:G | C:C | T:C | A:G | T:A |
| C:C | A:A | T:T | C:C | T:T | A:G | T:A |
| C:A | G:G | T:G | T:C | T:C | A:G | A:A |
| C:A | G:A | G:G | C:C | ? | ? | T:T |
| A:A | G:G | T:G | T:C | ? | ? | T:A |
| C:C | G:A | T:G | T:C | T:C | A:A | T:A |
| A:A | G:G | T:T | T:C | T:T | A:G | T:T |
| C:C | G:G | G:G | T:C | T:C | A:A | T:T |
| C:C | G:G | T:T | T:T | T:T | A:A | T:A |
| C:C | G:G | T:G | T:C | T:C | A:G | T:T |
| C:C | G:G | T:T | T:C | T:C | A:G | T:A |
| C:C | G:A | T:G | T:T | T:T | A:G | T:T |
| C:A | G:G | T:G | C:C | ? | ? | T:A |
| C:C | G:A | T:G | C:C | T:T | A:A | T:T |
| C:C | G:A | T:G | C:C | T:T | G:G | T:A |
| C:C | G:G | T:T | C:C | T:C | A:G | T:A |
| C:C | G:A | G:G | T:T | T:C | A:G | T:A |
| C:A | G:G | T:T | C:C | T:C | A:A | T:T |
| A:A | G:A | T:T | C:C | C:C | ? | T:A |
| C:C | G:A | T:G | T:C | T:C | A:G | T:T |
| A:A | G:A | T:T | T:T | T:T | A:A | A:A |
| C:C | G:A | T:T | T:C | T:T | A:A | T:T |
| C:C | G:G | G:G | T:T | T:T | A:A | T:T |
| C:C | G:A | T:T | T:C | T:T | A:G | T:A |
| C:C | G:A | G:G | T:T | T:C | A:A | A:A |
| C:C | G:A | T:G | C:C | T:T | A:G | T:A |
| C:C | A:A | T:G | T:T | T:C | A:A | T:A |
| A:A | A:A | T:T | C:C | T:T | G:G | T:A |
| C:C | G:G | T:G | T:C | T:C | A:G | T:T |
| C:A | G:A | T:G | T:C | T:T | A:G | T:A |
| C:C | G:A | T:G | C:C | T:T | A:G | T:A |
| C:C | G:G | G:G | T:C | T:C | A:G | T:A |
| C:C | G:A | G:G | T:C | T:T | A:A | ? |
| C:C | G:G | G:G | T:C | T:T | A:A | T:T |
| C:A | G:G | G:G | C:C | T:C | A:A | T:A |
| C:A | G:G | G:G | T:C | T:C | A:G | A:A |
| C:A | A:A | T:T | C:C | T:T | A:A | T:T |
| A:A | G:A | T:G | T:C | T:C | G:G | T:T |

| | | | | | | |
|---|---|---|---|---|---|---|
| C:C | G:G | T:T | T:T | T:C | A:G | T:A |
| C:C | G:A | T:G | T:C | T:C | A:A | T:A |
| C:C | A:A | G:G | T:C | T:T | A:A | T:A |
| C:C | G:G | T:T | T:C | T:C | A:G | A:A |
| C:A | G:G | T:G | T:T | T:C | A:G | T:T |
| C:C | G:A | G:G | T:C | T:C | A:G | A:A |
| C:C | G:G | T:G | T:T | T:T | A:A | T:A |
| A:A | G:G | T:G | T:T | T:C | A:A | T:A |
| C:C | G:A | T:G | C:C | T:T | A:G | T:A |
| C:A | A:A | T:G | C:C | T:T | A:A | T:A |
| C:C | G:G | T:G | T:C | T:T | A:G | T:A |
| C:C | G:A | T:T | T:T | T:T | A:A | T:T |
| C:C | G:G | T:T | T:C | T:T | A:A | T:A |
| C:C | G:G | T:G | T:C | T:C | A:G | T:A |
| C:A | A:A | T:T | T:C | T:C | G:G | T:T |
| C:A | G:A | G:G | T:C | T:C | A:A | T:A |
| C:C | G:A | G:G | T:C | T:C | ? | T:A |
| C:C | G:G | G:G | T:T | T:C | A:G | T:A |
| C:C | G:G | T:G | T:C | T:C | A:G | T:A |
| C:C | G:A | G:G | T:C | C:C | A:A | T:A |
| C:A | G:G | T:T | C:C | T:T | A:A | T:T |
| C:C | G:G | T:T | T:C | T:T | A:A | T:A |
| C:C | G:G | G:G | T:T | T:T | A:G | T:T |
| A:A | G:G | G:G | T:T | T:T | A:A | T:A |
| C:A | G:G | T:T | T:T | T:C | A:A | T:A |
| C:C | G:G | G:G | T:C | T:T | A:G | T:T |
| C:C | G:G | G:G | T:T | T:T | A:A | T:T |
| C:A | G:G | T:T | T:T | T:C | G:G | ? |
| C:C | G:A | T:T | T:C | T:C | A:G | T:A |
| C:C | G:G | T:T | C:C | T:C | A:G | T:A |
| C:A | A:A | T:G | C:C | T:C | A:G | T:A |
| C:C | G:A | T:G | C:C | C:C | A:A | A:A |
| C:A | G:G | T:G | T:T | T:C | A:A | A:A |
| C:C | G:G | T:T | T:C | T:C | G:G | T:T |
| C:C | G:G | T:T | T:C | T:T | A:A | T:T |
| C:C | G:G | T:G | T:T | T:C | G:G | T:A |
| C:A | A:A | T:G | T:T | T:C | A:G | T:A |
| C:C | G:G | T:G | T:T | T:T | A:G | T:A |
| C:A | G:G | T:G | T:C | T:C | A:G | T:A |
| C:C | G:G | T:G | T:T | T:C | A:A | T:T |
| ? | G:A | T:G | C:C | ? | A:A | A:A |
| C:A | G:G | T:T | T:C | T:C | ? | A:A |
| C:C | G:A | T:G | T:C | T:C | A:G | T:T |
| C:C | G:A | G:G | T:C | T:T | A:G | T:A |
| C:A | G:G | T:G | T:C | T:C | A:A | A:A |
| C:A | G:A | T:G | T:T | T:C | G:G | T:T |
| C:C | G:A | T:G | T:C | T:T | ? | T:T |
| C:C | A:A | G:G | T:T | T:T | A:A | T:A |
| C:A | G:G | T:G | T:C | T:C | A:A | T:A |
| C:A | G:A | T:G | T:C | T:T | A:G | T:T |

| | | | | | | |
|---|---|---|---|---|---|---|
| C:C | G:A | G:G | T:C | ? | ? | T:A |
| C:C | G:G | G:G | T:T | T:C | A:G | T:T |
| C:A | G:G | T:G | T:C | C:C | A:G | T:A |
| C:A | G:A | T:G | T:T | T:T | A:A | T:A |
| C:C | A:A | T:G | C:C | T:C | A:A | T:T |
| C:A | G:A | G:G | C:C | T:C | A:G | T:A |
| C:A | G:A | T:G | T:T | T:C | A:G | T:A |
| A:A | G:G | T:G | C:C | T:C | A:G | T:A |
| C:A | G:A | T:T | T:T | T:T | ? | T:A |
| C:A | G:A | T:G | C:C | T:T | A:A | T:A |
| C:A | G:A | T:T | T:C | ? | G:G | T:A |
| C:C | G:A | T:G | C:C | T:C | A:A | T:A |
| C:C | G:G | T:G | T:C | T:T | A:G | T:A |
| C:A | G:A | T:T | T:T | T:T | G:G | T:A |
| C:C | G:G | T:G | T:T | C:C | A:G | T:A |
| A:A | G:A | T:G | C:C | T:C | A:A | T:A |
| C:A | A:A | T:G | T:T | C:C | A:A | T:A |
| C:A | G:G | T:G | T:T | T:C | A:A | T:T |
| C:C | G:A | T:G | T:T | T:T | G:G | T:A |
| C:C | G:A | G:G | T:T | T:C | A:G | T:T |
| C:A | G:G | G:G | T:T | T:T | G:G | T:A |
| C:A | G:A | T:T | T:C | T:T | G:G | T:T |
| C:C | ? | T:G | T:T | T:T | A:A | T:T |
| C:A | G:G | T:G | T:C | T:T | A:A | T:A |
| C:A | G:A | T:T | T:C | T:C | A:A | T:A |
| C:C | G:G | G:G | T:T | T:C | A:A | T:A |
| C:A | G:G | T:G | T:T | T:T | A:G | T:A |
| C:C | G:G | T:T | C:C | C:C | G:G | T:T |
| C:C | G:A | G:G | T:C | T:T | A:A | A:A |
| A:A | G:G | T:G | C:C | T:C | A:G | T:A |
| C:C | A:A | G:G | T:C | T:T | ? | A:A |
| C:C | G:A | G:G | T:C | C:C | A:G | T:A |
| C:A | A:A | T:G | T:T | C:C | A:G | T:A |
| C:A | G:A | T:G | T:C | T:C | ? | T:A |
| C:C | G:A | T:G | T:C | T:C | A:A | T:T |
| C:C | G:G | T:G | T:T | T:T | G:G | T:T |
| C:A | G:G | G:G | C:C | T:T | G:G | T:T |
| C:C | G:A | G:G | T:C | C:C | A:G | T:T |
| ? | ? | G:G | ? | ? | G:G | ? |
| ? | ? | ? | ? | ? | G:G | ? |
| ? | ? | ? | ? | ? | G:G | ? |
| ? | ? | ? | ? | ? | G:G | ? |
| ? | ? | ? | ? | ? | G:G | ? |
| NTC | NTC | NTC | NTC | NTC | NTC | NTC |

:he keyword DUPE is used.

| supt6h_var1 | usp_var1 | wiz_var1 | ND4_var12 |
|---|---|---|---|
| C:C | G:G | C:C | T:T |
| C:C | G:G | C:C | T:T |
| C:C | G:G | C:C | T:T |
| C:C | G:G | C:C | T:T |
| C:C | G:G | C:C | T:T |
| C:C | G:G | C:C | T:T |
| C:C | G:G | C:C | T:T |
| C:C | G:G | C:C | T:T |
| C:C | G:G | C:C | T:T |
| C:C | G:G | C:C | T:T |
| C:C | G:G | C:C | T:T |
| C:C | G:G | C:C | T:T |
| C:C | G:G | C:C | T:T |
| C:C | G:G | C:C | T:T |
| C:C | G:G | C:C | T:T |
| C:C | G:G | C:C | T:T |
| C:C | G:G | C:C | T:T |
| C:C | G:G | C:C | T:T |
| A:A | A:A | T:T | C:C |
| A:A | A:A | T:T | C:C |
| A:A | A:A | T:T | C:C |
| A:A | A:A | T:T | C:C |
| A:A | A:A | T:T | C:C |
| A:A | A:A | T:T | C:C |
| ? | G:G | C:C | T:T |
| C:C | G:G | C:C | T:T |
| C:C | G:G | C:C | T:T |
| A:A | A:A | T:T | C:C |
| A:A | A:A | T:T | C:C |
| A:A | A:A | T:T | C:C |
| A:A | A:A | T:T | C:C |
| A:A | A:A | T:T | C:C |
| A:A | A:A | T:T | C:C |
| A:A | A:A | T:T | C:C |
| A:A | A:A | T:T | C:C |
| A:A | A:A | T:T | C:C |
| A:A | A:A | T:T | C:C |
| A:A | A:A | T:T | C:C |
| A:A | A:A | T:T | C:C |
| A:A | A:A | T:T | C:C |

| | | | |
|---|---|---|---|
| A:A | A:A | T:T | C:C |
| A:A | A:A | T:T | C:C |
| C:A | G:G | C:C | T:T |
| C:A | G:G | C:C | T:T |
| C:C | G:G | C:T | T:T |
| C:A | G:A | C:C | T:T |
| C:A | A:A | C:C | T:T |
| C:A | G:G | C:C | T:T |
| C:A | G:G | C:T | T:T |
| C:A | G:A | C:T | T:T |
| C:C | G:A | C:C | T:T |
| A:A | G:G | C:C | ? |
| C:C | G:G | C:T | T:T |
| C:C | G:G | C:T | ? |
| C:A | G:A | C:C | ? |
| A:A | G:A | C:C | T:T |
| C:A | G:A | C:C | T:T |
| C:C | G:G | C:T | T:T |
| A:A | G:A | C:T | ? |
| A:A | ? | C:C | ? |
| C:A | G:G | C:C | T:T |
| C:A | G:A | C:T | T:T |
| C:A | G:G | T:T | T:T |
| C:A | G:G | C:C | T:T |
| C:C | G:A | C:C | T:T |
| C:C | G:G | C:C | T:T |
| C:A | G:G | C:C | ? |
| C:C | G:G | T:T | T:T |
| C:C | G:A | C:T | T:T |
| C:A | G:G | C:C | ? |
| A:A | G:A | C:C | ? |
| A:A | G:A | C:C | T:T |
| C:A | G:A | C:T | T:T |
| C:A | G:A | C:C | T:T |
| C:A | G:A | C:C | ? |
| C:A | G:G | ? | ? |
| C:C | G:G | C:T | ? |
| C:A | G:G | C:C | ? |
| C:A | G:G | C:C | T:T |
| ? | ? | ? | ? |
| C:C | G:G | C:T | ? |
| A:A | G:G | C:C | T:T |
| C:A | G:A | C:C | T:T |
| C:C | G:G | C:C | T:T |
| A:A | G:G | C:C | T:T |
| A:A | G:A | C:C | ? |
| C:C | G:A | C:C | T:T |
| A:A | G:A | C:C | T:T |
| C:A | G:A | C:C | T:T |
| C:C | G:A | C:C | T:T |

| | | | |
|---|---|---|---|
| C:C | G:G | C:T | T:T |
| A:A | G:G | C:C | ? |
| A:A | G:A | C:T | T:T |
| C:C | G:A | C:C | T:T |
| C:A | G:A | C:C | ? |
| C:C | G:G | T:T | T:T |
| C:A | A:A | C:C | T:T |
| C:A | A:A | C:C | T:T |
| C:C | G:A | C:T | T:T |
| C:C | G:G | ? | T:T |
| C:A | G:G | C:C | T:T |
| C:C | G:G | C:C | ? |
| C:C | G:G | C:C | T:T |
| C:A | G:G | C:C | T:T |
| A:A | G:G | C:C | ? |
| C:A | G:A | C:C | T:T |
| C:C | G:A | C:C | T:T |
| C:A | G:G | C:C | T:T |
| C:C | G:A | C:T | ? |
| C:A | A:A | ? | ? |
| C:A | G:G | C:C | T:T |
| C:A | G:A | C:T | ? |
| C:A | G:G | T:T | T:T |
| A:A | G:G | C:T | T:T |
| A:A | G:A | C:C | T:T |
| A:A | G:G | C:C | ? |
| C:A | G:G | C:C | T:T |
| C:A | G:G | C:T | T:T |
| C:C | G:G | C:C | T:T |
| C:A | G:A | C:C | T:T |
| C:C | G:G | C:C | T:T |
| C:A | G:G | C:T | T:T |
| C:A | G:G | T:T | T:T |
| C:C | G:G | C:C | T:T |
| C:C | G:A | C:T | T:T |
| C:C | G:A | C:T | T:T |
| C:C | G:G | C:T | T:T |
| C:A | G:G | C:C | T:T |
| C:A | G:G | C:T | T:T |
| C:C | G:G | C:C | T:T |
| C:C | G:G | C:C | T:T |
| C:C | G:G | C:C | T:T |
| C:A | G:G | C:C | T:T |
| A:A | G:G | C:C | T:T |
| C:A | G:A | C:C | T:T |
| C:A | G:A | C:C | T:T |
| C:C | G:A | C:T | T:T |
| C:C | G:G | C:T | T:T |
| C:A | G:G | C:C | T:T |
| C:C | G:G | C:C | T:T |

| | | | |
|---|---|---|---|
| C:C | G:A | C:C | T:T |
| C:C | ? | ? | ? |
| C:C | G:G | C:C | ? |
| C:A | G:G | ? | ? |
| C:A | G:G | ? | ? |
| C:A | G:A | C:T | T:T |
| C:A | G:G | C:C | T:T |
| C:C | G:G | ? | ? |
| C:A | G:A | T:T | T:T |
| C:A | G:G | C:C | T:T |
| C:A | G:A | C:C | T:T |
| A:A | G:G | C:C | ? |
| C:C | G:A | C:C | T:T |
| C:C | G:G | C:T | T:T |
| C:A | G:A | C:T | T:T |
| C:C | A:A | C:T | T:T |
| C:A | A:A | T:T | T:T |
| C:C | G:G | C:C | ? |
| C:A | G:G | C:C | T:T |
| A:A | G:G | C:C | ? |
| C:A | G:G | ? | T:T |
| C:A | G:A | C:C | T:T |
| A:A | A:A | C:C | T:T |
| C:A | G:A | C:T | T:T |
| C:A | G:G | C:C | T:T |
| A:A | G:G | C:T | T:T |
| ? | ? | ? | ? |
| A:A | ? | ? | ? |
| ? | ? | ? | ? |
| C:C | G:G | C:C | T:T |
| C:A | G:G | C:T | ? |
| C:C | G:A | C:C | T:T |
| C:C | G:G | ? | ? |
| A:A | ? | C:C | ? |
| C:A | G:A | ? | ? |
| C:C | G:A | C:T | ? |
| C:C | G:G | C:T | ? |
| C:A | G:A | C:C | ? |
| C:A | G:A | C:C | ? |
| C:A | G:A | C:T | T:T |
| C:A | G:A | C:T | ? |
| C:A | G:G | C:C | ? |
| C:A | G:G | C:C | T:T |
| C:C | G:G | C:C | T:T |
| C:C | G:G | C:C | T:T |
| C:C | G:A | C:C | T:T |
| C:A | G:G | C:T | T:T |
| C:A | ? | ? | ? |
| C:A | G:G | C:C | ? |
| C:A | G:A | C:C | ? |

| | | | |
|---|---|---|---|
| C:A | G:G | ? | ? |
| A:A | G:G | C:C | T:T |
| C:A | A:A | C:C | T:T |
| C:A | G:G | C:C | ? |
| C:C | G:G | C:C | T:T |
| C:C | G:G | C:C | ? |
| A:A | ? | ? | ? |
| A:A | G:G | C:T | T:T |
| C:C | G:G | C:T | T:T |
| C:A | G:G | C:C | T:T |
| C:C | G:G | C:C | T:T |
| C:C | G:A | C:C | T:T |
| ? | G:A | C:C | ? |
| C:A | ? | ? | ? |
| ? | ? | ? | ? |
| C:C | G:G | C:C | ? |
| C:C | G:G | ? | ? |
| A:A | G:A | ? | ? |
| A:A | G:G | C:C | T:T |
| C:C | G:G | C:T | ? |
| C:C | G:G | ? | T:T |
| C:C | G:G | C:C | T:T |
| C:A | G:A | ? | ? |
| C:A | G:G | C:C | ? |
| C:A | G:G | C:C | T:T |
| C:C | G:A | C:C | ? |
| C:A | G:A | ? | ? |
| C:A | G:A | C:C | T:T |
| C:A | G:G | C:C | T:T |
| A:A | G:G | C:C | T:T |
| C:A | G:G | C:C | ? |
| C:A | G:A | C:C | T:T |
| C:C | G:A | C:C | T:T |
| A:A | G:G | C:C | ? |
| C:A | G:A | C:T | T:T |
| C:C | G:G | C:C | T:T |
| C:C | G:G | C:C | T:T |
| A:A | G:G | C:C | T:T |
| C:A | G:G | C:T | T:T |
| C:A | G:G | C:C | T:T |
| C:C | G:A | C:C | T:T |
| C:A | G:G | C:C | T:T |
| C:A | G:A | C:T | T:T |
| C:C | G:A | C:C | T:T |
| C:C | G:G | C:C | T:T |
| C:A | G:A | C:C | T:T |
| A:A | G:G | C:C | T:T |
| C:C | G:A | C:C | T:T |
| C:A | A:A | C:C | T:T |
| A:A | G:A | C:C | T:T |

| | | | |
|---|---|---|---|
| C:C | G:G | C:T | T:T |
| A:A | G:A | C:C | T:T |
| C:A | G:G | C:T | T:T |
| ? | G:G | C:C | T:T |
| C:A | G:G | C:C | T:T |
| C:C | G:G | C:C | ? |
| C:A | G:A | C:C | T:T |
| C:A | G:A | T:T | T:T |
| C:A | G:G | C:C | T:T |
| C:C | G:G | C:C | T:T |
| C:A | G:G | C:C | T:T |
| A:A | G:G | C:C | T:T |
| C:C | G:G | C:C | T:T |
| C:A | G:G | C:C | T:T |
| C:C | G:G | C:C | T:T |
| C:C | G:G | C:C | ? |
| C:A | ? | ? | ? |
| A:A | G:G | C:T | T:T |
| A:A | G:G | C:C | T:T |
| C:A | G:A | T:T | T:T |
| A:A | G:G | C:C | T:T |
| C:A | G:A | C:C | T:T |
| C:A | G:G | C:T | T:T |
| C:C | G:G | C:C | T:T |
| C:C | G:G | C:C | ? |
| C:C | G:A | C:T | T:T |
| A:A | G:A | C:C | T:T |
| C:A | G:G | C:C | T:T |
| C:C | A:A | C:T | T:T |
| C:A | G:A | C:T | ? |
| C:A | G:G | C:C | T:T |
| C:A | G:A | T:T | T:T |
| A:A | G:A | C:C | T:T |
| C:A | G:G | C:T | T:T |
| C:A | G:A | C:C | T:T |
| A:A | G:G | C:C | ? |
| C:A | G:G | C:T | T:T |
| C:C | G:A | C:T | T:T |
| C:A | G:A | C:C | T:T |
| C:C | G:G | C:C | T:T |
| C:A | G:G | C:T | T:T |
| C:A | G:A | C:C | T:T |
| C:A | G:A | C:C | T:T |
| C:C | G:G | C:T | T:T |
| C:A | G:A | C:T | ? |
| C:C | G:G | C:C | T:T |
| C:A | G:G | C:C | T:T |
| C:C | G:A | C:C | T:T |
| C:C | G:A | C:T | T:T |
| C:C | G:G | T:T | T:T |

| | | | |
|---|---|---|---|
| C:C | A:A | C:T | T:T |
| C:A | G:G | C:C | T:T |
| C:A | G:A | C:C | T:T |
| A:A | G:A | C:T | T:T |
| C:A | G:G | C:C | T:T |
| C:C | G:A | C:T | T:T |
| A:A | G:A | C:T | T:T |
| C:A | G:A | C:C | T:T |
| A:A | G:A | C:C | T:T |
| C:C | G:G | C:C | T:T |
| C:A | G:G | C:C | T:T |
| C:A | G:G | C:C | T:T |
| C:A | G:G | C:C | T:T |
| C:C | G:G | C:C | T:T |
| A:A | G:A | C:T | T:T |
| A:A | G:G | C:C | T:T |
| C:C | G:G | C:T | T:T |
| C:A | G:G | C:T | T:T |
| C:A | G:A | C:C | T:T |
| C:C | G:G | C:T | T:T |
| C:C | G:G | C:C | T:T |
| C:A | G:G | C:T | T:T |
| C:A | G:G | C:C | T:T |
| C:C | G:G | C:C | T:T |
| C:A | G:A | C:T | T:T |
| C:C | G:G | C:C | T:T |
| C:A | A:A | C:C | T:T |
| A:A | G:A | C:T | T:T |
| C:A | G:G | C:C | T:T |
| C:A | G:G | C:C | T:T |
| C:C | A:A | C:C | T:T |
| C:A | G:G | C:C | T:T |
| A:A | G:A | C:T | T:T |
| C:A | G:A | C:T | T:T |
| C:C | G:G | C:C | T:T |
| A:A | G:A | C:C | T:T |
| A:A | G:G | C:C | T:T |
| C:A | A:A | C:C | ? |
| C:C | G:G | C:C | T:T |
| C:A | G:A | C:C | T:T |
| C:C | G:G | C:C | ? |
| C:A | G:G | C:C | T:T |
| C:A | G:A | T:T | ? |
| C:A | G:G | C:T | ? |
| C:C | G:A | C:C | T:T |
| A:A | A:A | C:T | T:T |
| C:C | A:A | C:C | T:T |
| C:A | ? | C:C | T:T |
| C:A | G:A | C:T | T:T |
| C:A | G:G | C:C | T:T |

| | | | |
|---|---|---|---|
| C:A | G:G | ? | ? |
| C:C | G:A | C:C | ? |
| C:A | G:G | C:T | T:T |
| C:A | G:A | C:C | T:T |
| C:A | G:G | C:C | T:T |
| C:C | G:A | C:T | T:T |
| C:A | G:G | C:C | T:T |
| C:A | G:A | C:C | T:T |
| C:C | A:A | C:C | T:T |
| A:A | G:A | C:T | T:T |
| C:A | G:G | C:C | T:T |
| C:C | G:G | C:C | T:T |
| C:C | G:G | T:T | T:T |
| C:A | G:G | C:C | T:T |
| C:A | G:G | T:T | ? |
| C:C | G:A | C:C | T:T |
| C:C | G:G | C:C | T:T |
| C:C | G:A | ? | ? |
| C:A | G:A | C:C | T:T |
| C:A | G:G | C:C | T:T |
| C:C | G:G | C:T | T:T |
| C:C | G:A | C:T | T:T |
| C:A | G:A | C:C | T:T |
| C:C | G:G | C:T | T:T |
| C:C | G:A | C:C | T:T |
| C:C | G:G | C:C | T:T |
| A:A | G:G | C:C | T:T |
| C:C | G:G | C:C | T:T |
| C:A | G:A | C:C | T:T |
| C:C | G:G | C:C | T:T |
| C:C | G:A | C:C | T:T |
| C:A | G:G | C:C | ? |
| C:C | G:A | C:C | T:T |
| C:C | G:A | C:C | ? |
| C:A | G:A | C:C | T:T |
| C:C | G:A | C:C | ? |
| C:A | G:G | T:T | T:T |
| C:A | G:G | C:C | T:T |
| ? | A:A | T:T | T:T |
| ? | ? | ? | T:T |
| ? | ? | ? | T:T |
| ? | ? | ? | T:T |
| ? | ? | ? | ? |
| NTC | NTC | NTC | NTC |

KBiosciences genotyping report

| | |
|---|---|
| Export version | 1.21 |
| Customer | Naturalis - Naturalis |
| Customer number | 7 |
| Project number | 7.004 |
| Master plate type | 96 |
| Order number | |
| View mode | auto |
| Viewing | Please download SNPViewer to graphically view this file c |
| Editing | This file maybe graphically edited and re-called using LGC |
| Title | Ben Wielstra Sheffiled project |

Statistics

| SNP | Plate | Y | Het | X | NTC |
|---|---|---|---|---|---|
| arh_var1 | Vlasi-1 | 37 | 48 | 11 | 0 |
| arh_var1 | Vlasi-2 | 40 | 46 | 9 | 0 |
| arh_var1 | Vlasi-3 | 45 | 38 | 13 | 0 |
| arh_var1 | Vlasi-4 | 36 | 26 | 29 | 1 |
| clasp2_var1 | Vlasi-1 | 39 | 38 | 19 | 0 |
| clasp2_var1 | Vlasi-2 | 37 | 45 | 14 | 0 |
| clasp2_var1 | Vlasi-3 | 30 | 55 | 10 | 0 |
| clasp2_var1 | Vlasi-4 | 40 | 22 | 30 | 1 |
| col18_var1 | Vlasi-1 | 13 | 54 | 28 | 0 |
| col18_var1 | Vlasi-2 | 31 | 45 | 19 | 0 |
| col18_var1 | Vlasi-3 | 29 | 44 | 22 | 0 |
| col18_var1 | Vlasi-4 | 32 | 29 | 29 | 1 |
| ddx17_var1 | Vlasi-1 | 41 | 45 | 10 | 0 |
| ddx17_var1 | Vlasi-2 | 40 | 45 | 9 | 0 |
| ddx17_var1 | Vlasi-3 | 43 | 40 | 12 | 0 |
| ddx17_var1 | Vlasi-4 | 43 | 22 | 25 | 1 |
| dnaj_var1 | Vlasi-1 | 48 | 40 | 7 | 0 |
| dnaj_var1 | Vlasi-2 | 42 | 44 | 10 | 0 |
| dnaj_var1 | Vlasi-3 | 40 | 44 | 11 | 0 |
| dnaj_var1 | Vlasi-4 | 38 | 29 | 23 | 1 |
| fam178_var1 | Vlasi-1 | 39 | 42 | 15 | 0 |
| fam178_var1 | Vlasi-2 | 54 | 34 | 8 | 0 |
| fam178_var1 | Vlasi-3 | 43 | 42 | 11 | 0 |
| fam178_var1 | Vlasi-4 | 37 | 23 | 30 | 1 |
| gak_var1 | Vlasi-1 | 17 | 55 | 23 | 0 |
| gak_var1 | Vlasi-2 | 21 | 56 | 18 | 0 |
| gak_var1 | Vlasi-3 | 24 | 51 | 19 | 0 |
| gak_var1 | Vlasi-4 | 32 | 27 | 36 | 1 |
| ganab_var1 | Vlasi-1 | 45 | 47 | 4 | 0 |
| ganab_var1 | Vlasi-2 | 53 | 30 | 8 | 0 |
| ganab_var1 | Vlasi-3 | 58 | 28 | 10 | 0 |
| ganab_var1 | Vlasi-4 | 42 | 23 | 24 | 1 |
| hmp19_var1 | Vlasi-1 | 50 | 42 | 4 | 0 |
| hmp19_var1 | Vlasi-2 | 43 | 42 | 11 | 0 |
| hmp19_var1 | Vlasi-3 | 52 | 33 | 11 | 0 |
| hmp19_var1 | Vlasi-4 | 38 | 25 | 26 | 1 |

| | | | | | |
|---|---|---|---|---|---|
| msantd4_var1 | Vlasi-1 | 26 | 45 | 25 | 0 |
| msantd4_var1 | Vlasi-2 | 22 | 52 | 22 | 0 |
| msantd4_var1 | Vlasi-3 | 30 | 42 | 24 | 0 |
| msantd4_var1 | Vlasi-4 | 28 | 28 | 35 | 1 |
| opa_var1 | Vlasi-1 | 28 | 45 | 23 | 0 |
| opa_var1 | Vlasi-2 | 29 | 46 | 20 | 0 |
| opa_var1 | Vlasi-3 | 28 | 48 | 20 | 0 |
| opa_var1 | Vlasi-4 | 39 | 20 | 31 | 1 |
| plekhg1_var1 | Vlasi-1 | 42 | 39 | 10 | 0 |
| plekhg1_var1 | Vlasi-2 | 44 | 32 | 9 | 0 |
| plekhg1_var1 | Vlasi-3 | 41 | 48 | 4 | 0 |
| plekhg1_var1 | Vlasi-4 | 40 | 19 | 28 | 1 |
| slc25_var1 | Vlasi-1 | 35 | 33 | 12 | 0 |
| slc25_var1 | Vlasi-2 | 35 | 32 | 12 | 0 |
| slc25_var1 | Vlasi-3 | 39 | 41 | 11 | 0 |
| slc25_var1 | Vlasi-4 | 21 | 16 | 35 | 1 |
| sre_var1 | Vlasi-1 | 40 | 46 | 9 | 0 |
| sre_var1 | Vlasi-2 | 37 | 45 | 12 | 0 |
| sre_var1 | Vlasi-3 | 37 | 48 | 9 | 0 |
| sre_var1 | Vlasi-4 | 36 | 28 | 26 | 1 |
| supt6h_var1 | Vlasi-1 | 34 | 44 | 17 | 0 |
| supt6h_var1 | Vlasi-2 | 33 | 45 | 14 | 0 |
| supt6h_var1 | Vlasi-3 | 31 | 45 | 19 | 0 |
| supt6h_var1 | Vlasi-4 | 42 | 23 | 24 | 1 |
| usp_var1 | Vlasi-1 | 54 | 36 | 4 | 0 |
| usp_var1 | Vlasi-2 | 53 | 30 | 4 | 0 |
| usp_var1 | Vlasi-3 | 54 | 35 | 6 | 0 |
| usp_var1 | Vlasi-4 | 45 | 20 | 25 | 1 |
| wiz_var1 | Vlasi-1 | 61 | 26 | 5 | 0 |
| wiz_var1 | Vlasi-2 | 56 | 18 | 2 | 0 |
| wiz_var1 | Vlasi-3 | 64 | 27 | 4 | 0 |
| wiz_var1 | Vlasi-4 | 54 | 9 | 26 | 1 |
| ND4_var12 | Vlasi-1 | 73 | 0 | 0 | 0 |
| ND4_var12 | Vlasi-2 | 55 | 0 | 0 | 0 |
| ND4_var12 | Vlasi-3 | 88 | 0 | 0 | 0 |
| ND4_var12 | Vlasi-4 | 63 | 0 | 21 | 1 |

DNA

| MasterPlate | Density | Barcode |
|---|---|---|
| Vlasi-1 | 96 | |
| Vlasi-2 | 96 | |
| Vlasi-3 | 96 | |
| Vlasi-4 | 96 | |

# Please note: AL1=AlleleY axis and AL2=AlleleX axis, Allele Y is defined before allele X in the following h

SNPs

| SNPID | SNPNum | AlleleY | AlleleX | Sequence |
|---|---|---|---|---|
| arh_var1 | 70224 | C | G | GCCTGAGTCTTAGAG[G |
| clasp2_var1 | 70225 | - | C | TAAGCCTCCTGCCCC[C/ |

| | | | | |
|---|---|---|---|---|
| col18_var1 | 70226 | C | G | TGCTTCGGGGCTATC[G |
| ddx17_var1 | 70229 | C | T | CTGTAGAACTTTAGT[T/ |
| dnaj_var1 | 70230 | G | A | TTGGAGCAGTATCCY[A, |
| fam178_var1 | 70233 | A | C | GATTTGAAATTTACA[C/ |
| gak_var1 | 70234 | C | G | CCGTTCCATTCACCA[G/ |
| ganab_var1 | 70235 | C | A | CTGAAATCATTGTTG[A, |
| hmp19_var1 | 70236 | G | A | AAACAACATTTATTC[A/ |
| msantd4_var1 | 70238 | T | G | CTACCASTAGGAATT[G, |
| ND4_var12 | 70278 | T | C | YGYAAATTTATCAAA[C/ |
| opa_var1 | 70239 | T | C | TGATGTCAAAGAGGG[( |
| plekhg1_var1 | 70240 | T | C | AGACGACAATGACGA[( |
| slc25_var1 | 70241 | A | G | CATWCAGTNGTCACT[( |
| sre_var1 | 70242 | T | A | CATTGGCTTGCACCA[A, |
| supt6h_var1 | 70244 | C | A | CCTACAGGAGTCTTT[A, |
| usp_var1 | 70247 | G | A | CCCGCTCTAGTGTGG[A |
| wiz_var1 | 70248 | C | T | AGTTYGTGATTTCTA[T/ |

Scaling

| DaughterPlate | | SNPNum | Xmin | Xmax | Ymin | Ymax |
|---|---|---|---|---|---|---|
| | 2031013 | 70224 | 0.40097 | 3.7982 | 0.47152 | 3.9288 |
| | 2031013 | 70225 | 0.38356 | 3.86969 | 0.50702 | 4.00439 |
| | 2031013 | 70226 | 0.36191 | 4.01757 | 0.39902 | 3.93352 |
| | 2031011 | 70229 | 0.35694 | 3.82633 | 0.44194 | 3.80534 |
| | 2031011 | 70230 | 0.3178 | 3.82157 | 0.41459 | 3.72135 |
| | 2031011 | 70233 | 0.39336 | 3.99147 | 0.51867 | 3.87462 |
| | 2031011 | 70234 | 0.35752 | 3.94035 | 0.34176 | 3.5195 |
| | 2031012 | 70235 | 0.34708 | 3.99873 | 0.36498 | 3.97764 |
| | 2031012 | 70236 | 0.33211 | 4.08509 | 0.41089 | 3.95211 |
| | 2031012 | 70238 | 0.3198 | 4.18354 | 0.42288 | 3.92296 |
| | 2031012 | 70239 | 0.33984 | 4.29064 | 0.39498 | 4.01285 |
| | 2031014 | 70240 | 0.3749 | 3.63899 | 0.33724 | 3.76062 |
| | 2031014 | 70241 | 0.41175 | 3.95232 | 0.37723 | 4.02174 |
| | 2031014 | 70242 | 0.45039 | 4.112 | 0.42414 | 4.09822 |
| | 2031014 | 70244 | 0.37454 | 4.10881 | 0.38444 | 3.8175 |
| | 2031015 | 70246 | 0.33446 | 3.64142 | 0.38745 | 3.84632 |
| | 2031015 | 70247 | 0.3547 | 3.76864 | 0.33784 | 3.95089 |
| | 2031015 | 70248 | 0.35636 | 3.51717 | 0.46573 | 3.89136 |
| | 2031015 | 70278 | 0.40233 | 3.75023 | 0.3254 | 3.75776 |

Data

| DaughterPlate | | MasterPlat | MasterWel | Call | X | Y |
|---|---|---|---|---|---|---|
| | 2031013 | Vlasi-1 | A01 | C:G | 2.12261 | 2.20744 |
| | 2031013 | Vlasi-1 | B01 | C:G | 2.18185 | 2.33917 |
| | 2031013 | Vlasi-1 | C01 | C:G | 2.1521 | 2.24024 |
| | 2031013 | Vlasi-1 | D01 | G:G | 3.46553 | 0.50259 |
| | 2031013 | Vlasi-1 | E01 | C:G | 2.22748 | 2.31629 |
| | 2031013 | Vlasi-1 | F01 | C:G | 2.21173 | 2.48736 |
| | 2031013 | Vlasi-1 | G01 | C:G | 2.14131 | 2.3243 |
| | 2031013 | Vlasi-1 | H01 | C:C | 0.43379 | 3.30042 |
| | 2031013 | Vlasi-1 | A02 | C:C | 0.52096 | 3.30382 |

| | | | | | |
|---|---|---|---|---|---|
| 2031013 | Vlasi-1 | B02 | C:C | 0.50358 | 3.54044 |
| 2031013 | Vlasi-1 | C02 | C:C | 0.45437 | 3.57739 |
| 2031013 | Vlasi-1 | D02 | C:G | 2.14424 | 2.30066 |
| 2031013 | Vlasi-1 | E02 | C:G | 1.96187 | 2.29421 |
| 2031013 | Vlasi-1 | F02 | C:C | 0.45 | 3.66542 |
| 2031013 | Vlasi-1 | G02 | C:C | 0.46374 | 3.49485 |
| 2031013 | Vlasi-1 | H02 | C:G | 2.03149 | 2.28586 |
| 2031013 | Vlasi-1 | A03 | C:G | 1.97716 | 2.30589 |
| 2031013 | Vlasi-1 | B03 | G:G | 3.35083 | 0.5717 |
| 2031013 | Vlasi-1 | C03 | C:G | 2.08241 | 2.31176 |
| 2031013 | Vlasi-1 | D03 | G:G | 3.46817 | 0.65214 |
| 2031013 | Vlasi-1 | E03 | C:G | 1.99703 | 2.35382 |
| 2031013 | Vlasi-1 | F03 | C:G | 1.92855 | 2.43479 |
| 2031013 | Vlasi-1 | G03 | C:G | 2.0642 | 2.4387 |
| 2031013 | Vlasi-1 | H03 | C:G | 1.99117 | 2.30441 |
| 2031013 | Vlasi-1 | A04 | C:G | 2.139 | 2.25837 |
| 2031013 | Vlasi-1 | B04 | C:C | 0.45956 | 3.53252 |
| 2031013 | Vlasi-1 | C04 | C:C | 0.44021 | 3.50922 |
| 2031013 | Vlasi-1 | D04 | G:G | 3.47861 | 0.58305 |
| 2031013 | Vlasi-1 | E04 | C:G | 2.06066 | 2.36259 |
| 2031013 | Vlasi-1 | F04 | C:G | 2.01163 | 2.27312 |
| 2031013 | Vlasi-1 | G04 | C:C | 0.43758 | 3.67779 |
| 2031013 | Vlasi-1 | H04 | C:C | 0.43367 | 3.53822 |
| 2031013 | Vlasi-1 | A05 | C:C | 0.52065 | 3.21921 |
| 2031013 | Vlasi-1 | B05 | C:G | 2.08126 | 2.42139 |
| 2031013 | Vlasi-1 | C05 | C:G | 2.06706 | 2.23456 |
| 2031013 | Vlasi-1 | D05 | C:G | 1.96271 | 2.5339 |
| 2031013 | Vlasi-1 | E05 | C:G | 1.99722 | 2.29698 |
| 2031013 | Vlasi-1 | F05 | C:C | 0.44038 | 3.74988 |
| 2031013 | Vlasi-1 | G05 | C:C | 0.42316 | 3.63715 |
| 2031013 | Vlasi-1 | H05 | C:C | 0.43376 | 3.61929 |
| 2031013 | Vlasi-1 | A06 | C:G | 1.93095 | 2.30557 |
| 2031013 | Vlasi-1 | B06 | C:C | 0.44629 | 3.50283 |
| 2031013 | Vlasi-1 | C06 | C:C | 0.44371 | 3.73912 |
| 2031013 | Vlasi-1 | D06 | C:G | 2.07475 | 2.4134 |
| 2031013 | Vlasi-1 | E06 | C:C | 0.42264 | 3.35498 |
| 2031013 | Vlasi-1 | F06 | C:G | 1.86178 | 2.48668 |
| 2031013 | Vlasi-1 | G06 | C:C | 0.4649 | 3.87793 |
| 2031013 | Vlasi-1 | H06 | C:C | 0.50977 | 3.64053 |
| 2031013 | Vlasi-1 | A07 | G:G | 3.30048 | 0.60638 |
| 2031013 | Vlasi-1 | B07 | C:C | 0.49031 | 3.49744 |
| 2031013 | Vlasi-1 | C07 | C:G | 1.95887 | 2.30169 |
| 2031013 | Vlasi-1 | D07 | C:G | 1.98967 | 2.26929 |
| 2031013 | Vlasi-1 | E07 | C:C | 0.43088 | 3.55811 |
| 2031013 | Vlasi-1 | F07 | C:G | 2.07767 | 2.32681 |
| 2031013 | Vlasi-1 | G07 | C:G | 2.02003 | 2.12395 |
| 2031013 | Vlasi-1 | H07 | C:C | 0.47653 | 3.49074 |
| 2031013 | Vlasi-1 | A08 | C:G | 1.92533 | 2.38249 |
| 2031013 | Vlasi-1 | B08 | C:C | 0.472 | 3.70135 |
| 2031013 | Vlasi-1 | C08 | C:C | 0.44292 | 3.61168 |

| | | | | | |
|---|---|---|---|---|---|
| 2031013 | Vlasi-1 | D08 | C:G | 2.06145 | 2.29347 |
| 2031013 | Vlasi-1 | E08 | C:G | 2.1024 | 2.30906 |
| 2031013 | Vlasi-1 | F08 | C:G | 2.06442 | 2.35267 |
| 2031013 | Vlasi-1 | G08 | C:C | 0.43774 | 3.72681 |
| 2031013 | Vlasi-1 | H08 | C:C | 0.44976 | 3.79149 |
| 2031013 | Vlasi-1 | A09 | C:G | 1.82494 | 2.49702 |
| 2031013 | Vlasi-1 | B09 | C:C | 0.46404 | 3.53066 |
| 2031013 | Vlasi-1 | C09 | C:C | 0.44392 | 3.67195 |
| 2031013 | Vlasi-1 | D09 | C:G | 1.99753 | 2.33377 |
| 2031013 | Vlasi-1 | E09 | C:G | 1.97515 | 2.47771 |
| 2031013 | Vlasi-1 | F09 | C:G | 1.96176 | 2.30036 |
| 2031013 | Vlasi-1 | G09 | C:C | 0.42962 | 3.50702 |
| 2031013 | Vlasi-1 | H09 | C:G | 2.08605 | 2.41844 |
| 2031013 | Vlasi-1 | A10 | G:G | 3.28519 | 0.59394 |
| 2031013 | Vlasi-1 | B10 | C:G | 2.02198 | 2.22008 |
| 2031013 | Vlasi-1 | C10 | C:C | 0.44154 | 3.65112 |
| 2031013 | Vlasi-1 | D10 | G:G | 3.3191 | 0.52971 |
| 2031013 | Vlasi-1 | E10 | G:G | 3.48546 | 0.56295 |
| 2031013 | Vlasi-1 | F10 | G:G | 3.49559 | 0.53063 |
| 2031013 | Vlasi-1 | G10 | C:G | 2.07361 | 2.45026 |
| 2031013 | Vlasi-1 | H10 | C:C | 0.43172 | 3.49092 |
| 2031013 | Vlasi-1 | A11 | C:C | 0.49747 | 3.46503 |
| 2031013 | Vlasi-1 | B11 | C:G | 2.02764 | 2.52207 |
| 2031013 | Vlasi-1 | C11 | C:C | 0.45142 | 3.69099 |
| 2031013 | Vlasi-1 | D11 | C:G | 2.02623 | 2.3708 |
| 2031013 | Vlasi-1 | E11 | G:G | 3.44132 | 0.60134 |
| 2031013 | Vlasi-1 | F11 | G:G | 3.50288 | 0.55913 |
| 2031013 | Vlasi-1 | G11 | C:C | 0.44892 | 3.67105 |
| 2031013 | Vlasi-1 | H11 | C:G | 2.0369 | 2.3139 |
| 2031013 | Vlasi-1 | A12 | C:C | 0.49078 | 3.28176 |
| 2031013 | Vlasi-1 | B12 | C:G | 2.06015 | 2.40324 |
| 2031013 | Vlasi-1 | C12 | C:G | 1.8444 | 2.01229 |
| 2031013 | Vlasi-1 | D12 | C:G | 1.92418 | 2.21956 |
| 2031013 | Vlasi-1 | E12 | C:G | 1.95988 | 2.24025 |
| 2031013 | Vlasi-1 | F12 | C:C | 0.45795 | 3.57408 |
| 2031013 | Vlasi-1 | G12 | C:G | 2.06329 | 2.46048 |
| 2031013 | Vlasi-1 | H12 | C:C | 0.46151 | 3.55578 |
| 2031013 | Vlasi-2 | A01 | C:G | 2.0968 | 2.30065 |
| 2031013 | Vlasi-2 | B01 | C:G | 2.03293 | 2.30439 |
| 2031013 | Vlasi-2 | C01 | C:C | 0.46413 | 3.5939 |
| 2031013 | Vlasi-2 | D01 | C:C | 0.43281 | 3.62261 |
| 2031013 | Vlasi-2 | E01 | C:C | 0.44328 | 3.73039 |
| 2031013 | Vlasi-2 | F01 | C:G | 2.13237 | 2.53305 |
| 2031013 | Vlasi-2 | G01 | C:G | 2.18851 | 2.42936 |
| 2031013 | Vlasi-2 | H01 | C:C | 0.45845 | 3.70119 |
| 2031013 | Vlasi-2 | A02 | C:G | 2.10535 | 2.40418 |
| 2031013 | Vlasi-2 | B02 | C:G | 2.10712 | 2.38738 |
| 2031013 | Vlasi-2 | C02 | C:G | 2.16414 | 2.4828 |
| 2031013 | Vlasi-2 | D02 | C:C | 0.44283 | 3.46947 |
| 2031013 | Vlasi-2 | E02 | C:C | 0.4173 | 3.49349 |

| | | | | | |
|---|---|---|---|---|---|
| 2031013 | Vlasi-2 | F02 | C:C | 0.435 | 3.72038 |
| 2031013 | Vlasi-2 | G02 | C:C | 0.4132 | 3.44819 |
| 2031013 | Vlasi-2 | H02 | C:G | 2.15434 | 2.24634 |
| 2031013 | Vlasi-2 | A03 | C:C | 0.57876 | 3.50079 |
| 2031013 | Vlasi-2 | B03 | C:G | 2.02639 | 2.36391 |
| 2031013 | Vlasi-2 | C03 | C:G | 2.09466 | 2.34195 |
| 2031013 | Vlasi-2 | D03 | C:G | 2.04145 | 2.36819 |
| 2031013 | Vlasi-2 | E03 | C:C | 0.4211 | 3.61154 |
| 2031013 | Vlasi-2 | F03 | C:G | 1.99633 | 2.42644 |
| 2031013 | Vlasi-2 | G03 | C:C | 0.57176 | 3.64819 |
| 2031013 | Vlasi-2 | H03 | C:G | 2.04976 | 2.38777 |
| 2031013 | Vlasi-2 | A04 | C:C | 0.56104 | 3.40757 |
| 2031013 | Vlasi-2 | B04 | G:G | 3.35695 | 0.5429 |
| 2031013 | Vlasi-2 | C04 | C:C | 0.43355 | 3.55862 |
| 2031013 | Vlasi-2 | D04 | C:G | 2.13866 | 2.2122 |
| 2031013 | Vlasi-2 | E04 | C:G | 2.16336 | 2.42137 |
| 2031013 | Vlasi-2 | F04 | C:G | 1.86499 | 2.05586 |
| 2031013 | Vlasi-2 | G04 | C:C | 0.4531 | 3.79965 |
| 2031013 | Vlasi-2 | H04 | C:G | 2.09851 | 2.3203 |
| 2031013 | Vlasi-2 | A05 | G:G | 3.39779 | 0.58427 |
| 2031013 | Vlasi-2 | B05 | G:G | 3.37856 | 0.51873 |
| 2031013 | Vlasi-2 | C05 | C:C | 0.43453 | 3.60698 |
| 2031013 | Vlasi-2 | D05 | C:C | 0.47003 | 3.91049 |
| 2031013 | Vlasi-2 | E05 | C:G | 2.04514 | 2.38179 |
| 2031013 | Vlasi-2 | F05 | C:G | 1.97514 | 2.33477 |
| 2031013 | Vlasi-2 | G05 | C:C | 0.45551 | 3.69779 |
| 2031013 | Vlasi-2 | H05 | C:C | 0.46698 | 3.83051 |
| 2031013 | Vlasi-2 | A06 | G:G | 3.36932 | 0.53069 |
| 2031013 | Vlasi-2 | B06 | C:G | 2.06864 | 2.29031 |
| 2031013 | Vlasi-2 | C06 | C:C | 0.46867 | 3.68483 |
| 2031013 | Vlasi-2 | D06 | C:G | 1.92006 | 2.38861 |
| 2031013 | Vlasi-2 | E06 | C:G | 2.15939 | 2.42356 |
| 2031013 | Vlasi-2 | F06 | C:G | 2.03266 | 2.3411 |
| 2031013 | Vlasi-2 | G06 | C:C | 0.42673 | 3.66481 |
| 2031013 | Vlasi-2 | H06 | C:G | 2.05743 | 2.35482 |
| 2031013 | Vlasi-2 | A07 | ? | 0.52354 | 2.71162 |
| 2031013 | Vlasi-2 | B07 | C:G | 2.06907 | 2.48442 |
| 2031013 | Vlasi-2 | C07 | C:G | 2.07046 | 2.3788 |
| 2031013 | Vlasi-2 | D07 | C:G | 2.04793 | 2.49017 |
| 2031013 | Vlasi-2 | E07 | C:C | 0.4565 | 3.51717 |
| 2031013 | Vlasi-2 | F07 | C:C | 0.42484 | 3.62074 |
| 2031013 | Vlasi-2 | G07 | C:G | 2.00242 | 2.29235 |
| 2031013 | Vlasi-2 | H07 | G:G | 3.64774 | 0.50842 |
| 2031013 | Vlasi-2 | A08 | G:G | 3.27745 | 0.59511 |
| 2031013 | Vlasi-2 | B08 | C:G | 2.04205 | 2.33117 |
| 2031013 | Vlasi-2 | C08 | C:G | 2.05855 | 2.29234 |
| 2031013 | Vlasi-2 | D08 | C:G | 2.06196 | 2.42595 |
| 2031013 | Vlasi-2 | E08 | C:G | 2.00714 | 2.30957 |
| 2031013 | Vlasi-2 | F08 | C:G | 2.05897 | 2.28865 |
| 2031013 | Vlasi-2 | G08 | C:C | 0.45328 | 3.67125 |

| | | | | | |
|---|---|---|---|---|---|
| 2031013 | Vlasi-2 | H08 | C:C | 0.47495 | 3.65081 |
| 2031013 | Vlasi-2 | A09 | C:C | 0.52702 | 3.36418 |
| 2031013 | Vlasi-2 | B09 | C:G | 2.02185 | 2.26893 |
| 2031013 | Vlasi-2 | C09 | C:C | 0.50459 | 3.85451 |
| 2031013 | Vlasi-2 | D09 | C:G | 2.09131 | 2.2749 |
| 2031013 | Vlasi-2 | E09 | C:G | 1.987 | 2.26909 |
| 2031013 | Vlasi-2 | F09 | C:G | 1.73097 | 2.20982 |
| 2031013 | Vlasi-2 | G09 | C:C | 0.43983 | 3.64725 |
| 2031013 | Vlasi-2 | H09 | C:C | 0.46544 | 3.72492 |
| 2031013 | Vlasi-2 | A10 | C:C | 0.54 | 3.39562 |
| 2031013 | Vlasi-2 | B10 | C:C | 0.46366 | 3.55087 |
| 2031013 | Vlasi-2 | C10 | C:C | 0.54921 | 3.91107 |
| 2031013 | Vlasi-2 | D10 | C:C | 0.45039 | 3.63743 |
| 2031013 | Vlasi-2 | E10 | G:G | 3.4324 | 0.54677 |
| 2031013 | Vlasi-2 | F10 | C:C | 0.4639 | 3.65574 |
| 2031013 | Vlasi-2 | G10 | G:G | 3.74039 | 0.60463 |
| 2031013 | Vlasi-2 | H10 | C:G | 2.04443 | 2.36658 |
| 2031013 | Vlasi-2 | A11 | C:G | 1.73781 | 2.03105 |
| 2031013 | Vlasi-2 | B11 | C:G | 2.01042 | 2.32317 |
| 2031013 | Vlasi-2 | C11 | C:C | 0.50902 | 3.76905 |
| 2031013 | Vlasi-2 | D11 | C:G | 2.04966 | 2.37897 |
| 2031013 | Vlasi-2 | E11 | C:C | 0.42507 | 3.6635 |
| 2031013 | Vlasi-2 | F11 | G:G | 3.54663 | 0.56127 |
| 2031013 | Vlasi-2 | G11 | C:C | 0.46582 | 3.59943 |
| 2031013 | Vlasi-2 | H11 | C:C | 0.45452 | 3.73031 |
| 2031013 | Vlasi-2 | A12 | C:G | 2.07592 | 2.1764 |
| 2031013 | Vlasi-2 | B12 | C:G | 2.12739 | 2.25447 |
| 2031013 | Vlasi-2 | C12 | C:C | 0.50317 | 3.58847 |
| 2031013 | Vlasi-2 | D12 | C:C | 0.49053 | 3.71835 |
| 2031013 | Vlasi-2 | E12 | C:G | 2.03216 | 2.52856 |
| 2031013 | Vlasi-2 | F12 | C:G | 2.13974 | 2.33407 |
| 2031013 | Vlasi-2 | G12 | C:C | 0.56835 | 3.528 |
| 2031013 | Vlasi-2 | H12 | C:G | 2.1051 | 2.42221 |
| 2031013 | Vlasi-3 | A01 | C:C | 0.5183 | 3.43675 |
| 2031013 | Vlasi-3 | B01 | C:C | 0.46167 | 3.4894 |
| 2031013 | Vlasi-3 | C01 | C:G | 2.18189 | 2.4673 |
| 2031013 | Vlasi-3 | D01 | C:C | 0.40643 | 3.64149 |
| 2031013 | Vlasi-3 | E01 | C:C | 0.41049 | 3.53015 |
| 2031013 | Vlasi-3 | F01 | C:C | 0.42831 | 3.59527 |
| 2031013 | Vlasi-3 | G01 | C:G | 2.14864 | 2.32817 |
| 2031013 | Vlasi-3 | H01 | G:G | 3.77259 | 0.55661 |
| 2031013 | Vlasi-3 | A02 | C:C | 0.49287 | 3.59292 |
| 2031013 | Vlasi-3 | B02 | C:C | 0.46583 | 3.69539 |
| 2031013 | Vlasi-3 | C02 | C:C | 0.44885 | 3.80052 |
| 2031013 | Vlasi-3 | D02 | C:C | 0.45461 | 3.9288 |
| 2031013 | Vlasi-3 | E02 | C:G | 2.0385 | 2.45449 |
| 2031013 | Vlasi-3 | F02 | C:C | 0.44757 | 3.60284 |
| 2031013 | Vlasi-3 | G02 | C:C | 0.43326 | 3.4902 |
| 2031013 | Vlasi-3 | H02 | C:G | 2.21031 | 2.39083 |
| 2031013 | Vlasi-3 | A03 | G:G | 3.22825 | 0.5999 |

| | | | | | |
|---|---|---|---|---|---|
| 2031013 | Vlasi-3 | B03 | C:C | 0.45043 | 3.56075 |
| 2031013 | Vlasi-3 | C03 | C:G | 2.07614 | 2.35305 |
| 2031013 | Vlasi-3 | D03 | C:C | 0.43166 | 3.56337 |
| 2031013 | Vlasi-3 | E03 | C:G | 1.909 | 2.48636 |
| 2031013 | Vlasi-3 | F03 | C:G | 2.08655 | 2.35446 |
| 2031013 | Vlasi-3 | G03 | C:G | 2.16232 | 2.37513 |
| 2031013 | Vlasi-3 | H03 | C:G | 2.18145 | 2.49048 |
| 2031013 | Vlasi-3 | A04 | C:C | 0.46262 | 3.50367 |
| 2031013 | Vlasi-3 | B04 | C:G | 2.01909 | 2.23712 |
| 2031013 | Vlasi-3 | C04 | C:C | 0.4523 | 3.71274 |
| 2031013 | Vlasi-3 | D04 | C:C | 0.45937 | 3.64228 |
| 2031013 | Vlasi-3 | E04 | G:G | 3.44173 | 0.59579 |
| 2031013 | Vlasi-3 | F04 | C:C | 0.42114 | 3.66495 |
| 2031013 | Vlasi-3 | G04 | C:G | 2.09116 | 2.2304 |
| 2031013 | Vlasi-3 | H04 | C:G | 2.06437 | 2.32779 |
| 2031013 | Vlasi-3 | A05 | G:G | 3.32245 | 0.57773 |
| 2031013 | Vlasi-3 | B05 | G:G | 3.36588 | 0.55943 |
| 2031013 | Vlasi-3 | C05 | C:C | 0.44223 | 3.45813 |
| 2031013 | Vlasi-3 | D05 | C:C | 0.43032 | 3.57723 |
| 2031013 | Vlasi-3 | E05 | C:C | 0.45679 | 3.8018 |
| 2031013 | Vlasi-3 | F05 | C:G | 2.00542 | 2.27132 |
| 2031013 | Vlasi-3 | G05 | C:G | 2.0903 | 2.34089 |
| 2031013 | Vlasi-3 | H05 | C:G | 2.21833 | 2.53544 |
| 2031013 | Vlasi-3 | A06 | C:C | 0.42933 | 3.26066 |
| 2031013 | Vlasi-3 | B06 | G:G | 3.296 | 0.50555 |
| 2031013 | Vlasi-3 | C06 | C:G | 1.97828 | 2.20673 |
| 2031013 | Vlasi-3 | D06 | C:C | 0.40902 | 3.60415 |
| 2031013 | Vlasi-3 | E06 | C:G | 1.98149 | 2.27253 |
| 2031013 | Vlasi-3 | F06 | C:C | 0.43231 | 3.68404 |
| 2031013 | Vlasi-3 | G06 | C:C | 0.43661 | 3.52092 |
| 2031013 | Vlasi-3 | H06 | C:C | 0.48943 | 3.65267 |
| 2031013 | Vlasi-3 | A07 | C:G | 1.97466 | 2.32663 |
| 2031013 | Vlasi-3 | B07 | C:G | 1.90401 | 2.26085 |
| 2031013 | Vlasi-3 | C07 | C:C | 0.43365 | 3.53084 |
| 2031013 | Vlasi-3 | D07 | C:C | 0.43603 | 3.64532 |
| 2031013 | Vlasi-3 | E07 | G:G | 3.51723 | 0.5591 |
| 2031013 | Vlasi-3 | F07 | C:G | 2.00745 | 2.39252 |
| 2031013 | Vlasi-3 | G07 | C:G | 2.12566 | 2.46858 |
| 2031013 | Vlasi-3 | H07 | C:C | 0.48653 | 3.816 |
| 2031013 | Vlasi-3 | A08 | C:C | 0.44669 | 3.55803 |
| 2031013 | Vlasi-3 | B08 | G:G | 3.32623 | 0.55135 |
| 2031013 | Vlasi-3 | C08 | C:G | 1.91768 | 2.32756 |
| 2031013 | Vlasi-3 | D08 | C:G | 2.02927 | 2.40302 |
| 2031013 | Vlasi-3 | E08 | C:C | 0.44079 | 3.80446 |
| 2031013 | Vlasi-3 | F08 | C:G | 1.8448 | 2.09737 |
| 2031013 | Vlasi-3 | G08 | C:C | 0.48854 | 3.5811 |
| 2031013 | Vlasi-3 | H08 | C:G | 2.18048 | 2.39508 |
| 2031013 | Vlasi-3 | A09 | C:G | 2.04556 | 2.36489 |
| 2031013 | Vlasi-3 | B09 | C:C | 0.44214 | 3.51009 |
| 2031013 | Vlasi-3 | C09 | C:G | 1.96757 | 2.31147 |

| | | | | | |
|---|---|---|---|---|---|
| 2031013 | Vlasi-3 | D09 | C:C | 0.40159 | 3.39889 |
| 2031013 | Vlasi-3 | E09 | C:G | 2.0283 | 2.26817 |
| 2031013 | Vlasi-3 | F09 | C:G | 2.00233 | 2.35504 |
| 2031013 | Vlasi-3 | G09 | C:C | 0.4616 | 3.7459 |
| 2031013 | Vlasi-3 | H09 | C:G | 2.17649 | 2.47568 |
| 2031013 | Vlasi-3 | A10 | C:G | 2.03154 | 2.19275 |
| 2031013 | Vlasi-3 | B10 | G:G | 3.48802 | 0.52177 |
| 2031013 | Vlasi-3 | C10 | C:C | 0.46088 | 3.64801 |
| 2031013 | Vlasi-3 | D10 | C:C | 0.48721 | 3.63207 |
| 2031013 | Vlasi-3 | E10 | C:C | 0.43119 | 3.66082 |
| 2031013 | Vlasi-3 | F10 | G:G | 3.6515 | 0.51468 |
| 2031013 | Vlasi-3 | G10 | C:C | 0.44399 | 3.60671 |
| 2031013 | Vlasi-3 | H10 | C:G | 2.07609 | 2.43143 |
| 2031013 | Vlasi-3 | A11 | C:G | 1.96924 | 2.30861 |
| 2031013 | Vlasi-3 | B11 | C:G | 2.00406 | 2.32503 |
| 2031013 | Vlasi-3 | C11 | C:C | 0.40847 | 3.54723 |
| 2031013 | Vlasi-3 | D11 | C:G | 1.98351 | 2.28292 |
| 2031013 | Vlasi-3 | E11 | C:C | 0.47787 | 3.43106 |
| 2031013 | Vlasi-3 | F11 | C:C | 0.43699 | 3.64234 |
| 2031013 | Vlasi-3 | G11 | C:C | 0.443 | 3.49897 |
| 2031013 | Vlasi-3 | H11 | G:G | 3.34973 | 0.58039 |
| 2031013 | Vlasi-3 | A12 | G:G | 3.3826 | 0.55745 |
| 2031013 | Vlasi-3 | B12 | C:C | 0.45269 | 3.54901 |
| 2031013 | Vlasi-3 | C12 | C:C | 0.43423 | 3.5255 |
| 2031013 | Vlasi-3 | D12 | C:G | 2.01285 | 2.54428 |
| 2031013 | Vlasi-3 | E12 | G:G | 3.49185 | 0.56176 |
| 2031013 | Vlasi-3 | F12 | C:G | 2.07183 | 2.4636 |
| 2031013 | Vlasi-3 | G12 | C:C | 0.65822 | 3.33464 |
| 2031013 | Vlasi-3 | H12 | C:G | 2.23439 | 2.43816 |
| 2031013 | Vlasi-4 | A01 | G:G | 3.39493 | 0.57077 |
| 2031013 | Vlasi-4 | B01 | C:G | 2.09036 | 2.42248 |
| 2031013 | Vlasi-4 | C01 | C:G | 2.02755 | 2.13217 |
| 2031013 | Vlasi-4 | D01 | C:C | 0.40757 | 3.36384 |
| 2031013 | Vlasi-4 | E01 | C:G | 2.06841 | 2.34526 |
| 2031013 | Vlasi-4 | F01 | C:C | 0.43357 | 3.68838 |
| 2031013 | Vlasi-4 | G01 | C:G | 2.07846 | 2.41448 |
| 2031013 | Vlasi-4 | H01 | G:G | 3.68471 | 0.60061 |
| 2031013 | Vlasi-4 | A02 | C:G | 2.06222 | 2.35878 |
| 2031013 | Vlasi-4 | B02 | C:G | 2.17129 | 2.39303 |
| 2031013 | Vlasi-4 | C02 | C:G | 2.08848 | 2.45047 |
| 2031013 | Vlasi-4 | D02 | C:G | 2.08044 | 2.34617 |
| 2031013 | Vlasi-4 | E02 | G:G | 3.50029 | 0.52846 |
| 2031013 | Vlasi-4 | F02 | C:G | 2.04214 | 2.41149 |
| 2031013 | Vlasi-4 | G02 | C:C | 0.46129 | 3.67178 |
| 2031013 | Vlasi-4 | H02 | C:G | 2.08729 | 2.43981 |
| 2031013 | Vlasi-4 | A03 | C:G | 2.05517 | 2.15939 |
| 2031013 | Vlasi-4 | B03 | G:G | 3.2771 | 0.50396 |
| 2031013 | Vlasi-4 | C03 | C:C | 0.44731 | 3.63832 |
| 2031013 | Vlasi-4 | D03 | C:G | 2.04229 | 2.32907 |
| 2031013 | Vlasi-4 | E03 | C:C | 0.42339 | 3.54773 |

| | | | | | |
|---|---|---|---|---|---|
| 2031013 | Vlasi-4 | F03 | C:G | 1.99172 | 2.35738 |
| 2031013 | Vlasi-4 | G03 | C:C | 0.46817 | 3.72126 |
| 2031013 | Vlasi-4 | H03 | C:C | 0.48277 | 3.765 |
| 2031013 | Vlasi-4 | A04 | C:G | 1.89395 | 2.22875 |
| 2031013 | Vlasi-4 | B04 | C:G | 1.90936 | 2.46443 |
| 2031013 | Vlasi-4 | C04 | C:G | 2.08591 | 2.3288 |
| 2031013 | Vlasi-4 | D04 | C:C | 0.42797 | 3.61842 |
| 2031013 | Vlasi-4 | E04 | G:G | 3.4628 | 0.56762 |
| 2031013 | Vlasi-4 | F04 | C:C | 0.41313 | 3.57522 |
| 2031013 | Vlasi-4 | G04 | C:G | 2.16372 | 2.35988 |
| 2031013 | Vlasi-4 | H04 | C:C | 0.45102 | 3.61655 |
| 2031013 | Vlasi-4 | A05 | C:G | 1.96819 | 2.26693 |
| 2031013 | Vlasi-4 | B05 | C:G | 1.91066 | 2.29282 |
| 2031013 | Vlasi-4 | C05 | C:C | 0.42679 | 3.65778 |
| 2031013 | Vlasi-4 | D05 | C:G | 2.12472 | 2.33664 |
| 2031013 | Vlasi-4 | E05 | G:G | 3.39355 | 0.49993 |
| 2031013 | Vlasi-4 | F05 | C:G | 1.94797 | 2.32029 |
| 2031013 | Vlasi-4 | G05 | G:G | 3.19545 | 0.47152 |
| 2031013 | Vlasi-4 | H05 | C:G | 2.0903 | 2.25346 |
| 2031013 | Vlasi-4 | A06 | C:G | 1.85412 | 2.24658 |
| 2031013 | Vlasi-4 | B06 | C:G | 1.82474 | 2.27916 |
| 2031013 | Vlasi-4 | C06 | C:G | 1.93545 | 2.42673 |
| 2031013 | Vlasi-4 | D06 | C:G | 2.01757 | 2.15746 |
| 2031013 | Vlasi-4 | E06 | C:C | 0.40351 | 3.42628 |
| 2031013 | Vlasi-4 | F06 | C:C | 0.44612 | 3.73583 |
| 2031013 | Vlasi-4 | G06 | C:C | 0.45121 | 3.74967 |
| 2031013 | Vlasi-4 | H06 | G:G | 3.53186 | 0.53946 |
| 2031013 | Vlasi-4 | A07 | ? | 0.5812 | 0.52558 |
| 2031013 | Vlasi-4 | B07 | ? | 0.56515 | 0.50947 |
| 2031013 | Vlasi-4 | C07 | C:C | 0.44571 | 3.38253 |
| 2031013 | Vlasi-4 | D07 | ? | 0.6588 | 0.68664 |
| 2031013 | Vlasi-4 | E07 | ? | 0.67276 | 0.57763 |
| 2031013 | Vlasi-4 | F07 | G:G | 3.43676 | 0.54744 |
| 2031013 | Vlasi-4 | G07 | G:G | 3.69771 | 0.58126 |
| 2031013 | Vlasi-4 | H07 | G:G | 3.75274 | 0.63242 |
| 2031013 | Vlasi-4 | A08 | G:G | 3.46831 | 0.59155 |
| 2031013 | Vlasi-4 | B08 | G:G | 3.32027 | 0.59773 |
| 2031013 | Vlasi-4 | C08 | G:G | 3.50049 | 0.59261 |
| 2031013 | Vlasi-4 | D08 | G:G | 3.48119 | 0.59568 |
| 2031013 | Vlasi-4 | E08 | G:G | 3.48662 | 0.62672 |
| 2031013 | Vlasi-4 | F08 | G:G | 3.44819 | 0.58164 |
| 2031013 | Vlasi-4 | G08 | G:G | 3.59936 | 0.58374 |
| 2031013 | Vlasi-4 | H08 | G:G | 3.7982 | 0.6308 |
| 2031013 | Vlasi-4 | A09 | G:G | 3.54439 | 0.63097 |
| 2031013 | Vlasi-4 | B09 | G:G | 3.51306 | 0.62009 |
| 2031013 | Vlasi-4 | C09 | G:G | 3.4089 | 0.56604 |
| 2031013 | Vlasi-4 | D09 | G:G | 3.65466 | 0.62447 |
| 2031013 | Vlasi-4 | E09 | G:G | 3.51452 | 0.6001 |
| 2031013 | Vlasi-4 | F09 | G:G | 3.67548 | 0.59255 |
| 2031013 | Vlasi-4 | G09 | G:G | 3.54724 | 0.61606 |

| | | | | | |
|---|---|---|---|---|---|
| 2031013 | Vlasi-4 | H09 | G:G | 3.03166 | 0.49053 |
| 2031013 | Vlasi-4 | A10 | G:G | 3.48538 | 0.62935 |
| 2031013 | Vlasi-4 | B10 | G:G | 3.44882 | 0.57279 |
| 2031013 | Vlasi-4 | C10 | C:C | 0.41532 | 3.64604 |
| 2031013 | Vlasi-4 | D10 | C:C | 0.42164 | 3.60642 |
| 2031013 | Vlasi-4 | E10 | C:C | 0.44479 | 3.4723 |
| 2031013 | Vlasi-4 | F10 | C:C | 0.44748 | 3.55037 |
| 2031013 | Vlasi-4 | G10 | C:C | 0.45304 | 3.59027 |
| 2031013 | Vlasi-4 | H10 | C:C | 0.50087 | 3.52978 |
| 2031013 | Vlasi-4 | A11 | C:C | 0.4757 | 3.35638 |
| 2031013 | Vlasi-4 | B11 | C:C | 0.46891 | 3.62736 |
| 2031013 | Vlasi-4 | C11 | C:C | 0.45906 | 3.63248 |
| 2031013 | Vlasi-4 | D11 | C:C | 0.40097 | 3.17884 |
| 2031013 | Vlasi-4 | E11 | C:C | 0.4341 | 3.45069 |
| 2031013 | Vlasi-4 | F11 | C:C | 0.42433 | 3.43938 |
| 2031013 | Vlasi-4 | G11 | C:C | 0.45304 | 3.62483 |
| 2031013 | Vlasi-4 | H11 | C:C | 0.49405 | 3.63755 |
| 2031013 | Vlasi-4 | A12 | C:C | 0.56673 | 3.31086 |
| 2031013 | Vlasi-4 | B12 | C:C | 0.52386 | 3.40679 |
| 2031013 | Vlasi-4 | C12 | C:C | 0.49006 | 3.39103 |
| 2031013 | Vlasi-4 | D12 | C:C | 0.55267 | 3.45435 |
| 2031013 | Vlasi-4 | E12 | C:C | 0.57413 | 3.43564 |
| 2031013 | Vlasi-4 | F12 | C:C | 0.56708 | 3.62664 |
| 2031013 | Vlasi-4 | G12 | C:C | 0.54809 | 3.52607 |
| 2031013 | Vlasi-4 | H12 | NTC | 0.59933 | 0.56437 |
| 2031013 | Vlasi-1 | A01 | -:C | 2.42218 | 2.33697 |
| 2031013 | Vlasi-1 | B01 | -:C | 2.4843 | 2.27989 |
| 2031013 | Vlasi-1 | C01 | -:C | 2.40115 | 2.35223 |
| 2031013 | Vlasi-1 | D01 | -:- | 0.38356 | 3.60799 |
| 2031013 | Vlasi-1 | E01 | -:- | 0.38453 | 3.7456 |
| 2031013 | Vlasi-1 | F01 | -:- | 0.41532 | 3.77214 |
| 2031013 | Vlasi-1 | G01 | -:C | 2.38026 | 2.29721 |
| 2031013 | Vlasi-1 | H01 | -:C | 2.34044 | 2.32911 |
| 2031013 | Vlasi-1 | A02 | -:C | 2.28195 | 2.26672 |
| 2031013 | Vlasi-1 | B02 | -:- | 0.43264 | 3.51645 |
| 2031013 | Vlasi-1 | C02 | C:C | 3.75814 | 0.54124 |
| 2031013 | Vlasi-1 | D02 | -:C | 2.26751 | 2.25656 |
| 2031013 | Vlasi-1 | E02 | -:C | 2.41198 | 2.17002 |
| 2031013 | Vlasi-1 | F02 | C:C | 3.67144 | 0.56542 |
| 2031013 | Vlasi-1 | G02 | -:C | 2.17638 | 2.2246 |
| 2031013 | Vlasi-1 | H02 | -:- | 0.46536 | 3.53452 |
| 2031013 | Vlasi-1 | A03 | -:- | 0.44993 | 3.50368 |
| 2031013 | Vlasi-1 | B03 | C:C | 3.50078 | 0.54466 |
| 2031013 | Vlasi-1 | C03 | -:C | 2.2195 | 2.33334 |
| 2031013 | Vlasi-1 | D03 | -:- | 0.45099 | 3.69679 |
| 2031013 | Vlasi-1 | E03 | -:C | 2.26877 | 2.19093 |
| 2031013 | Vlasi-1 | F03 | C:C | 3.63257 | 0.59069 |
| 2031013 | Vlasi-1 | G03 | -:C | 2.31716 | 2.17145 |
| 2031013 | Vlasi-1 | H03 | -:- | 0.46278 | 3.76219 |
| 2031013 | Vlasi-1 | A04 | C:C | 3.4681 | 0.54522 |

| | | | | | |
|---|---|---|---|---|---|
| 2031013 | Vlasi-1 | B04 | -:- | 0.41066 | 3.52467 |
| 2031013 | Vlasi-1 | C04 | -:C | 2.19818 | 2.31752 |
| 2031013 | Vlasi-1 | D04 | -:- | 0.43259 | 3.81167 |
| 2031013 | Vlasi-1 | E04 | -:- | 0.43364 | 3.636 |
| 2031013 | Vlasi-1 | F04 | -:- | 0.45 | 3.75255 |
| 2031013 | Vlasi-1 | G04 | -:C | 2.2824 | 2.22358 |
| 2031013 | Vlasi-1 | H04 | -:C | 2.13188 | 2.39356 |
| 2031013 | Vlasi-1 | A05 | -:- | 0.48269 | 3.63304 |
| 2031013 | Vlasi-1 | B05 | -:- | 0.4354 | 3.65244 |
| 2031013 | Vlasi-1 | C05 | -:- | 0.41955 | 3.63533 |
| 2031013 | Vlasi-1 | D05 | -:- | 0.42317 | 3.69734 |
| 2031013 | Vlasi-1 | E05 | -:- | 0.42268 | 3.65055 |
| 2031013 | Vlasi-1 | F05 | -:C | 2.18219 | 2.1341 |
| 2031013 | Vlasi-1 | G05 | -:- | 0.43641 | 3.46071 |
| 2031013 | Vlasi-1 | H05 | C:C | 3.69368 | 0.5596 |
| 2031013 | Vlasi-1 | A06 | -:C | 2.23897 | 2.15468 |
| 2031013 | Vlasi-1 | B06 | C:C | 3.69028 | 0.55255 |
| 2031013 | Vlasi-1 | C06 | -:- | 0.44494 | 3.66152 |
| 2031013 | Vlasi-1 | D06 | C:C | 3.71385 | 0.57516 |
| 2031013 | Vlasi-1 | E06 | -:C | 2.15992 | 2.26437 |
| 2031013 | Vlasi-1 | F06 | -:C | 2.3361 | 2.07637 |
| 2031013 | Vlasi-1 | G06 | C:C | 3.50533 | 0.56449 |
| 2031013 | Vlasi-1 | H06 | -:- | 0.44947 | 3.76113 |
| 2031013 | Vlasi-1 | A07 | -:- | 0.48361 | 3.56296 |
| 2031013 | Vlasi-1 | B07 | -:- | 0.45435 | 3.78087 |
| 2031013 | Vlasi-1 | C07 | C:C | 3.56365 | 0.58835 |
| 2031013 | Vlasi-1 | D07 | -:C | 2.12468 | 2.3208 |
| 2031013 | Vlasi-1 | E07 | -:C | 2.16088 | 2.19274 |
| 2031013 | Vlasi-1 | F07 | C:C | 3.58047 | 0.60571 |
| 2031013 | Vlasi-1 | G07 | -:- | 0.45897 | 3.71341 |
| 2031013 | Vlasi-1 | H07 | -:C | 2.27882 | 2.31069 |
| 2031013 | Vlasi-1 | A08 | -:C | 2.11355 | 2.10605 |
| 2031013 | Vlasi-1 | B08 | -:C | 2.33671 | 2.18372 |
| 2031013 | Vlasi-1 | C08 | -:- | 0.46343 | 3.61815 |
| 2031013 | Vlasi-1 | D08 | -:C | 2.21851 | 2.04389 |
| 2031013 | Vlasi-1 | E08 | -:C | 2.18206 | 2.15533 |
| 2031013 | Vlasi-1 | F08 | C:C | 3.7213 | 0.5795 |
| 2031013 | Vlasi-1 | G08 | -:C | 2.20365 | 2.23635 |
| 2031013 | Vlasi-1 | H08 | C:C | 3.76752 | 0.64447 |
| 2031013 | Vlasi-1 | A09 | -:C | 2.25058 | 2.19727 |
| 2031013 | Vlasi-1 | B09 | C:C | 3.6743 | 0.57698 |
| 2031013 | Vlasi-1 | C09 | -:- | 0.41031 | 3.50857 |
| 2031013 | Vlasi-1 | D09 | -:- | 0.42577 | 3.63836 |
| 2031013 | Vlasi-1 | E09 | -:- | 0.42793 | 3.66074 |
| 2031013 | Vlasi-1 | F09 | -:- | 0.44334 | 3.68341 |
| 2031013 | Vlasi-1 | G09 | -:- | 0.45322 | 3.59431 |
| 2031013 | Vlasi-1 | H09 | -:C | 2.38143 | 2.12688 |
| 2031013 | Vlasi-1 | A10 | -:C | 2.13224 | 2.16257 |
| 2031013 | Vlasi-1 | B10 | -:C | 2.18125 | 2.10865 |
| 2031013 | Vlasi-1 | C10 | -:C | 2.20482 | 2.28581 |

| | | | | | |
|---|---|---|---|---|---|
| 2031013 | Vlasi-1 | D10 | -:- | 0.41599 | 3.65643 |
| 2031013 | Vlasi-1 | E10 | C:C | 3.67789 | 0.55205 |
| 2031013 | Vlasi-1 | F10 | C:C | 3.64141 | 0.55307 |
| 2031013 | Vlasi-1 | G10 | -:C | 2.09964 | 2.19492 |
| 2031013 | Vlasi-1 | H10 | -:C | 2.22845 | 2.32341 |
| 2031013 | Vlasi-1 | A11 | -:C | 2.09495 | 2.06179 |
| 2031013 | Vlasi-1 | B11 | -:- | 0.43856 | 3.67262 |
| 2031013 | Vlasi-1 | C11 | -:C | 2.20771 | 2.35364 |
| 2031013 | Vlasi-1 | D11 | -:- | 0.42025 | 3.54985 |
| 2031013 | Vlasi-1 | E11 | C:C | 3.59955 | 0.5429 |
| 2031013 | Vlasi-1 | F11 | C:C | 3.73007 | 0.5398 |
| 2031013 | Vlasi-1 | G11 | -:- | 0.4275 | 3.5545 |
| 2031013 | Vlasi-1 | H11 | -:- | 0.44787 | 3.64061 |
| 2031013 | Vlasi-1 | A12 | -:- | 0.45008 | 3.44544 |
| 2031013 | Vlasi-1 | B12 | -:- | 0.41908 | 3.4622 |
| 2031013 | Vlasi-1 | C12 | -:C | 2.17005 | 2.26724 |
| 2031013 | Vlasi-1 | D12 | -:C | 2.21846 | 2.2648 |
| 2031013 | Vlasi-1 | E12 | C:C | 3.69518 | 0.5325 |
| 2031013 | Vlasi-1 | F12 | -:- | 0.44676 | 3.47523 |
| 2031013 | Vlasi-1 | G12 | -:- | 0.44392 | 3.70667 |
| 2031013 | Vlasi-1 | H12 | -:- | 0.4519 | 3.62863 |
| 2031013 | Vlasi-2 | A01 | -:C | 2.34306 | 2.302 |
| 2031013 | Vlasi-2 | B01 | -:C | 2.43544 | 2.34759 |
| 2031013 | Vlasi-2 | C01 | C:C | 3.8253 | 0.59264 |
| 2031013 | Vlasi-2 | D01 | -:C | 2.39749 | 2.04936 |
| 2031013 | Vlasi-2 | E01 | C:C | 3.69584 | 0.53466 |
| 2031013 | Vlasi-2 | F01 | -:C | 2.31939 | 2.19966 |
| 2031013 | Vlasi-2 | G01 | -:- | 0.46454 | 3.76514 |
| 2031013 | Vlasi-2 | H01 | -:- | 0.47062 | 3.69184 |
| 2031013 | Vlasi-2 | A02 | -:C | 2.25772 | 2.19908 |
| 2031013 | Vlasi-2 | B02 | -:- | 0.44228 | 3.71001 |
| 2031013 | Vlasi-2 | C02 | -:C | 2.18753 | 2.15323 |
| 2031013 | Vlasi-2 | D02 | -:C | 2.17569 | 2.32545 |
| 2031013 | Vlasi-2 | E02 | -:C | 2.23489 | 2.12761 |
| 2031013 | Vlasi-2 | F02 | C:C | 3.50193 | 0.54274 |
| 2031013 | Vlasi-2 | G02 | -:- | 0.43179 | 3.55321 |
| 2031013 | Vlasi-2 | H02 | -:- | 0.46865 | 3.80649 |
| 2031013 | Vlasi-2 | A03 | -:C | 2.28745 | 2.19074 |
| 2031013 | Vlasi-2 | B03 | -:C | 2.19816 | 2.29333 |
| 2031013 | Vlasi-2 | C03 | -:- | 0.43024 | 3.60715 |
| 2031013 | Vlasi-2 | D03 | -:C | 2.25631 | 2.25125 |
| 2031013 | Vlasi-2 | E03 | -:C | 2.18661 | 2.15444 |
| 2031013 | Vlasi-2 | F03 | -:- | 0.42782 | 3.57958 |
| 2031013 | Vlasi-2 | G03 | -:C | 2.25525 | 2.2562 |
| 2031013 | Vlasi-2 | H03 | C:C | 3.81392 | 0.58554 |
| 2031013 | Vlasi-2 | A04 | -:C | 2.20608 | 2.1812 |
| 2031013 | Vlasi-2 | B04 | -:C | 2.29782 | 2.13163 |
| 2031013 | Vlasi-2 | C04 | -:- | 0.44067 | 3.73946 |
| 2031013 | Vlasi-2 | D04 | -:C | 2.20169 | 2.28567 |
| 2031013 | Vlasi-2 | E04 | -:- | 0.4615 | 3.65177 |

| | | | | | |
|---|---|---|---|---|---|
| 2031013 | Vlasi-2 | F04 | -:- | 0.44367 | 3.7284 |
| 2031013 | Vlasi-2 | G04 | -:- | 0.46766 | 3.86508 |
| 2031013 | Vlasi-2 | H04 | C:C | 3.67038 | 0.60604 |
| 2031013 | Vlasi-2 | A05 | C:C | 3.41896 | 0.58625 |
| 2031013 | Vlasi-2 | B05 | -:C | 2.25903 | 2.20331 |
| 2031013 | Vlasi-2 | C05 | -:- | 0.43714 | 3.69193 |
| 2031013 | Vlasi-2 | D05 | -:C | 2.18483 | 2.26123 |
| 2031013 | Vlasi-2 | E05 | -:- | 0.43869 | 3.7549 |
| 2031013 | Vlasi-2 | F05 | -:- | 0.415 | 3.51407 |
| 2031013 | Vlasi-2 | G05 | -:C | 2.20612 | 2.37604 |
| 2031013 | Vlasi-2 | H05 | -:C | 2.25711 | 2.38869 |
| 2031013 | Vlasi-2 | A06 | -:- | 0.51675 | 3.53786 |
| 2031013 | Vlasi-2 | B06 | -:C | 2.23647 | 2.26038 |
| 2031013 | Vlasi-2 | C06 | -:C | 2.29402 | 2.35196 |
| 2031013 | Vlasi-2 | D06 | -:C | 2.30832 | 2.01961 |
| 2031013 | Vlasi-2 | E06 | C:C | 3.78753 | 0.55146 |
| 2031013 | Vlasi-2 | F06 | -:C | 2.31375 | 2.28445 |
| 2031013 | Vlasi-2 | G06 | -:- | 0.46009 | 3.82456 |
| 2031013 | Vlasi-2 | H06 | -:- | 0.45162 | 3.59736 |
| 2031013 | Vlasi-2 | A07 | C:C | 3.44309 | 0.60174 |
| 2031013 | Vlasi-2 | B07 | C:C | 3.76185 | 0.50702 |
| 2031013 | Vlasi-2 | C07 | C:C | 3.56345 | 0.5572 |
| 2031013 | Vlasi-2 | D07 | -:C | 2.12731 | 2.17422 |
| 2031013 | Vlasi-2 | E07 | -:- | 0.46733 | 3.89701 |
| 2031013 | Vlasi-2 | F07 | C:C | 3.61165 | 0.59487 |
| 2031013 | Vlasi-2 | G07 | -:- | 0.45349 | 3.76774 |
| 2031013 | Vlasi-2 | H07 | C:C | 3.76367 | 0.61002 |
| 2031013 | Vlasi-2 | A08 | C:C | 3.42654 | 0.57958 |
| 2031013 | Vlasi-2 | B08 | -:C | 2.28398 | 2.19673 |
| 2031013 | Vlasi-2 | C08 | -:- | 0.49243 | 3.77165 |
| 2031013 | Vlasi-2 | D08 | -:C | 2.20778 | 2.24764 |
| 2031013 | Vlasi-2 | E08 | -:C | 2.19778 | 2.18791 |
| 2031013 | Vlasi-2 | F08 | -:C | 2.29771 | 2.18101 |
| 2031013 | Vlasi-2 | G08 | -:- | 0.4344 | 3.56588 |
| 2031013 | Vlasi-2 | H08 | -:- | 0.46786 | 3.89592 |
| 2031013 | Vlasi-2 | A09 | -:- | 0.46663 | 3.36952 |
| 2031013 | Vlasi-2 | B09 | -:- | 0.46804 | 3.58232 |
| 2031013 | Vlasi-2 | C09 | -:C | 2.15546 | 2.17771 |
| 2031013 | Vlasi-2 | D09 | -:- | 0.43255 | 3.53356 |
| 2031013 | Vlasi-2 | E09 | -:C | 2.24881 | 2.43806 |
| 2031013 | Vlasi-2 | F09 | -:C | 2.06674 | 2.01385 |
| 2031013 | Vlasi-2 | G09 | -:- | 0.44928 | 3.67489 |
| 2031013 | Vlasi-2 | H09 | -:- | 0.4392 | 3.56994 |
| 2031013 | Vlasi-2 | A10 | -:- | 0.64267 | 3.51433 |
| 2031013 | Vlasi-2 | B10 | -:C | 2.31244 | 2.13906 |
| 2031013 | Vlasi-2 | C10 | -:C | 2.25186 | 2.27727 |
| 2031013 | Vlasi-2 | D10 | -:C | 2.19476 | 2.24148 |
| 2031013 | Vlasi-2 | E10 | -:C | 2.2336 | 2.25292 |
| 2031013 | Vlasi-2 | F10 | -:C | 2.26546 | 2.26853 |
| 2031013 | Vlasi-2 | G10 | -:- | 0.4375 | 3.61805 |

| | | | | | |
|---|---|---|---|---|---|
| 2031013 | Vlasi-2 | H10 | -:- | 0.49715 | 3.86162 |
| 2031013 | Vlasi-2 | A11 | -:C | 2.016 | 2.28886 |
| 2031013 | Vlasi-2 | B11 | -:C | 2.2375 | 2.2641 |
| 2031013 | Vlasi-2 | C11 | -:- | 0.45368 | 3.74543 |
| 2031013 | Vlasi-2 | D11 | -:- | 0.42468 | 3.52549 |
| 2031013 | Vlasi-2 | E11 | -:- | 0.43566 | 3.6491 |
| 2031013 | Vlasi-2 | F11 | -:- | 0.43892 | 3.70556 |
| 2031013 | Vlasi-2 | G11 | -:C | 2.16248 | 2.35112 |
| 2031013 | Vlasi-2 | H11 | -:C | 2.14013 | 2.31012 |
| 2031013 | Vlasi-2 | A12 | C:C | 3.39058 | 0.56382 |
| 2031013 | Vlasi-2 | B12 | -:C | 2.18026 | 2.17149 |
| 2031013 | Vlasi-2 | C12 | -:C | 2.2542 | 2.25197 |
| 2031013 | Vlasi-2 | D12 | -:- | 0.42715 | 3.73828 |
| 2031013 | Vlasi-2 | E12 | -:C | 2.28682 | 2.25163 |
| 2031013 | Vlasi-2 | F12 | -:- | 0.44634 | 3.69828 |
| 2031013 | Vlasi-2 | G12 | -:C | 2.43528 | 2.2675 |
| 2031013 | Vlasi-2 | H12 | -:- | 0.45666 | 3.57465 |
| 2031013 | Vlasi-3 | A01 | -:C | 2.39327 | 2.34872 |
| 2031013 | Vlasi-3 | B01 | -:- | 0.4055 | 3.70018 |
| 2031013 | Vlasi-3 | C01 | -:C | 2.41277 | 2.3302 |
| 2031013 | Vlasi-3 | D01 | -:C | 2.35561 | 2.20438 |
| 2031013 | Vlasi-3 | E01 | C:C | 3.86969 | 0.52818 |
| 2031013 | Vlasi-3 | F01 | -:- | 0.40233 | 3.64225 |
| 2031013 | Vlasi-3 | G01 | -:C | 2.37174 | 2.37631 |
| 2031013 | Vlasi-3 | H01 | ? | 0.79273 | 0.67287 |
| 2031013 | Vlasi-3 | A02 | -:- | 0.43222 | 3.61358 |
| 2031013 | Vlasi-3 | B02 | -:C | 2.37904 | 2.21567 |
| 2031013 | Vlasi-3 | C02 | -:- | 0.40391 | 3.61011 |
| 2031013 | Vlasi-3 | D02 | -:- | 0.43092 | 3.6381 |
| 2031013 | Vlasi-3 | E02 | -:C | 2.34078 | 2.16514 |
| 2031013 | Vlasi-3 | F02 | C:C | 3.70822 | 0.60205 |
| 2031013 | Vlasi-3 | G02 | -:C | 2.15227 | 2.13732 |
| 2031013 | Vlasi-3 | H02 | -:C | 2.32066 | 2.24207 |
| 2031013 | Vlasi-3 | A03 | C:C | 3.45117 | 0.55912 |
| 2031013 | Vlasi-3 | B03 | -:C | 2.23072 | 2.17089 |
| 2031013 | Vlasi-3 | C03 | -:- | 0.43632 | 3.63531 |
| 2031013 | Vlasi-3 | D03 | -:C | 2.14982 | 2.18916 |
| 2031013 | Vlasi-3 | E03 | -:C | 2.28691 | 2.21927 |
| 2031013 | Vlasi-3 | F03 | -:- | 0.47362 | 4.00439 |
| 2031013 | Vlasi-3 | G03 | -:C | 2.21515 | 2.16815 |
| 2031013 | Vlasi-3 | H03 | -:C | 2.3682 | 2.39413 |
| 2031013 | Vlasi-3 | A04 | C:C | 3.57278 | 0.55681 |
| 2031013 | Vlasi-3 | B04 | C:C | 3.62805 | 0.52931 |
| 2031013 | Vlasi-3 | C04 | -:- | 0.44076 | 3.62929 |
| 2031013 | Vlasi-3 | D04 | -:C | 2.16886 | 2.32181 |
| 2031013 | Vlasi-3 | E04 | -:- | 0.42346 | 3.68391 |
| 2031013 | Vlasi-3 | F04 | -:C | 2.24527 | 2.36574 |
| 2031013 | Vlasi-3 | G04 | -:C | 2.30177 | 2.23143 |
| 2031013 | Vlasi-3 | H04 | -:C | 2.29746 | 2.31691 |
| 2031013 | Vlasi-3 | A05 | -:C | 2.20507 | 2.27952 |

| | | | | | |
|---|---|---|---|---|---|
| 2031013 | Vlasi-3 | B05 | -:- | 0.46584 | 3.75655 |
| 2031013 | Vlasi-3 | C05 | -:C | 2.21113 | 2.20912 |
| 2031013 | Vlasi-3 | D05 | -:C | 2.32486 | 2.25454 |
| 2031013 | Vlasi-3 | E05 | -:C | 2.21151 | 2.25585 |
| 2031013 | Vlasi-3 | F05 | C:C | 3.66133 | 0.55036 |
| 2031013 | Vlasi-3 | G05 | -:C | 2.18763 | 2.18761 |
| 2031013 | Vlasi-3 | H05 | -:C | 2.41109 | 2.35789 |
| 2031013 | Vlasi-3 | A06 | -:C | 2.13199 | 2.03342 |
| 2031013 | Vlasi-3 | B06 | -:C | 2.14249 | 2.22538 |
| 2031013 | Vlasi-3 | C06 | -:- | 0.4203 | 3.66197 |
| 2031013 | Vlasi-3 | D06 | -:C | 2.21654 | 2.05231 |
| 2031013 | Vlasi-3 | E06 | -:C | 2.26442 | 2.16173 |
| 2031013 | Vlasi-3 | F06 | -:- | 0.47842 | 3.76333 |
| 2031013 | Vlasi-3 | G06 | -:C | 2.32928 | 2.07659 |
| 2031013 | Vlasi-3 | H06 | -:- | 0.479 | 3.64644 |
| 2031013 | Vlasi-3 | A07 | -:C | 2.19525 | 2.2442 |
| 2031013 | Vlasi-3 | B07 | -:- | 0.4316 | 3.54291 |
| 2031013 | Vlasi-3 | C07 | -:- | 0.43918 | 3.64241 |
| 2031013 | Vlasi-3 | D07 | -:- | 0.4238 | 3.66109 |
| 2031013 | Vlasi-3 | E07 | -:C | 2.27354 | 2.11921 |
| 2031013 | Vlasi-3 | F07 | -:C | 2.14108 | 2.22485 |
| 2031013 | Vlasi-3 | G07 | -:C | 2.17432 | 2.26436 |
| 2031013 | Vlasi-3 | H07 | -:C | 2.46072 | 2.32943 |
| 2031013 | Vlasi-3 | A08 | -:- | 0.43241 | 3.34674 |
| 2031013 | Vlasi-3 | B08 | -:- | 0.42881 | 3.65004 |
| 2031013 | Vlasi-3 | C08 | C:C | 3.69496 | 0.55854 |
| 2031013 | Vlasi-3 | D08 | -:C | 2.26916 | 2.18376 |
| 2031013 | Vlasi-3 | E08 | -:C | 2.24837 | 2.12193 |
| 2031013 | Vlasi-3 | F08 | -:C | 2.20528 | 2.21281 |
| 2031013 | Vlasi-3 | G08 | -:C | 2.1736 | 2.0702 |
| 2031013 | Vlasi-3 | H08 | C:C | 3.78502 | 0.66391 |
| 2031013 | Vlasi-3 | A09 | -:- | 0.45513 | 3.53602 |
| 2031013 | Vlasi-3 | B09 | -:- | 0.40167 | 3.546 |
| 2031013 | Vlasi-3 | C09 | -:C | 2.25193 | 2.16005 |
| 2031013 | Vlasi-3 | D09 | -:C | 2.19113 | 2.21347 |
| 2031013 | Vlasi-3 | E09 | -:C | 2.09264 | 2.20691 |
| 2031013 | Vlasi-3 | F09 | -:- | 0.43257 | 3.59498 |
| 2031013 | Vlasi-3 | G09 | -:C | 2.24195 | 2.11878 |
| 2031013 | Vlasi-3 | H09 | -:- | 0.51815 | 3.86921 |
| 2031013 | Vlasi-3 | A10 | C:C | 3.58937 | 0.56393 |
| 2031013 | Vlasi-3 | B10 | -:- | 0.43001 | 3.67472 |
| 2031013 | Vlasi-3 | C10 | -:C | 2.26549 | 2.09068 |
| 2031013 | Vlasi-3 | D10 | -:- | 0.43606 | 3.76931 |
| 2031013 | Vlasi-3 | E10 | -:- | 0.42785 | 3.62984 |
| 2031013 | Vlasi-3 | F10 | -:C | 2.26479 | 2.16432 |
| 2031013 | Vlasi-3 | G10 | -:C | 2.24144 | 2.25085 |
| 2031013 | Vlasi-3 | H10 | -:C | 2.4058 | 2.17165 |
| 2031013 | Vlasi-3 | A11 | -:C | 2.19232 | 2.19991 |
| 2031013 | Vlasi-3 | B11 | -:C | 2.17597 | 2.06093 |
| 2031013 | Vlasi-3 | C11 | -:C | 2.16751 | 2.18823 |

| | | | | | |
|---|---|---|---|---|---|
| 2031013 | Vlasi-3 | D11 | -:- | 0.40637 | 3.44783 |
| 2031013 | Vlasi-3 | E11 | -:C | 2.25565 | 2.2308 |
| 2031013 | Vlasi-3 | F11 | -:C | 2.1089 | 2.14579 |
| 2031013 | Vlasi-3 | G11 | -:C | 2.26134 | 2.27414 |
| 2031013 | Vlasi-3 | H11 | -:- | 0.51198 | 3.83708 |
| 2031013 | Vlasi-3 | A12 | -:- | 0.45957 | 3.42586 |
| 2031013 | Vlasi-3 | B12 | -:C | 2.16507 | 2.21776 |
| 2031013 | Vlasi-3 | C12 | -:C | 2.26008 | 2.25384 |
| 2031013 | Vlasi-3 | D12 | -:- | 0.41587 | 3.66195 |
| 2031013 | Vlasi-3 | E12 | C:C | 3.53605 | 0.53517 |
| 2031013 | Vlasi-3 | F12 | -:- | 0.42156 | 3.64584 |
| 2031013 | Vlasi-3 | G12 | -:C | 2.20125 | 2.18092 |
| 2031013 | Vlasi-3 | H12 | -:C | 2.12458 | 2.4009 |
| 2031013 | Vlasi-4 | A01 | -:- | 0.47574 | 3.54595 |
| 2031013 | Vlasi-4 | B01 | -:C | 2.28246 | 2.20695 |
| 2031013 | Vlasi-4 | C01 | C:C | 3.76046 | 0.55629 |
| 2031013 | Vlasi-4 | D01 | -:C | 2.39884 | 2.25622 |
| 2031013 | Vlasi-4 | E01 | -:C | 2.35284 | 2.07118 |
| 2031013 | Vlasi-4 | F01 | -:C | 2.42133 | 2.20143 |
| 2031013 | Vlasi-4 | G01 | -:C | 2.20244 | 2.13549 |
| 2031013 | Vlasi-4 | H01 | -:- | 0.4979 | 3.53906 |
| 2031013 | Vlasi-4 | A02 | C:C | 3.68926 | 0.5757 |
| 2031013 | Vlasi-4 | B02 | -:- | 0.41672 | 3.52018 |
| 2031013 | Vlasi-4 | C02 | -:- | 0.47579 | 3.67341 |
| 2031013 | Vlasi-4 | D02 | -:C | 2.26365 | 2.22267 |
| 2031013 | Vlasi-4 | E02 | C:C | 3.64346 | 0.56924 |
| 2031013 | Vlasi-4 | F02 | -:C | 2.24398 | 2.23874 |
| 2031013 | Vlasi-4 | G02 | -:- | 0.45927 | 3.57508 |
| 2031013 | Vlasi-4 | H02 | -:- | 0.49245 | 3.6728 |
| 2031013 | Vlasi-4 | A03 | -:C | 2.11047 | 2.36304 |
| 2031013 | Vlasi-4 | B03 | C:C | 3.65333 | 0.53758 |
| 2031013 | Vlasi-4 | C03 | -:- | 0.44251 | 3.67628 |
| 2031013 | Vlasi-4 | D03 | -:C | 2.23158 | 2.282 |
| 2031013 | Vlasi-4 | E03 | -:C | 2.31313 | 2.07061 |
| 2031013 | Vlasi-4 | F03 | -:C | 2.18583 | 2.1845 |
| 2031013 | Vlasi-4 | G03 | -:- | 0.45186 | 3.77669 |
| 2031013 | Vlasi-4 | H03 | -:C | 2.34289 | 2.31006 |
| 2031013 | Vlasi-4 | A04 | -:C | 2.13831 | 2.11111 |
| 2031013 | Vlasi-4 | B04 | -:- | 0.41928 | 3.62841 |
| 2031013 | Vlasi-4 | C04 | C:C | 3.59917 | 0.55318 |
| 2031013 | Vlasi-4 | D04 | -:- | 0.41763 | 3.68309 |
| 2031013 | Vlasi-4 | E04 | -:- | 0.41704 | 3.53922 |
| 2031013 | Vlasi-4 | F04 | -:C | 2.08872 | 2.1963 |
| 2031013 | Vlasi-4 | G04 | -:C | 2.23217 | 2.10446 |
| 2031013 | Vlasi-4 | H04 | C:C | 3.82817 | 0.69442 |
| 2031013 | Vlasi-4 | A05 | -:- | 0.47775 | 3.34568 |
| 2031013 | Vlasi-4 | B05 | -:C | 2.11363 | 2.24332 |
| 2031013 | Vlasi-4 | C05 | C:C | 3.8369 | 0.5693 |
| 2031013 | Vlasi-4 | D05 | C:C | 3.65165 | 0.54002 |
| 2031013 | Vlasi-4 | E05 | -:C | 2.17556 | 2.11776 |

| | | | | | |
|---|---|---|---|---|---|
| 2031013 | Vlasi-4 | F05 | -:- | 0.4213 | 3.53857 |
| 2031013 | Vlasi-4 | G05 | -:C | 2.22147 | 2.16233 |
| 2031013 | Vlasi-4 | H05 | -:- | 0.49754 | 3.65518 |
| 2031013 | Vlasi-4 | A06 | -:C | 2.05709 | 2.02906 |
| 2031013 | Vlasi-4 | B06 | -:C | 2.09892 | 2.26759 |
| 2031013 | Vlasi-4 | C06 | -:C | 2.0953 | 2.23584 |
| 2031013 | Vlasi-4 | D06 | C:C | 3.62374 | 0.58637 |
| 2031013 | Vlasi-4 | E06 | -:- | 0.43527 | 3.61985 |
| 2031013 | Vlasi-4 | F06 | -:- | 0.44603 | 3.64214 |
| 2031013 | Vlasi-4 | G06 | -:C | 2.13231 | 2.23276 |
| 2031013 | Vlasi-4 | H06 | -:- | 0.46376 | 3.64162 |
| 2031013 | Vlasi-4 | A07 | ? | 0.5858 | 0.54497 |
| 2031013 | Vlasi-4 | B07 | ? | 0.55776 | 0.52106 |
| 2031013 | Vlasi-4 | C07 | -:- | 0.44923 | 3.3043 |
| 2031013 | Vlasi-4 | D07 | -:- | 0.47115 | 2.94562 |
| 2031013 | Vlasi-4 | E07 | ? | 0.94342 | 1.10587 |
| 2031013 | Vlasi-4 | F07 | C:C | 3.47886 | 0.62859 |
| 2031013 | Vlasi-4 | G07 | C:C | 3.63069 | 0.64298 |
| 2031013 | Vlasi-4 | H07 | C:C | 3.80685 | 0.85117 |
| 2031013 | Vlasi-4 | A08 | C:C | 3.31987 | 0.68724 |
| 2031013 | Vlasi-4 | B08 | C:C | 3.51358 | 0.71567 |
| 2031013 | Vlasi-4 | C08 | C:C | 3.63536 | 0.73599 |
| 2031013 | Vlasi-4 | D08 | C:C | 3.62517 | 0.69158 |
| 2031013 | Vlasi-4 | E08 | C:C | 3.56598 | 0.68966 |
| 2031013 | Vlasi-4 | F08 | C:C | 3.64966 | 0.64975 |
| 2031013 | Vlasi-4 | G08 | C:C | 3.73581 | 0.73783 |
| 2031013 | Vlasi-4 | H08 | C:C | 3.57534 | 0.82786 |
| 2031013 | Vlasi-4 | A09 | C:C | 3.34156 | 0.70575 |
| 2031013 | Vlasi-4 | B09 | C:C | 3.59376 | 0.649 |
| 2031013 | Vlasi-4 | C09 | C:C | 3.43039 | 0.67179 |
| 2031013 | Vlasi-4 | D09 | C:C | 3.55121 | 0.64871 |
| 2031013 | Vlasi-4 | E09 | C:C | 3.53801 | 0.64743 |
| 2031013 | Vlasi-4 | F09 | C:C | 3.47594 | 0.63153 |
| 2031013 | Vlasi-4 | G09 | C:C | 3.56205 | 0.65759 |
| 2031013 | Vlasi-4 | H09 | C:C | 3.74427 | 0.82503 |
| 2031013 | Vlasi-4 | A10 | C:C | 3.39291 | 0.68635 |
| 2031013 | Vlasi-4 | B10 | C:C | 3.56697 | 0.63343 |
| 2031013 | Vlasi-4 | C10 | -:- | 0.47192 | 3.63133 |
| 2031013 | Vlasi-4 | D10 | -:- | 0.42505 | 3.60511 |
| 2031013 | Vlasi-4 | E10 | -:- | 0.43438 | 3.54968 |
| 2031013 | Vlasi-4 | F10 | -:- | 0.49391 | 3.53413 |
| 2031013 | Vlasi-4 | G10 | -:- | 0.45281 | 3.59192 |
| 2031013 | Vlasi-4 | H10 | -:- | 0.5073 | 3.63053 |
| 2031013 | Vlasi-4 | A11 | -:- | 0.47059 | 3.45158 |
| 2031013 | Vlasi-4 | B11 | -:- | 0.43463 | 3.60031 |
| 2031013 | Vlasi-4 | C11 | -:- | 0.47412 | 3.61671 |
| 2031013 | Vlasi-4 | D11 | -:- | 0.45109 | 3.6768 |
| 2031013 | Vlasi-4 | E11 | -:- | 0.46206 | 3.58857 |
| 2031013 | Vlasi-4 | F11 | -:- | 0.4729 | 3.57497 |
| 2031013 | Vlasi-4 | G11 | -:- | 0.45592 | 3.56375 |

| | | | | | | |
|---|---|---|---|---|---|---|
| 2031013 | Vlasi-4 | H11 | -:- | 0.55104 | 3.53333 |
| 2031013 | Vlasi-4 | A12 | -:- | 0.52886 | 3.34681 |
| 2031013 | Vlasi-4 | B12 | -:- | 0.4734 | 3.5606 |
| 2031013 | Vlasi-4 | C12 | -:- | 0.43837 | 3.47692 |
| 2031013 | Vlasi-4 | D12 | -:- | 0.44779 | 3.55192 |
| 2031013 | Vlasi-4 | E12 | -:- | 0.44136 | 3.49646 |
| 2031013 | Vlasi-4 | F12 | -:- | 0.44797 | 3.48012 |
| 2031013 | Vlasi-4 | G12 | -:- | 0.47185 | 3.54977 |
| 2031013 | Vlasi-4 | H12 | NTC | 0.74068 | 0.68808 |
| 2031013 | Vlasi-1 | A01 | G:G | 3.65169 | 0.49609 |
| 2031013 | Vlasi-1 | B01 | C:C | 0.3852 | 3.75797 |
| 2031013 | Vlasi-1 | C01 | C:G | 2.33342 | 1.84239 |
| 2031013 | Vlasi-1 | D01 | C:G | 2.34318 | 1.75941 |
| 2031013 | Vlasi-1 | E01 | C:G | 2.35495 | 1.84866 |
| 2031013 | Vlasi-1 | F01 | C:G | 2.44584 | 1.85276 |
| 2031013 | Vlasi-1 | G01 | C:G | 2.32416 | 1.84356 |
| 2031013 | Vlasi-1 | H01 | G:G | 3.87688 | 0.57772 |
| 2031013 | Vlasi-1 | A02 | C:C | 0.41309 | 3.62401 |
| 2031013 | Vlasi-1 | B02 | G:G | 3.66851 | 0.54187 |
| 2031013 | Vlasi-1 | C02 | C:G | 2.36298 | 1.89697 |
| 2031013 | Vlasi-1 | D02 | G:G | 3.7832 | 0.49966 |
| 2031013 | Vlasi-1 | E02 | G:G | 3.61471 | 0.49913 |
| 2031013 | Vlasi-1 | F02 | C:G | 2.42608 | 1.9047 |
| 2031013 | Vlasi-1 | G02 | C:G | 2.37589 | 1.90774 |
| 2031013 | Vlasi-1 | H02 | C:G | 2.37664 | 1.88553 |
| 2031013 | Vlasi-1 | A03 | C:G | 2.38545 | 1.5768 |
| 2031013 | Vlasi-1 | B03 | C:G | 2.36946 | 1.80242 |
| 2031013 | Vlasi-1 | C03 | C:C | 0.39596 | 3.71035 |
| 2031013 | Vlasi-1 | D03 | G:G | 3.54203 | 0.53594 |
| 2031013 | Vlasi-1 | E03 | C:G | 2.20786 | 1.80841 |
| 2031013 | Vlasi-1 | F03 | C:G | 2.26847 | 1.86472 |
| 2031013 | Vlasi-1 | G03 | G:G | 3.63879 | 0.56355 |
| 2031013 | Vlasi-1 | H03 | C:G | 2.43037 | 2.05926 |
| 2031013 | Vlasi-1 | A04 | C:G | 2.31501 | 1.48094 |
| 2031013 | Vlasi-1 | B04 | G:G | 3.53988 | 0.49528 |
| 2031013 | Vlasi-1 | C04 | C:G | 2.31446 | 1.78934 |
| 2031013 | Vlasi-1 | D04 | C:G | 2.29072 | 1.89839 |
| 2031013 | Vlasi-1 | E04 | C:G | 2.19652 | 1.8999 |
| 2031013 | Vlasi-1 | F04 | C:G | 2.21827 | 1.91857 |
| 2031013 | Vlasi-1 | G04 | C:G | 2.1771 | 1.7405 |
| 2031013 | Vlasi-1 | H04 | C:G | 2.38487 | 1.77974 |
| 2031013 | Vlasi-1 | A05 | C:C | 0.4065 | 3.52443 |
| 2031013 | Vlasi-1 | B05 | G:G | 3.69035 | 0.5124 |
| 2031013 | Vlasi-1 | C05 | C:G | 2.23434 | 1.83995 |
| 2031013 | Vlasi-1 | D05 | C:G | 2.18991 | 1.85577 |
| 2031013 | Vlasi-1 | E05 | C:C | 0.37857 | 3.46749 |
| 2031013 | Vlasi-1 | F05 | G:G | 3.72492 | 0.54609 |
| 2031013 | Vlasi-1 | G05 | G:G | 3.68659 | 0.61602 |
| 2031013 | Vlasi-1 | H05 | C:G | 2.43859 | 1.79903 |
| 2031013 | Vlasi-1 | A06 | G:G | 3.63405 | 0.52137 |

| | | | | | |
|---|---|---|---|---|---|
| 2031013 | Vlasi-1 | B06 | C:G | 2.14383 | 1.96575 |
| 2031013 | Vlasi-1 | C06 | G:G | 3.67175 | 0.49212 |
| 2031013 | Vlasi-1 | D06 | G:G | 3.61539 | 0.54577 |
| 2031013 | Vlasi-1 | E06 | C:C | 0.36954 | 3.58405 |
| 2031013 | Vlasi-1 | F06 | G:G | 3.59213 | 0.54528 |
| 2031013 | Vlasi-1 | G06 | G:G | 3.6619 | 0.5884 |
| 2031013 | Vlasi-1 | H06 | G:G | 3.5939 | 0.55544 |
| 2031013 | Vlasi-1 | A07 | C:G | 2.27097 | 1.83464 |
| 2031013 | Vlasi-1 | B07 | C:C | 0.42445 | 3.60436 |
| 2031013 | Vlasi-1 | C07 | G:G | 3.69486 | 0.49473 |
| 2031013 | Vlasi-1 | D07 | C:G | 2.29335 | 1.8392 |
| 2031013 | Vlasi-1 | E07 | C:C | 0.40557 | 3.73635 |
| 2031013 | Vlasi-1 | F07 | C:G | 2.18526 | 1.80739 |
| 2031013 | Vlasi-1 | G07 | C:G | 2.32596 | 1.91357 |
| 2031013 | Vlasi-1 | H07 | C:G | 2.34945 | 1.87049 |
| 2031013 | Vlasi-1 | A08 | C:G | 2.35328 | 1.74573 |
| 2031013 | Vlasi-1 | B08 | G:G | 3.67644 | 0.51892 |
| 2031013 | Vlasi-1 | C08 | C:G | 2.25778 | 1.73947 |
| 2031013 | Vlasi-1 | D08 | C:G | 2.20001 | 1.81094 |
| 2031013 | Vlasi-1 | E08 | C:G | 2.19325 | 1.81704 |
| 2031013 | Vlasi-1 | F08 | C:G | 2.18328 | 1.87301 |
| 2031013 | Vlasi-1 | G08 | C:G | 2.37502 | 1.78376 |
| 2031013 | Vlasi-1 | H08 | C:G | 2.3208 | 1.91009 |
| 2031013 | Vlasi-1 | A09 | C:G | 2.13287 | 1.87359 |
| 2031013 | Vlasi-1 | B09 | G:G | 3.55754 | 0.48666 |
| 2031013 | Vlasi-1 | C09 | C:C | 0.39358 | 3.80093 |
| 2031013 | Vlasi-1 | D09 | G:G | 3.58064 | 0.47548 |
| 2031013 | Vlasi-1 | E09 | ? | 0.61595 | 0.39902 |
| 2031013 | Vlasi-1 | F09 | G:G | 3.68242 | 0.50545 |
| 2031013 | Vlasi-1 | G09 | C:G | 2.37439 | 1.87215 |
| 2031013 | Vlasi-1 | H09 | C:G | 2.27795 | 2.00278 |
| 2031013 | Vlasi-1 | A10 | C:G | 2.34644 | 1.79104 |
| 2031013 | Vlasi-1 | B10 | C:G | 2.30087 | 1.89584 |
| 2031013 | Vlasi-1 | C10 | C:G | 2.21125 | 1.88693 |
| 2031013 | Vlasi-1 | D10 | C:C | 0.39484 | 3.69725 |
| 2031013 | Vlasi-1 | E10 | C:G | 2.25919 | 1.80857 |
| 2031013 | Vlasi-1 | F10 | G:G | 3.68866 | 0.52879 |
| 2031013 | Vlasi-1 | G10 | C:G | 2.2751 | 1.91594 |
| 2031013 | Vlasi-1 | H10 | C:C | 0.43989 | 3.76118 |
| 2031013 | Vlasi-1 | A11 | G:G | 3.43623 | 0.57085 |
| 2031013 | Vlasi-1 | B11 | C:G | 2.3163 | 1.73867 |
| 2031013 | Vlasi-1 | C11 | G:G | 3.65887 | 0.51948 |
| 2031013 | Vlasi-1 | D11 | C:G | 2.25663 | 1.91538 |
| 2031013 | Vlasi-1 | E11 | C:G | 2.22968 | 1.87656 |
| 2031013 | Vlasi-1 | F11 | C:G | 2.28059 | 1.77863 |
| 2031013 | Vlasi-1 | G11 | G:G | 3.65302 | 0.56226 |
| 2031013 | Vlasi-1 | H11 | C:C | 0.45711 | 3.87834 |
| 2031013 | Vlasi-1 | A12 | C:G | 2.29266 | 1.69882 |
| 2031013 | Vlasi-1 | B12 | C:G | 2.2867 | 1.91575 |
| 2031013 | Vlasi-1 | C12 | C:C | 0.38356 | 3.57173 |

| | | | | | |
|---|---|---|---|---|---|
| 2031013 | Vlasi-1 | D12 | G:G | 3.78633 | 0.54422 |
| 2031013 | Vlasi-1 | E12 | C:G | 2.41246 | 1.70607 |
| 2031013 | Vlasi-1 | F12 | C:G | 2.37566 | 1.89993 |
| 2031013 | Vlasi-1 | G12 | C:G | 2.39028 | 2.00479 |
| 2031013 | Vlasi-1 | H12 | G:G | 3.74547 | 0.58661 |
| 2031013 | Vlasi-2 | A01 | C:C | 0.45308 | 3.65288 |
| 2031013 | Vlasi-2 | B01 | C:G | 2.49759 | 1.88477 |
| 2031013 | Vlasi-2 | C01 | C:C | 0.39021 | 3.64981 |
| 2031013 | Vlasi-2 | D01 | G:G | 3.84289 | 0.50887 |
| 2031013 | Vlasi-2 | E01 | C:G | 2.44367 | 1.84009 |
| 2031013 | Vlasi-2 | F01 | C:C | 0.43035 | 3.76344 |
| 2031013 | Vlasi-2 | G01 | C:G | 2.444 | 1.84649 |
| 2031013 | Vlasi-2 | H01 | C:C | 0.46712 | 3.81464 |
| 2031013 | Vlasi-2 | A02 | G:G | 3.61681 | 0.53643 |
| 2031013 | Vlasi-2 | B02 | C:G | 2.58053 | 1.74159 |
| 2031013 | Vlasi-2 | C02 | C:C | 0.38636 | 3.52455 |
| 2031013 | Vlasi-2 | D02 | G:G | 3.66423 | 0.52785 |
| 2031013 | Vlasi-2 | E02 | C:G | 2.23997 | 1.80589 |
| 2031013 | Vlasi-2 | F02 | C:C | 0.43328 | 3.64451 |
| 2031013 | Vlasi-2 | G02 | C:C | 0.44032 | 3.9134 |
| 2031013 | Vlasi-2 | H02 | G:G | 3.88778 | 0.64193 |
| 2031013 | Vlasi-2 | A03 | C:C | 0.39895 | 3.39254 |
| 2031013 | Vlasi-2 | B03 | C:C | 0.39411 | 3.70763 |
| 2031013 | Vlasi-2 | C03 | C:C | 0.38419 | 3.65721 |
| 2031013 | Vlasi-2 | D03 | C:C | 0.38385 | 3.79405 |
| 2031013 | Vlasi-2 | E03 | C:G | 2.22417 | 1.76324 |
| 2031013 | Vlasi-2 | F03 | C:G | 2.27655 | 1.76575 |
| 2031013 | Vlasi-2 | G03 | C:C | 0.4698 | 3.7627 |
| 2031013 | Vlasi-2 | H03 | G:G | 3.60366 | 0.58186 |
| 2031013 | Vlasi-2 | A04 | C:G | 2.32882 | 1.74859 |
| 2031013 | Vlasi-2 | B04 | C:G | 2.28978 | 1.77114 |
| 2031013 | Vlasi-2 | C04 | C:G | 2.26702 | 1.86456 |
| 2031013 | Vlasi-2 | D04 | C:C | 0.39022 | 3.78627 |
| 2031013 | Vlasi-2 | E04 | C:C | 0.47818 | 3.8679 |
| 2031013 | Vlasi-2 | F04 | C:G | 2.25578 | 1.91324 |
| 2031013 | Vlasi-2 | G04 | C:G | 2.31854 | 1.82705 |
| 2031013 | Vlasi-2 | H04 | C:C | 0.43045 | 3.62742 |
| 2031013 | Vlasi-2 | A05 | G:G | 3.57636 | 0.50779 |
| 2031013 | Vlasi-2 | B05 | C:C | 0.39348 | 3.70752 |
| 2031013 | Vlasi-2 | C05 | C:C | 0.39477 | 3.57888 |
| 2031013 | Vlasi-2 | D05 | C:C | 0.42153 | 3.90876 |
| 2031013 | Vlasi-2 | E05 | C:G | 2.27855 | 1.76794 |
| 2031013 | Vlasi-2 | F05 | C:G | 2.25169 | 1.71471 |
| 2031013 | Vlasi-2 | G05 | C:G | 2.32335 | 1.74561 |
| 2031013 | Vlasi-2 | H05 | C:G | 2.46081 | 2.0329 |
| 2031013 | Vlasi-2 | A06 | C:G | 2.33288 | 1.65012 |
| 2031013 | Vlasi-2 | B06 | C:G | 2.1756 | 1.90773 |
| 2031013 | Vlasi-2 | C06 | C:G | 2.27267 | 1.69316 |
| 2031013 | Vlasi-2 | D06 | C:G | 2.21358 | 1.88663 |
| 2031013 | Vlasi-2 | E06 | C:C | 0.3927 | 3.72705 |

| | | | | | |
|---|---|---|---|---|---|
| 2031013 | Vlasi-2 | F06 | C:C | 0.39208 | 3.77592 |
| 2031013 | Vlasi-2 | G06 | C:G | 2.35359 | 1.94267 |
| 2031013 | Vlasi-2 | H06 | G:G | 3.64973 | 0.64264 |
| 2031013 | Vlasi-2 | A07 | G:G | 3.33051 | 0.51416 |
| 2031013 | Vlasi-2 | B07 | C:G | 2.38949 | 1.57982 |
| 2031013 | Vlasi-2 | C07 | C:G | 2.27424 | 1.78988 |
| 2031013 | Vlasi-2 | D07 | C:C | 0.39427 | 3.73197 |
| 2031013 | Vlasi-2 | E07 | G:G | 3.70136 | 0.52046 |
| 2031013 | Vlasi-2 | F07 | G:G | 3.51766 | 0.52821 |
| 2031013 | Vlasi-2 | G07 | C:G | 2.23532 | 1.78555 |
| 2031013 | Vlasi-2 | H07 | C:G | 2.4854 | 2.01433 |
| 2031013 | Vlasi-2 | A08 | G:G | 3.54944 | 0.49429 |
| 2031013 | Vlasi-2 | B08 | C:G | 2.29175 | 1.72405 |
| 2031013 | Vlasi-2 | C08 | C:G | 2.46984 | 1.63183 |
| 2031013 | Vlasi-2 | D08 | G:G | 3.82255 | 0.54461 |
| 2031013 | Vlasi-2 | E08 | C:C | 0.40001 | 3.4348 |
| 2031013 | Vlasi-2 | F08 | C:C | 0.41342 | 3.65604 |
| 2031013 | Vlasi-2 | G08 | G:G | 3.57624 | 0.5804 |
| 2031013 | Vlasi-2 | H08 | G:G | 3.73259 | 0.58519 |
| 2031013 | Vlasi-2 | A09 | C:G | 2.31863 | 1.5006 |
| 2031013 | Vlasi-2 | B09 | C:G | 2.38451 | 1.67586 |
| 2031013 | Vlasi-2 | C09 | C:C | 0.51544 | 3.38656 |
| 2031013 | Vlasi-2 | D09 | G:G | 3.66134 | 0.52327 |
| 2031013 | Vlasi-2 | E09 | C:G | 2.26844 | 1.75917 |
| 2031013 | Vlasi-2 | F09 | C:C | 0.43147 | 3.30988 |
| 2031013 | Vlasi-2 | G09 | G:G | 3.83704 | 0.60022 |
| 2031013 | Vlasi-2 | H09 | C:C | 0.4675 | 3.79613 |
| 2031013 | Vlasi-2 | A10 | C:G | 2.36109 | 1.70576 |
| 2031013 | Vlasi-2 | B10 | C:G | 2.31147 | 1.67862 |
| 2031013 | Vlasi-2 | C10 | G:G | 3.88931 | 0.51209 |
| 2031013 | Vlasi-2 | D10 | C:C | 0.38706 | 3.63384 |
| 2031013 | Vlasi-2 | E10 | C:G | 2.29604 | 1.92053 |
| 2031013 | Vlasi-2 | F10 | C:C | 0.41328 | 3.73352 |
| 2031013 | Vlasi-2 | G10 | C:G | 2.26555 | 1.87969 |
| 2031013 | Vlasi-2 | H10 | C:G | 2.37862 | 1.81717 |
| 2031013 | Vlasi-2 | A11 | C:G | 2.27511 | 1.67898 |
| 2031013 | Vlasi-2 | B11 | C:C | 0.38958 | 3.5894 |
| 2031013 | Vlasi-2 | C11 | C:G | 2.41909 | 1.7096 |
| 2031013 | Vlasi-2 | D11 | C:C | 0.36875 | 3.6176 |
| 2031013 | Vlasi-2 | E11 | C:G | 2.31598 | 1.82931 |
| 2031013 | Vlasi-2 | F11 | C:G | 2.39708 | 1.77028 |
| 2031013 | Vlasi-2 | G11 | C:G | 2.39546 | 1.85864 |
| 2031013 | Vlasi-2 | H11 | C:G | 2.34548 | 1.88871 |
| 2031013 | Vlasi-2 | A12 | ? | 0.59864 | 0.44245 |
| 2031013 | Vlasi-2 | B12 | C:G | 2.4397 | 1.80874 |
| 2031013 | Vlasi-2 | C12 | C:G | 2.36383 | 1.92398 |
| 2031013 | Vlasi-2 | D12 | C:G | 2.49082 | 1.82247 |
| 2031013 | Vlasi-2 | E12 | C:C | 0.52312 | 3.61541 |
| 2031013 | Vlasi-2 | F12 | G:G | 3.73255 | 0.46752 |
| 2031013 | Vlasi-2 | G12 | G:G | 3.77423 | 0.5721 |

| | | | | | |
|---|---|---|---|---|---|
| 2031013 | Vlasi-2 | H12 | C:G | 2.60606 | 1.94487 |
| 2031013 | Vlasi-3 | A01 | C:G | 2.42113 | 1.85406 |
| 2031013 | Vlasi-3 | B01 | C:G | 2.48344 | 1.89331 |
| 2031013 | Vlasi-3 | C01 | C:C | 0.38907 | 3.89205 |
| 2031013 | Vlasi-3 | D01 | C:G | 2.29607 | 1.81276 |
| 2031013 | Vlasi-3 | E01 | C:C | 0.36655 | 3.62769 |
| 2031013 | Vlasi-3 | F01 | C:G | 2.27134 | 1.84849 |
| 2031013 | Vlasi-3 | G01 | C:G | 2.40337 | 2.01155 |
| 2031013 | Vlasi-3 | H01 | C:G | 2.57068 | 1.88346 |
| 2031013 | Vlasi-3 | A02 | C:C | 0.41396 | 3.68025 |
| 2031013 | Vlasi-3 | B02 | C:G | 2.3841 | 1.84374 |
| 2031013 | Vlasi-3 | C02 | G:G | 3.67802 | 0.50892 |
| 2031013 | Vlasi-3 | D02 | C:C | 0.37999 | 3.73571 |
| 2031013 | Vlasi-3 | E02 | G:G | 3.75843 | 0.54918 |
| 2031013 | Vlasi-3 | F02 | C:C | 0.49283 | 3.85521 |
| 2031013 | Vlasi-3 | G02 | G:G | 3.83312 | 0.63381 |
| 2031013 | Vlasi-3 | H02 | C:G | 2.39364 | 2.13589 |
| 2031013 | Vlasi-3 | A03 | C:C | 0.44092 | 3.56659 |
| 2031013 | Vlasi-3 | B03 | G:G | 3.48814 | 0.48729 |
| 2031013 | Vlasi-3 | C03 | C:C | 0.50738 | 3.49423 |
| 2031013 | Vlasi-3 | D03 | G:G | 3.4916 | 0.4974 |
| 2031013 | Vlasi-3 | E03 | C:C | 0.39139 | 3.54575 |
| 2031013 | Vlasi-3 | F03 | C:G | 2.29452 | 1.68305 |
| 2031013 | Vlasi-3 | G03 | C:G | 2.37879 | 1.83321 |
| 2031013 | Vlasi-3 | H03 | C:C | 0.47255 | 3.88323 |
| 2031013 | Vlasi-3 | A04 | G:G | 3.74019 | 0.51416 |
| 2031013 | Vlasi-3 | B04 | C:G | 2.31367 | 1.9213 |
| 2031013 | Vlasi-3 | C04 | G:G | 3.68727 | 0.52797 |
| 2031013 | Vlasi-3 | D04 | C:C | 0.37556 | 3.70132 |
| 2031013 | Vlasi-3 | E04 | C:G | 2.23971 | 1.93584 |
| 2031013 | Vlasi-3 | F04 | C:G | 2.18465 | 1.80052 |
| 2031013 | Vlasi-3 | G04 | C:G | 2.30677 | 1.85682 |
| 2031013 | Vlasi-3 | H04 | C:G | 2.53859 | 1.92021 |
| 2031013 | Vlasi-3 | A05 | C:G | 2.41321 | 1.78923 |
| 2031013 | Vlasi-3 | B05 | C:G | 2.17592 | 1.76008 |
| 2031013 | Vlasi-3 | C05 | G:G | 3.67613 | 0.50745 |
| 2031013 | Vlasi-3 | D05 | G:G | 3.61287 | 0.47376 |
| 2031013 | Vlasi-3 | E05 | C:G | 2.29703 | 1.85282 |
| 2031013 | Vlasi-3 | F05 | C:G | 2.23698 | 1.84142 |
| 2031013 | Vlasi-3 | G05 | C:C | 0.43474 | 3.70819 |
| 2031013 | Vlasi-3 | H05 | C:G | 2.51269 | 1.95929 |
| 2031013 | Vlasi-3 | A06 | C:G | 2.20564 | 1.76815 |
| 2031013 | Vlasi-3 | B06 | G:G | 3.74021 | 0.54584 |
| 2031013 | Vlasi-3 | C06 | C:C | 0.39892 | 3.66411 |
| 2031013 | Vlasi-3 | D06 | C:G | 2.24587 | 1.99276 |
| 2031013 | Vlasi-3 | E06 | G:G | 3.5934 | 0.5379 |
| 2031013 | Vlasi-3 | F06 | C:C | 0.41456 | 3.71614 |
| 2031013 | Vlasi-3 | G06 | C:G | 2.32907 | 1.95914 |
| 2031013 | Vlasi-3 | H06 | C:G | 2.53116 | 1.88269 |
| 2031013 | Vlasi-3 | A07 | C:G | 2.27516 | 1.74234 |

| | | | | | |
|---|---|---|---|---|---|
| 2031013 | Vlasi-3 | B07 | C:C | 0.3863 | 3.56201 |
| 2031013 | Vlasi-3 | C07 | G:G | 3.70987 | 0.4681 |
| 2031013 | Vlasi-3 | D07 | G:G | 3.67639 | 0.5447 |
| 2031013 | Vlasi-3 | E07 | C:C | 0.38678 | 3.61657 |
| 2031013 | Vlasi-3 | F07 | G:G | 3.51335 | 0.50519 |
| 2031013 | Vlasi-3 | G07 | C:G | 2.30304 | 1.91043 |
| 2031013 | Vlasi-3 | H07 | C:C | 0.5108 | 3.60498 |
| 2031013 | Vlasi-3 | A08 | C:C | 0.38932 | 3.44923 |
| 2031013 | Vlasi-3 | B08 | C:C | 0.36891 | 3.62376 |
| 2031013 | Vlasi-3 | C08 | G:G | 3.5377 | 0.47418 |
| 2031013 | Vlasi-3 | D08 | C:G | 2.30555 | 1.79415 |
| 2031013 | Vlasi-3 | E08 | C:C | 0.37561 | 3.65142 |
| 2031013 | Vlasi-3 | F08 | C:C | 0.43651 | 3.81936 |
| 2031013 | Vlasi-3 | G08 | C:C | 0.43456 | 3.79248 |
| 2031013 | Vlasi-3 | H08 | C:C | 0.45356 | 3.62764 |
| 2031013 | Vlasi-3 | A09 | G:G | 3.41206 | 0.47099 |
| 2031013 | Vlasi-3 | B09 | C:G | 2.23027 | 1.8611 |
| 2031013 | Vlasi-3 | C09 | C:C | 0.39666 | 3.77971 |
| 2031013 | Vlasi-3 | D09 | G:G | 3.65495 | 0.49758 |
| 2031013 | Vlasi-3 | E09 | G:G | 3.76791 | 0.56349 |
| 2031013 | Vlasi-3 | F09 | C:G | 2.32641 | 1.86159 |
| 2031013 | Vlasi-3 | G09 | C:C | 0.44144 | 3.73518 |
| 2031013 | Vlasi-3 | H09 | C:G | 2.57525 | 2.09223 |
| 2031013 | Vlasi-3 | A10 | C:C | 0.42301 | 3.49318 |
| 2031013 | Vlasi-3 | B10 | G:G | 3.6139 | 0.53709 |
| 2031013 | Vlasi-3 | C10 | C:G | 2.17361 | 1.99209 |
| 2031013 | Vlasi-3 | D10 | C:G | 2.35502 | 1.70432 |
| 2031013 | Vlasi-3 | E10 | C:G | 2.27233 | 1.84221 |
| 2031013 | Vlasi-3 | F10 | C:G | 2.21964 | 1.80075 |
| 2031013 | Vlasi-3 | G10 | C:G | 2.42812 | 1.89085 |
| 2031013 | Vlasi-3 | H10 | C:G | 2.51359 | 2.02241 |
| 2031013 | Vlasi-3 | A11 | C:G | 2.14377 | 1.61987 |
| 2031013 | Vlasi-3 | B11 | C:G | 2.35397 | 1.88933 |
| 2031013 | Vlasi-3 | C11 | G:G | 3.71791 | 0.51843 |
| 2031013 | Vlasi-3 | D11 | C:G | 2.30644 | 1.94161 |
| 2031013 | Vlasi-3 | E11 | C:C | 0.40144 | 3.64466 |
| 2031013 | Vlasi-3 | F11 | C:G | 2.26767 | 1.85504 |
| 2031013 | Vlasi-3 | G11 | G:G | 3.76078 | 0.60434 |
| 2031013 | Vlasi-3 | H11 | ? | 0.6364 | 1.54367 |
| 2031013 | Vlasi-3 | A12 | C:G | 2.33387 | 1.73055 |
| 2031013 | Vlasi-3 | B12 | G:G | 3.71344 | 0.52364 |
| 2031013 | Vlasi-3 | C12 | C:C | 0.37774 | 3.72726 |
| 2031013 | Vlasi-3 | D12 | C:G | 2.38218 | 1.80441 |
| 2031013 | Vlasi-3 | E12 | C:C | 0.38648 | 3.65263 |
| 2031013 | Vlasi-3 | F12 | C:G | 2.36882 | 1.9772 |
| 2031013 | Vlasi-3 | G12 | C:G | 2.47912 | 1.96649 |
| 2031013 | Vlasi-3 | H12 | C:C | 0.48429 | 3.93352 |
| 2031013 | Vlasi-4 | A01 | C:G | 2.46312 | 1.73449 |
| 2031013 | Vlasi-4 | B01 | G:G | 3.76095 | 0.50881 |
| 2031013 | Vlasi-4 | C01 | C:G | 2.30006 | 1.67449 |

| | | | | | |
|---|---|---|---|---|---|
| 2031013 | Vlasi-4 | D01 | C:G | 2.27564 | 1.79184 |
| 2031013 | Vlasi-4 | E01 | C:G | 2.26401 | 1.74183 |
| 2031013 | Vlasi-4 | F01 | C:C | 0.40992 | 3.63671 |
| 2031013 | Vlasi-4 | G01 | C:G | 2.33409 | 1.89428 |
| 2031013 | Vlasi-4 | H01 | C:C | 0.47428 | 3.6614 |
| 2031013 | Vlasi-4 | A02 | C:G | 2.41429 | 1.86616 |
| 2031013 | Vlasi-4 | B02 | C:C | 0.40108 | 3.75497 |
| 2031013 | Vlasi-4 | C02 | C:G | 2.36985 | 1.81582 |
| 2031013 | Vlasi-4 | D02 | G:G | 3.6977 | 0.50516 |
| 2031013 | Vlasi-4 | E02 | C:G | 2.19948 | 1.7579 |
| 2031013 | Vlasi-4 | F02 | C:G | 2.29028 | 1.80871 |
| 2031013 | Vlasi-4 | G02 | C:G | 2.32682 | 1.89032 |
| 2031013 | Vlasi-4 | H02 | C:C | 0.46729 | 3.70154 |
| 2031013 | Vlasi-4 | A03 | C:C | 0.38257 | 3.46617 |
| 2031013 | Vlasi-4 | B03 | C:G | 2.33162 | 1.92473 |
| 2031013 | Vlasi-4 | C03 | C:C | 0.36191 | 3.53146 |
| 2031013 | Vlasi-4 | D03 | G:G | 3.62888 | 0.47991 |
| 2031013 | Vlasi-4 | E03 | C:G | 2.27971 | 1.81524 |
| 2031013 | Vlasi-4 | F03 | C:G | 2.26596 | 1.79145 |
| 2031013 | Vlasi-4 | G03 | C:G | 2.28613 | 2.06885 |
| 2031013 | Vlasi-4 | H03 | C:G | 2.50809 | 1.82053 |
| 2031013 | Vlasi-4 | A04 | C:G | 2.45296 | 1.64198 |
| 2031013 | Vlasi-4 | B04 | G:G | 3.5994 | 0.50986 |
| 2031013 | Vlasi-4 | C04 | C:C | 0.37923 | 3.55759 |
| 2031013 | Vlasi-4 | D04 | G:G | 3.48329 | 0.49065 |
| 2031013 | Vlasi-4 | E04 | C:G | 2.1946 | 1.87364 |
| 2031013 | Vlasi-4 | F04 | C:C | 0.41345 | 3.85113 |
| 2031013 | Vlasi-4 | G04 | C:C | 0.44015 | 3.67213 |
| 2031013 | Vlasi-4 | H04 | C:G | 2.51842 | 1.96397 |
| 2031013 | Vlasi-4 | A05 | C:G | 2.29902 | 1.65099 |
| 2031013 | Vlasi-4 | B05 | C:G | 2.23915 | 1.82966 |
| 2031013 | Vlasi-4 | C05 | G:G | 3.632 | 0.51021 |
| 2031013 | Vlasi-4 | D05 | C:C | 0.39003 | 3.55286 |
| 2031013 | Vlasi-4 | E05 | G:G | 3.5975 | 0.50056 |
| 2031013 | Vlasi-4 | F05 | C:C | 0.41011 | 3.6031 |
| 2031013 | Vlasi-4 | G05 | C:G | 2.24779 | 1.85453 |
| 2031013 | Vlasi-4 | H05 | C:C | 0.45597 | 3.66938 |
| 2031013 | Vlasi-4 | A06 | C:G | 2.20586 | 1.69383 |
| 2031013 | Vlasi-4 | B06 | C:G | 2.29434 | 1.6884 |
| 2031013 | Vlasi-4 | C06 | C:G | 2.20989 | 1.77702 |
| 2031013 | Vlasi-4 | D06 | C:G | 2.10239 | 1.67584 |
| 2031013 | Vlasi-4 | E06 | C:G | 2.10158 | 1.90829 |
| 2031013 | Vlasi-4 | F06 | G:G | 3.7786 | 0.55474 |
| 2031013 | Vlasi-4 | G06 | C:G | 2.23444 | 1.84602 |
| 2031013 | Vlasi-4 | H06 | C:G | 2.3741 | 1.91985 |
| 2031013 | Vlasi-4 | A07 | G:G | 3.30987 | 0.64349 |
| 2031013 | Vlasi-4 | B07 | ? | 0.56142 | 0.52521 |
| 2031013 | Vlasi-4 | C07 | ? | 0.58822 | 0.54738 |
| 2031013 | Vlasi-4 | D07 | ? | 0.59088 | 0.51329 |
| 2031013 | Vlasi-4 | E07 | ? | 0.60455 | 0.609 |

| | | | | | |
|---|---|---|---|---|---|
| 2031013 | Vlasi-4 | F07 | G:G | 3.69283 | 0.5771 |
| 2031013 | Vlasi-4 | G07 | G:G | 3.76048 | 0.57557 |
| 2031013 | Vlasi-4 | H07 | G:G | 4.01757 | 0.73623 |
| 2031013 | Vlasi-4 | A08 | G:G | 3.65451 | 0.58452 |
| 2031013 | Vlasi-4 | B08 | G:G | 3.6513 | 0.53338 |
| 2031013 | Vlasi-4 | C08 | G:G | 3.64544 | 0.54156 |
| 2031013 | Vlasi-4 | D08 | G:G | 3.65682 | 0.54402 |
| 2031013 | Vlasi-4 | E08 | G:G | 3.77031 | 0.56602 |
| 2031013 | Vlasi-4 | F08 | G:G | 3.7419 | 0.58601 |
| 2031013 | Vlasi-4 | G08 | G:G | 3.87298 | 0.6239 |
| 2031013 | Vlasi-4 | H08 | G:G | 3.8899 | 0.72079 |
| 2031013 | Vlasi-4 | A09 | G:G | 3.62955 | 0.55133 |
| 2031013 | Vlasi-4 | B09 | G:G | 3.58274 | 0.61146 |
| 2031013 | Vlasi-4 | C09 | G:G | 3.83704 | 0.57402 |
| 2031013 | Vlasi-4 | D09 | G:G | 3.67806 | 0.56307 |
| 2031013 | Vlasi-4 | E09 | G:G | 3.81512 | 0.55302 |
| 2031013 | Vlasi-4 | F09 | C:G | 2.39905 | 2.04659 |
| 2031013 | Vlasi-4 | G09 | G:G | 3.88144 | 0.63665 |
| 2031013 | Vlasi-4 | H09 | G:G | 3.90736 | 0.67878 |
| 2031013 | Vlasi-4 | A10 | G:G | 3.57424 | 0.58926 |
| 2031013 | Vlasi-4 | B10 | G:G | 3.57303 | 0.53909 |
| 2031013 | Vlasi-4 | C10 | C:C | 0.36783 | 3.5812 |
| 2031013 | Vlasi-4 | D10 | C:C | 0.38483 | 3.62546 |
| 2031013 | Vlasi-4 | E10 | C:C | 0.41705 | 3.60718 |
| 2031013 | Vlasi-4 | F10 | C:C | 0.40908 | 3.58193 |
| 2031013 | Vlasi-4 | G10 | C:C | 0.42457 | 3.51759 |
| 2031013 | Vlasi-4 | H10 | C:C | 0.47671 | 3.74687 |
| 2031013 | Vlasi-4 | A11 | C:C | 0.41433 | 3.48514 |
| 2031013 | Vlasi-4 | B11 | C:C | 0.42899 | 3.84239 |
| 2031013 | Vlasi-4 | C11 | C:C | 0.3965 | 3.65992 |
| 2031013 | Vlasi-4 | D11 | C:C | 0.3925 | 3.52761 |
| 2031013 | Vlasi-4 | E11 | C:C | 0.4123 | 3.56859 |
| 2031013 | Vlasi-4 | F11 | C:C | 0.41056 | 3.6797 |
| 2031013 | Vlasi-4 | G11 | C:C | 0.45493 | 3.72205 |
| 2031013 | Vlasi-4 | H11 | C:C | 0.50112 | 3.66337 |
| 2031013 | Vlasi-4 | A12 | C:C | 0.44986 | 3.47585 |
| 2031013 | Vlasi-4 | B12 | C:C | 0.48894 | 3.26511 |
| 2031013 | Vlasi-4 | C12 | C:C | 0.41397 | 3.49545 |
| 2031013 | Vlasi-4 | D12 | C:C | 0.44998 | 3.60684 |
| 2031013 | Vlasi-4 | E12 | ? | 0.63626 | 0.43995 |
| 2031013 | Vlasi-4 | F12 | C:C | 0.46971 | 3.65968 |
| 2031013 | Vlasi-4 | G12 | C:C | 0.4575 | 3.50601 |
| 2031013 | Vlasi-4 | H12 | NTC | 0.66735 | 0.65563 |
| 2031011 | Vlasi-1 | A01 | C:C | 0.4625 | 3.59521 |
| 2031011 | Vlasi-1 | B01 | C:T | 1.9632 | 2.57678 |
| 2031011 | Vlasi-1 | C01 | C:T | 1.82537 | 2.63422 |
| 2031011 | Vlasi-1 | D01 | C:T | 1.75347 | 2.52834 |
| 2031011 | Vlasi-1 | E01 | C:C | 0.37082 | 3.62987 |
| 2031011 | Vlasi-1 | F01 | C:T | 2.00291 | 2.53268 |
| 2031011 | Vlasi-1 | G01 | C:T | 1.7269 | 2.54201 |

| | | | | | |
|---|---|---|---|---|---|
| 2031011 | Vlasi-1 | H01 | C:C | 0.37224 | 3.68469 |
| 2031011 | Vlasi-1 | A02 | C:C | 0.48778 | 3.29517 |
| 2031011 | Vlasi-1 | B02 | C:T | 1.87639 | 2.45975 |
| 2031011 | Vlasi-1 | C02 | C:C | 0.43969 | 3.50867 |
| 2031011 | Vlasi-1 | D02 | C:C | 0.40915 | 3.56621 |
| 2031011 | Vlasi-1 | E02 | C:T | 1.66199 | 2.27388 |
| 2031011 | Vlasi-1 | F02 | T:T | 3.73746 | 0.48082 |
| 2031011 | Vlasi-1 | G02 | C:C | 0.42088 | 3.51554 |
| 2031011 | Vlasi-1 | H02 | C:T | 1.61105 | 2.48568 |
| 2031011 | Vlasi-1 | A03 | T:T | 3.59067 | 0.52668 |
| 2031011 | Vlasi-1 | B03 | T:T | 3.67111 | 0.53545 |
| 2031011 | Vlasi-1 | C03 | C:C | 0.42742 | 3.53133 |
| 2031011 | Vlasi-1 | D03 | C:T | 1.66403 | 2.50262 |
| 2031011 | Vlasi-1 | E03 | C:T | 1.49585 | 2.27385 |
| 2031011 | Vlasi-1 | F03 | C:C | 0.38247 | 3.43629 |
| 2031011 | Vlasi-1 | G03 | C:T | 1.65217 | 2.49395 |
| 2031011 | Vlasi-1 | H03 | C:T | 1.62768 | 2.53966 |
| 2031011 | Vlasi-1 | A04 | C:T | 1.83754 | 2.47808 |
| 2031011 | Vlasi-1 | B04 | C:C | 0.41636 | 3.46588 |
| 2031011 | Vlasi-1 | C04 | C:C | 0.41739 | 3.63929 |
| 2031011 | Vlasi-1 | D04 | C:T | 1.66265 | 2.38547 |
| 2031011 | Vlasi-1 | E04 | C:T | 1.64848 | 2.25562 |
| 2031011 | Vlasi-1 | F04 | C:T | 1.49791 | 2.39406 |
| 2031011 | Vlasi-1 | G04 | C:C | 0.42396 | 3.50005 |
| 2031011 | Vlasi-1 | H04 | C:C | 0.42364 | 3.5919 |
| 2031011 | Vlasi-1 | A05 | C:C | 0.49506 | 3.55692 |
| 2031011 | Vlasi-1 | B05 | C:T | 1.83228 | 2.25019 |
| 2031011 | Vlasi-1 | C05 | C:T | 1.6834 | 2.42275 |
| 2031011 | Vlasi-1 | D05 | C:T | 1.54761 | 2.49708 |
| 2031011 | Vlasi-1 | E05 | C:C | 0.42004 | 3.57257 |
| 2031011 | Vlasi-1 | F05 | C:C | 0.40856 | 3.50549 |
| 2031011 | Vlasi-1 | G05 | C:C | 0.4257 | 3.52177 |
| 2031011 | Vlasi-1 | H05 | C:T | 1.49399 | 2.54452 |
| 2031011 | Vlasi-1 | A06 | C:T | 1.93295 | 2.47524 |
| 2031011 | Vlasi-1 | B06 | C:T | 1.78451 | 2.30555 |
| 2031011 | Vlasi-1 | C06 | C:T | 1.59104 | 2.49524 |
| 2031011 | Vlasi-1 | D06 | C:C | 0.41501 | 3.68189 |
| 2031011 | Vlasi-1 | E06 | C:T | 1.54768 | 2.30108 |
| 2031011 | Vlasi-1 | F06 | C:T | 1.53269 | 2.46118 |
| 2031011 | Vlasi-1 | G06 | C:T | 1.45004 | 2.00749 |
| 2031011 | Vlasi-1 | H06 | C:C | 0.42985 | 3.59173 |
| 2031011 | Vlasi-1 | A07 | T:T | 3.4531 | 0.5225 |
| 2031011 | Vlasi-1 | B07 | C:T | 1.61264 | 2.30728 |
| 2031011 | Vlasi-1 | C07 | C:T | 1.64046 | 2.29103 |
| 2031011 | Vlasi-1 | D07 | C:C | 0.42464 | 3.61168 |
| 2031011 | Vlasi-1 | E07 | C:C | 0.42036 | 3.66841 |
| 2031011 | Vlasi-1 | F07 | C:C | 0.41368 | 3.65429 |
| 2031011 | Vlasi-1 | G07 | C:C | 0.43485 | 3.62731 |
| 2031011 | Vlasi-1 | H07 | C:C | 0.44822 | 3.65564 |
| 2031011 | Vlasi-1 | A08 | C:T | 1.83357 | 2.52562 |

| | | | | | |
|---|---|---|---|---|---|
| 2031011 | Vlasi-1 | B08 | C:C | 0.43624 | 3.70093 |
| 2031011 | Vlasi-1 | C08 | C:C | 0.39144 | 3.36078 |
| 2031011 | Vlasi-1 | D08 | T:T | 3.63729 | 0.48741 |
| 2031011 | Vlasi-1 | E08 | T:T | 3.58821 | 0.48692 |
| 2031011 | Vlasi-1 | F08 | C:T | 1.63601 | 2.44732 |
| 2031011 | Vlasi-1 | G08 | C:C | 0.43068 | 3.74208 |
| 2031011 | Vlasi-1 | H08 | C:T | 1.67153 | 2.42317 |
| 2031011 | Vlasi-1 | A09 | C:T | 1.72967 | 2.47006 |
| 2031011 | Vlasi-1 | B09 | C:C | 0.41777 | 3.64567 |
| 2031011 | Vlasi-1 | C09 | C:T | 1.68474 | 2.45208 |
| 2031011 | Vlasi-1 | D09 | C:C | 0.42991 | 3.63646 |
| 2031011 | Vlasi-1 | E09 | C:T | 1.58795 | 2.51411 |
| 2031011 | Vlasi-1 | F09 | C:T | 1.74737 | 2.45516 |
| 2031011 | Vlasi-1 | G09 | T:T | 3.61789 | 0.47784 |
| 2031011 | Vlasi-1 | H09 | C:C | 0.43808 | 3.6124 |
| 2031011 | Vlasi-1 | A10 | C:T | 1.75872 | 2.46924 |
| 2031011 | Vlasi-1 | B10 | C:T | 1.60579 | 2.40108 |
| 2031011 | Vlasi-1 | C10 | C:C | 0.39544 | 3.5379 |
| 2031011 | Vlasi-1 | D10 | C:T | 1.58569 | 2.45051 |
| 2031011 | Vlasi-1 | E10 | T:T | 3.54547 | 0.48873 |
| 2031011 | Vlasi-1 | F10 | T:T | 3.49515 | 0.49456 |
| 2031011 | Vlasi-1 | G10 | C:T | 1.52153 | 2.30109 |
| 2031011 | Vlasi-1 | H10 | C:C | 0.42242 | 3.5556 |
| 2031011 | Vlasi-1 | A11 | C:C | 0.44723 | 3.49412 |
| 2031011 | Vlasi-1 | B11 | C:T | 1.77488 | 2.30905 |
| 2031011 | Vlasi-1 | C11 | C:C | 0.39498 | 3.46651 |
| 2031011 | Vlasi-1 | D11 | C:T | 1.54425 | 2.27987 |
| 2031011 | Vlasi-1 | E11 | C:T | 1.71439 | 2.18266 |
| 2031011 | Vlasi-1 | F11 | T:T | 3.70468 | 0.50003 |
| 2031011 | Vlasi-1 | G11 | C:C | 0.40883 | 3.51489 |
| 2031011 | Vlasi-1 | H11 | C:C | 0.43786 | 3.80534 |
| 2031011 | Vlasi-1 | A12 | C:C | 0.48319 | 3.73999 |
| 2031011 | Vlasi-1 | B12 | C:C | 0.39193 | 3.35585 |
| 2031011 | Vlasi-1 | C12 | C:T | 1.79022 | 2.41475 |
| 2031011 | Vlasi-1 | D12 | C:T | 1.90286 | 2.47523 |
| 2031011 | Vlasi-1 | E12 | C:T | 1.66596 | 2.40515 |
| 2031011 | Vlasi-1 | F12 | C:C | 0.42054 | 3.69545 |
| 2031011 | Vlasi-1 | G12 | C:C | 0.42631 | 3.6489 |
| 2031011 | Vlasi-1 | H12 | C:C | 0.44875 | 3.44812 |
| 2031011 | Vlasi-2 | A01 | T:T | 3.52509 | 0.564 |
| 2031011 | Vlasi-2 | B01 | T:T | 3.72825 | 0.50972 |
| 2031011 | Vlasi-2 | C01 | C:T | 1.65704 | 2.62152 |
| 2031011 | Vlasi-2 | D01 | C:C | 0.41701 | 3.68269 |
| 2031011 | Vlasi-2 | E01 | C:C | 0.3896 | 3.56469 |
| 2031011 | Vlasi-2 | F01 | C:T | 1.60543 | 2.35756 |
| 2031011 | Vlasi-2 | G01 | C:T | 1.607 | 2.40424 |
| 2031011 | Vlasi-2 | H01 | C:C | 0.42552 | 3.53901 |
| 2031011 | Vlasi-2 | A02 | C:C | 0.52344 | 3.41289 |
| 2031011 | Vlasi-2 | B02 | C:T | 1.68997 | 2.34424 |
| 2031011 | Vlasi-2 | C02 | C:T | 1.67346 | 2.30854 |

| | | | | | |
|---|---|---|---|---|---|
| 2031011 | Vlasi-2 | D02 | C:C | 0.43636 | 3.73568 |
| 2031011 | Vlasi-2 | E02 | C:C | 0.39558 | 3.4619 |
| 2031011 | Vlasi-2 | F02 | C:C | 0.39445 | 3.53857 |
| 2031011 | Vlasi-2 | G02 | C:T | 1.70723 | 2.13191 |
| 2031011 | Vlasi-2 | H02 | C:C | 0.41952 | 3.58119 |
| 2031011 | Vlasi-2 | A03 | C:T | 1.90606 | 2.48774 |
| 2031011 | Vlasi-2 | B03 | C:T | 1.71557 | 2.42119 |
| 2031011 | Vlasi-2 | C03 | C:T | 1.78633 | 2.34722 |
| 2031011 | Vlasi-2 | D03 | C:T | 1.52897 | 2.36118 |
| 2031011 | Vlasi-2 | E03 | C:T | 1.58856 | 2.39226 |
| 2031011 | Vlasi-2 | F03 | C:T | 1.57253 | 2.36798 |
| 2031011 | Vlasi-2 | G03 | C:T | 1.54633 | 2.53801 |
| 2031011 | Vlasi-2 | H03 | C:T | 1.56186 | 2.42762 |
| 2031011 | Vlasi-2 | A04 | C:T | 1.96084 | 2.6189 |
| 2031011 | Vlasi-2 | B04 | C:T | 1.66615 | 2.35285 |
| 2031011 | Vlasi-2 | C04 | C:C | 0.41773 | 3.56544 |
| 2031011 | Vlasi-2 | D04 | C:T | 1.53048 | 2.30109 |
| 2031011 | Vlasi-2 | E04 | C:T | 1.4557 | 2.25578 |
| 2031011 | Vlasi-2 | F04 | T:T | 3.63427 | 0.50625 |
| 2031011 | Vlasi-2 | G04 | C:C | 0.43031 | 3.49476 |
| 2031011 | Vlasi-2 | H04 | T:T | 3.73019 | 0.51311 |
| 2031011 | Vlasi-2 | A05 | ? | 0.75875 | 2.59578 |
| 2031011 | Vlasi-2 | B05 | T:T | 3.56201 | 0.53328 |
| 2031011 | Vlasi-2 | C05 | C:C | 0.42678 | 3.62118 |
| 2031011 | Vlasi-2 | D05 | C:C | 0.40626 | 3.64059 |
| 2031011 | Vlasi-2 | E05 | C:T | 1.50935 | 2.43239 |
| 2031011 | Vlasi-2 | F05 | C:T | 1.50881 | 2.28514 |
| 2031011 | Vlasi-2 | G05 | C:C | 0.4373 | 3.69945 |
| 2031011 | Vlasi-2 | H05 | C:C | 0.44366 | 3.67757 |
| 2031011 | Vlasi-2 | A06 | C:T | 1.7715 | 2.42992 |
| 2031011 | Vlasi-2 | B06 | C:T | 1.70236 | 2.36287 |
| 2031011 | Vlasi-2 | C06 | C:C | 0.42975 | 3.79837 |
| 2031011 | Vlasi-2 | D06 | C:C | 0.41409 | 3.56868 |
| 2031011 | Vlasi-2 | E06 | C:T | 1.60019 | 2.35775 |
| 2031011 | Vlasi-2 | F06 | ? | 0.44453 | 1.00572 |
| 2031011 | Vlasi-2 | G06 | C:C | 0.42429 | 3.60481 |
| 2031011 | Vlasi-2 | H06 | C:T | 1.73118 | 2.36011 |
| 2031011 | Vlasi-2 | A07 | C:C | 0.43693 | 3.42657 |
| 2031011 | Vlasi-2 | B07 | C:T | 1.63852 | 2.46752 |
| 2031011 | Vlasi-2 | C07 | C:C | 0.40992 | 3.52792 |
| 2031011 | Vlasi-2 | D07 | T:T | 3.70638 | 0.48704 |
| 2031011 | Vlasi-2 | E07 | C:C | 0.4221 | 3.64957 |
| 2031011 | Vlasi-2 | F07 | C:T | 1.58596 | 2.22888 |
| 2031011 | Vlasi-2 | G07 | C:T | 1.60397 | 2.32618 |
| 2031011 | Vlasi-2 | H07 | T:T | 3.82633 | 0.50617 |
| 2031011 | Vlasi-2 | A08 | C:T | 1.76392 | 2.43835 |
| 2031011 | Vlasi-2 | B08 | C:C | 0.42256 | 3.5704 |
| 2031011 | Vlasi-2 | C08 | T:T | 3.52355 | 0.44194 |
| 2031011 | Vlasi-2 | D08 | C:T | 1.64268 | 2.42131 |
| 2031011 | Vlasi-2 | E08 | C:T | 1.60546 | 2.36857 |

| | | | | | |
|---|---|---|---|---|---|
| 2031011 | Vlasi-2 | F08 | C:T | 1.49839 | 2.31975 |
| 2031011 | Vlasi-2 | G08 | C:T | 1.57008 | 2.37049 |
| 2031011 | Vlasi-2 | H08 | C:C | 0.4113 | 3.5951 |
| 2031011 | Vlasi-2 | A09 | C:C | 0.49872 | 3.48203 |
| 2031011 | Vlasi-2 | B09 | C:T | 1.64266 | 2.30699 |
| 2031011 | Vlasi-2 | C09 | C:C | 0.43227 | 3.64022 |
| 2031011 | Vlasi-2 | D09 | C:C | 0.41094 | 3.70274 |
| 2031011 | Vlasi-2 | E09 | T:T | 3.70026 | 0.522 |
| 2031011 | Vlasi-2 | F09 | C:T | 1.22276 | 2.2152 |
| 2031011 | Vlasi-2 | G09 | C:T | 1.62451 | 2.52137 |
| 2031011 | Vlasi-2 | H09 | C:C | 0.42518 | 3.61648 |
| 2031011 | Vlasi-2 | A10 | C:C | 0.50648 | 3.39826 |
| 2031011 | Vlasi-2 | B10 | C:C | 0.42382 | 3.5748 |
| 2031011 | Vlasi-2 | C10 | C:C | 0.41225 | 3.48478 |
| 2031011 | Vlasi-2 | D10 | C:C | 0.40826 | 3.54159 |
| 2031011 | Vlasi-2 | E10 | C:T | 1.5529 | 2.3816 |
| 2031011 | Vlasi-2 | F10 | C:T | 1.60606 | 2.46703 |
| 2031011 | Vlasi-2 | G10 | C:T | 1.59282 | 2.36351 |
| 2031011 | Vlasi-2 | H10 | C:C | 0.52213 | 3.77005 |
| 2031011 | Vlasi-2 | A11 | C:C | 0.47638 | 3.50662 |
| 2031011 | Vlasi-2 | B11 | C:T | 1.775 | 2.32835 |
| 2031011 | Vlasi-2 | C11 | C:C | 0.4263 | 3.48591 |
| 2031011 | Vlasi-2 | D11 | C:T | 1.62083 | 2.31038 |
| 2031011 | Vlasi-2 | E11 | C:C | 0.40771 | 3.56053 |
| 2031011 | Vlasi-2 | F11 | C:T | 1.83382 | 2.34731 |
| 2031011 | Vlasi-2 | G11 | C:C | 0.42853 | 3.62122 |
| 2031011 | Vlasi-2 | H11 | C:T | 1.61345 | 2.54393 |
| 2031011 | Vlasi-2 | A12 | C:T | 1.86631 | 2.58538 |
| 2031011 | Vlasi-2 | B12 | C:T | 1.6658 | 2.39977 |
| 2031011 | Vlasi-2 | C12 | C:C | 0.4254 | 3.53276 |
| 2031011 | Vlasi-2 | D12 | C:C | 0.43622 | 3.59135 |
| 2031011 | Vlasi-2 | E12 | C:T | 1.69381 | 2.42163 |
| 2031011 | Vlasi-2 | F12 | C:C | 0.43239 | 3.62426 |
| 2031011 | Vlasi-2 | G12 | C:C | 0.42505 | 3.67166 |
| 2031011 | Vlasi-2 | H12 | C:C | 0.44223 | 3.6528 |
| 2031011 | Vlasi-3 | A01 | C:T | 2.03788 | 2.68624 |
| 2031011 | Vlasi-3 | B01 | C:C | 0.38383 | 3.72982 |
| 2031011 | Vlasi-3 | C01 | C:C | 0.36118 | 3.46748 |
| 2031011 | Vlasi-3 | D01 | C:C | 0.36456 | 3.69715 |
| 2031011 | Vlasi-3 | E01 | C:C | 0.35747 | 3.64801 |
| 2031011 | Vlasi-3 | F01 | C:C | 0.35694 | 3.5373 |
| 2031011 | Vlasi-3 | G01 | C:T | 1.81687 | 2.46316 |
| 2031011 | Vlasi-3 | H01 | C:C | 0.37614 | 3.62108 |
| 2031011 | Vlasi-3 | A02 | C:C | 0.45797 | 3.56481 |
| 2031011 | Vlasi-3 | B02 | C:C | 0.42322 | 3.5533 |
| 2031011 | Vlasi-3 | C02 | C:C | 0.40273 | 3.48041 |
| 2031011 | Vlasi-3 | D02 | C:C | 0.4368 | 3.63699 |
| 2031011 | Vlasi-3 | E02 | C:T | 1.60368 | 2.39814 |
| 2031011 | Vlasi-3 | F02 | C:C | 0.41001 | 3.65712 |
| 2031011 | Vlasi-3 | G02 | C:C | 0.44728 | 3.64042 |

| | | | | | |
|---|---|---|---|---|---|
| 2031011 | Vlasi-3 | H02 | C:T | 1.70823 | 2.44492 |
| 2031011 | Vlasi-3 | A03 | T:T | 3.66643 | 0.52575 |
| 2031011 | Vlasi-3 | B03 | C:C | 0.42904 | 3.47333 |
| 2031011 | Vlasi-3 | C03 | C:T | 1.66053 | 2.40773 |
| 2031011 | Vlasi-3 | D03 | C:C | 0.41528 | 3.50944 |
| 2031011 | Vlasi-3 | E03 | T:T | 3.72859 | 0.5273 |
| 2031011 | Vlasi-3 | F03 | C:C | 0.42383 | 3.45808 |
| 2031011 | Vlasi-3 | G03 | C:T | 1.55718 | 2.34716 |
| 2031011 | Vlasi-3 | H03 | C:T | 1.55224 | 2.66959 |
| 2031011 | Vlasi-3 | A04 | C:C | 0.42486 | 3.59178 |
| 2031011 | Vlasi-3 | B04 | C:C | 0.41587 | 3.45027 |
| 2031011 | Vlasi-3 | C04 | C:T | 1.57086 | 2.42879 |
| 2031011 | Vlasi-3 | D04 | C:C | 0.4049 | 3.57341 |
| 2031011 | Vlasi-3 | E04 | C:T | 1.77715 | 2.50603 |
| 2031011 | Vlasi-3 | F04 | C:C | 0.41046 | 3.48862 |
| 2031011 | Vlasi-3 | G04 | C:T | 1.52102 | 2.25189 |
| 2031011 | Vlasi-3 | H04 | C:C | 0.44411 | 3.55524 |
| 2031011 | Vlasi-3 | A05 | C:T | 1.89182 | 2.3245 |
| 2031011 | Vlasi-3 | B05 | C:T | 1.80877 | 2.61679 |
| 2031011 | Vlasi-3 | C05 | C:T | 1.89702 | 2.34294 |
| 2031011 | Vlasi-3 | D05 | C:C | 0.44073 | 3.49016 |
| 2031011 | Vlasi-3 | E05 | T:T | 3.5813 | 0.53009 |
| 2031011 | Vlasi-3 | F05 | C:C | 0.40877 | 3.52017 |
| 2031011 | Vlasi-3 | G05 | C:T | 1.70621 | 2.21978 |
| 2031011 | Vlasi-3 | H05 | C:C | 0.41275 | 3.55114 |
| 2031011 | Vlasi-3 | A06 | C:C | 0.43103 | 3.50791 |
| 2031011 | Vlasi-3 | B06 | T:T | 3.62837 | 0.48776 |
| 2031011 | Vlasi-3 | C06 | C:T | 1.62773 | 2.3031 |
| 2031011 | Vlasi-3 | D06 | C:T | 1.65477 | 2.52689 |
| 2031011 | Vlasi-3 | E06 | C:T | 1.49925 | 2.37983 |
| 2031011 | Vlasi-3 | F06 | C:C | 0.40436 | 3.63948 |
| 2031011 | Vlasi-3 | G06 | C:C | 0.42176 | 3.60147 |
| 2031011 | Vlasi-3 | H06 | C:C | 0.4402 | 3.70681 |
| 2031011 | Vlasi-3 | A07 | C:T | 1.73316 | 2.38775 |
| 2031011 | Vlasi-3 | B07 | C:T | 1.60307 | 2.3831 |
| 2031011 | Vlasi-3 | C07 | C:C | 0.43614 | 3.7726 |
| 2031011 | Vlasi-3 | D07 | C:C | 0.41695 | 3.73075 |
| 2031011 | Vlasi-3 | E07 | C:T | 1.57813 | 2.44951 |
| 2031011 | Vlasi-3 | F07 | T:T | 3.55584 | 0.47234 |
| 2031011 | Vlasi-3 | G07 | C:C | 0.40218 | 3.57662 |
| 2031011 | Vlasi-3 | H07 | C:C | 0.45185 | 3.61099 |
| 2031011 | Vlasi-3 | A08 | C:T | 1.73398 | 2.23272 |
| 2031011 | Vlasi-3 | B08 | C:T | 1.73871 | 2.24943 |
| 2031011 | Vlasi-3 | C08 | C:T | 1.63082 | 2.31326 |
| 2031011 | Vlasi-3 | D08 | T:T | 3.42753 | 0.49285 |
| 2031011 | Vlasi-3 | E08 | T:T | 3.53641 | 0.45804 |
| 2031011 | Vlasi-3 | F08 | C:C | 0.44348 | 3.59727 |
| 2031011 | Vlasi-3 | G08 | C:T | 1.387 | 2.3051 |
| 2031011 | Vlasi-3 | H08 | C:C | 0.4341 | 3.47144 |
| 2031011 | Vlasi-3 | A09 | C:T | 1.67 | 2.48377 |

| | | | | | |
|---|---|---|---|---|---|
| 2031011 | Vlasi-3 | B09 | C:T | 1.69523 | 2.40311 |
| 2031011 | Vlasi-3 | C09 | C:T | 1.62008 | 2.34882 |
| 2031011 | Vlasi-3 | D09 | C:C | 0.40657 | 3.56453 |
| 2031011 | Vlasi-3 | E09 | C:T | 1.75762 | 2.26585 |
| 2031011 | Vlasi-3 | F09 | C:T | 1.53659 | 2.41381 |
| 2031011 | Vlasi-3 | G09 | C:T | 1.69632 | 2.34933 |
| 2031011 | Vlasi-3 | H09 | C:T | 1.75795 | 2.52398 |
| 2031011 | Vlasi-3 | A10 | C:T | 1.68871 | 2.40307 |
| 2031011 | Vlasi-3 | B10 | C:C | 0.40771 | 3.42966 |
| 2031011 | Vlasi-3 | C10 | C:C | 0.40238 | 3.46839 |
| 2031011 | Vlasi-3 | D10 | C:T | 1.62543 | 2.32171 |
| 2031011 | Vlasi-3 | E10 | C:C | 0.42452 | 3.72433 |
| 2031011 | Vlasi-3 | F10 | C:T | 1.57749 | 2.45957 |
| 2031011 | Vlasi-3 | G10 | C:C | 0.40706 | 3.47262 |
| 2031011 | Vlasi-3 | H10 | T:T | 3.62944 | 0.47558 |
| 2031011 | Vlasi-3 | A11 | C:T | 1.76822 | 2.47932 |
| 2031011 | Vlasi-3 | B11 | C:C | 0.43646 | 3.52736 |
| 2031011 | Vlasi-3 | C11 | C:C | 0.42299 | 3.49432 |
| 2031011 | Vlasi-3 | D11 | ? | 1.00469 | 2.23859 |
| 2031011 | Vlasi-3 | E11 | C:T | 1.72852 | 2.33092 |
| 2031011 | Vlasi-3 | F11 | C:T | 1.73301 | 2.33018 |
| 2031011 | Vlasi-3 | G11 | T:T | 3.66046 | 0.53556 |
| 2031011 | Vlasi-3 | H11 | T:T | 3.79448 | 0.54987 |
| 2031011 | Vlasi-3 | A12 | T:T | 3.53159 | 0.51397 |
| 2031011 | Vlasi-3 | B12 | C:C | 0.42656 | 3.64464 |
| 2031011 | Vlasi-3 | C12 | C:C | 0.42968 | 3.68255 |
| 2031011 | Vlasi-3 | D12 | C:T | 1.72531 | 2.4138 |
| 2031011 | Vlasi-3 | E12 | T:T | 3.72808 | 0.53164 |
| 2031011 | Vlasi-3 | F12 | C:T | 1.72953 | 2.3496 |
| 2031011 | Vlasi-3 | G12 | C:T | 1.69419 | 2.44032 |
| 2031011 | Vlasi-3 | H12 | C:C | 0.44363 | 3.66437 |
| 2031011 | Vlasi-4 | A01 | T:T | 3.60591 | 0.60481 |
| 2031011 | Vlasi-4 | B01 | ? | 1.90405 | 0.6342 |
| 2031011 | Vlasi-4 | C01 | C:C | 0.3984 | 3.50882 |
| 2031011 | Vlasi-4 | D01 | C:T | 1.53523 | 2.32638 |
| 2031011 | Vlasi-4 | E01 | C:T | 1.56684 | 2.20801 |
| 2031011 | Vlasi-4 | F01 | C:C | 0.39324 | 3.42605 |
| 2031011 | Vlasi-4 | G01 | C:C | 0.39916 | 3.50397 |
| 2031011 | Vlasi-4 | H01 | T:T | 3.70561 | 0.51164 |
| 2031011 | Vlasi-4 | A02 | C:T | 1.93766 | 2.51145 |
| 2031011 | Vlasi-4 | B02 | C:C | 0.43566 | 3.47218 |
| 2031011 | Vlasi-4 | C02 | C:T | 1.61324 | 2.46263 |
| 2031011 | Vlasi-4 | D02 | C:T | 1.61768 | 2.52962 |
| 2031011 | Vlasi-4 | E02 | C:C | 0.40542 | 3.57141 |
| 2031011 | Vlasi-4 | F02 | C:T | 1.59938 | 2.3281 |
| 2031011 | Vlasi-4 | G02 | C:C | 0.45172 | 3.58967 |
| 2031011 | Vlasi-4 | H02 | C:C | 0.44591 | 3.64206 |
| 2031011 | Vlasi-4 | A03 | C:T | 1.78905 | 2.41074 |
| 2031011 | Vlasi-4 | B03 | C:T | 1.6807 | 2.36351 |
| 2031011 | Vlasi-4 | C03 | C:C | 0.41403 | 3.5532 |

| | | | | | |
|---|---|---|---|---|---|
| 2031011 | Vlasi-4 | D03 | C:T | 1.56486 | 2.29015 |
| 2031011 | Vlasi-4 | E03 | ? | 0.7448 | 2.35302 |
| 2031011 | Vlasi-4 | F03 | C:T | 1.55326 | 2.32798 |
| 2031011 | Vlasi-4 | G03 | C:T | 1.60888 | 2.43588 |
| 2031011 | Vlasi-4 | H03 | C:C | 0.43773 | 3.62038 |
| 2031011 | Vlasi-4 | A04 | C:C | 0.43448 | 3.35387 |
| 2031011 | Vlasi-4 | B04 | C:T | 1.81567 | 2.28751 |
| 2031011 | Vlasi-4 | C04 | C:T | 1.69859 | 2.5455 |
| 2031011 | Vlasi-4 | D04 | C:C | 0.40648 | 3.48463 |
| 2031011 | Vlasi-4 | E04 | C:T | 1.5449 | 2.28416 |
| 2031011 | Vlasi-4 | F04 | C:C | 0.41371 | 3.55736 |
| 2031011 | Vlasi-4 | G04 | C:C | 0.41702 | 3.4966 |
| 2031011 | Vlasi-4 | H04 | C:C | 0.43012 | 3.57309 |
| 2031011 | Vlasi-4 | A05 | C:C | 0.42401 | 3.42294 |
| 2031011 | Vlasi-4 | B05 | C:T | 1.58996 | 2.31176 |
| 2031011 | Vlasi-4 | C05 | C:T | 1.56941 | 2.39306 |
| 2031011 | Vlasi-4 | D05 | C:T | 1.44155 | 2.46082 |
| 2031011 | Vlasi-4 | E05 | C:C | 0.39328 | 3.42499 |
| 2031011 | Vlasi-4 | F05 | C:C | 0.42094 | 3.46501 |
| 2031011 | Vlasi-4 | G05 | T:T | 3.6121 | 0.49702 |
| 2031011 | Vlasi-4 | H05 | C:T | 1.75442 | 2.449 |
| 2031011 | Vlasi-4 | A06 | T:T | 3.55159 | 0.49268 |
| 2031011 | Vlasi-4 | B06 | C:T | 1.70535 | 2.08404 |
| 2031011 | Vlasi-4 | C06 | C:T | 1.66955 | 2.2835 |
| 2031011 | Vlasi-4 | D06 | C:T | 1.57431 | 2.29048 |
| 2031011 | Vlasi-4 | E06 | C:C | 0.39585 | 3.52886 |
| 2031011 | Vlasi-4 | F06 | C:C | 0.40902 | 3.37921 |
| 2031011 | Vlasi-4 | G06 | C:C | 0.43889 | 3.50815 |
| 2031011 | Vlasi-4 | H06 | C:T | 1.67088 | 2.44059 |
| 2031011 | Vlasi-4 | A07 | C:C | 0.38953 | 3.17664 |
| 2031011 | Vlasi-4 | B07 | ? | 0.53143 | 0.61204 |
| 2031011 | Vlasi-4 | C07 | C:C | 0.4419 | 3.32829 |
| 2031011 | Vlasi-4 | D07 | ? | 0.58036 | 0.54085 |
| 2031011 | Vlasi-4 | E07 | ? | 0.61051 | 0.52656 |
| 2031011 | Vlasi-4 | F07 | T:T | 3.68921 | 0.62323 |
| 2031011 | Vlasi-4 | G07 | T:T | 3.67746 | 0.5743 |
| 2031011 | Vlasi-4 | H07 | T:T | 3.70882 | 0.634 |
| 2031011 | Vlasi-4 | A08 | T:T | 3.53152 | 0.63742 |
| 2031011 | Vlasi-4 | B08 | T:T | 3.428 | 0.62718 |
| 2031011 | Vlasi-4 | C08 | T:T | 3.60921 | 0.6448 |
| 2031011 | Vlasi-4 | D08 | T:T | 3.50326 | 0.62386 |
| 2031011 | Vlasi-4 | E08 | T:T | 3.55701 | 0.60413 |
| 2031011 | Vlasi-4 | F08 | T:T | 3.59291 | 0.59911 |
| 2031011 | Vlasi-4 | G08 | T:T | 3.6734 | 0.62624 |
| 2031011 | Vlasi-4 | H08 | T:T | 3.65302 | 0.62209 |
| 2031011 | Vlasi-4 | A09 | T:T | 3.37048 | 0.69135 |
| 2031011 | Vlasi-4 | B09 | T:T | 3.37282 | 0.57255 |
| 2031011 | Vlasi-4 | C09 | T:T | 3.47738 | 0.70281 |
| 2031011 | Vlasi-4 | D09 | T:T | 3.61166 | 0.63391 |
| 2031011 | Vlasi-4 | E09 | T:T | 3.56337 | 0.59329 |

| | | | | | |
|---|---|---|---|---|---|
| 2031011 | Vlasi-4 | F09 | T:T | 3.55767 | 0.57874 |
| 2031011 | Vlasi-4 | G09 | T:T | 3.59173 | 0.61963 |
| 2031011 | Vlasi-4 | H09 | T:T | 3.77497 | 0.59601 |
| 2031011 | Vlasi-4 | A10 | T:T | 3.24588 | 0.72984 |
| 2031011 | Vlasi-4 | B10 | T:T | 3.49867 | 0.60562 |
| 2031011 | Vlasi-4 | C10 | C:C | 0.39741 | 3.59996 |
| 2031011 | Vlasi-4 | D10 | C:C | 0.3884 | 3.51648 |
| 2031011 | Vlasi-4 | E10 | C:C | 0.44317 | 3.54794 |
| 2031011 | Vlasi-4 | F10 | C:C | 0.40924 | 3.39677 |
| 2031011 | Vlasi-4 | G10 | C:C | 0.42633 | 3.44534 |
| 2031011 | Vlasi-4 | H10 | C:C | 0.4425 | 3.58612 |
| 2031011 | Vlasi-4 | A11 | C:C | 0.46098 | 3.48063 |
| 2031011 | Vlasi-4 | B11 | C:C | 0.43861 | 3.59205 |
| 2031011 | Vlasi-4 | C11 | C:C | 0.43709 | 3.59381 |
| 2031011 | Vlasi-4 | D11 | C:C | 0.40884 | 3.42687 |
| 2031011 | Vlasi-4 | E11 | C:C | 0.4442 | 3.34088 |
| 2031011 | Vlasi-4 | F11 | C:C | 0.40382 | 3.27564 |
| 2031011 | Vlasi-4 | G11 | C:C | 0.44225 | 3.63107 |
| 2031011 | Vlasi-4 | H11 | C:C | 0.44394 | 3.48137 |
| 2031011 | Vlasi-4 | A12 | C:C | 0.50413 | 3.43878 |
| 2031011 | Vlasi-4 | B12 | C:C | 0.4745 | 3.4529 |
| 2031011 | Vlasi-4 | C12 | C:C | 0.46877 | 3.48698 |
| 2031011 | Vlasi-4 | D12 | C:C | 0.47486 | 3.42022 |
| 2031011 | Vlasi-4 | E12 | C:C | 0.50851 | 3.51822 |
| 2031011 | Vlasi-4 | F12 | C:C | 0.50313 | 3.49477 |
| 2031011 | Vlasi-4 | G12 | C:C | 0.47734 | 3.51845 |
| 2031011 | Vlasi-4 | H12 | NTC | 0.60943 | 0.53782 |
| 2031011 | Vlasi-1 | A01 | ? | 0.54993 | 0.42953 |
| 2031011 | Vlasi-1 | B01 | G:A | 1.41184 | 2.89965 |
| 2031011 | Vlasi-1 | C01 | G:A | 1.1985 | 2.78086 |
| 2031011 | Vlasi-1 | D01 | G:A | 1.17416 | 2.68211 |
| 2031011 | Vlasi-1 | E01 | G:G | 0.34431 | 3.68782 |
| 2031011 | Vlasi-1 | F01 | G:A | 1.11414 | 2.88001 |
| 2031011 | Vlasi-1 | G01 | G:G | 0.34762 | 3.56416 |
| 2031011 | Vlasi-1 | H01 | G:G | 0.38768 | 3.5699 |
| 2031011 | Vlasi-1 | A02 | G:G | 0.46186 | 3.10421 |
| 2031011 | Vlasi-1 | B02 | G:G | 0.43517 | 3.52225 |
| 2031011 | Vlasi-1 | C02 | G:G | 0.41358 | 3.49342 |
| 2031011 | Vlasi-1 | D02 | G:A | 1.15745 | 2.68363 |
| 2031011 | Vlasi-1 | E02 | G:G | 0.41405 | 3.55986 |
| 2031011 | Vlasi-1 | F02 | G:G | 0.41796 | 3.62296 |
| 2031011 | Vlasi-1 | G02 | G:A | 0.99398 | 2.79029 |
| 2031011 | Vlasi-1 | H02 | A:A | 3.37419 | 0.5118 |
| 2031011 | Vlasi-1 | A03 | G:A | 1.45262 | 2.54653 |
| 2031011 | Vlasi-1 | B03 | G:A | 1.27562 | 2.53156 |
| 2031011 | Vlasi-1 | C03 | G:G | 0.40345 | 3.44474 |
| 2031011 | Vlasi-1 | D03 | G:A | 1.2431 | 2.83161 |
| 2031011 | Vlasi-1 | E03 | G:A | 1.08593 | 2.68689 |
| 2031011 | Vlasi-1 | F03 | G:G | 0.41756 | 3.38447 |
| 2031011 | Vlasi-1 | G03 | G:A | 1.01185 | 2.65851 |

| | | | | | |
|---|---|---|---|---|---|
| 2031011 | Vlasi-1 | H03 | G:G | 0.41693 | 3.44836 |
| 2031011 | Vlasi-1 | A04 | G:A | 1.44939 | 2.58318 |
| 2031011 | Vlasi-1 | B04 | G:A | 1.35668 | 2.515 |
| 2031011 | Vlasi-1 | C04 | G:G | 0.38818 | 3.37928 |
| 2031011 | Vlasi-1 | D04 | G:A | 1.10891 | 2.68193 |
| 2031011 | Vlasi-1 | E04 | G:G | 0.38395 | 3.45246 |
| 2031011 | Vlasi-1 | F04 | G:A | 1.10103 | 2.57186 |
| 2031011 | Vlasi-1 | G04 | G:A | 1.08267 | 2.82963 |
| 2031011 | Vlasi-1 | H04 | G:G | 0.41105 | 3.72135 |
| 2031011 | Vlasi-1 | A05 | G:G | 0.44507 | 3.22426 |
| 2031011 | Vlasi-1 | B05 | G:A | 1.24581 | 2.68782 |
| 2031011 | Vlasi-1 | C05 | G:A | 1.0605 | 2.75805 |
| 2031011 | Vlasi-1 | D05 | A:A | 3.43361 | 0.47666 |
| 2031011 | Vlasi-1 | E05 | G:G | 0.38736 | 3.5089 |
| 2031011 | Vlasi-1 | F05 | G:G | 0.41075 | 3.43941 |
| 2031011 | Vlasi-1 | G05 | G:A | 0.99559 | 2.69586 |
| 2031011 | Vlasi-1 | H05 | G:G | 0.41052 | 3.42527 |
| 2031011 | Vlasi-1 | A06 | A:A | 3.44116 | 0.45948 |
| 2031011 | Vlasi-1 | B06 | G:G | 0.38537 | 3.28636 |
| 2031011 | Vlasi-1 | C06 | G:A | 1.03412 | 2.6983 |
| 2031011 | Vlasi-1 | D06 | G:G | 0.40691 | 3.45041 |
| 2031011 | Vlasi-1 | E06 | G:G | 0.37034 | 3.36839 |
| 2031011 | Vlasi-1 | F06 | G:A | 1.02656 | 2.66505 |
| 2031011 | Vlasi-1 | G06 | G:G | 0.39567 | 3.29413 |
| 2031011 | Vlasi-1 | H06 | G:G | 0.40193 | 3.51738 |
| 2031011 | Vlasi-1 | A07 | G:G | 0.47429 | 3.32579 |
| 2031011 | Vlasi-1 | B07 | G:G | 0.42205 | 3.45721 |
| 2031011 | Vlasi-1 | C07 | G:G | 0.40436 | 3.39217 |
| 2031011 | Vlasi-1 | D07 | G:G | 0.37411 | 3.40667 |
| 2031011 | Vlasi-1 | E07 | G:G | 0.41686 | 3.45552 |
| 2031011 | Vlasi-1 | F07 | G:G | 0.3877 | 3.55718 |
| 2031011 | Vlasi-1 | G07 | G:A | 1.04803 | 2.61974 |
| 2031011 | Vlasi-1 | H07 | G:G | 0.41195 | 3.46879 |
| 2031011 | Vlasi-1 | A08 | G:A | 1.22403 | 2.75672 |
| 2031011 | Vlasi-1 | B08 | G:G | 0.39969 | 3.4731 |
| 2031011 | Vlasi-1 | C08 | A:A | 3.46193 | 0.46946 |
| 2031011 | Vlasi-1 | D08 | G:A | 1.07691 | 2.77267 |
| 2031011 | Vlasi-1 | E08 | G:A | 1.1596 | 2.78875 |
| 2031011 | Vlasi-1 | F08 | G:A | 1.14797 | 2.68096 |
| 2031011 | Vlasi-1 | G08 | G:G | 0.40698 | 3.37388 |
| 2031011 | Vlasi-1 | H08 | G:A | 1.05491 | 2.59532 |
| 2031011 | Vlasi-1 | A09 | G:G | 0.44152 | 3.3314 |
| 2031011 | Vlasi-1 | B09 | G:G | 0.40687 | 3.45386 |
| 2031011 | Vlasi-1 | C09 | A:A | 3.58075 | 0.4706 |
| 2031011 | Vlasi-1 | D09 | G:G | 0.40392 | 3.49715 |
| 2031011 | Vlasi-1 | E09 | G:A | 1.20401 | 2.79685 |
| 2031011 | Vlasi-1 | F09 | G:A | 1.10519 | 2.88396 |
| 2031011 | Vlasi-1 | G09 | G:G | 0.39923 | 3.55178 |
| 2031011 | Vlasi-1 | H09 | G:G | 0.42435 | 3.64576 |
| 2031011 | Vlasi-1 | A10 | G:A | 1.33062 | 2.60367 |

| | | | | | |
|---|---|---|---|---|---|
| 2031011 | Vlasi-1 | B10 | G:A | 1.18231 | 2.67134 |
| 2031011 | Vlasi-1 | C10 | G:G | 0.35828 | 3.39697 |
| 2031011 | Vlasi-1 | D10 | G:A | 1.08467 | 2.76667 |
| 2031011 | Vlasi-1 | E10 | G:A | 1.09786 | 2.62365 |
| 2031011 | Vlasi-1 | F10 | G:A | 1.08169 | 2.75905 |
| 2031011 | Vlasi-1 | G10 | A:A | 3.5218 | 0.4938 |
| 2031011 | Vlasi-1 | H10 | G:G | 0.38905 | 3.39888 |
| 2031011 | Vlasi-1 | A11 | G:G | 0.42253 | 3.3669 |
| 2031011 | Vlasi-1 | B11 | G:A | 1.22435 | 2.60628 |
| 2031011 | Vlasi-1 | C11 | G:A | 1.04699 | 2.71936 |
| 2031011 | Vlasi-1 | D11 | G:G | 0.39005 | 3.64477 |
| 2031011 | Vlasi-1 | E11 | G:G | 0.38999 | 3.49176 |
| 2031011 | Vlasi-1 | F11 | A:A | 3.49427 | 0.46582 |
| 2031011 | Vlasi-1 | G11 | G:A | 1.12651 | 2.72786 |
| 2031011 | Vlasi-1 | H11 | G:G | 0.42401 | 3.60867 |
| 2031011 | Vlasi-1 | A12 | G:A | 1.21232 | 2.71322 |
| 2031011 | Vlasi-1 | B12 | G:G | 0.39779 | 3.46677 |
| 2031011 | Vlasi-1 | C12 | G:A | 1.24747 | 2.6165 |
| 2031011 | Vlasi-1 | D12 | G:G | 0.3929 | 3.64713 |
| 2031011 | Vlasi-1 | E12 | G:A | 1.2262 | 2.77484 |
| 2031011 | Vlasi-1 | F12 | G:G | 0.3978 | 3.57334 |
| 2031011 | Vlasi-1 | G12 | G:G | 0.41671 | 3.60215 |
| 2031011 | Vlasi-1 | H12 | G:G | 0.40582 | 3.57517 |
| 2031011 | Vlasi-2 | A01 | G:A | 1.56959 | 2.67395 |
| 2031011 | Vlasi-2 | B01 | G:A | 1.29779 | 2.73903 |
| 2031011 | Vlasi-2 | C01 | G:A | 1.1529 | 2.73977 |
| 2031011 | Vlasi-2 | D01 | G:A | 1.06593 | 2.55531 |
| 2031011 | Vlasi-2 | E01 | G:G | 0.40336 | 3.50816 |
| 2031011 | Vlasi-2 | F01 | G:A | 1.10938 | 2.69143 |
| 2031011 | Vlasi-2 | G01 | G:A | 1.09017 | 2.70447 |
| 2031011 | Vlasi-2 | H01 | G:G | 0.41921 | 3.5455 |
| 2031011 | Vlasi-2 | A02 | G:A | 1.56915 | 2.59012 |
| 2031011 | Vlasi-2 | B02 | G:A | 1.33037 | 2.65849 |
| 2031011 | Vlasi-2 | C02 | G:A | 1.2705 | 2.77629 |
| 2031011 | Vlasi-2 | D02 | G:G | 0.3895 | 3.53505 |
| 2031011 | Vlasi-2 | E02 | G:G | 0.38985 | 3.42382 |
| 2031011 | Vlasi-2 | F02 | G:G | 0.39451 | 3.37247 |
| 2031011 | Vlasi-2 | G02 | G:G | 0.40877 | 3.54535 |
| 2031011 | Vlasi-2 | H02 | G:A | 1.08147 | 2.64842 |
| 2031011 | Vlasi-2 | A03 | G:A | 1.61331 | 2.62534 |
| 2031011 | Vlasi-2 | B03 | G:A | 1.18118 | 2.69224 |
| 2031011 | Vlasi-2 | C03 | A:A | 3.7263 | 0.51439 |
| 2031011 | Vlasi-2 | D03 | G:A | 1.17217 | 2.85481 |
| 2031011 | Vlasi-2 | E03 | G:A | 1.13637 | 2.65522 |
| 2031011 | Vlasi-2 | F03 | G:A | 0.97242 | 2.57515 |
| 2031011 | Vlasi-2 | G03 | G:G | 0.43795 | 3.62952 |
| 2031011 | Vlasi-2 | H03 | G:G | 0.41578 | 3.47366 |
| 2031011 | Vlasi-2 | A04 | G:G | 0.47499 | 3.35252 |
| 2031011 | Vlasi-2 | B04 | G:A | 1.31006 | 2.64105 |
| 2031011 | Vlasi-2 | C04 | G:A | 1.23454 | 2.62774 |

| | | | | | |
|---|---|---|---|---|---|
| 2031011 | Vlasi-2 | D04 | G:A | 1.12347 | 2.74566 |
| 2031011 | Vlasi-2 | E04 | G:A | 1.16617 | 2.78832 |
| 2031011 | Vlasi-2 | F04 | G:G | 0.40594 | 3.43612 |
| 2031011 | Vlasi-2 | G04 | G:G | 0.42385 | 3.45366 |
| 2031011 | Vlasi-2 | H04 | A:A | 3.45064 | 0.46996 |
| 2031011 | Vlasi-2 | A05 | G:G | 0.48365 | 3.46312 |
| 2031011 | Vlasi-2 | B05 | A:A | 3.47414 | 0.46258 |
| 2031011 | Vlasi-2 | C05 | G:G | 0.41133 | 3.41607 |
| 2031011 | Vlasi-2 | D05 | G:G | 0.4086 | 3.58166 |
| 2031011 | Vlasi-2 | E05 | A:A | 3.18652 | 0.51987 |
| 2031011 | Vlasi-2 | F05 | G:A | 1.048 | 2.61913 |
| 2031011 | Vlasi-2 | G05 | G:A | 1.09738 | 2.58106 |
| 2031011 | Vlasi-2 | H05 | G:G | 0.42919 | 3.54327 |
| 2031011 | Vlasi-2 | A06 | A:A | 3.35133 | 0.5025 |
| 2031011 | Vlasi-2 | B06 | G:A | 1.17518 | 2.66054 |
| 2031011 | Vlasi-2 | C06 | G:G | 0.40357 | 3.55058 |
| 2031011 | Vlasi-2 | D06 | G:A | 1.15936 | 2.77047 |
| 2031011 | Vlasi-2 | E06 | G:A | 1.15354 | 2.63026 |
| 2031011 | Vlasi-2 | F06 | G:G | 0.39593 | 3.55708 |
| 2031011 | Vlasi-2 | G06 | G:A | 1.10919 | 2.59051 |
| 2031011 | Vlasi-2 | H06 | A:A | 3.50716 | 0.49101 |
| 2031011 | Vlasi-2 | A07 | G:G | 0.41438 | 3.21851 |
| 2031011 | Vlasi-2 | B07 | G:G | 0.41526 | 3.34083 |
| 2031011 | Vlasi-2 | C07 | G:G | 0.39578 | 3.46721 |
| 2031011 | Vlasi-2 | D07 | G:A | 1.09833 | 2.71547 |
| 2031011 | Vlasi-2 | E07 | G:G | 0.43437 | 3.49798 |
| 2031011 | Vlasi-2 | F07 | G:A | 1.02141 | 2.67582 |
| 2031011 | Vlasi-2 | G07 | G:A | 1.00912 | 2.62521 |
| 2031011 | Vlasi-2 | H07 | A:A | 3.78434 | 0.54416 |
| 2031011 | Vlasi-2 | A08 | G:A | 1.34361 | 2.72042 |
| 2031011 | Vlasi-2 | B08 | G:G | 0.40339 | 3.39221 |
| 2031011 | Vlasi-2 | C08 | G:G | 0.44447 | 3.47689 |
| 2031011 | Vlasi-2 | D08 | G:A | 1.13083 | 2.76418 |
| 2031011 | Vlasi-2 | E08 | A:A | 3.47767 | 0.54518 |
| 2031011 | Vlasi-2 | F08 | G:G | 0.40959 | 3.48275 |
| 2031011 | Vlasi-2 | G08 | G:A | 1.18606 | 2.69111 |
| 2031011 | Vlasi-2 | H08 | G:A | 1.08988 | 2.68715 |
| 2031011 | Vlasi-2 | A09 | G:A | 1.34992 | 2.59114 |
| 2031011 | Vlasi-2 | B09 | A:A | 3.42704 | 0.44693 |
| 2031011 | Vlasi-2 | C09 | G:A | 1.00051 | 2.65851 |
| 2031011 | Vlasi-2 | D09 | G:A | 1.15841 | 2.6444 |
| 2031011 | Vlasi-2 | E09 | G:G | 0.39096 | 3.33828 |
| 2031011 | Vlasi-2 | F09 | G:G | 0.39212 | 3.1961 |
| 2031011 | Vlasi-2 | G09 | G:G | 0.39242 | 3.56733 |
| 2031011 | Vlasi-2 | H09 | G:G | 0.42701 | 3.49151 |
| 2031011 | Vlasi-2 | A10 | G:G | 0.41773 | 3.25207 |
| 2031011 | Vlasi-2 | B10 | G:G | 0.39928 | 3.43358 |
| 2031011 | Vlasi-2 | C10 | G:G | 0.38427 | 3.3393 |
| 2031011 | Vlasi-2 | D10 | G:G | 0.41252 | 3.5765 |
| 2031011 | Vlasi-2 | E10 | G:A | 1.12848 | 2.6785 |

| | | | | | |
|---|---|---|---|---|---|
| 2031011 | Vlasi-2 | F10 | G:G | 0.41139 | 3.44076 |
| 2031011 | Vlasi-2 | G10 | G:G | 0.40914 | 3.62422 |
| 2031011 | Vlasi-2 | H10 | G:G | 0.43064 | 3.5722 |
| 2031011 | Vlasi-2 | A11 | G:A | 1.38663 | 2.7114 |
| 2031011 | Vlasi-2 | B11 | A:A | 3.47367 | 0.44015 |
| 2031011 | Vlasi-2 | C11 | G:G | 0.40881 | 3.46582 |
| 2031011 | Vlasi-2 | D11 | G:A | 1.17517 | 2.71048 |
| 2031011 | Vlasi-2 | E11 | G:G | 0.37194 | 3.41892 |
| 2031011 | Vlasi-2 | F11 | G:A | 1.11792 | 2.86778 |
| 2031011 | Vlasi-2 | G11 | G:G | 0.42303 | 3.56222 |
| 2031011 | Vlasi-2 | H11 | G:G | 0.41203 | 3.56674 |
| 2031011 | Vlasi-2 | A12 | G:G | 0.46129 | 3.27154 |
| 2031011 | Vlasi-2 | B12 | G:A | 1.43534 | 2.73454 |
| 2031011 | Vlasi-2 | C12 | G:G | 0.42958 | 3.59153 |
| 2031011 | Vlasi-2 | D12 | G:A | 1.36442 | 2.70109 |
| 2031011 | Vlasi-2 | E12 | G:A | 1.42599 | 2.77054 |
| 2031011 | Vlasi-2 | F12 | G:G | 0.43246 | 3.48874 |
| 2031011 | Vlasi-2 | G12 | G:A | 1.41725 | 2.73615 |
| 2031011 | Vlasi-2 | H12 | G:A | 1.36849 | 2.83826 |
| 2031011 | Vlasi-3 | A01 | G:G | 0.37775 | 3.37232 |
| 2031011 | Vlasi-3 | B01 | G:G | 0.367 | 3.6175 |
| 2031011 | Vlasi-3 | C01 | G:G | 0.34118 | 3.55053 |
| 2031011 | Vlasi-3 | D01 | G:G | 0.32757 | 3.55669 |
| 2031011 | Vlasi-3 | E01 | G:G | 0.3178 | 3.46994 |
| 2031011 | Vlasi-3 | F01 | G:A | 1.18561 | 2.66105 |
| 2031011 | Vlasi-3 | G01 | A:A | 3.69215 | 0.41459 |
| 2031011 | Vlasi-3 | H01 | G:G | 0.37952 | 3.43785 |
| 2031011 | Vlasi-3 | A02 | G:A | 1.38628 | 2.57794 |
| 2031011 | Vlasi-3 | B02 | G:G | 0.44013 | 3.57192 |
| 2031011 | Vlasi-3 | C02 | G:G | 0.39388 | 3.5365 |
| 2031011 | Vlasi-3 | D02 | A:A | 3.6969 | 0.49261 |
| 2031011 | Vlasi-3 | E02 | G:A | 1.0865 | 2.73866 |
| 2031011 | Vlasi-3 | F02 | G:A | 0.99715 | 2.62653 |
| 2031011 | Vlasi-3 | G02 | G:A | 0.97016 | 2.65526 |
| 2031011 | Vlasi-3 | H02 | G:A | 1.2559 | 2.71693 |
| 2031011 | Vlasi-3 | A03 | G:A | 1.31277 | 2.61879 |
| 2031011 | Vlasi-3 | B03 | G:A | 1.22738 | 2.65246 |
| 2031011 | Vlasi-3 | C03 | G:A | 1.23258 | 2.70849 |
| 2031011 | Vlasi-3 | D03 | A:A | 3.47554 | 0.54892 |
| 2031011 | Vlasi-3 | E03 | G:A | 1.04774 | 2.69034 |
| 2031011 | Vlasi-3 | F03 | G:G | 0.40786 | 3.32137 |
| 2031011 | Vlasi-3 | G03 | G:A | 1.22646 | 2.70447 |
| 2031011 | Vlasi-3 | H03 | G:A | 1.23962 | 2.59678 |
| 2031011 | Vlasi-3 | A04 | G:G | 0.42345 | 3.4434 |
| 2031011 | Vlasi-3 | B04 | A:A | 3.35946 | 0.43367 |
| 2031011 | Vlasi-3 | C04 | G:G | 0.38409 | 3.44236 |
| 2031011 | Vlasi-3 | D04 | G:G | 0.41901 | 3.63774 |
| 2031011 | Vlasi-3 | E04 | A:A | 3.36661 | 0.47826 |
| 2031011 | Vlasi-3 | F04 | G:A | 1.07917 | 2.61474 |
| 2031011 | Vlasi-3 | G04 | G:A | 1.0851 | 2.74705 |

| | | | | | |
|---|---|---|---|---|---|
| 2031011 | Vlasi-3 | H04 | G:G | 0.40587 | 3.51546 |
| 2031011 | Vlasi-3 | A05 | G:A | 1.40621 | 2.73203 |
| 2031011 | Vlasi-3 | B05 | G:A | 1.1783 | 2.77955 |
| 2031011 | Vlasi-3 | C05 | G:A | 1.18949 | 2.80393 |
| 2031011 | Vlasi-3 | D05 | G:G | 0.40447 | 3.61918 |
| 2031011 | Vlasi-3 | E05 | G:A | 1.13872 | 2.76758 |
| 2031011 | Vlasi-3 | F05 | G:G | 0.39567 | 3.45601 |
| 2031011 | Vlasi-3 | G05 | A:A | 3.32305 | 0.446 |
| 2031011 | Vlasi-3 | H05 | G:G | 0.44061 | 3.71535 |
| 2031011 | Vlasi-3 | A06 | G:G | 0.40168 | 3.26682 |
| 2031011 | Vlasi-3 | B06 | ? | 2.22314 | 0.45647 |
| 2031011 | Vlasi-3 | C06 | G:A | 1.07375 | 2.60129 |
| 2031011 | Vlasi-3 | D06 | G:A | 1.03631 | 2.79126 |
| 2031011 | Vlasi-3 | E06 | G:A | 1.14352 | 2.70325 |
| 2031011 | Vlasi-3 | F06 | G:G | 0.41234 | 3.49657 |
| 2031011 | Vlasi-3 | G06 | G:G | 0.41185 | 3.36849 |
| 2031011 | Vlasi-3 | H06 | A:A | 3.69818 | 0.46388 |
| 2031011 | Vlasi-3 | A07 | A:A | 3.45545 | 0.44162 |
| 2031011 | Vlasi-3 | B07 | G:A | 1.06406 | 2.71747 |
| 2031011 | Vlasi-3 | C07 | G:A | 1.17159 | 2.6382 |
| 2031011 | Vlasi-3 | D07 | G:G | 0.38362 | 3.56584 |
| 2031011 | Vlasi-3 | E07 | G:A | 1.11938 | 2.66808 |
| 2031011 | Vlasi-3 | F07 | G:G | 0.41957 | 3.41082 |
| 2031011 | Vlasi-3 | G07 | G:G | 0.42611 | 3.51327 |
| 2031011 | Vlasi-3 | H07 | G:G | 0.4258 | 3.53833 |
| 2031011 | Vlasi-3 | A08 | G:G | 0.39585 | 3.40948 |
| 2031011 | Vlasi-3 | B08 | G:A | 1.15939 | 2.80696 |
| 2031011 | Vlasi-3 | C08 | G:G | 0.382 | 3.47232 |
| 2031011 | Vlasi-3 | D08 | G:A | 1.22236 | 2.79213 |
| 2031011 | Vlasi-3 | E08 | G:A | 1.105 | 2.65993 |
| 2031011 | Vlasi-3 | F08 | G:A | 1.12478 | 2.64765 |
| 2031011 | Vlasi-3 | G08 | G:A | 0.99596 | 2.69713 |
| 2031011 | Vlasi-3 | H08 | G:A | 1.23487 | 2.66903 |
| 2031011 | Vlasi-3 | A09 | G:A | 1.28139 | 2.71929 |
| 2031011 | Vlasi-3 | B09 | G:G | 0.39928 | 3.46122 |
| 2031011 | Vlasi-3 | C09 | G:A | 1.11131 | 2.65959 |
| 2031011 | Vlasi-3 | D09 | G:A | 1.00485 | 2.6602 |
| 2031011 | Vlasi-3 | E09 | G:G | 0.41 | 3.56034 |
| 2031011 | Vlasi-3 | F09 | G:A | 1.07464 | 2.62242 |
| 2031011 | Vlasi-3 | G09 | G:G | 0.4075 | 3.64572 |
| 2031011 | Vlasi-3 | H09 | G:A | 1.26649 | 2.75183 |
| 2031011 | Vlasi-3 | A10 | A:A | 3.2541 | 0.44814 |
| 2031011 | Vlasi-3 | B10 | G:A | 1.09431 | 2.62774 |
| 2031011 | Vlasi-3 | C10 | G:G | 0.37563 | 3.53532 |
| 2031011 | Vlasi-3 | D10 | G:G | 0.38012 | 3.41764 |
| 2031011 | Vlasi-3 | E10 | G:G | 0.37558 | 3.50562 |
| 2031011 | Vlasi-3 | F10 | G:A | 1.03028 | 2.7523 |
| 2031011 | Vlasi-3 | G10 | G:G | 0.4065 | 3.5124 |
| 2031011 | Vlasi-3 | H10 | G:A | 1.2551 | 2.66155 |
| 2031011 | Vlasi-3 | A11 | G:G | 0.40476 | 3.33291 |

| | | | | | |
|---|---|---|---|---|---|
| 2031011 | Vlasi-3 | B11 | G:G | 0.37235 | 3.43797 |
| 2031011 | Vlasi-3 | C11 | G:G | 0.39882 | 3.64165 |
| 2031011 | Vlasi-3 | D11 | G:A | 1.19933 | 2.70288 |
| 2031011 | Vlasi-3 | E11 | G:A | 1.10739 | 2.73977 |
| 2031011 | Vlasi-3 | F11 | G:A | 1.12003 | 2.65053 |
| 2031011 | Vlasi-3 | G11 | G:G | 0.39952 | 3.56604 |
| 2031011 | Vlasi-3 | H11 | A:A | 3.82157 | 0.46858 |
| 2031011 | Vlasi-3 | A12 | A:A | 3.34074 | 0.45573 |
| 2031011 | Vlasi-3 | B12 | G:G | 0.40707 | 3.47965 |
| 2031011 | Vlasi-3 | C12 | G:G | 0.38563 | 3.63989 |
| 2031011 | Vlasi-3 | D12 | G:A | 1.23473 | 2.85338 |
| 2031011 | Vlasi-3 | E12 | G:A | 1.25221 | 2.71773 |
| 2031011 | Vlasi-3 | F12 | G:G | 0.39704 | 3.43986 |
| 2031011 | Vlasi-3 | G12 | G:G | 0.45361 | 3.60066 |
| 2031011 | Vlasi-3 | H12 | G:A | 1.28621 | 2.86049 |
| 2031011 | Vlasi-4 | A01 | G:A | 1.23757 | 2.50778 |
| 2031011 | Vlasi-4 | B01 | G:G | 0.39095 | 3.32141 |
| 2031011 | Vlasi-4 | C01 | G:G | 0.38514 | 3.51602 |
| 2031011 | Vlasi-4 | D01 | G:A | 0.99639 | 2.61281 |
| 2031011 | Vlasi-4 | E01 | G:A | 0.93795 | 2.7734 |
| 2031011 | Vlasi-4 | F01 | G:A | 1.10258 | 2.48734 |
| 2031011 | Vlasi-4 | G01 | G:G | 0.40869 | 3.49381 |
| 2031011 | Vlasi-4 | H01 | G:A | 1.24503 | 2.75402 |
| 2031011 | Vlasi-4 | A02 | G:A | 1.40536 | 2.72544 |
| 2031011 | Vlasi-4 | B02 | G:G | 0.39767 | 3.48754 |
| 2031011 | Vlasi-4 | C02 | G:A | 1.20279 | 2.77877 |
| 2031011 | Vlasi-4 | D02 | G:A | 1.19045 | 2.7464 |
| 2031011 | Vlasi-4 | E02 | G:A | 1.11126 | 2.66878 |
| 2031011 | Vlasi-4 | F02 | G:A | 1.08099 | 2.52731 |
| 2031011 | Vlasi-4 | G02 | G:A | 0.946 | 2.61126 |
| 2031011 | Vlasi-4 | H02 | G:A | 1.21495 | 2.80192 |
| 2031011 | Vlasi-4 | A03 | A:A | 3.40731 | 0.488 |
| 2031011 | Vlasi-4 | B03 | G:A | 1.27367 | 2.70581 |
| 2031011 | Vlasi-4 | C03 | G:A | 1.14537 | 2.58874 |
| 2031011 | Vlasi-4 | D03 | G:A | 1.15898 | 2.63416 |
| 2031011 | Vlasi-4 | E03 | G:G | 0.42113 | 3.68867 |
| 2031011 | Vlasi-4 | F03 | G:A | 1.1012 | 2.76669 |
| 2031011 | Vlasi-4 | G03 | G:G | 0.39776 | 3.58654 |
| 2031011 | Vlasi-4 | H03 | G:G | 0.41298 | 3.49353 |
| 2031011 | Vlasi-4 | A04 | G:A | 1.23601 | 2.83061 |
| 2031011 | Vlasi-4 | B04 | G:A | 1.05353 | 2.73094 |
| 2031011 | Vlasi-4 | C04 | G:A | 1.05769 | 2.78087 |
| 2031011 | Vlasi-4 | D04 | G:G | 0.40669 | 3.58041 |
| 2031011 | Vlasi-4 | E04 | G:A | 1.03186 | 2.67039 |
| 2031011 | Vlasi-4 | F04 | G:G | 0.40569 | 3.59931 |
| 2031011 | Vlasi-4 | G04 | G:A | 1.08981 | 2.60989 |
| 2031011 | Vlasi-4 | H04 | G:G | 0.44471 | 3.5432 |
| 2031011 | Vlasi-4 | A05 | G:G | 0.42098 | 3.28668 |
| 2031011 | Vlasi-4 | B05 | G:G | 0.40239 | 3.43787 |
| 2031011 | Vlasi-4 | C05 | G:G | 0.36841 | 3.39524 |

| | | | | | |
|---|---|---|---|---|---|
| 2031011 | Vlasi-4 | D05 | G:A | 1.03786 | 2.58742 |
| 2031011 | Vlasi-4 | E05 | G:A | 1.01379 | 2.56911 |
| 2031011 | Vlasi-4 | F05 | G:G | 0.40011 | 3.33758 |
| 2031011 | Vlasi-4 | G05 | A:A | 3.19325 | 0.46399 |
| 2031011 | Vlasi-4 | H05 | G:A | 1.26431 | 2.67505 |
| 2031011 | Vlasi-4 | A06 | G:A | 1.1017 | 2.46114 |
| 2031011 | Vlasi-4 | B06 | G:G | 0.38002 | 3.27789 |
| 2031011 | Vlasi-4 | C06 | G:A | 1.16589 | 2.73401 |
| 2031011 | Vlasi-4 | D06 | G:A | 0.92585 | 2.55769 |
| 2031011 | Vlasi-4 | E06 | G:A | 1.12577 | 2.74507 |
| 2031011 | Vlasi-4 | F06 | G:G | 0.40197 | 3.27644 |
| 2031011 | Vlasi-4 | G06 | G:G | 0.40522 | 3.40188 |
| 2031011 | Vlasi-4 | H06 | G:A | 1.1272 | 2.78875 |
| 2031011 | Vlasi-4 | A07 | ? | 0.53992 | 0.48676 |
| 2031011 | Vlasi-4 | B07 | ? | 0.5706 | 0.47527 |
| 2031011 | Vlasi-4 | C07 | ? | 0.58518 | 0.50007 |
| 2031011 | Vlasi-4 | D07 | ? | 0.60605 | 0.52351 |
| 2031011 | Vlasi-4 | E07 | ? | 0.56666 | 0.51062 |
| 2031011 | Vlasi-4 | F07 | A:A | 3.57266 | 0.61478 |
| 2031011 | Vlasi-4 | G07 | A:A | 3.74351 | 0.5384 |
| 2031011 | Vlasi-4 | H07 | A:A | 3.68647 | 0.60137 |
| 2031011 | Vlasi-4 | A08 | A:A | 3.51542 | 0.63124 |
| 2031011 | Vlasi-4 | B08 | A:A | 3.45925 | 0.56597 |
| 2031011 | Vlasi-4 | C08 | A:A | 3.44795 | 0.73258 |
| 2031011 | Vlasi-4 | D08 | A:A | 3.66733 | 0.61174 |
| 2031011 | Vlasi-4 | E08 | A:A | 3.6732 | 0.64035 |
| 2031011 | Vlasi-4 | F08 | A:A | 3.58543 | 0.58662 |
| 2031011 | Vlasi-4 | G08 | A:A | 3.66389 | 0.57303 |
| 2031011 | Vlasi-4 | H08 | A:A | 3.76282 | 0.68849 |
| 2031011 | Vlasi-4 | A09 | A:A | 3.43209 | 0.69536 |
| 2031011 | Vlasi-4 | B09 | A:A | 3.4244 | 0.58859 |
| 2031011 | Vlasi-4 | C09 | A:A | 3.45489 | 0.65896 |
| 2031011 | Vlasi-4 | D09 | A:A | 3.50332 | 0.62296 |
| 2031011 | Vlasi-4 | E09 | A:A | 3.61232 | 0.61903 |
| 2031011 | Vlasi-4 | F09 | A:A | 3.46445 | 0.64497 |
| 2031011 | Vlasi-4 | G09 | A:A | 3.46049 | 0.6277 |
| 2031011 | Vlasi-4 | H09 | A:A | 3.69293 | 0.63122 |
| 2031011 | Vlasi-4 | A10 | A:A | 3.56936 | 0.73144 |
| 2031011 | Vlasi-4 | B10 | A:A | 3.55177 | 0.58543 |
| 2031011 | Vlasi-4 | C10 | G:G | 0.36521 | 3.40845 |
| 2031011 | Vlasi-4 | D10 | G:G | 0.37244 | 3.52718 |
| 2031011 | Vlasi-4 | E10 | G:G | 0.4007 | 3.54783 |
| 2031011 | Vlasi-4 | F10 | G:G | 0.40277 | 3.56108 |
| 2031011 | Vlasi-4 | G10 | G:G | 0.39983 | 3.42864 |
| 2031011 | Vlasi-4 | H10 | G:G | 0.43722 | 3.58499 |
| 2031011 | Vlasi-4 | A11 | G:G | 0.42806 | 3.33286 |
| 2031011 | Vlasi-4 | B11 | G:G | 0.41172 | 3.53045 |
| 2031011 | Vlasi-4 | C11 | G:G | 0.38914 | 3.51468 |
| 2031011 | Vlasi-4 | D11 | G:G | 0.38795 | 3.49004 |
| 2031011 | Vlasi-4 | E11 | G:G | 0.41393 | 3.52456 |

| | | | | | |
|---|---|---|---|---|---|
| 2031011 | Vlasi-4 | F11 | G:G | 0.42198 | 3.49082 |
| 2031011 | Vlasi-4 | G11 | G:G | 0.41225 | 3.43892 |
| 2031011 | Vlasi-4 | H11 | G:G | 0.4207 | 3.53391 |
| 2031011 | Vlasi-4 | A12 | G:G | 0.52019 | 3.37705 |
| 2031011 | Vlasi-4 | B12 | G:G | 0.46443 | 3.47444 |
| 2031011 | Vlasi-4 | C12 | G:G | 0.44921 | 3.46461 |
| 2031011 | Vlasi-4 | D12 | G:G | 0.50654 | 3.5987 |
| 2031011 | Vlasi-4 | E12 | G:G | 0.49556 | 3.48922 |
| 2031011 | Vlasi-4 | F12 | G:G | 0.48452 | 3.48939 |
| 2031011 | Vlasi-4 | G12 | G:G | 0.48755 | 3.44937 |
| 2031011 | Vlasi-4 | H12 | NTC | 0.6149 | 0.54661 |
| 2031011 | Vlasi-1 | A01 | A:A | 0.51979 | 3.59894 |
| 2031011 | Vlasi-1 | B01 | A:C | 2.54909 | 2.23765 |
| 2031011 | Vlasi-1 | C01 | A:C | 2.4589 | 2.15591 |
| 2031011 | Vlasi-1 | D01 | A:A | 0.39336 | 3.75941 |
| 2031011 | Vlasi-1 | E01 | A:C | 2.5635 | 2.14249 |
| 2031011 | Vlasi-1 | F01 | A:C | 2.55064 | 2.14676 |
| 2031011 | Vlasi-1 | G01 | C:C | 3.8787 | 0.57053 |
| 2031011 | Vlasi-1 | H01 | A:A | 0.43902 | 3.75316 |
| 2031011 | Vlasi-1 | A02 | A:C | 2.33041 | 1.93224 |
| 2031011 | Vlasi-1 | B02 | A:C | 2.44497 | 2.2299 |
| 2031011 | Vlasi-1 | C02 | A:C | 2.33262 | 2.07271 |
| 2031011 | Vlasi-1 | D02 | A:C | 2.2337 | 2.05085 |
| 2031011 | Vlasi-1 | E02 | A:C | 2.26451 | 1.98039 |
| 2031011 | Vlasi-1 | F02 | A:C | 2.32735 | 2.08474 |
| 2031011 | Vlasi-1 | G02 | A:A | 0.43973 | 3.59542 |
| 2031011 | Vlasi-1 | H02 | C:C | 3.70823 | 0.62751 |
| 2031011 | Vlasi-1 | A03 | A:C | 2.45047 | 1.95495 |
| 2031011 | Vlasi-1 | B03 | A:C | 2.3566 | 2.12278 |
| 2031011 | Vlasi-1 | C03 | A:A | 0.43785 | 3.5148 |
| 2031011 | Vlasi-1 | D03 | A:C | 2.28435 | 2.06189 |
| 2031011 | Vlasi-1 | E03 | A:A | 0.43636 | 3.73704 |
| 2031011 | Vlasi-1 | F03 | A:A | 0.41738 | 3.62488 |
| 2031011 | Vlasi-1 | G03 | A:A | 0.44106 | 3.52392 |
| 2031011 | Vlasi-1 | H03 | C:C | 3.70783 | 0.56594 |
| 2031011 | Vlasi-1 | A04 | A:C | 2.41989 | 2.01594 |
| 2031011 | Vlasi-1 | B04 | A:C | 2.34343 | 2.09186 |
| 2031011 | Vlasi-1 | C04 | A:C | 2.25681 | 2.08368 |
| 2031011 | Vlasi-1 | D04 | A:A | 0.43448 | 3.72012 |
| 2031011 | Vlasi-1 | E04 | A:A | 0.42371 | 3.66599 |
| 2031011 | Vlasi-1 | F04 | A:A | 0.44766 | 3.80248 |
| 2031011 | Vlasi-1 | G04 | C:C | 3.64503 | 0.54014 |
| 2031011 | Vlasi-1 | H04 | A:C | 2.33849 | 1.98633 |
| 2031011 | Vlasi-1 | A05 | A:A | 0.54466 | 3.58746 |
| 2031011 | Vlasi-1 | B05 | A:A | 0.41035 | 3.54798 |
| 2031011 | Vlasi-1 | C05 | C:C | 3.7426 | 0.53941 |
| 2031011 | Vlasi-1 | D05 | A:C | 2.24422 | 2.05412 |
| 2031011 | Vlasi-1 | E05 | A:A | 0.44431 | 3.6311 |
| 2031011 | Vlasi-1 | F05 | A:C | 2.10387 | 1.9736 |
| 2031011 | Vlasi-1 | G05 | A:A | 0.44657 | 3.56176 |

| | | | | | |
|---|---|---|---|---|---|
| 2031011 | Vlasi-1 | H05 | A:A | 0.47199 | 3.63618 |
| 2031011 | Vlasi-1 | A06 | A:A | 0.49023 | 3.39799 |
| 2031011 | Vlasi-1 | B06 | A:A | 0.43094 | 3.41474 |
| 2031011 | Vlasi-1 | C06 | A:C | 2.38721 | 2.06363 |
| 2031011 | Vlasi-1 | D06 | A:C | 2.23075 | 2.05709 |
| 2031011 | Vlasi-1 | E06 | A:A | 0.42782 | 3.57134 |
| 2031011 | Vlasi-1 | F06 | A:A | 0.47047 | 3.5101 |
| 2031011 | Vlasi-1 | G06 | A:C | 2.22053 | 1.95267 |
| 2031011 | Vlasi-1 | H06 | C:C | 3.68237 | 0.54489 |
| 2031011 | Vlasi-1 | A07 | A:A | 0.48384 | 3.46447 |
| 2031011 | Vlasi-1 | B07 | A:C | 2.2759 | 2.06587 |
| 2031011 | Vlasi-1 | C07 | C:C | 3.61381 | 0.54279 |
| 2031011 | Vlasi-1 | D07 | A:C | 2.26133 | 1.98994 |
| 2031011 | Vlasi-1 | E07 | A:A | 0.44111 | 3.62046 |
| 2031011 | Vlasi-1 | F07 | A:C | 2.15728 | 1.8879 |
| 2031011 | Vlasi-1 | G07 | C:C | 3.72465 | 0.54493 |
| 2031011 | Vlasi-1 | H07 | A:A | 0.4825 | 3.53261 |
| 2031011 | Vlasi-1 | A08 | A:A | 0.46171 | 3.42554 |
| 2031011 | Vlasi-1 | B08 | C:C | 3.63071 | 0.55351 |
| 2031011 | Vlasi-1 | C08 | A:C | 2.33674 | 2.11063 |
| 2031011 | Vlasi-1 | D08 | A:A | 0.43007 | 3.77755 |
| 2031011 | Vlasi-1 | E08 | A:A | 0.46282 | 3.68744 |
| 2031011 | Vlasi-1 | F08 | A:A | 0.42887 | 3.70081 |
| 2031011 | Vlasi-1 | G08 | C:C | 3.61915 | 0.56055 |
| 2031011 | Vlasi-1 | H08 | C:C | 3.85685 | 0.60195 |
| 2031011 | Vlasi-1 | A09 | A:C | 2.36436 | 2.18402 |
| 2031011 | Vlasi-1 | B09 | A:A | 0.4619 | 3.44198 |
| 2031011 | Vlasi-1 | C09 | A:A | 0.41427 | 3.53542 |
| 2031011 | Vlasi-1 | D09 | A:A | 0.4103 | 3.54552 |
| 2031011 | Vlasi-1 | E09 | A:A | 0.42184 | 3.61821 |
| 2031011 | Vlasi-1 | F09 | A:C | 2.25343 | 2.04544 |
| 2031011 | Vlasi-1 | G09 | A:C | 2.17132 | 2.03197 |
| 2031011 | Vlasi-1 | H09 | A:C | 2.31047 | 1.98281 |
| 2031011 | Vlasi-1 | A10 | A:C | 2.34161 | 2.08942 |
| 2031011 | Vlasi-1 | B10 | A:C | 2.21968 | 2.1396 |
| 2031011 | Vlasi-1 | C10 | A:A | 0.42896 | 3.61015 |
| 2031011 | Vlasi-1 | D10 | A:C | 2.16412 | 2.00639 |
| 2031011 | Vlasi-1 | E10 | A:A | 0.43514 | 3.67717 |
| 2031011 | Vlasi-1 | F10 | C:C | 3.77306 | 0.58943 |
| 2031011 | Vlasi-1 | G10 | A:A | 0.45268 | 3.66947 |
| 2031011 | Vlasi-1 | H10 | A:A | 0.62101 | 3.25736 |
| 2031011 | Vlasi-1 | A11 | C:C | 3.57532 | 0.71413 |
| 2031011 | Vlasi-1 | B11 | A:C | 2.33057 | 2.02923 |
| 2031011 | Vlasi-1 | C11 | A:C | 2.28525 | 2.02467 |
| 2031011 | Vlasi-1 | D11 | A:C | 2.36391 | 2.08451 |
| 2031011 | Vlasi-1 | E11 | A:A | 0.43048 | 3.71868 |
| 2031011 | Vlasi-1 | F11 | A:A | 0.44449 | 3.69878 |
| 2031011 | Vlasi-1 | G11 | A:A | 0.441 | 3.63906 |
| 2031011 | Vlasi-1 | H11 | A:C | 2.29336 | 1.88803 |
| 2031011 | Vlasi-1 | A12 | C:C | 3.51871 | 0.6426 |

| | | | | | |
|---|---|---|---|---|---|
| 2031011 | Vlasi-1 | B12 | A:C | 2.27094 | 2.01209 |
| 2031011 | Vlasi-1 | C12 | A:C | 2.30317 | 2.09278 |
| 2031011 | Vlasi-1 | D12 | A:C | 2.2506 | 2.03325 |
| 2031011 | Vlasi-1 | E12 | C:C | 3.82993 | 0.55813 |
| 2031011 | Vlasi-1 | F12 | A:C | 2.23664 | 2.04328 |
| 2031011 | Vlasi-1 | G12 | A:C | 2.3444 | 1.92467 |
| 2031011 | Vlasi-1 | H12 | A:A | 0.46505 | 3.51934 |
| 2031011 | Vlasi-2 | A01 | A:C | 2.35639 | 2.14598 |
| 2031011 | Vlasi-2 | B01 | A:A | 0.42655 | 3.46261 |
| 2031011 | Vlasi-2 | C01 | A:A | 0.39666 | 3.5373 |
| 2031011 | Vlasi-2 | D01 | A:C | 2.29037 | 2.04041 |
| 2031011 | Vlasi-2 | E01 | A:A | 0.42344 | 3.75615 |
| 2031011 | Vlasi-2 | F01 | A:C | 2.22928 | 2.02172 |
| 2031011 | Vlasi-2 | G01 | A:C | 2.36939 | 1.93848 |
| 2031011 | Vlasi-2 | H01 | A:C | 2.40776 | 1.99448 |
| 2031011 | Vlasi-2 | A02 | A:C | 2.38634 | 2.17835 |
| 2031011 | Vlasi-2 | B02 | A:A | 0.46866 | 3.6346 |
| 2031011 | Vlasi-2 | C02 | A:A | 0.43765 | 3.59632 |
| 2031011 | Vlasi-2 | D02 | A:C | 2.27114 | 2.09737 |
| 2031011 | Vlasi-2 | E02 | A:A | 0.42015 | 3.46779 |
| 2031011 | Vlasi-2 | F02 | A:A | 0.42972 | 3.59338 |
| 2031011 | Vlasi-2 | G02 | A:C | 2.28797 | 2.03615 |
| 2031011 | Vlasi-2 | H02 | A:A | 0.46345 | 3.5362 |
| 2031011 | Vlasi-2 | A03 | A:A | 0.59534 | 3.47097 |
| 2031011 | Vlasi-2 | B03 | A:C | 2.32287 | 2.01989 |
| 2031011 | Vlasi-2 | C03 | A:A | 0.45884 | 3.6111 |
| 2031011 | Vlasi-2 | D03 | A:C | 2.24598 | 2.1159 |
| 2031011 | Vlasi-2 | E03 | A:A | 0.44733 | 3.86824 |
| 2031011 | Vlasi-2 | F03 | A:A | 0.42051 | 3.51394 |
| 2031011 | Vlasi-2 | G03 | C:C | 3.47054 | 0.61031 |
| 2031011 | Vlasi-2 | H03 | A:A | 0.44939 | 3.54043 |
| 2031011 | Vlasi-2 | A04 | A:A | 0.52917 | 3.44259 |
| 2031011 | Vlasi-2 | B04 | A:A | 0.4165 | 3.39217 |
| 2031011 | Vlasi-2 | C04 | A:C | 2.27809 | 2.0234 |
| 2031011 | Vlasi-2 | D04 | A:A | 0.42987 | 3.5869 |
| 2031011 | Vlasi-2 | E04 | C:C | 3.70078 | 0.52276 |
| 2031011 | Vlasi-2 | F04 | A:A | 0.47308 | 3.62792 |
| 2031011 | Vlasi-2 | G04 | A:C | 2.12399 | 1.93886 |
| 2031011 | Vlasi-2 | H04 | A:A | 0.44909 | 3.58068 |
| 2031011 | Vlasi-2 | A05 | A:A | 0.59652 | 3.3556 |
| 2031011 | Vlasi-2 | B05 | A:A | 0.42274 | 3.47993 |
| 2031011 | Vlasi-2 | C05 | A:C | 2.29943 | 2.11533 |
| 2031011 | Vlasi-2 | D05 | A:C | 2.27913 | 2.00552 |
| 2031011 | Vlasi-2 | E05 | A:A | 0.43136 | 3.61876 |
| 2031011 | Vlasi-2 | F05 | A:A | 0.44603 | 3.62017 |
| 2031011 | Vlasi-2 | G05 | A:C | 2.1277 | 1.92158 |
| 2031011 | Vlasi-2 | H05 | A:A | 0.46797 | 3.57203 |
| 2031011 | Vlasi-2 | A06 | A:C | 2.36606 | 1.95939 |
| 2031011 | Vlasi-2 | B06 | A:A | 0.43479 | 3.48875 |
| 2031011 | Vlasi-2 | C06 | A:A | 0.43598 | 3.55958 |

| | | | | | |
|---|---|---|---|---|---|
| 2031011 | Vlasi-2 | D06 | A:C | 2.32277 | 2.12939 |
| 2031011 | Vlasi-2 | E06 | A:C | 2.18774 | 2.01526 |
| 2031011 | Vlasi-2 | F06 | A:A | 0.41568 | 3.51773 |
| 2031011 | Vlasi-2 | G06 | A:C | 2.1957 | 1.94251 |
| 2031011 | Vlasi-2 | H06 | A:A | 0.53429 | 3.55782 |
| 2031011 | Vlasi-2 | A07 | A:A | 0.47073 | 3.34737 |
| 2031011 | Vlasi-2 | B07 | A:A | 0.48732 | 3.61559 |
| 2031011 | Vlasi-2 | C07 | A:C | 2.24127 | 2.06225 |
| 2031011 | Vlasi-2 | D07 | A:A | 0.4279 | 3.70216 |
| 2031011 | Vlasi-2 | E07 | C:C | 3.65058 | 0.51867 |
| 2031011 | Vlasi-2 | F07 | A:C | 2.24898 | 1.95429 |
| 2031011 | Vlasi-2 | G07 | A:C | 2.22892 | 1.8966 |
| 2031011 | Vlasi-2 | H07 | A:C | 2.23932 | 1.95407 |
| 2031011 | Vlasi-2 | A08 | A:A | 0.52171 | 3.45043 |
| 2031011 | Vlasi-2 | B08 | A:C | 2.26682 | 2.0933 |
| 2031011 | Vlasi-2 | C08 | A:A | 0.4855 | 3.63825 |
| 2031011 | Vlasi-2 | D08 | A:A | 0.44474 | 3.69113 |
| 2031011 | Vlasi-2 | E08 | A:A | 0.44873 | 3.69184 |
| 2031011 | Vlasi-2 | F08 | C:C | 3.77799 | 0.55649 |
| 2031011 | Vlasi-2 | G08 | A:A | 0.45821 | 3.61449 |
| 2031011 | Vlasi-2 | H08 | A:C | 2.39009 | 2.08378 |
| 2031011 | Vlasi-2 | A09 | A:A | 0.46665 | 3.26105 |
| 2031011 | Vlasi-2 | B09 | C:C | 3.6124 | 0.59852 |
| 2031011 | Vlasi-2 | C09 | A:A | 0.43203 | 3.69643 |
| 2031011 | Vlasi-2 | D09 | A:A | 0.41924 | 3.75374 |
| 2031011 | Vlasi-2 | E09 | A:A | 0.43609 | 3.74797 |
| 2031011 | Vlasi-2 | F09 | A:C | 2.16855 | 1.78542 |
| 2031011 | Vlasi-2 | G09 | A:C | 2.1687 | 1.8919 |
| 2031011 | Vlasi-2 | H09 | A:C | 2.31836 | 2.06627 |
| 2031011 | Vlasi-2 | A10 | A:A | 0.53341 | 3.40182 |
| 2031011 | Vlasi-2 | B10 | A:C | 2.26145 | 2.03499 |
| 2031011 | Vlasi-2 | C10 | A:A | 0.44808 | 3.6457 |
| 2031011 | Vlasi-2 | D10 | C:C | 3.64604 | 0.52617 |
| 2031011 | Vlasi-2 | E10 | A:C | 2.16508 | 2.08928 |
| 2031011 | Vlasi-2 | F10 | A:A | 0.46197 | 3.81575 |
| 2031011 | Vlasi-2 | G10 | A:A | 0.45985 | 3.69937 |
| 2031011 | Vlasi-2 | H10 | A:A | 0.50786 | 3.71959 |
| 2031011 | Vlasi-2 | A11 | C:C | 3.42988 | 0.63973 |
| 2031011 | Vlasi-2 | B11 | A:A | 0.48865 | 3.48171 |
| 2031011 | Vlasi-2 | C11 | A:C | 2.35119 | 2.14861 |
| 2031011 | Vlasi-2 | D11 | A:A | 0.43019 | 3.67696 |
| 2031011 | Vlasi-2 | E11 | A:A | 0.4354 | 3.77864 |
| 2031011 | Vlasi-2 | F11 | A:A | 0.46141 | 3.71581 |
| 2031011 | Vlasi-2 | G11 | A:C | 2.3543 | 2.05549 |
| 2031011 | Vlasi-2 | H11 | A:A | 0.48946 | 3.68478 |
| 2031011 | Vlasi-2 | A12 | A:A | 0.51849 | 3.49589 |
| 2031011 | Vlasi-2 | B12 | A:A | 0.49699 | 3.68189 |
| 2031011 | Vlasi-2 | C12 | A:C | 2.45675 | 2.13015 |
| 2031011 | Vlasi-2 | D12 | A:A | 0.45441 | 3.76103 |
| 2031011 | Vlasi-2 | E12 | A:A | 0.46465 | 3.62497 |

| | | | | | |
|---|---|---|---|---|---|
| 2031011 | Vlasi-2 | F12 | A:A | 0.52638 | 3.70563 |
| 2031011 | Vlasi-2 | G12 | A:C | 2.29087 | 2.10603 |
| 2031011 | Vlasi-2 | H12 | C:C | 3.621 | 0.63228 |
| 2031011 | Vlasi-3 | A01 | A:C | 2.62794 | 2.10332 |
| 2031011 | Vlasi-3 | B01 | A:C | 2.66603 | 2.12457 |
| 2031011 | Vlasi-3 | C01 | C:C | 3.99147 | 0.59975 |
| 2031011 | Vlasi-3 | D01 | A:A | 0.40499 | 3.69372 |
| 2031011 | Vlasi-3 | E01 | A:C | 2.42014 | 2.26867 |
| 2031011 | Vlasi-3 | F01 | A:C | 2.64604 | 2.15528 |
| 2031011 | Vlasi-3 | G01 | A:A | 0.40156 | 3.62008 |
| 2031011 | Vlasi-3 | H01 | C:C | 3.89255 | 0.72006 |
| 2031011 | Vlasi-3 | A02 | A:A | 0.48468 | 3.43036 |
| 2031011 | Vlasi-3 | B02 | C:C | 3.67399 | 0.56527 |
| 2031011 | Vlasi-3 | C02 | A:C | 2.24913 | 2.18185 |
| 2031011 | Vlasi-3 | D02 | A:A | 0.42765 | 3.50755 |
| 2031011 | Vlasi-3 | E02 | A:A | 0.4268 | 3.59767 |
| 2031011 | Vlasi-3 | F02 | A:A | 0.44481 | 3.53042 |
| 2031011 | Vlasi-3 | G02 | A:C | 2.30469 | 2.06923 |
| 2031011 | Vlasi-3 | H02 | A:A | 0.62011 | 3.4521 |
| 2031011 | Vlasi-3 | A03 | A:A | 0.45807 | 3.37002 |
| 2031011 | Vlasi-3 | B03 | A:A | 0.41302 | 3.45289 |
| 2031011 | Vlasi-3 | C03 | C:C | 3.65373 | 0.62643 |
| 2031011 | Vlasi-3 | D03 | A:A | 0.40933 | 3.5938 |
| 2031011 | Vlasi-3 | E03 | A:A | 0.42976 | 3.60975 |
| 2031011 | Vlasi-3 | F03 | A:A | 0.46749 | 3.81481 |
| 2031011 | Vlasi-3 | G03 | A:A | 0.45225 | 3.69608 |
| 2031011 | Vlasi-3 | H03 | A:A | 0.54825 | 3.60016 |
| 2031011 | Vlasi-3 | A04 | A:C | 2.13942 | 2.08053 |
| 2031011 | Vlasi-3 | B04 | A:A | 0.44969 | 3.68723 |
| 2031011 | Vlasi-3 | C04 | A:C | 2.42331 | 2.18089 |
| 2031011 | Vlasi-3 | D04 | A:C | 2.24611 | 2.13298 |
| 2031011 | Vlasi-3 | E04 | A:C | 2.14728 | 1.96823 |
| 2031011 | Vlasi-3 | F04 | A:C | 2.25237 | 1.93068 |
| 2031011 | Vlasi-3 | G04 | A:C | 2.24893 | 1.88618 |
| 2031011 | Vlasi-3 | H04 | A:A | 0.53998 | 3.4723 |
| 2031011 | Vlasi-3 | A05 | A:A | 0.48333 | 3.61285 |
| 2031011 | Vlasi-3 | B05 | A:A | 0.44877 | 3.72686 |
| 2031011 | Vlasi-3 | C05 | A:A | 0.42718 | 3.63342 |
| 2031011 | Vlasi-3 | D05 | A:A | 0.47723 | 3.62012 |
| 2031011 | Vlasi-3 | E05 | A:C | 2.31722 | 1.96946 |
| 2031011 | Vlasi-3 | F05 | A:A | 0.43735 | 3.70162 |
| 2031011 | Vlasi-3 | G05 | A:C | 2.25053 | 2.06777 |
| 2031011 | Vlasi-3 | H05 | A:A | 0.55965 | 3.5489 |
| 2031011 | Vlasi-3 | A06 | A:A | 0.45288 | 3.50906 |
| 2031011 | Vlasi-3 | B06 | A:A | 0.39861 | 3.39661 |
| 2031011 | Vlasi-3 | C06 | A:C | 2.22942 | 2.07809 |
| 2031011 | Vlasi-3 | D06 | A:C | 2.22731 | 2.00776 |
| 2031011 | Vlasi-3 | E06 | C:C | 3.69234 | 0.52539 |
| 2031011 | Vlasi-3 | F06 | A:A | 0.4206 | 3.45375 |
| 2031011 | Vlasi-3 | G06 | C:C | 3.77209 | 0.56695 |

| | | | | | |
|---|---|---|---|---|---|
| 2031011 | Vlasi-3 | H06 | A:C | 2.32113 | 2.11475 |
| 2031011 | Vlasi-3 | A07 | A:A | 0.42986 | 3.50313 |
| 2031011 | Vlasi-3 | B07 | A:A | 0.51833 | 3.16225 |
| 2031011 | Vlasi-3 | C07 | A:A | 0.4345 | 3.5379 |
| 2031011 | Vlasi-3 | D07 | A:A | 0.423 | 3.65024 |
| 2031011 | Vlasi-3 | E07 | A:C | 2.21471 | 2.02027 |
| 2031011 | Vlasi-3 | F07 | A:C | 2.25391 | 1.93759 |
| 2031011 | Vlasi-3 | G07 | A:A | 0.44486 | 3.55065 |
| 2031011 | Vlasi-3 | H07 | A:C | 2.4639 | 2.0515 |
| 2031011 | Vlasi-3 | A08 | A:C | 2.28286 | 1.9929 |
| 2031011 | Vlasi-3 | B08 | A:C | 2.16797 | 2.05483 |
| 2031011 | Vlasi-3 | C08 | C:C | 3.60938 | 0.57488 |
| 2031011 | Vlasi-3 | D08 | A:A | 0.43388 | 3.58857 |
| 2031011 | Vlasi-3 | E08 | A:A | 0.41646 | 3.7487 |
| 2031011 | Vlasi-3 | F08 | A:C | 2.26894 | 2.00869 |
| 2031011 | Vlasi-3 | G08 | A:A | 0.45352 | 3.65592 |
| 2031011 | Vlasi-3 | H08 | A:C | 2.33918 | 1.84812 |
| 2031011 | Vlasi-3 | A09 | C:C | 3.65589 | 0.59611 |
| 2031011 | Vlasi-3 | B09 | A:C | 2.14032 | 2.14527 |
| 2031011 | Vlasi-3 | C09 | A:C | 2.30664 | 2.02798 |
| 2031011 | Vlasi-3 | D09 | C:C | 3.5106 | 0.53363 |
| 2031011 | Vlasi-3 | E09 | A:C | 2.36058 | 1.89233 |
| 2031011 | Vlasi-3 | F09 | A:C | 2.29914 | 1.95775 |
| 2031011 | Vlasi-3 | G09 | A:C | 2.17295 | 1.91339 |
| 2031011 | Vlasi-3 | H09 | A:A | 0.54933 | 3.55955 |
| 2031011 | Vlasi-3 | A10 | A:A | 0.45036 | 3.47647 |
| 2031011 | Vlasi-3 | B10 | A:C | 2.26084 | 2.03034 |
| 2031011 | Vlasi-3 | C10 | A:C | 2.31798 | 2.06127 |
| 2031011 | Vlasi-3 | D10 | A:A | 0.46868 | 3.67384 |
| 2031011 | Vlasi-3 | E10 | A:A | 0.42494 | 3.63981 |
| 2031011 | Vlasi-3 | F10 | A:C | 2.2527 | 1.93885 |
| 2031011 | Vlasi-3 | G10 | A:C | 2.33752 | 2.05379 |
| 2031011 | Vlasi-3 | H10 | A:A | 0.53271 | 3.51652 |
| 2031011 | Vlasi-3 | A11 | A:A | 0.44302 | 3.53738 |
| 2031011 | Vlasi-3 | B11 | A:A | 0.42063 | 3.56011 |
| 2031011 | Vlasi-3 | C11 | A:C | 2.13316 | 2.26763 |
| 2031011 | Vlasi-3 | D11 | A:C | 2.28382 | 2.07465 |
| 2031011 | Vlasi-3 | E11 | A:C | 2.23084 | 2.17289 |
| 2031011 | Vlasi-3 | F11 | A:A | 0.45302 | 3.6905 |
| 2031011 | Vlasi-3 | G11 | A:C | 2.39649 | 1.96619 |
| 2031011 | Vlasi-3 | H11 | A:C | 2.50063 | 2.24827 |
| 2031011 | Vlasi-3 | A12 | C:C | 3.55427 | 0.60995 |
| 2031011 | Vlasi-3 | B12 | C:C | 3.68005 | 0.58628 |
| 2031011 | Vlasi-3 | C12 | A:C | 2.138 | 2.08973 |
| 2031011 | Vlasi-3 | D12 | A:C | 2.37352 | 2.06457 |
| 2031011 | Vlasi-3 | E12 | A:A | 0.42274 | 3.47001 |
| 2031011 | Vlasi-3 | F12 | A:C | 2.3727 | 1.94432 |
| 2031011 | Vlasi-3 | G12 | A:A | 0.46336 | 3.55829 |
| 2031011 | Vlasi-3 | H12 | A:C | 2.47945 | 2.30783 |
| 2031011 | Vlasi-4 | A01 | A:A | 0.45924 | 3.47612 |

| | | | | | |
|---|---|---|---|---|---|
| 2031011 | Vlasi-4 | B01 | A:A | 0.4155 | 3.64416 |
| 2031011 | Vlasi-4 | C01 | A:A | 0.41451 | 3.87462 |
| 2031011 | Vlasi-4 | D01 | A:A | 0.40694 | 3.73668 |
| 2031011 | Vlasi-4 | E01 | C:C | 3.87704 | 0.54214 |
| 2031011 | Vlasi-4 | F01 | A:A | 0.40929 | 3.67371 |
| 2031011 | Vlasi-4 | G01 | A:C | 2.14455 | 1.94908 |
| 2031011 | Vlasi-4 | H01 | A:C | 2.48876 | 2.1593 |
| 2031011 | Vlasi-4 | A02 | A:A | 0.502 | 3.69643 |
| 2031011 | Vlasi-4 | B02 | A:A | 0.42405 | 3.42425 |
| 2031011 | Vlasi-4 | C02 | A:A | 0.44228 | 3.79501 |
| 2031011 | Vlasi-4 | D02 | A:C | 2.29788 | 2.09458 |
| 2031011 | Vlasi-4 | E02 | C:C | 3.67825 | 0.52698 |
| 2031011 | Vlasi-4 | F02 | A:C | 2.22376 | 1.97764 |
| 2031011 | Vlasi-4 | G02 | A:C | 2.3041 | 1.91342 |
| 2031011 | Vlasi-4 | H02 | A:C | 2.35924 | 2.15085 |
| 2031011 | Vlasi-4 | A03 | A:C | 2.27656 | 2.10173 |
| 2031011 | Vlasi-4 | B03 | A:C | 2.2353 | 2.11365 |
| 2031011 | Vlasi-4 | C03 | A:C | 2.13073 | 2.04827 |
| 2031011 | Vlasi-4 | D03 | A:C | 2.23437 | 2.10604 |
| 2031011 | Vlasi-4 | E03 | A:C | 2.13541 | 1.92681 |
| 2031011 | Vlasi-4 | F03 | C:C | 3.64826 | 0.5439 |
| 2031011 | Vlasi-4 | G03 | C:C | 3.63564 | 0.56799 |
| 2031011 | Vlasi-4 | H03 | A:A | 0.55758 | 3.61259 |
| 2031011 | Vlasi-4 | A04 | A:C | 2.31965 | 1.87654 |
| 2031011 | Vlasi-4 | B04 | A:A | 0.43155 | 3.55243 |
| 2031011 | Vlasi-4 | C04 | A:C | 2.22174 | 1.98891 |
| 2031011 | Vlasi-4 | D04 | A:C | 2.23394 | 2.031 |
| 2031011 | Vlasi-4 | E04 | A:A | 0.43714 | 3.61033 |
| 2031011 | Vlasi-4 | F04 | C:C | 3.5958 | 0.5374 |
| 2031011 | Vlasi-4 | G04 | A:C | 2.24856 | 2.00453 |
| 2031011 | Vlasi-4 | H04 | A:A | 0.55872 | 3.48714 |
| 2031011 | Vlasi-4 | A05 | C:C | 3.62122 | 0.54174 |
| 2031011 | Vlasi-4 | B05 | A:C | 2.30321 | 2.01062 |
| 2031011 | Vlasi-4 | C05 | A:A | 0.41699 | 3.72929 |
| 2031011 | Vlasi-4 | D05 | A:A | 0.41082 | 3.47814 |
| 2031011 | Vlasi-4 | E05 | A:C | 2.35107 | 1.97092 |
| 2031011 | Vlasi-4 | F05 | A:A | 0.41301 | 3.38664 |
| 2031011 | Vlasi-4 | G05 | A:A | 0.44761 | 3.43506 |
| 2031011 | Vlasi-4 | H05 | C:C | 3.69282 | 0.72808 |
| 2031011 | Vlasi-4 | A06 | A:C | 2.24321 | 2.00208 |
| 2031011 | Vlasi-4 | B06 | A:C | 2.13864 | 1.97102 |
| 2031011 | Vlasi-4 | C06 | C:C | 3.66393 | 0.52574 |
| 2031011 | Vlasi-4 | D06 | C:C | 3.62843 | 0.54012 |
| 2031011 | Vlasi-4 | E06 | A:C | 2.14194 | 1.91522 |
| 2031011 | Vlasi-4 | F06 | A:C | 2.14015 | 2.02473 |
| 2031011 | Vlasi-4 | G06 | A:C | 2.21396 | 1.9825 |
| 2031011 | Vlasi-4 | H06 | A:C | 2.3851 | 2.08783 |
| 2031011 | Vlasi-4 | A07 | ? | 0.6552 | 0.64623 |
| 2031011 | Vlasi-4 | B07 | ? | 0.54318 | 0.67163 |
| 2031011 | Vlasi-4 | C07 | ? | 0.57416 | 0.73144 |

| | | | | | |
|---|---|---|---|---|---|
| 2031011 | Vlasi-4 | D07 | ? | 2.71585 | 0.69054 |
| 2031011 | Vlasi-4 | E07 | ? | 0.60631 | 0.62924 |
| 2031011 | Vlasi-4 | F07 | C:C | 3.77138 | 0.61604 |
| 2031011 | Vlasi-4 | G07 | C:C | 3.80042 | 0.5967 |
| 2031011 | Vlasi-4 | H07 | C:C | 3.80603 | 0.72496 |
| 2031011 | Vlasi-4 | A08 | C:C | 3.64094 | 0.63216 |
| 2031011 | Vlasi-4 | B08 | C:C | 3.60032 | 0.61524 |
| 2031011 | Vlasi-4 | C08 | C:C | 3.55592 | 0.61851 |
| 2031011 | Vlasi-4 | D08 | C:C | 3.69002 | 0.61382 |
| 2031011 | Vlasi-4 | E08 | C:C | 3.71835 | 0.63355 |
| 2031011 | Vlasi-4 | F08 | C:C | 3.70986 | 0.59815 |
| 2031011 | Vlasi-4 | G08 | C:C | 3.66052 | 0.64766 |
| 2031011 | Vlasi-4 | H08 | C:C | 3.63116 | 0.71022 |
| 2031011 | Vlasi-4 | A09 | C:C | 3.58144 | 0.67334 |
| 2031011 | Vlasi-4 | B09 | C:C | 3.68013 | 0.63195 |
| 2031011 | Vlasi-4 | C09 | C:C | 3.60967 | 0.64517 |
| 2031011 | Vlasi-4 | D09 | C:C | 3.72029 | 0.60195 |
| 2031011 | Vlasi-4 | E09 | C:C | 3.64282 | 0.56535 |
| 2031011 | Vlasi-4 | F09 | C:C | 3.75511 | 0.62188 |
| 2031011 | Vlasi-4 | G09 | C:C | 3.67897 | 0.59639 |
| 2031011 | Vlasi-4 | H09 | C:C | 3.6388 | 0.70726 |
| 2031011 | Vlasi-4 | A10 | C:C | 3.60716 | 0.61782 |
| 2031011 | Vlasi-4 | B10 | C:C | 3.66962 | 0.57011 |
| 2031011 | Vlasi-4 | C10 | A:A | 0.42446 | 3.47747 |
| 2031011 | Vlasi-4 | D10 | A:A | 0.40399 | 3.48896 |
| 2031011 | Vlasi-4 | E10 | A:A | 0.44584 | 3.58547 |
| 2031011 | Vlasi-4 | F10 | A:A | 0.46129 | 3.78232 |
| 2031011 | Vlasi-4 | G10 | A:A | 0.42916 | 3.4103 |
| 2031011 | Vlasi-4 | H10 | A:A | 0.53606 | 3.45054 |
| 2031011 | Vlasi-4 | A11 | A:A | 0.47459 | 3.49003 |
| 2031011 | Vlasi-4 | B11 | A:A | 0.44293 | 3.58057 |
| 2031011 | Vlasi-4 | C11 | A:A | 0.47256 | 3.75726 |
| 2031011 | Vlasi-4 | D11 | A:A | 0.4346 | 3.72745 |
| 2031011 | Vlasi-4 | E11 | A:A | 0.42247 | 3.47623 |
| 2031011 | Vlasi-4 | F11 | A:A | 0.45344 | 3.64879 |
| 2031011 | Vlasi-4 | G11 | A:A | 0.4705 | 3.46958 |
| 2031011 | Vlasi-4 | H11 | A:A | 0.56318 | 3.59054 |
| 2031011 | Vlasi-4 | A12 | A:A | 0.6959 | 3.33141 |
| 2031011 | Vlasi-4 | B12 | A:A | 0.50744 | 3.57226 |
| 2031011 | Vlasi-4 | C12 | A:A | 0.4765 | 3.5978 |
| 2031011 | Vlasi-4 | D12 | A:A | 0.48254 | 3.55469 |
| 2031011 | Vlasi-4 | E12 | A:A | 0.50655 | 3.59211 |
| 2031011 | Vlasi-4 | F12 | A:A | 0.50538 | 3.62851 |
| 2031011 | Vlasi-4 | G12 | A:A | 0.50682 | 3.63939 |
| 2031011 | Vlasi-4 | H12 | NTC | 0.62882 | 0.58179 |
| 2031011 | Vlasi-1 | A01 | C:C | 0.50344 | 3.27993 |
| 2031011 | Vlasi-1 | B01 | C:G | 2.11877 | 1.99155 |
| 2031011 | Vlasi-1 | C01 | G:G | 3.52425 | 0.45012 |
| 2031011 | Vlasi-1 | D01 | C:G | 2.13593 | 1.82989 |
| 2031011 | Vlasi-1 | E01 | C:G | 2.0091 | 1.79377 |

| | | | | | |
|---|---|---|---|---|---|
| 2031011 | Vlasi-1 | F01 | C:G | 2.12485 | 1.93058 |
| 2031011 | Vlasi-1 | G01 | C:G | 2.00148 | 1.75727 |
| 2031011 | Vlasi-1 | H01 | C:G | 2.19715 | 2.12224 |
| 2031011 | Vlasi-1 | A02 | G:G | 3.3466 | 0.48331 |
| 2031011 | Vlasi-1 | B02 | C:C | 0.43304 | 3.32252 |
| 2031011 | Vlasi-1 | C02 | G:G | 3.3288 | 0.48961 |
| 2031011 | Vlasi-1 | D02 | C:G | 1.8497 | 1.92286 |
| 2031011 | Vlasi-1 | E02 | C:G | 2.05926 | 1.73097 |
| 2031011 | Vlasi-1 | F02 | C:G | 1.90599 | 1.863 |
| 2031011 | Vlasi-1 | G02 | C:G | 1.75536 | 1.837 |
| 2031011 | Vlasi-1 | H02 | C:G | 1.97626 | 1.93465 |
| 2031011 | Vlasi-1 | A03 | C:G | 2.10216 | 1.62904 |
| 2031011 | Vlasi-1 | B03 | G:G | 3.29927 | 0.41884 |
| 2031011 | Vlasi-1 | C03 | C:G | 1.96309 | 1.96572 |
| 2031011 | Vlasi-1 | D03 | C:G | 1.93272 | 1.81284 |
| 2031011 | Vlasi-1 | E03 | C:C | 0.35752 | 3.19725 |
| 2031011 | Vlasi-1 | F03 | C:G | 1.96835 | 1.80216 |
| 2031011 | Vlasi-1 | G03 | C:G | 1.97305 | 1.9725 |
| 2031011 | Vlasi-1 | H03 | G:G | 3.50403 | 0.54432 |
| 2031011 | Vlasi-1 | A04 | C:G | 2.03113 | 1.56905 |
| 2031011 | Vlasi-1 | B04 | C:C | 0.39277 | 3.19409 |
| 2031011 | Vlasi-1 | C04 | C:G | 2.10986 | 1.95721 |
| 2031011 | Vlasi-1 | D04 | G:G | 3.4626 | 0.4861 |
| 2031011 | Vlasi-1 | E04 | C:G | 1.99956 | 1.80929 |
| 2031011 | Vlasi-1 | F04 | ? | 0.58753 | 0.34176 |
| 2031011 | Vlasi-1 | G04 | C:G | 1.92788 | 1.7834 |
| 2031011 | Vlasi-1 | H04 | G:G | 3.47339 | 0.48407 |
| 2031011 | Vlasi-1 | A05 | G:G | 2.95266 | 0.44853 |
| 2031011 | Vlasi-1 | B05 | C:G | 2.13812 | 1.83553 |
| 2031011 | Vlasi-1 | C05 | C:C | 0.42057 | 3.44775 |
| 2031011 | Vlasi-1 | D05 | C:G | 1.99244 | 1.91057 |
| 2031011 | Vlasi-1 | E05 | C:G | 1.93497 | 1.93694 |
| 2031011 | Vlasi-1 | F05 | C:G | 1.81659 | 1.80136 |
| 2031011 | Vlasi-1 | G05 | C:G | 1.74242 | 1.85839 |
| 2031011 | Vlasi-1 | H05 | C:G | 2.08498 | 1.88832 |
| 2031011 | Vlasi-1 | A06 | C:G | 1.97675 | 1.92225 |
| 2031011 | Vlasi-1 | B06 | G:G | 3.42732 | 0.48985 |
| 2031011 | Vlasi-1 | C06 | C:G | 1.84478 | 1.9075 |
| 2031011 | Vlasi-1 | D06 | G:G | 3.55147 | 0.48266 |
| 2031011 | Vlasi-1 | E06 | G:G | 3.18419 | 0.42065 |
| 2031011 | Vlasi-1 | F06 | C:G | 1.78548 | 1.72444 |
| 2031011 | Vlasi-1 | G06 | C:G | 1.87366 | 1.7387 |
| 2031011 | Vlasi-1 | H06 | C:C | 0.43015 | 3.37135 |
| 2031011 | Vlasi-1 | A07 | G:G | 3.44541 | 0.47042 |
| 2031011 | Vlasi-1 | B07 | C:C | 0.409 | 3.16749 |
| 2031011 | Vlasi-1 | C07 | C:C | 0.44408 | 3.48421 |
| 2031011 | Vlasi-1 | D07 | G:G | 3.22619 | 0.45334 |
| 2031011 | Vlasi-1 | E07 | C:G | 1.88594 | 1.87303 |
| 2031011 | Vlasi-1 | F07 | C:G | 1.80289 | 1.8248 |
| 2031011 | Vlasi-1 | G07 | G:G | 3.17338 | 0.48206 |

| | | | | | |
|---|---|---|---|---|---|
| 2031011 | Vlasi-1 | H07 | G:G | 3.33435 | 0.50797 |
| 2031011 | Vlasi-1 | A08 | C:G | 1.95827 | 1.84427 |
| 2031011 | Vlasi-1 | B08 | C:G | 2.01871 | 1.76762 |
| 2031011 | Vlasi-1 | C08 | C:G | 2.00317 | 1.91621 |
| 2031011 | Vlasi-1 | D08 | C:C | 0.37421 | 3.13712 |
| 2031011 | Vlasi-1 | E08 | C:C | 0.39547 | 3.35663 |
| 2031011 | Vlasi-1 | F08 | C:G | 1.96098 | 1.81992 |
| 2031011 | Vlasi-1 | G08 | G:G | 2.87729 | 0.42792 |
| 2031011 | Vlasi-1 | H08 | C:G | 2.03817 | 1.88266 |
| 2031011 | Vlasi-1 | A09 | C:G | 2.02685 | 1.73158 |
| 2031011 | Vlasi-1 | B09 | C:G | 2.06073 | 1.87093 |
| 2031011 | Vlasi-1 | C09 | C:G | 2.04869 | 1.92182 |
| 2031011 | Vlasi-1 | D09 | C:C | 0.41219 | 3.29758 |
| 2031011 | Vlasi-1 | E09 | C:G | 2.03799 | 1.89787 |
| 2031011 | Vlasi-1 | F09 | C:G | 1.99522 | 1.75527 |
| 2031011 | Vlasi-1 | G09 | C:C | 0.4523 | 3.15215 |
| 2031011 | Vlasi-1 | H09 | C:G | 1.9939 | 2.06524 |
| 2031011 | Vlasi-1 | A10 | G:G | 3.2677 | 0.48605 |
| 2031011 | Vlasi-1 | B10 | C:G | 2.09156 | 1.80434 |
| 2031011 | Vlasi-1 | C10 | C:G | 2.05532 | 1.958 |
| 2031011 | Vlasi-1 | D10 | C:C | 0.38927 | 3.29441 |
| 2031011 | Vlasi-1 | E10 | G:G | 3.18151 | 0.42793 |
| 2031011 | Vlasi-1 | F10 | G:G | 3.46273 | 0.4696 |
| 2031011 | Vlasi-1 | G10 | C:C | 0.40483 | 3.38132 |
| 2031011 | Vlasi-1 | H10 | G:G | 3.22817 | 0.46072 |
| 2031011 | Vlasi-1 | A11 | C:C | 0.43546 | 3.01036 |
| 2031011 | Vlasi-1 | B11 | G:G | 3.35881 | 0.48721 |
| 2031011 | Vlasi-1 | C11 | C:G | 2.05083 | 2.06659 |
| 2031011 | Vlasi-1 | D11 | C:G | 1.96046 | 1.98003 |
| 2031011 | Vlasi-1 | E11 | G:G | 3.37441 | 0.45607 |
| 2031011 | Vlasi-1 | F11 | C:G | 2.07488 | 1.90996 |
| 2031011 | Vlasi-1 | G11 | C:C | 0.39776 | 3.22279 |
| 2031011 | Vlasi-1 | H11 | C:G | 1.95344 | 1.8188 |
| 2031011 | Vlasi-1 | A12 | C:G | 1.99868 | 1.81067 |
| 2031011 | Vlasi-1 | B12 | C:G | 1.98454 | 1.85583 |
| 2031011 | Vlasi-1 | C12 | C:C | 0.40398 | 3.25495 |
| 2031011 | Vlasi-1 | D12 | C:G | 2.02511 | 2.06411 |
| 2031011 | Vlasi-1 | E12 | C:G | 2.01914 | 1.87245 |
| 2031011 | Vlasi-1 | F12 | C:G | 2.02383 | 1.86608 |
| 2031011 | Vlasi-1 | G12 | C:G | 2.16809 | 1.93002 |
| 2031011 | Vlasi-1 | H12 | G:G | 3.51649 | 0.47898 |
| 2031011 | Vlasi-2 | A01 | C:G | 2.1486 | 1.75598 |
| 2031011 | Vlasi-2 | B01 | C:G | 2.03429 | 1.87835 |
| 2031011 | Vlasi-2 | C01 | G:G | 3.28393 | 0.39542 |
| 2031011 | Vlasi-2 | D01 | C:G | 1.89877 | 1.71578 |
| 2031011 | Vlasi-2 | E01 | C:C | 0.38886 | 3.24195 |
| 2031011 | Vlasi-2 | F01 | C:G | 1.90678 | 1.78573 |
| 2031011 | Vlasi-2 | G01 | C:C | 0.41669 | 3.18193 |
| 2031011 | Vlasi-2 | H01 | C:C | 0.47033 | 3.42383 |
| 2031011 | Vlasi-2 | A02 | C:G | 2.10152 | 1.75513 |

| | | | | | |
|---|---|---|---|---|---|
| 2031011 | Vlasi-2 | B02 | C:G | 2.07952 | 1.74746 |
| 2031011 | Vlasi-2 | C02 | C:G | 2.09862 | 1.9112 |
| 2031011 | Vlasi-2 | D02 | C:G | 1.97698 | 1.86412 |
| 2031011 | Vlasi-2 | E02 | C:G | 1.94498 | 1.83816 |
| 2031011 | Vlasi-2 | F02 | C:G | 1.83425 | 1.91534 |
| 2031011 | Vlasi-2 | G02 | C:G | 1.80792 | 1.84693 |
| 2031011 | Vlasi-2 | H02 | C:G | 1.97256 | 1.96874 |
| 2031011 | Vlasi-2 | A03 | C:C | 0.44064 | 3.14935 |
| 2031011 | Vlasi-2 | B03 | G:G | 3.394 | 0.47162 |
| 2031011 | Vlasi-2 | C03 | G:G | 3.22276 | 0.38813 |
| 2031011 | Vlasi-2 | D03 | C:G | 1.88676 | 1.78621 |
| 2031011 | Vlasi-2 | E03 | C:G | 1.87682 | 1.83119 |
| 2031011 | Vlasi-2 | F03 | C:C | 0.40769 | 3.16118 |
| 2031011 | Vlasi-2 | G03 | C:G | 2.03237 | 1.88091 |
| 2031011 | Vlasi-2 | H03 | C:G | 2.07217 | 2.08418 |
| 2031011 | Vlasi-2 | A04 | C:G | 2.05442 | 1.79289 |
| 2031011 | Vlasi-2 | B04 | C:G | 2.00715 | 1.93526 |
| 2031011 | Vlasi-2 | C04 | C:C | 0.38124 | 3.26842 |
| 2031011 | Vlasi-2 | D04 | C:C | 0.40181 | 3.3398 |
| 2031011 | Vlasi-2 | E04 | G:G | 3.19545 | 0.45053 |
| 2031011 | Vlasi-2 | F04 | C:G | 1.83204 | 1.95318 |
| 2031011 | Vlasi-2 | G04 | C:C | 0.46089 | 3.2553 |
| 2031011 | Vlasi-2 | H04 | G:G | 3.47253 | 0.48551 |
| 2031011 | Vlasi-2 | A05 | C:G | 2.12958 | 1.7316 |
| 2031011 | Vlasi-2 | B05 | C:C | 0.38948 | 3.24857 |
| 2031011 | Vlasi-2 | C05 | C:G | 1.98593 | 1.87223 |
| 2031011 | Vlasi-2 | D05 | C:G | 1.84559 | 1.87591 |
| 2031011 | Vlasi-2 | E05 | C:G | 1.93983 | 1.88368 |
| 2031011 | Vlasi-2 | F05 | G:G | 3.20579 | 0.45045 |
| 2031011 | Vlasi-2 | G05 | G:G | 3.33457 | 0.49834 |
| 2031011 | Vlasi-2 | H05 | C:G | 2.0103 | 2.00792 |
| 2031011 | Vlasi-2 | A06 | G:G | 3.11261 | 0.42638 |
| 2031011 | Vlasi-2 | B06 | C:G | 2.1796 | 1.85814 |
| 2031011 | Vlasi-2 | C06 | G:G | 3.21677 | 0.40962 |
| 2031011 | Vlasi-2 | D06 | C:G | 2.04432 | 1.86951 |
| 2031011 | Vlasi-2 | E06 | C:G | 1.9831 | 1.90914 |
| 2031011 | Vlasi-2 | F06 | C:G | 1.92589 | 1.76728 |
| 2031011 | Vlasi-2 | G06 | C:G | 1.96222 | 1.80759 |
| 2031011 | Vlasi-2 | H06 | C:G | 2.02067 | 1.85673 |
| 2031011 | Vlasi-2 | A07 | C:C | 0.42646 | 3.06644 |
| 2031011 | Vlasi-2 | B07 | C:C | 0.42441 | 3.02155 |
| 2031011 | Vlasi-2 | C07 | C:G | 1.82784 | 1.72762 |
| 2031011 | Vlasi-2 | D07 | C:C | 0.39842 | 3.31385 |
| 2031011 | Vlasi-2 | E07 | C:G | 1.9369 | 1.72384 |
| 2031011 | Vlasi-2 | F07 | C:G | 1.92458 | 1.77789 |
| 2031011 | Vlasi-2 | G07 | C:C | 0.424 | 3.20999 |
| 2031011 | Vlasi-2 | H07 | G:G | 3.45308 | 0.51433 |
| 2031011 | Vlasi-2 | A08 | C:C | 0.47521 | 3.33385 |
| 2031011 | Vlasi-2 | B08 | C:G | 1.9986 | 1.84388 |
| 2031011 | Vlasi-2 | C08 | C:C | 0.44968 | 3.13835 |

| | | | | | |
|---|---|---|---|---|---|
| 2031011 | Vlasi-2 | D08 | C:C | 0.3969 | 3.29986 |
| 2031011 | Vlasi-2 | E08 | C:C | 0.3878 | 3.24916 |
| 2031011 | Vlasi-2 | F08 | C:G | 1.85652 | 1.97487 |
| 2031011 | Vlasi-2 | G08 | C:G | 2.01646 | 1.82343 |
| 2031011 | Vlasi-2 | H08 | C:G | 1.97171 | 1.80735 |
| 2031011 | Vlasi-2 | A09 | C:G | 2.06539 | 1.60616 |
| 2031011 | Vlasi-2 | B09 | G:G | 3.41267 | 0.46724 |
| 2031011 | Vlasi-2 | C09 | C:G | 1.78341 | 1.55101 |
| 2031011 | Vlasi-2 | D09 | C:C | 0.42107 | 3.31701 |
| 2031011 | Vlasi-2 | E09 | G:G | 3.23602 | 0.44588 |
| 2031011 | Vlasi-2 | F09 | ? | 0.49197 | 1.66228 |
| 2031011 | Vlasi-2 | G09 | C:G | 1.79326 | 1.93982 |
| 2031011 | Vlasi-2 | H09 | C:C | 0.43011 | 3.40865 |
| 2031011 | Vlasi-2 | A10 | C:G | 1.96362 | 1.75195 |
| 2031011 | Vlasi-2 | B10 | G:G | 3.36745 | 0.47577 |
| 2031011 | Vlasi-2 | C10 | C:G | 1.96249 | 1.74023 |
| 2031011 | Vlasi-2 | D10 | C:G | 1.96094 | 1.77468 |
| 2031011 | Vlasi-2 | E10 | C:G | 2.06881 | 1.84452 |
| 2031011 | Vlasi-2 | F10 | C:G | 2.04567 | 1.89196 |
| 2031011 | Vlasi-2 | G10 | C:C | 0.44052 | 3.4204 |
| 2031011 | Vlasi-2 | H10 | C:G | 2.07129 | 1.98422 |
| 2031011 | Vlasi-2 | A11 | G:G | 3.39516 | 0.49774 |
| 2031011 | Vlasi-2 | B11 | C:G | 2.00082 | 1.74037 |
| 2031011 | Vlasi-2 | C11 | C:G | 2.07013 | 1.88469 |
| 2031011 | Vlasi-2 | D11 | C:G | 2.03075 | 1.70732 |
| 2031011 | Vlasi-2 | E11 | G:G | 3.19987 | 0.48748 |
| 2031011 | Vlasi-2 | F11 | G:G | 3.31244 | 0.44541 |
| 2031011 | Vlasi-2 | G11 | C:C | 0.45323 | 3.33249 |
| 2031011 | Vlasi-2 | H11 | C:G | 2.06281 | 2.01759 |
| 2031011 | Vlasi-2 | A12 | C:G | 2.05331 | 1.61552 |
| 2031011 | Vlasi-2 | B12 | C:G | 2.11243 | 1.85876 |
| 2031011 | Vlasi-2 | C12 | C:G | 2.24778 | 1.93849 |
| 2031011 | Vlasi-2 | D12 | C:G | 2.15756 | 1.84956 |
| 2031011 | Vlasi-2 | E12 | G:G | 3.37417 | 0.4659 |
| 2031011 | Vlasi-2 | F12 | G:G | 3.59588 | 0.53993 |
| 2031011 | Vlasi-2 | G12 | C:G | 2.11805 | 1.81526 |
| 2031011 | Vlasi-2 | H12 | C:G | 2.14787 | 1.85948 |
| 2031011 | Vlasi-3 | A01 | G:G | 3.613 | 0.42918 |
| 2031011 | Vlasi-3 | B01 | C:C | 0.44123 | 3.41353 |
| 2031011 | Vlasi-3 | C01 | C:G | 2.00993 | 1.90051 |
| 2031011 | Vlasi-3 | D01 | G:G | 3.57788 | 0.42696 |
| 2031011 | Vlasi-3 | E01 | C:C | 0.38606 | 3.36135 |
| 2031011 | Vlasi-3 | F01 | G:G | 3.4601 | 0.44229 |
| 2031011 | Vlasi-3 | G01 | C:C | 0.39152 | 3.31684 |
| 2031011 | Vlasi-3 | H01 | C:G | 2.26482 | 1.85365 |
| 2031011 | Vlasi-3 | A02 | G:G | 3.65507 | 0.48748 |
| 2031011 | Vlasi-3 | B02 | C:C | 0.41393 | 3.4677 |
| 2031011 | Vlasi-3 | C02 | C:C | 0.38347 | 3.3506 |
| 2031011 | Vlasi-3 | D02 | C:C | 0.38832 | 3.28634 |
| 2031011 | Vlasi-3 | E02 | C:G | 1.90752 | 1.8048 |

| | | | | | |
|---|---|---|---|---|---|
| 2031011 | Vlasi-3 | F02 | C:C | 0.43543 | 3.33835 |
| 2031011 | Vlasi-3 | G02 | C:G | 1.91919 | 1.82623 |
| 2031011 | Vlasi-3 | H02 | C:C | 0.46562 | 3.45067 |
| 2031011 | Vlasi-3 | A03 | C:C | 0.46725 | 3.12086 |
| 2031011 | Vlasi-3 | B03 | G:G | 3.28359 | 0.46391 |
| 2031011 | Vlasi-3 | C03 | C:G | 1.90449 | 1.77289 |
| 2031011 | Vlasi-3 | D03 | C:G | 1.93345 | 1.85339 |
| 2031011 | Vlasi-3 | E03 | G:G | 3.39277 | 0.47104 |
| 2031011 | Vlasi-3 | F03 | G:G | 3.21035 | 0.49393 |
| 2031011 | Vlasi-3 | G03 | C:C | 0.44422 | 3.34668 |
| 2031011 | Vlasi-3 | H03 | C:C | 0.44723 | 3.43788 |
| 2031011 | Vlasi-3 | A04 | C:C | 0.43769 | 3.2204 |
| 2031011 | Vlasi-3 | B04 | C:G | 1.92017 | 1.94451 |
| 2031011 | Vlasi-3 | C04 | C:G | 2.06836 | 1.9335 |
| 2031011 | Vlasi-3 | D04 | C:C | 0.39524 | 3.18329 |
| 2031011 | Vlasi-3 | E04 | ? | 2.11152 | 0.41515 |
| 2031011 | Vlasi-3 | F04 | C:C | 0.43484 | 3.32942 |
| 2031011 | Vlasi-3 | G04 | C:G | 1.92039 | 1.82148 |
| 2031011 | Vlasi-3 | H04 | C:G | 2.17793 | 2.02029 |
| 2031011 | Vlasi-3 | A05 | C:G | 2.0433 | 1.75556 |
| 2031011 | Vlasi-3 | B05 | C:G | 2.21689 | 1.97383 |
| 2031011 | Vlasi-3 | C05 | C:G | 1.96111 | 2.02613 |
| 2031011 | Vlasi-3 | D05 | C:C | 0.43728 | 3.24699 |
| 2031011 | Vlasi-3 | E05 | C:G | 1.89417 | 1.72988 |
| 2031011 | Vlasi-3 | F05 | C:G | 1.8447 | 1.71745 |
| 2031011 | Vlasi-3 | G05 | C:C | 0.43595 | 3.326 |
| 2031011 | Vlasi-3 | H05 | C:G | 2.27678 | 2.16146 |
| 2031011 | Vlasi-3 | A06 | C:G | 1.98852 | 1.8951 |
| 2031011 | Vlasi-3 | B06 | C:G | 1.96549 | 1.83879 |
| 2031011 | Vlasi-3 | C06 | G:G | 3.14634 | 0.41383 |
| 2031011 | Vlasi-3 | D06 | C:G | 1.82792 | 1.85237 |
| 2031011 | Vlasi-3 | E06 | C:G | 1.91736 | 1.88897 |
| 2031011 | Vlasi-3 | F06 | G:G | 3.24866 | 0.47967 |
| 2031011 | Vlasi-3 | G06 | C:G | 2.03029 | 1.98824 |
| 2031011 | Vlasi-3 | H06 | C:G | 2.20055 | 2.13896 |
| 2031011 | Vlasi-3 | A07 | C:G | 1.92977 | 1.69099 |
| 2031011 | Vlasi-3 | B07 | C:G | 1.90598 | 1.78232 |
| 2031011 | Vlasi-3 | C07 | C:G | 1.80714 | 1.71002 |
| 2031011 | Vlasi-3 | D07 | C:G | 1.979 | 1.72335 |
| 2031011 | Vlasi-3 | E07 | C:G | 1.94154 | 1.79733 |
| 2031011 | Vlasi-3 | F07 | G:G | 3.2793 | 0.43967 |
| 2031011 | Vlasi-3 | G07 | C:G | 1.95825 | 1.82017 |
| 2031011 | Vlasi-3 | H07 | C:C | 0.50018 | 3.4658 |
| 2031011 | Vlasi-3 | A08 | C:G | 2.10857 | 1.88319 |
| 2031011 | Vlasi-3 | B08 | C:G | 1.98178 | 1.72447 |
| 2031011 | Vlasi-3 | C08 | G:G | 3.30878 | 0.4483 |
| 2031011 | Vlasi-3 | D08 | C:G | 1.95578 | 1.91413 |
| 2031011 | Vlasi-3 | E08 | G:G | 3.35644 | 0.46151 |
| 2031011 | Vlasi-3 | F08 | G:G | 3.22823 | 0.44546 |
| 2031011 | Vlasi-3 | G08 | C:C | 0.44961 | 3.34059 |

| | | | | | |
|---|---|---|---|---|---|
| 2031011 | Vlasi-3 | H08 | C:G | 2.19214 | 1.88456 |
| 2031011 | Vlasi-3 | A09 | C:G | 2.06095 | 1.79284 |
| 2031011 | Vlasi-3 | B09 | G:G | 3.46089 | 0.46321 |
| 2031011 | Vlasi-3 | C09 | C:G | 1.98421 | 1.82675 |
| 2031011 | Vlasi-3 | D09 | C:G | 2.08996 | 1.7304 |
| 2031011 | Vlasi-3 | E09 | C:G | 1.86467 | 1.97931 |
| 2031011 | Vlasi-3 | F09 | G:G | 3.26889 | 0.45714 |
| 2031011 | Vlasi-3 | G09 | C:G | 1.95097 | 1.86901 |
| 2031011 | Vlasi-3 | H09 | C:G | 2.22108 | 2.06315 |
| 2031011 | Vlasi-3 | A10 | C:G | 1.9404 | 1.65944 |
| 2031011 | Vlasi-3 | B10 | C:G | 1.94402 | 1.95842 |
| 2031011 | Vlasi-3 | C10 | C:G | 2.03876 | 2.03087 |
| 2031011 | Vlasi-3 | D10 | G:G | 3.50572 | 0.48663 |
| 2031011 | Vlasi-3 | E10 | C:G | 2.06351 | 1.88049 |
| 2031011 | Vlasi-3 | F10 | C:G | 1.97148 | 1.81151 |
| 2031011 | Vlasi-3 | G10 | G:G | 3.51301 | 0.4913 |
| 2031011 | Vlasi-3 | H10 | C:C | 0.44149 | 3.38677 |
| 2031011 | Vlasi-3 | A11 | C:C | 0.41825 | 3.29389 |
| 2031011 | Vlasi-3 | B11 | G:G | 3.40547 | 0.49084 |
| 2031011 | Vlasi-3 | C11 | C:C | 0.42699 | 3.38907 |
| 2031011 | Vlasi-3 | D11 | G:G | 3.41912 | 0.45037 |
| 2031011 | Vlasi-3 | E11 | C:G | 2.00818 | 1.78939 |
| 2031011 | Vlasi-3 | F11 | C:C | 0.4256 | 3.36049 |
| 2031011 | Vlasi-3 | G11 | C:G | 2.01837 | 1.77906 |
| 2031011 | Vlasi-3 | H11 | C:G | 2.34745 | 2.02398 |
| 2031011 | Vlasi-3 | A12 | C:C | 0.43399 | 3.10096 |
| 2031011 | Vlasi-3 | B12 | C:C | 0.40306 | 3.21288 |
| 2031011 | Vlasi-3 | C12 | C:G | 1.93387 | 1.93111 |
| 2031011 | Vlasi-3 | D12 | C:G | 2.12489 | 1.86059 |
| 2031011 | Vlasi-3 | E12 | C:G | 1.99245 | 1.94648 |
| 2031011 | Vlasi-3 | F12 | C:G | 2.08887 | 2.02892 |
| 2031011 | Vlasi-3 | G12 | ? | 0.64961 | 0.42151 |
| 2031011 | Vlasi-3 | H12 | C:G | 2.26299 | 2.15813 |
| 2031011 | Vlasi-4 | A01 | C:C | 0.44589 | 3.12707 |
| 2031011 | Vlasi-4 | B01 | C:C | 0.4323 | 3.34791 |
| 2031011 | Vlasi-4 | C01 | C:G | 1.93702 | 1.74376 |
| 2031011 | Vlasi-4 | D01 | C:C | 0.42312 | 3.19141 |
| 2031011 | Vlasi-4 | E01 | C:G | 1.70941 | 1.75075 |
| 2031011 | Vlasi-4 | F01 | C:C | 0.42671 | 3.34283 |
| 2031011 | Vlasi-4 | G01 | G:G | 3.36932 | 0.48689 |
| 2031011 | Vlasi-4 | H01 | G:G | 3.45371 | 0.51438 |
| 2031011 | Vlasi-4 | A02 | C:C | 0.43492 | 3.30746 |
| 2031011 | Vlasi-4 | B02 | C:G | 1.93853 | 1.88445 |
| 2031011 | Vlasi-4 | C02 | G:G | 3.26126 | 0.44919 |
| 2031011 | Vlasi-4 | D02 | C:G | 1.99387 | 1.788 |
| 2031011 | Vlasi-4 | E02 | G:G | 3.29503 | 0.45863 |
| 2031011 | Vlasi-4 | F02 | C:G | 1.88536 | 1.82154 |
| 2031011 | Vlasi-4 | G02 | C:C | 0.43758 | 3.317 |
| 2031011 | Vlasi-4 | H02 | C:G | 2.14512 | 1.85865 |
| 2031011 | Vlasi-4 | A03 | C:C | 0.41699 | 3.38006 |

| | | | | | |
|---|---|---|---|---|---|
| 2031011 | Vlasi-4 | B03 | G:G | 3.35512 | 0.43746 |
| 2031011 | Vlasi-4 | C03 | C:G | 2.00249 | 1.78431 |
| 2031011 | Vlasi-4 | D03 | C:G | 1.89068 | 1.75054 |
| 2031011 | Vlasi-4 | E03 | C:C | 0.43271 | 3.19947 |
| 2031011 | Vlasi-4 | F03 | C:C | 0.39452 | 3.22557 |
| 2031011 | Vlasi-4 | G03 | C:G | 2.03224 | 1.85626 |
| 2031011 | Vlasi-4 | H03 | C:G | 2.23389 | 1.92674 |
| 2031011 | Vlasi-4 | A04 | C:G | 1.9354 | 1.71114 |
| 2031011 | Vlasi-4 | B04 | C:G | 2.01471 | 1.71116 |
| 2031011 | Vlasi-4 | C04 | C:C | 0.42388 | 3.40929 |
| 2031011 | Vlasi-4 | D04 | C:G | 1.84106 | 1.7622 |
| 2031011 | Vlasi-4 | E04 | C:G | 1.93371 | 1.69702 |
| 2031011 | Vlasi-4 | F04 | C:G | 1.79156 | 1.79767 |
| 2031011 | Vlasi-4 | G04 | C:G | 1.93954 | 1.755 |
| 2031011 | Vlasi-4 | H04 | C:G | 2.16968 | 1.89833 |
| 2031011 | Vlasi-4 | A05 | G:G | 3.34618 | 0.43609 |
| 2031011 | Vlasi-4 | B05 | G:G | 3.22765 | 0.44171 |
| 2031011 | Vlasi-4 | C05 | C:G | 2.0465 | 1.86165 |
| 2031011 | Vlasi-4 | D05 | C:G | 1.83773 | 1.81903 |
| 2031011 | Vlasi-4 | E05 | C:G | 1.83072 | 1.86831 |
| 2031011 | Vlasi-4 | F05 | C:G | 1.83933 | 1.8817 |
| 2031011 | Vlasi-4 | G05 | C:G | 1.86691 | 1.77423 |
| 2031011 | Vlasi-4 | H05 | C:G | 2.1746 | 1.9877 |
| 2031011 | Vlasi-4 | A06 | C:G | 1.96279 | 1.79675 |
| 2031011 | Vlasi-4 | B06 | G:G | 3.18866 | 0.44232 |
| 2031011 | Vlasi-4 | C06 | G:G | 3.17414 | 0.48691 |
| 2031011 | Vlasi-4 | D06 | C:G | 1.85882 | 1.48347 |
| 2031011 | Vlasi-4 | E06 | C:C | 0.39931 | 3.37483 |
| 2031011 | Vlasi-4 | F06 | C:G | 1.85721 | 1.68286 |
| 2031011 | Vlasi-4 | G06 | G:G | 2.99857 | 0.49612 |
| 2031011 | Vlasi-4 | H06 | C:G | 2.21098 | 1.88913 |
| 2031011 | Vlasi-4 | A07 | G:G | 3.07274 | 0.64478 |
| 2031011 | Vlasi-4 | B07 | G:G | 3.35146 | 0.4318 |
| 2031011 | Vlasi-4 | C07 | G:G | 3.25867 | 0.54941 |
| 2031011 | Vlasi-4 | D07 | G:G | 3.05611 | 0.87423 |
| 2031011 | Vlasi-4 | E07 | G:G | 3.45347 | 0.49957 |
| 2031011 | Vlasi-4 | F07 | G:G | 3.55316 | 0.51003 |
| 2031011 | Vlasi-4 | G07 | G:G | 3.58265 | 0.51856 |
| 2031011 | Vlasi-4 | H07 | G:G | 3.94035 | 0.58148 |
| 2031011 | Vlasi-4 | A08 | G:G | 3.46015 | 0.48837 |
| 2031011 | Vlasi-4 | B08 | G:G | 3.26202 | 0.4978 |
| 2031011 | Vlasi-4 | C08 | G:G | 3.49042 | 0.49499 |
| 2031011 | Vlasi-4 | D08 | G:G | 3.59625 | 0.54752 |
| 2031011 | Vlasi-4 | E08 | G:G | 3.50107 | 0.49806 |
| 2031011 | Vlasi-4 | F08 | G:G | 3.58357 | 0.55941 |
| 2031011 | Vlasi-4 | G08 | G:G | 3.55905 | 0.55219 |
| 2031011 | Vlasi-4 | H08 | G:G | 3.63655 | 0.5871 |
| 2031011 | Vlasi-4 | A09 | G:G | 3.59795 | 0.52289 |
| 2031011 | Vlasi-4 | B09 | G:G | 3.55864 | 0.4993 |
| 2031011 | Vlasi-4 | C09 | G:G | 3.68325 | 0.55839 |

| | | | | | |
|---|---|---|---|---|---|
| 2031011 | Vlasi-4 | D09 | G:G | 3.56143 | 0.51602 |
| 2031011 | Vlasi-4 | E09 | G:G | 3.49774 | 0.46094 |
| 2031011 | Vlasi-4 | F09 | G:G | 3.42759 | 0.53518 |
| 2031011 | Vlasi-4 | G09 | G:G | 3.34359 | 0.58306 |
| 2031011 | Vlasi-4 | H09 | G:G | 3.68074 | 0.56018 |
| 2031011 | Vlasi-4 | A10 | G:G | 3.48844 | 0.50201 |
| 2031011 | Vlasi-4 | B10 | G:G | 3.3545 | 0.50538 |
| 2031011 | Vlasi-4 | C10 | C:C | 0.43516 | 3.4585 |
| 2031011 | Vlasi-4 | D10 | C:C | 0.46393 | 3.19703 |
| 2031011 | Vlasi-4 | E10 | C:C | 0.43724 | 3.49683 |
| 2031011 | Vlasi-4 | F10 | C:C | 0.51662 | 3.34168 |
| 2031011 | Vlasi-4 | G10 | C:C | 0.4754 | 3.42845 |
| 2031011 | Vlasi-4 | H10 | C:C | 0.66352 | 3.2259 |
| 2031011 | Vlasi-4 | A11 | C:C | 0.4435 | 3.2292 |
| 2031011 | Vlasi-4 | B11 | C:C | 0.50993 | 3.40089 |
| 2031011 | Vlasi-4 | C11 | C:C | 0.46292 | 3.39047 |
| 2031011 | Vlasi-4 | D11 | C:C | 0.67716 | 3.22351 |
| 2031011 | Vlasi-4 | E11 | C:C | 0.55236 | 3.21881 |
| 2031011 | Vlasi-4 | F11 | C:C | 0.63072 | 2.91798 |
| 2031011 | Vlasi-4 | G11 | C:C | 0.49234 | 3.23553 |
| 2031011 | Vlasi-4 | H11 | C:C | 0.52754 | 3.33057 |
| 2031011 | Vlasi-4 | A12 | C:C | 0.4847 | 2.99928 |
| 2031011 | Vlasi-4 | B12 | C:C | 0.56704 | 3.23056 |
| 2031011 | Vlasi-4 | C12 | C:C | 0.5737 | 3.36822 |
| 2031011 | Vlasi-4 | D12 | C:C | 0.54527 | 3.47546 |
| 2031011 | Vlasi-4 | E12 | C:C | 0.52461 | 3.18703 |
| 2031011 | Vlasi-4 | F12 | C:C | 0.48827 | 3.5195 |
| 2031011 | Vlasi-4 | G12 | C:C | 0.52474 | 3.43734 |
| 2031011 | Vlasi-4 | H12 | NTC | 3.07652 | 0.71324 |
| 2031012 | Vlasi-1 | A01 | C:C | 0.44609 | 3.48801 |
| 2031012 | Vlasi-1 | B01 | C:A | 2.0475 | 2.55035 |
| 2031012 | Vlasi-1 | C01 | C:C | 0.38646 | 3.60287 |
| 2031012 | Vlasi-1 | D01 | A:A | 3.80618 | 0.44027 |
| 2031012 | Vlasi-1 | E01 | C:A | 1.78032 | 2.64908 |
| 2031012 | Vlasi-1 | F01 | C:C | 0.36334 | 3.60601 |
| 2031012 | Vlasi-1 | G01 | C:C | 0.38201 | 3.71551 |
| 2031012 | Vlasi-1 | H01 | C:C | 0.4091 | 3.75402 |
| 2031012 | Vlasi-1 | A02 | C:C | 0.55654 | 3.31045 |
| 2031012 | Vlasi-1 | B02 | C:A | 1.95767 | 2.76562 |
| 2031012 | Vlasi-1 | C02 | C:A | 1.75933 | 2.38496 |
| 2031012 | Vlasi-1 | D02 | C:A | 1.70735 | 2.50159 |
| 2031012 | Vlasi-1 | E02 | C:A | 1.72231 | 2.56557 |
| 2031012 | Vlasi-1 | F02 | C:C | 0.42185 | 3.59977 |
| 2031012 | Vlasi-1 | G02 | C:A | 1.7635 | 2.30575 |
| 2031012 | Vlasi-1 | H02 | C:A | 1.71243 | 2.57953 |
| 2031012 | Vlasi-1 | A03 | C:A | 1.77273 | 2.45894 |
| 2031012 | Vlasi-1 | B03 | C:A | 1.7847 | 2.56979 |
| 2031012 | Vlasi-1 | C03 | C:A | 1.74893 | 2.63209 |
| 2031012 | Vlasi-1 | D03 | C:A | 1.77619 | 2.57226 |
| 2031012 | Vlasi-1 | E03 | C:C | 0.41273 | 3.53561 |

| | | | | | |
|---|---|---|---|---|---|
| 2031012 | Vlasi-1 | F03 | C:C | 0.44157 | 3.4986 |
| 2031012 | Vlasi-1 | G03 | C:C | 0.40884 | 3.4346 |
| 2031012 | Vlasi-1 | H03 | C:A | 1.70504 | 2.52305 |
| 2031012 | Vlasi-1 | A04 | A:A | 3.47127 | 0.54335 |
| 2031012 | Vlasi-1 | B04 | C:C | 0.45865 | 3.52441 |
| 2031012 | Vlasi-1 | C04 | C:C | 0.43752 | 3.64281 |
| 2031012 | Vlasi-1 | D04 | C:A | 1.62497 | 2.38324 |
| 2031012 | Vlasi-1 | E04 | C:A | 1.68451 | 2.50666 |
| 2031012 | Vlasi-1 | F04 | C:C | 0.45854 | 3.70037 |
| 2031012 | Vlasi-1 | G04 | C:A | 1.64042 | 2.40471 |
| 2031012 | Vlasi-1 | H04 | C:A | 1.74716 | 2.48992 |
| 2031012 | Vlasi-1 | A05 | C:A | 1.93109 | 2.41946 |
| 2031012 | Vlasi-1 | B05 | C:C | 0.43427 | 3.39498 |
| 2031012 | Vlasi-1 | C05 | C:C | 0.41835 | 3.29796 |
| 2031012 | Vlasi-1 | D05 | C:C | 0.41689 | 3.47393 |
| 2031012 | Vlasi-1 | E05 | C:A | 1.70545 | 2.45728 |
| 2031012 | Vlasi-1 | F05 | C:A | 1.75042 | 2.40648 |
| 2031012 | Vlasi-1 | G05 | A:A | 3.63523 | 0.46967 |
| 2031012 | Vlasi-1 | H05 | C:A | 1.68684 | 2.48465 |
| 2031012 | Vlasi-1 | A06 | C:C | 0.53576 | 3.47609 |
| 2031012 | Vlasi-1 | B06 | C:A | 1.76511 | 2.52967 |
| 2031012 | Vlasi-1 | C06 | C:C | 0.40947 | 3.52016 |
| 2031012 | Vlasi-1 | D06 | C:C | 0.40762 | 3.49885 |
| 2031012 | Vlasi-1 | E06 | C:A | 1.58817 | 2.72064 |
| 2031012 | Vlasi-1 | F06 | C:C | 0.41673 | 3.55032 |
| 2031012 | Vlasi-1 | G06 | C:A | 1.68261 | 2.3641 |
| 2031012 | Vlasi-1 | H06 | C:C | 0.41538 | 3.6141 |
| 2031012 | Vlasi-1 | A07 | C:A | 2.10716 | 2.50734 |
| 2031012 | Vlasi-1 | B07 | C:C | 0.4624 | 3.53783 |
| 2031012 | Vlasi-1 | C07 | C:C | 0.42232 | 3.53628 |
| 2031012 | Vlasi-1 | D07 | C:C | 0.45757 | 3.76133 |
| 2031012 | Vlasi-1 | E07 | C:C | 0.4243 | 3.60312 |
| 2031012 | Vlasi-1 | F07 | C:A | 1.68867 | 2.49915 |
| 2031012 | Vlasi-1 | G07 | C:A | 1.62622 | 2.56073 |
| 2031012 | Vlasi-1 | H07 | C:A | 1.66549 | 2.57975 |
| 2031012 | Vlasi-1 | A08 | C:C | 0.49896 | 3.73317 |
| 2031012 | Vlasi-1 | B08 | C:A | 1.62604 | 2.65872 |
| 2031012 | Vlasi-1 | C08 | C:A | 1.78166 | 2.53223 |
| 2031012 | Vlasi-1 | D08 | C:C | 0.47194 | 3.59452 |
| 2031012 | Vlasi-1 | E08 | C:C | 0.42232 | 3.52355 |
| 2031012 | Vlasi-1 | F08 | C:A | 1.70128 | 2.6142 |
| 2031012 | Vlasi-1 | G08 | C:C | 0.43036 | 3.52674 |
| 2031012 | Vlasi-1 | H08 | C:A | 1.37305 | 2.23041 |
| 2031012 | Vlasi-1 | A09 | C:C | 0.51973 | 3.49474 |
| 2031012 | Vlasi-1 | B09 | C:A | 1.76754 | 2.57188 |
| 2031012 | Vlasi-1 | C09 | A:A | 3.66086 | 0.49585 |
| 2031012 | Vlasi-1 | D09 | C:A | 1.68739 | 2.60633 |
| 2031012 | Vlasi-1 | E09 | C:A | 1.81768 | 2.56886 |
| 2031012 | Vlasi-1 | F09 | C:C | 0.4333 | 3.54862 |
| 2031012 | Vlasi-1 | G09 | C:A | 1.6767 | 2.60656 |

| | | | | | |
|---|---|---|---|---|---|
| 2031012 | Vlasi-1 | H09 | C:A | 1.70057 | 2.5679 |
| 2031012 | Vlasi-1 | A10 | C:C | 0.5439 | 3.48871 |
| 2031012 | Vlasi-1 | B10 | C:C | 0.43542 | 3.5209 |
| 2031012 | Vlasi-1 | C10 | C:C | 0.41509 | 3.46946 |
| 2031012 | Vlasi-1 | D10 | C:A | 1.71036 | 2.61906 |
| 2031012 | Vlasi-1 | E10 | C:A | 1.76625 | 2.61864 |
| 2031012 | Vlasi-1 | F10 | C:C | 0.41315 | 3.54013 |
| 2031012 | Vlasi-1 | G10 | C:A | 1.73983 | 2.51059 |
| 2031012 | Vlasi-1 | H10 | C:A | 1.74721 | 2.44558 |
| 2031012 | Vlasi-1 | A11 | C:C | 0.49315 | 3.41085 |
| 2031012 | Vlasi-1 | B11 | C:A | 1.75022 | 2.53397 |
| 2031012 | Vlasi-1 | C11 | C:C | 0.46917 | 3.78826 |
| 2031012 | Vlasi-1 | D11 | C:C | 0.50758 | 3.78658 |
| 2031012 | Vlasi-1 | E11 | C:C | 0.41639 | 3.66841 |
| 2031012 | Vlasi-1 | F11 | C:C | 0.44678 | 3.72931 |
| 2031012 | Vlasi-1 | G11 | C:A | 1.76525 | 2.45262 |
| 2031012 | Vlasi-1 | H11 | C:C | 0.41806 | 3.60112 |
| 2031012 | Vlasi-1 | A12 | C:A | 1.9189 | 2.57868 |
| 2031012 | Vlasi-1 | B12 | C:A | 1.91528 | 2.60481 |
| 2031012 | Vlasi-1 | C12 | C:C | 0.45294 | 3.54613 |
| 2031012 | Vlasi-1 | D12 | C:C | 0.43915 | 3.6939 |
| 2031012 | Vlasi-1 | E12 | C:C | 0.43575 | 3.69802 |
| 2031012 | Vlasi-1 | F12 | C:A | 1.87677 | 2.56383 |
| 2031012 | Vlasi-1 | G12 | C:C | 0.46202 | 3.68526 |
| 2031012 | Vlasi-1 | H12 | C:A | 1.88178 | 2.56823 |
| 2031012 | Vlasi-2 | A01 | ? | 0.51167 | 2.33163 |
| 2031012 | Vlasi-2 | B01 | C:A | 1.65722 | 2.41701 |
| 2031012 | Vlasi-2 | C01 | C:C | 0.40697 | 3.4679 |
| 2031012 | Vlasi-2 | D01 | C:A | 1.37345 | 2.09541 |
| 2031012 | Vlasi-2 | E01 | C:C | 0.41914 | 3.83977 |
| 2031012 | Vlasi-2 | F01 | C:A | 1.25053 | 1.9829 |
| 2031012 | Vlasi-2 | G01 | C:C | 0.44609 | 3.56658 |
| 2031012 | Vlasi-2 | H01 | C:C | 0.45168 | 3.76466 |
| 2031012 | Vlasi-2 | A02 | C:C | 0.5592 | 3.51552 |
| 2031012 | Vlasi-2 | B02 | C:C | 0.47252 | 3.23174 |
| 2031012 | Vlasi-2 | C02 | C:C | 0.41118 | 3.52347 |
| 2031012 | Vlasi-2 | D02 | C:C | 0.4571 | 3.71918 |
| 2031012 | Vlasi-2 | E02 | C:C | 0.39487 | 3.56867 |
| 2031012 | Vlasi-2 | F02 | C:A | 1.69552 | 2.48536 |
| 2031012 | Vlasi-2 | G02 | C:C | 0.44622 | 3.82449 |
| 2031012 | Vlasi-2 | H02 | C:A | 1.61769 | 2.52763 |
| 2031012 | Vlasi-2 | A03 | C:C | 0.57857 | 3.39668 |
| 2031012 | Vlasi-2 | B03 | C:A | 1.78071 | 2.38634 |
| 2031012 | Vlasi-2 | C03 | C:A | 2.00928 | 2.47514 |
| 2031012 | Vlasi-2 | D03 | C:A | 1.65524 | 2.46012 |
| 2031012 | Vlasi-2 | E03 | A:A | 3.6259 | 0.51767 |
| 2031012 | Vlasi-2 | F03 | C:C | 0.41549 | 3.6097 |
| 2031012 | Vlasi-2 | G03 | C:C | 0.53253 | 3.48965 |
| 2031012 | Vlasi-2 | H03 | C:C | 0.42114 | 3.61283 |
| 2031012 | Vlasi-2 | A04 | C:C | 0.5833 | 3.51356 |

| | | | | | |
|---|---|---|---|---|---|
| 2031012 | Vlasi-2 | B04 | C:C | 0.43498 | 3.53771 |
| 2031012 | Vlasi-2 | C04 | C:C | 0.42402 | 3.44122 |
| 2031012 | Vlasi-2 | D04 | C:C | 0.41965 | 3.44413 |
| 2031012 | Vlasi-2 | E04 | ? | 0.88186 | 0.43928 |
| 2031012 | Vlasi-2 | F04 | C:A | 1.61338 | 2.5626 |
| 2031012 | Vlasi-2 | G04 | C:A | 1.28246 | 2.60249 |
| 2031012 | Vlasi-2 | H04 | C:A | 1.59137 | 2.64216 |
| 2031012 | Vlasi-2 | A05 | A:A | 3.71143 | 0.63938 |
| 2031012 | Vlasi-2 | B05 | C:A | 1.76109 | 2.48006 |
| 2031012 | Vlasi-2 | C05 | C:C | 0.46364 | 3.82347 |
| 2031012 | Vlasi-2 | D05 | C:A | 1.62051 | 2.45793 |
| 2031012 | Vlasi-2 | E05 | C:A | 1.53649 | 2.44567 |
| 2031012 | Vlasi-2 | F05 | A:A | 3.51522 | 0.45114 |
| 2031012 | Vlasi-2 | G05 | C:A | 1.60974 | 2.44239 |
| 2031012 | Vlasi-2 | H05 | C:C | 0.47211 | 3.94118 |
| 2031012 | Vlasi-2 | A06 | C:A | 1.79275 | 2.5051 |
| 2031012 | Vlasi-2 | B06 | C:C | 0.43194 | 3.65363 |
| 2031012 | Vlasi-2 | C06 | C:C | 0.44362 | 3.63294 |
| 2031012 | Vlasi-2 | D06 | C:C | 0.44466 | 3.77932 |
| 2031012 | Vlasi-2 | E06 | C:C | 0.43151 | 3.65838 |
| 2031012 | Vlasi-2 | F06 | C:C | 0.4441 | 3.76464 |
| 2031012 | Vlasi-2 | G06 | C:A | 1.73147 | 2.53192 |
| 2031012 | Vlasi-2 | H06 | C:C | 0.50244 | 3.60726 |
| 2031012 | Vlasi-2 | A07 | C:A | 1.78231 | 2.6831 |
| 2031012 | Vlasi-2 | B07 | ? | 0.75571 | 0.42391 |
| 2031012 | Vlasi-2 | C07 | C:C | 0.46728 | 3.72889 |
| 2031012 | Vlasi-2 | D07 | C:C | 0.4198 | 3.61479 |
| 2031012 | Vlasi-2 | E07 | C:C | 0.44535 | 3.57936 |
| 2031012 | Vlasi-2 | F07 | C:C | 0.38896 | 3.47057 |
| 2031012 | Vlasi-2 | G07 | C:A | 1.57027 | 2.42126 |
| 2031012 | Vlasi-2 | H07 | C:C | 0.45921 | 3.81922 |
| 2031012 | Vlasi-2 | A08 | C:C | 0.54981 | 3.5379 |
| 2031012 | Vlasi-2 | B08 | C:C | 0.50275 | 3.71769 |
| 2031012 | Vlasi-2 | C08 | A:A | 3.04371 | 0.37141 |
| 2031012 | Vlasi-2 | D08 | C:A | 1.70601 | 2.5749 |
| 2031012 | Vlasi-2 | E08 | C:C | 0.42603 | 3.66045 |
| 2031012 | Vlasi-2 | F08 | C:A | 1.64631 | 2.40061 |
| 2031012 | Vlasi-2 | G08 | C:C | 0.41021 | 3.69887 |
| 2031012 | Vlasi-2 | H08 | C:C | 0.4313 | 3.67293 |
| 2031012 | Vlasi-2 | A09 | C:C | 0.54969 | 3.57926 |
| 2031012 | Vlasi-2 | B09 | A:A | 3.64812 | 0.49912 |
| 2031012 | Vlasi-2 | C09 | ? | 0.71391 | 0.44296 |
| 2031012 | Vlasi-2 | D09 | C:C | 0.45662 | 3.63811 |
| 2031012 | Vlasi-2 | E09 | C:A | 1.58141 | 2.3806 |
| 2031012 | Vlasi-2 | F09 | ? | 0.59523 | 0.41865 |
| 2031012 | Vlasi-2 | G09 | C:A | 1.66912 | 2.51781 |
| 2031012 | Vlasi-2 | H09 | C:C | 0.44118 | 3.66195 |
| 2031012 | Vlasi-2 | A10 | C:C | 0.68951 | 3.4891 |
| 2031012 | Vlasi-2 | B10 | C:C | 0.47912 | 3.54914 |
| 2031012 | Vlasi-2 | C10 | C:C | 0.48887 | 3.6538 |

| | | | | | |
|---|---|---|---|---|---|
| 2031012 | Vlasi-2 | D10 | C:C | 0.47116 | 3.82263 |
| 2031012 | Vlasi-2 | E10 | C:C | 0.46885 | 3.73828 |
| 2031012 | Vlasi-2 | F10 | C:A | 1.66842 | 2.63252 |
| 2031012 | Vlasi-2 | G10 | C:C | 0.54292 | 3.5108 |
| 2031012 | Vlasi-2 | H10 | C:C | 0.46414 | 3.6973 |
| 2031012 | Vlasi-2 | A11 | C:A | 1.911 | 2.55618 |
| 2031012 | Vlasi-2 | B11 | A:A | 3.54786 | 0.47027 |
| 2031012 | Vlasi-2 | C11 | C:C | 0.47225 | 3.49419 |
| 2031012 | Vlasi-2 | D11 | C:C | 0.43494 | 3.56846 |
| 2031012 | Vlasi-2 | E11 | A:A | 3.43545 | 0.60184 |
| 2031012 | Vlasi-2 | F11 | C:C | 0.46874 | 3.85575 |
| 2031012 | Vlasi-2 | G11 | C:A | 1.75065 | 2.60853 |
| 2031012 | Vlasi-2 | H11 | C:A | 1.71508 | 2.7486 |
| 2031012 | Vlasi-2 | A12 | A:A | 3.58948 | 0.60771 |
| 2031012 | Vlasi-2 | B12 | C:C | 0.5051 | 3.56884 |
| 2031012 | Vlasi-2 | C12 | C:A | 1.79335 | 2.65613 |
| 2031012 | Vlasi-2 | D12 | C:C | 0.45622 | 3.39401 |
| 2031012 | Vlasi-2 | E12 | C:A | 1.85982 | 2.62907 |
| 2031012 | Vlasi-2 | F12 | C:C | 0.49107 | 3.54766 |
| 2031012 | Vlasi-2 | G12 | C:C | 0.47331 | 3.63059 |
| 2031012 | Vlasi-2 | H12 | C:A | 1.83129 | 2.76615 |
| 2031012 | Vlasi-3 | A01 | C:C | 0.49836 | 3.64755 |
| 2031012 | Vlasi-3 | B01 | C:A | 1.86206 | 2.61677 |
| 2031012 | Vlasi-3 | C01 | A:A | 3.9924 | 0.45898 |
| 2031012 | Vlasi-3 | D01 | C:A | 1.7784 | 2.64286 |
| 2031012 | Vlasi-3 | E01 | C:C | 0.34708 | 3.619 |
| 2031012 | Vlasi-3 | F01 | C:A | 1.88675 | 2.39593 |
| 2031012 | Vlasi-3 | G01 | C:C | 0.40326 | 3.75095 |
| 2031012 | Vlasi-3 | H01 | C:A | 1.79595 | 2.59154 |
| 2031012 | Vlasi-3 | A02 | C:A | 1.9465 | 2.57217 |
| 2031012 | Vlasi-3 | B02 | C:C | 0.44024 | 3.59044 |
| 2031012 | Vlasi-3 | C02 | C:C | 0.38797 | 3.54214 |
| 2031012 | Vlasi-3 | D02 | C:C | 0.39959 | 3.52387 |
| 2031012 | Vlasi-3 | E02 | C:A | 1.60818 | 2.37766 |
| 2031012 | Vlasi-3 | F02 | C:A | 1.58598 | 2.48294 |
| 2031012 | Vlasi-3 | G02 | C:A | 1.78239 | 2.48373 |
| 2031012 | Vlasi-3 | H02 | C:C | 0.46026 | 3.67512 |
| 2031012 | Vlasi-3 | A03 | C:C | 0.45813 | 3.55928 |
| 2031012 | Vlasi-3 | B03 | C:A | 1.71641 | 2.46573 |
| 2031012 | Vlasi-3 | C03 | A:A | 3.59286 | 0.49021 |
| 2031012 | Vlasi-3 | D03 | C:C | 0.4063 | 3.48102 |
| 2031012 | Vlasi-3 | E03 | C:A | 1.65139 | 2.63376 |
| 2031012 | Vlasi-3 | F03 | C:A | 1.57596 | 2.40504 |
| 2031012 | Vlasi-3 | G03 | A:A | 3.66148 | 0.49247 |
| 2031012 | Vlasi-3 | H03 | C:C | 0.52841 | 3.62256 |
| 2031012 | Vlasi-3 | A04 | A:A | 3.70366 | 0.519 |
| 2031012 | Vlasi-3 | B04 | C:C | 0.43254 | 3.49162 |
| 2031012 | Vlasi-3 | C04 | C:C | 0.43511 | 3.6646 |
| 2031012 | Vlasi-3 | D04 | C:C | 0.40598 | 3.59288 |
| 2031012 | Vlasi-3 | E04 | C:C | 0.43231 | 3.73514 |

| | | | | | |
|---|---|---|---|---|---|
| 2031012 | Vlasi-3 | F04 | C:C | 0.43594 | 3.65711 |
| 2031012 | Vlasi-3 | G04 | C:A | 1.52061 | 2.33052 |
| 2031012 | Vlasi-3 | H04 | C:C | 0.43284 | 3.61518 |
| 2031012 | Vlasi-3 | A05 | C:C | 0.51888 | 3.648 |
| 2031012 | Vlasi-3 | B05 | C:C | 0.4519 | 3.01827 |
| 2031012 | Vlasi-3 | C05 | C:C | 0.41873 | 3.68222 |
| 2031012 | Vlasi-3 | D05 | C:A | 1.52254 | 2.54478 |
| 2031012 | Vlasi-3 | E05 | A:A | 3.71834 | 0.5009 |
| 2031012 | Vlasi-3 | F05 | C:C | 0.44332 | 3.72662 |
| 2031012 | Vlasi-3 | G05 | A:A | 3.63054 | 0.4694 |
| 2031012 | Vlasi-3 | H05 | C:C | 0.49805 | 3.60133 |
| 2031012 | Vlasi-3 | A06 | C:C | 0.48077 | 3.43996 |
| 2031012 | Vlasi-3 | B06 | C:C | 0.4317 | 3.43728 |
| 2031012 | Vlasi-3 | C06 | C:C | 0.43118 | 3.58669 |
| 2031012 | Vlasi-3 | D06 | C:C | 0.42905 | 3.62633 |
| 2031012 | Vlasi-3 | E06 | C:C | 0.43195 | 3.62489 |
| 2031012 | Vlasi-3 | F06 | A:A | 3.60149 | 0.50054 |
| 2031012 | Vlasi-3 | G06 | C:C | 0.43311 | 3.62029 |
| 2031012 | Vlasi-3 | H06 | C:A | 1.69367 | 2.70449 |
| 2031012 | Vlasi-3 | A07 | C:C | 0.44463 | 3.5659 |
| 2031012 | Vlasi-3 | B07 | C:C | 0.41381 | 3.48595 |
| 2031012 | Vlasi-3 | C07 | C:C | 0.45897 | 3.70787 |
| 2031012 | Vlasi-3 | D07 | C:C | 0.40766 | 3.57754 |
| 2031012 | Vlasi-3 | E07 | C:A | 1.61526 | 2.22344 |
| 2031012 | Vlasi-3 | F07 | C:A | 1.59075 | 2.45128 |
| 2031012 | Vlasi-3 | G07 | C:A | 1.72246 | 2.48066 |
| 2031012 | Vlasi-3 | H07 | A:A | 3.53681 | 0.61638 |
| 2031012 | Vlasi-3 | A08 | C:C | 0.43881 | 3.56622 |
| 2031012 | Vlasi-3 | B08 | C:C | 0.42549 | 3.61169 |
| 2031012 | Vlasi-3 | C08 | C:C | 0.40676 | 3.54998 |
| 2031012 | Vlasi-3 | D08 | C:C | 0.43628 | 3.61031 |
| 2031012 | Vlasi-3 | E08 | C:A | 1.74083 | 2.32545 |
| 2031012 | Vlasi-3 | F08 | C:C | 0.42031 | 3.65518 |
| 2031012 | Vlasi-3 | G08 | C:C | 0.41726 | 3.64885 |
| 2031012 | Vlasi-3 | H08 | A:A | 3.99873 | 0.52958 |
| 2031012 | Vlasi-3 | A09 | C:C | 0.48077 | 3.63193 |
| 2031012 | Vlasi-3 | B09 | C:A | 1.73774 | 2.47058 |
| 2031012 | Vlasi-3 | C09 | C:C | 0.42931 | 3.47787 |
| 2031012 | Vlasi-3 | D09 | C:C | 0.40355 | 3.44713 |
| 2031012 | Vlasi-3 | E09 | C:C | 0.39678 | 3.45979 |
| 2031012 | Vlasi-3 | F09 | C:C | 0.41019 | 3.42494 |
| 2031012 | Vlasi-3 | G09 | C:A | 1.66037 | 2.43974 |
| 2031012 | Vlasi-3 | H09 | C:A | 1.81437 | 2.69925 |
| 2031012 | Vlasi-3 | A10 | C:C | 0.51343 | 3.59239 |
| 2031012 | Vlasi-3 | B10 | C:C | 0.41587 | 3.45196 |
| 2031012 | Vlasi-3 | C10 | C:C | 0.44665 | 3.71878 |
| 2031012 | Vlasi-3 | D10 | C:C | 0.40918 | 3.69715 |
| 2031012 | Vlasi-3 | E10 | C:A | 1.87323 | 2.51297 |
| 2031012 | Vlasi-3 | F10 | C:C | 0.44608 | 3.79374 |
| 2031012 | Vlasi-3 | G10 | C:C | 0.45621 | 3.69634 |

| | | | | | |
|---|---|---|---|---|---|
| 2031012 | Vlasi-3 | H10 | A:A | 3.70225 | 0.46877 |
| 2031012 | Vlasi-3 | A11 | C:A | 1.94771 | 2.59988 |
| 2031012 | Vlasi-3 | B11 | C:C | 0.43867 | 3.68208 |
| 2031012 | Vlasi-3 | C11 | C:C | 0.43481 | 3.5169 |
| 2031012 | Vlasi-3 | D11 | C:A | 1.9652 | 2.46264 |
| 2031012 | Vlasi-3 | E11 | C:C | 0.40733 | 3.50551 |
| 2031012 | Vlasi-3 | F11 | C:C | 0.43759 | 3.61106 |
| 2031012 | Vlasi-3 | G11 | C:A | 1.79933 | 2.55143 |
| 2031012 | Vlasi-3 | H11 | C:C | 0.4782 | 3.97764 |
| 2031012 | Vlasi-3 | A12 | C:A | 1.8118 | 2.51689 |
| 2031012 | Vlasi-3 | B12 | C:C | 0.4693 | 3.58394 |
| 2031012 | Vlasi-3 | C12 | C:C | 0.45737 | 3.82167 |
| 2031012 | Vlasi-3 | D12 | C:C | 0.45742 | 3.64234 |
| 2031012 | Vlasi-3 | E12 | C:A | 1.83945 | 2.47364 |
| 2031012 | Vlasi-3 | F12 | C:C | 0.47373 | 3.51933 |
| 2031012 | Vlasi-3 | G12 | C:A | 1.67808 | 2.50185 |
| 2031012 | Vlasi-3 | H12 | C:C | 0.43546 | 3.5103 |
| 2031012 | Vlasi-4 | A01 | ? | 0.60397 | 0.36498 |
| 2031012 | Vlasi-4 | B01 | C:A | 1.70895 | 2.30909 |
| 2031012 | Vlasi-4 | C01 | C:C | 0.39497 | 3.56939 |
| 2031012 | Vlasi-4 | D01 | C:C | 0.40957 | 3.5486 |
| 2031012 | Vlasi-4 | E01 | C:A | 1.34462 | 2.43459 |
| 2031012 | Vlasi-4 | F01 | C:A | 1.69242 | 2.35777 |
| 2031012 | Vlasi-4 | G01 | C:C | 0.42182 | 3.68701 |
| 2031012 | Vlasi-4 | H01 | C:C | 0.42607 | 3.71156 |
| 2031012 | Vlasi-4 | A02 | C:A | 1.86588 | 2.71345 |
| 2031012 | Vlasi-4 | B02 | C:A | 1.93579 | 2.45596 |
| 2031012 | Vlasi-4 | C02 | C:C | 0.46644 | 3.72433 |
| 2031012 | Vlasi-4 | D02 | C:C | 0.47746 | 3.66052 |
| 2031012 | Vlasi-4 | E02 | C:A | 1.59404 | 2.40188 |
| 2031012 | Vlasi-4 | F02 | C:A | 1.5793 | 2.24588 |
| 2031012 | Vlasi-4 | G02 | C:C | 0.41763 | 3.59411 |
| 2031012 | Vlasi-4 | H02 | C:A | 1.71764 | 2.53563 |
| 2031012 | Vlasi-4 | A03 | C:A | 1.99894 | 2.61785 |
| 2031012 | Vlasi-4 | B03 | A:A | 3.61611 | 0.47935 |
| 2031012 | Vlasi-4 | C03 | C:A | 1.6476 | 2.49817 |
| 2031012 | Vlasi-4 | D03 | C:A | 1.61639 | 2.38325 |
| 2031012 | Vlasi-4 | E03 | C:A | 1.45973 | 2.54691 |
| 2031012 | Vlasi-4 | F03 | C:C | 0.3984 | 3.56527 |
| 2031012 | Vlasi-4 | G03 | C:C | 0.41497 | 3.63813 |
| 2031012 | Vlasi-4 | H03 | C:A | 1.59322 | 2.53641 |
| 2031012 | Vlasi-4 | A04 | C:C | 0.47733 | 3.53036 |
| 2031012 | Vlasi-4 | B04 | A:A | 3.47796 | 0.45442 |
| 2031012 | Vlasi-4 | C04 | C:A | 1.67003 | 2.39388 |
| 2031012 | Vlasi-4 | D04 | C:A | 1.5289 | 2.24281 |
| 2031012 | Vlasi-4 | E04 | C:C | 0.42037 | 3.52168 |
| 2031012 | Vlasi-4 | F04 | C:C | 0.41555 | 3.71034 |
| 2031012 | Vlasi-4 | G04 | C:A | 1.55865 | 2.49403 |
| 2031012 | Vlasi-4 | H04 | C:A | 1.70029 | 2.61986 |
| 2031012 | Vlasi-4 | A05 | C:C | 0.46921 | 3.47914 |

| | | | | | |
|---|---|---|---|---|---|
| 2031012 | Vlasi-4 | B05 | C:A | 1.59901 | 2.42658 |
| 2031012 | Vlasi-4 | C05 | C:A | 1.66512 | 2.27876 |
| 2031012 | Vlasi-4 | D05 | C:C | 0.41264 | 3.39499 |
| 2031012 | Vlasi-4 | E05 | C:A | 1.52995 | 2.37868 |
| 2031012 | Vlasi-4 | F05 | C:C | 0.40357 | 3.39923 |
| 2031012 | Vlasi-4 | G05 | C:C | 0.43701 | 3.67549 |
| 2031012 | Vlasi-4 | H05 | A:A | 3.69269 | 0.4843 |
| 2031012 | Vlasi-4 | A06 | C:C | 0.46675 | 3.40949 |
| 2031012 | Vlasi-4 | B06 | C:C | 0.39997 | 3.24073 |
| 2031012 | Vlasi-4 | C06 | C:A | 1.70117 | 2.47211 |
| 2031012 | Vlasi-4 | D06 | C:A | 1.519 | 2.23162 |
| 2031012 | Vlasi-4 | E06 | C:C | 0.4059 | 3.50348 |
| 2031012 | Vlasi-4 | F06 | C:C | 0.41569 | 3.42283 |
| 2031012 | Vlasi-4 | G06 | C:A | 1.59004 | 2.39966 |
| 2031012 | Vlasi-4 | H06 | C:C | 0.43617 | 3.53633 |
| 2031012 | Vlasi-4 | A07 | ? | 0.57934 | 1.20726 |
| 2031012 | Vlasi-4 | B07 | ? | 1.37049 | 0.43374 |
| 2031012 | Vlasi-4 | C07 | ? | 0.51777 | 0.45981 |
| 2031012 | Vlasi-4 | D07 | ? | 0.60713 | 0.52078 |
| 2031012 | Vlasi-4 | E07 | ? | 0.60593 | 0.48665 |
| 2031012 | Vlasi-4 | F07 | A:A | 3.68991 | 0.54614 |
| 2031012 | Vlasi-4 | G07 | A:A | 3.71102 | 0.53656 |
| 2031012 | Vlasi-4 | H07 | A:A | 3.88293 | 0.61223 |
| 2031012 | Vlasi-4 | A08 | A:A | 3.66155 | 0.59661 |
| 2031012 | Vlasi-4 | B08 | A:A | 3.52749 | 0.5226 |
| 2031012 | Vlasi-4 | C08 | A:A | 3.88133 | 0.58724 |
| 2031012 | Vlasi-4 | D08 | A:A | 3.70321 | 0.54115 |
| 2031012 | Vlasi-4 | E08 | A:A | 3.71776 | 0.5852 |
| 2031012 | Vlasi-4 | F08 | A:A | 3.77719 | 0.59877 |
| 2031012 | Vlasi-4 | G08 | A:A | 3.60786 | 0.75566 |
| 2031012 | Vlasi-4 | H08 | A:A | 3.8258 | 0.58765 |
| 2031012 | Vlasi-4 | A09 | A:A | 3.73177 | 0.62797 |
| 2031012 | Vlasi-4 | B09 | A:A | 3.60035 | 0.57428 |
| 2031012 | Vlasi-4 | C09 | A:A | 3.55549 | 0.58728 |
| 2031012 | Vlasi-4 | D09 | A:A | 3.75175 | 0.57822 |
| 2031012 | Vlasi-4 | E09 | A:A | 3.71354 | 0.57404 |
| 2031012 | Vlasi-4 | F09 | A:A | 3.81024 | 0.57006 |
| 2031012 | Vlasi-4 | G09 | A:A | 3.82207 | 0.49433 |
| 2031012 | Vlasi-4 | H09 | A:A | 3.87008 | 0.60636 |
| 2031012 | Vlasi-4 | A10 | A:A | 3.66076 | 0.65049 |
| 2031012 | Vlasi-4 | B10 | A:A | 3.89637 | 0.57152 |
| 2031012 | Vlasi-4 | C10 | C:C | 0.45438 | 3.55953 |
| 2031012 | Vlasi-4 | D10 | C:C | 0.38867 | 3.54175 |
| 2031012 | Vlasi-4 | E10 | C:C | 0.4443 | 3.65593 |
| 2031012 | Vlasi-4 | F10 | C:C | 0.46185 | 3.77908 |
| 2031012 | Vlasi-4 | G10 | C:C | 0.46316 | 3.73269 |
| 2031012 | Vlasi-4 | H10 | C:C | 0.45571 | 3.76102 |
| 2031012 | Vlasi-4 | A11 | C:C | 0.51181 | 3.58996 |
| 2031012 | Vlasi-4 | B11 | C:C | 0.52173 | 3.66431 |
| 2031012 | Vlasi-4 | C11 | C:C | 0.4519 | 3.43132 |

| | | | | | |
|---|---|---|---|---|---|
| 2031012 | Vlasi-4 | D11 | C:C | 0.45135 | 3.66587 |
| 2031012 | Vlasi-4 | E11 | C:C | 0.46441 | 3.57254 |
| 2031012 | Vlasi-4 | F11 | C:C | 0.46001 | 3.69277 |
| 2031012 | Vlasi-4 | G11 | C:C | 0.46858 | 3.69444 |
| 2031012 | Vlasi-4 | H11 | C:C | 0.62935 | 3.70298 |
| 2031012 | Vlasi-4 | A12 | C:C | 0.55662 | 3.519 |
| 2031012 | Vlasi-4 | B12 | C:C | 0.50797 | 3.49759 |
| 2031012 | Vlasi-4 | C12 | C:C | 0.66903 | 3.48407 |
| 2031012 | Vlasi-4 | D12 | C:C | 0.51032 | 3.61261 |
| 2031012 | Vlasi-4 | E12 | C:C | 0.50524 | 3.64116 |
| 2031012 | Vlasi-4 | F12 | C:C | 0.5135 | 3.5853 |
| 2031012 | Vlasi-4 | G12 | C:C | 0.49865 | 3.67703 |
| 2031012 | Vlasi-4 | H12 | NTC | 0.97759 | 0.52327 |
| 2031012 | Vlasi-1 | A01 | G:A | 2.24912 | 2.55585 |
| 2031012 | Vlasi-1 | B01 | G:G | 0.36332 | 3.77553 |
| 2031012 | Vlasi-1 | C01 | G:G | 0.33211 | 3.62727 |
| 2031012 | Vlasi-1 | D01 | G:G | 0.36347 | 3.84397 |
| 2031012 | Vlasi-1 | E01 | G:G | 0.35795 | 3.91474 |
| 2031012 | Vlasi-1 | F01 | G:A | 2.07129 | 2.44436 |
| 2031012 | Vlasi-1 | G01 | G:G | 0.3736 | 3.8365 |
| 2031012 | Vlasi-1 | H01 | G:G | 0.37816 | 3.77193 |
| 2031012 | Vlasi-1 | A02 | G:A | 2.30039 | 2.45514 |
| 2031012 | Vlasi-1 | B02 | G:G | 0.40539 | 3.58506 |
| 2031012 | Vlasi-1 | C02 | G:A | 1.99916 | 2.39759 |
| 2031012 | Vlasi-1 | D02 | G:G | 0.41161 | 3.80681 |
| 2031012 | Vlasi-1 | E02 | G:G | 0.39436 | 3.59096 |
| 2031012 | Vlasi-1 | F02 | G:G | 0.39727 | 3.65334 |
| 2031012 | Vlasi-1 | G02 | G:G | 0.40106 | 3.43038 |
| 2031012 | Vlasi-1 | H02 | G:G | 0.4024 | 3.64356 |
| 2031012 | Vlasi-1 | A03 | G:A | 2.16655 | 2.4848 |
| 2031012 | Vlasi-1 | B03 | G:G | 0.42271 | 3.66049 |
| 2031012 | Vlasi-1 | C03 | G:A | 2.07259 | 2.42178 |
| 2031012 | Vlasi-1 | D03 | G:A | 2.03841 | 2.44702 |
| 2031012 | Vlasi-1 | E03 | G:G | 0.40258 | 3.69616 |
| 2031012 | Vlasi-1 | F03 | G:G | 0.39669 | 3.46207 |
| 2031012 | Vlasi-1 | G03 | A:A | 3.78678 | 0.46523 |
| 2031012 | Vlasi-1 | H03 | G:G | 0.4058 | 3.70752 |
| 2031012 | Vlasi-1 | A04 | G:G | 0.49567 | 3.61224 |
| 2031012 | Vlasi-1 | B04 | G:A | 2.21456 | 2.51706 |
| 2031012 | Vlasi-1 | C04 | G:G | 0.41527 | 3.65552 |
| 2031012 | Vlasi-1 | D04 | G:G | 0.42431 | 3.70994 |
| 2031012 | Vlasi-1 | E04 | G:A | 1.99849 | 2.43983 |
| 2031012 | Vlasi-1 | F04 | G:G | 0.44283 | 3.81403 |
| 2031012 | Vlasi-1 | G04 | G:A | 2.05234 | 2.45315 |
| 2031012 | Vlasi-1 | H04 | G:G | 0.42509 | 3.78817 |
| 2031012 | Vlasi-1 | A05 | G:A | 2.10445 | 2.51858 |
| 2031012 | Vlasi-1 | B05 | G:G | 0.38051 | 3.4865 |
| 2031012 | Vlasi-1 | C05 | G:A | 2.08713 | 2.55709 |
| 2031012 | Vlasi-1 | D05 | G:G | 0.41028 | 3.75743 |
| 2031012 | Vlasi-1 | E05 | G:G | 0.39264 | 3.53198 |

| | | | | | |
|---|---|---|---|---|---|
| 2031012 | Vlasi-1 | F05 | A:A | 3.59931 | 0.47888 |
| 2031012 | Vlasi-1 | G05 | G:G | 0.44341 | 3.66459 |
| 2031012 | Vlasi-1 | H05 | G:G | 0.41009 | 3.66605 |
| 2031012 | Vlasi-1 | A06 | G:G | 0.47093 | 3.47538 |
| 2031012 | Vlasi-1 | B06 | G:G | 0.3915 | 3.48952 |
| 2031012 | Vlasi-1 | C06 | G:A | 2.03194 | 2.4625 |
| 2031012 | Vlasi-1 | D06 | G:G | 0.44193 | 3.84065 |
| 2031012 | Vlasi-1 | E06 | G:A | 1.93368 | 2.38084 |
| 2031012 | Vlasi-1 | F06 | G:A | 2.10501 | 2.54369 |
| 2031012 | Vlasi-1 | G06 | G:A | 1.98419 | 2.52894 |
| 2031012 | Vlasi-1 | H06 | G:G | 0.40747 | 3.67842 |
| 2031012 | Vlasi-1 | A07 | G:G | 0.45454 | 3.48796 |
| 2031012 | Vlasi-1 | B07 | A:A | 3.67438 | 0.41206 |
| 2031012 | Vlasi-1 | C07 | G:G | 0.43215 | 3.63472 |
| 2031012 | Vlasi-1 | D07 | G:A | 2.0161 | 2.57519 |
| 2031012 | Vlasi-1 | E07 | G:A | 2.00472 | 2.33824 |
| 2031012 | Vlasi-1 | F07 | G:A | 1.94053 | 2.51281 |
| 2031012 | Vlasi-1 | G07 | G:A | 1.96297 | 2.54329 |
| 2031012 | Vlasi-1 | H07 | G:A | 2.14269 | 2.40978 |
| 2031012 | Vlasi-1 | A08 | G:A | 2.10335 | 2.61167 |
| 2031012 | Vlasi-1 | B08 | G:A | 2.10468 | 2.35246 |
| 2031012 | Vlasi-1 | C08 | G:G | 0.4205 | 3.62351 |
| 2031012 | Vlasi-1 | D08 | G:A | 2.01138 | 2.4732 |
| 2031012 | Vlasi-1 | E08 | G:A | 2.03033 | 2.4954 |
| 2031012 | Vlasi-1 | F08 | G:G | 0.40198 | 3.46472 |
| 2031012 | Vlasi-1 | G08 | G:A | 1.99158 | 2.47364 |
| 2031012 | Vlasi-1 | H08 | G:G | 0.38112 | 3.53395 |
| 2031012 | Vlasi-1 | A09 | G:A | 2.26581 | 2.56256 |
| 2031012 | Vlasi-1 | B09 | G:G | 0.42499 | 3.75882 |
| 2031012 | Vlasi-1 | C09 | G:A | 2.00949 | 2.65398 |
| 2031012 | Vlasi-1 | D09 | G:A | 2.10117 | 2.56968 |
| 2031012 | Vlasi-1 | E09 | G:G | 0.40313 | 3.73911 |
| 2031012 | Vlasi-1 | F09 | G:A | 2.0174 | 2.48639 |
| 2031012 | Vlasi-1 | G09 | G:A | 2.00619 | 2.57636 |
| 2031012 | Vlasi-1 | H09 | G:G | 0.4198 | 3.84489 |
| 2031012 | Vlasi-1 | A10 | G:A | 2.14179 | 2.64098 |
| 2031012 | Vlasi-1 | B10 | G:G | 0.394 | 3.416 |
| 2031012 | Vlasi-1 | C10 | G:A | 2.09714 | 2.51407 |
| 2031012 | Vlasi-1 | D10 | G:G | 0.38645 | 3.53407 |
| 2031012 | Vlasi-1 | E10 | G:A | 2.07623 | 2.50011 |
| 2031012 | Vlasi-1 | F10 | G:A | 1.74242 | 2.3585 |
| 2031012 | Vlasi-1 | G10 | G:A | 2.11147 | 2.59517 |
| 2031012 | Vlasi-1 | H10 | G:A | 2.00997 | 2.41876 |
| 2031012 | Vlasi-1 | A11 | A:A | 3.56858 | 0.48148 |
| 2031012 | Vlasi-1 | B11 | G:A | 2.10225 | 2.43969 |
| 2031012 | Vlasi-1 | C11 | G:G | 0.40731 | 3.57329 |
| 2031012 | Vlasi-1 | D11 | G:G | 0.40139 | 3.63581 |
| 2031012 | Vlasi-1 | E11 | G:A | 2.11976 | 2.46191 |
| 2031012 | Vlasi-1 | F11 | G:A | 2.03348 | 2.57584 |
| 2031012 | Vlasi-1 | G11 | G:A | 2.13681 | 2.52224 |

| | | | | | |
|---|---|---|---|---|---|
| 2031012 | Vlasi-1 | H11 | G:G | 0.41029 | 3.83289 |
| 2031012 | Vlasi-1 | A12 | G:G | 0.47742 | 3.59989 |
| 2031012 | Vlasi-1 | B12 | G:A | 2.14498 | 2.54063 |
| 2031012 | Vlasi-1 | C12 | G:G | 0.43026 | 3.62845 |
| 2031012 | Vlasi-1 | D12 | G:G | 0.40426 | 3.60046 |
| 2031012 | Vlasi-1 | E12 | G:G | 0.52176 | 3.62904 |
| 2031012 | Vlasi-1 | F12 | G:G | 0.43518 | 3.71448 |
| 2031012 | Vlasi-1 | G12 | G:G | 0.40459 | 3.72148 |
| 2031012 | Vlasi-1 | H12 | G:G | 0.42313 | 3.64419 |
| 2031012 | Vlasi-2 | A01 | G:A | 2.19659 | 2.51894 |
| 2031012 | Vlasi-2 | B01 | G:A | 2.05404 | 2.34276 |
| 2031012 | Vlasi-2 | C01 | G:A | 1.96694 | 2.35573 |
| 2031012 | Vlasi-2 | D01 | G:A | 1.96909 | 2.32515 |
| 2031012 | Vlasi-2 | E01 | G:A | 1.9897 | 2.60185 |
| 2031012 | Vlasi-2 | F01 | G:G | 0.42678 | 3.60545 |
| 2031012 | Vlasi-2 | G01 | G:A | 2.05656 | 2.48063 |
| 2031012 | Vlasi-2 | H01 | G:A | 2.18934 | 2.65941 |
| 2031012 | Vlasi-2 | A02 | G:G | 0.50049 | 3.58111 |
| 2031012 | Vlasi-2 | B02 | G:A | 2.0538 | 2.35269 |
| 2031012 | Vlasi-2 | C02 | G:G | 0.39179 | 3.50398 |
| 2031012 | Vlasi-2 | D02 | G:G | 0.4195 | 3.69509 |
| 2031012 | Vlasi-2 | E02 | G:G | 0.40246 | 3.56162 |
| 2031012 | Vlasi-2 | F02 | A:A | 3.75045 | 0.5125 |
| 2031012 | Vlasi-2 | G02 | G:A | 1.96481 | 2.52539 |
| 2031012 | Vlasi-2 | H02 | G:G | 0.44465 | 3.85707 |
| 2031012 | Vlasi-2 | A03 | G:G | 0.54629 | 3.48285 |
| 2031012 | Vlasi-2 | B03 | G:G | 0.40535 | 3.48941 |
| 2031012 | Vlasi-2 | C03 | G:A | 2.07717 | 2.51996 |
| 2031012 | Vlasi-2 | D03 | G:A | 2.0592 | 2.46473 |
| 2031012 | Vlasi-2 | E03 | G:G | 0.3937 | 3.57432 |
| 2031012 | Vlasi-2 | F03 | G:A | 1.92394 | 2.38162 |
| 2031012 | Vlasi-2 | G03 | G:A | 1.96582 | 2.46022 |
| 2031012 | Vlasi-2 | H03 | G:A | 2.06708 | 2.51795 |
| 2031012 | Vlasi-2 | A04 | G:G | 0.52926 | 3.73857 |
| 2031012 | Vlasi-2 | B04 | G:G | 0.45375 | 3.80465 |
| 2031012 | Vlasi-2 | C04 | A:A | 3.56379 | 0.43923 |
| 2031012 | Vlasi-2 | D04 | G:A | 2.06617 | 2.46542 |
| 2031012 | Vlasi-2 | E04 | G:A | 1.90589 | 2.26355 |
| 2031012 | Vlasi-2 | F04 | G:A | 1.98103 | 2.52983 |
| 2031012 | Vlasi-2 | G04 | G:A | 1.84212 | 2.39755 |
| 2031012 | Vlasi-2 | H04 | G:G | 0.43479 | 3.8736 |
| 2031012 | Vlasi-2 | A05 | G:G | 0.47605 | 3.46626 |
| 2031012 | Vlasi-2 | B05 | G:G | 0.4064 | 3.46018 |
| 2031012 | Vlasi-2 | C05 | G:A | 1.91431 | 2.39951 |
| 2031012 | Vlasi-2 | D05 | G:A | 2.05947 | 2.51007 |
| 2031012 | Vlasi-2 | E05 | G:G | 0.40588 | 3.65285 |
| 2031012 | Vlasi-2 | F05 | A:A | 3.42655 | 0.4649 |
| 2031012 | Vlasi-2 | G05 | A:A | 3.71665 | 0.49909 |
| 2031012 | Vlasi-2 | H05 | G:G | 0.44689 | 3.78778 |
| 2031012 | Vlasi-2 | A06 | G:G | 0.48753 | 3.5048 |

| | | | | | |
|---|---|---|---|---|---|
| 2031012 | Vlasi-2 | B06 | G:A | 2.09521 | 2.58609 |
| 2031012 | Vlasi-2 | C06 | A:A | 3.80444 | 0.609 |
| 2031012 | Vlasi-2 | D06 | G:G | 0.39916 | 3.62435 |
| 2031012 | Vlasi-2 | E06 | G:G | 0.37213 | 3.48397 |
| 2031012 | Vlasi-2 | F06 | G:G | 0.40759 | 3.64357 |
| 2031012 | Vlasi-2 | G06 | G:G | 0.4279 | 3.72318 |
| 2031012 | Vlasi-2 | H06 | G:G | 0.40633 | 3.70686 |
| 2031012 | Vlasi-2 | A07 | G:A | 2.03731 | 2.50552 |
| 2031012 | Vlasi-2 | B07 | G:A | 1.98244 | 2.48336 |
| 2031012 | Vlasi-2 | C07 | G:G | 0.40396 | 3.5858 |
| 2031012 | Vlasi-2 | D07 | G:G | 0.41845 | 3.78786 |
| 2031012 | Vlasi-2 | E07 | A:A | 3.67523 | 0.50773 |
| 2031012 | Vlasi-2 | F07 | G:G | 0.3838 | 3.61952 |
| 2031012 | Vlasi-2 | G07 | G:A | 2.0546 | 2.58161 |
| 2031012 | Vlasi-2 | H07 | G:G | 0.41961 | 3.83444 |
| 2031012 | Vlasi-2 | A08 | G:G | 0.4797 | 3.43893 |
| 2031012 | Vlasi-2 | B08 | G:A | 2.16677 | 2.61532 |
| 2031012 | Vlasi-2 | C08 | G:G | 0.46872 | 3.46467 |
| 2031012 | Vlasi-2 | D08 | G:A | 2.10516 | 2.42565 |
| 2031012 | Vlasi-2 | E08 | G:A | 2.01406 | 2.34438 |
| 2031012 | Vlasi-2 | F08 | G:A | 2.0599 | 2.41997 |
| 2031012 | Vlasi-2 | G08 | G:G | 0.40994 | 3.65291 |
| 2031012 | Vlasi-2 | H08 | G:A | 2.03265 | 2.35049 |
| 2031012 | Vlasi-2 | A09 | G:A | 2.0949 | 2.53886 |
| 2031012 | Vlasi-2 | B09 | G:A | 2.10296 | 2.38291 |
| 2031012 | Vlasi-2 | C09 | A:A | 3.22058 | 0.41088 |
| 2031012 | Vlasi-2 | D09 | G:G | 0.40674 | 3.64264 |
| 2031012 | Vlasi-2 | E09 | G:A | 2.20109 | 2.54928 |
| 2031012 | Vlasi-2 | F09 | G:G | 0.44152 | 3.0636 |
| 2031012 | Vlasi-2 | G09 | A:A | 3.83492 | 0.48198 |
| 2031012 | Vlasi-2 | H09 | A:A | 3.94117 | 0.51384 |
| 2031012 | Vlasi-2 | A10 | A:A | 3.49821 | 0.90348 |
| 2031012 | Vlasi-2 | B10 | G:A | 1.95701 | 2.3916 |
| 2031012 | Vlasi-2 | C10 | G:G | 0.44742 | 3.72316 |
| 2031012 | Vlasi-2 | D10 | G:A | 2.08069 | 2.51817 |
| 2031012 | Vlasi-2 | E10 | G:G | 0.43736 | 3.50384 |
| 2031012 | Vlasi-2 | F10 | G:A | 2.15965 | 2.53733 |
| 2031012 | Vlasi-2 | G10 | G:G | 0.42882 | 3.70266 |
| 2031012 | Vlasi-2 | H10 | G:G | 0.50435 | 3.95211 |
| 2031012 | Vlasi-2 | A11 | G:G | 0.50691 | 3.4506 |
| 2031012 | Vlasi-2 | B11 | G:A | 2.11604 | 2.49235 |
| 2031012 | Vlasi-2 | C11 | G:G | 0.42027 | 3.69332 |
| 2031012 | Vlasi-2 | D11 | G:G | 0.41108 | 3.70168 |
| 2031012 | Vlasi-2 | E11 | G:A | 2.21558 | 2.53154 |
| 2031012 | Vlasi-2 | F11 | G:G | 0.53228 | 3.70373 |
| 2031012 | Vlasi-2 | G11 | G:G | 0.41601 | 3.73737 |
| 2031012 | Vlasi-2 | H11 | A:A | 4.08509 | 0.52306 |
| 2031012 | Vlasi-2 | A12 | G:A | 2.24784 | 2.51452 |
| 2031012 | Vlasi-2 | B12 | G:A | 2.18566 | 2.50303 |
| 2031012 | Vlasi-2 | C12 | G:A | 2.26087 | 2.64757 |

| | | | | | |
|---|---|---|---|---|---|
| 2031012 | Vlasi-2 | D12 | G:A | 2.06838 | 2.68373 |
| 2031012 | Vlasi-2 | E12 | G:G | 0.47244 | 3.80944 |
| 2031012 | Vlasi-2 | F12 | G:G | 0.47289 | 3.61749 |
| 2031012 | Vlasi-2 | G12 | G:G | 0.45543 | 3.61648 |
| 2031012 | Vlasi-2 | H12 | G:A | 2.25177 | 2.60399 |
| 2031012 | Vlasi-3 | A01 | G:A | 1.86426 | 1.96072 |
| 2031012 | Vlasi-3 | B01 | G:G | 0.35248 | 3.61424 |
| 2031012 | Vlasi-3 | C01 | G:G | 0.34333 | 3.61132 |
| 2031012 | Vlasi-3 | D01 | G:A | 2.15321 | 2.56134 |
| 2031012 | Vlasi-3 | E01 | G:A | 2.13978 | 2.58617 |
| 2031012 | Vlasi-3 | F01 | G:A | 2.18158 | 2.66077 |
| 2031012 | Vlasi-3 | G01 | A:A | 3.8375 | 0.46322 |
| 2031012 | Vlasi-3 | H01 | G:A | 2.34574 | 2.74557 |
| 2031012 | Vlasi-3 | A02 | G:G | 0.45743 | 3.49245 |
| 2031012 | Vlasi-3 | B02 | G:G | 0.42371 | 3.61063 |
| 2031012 | Vlasi-3 | C02 | G:G | 0.39777 | 3.5854 |
| 2031012 | Vlasi-3 | D02 | G:A | 1.95598 | 2.4505 |
| 2031012 | Vlasi-3 | E02 | G:G | 0.3921 | 3.65724 |
| 2031012 | Vlasi-3 | F02 | G:G | 0.40673 | 3.58217 |
| 2031012 | Vlasi-3 | G02 | G:A | 1.97581 | 2.49341 |
| 2031012 | Vlasi-3 | H02 | G:G | 0.41107 | 3.5384 |
| 2031012 | Vlasi-3 | A03 | G:G | 0.42604 | 3.63731 |
| 2031012 | Vlasi-3 | B03 | A:A | 3.65477 | 0.47833 |
| 2031012 | Vlasi-3 | C03 | G:G | 0.51735 | 3.44216 |
| 2031012 | Vlasi-3 | D03 | A:A | 3.76777 | 0.47543 |
| 2031012 | Vlasi-3 | E03 | G:G | 0.38156 | 3.52191 |
| 2031012 | Vlasi-3 | F03 | G:A | 1.95005 | 2.46016 |
| 2031012 | Vlasi-3 | G03 | G:G | 0.41709 | 3.72954 |
| 2031012 | Vlasi-3 | H03 | G:A | 2.05879 | 2.40523 |
| 2031012 | Vlasi-3 | A04 | G:G | 0.42399 | 3.50322 |
| 2031012 | Vlasi-3 | B04 | G:G | 0.41049 | 3.63605 |
| 2031012 | Vlasi-3 | C04 | G:G | 0.41203 | 3.67204 |
| 2031012 | Vlasi-3 | D04 | G:G | 0.41012 | 3.73748 |
| 2031012 | Vlasi-3 | E04 | G:G | 0.38711 | 3.52254 |
| 2031012 | Vlasi-3 | F04 | G:A | 2.07794 | 2.3486 |
| 2031012 | Vlasi-3 | G04 | G:G | 0.41078 | 3.72422 |
| 2031012 | Vlasi-3 | H04 | G:A | 2.18702 | 2.695 |
| 2031012 | Vlasi-3 | A05 | G:A | 2.18584 | 2.50472 |
| 2031012 | Vlasi-3 | B05 | G:G | 0.39612 | 3.50692 |
| 2031012 | Vlasi-3 | C05 | G:A | 1.8572 | 2.56642 |
| 2031012 | Vlasi-3 | D05 | G:G | 0.44138 | 3.76206 |
| 2031012 | Vlasi-3 | E05 | G:A | 1.92787 | 2.51642 |
| 2031012 | Vlasi-3 | F05 | G:A | 2.11498 | 2.40821 |
| 2031012 | Vlasi-3 | G05 | G:A | 2.04764 | 2.46204 |
| 2031012 | Vlasi-3 | H05 | G:A | 2.11807 | 2.54082 |
| 2031012 | Vlasi-3 | A06 | G:G | 0.42965 | 3.58215 |
| 2031012 | Vlasi-3 | B06 | G:A | 1.87672 | 2.12571 |
| 2031012 | Vlasi-3 | C06 | G:A | 2.00978 | 2.32797 |
| 2031012 | Vlasi-3 | D06 | G:A | 1.96553 | 2.4701 |
| 2031012 | Vlasi-3 | E06 | A:A | 3.67967 | 0.44436 |

| | | | | | |
|---|---|---|---|---|---|
| 2031012 | Vlasi-3 | F06 | A:A | 3.69612 | 0.46407 |
| 2031012 | Vlasi-3 | G06 | G:G | 0.39488 | 3.57175 |
| 2031012 | Vlasi-3 | H06 | G:A | 2.1346 | 2.63027 |
| 2031012 | Vlasi-3 | A07 | G:A | 2.03972 | 2.48048 |
| 2031012 | Vlasi-3 | B07 | G:G | 0.39473 | 3.60352 |
| 2031012 | Vlasi-3 | C07 | G:A | 2.00783 | 2.3287 |
| 2031012 | Vlasi-3 | D07 | G:G | 0.40813 | 3.65928 |
| 2031012 | Vlasi-3 | E07 | G:G | 0.40006 | 3.57526 |
| 2031012 | Vlasi-3 | F07 | G:G | 0.41601 | 3.71038 |
| 2031012 | Vlasi-3 | G07 | A:A | 3.70315 | 0.52545 |
| 2031012 | Vlasi-3 | H07 | G:A | 2.09134 | 2.65146 |
| 2031012 | Vlasi-3 | A08 | G:G | 0.44791 | 3.61459 |
| 2031012 | Vlasi-3 | B08 | G:A | 2.14233 | 2.44917 |
| 2031012 | Vlasi-3 | C08 | A:A | 3.52055 | 0.48301 |
| 2031012 | Vlasi-3 | D08 | G:G | 0.41124 | 3.69545 |
| 2031012 | Vlasi-3 | E08 | G:G | 0.38864 | 3.47421 |
| 2031012 | Vlasi-3 | F08 | G:A | 2.05465 | 2.42635 |
| 2031012 | Vlasi-3 | G08 | G:G | 0.42691 | 3.66464 |
| 2031012 | Vlasi-3 | H08 | G:G | 0.42575 | 3.79491 |
| 2031012 | Vlasi-3 | A09 | G:A | 2.0191 | 2.4628 |
| 2031012 | Vlasi-3 | B09 | A:A | 3.68118 | 0.46837 |
| 2031012 | Vlasi-3 | C09 | G:G | 0.39521 | 3.49169 |
| 2031012 | Vlasi-3 | D09 | G:A | 2.10807 | 2.37671 |
| 2031012 | Vlasi-3 | E09 | G:G | 0.38742 | 3.56257 |
| 2031012 | Vlasi-3 | F09 | G:G | 0.41006 | 3.70146 |
| 2031012 | Vlasi-3 | G09 | A:A | 3.75673 | 0.54889 |
| 2031012 | Vlasi-3 | H09 | G:A | 2.30346 | 2.77913 |
| 2031012 | Vlasi-3 | A10 | G:A | 2.01097 | 2.55568 |
| 2031012 | Vlasi-3 | B10 | G:G | 0.42886 | 3.74822 |
| 2031012 | Vlasi-3 | C10 | G:G | 0.41924 | 3.7654 |
| 2031012 | Vlasi-3 | D10 | G:A | 1.98216 | 2.42837 |
| 2031012 | Vlasi-3 | E10 | G:G | 0.39463 | 3.55746 |
| 2031012 | Vlasi-3 | F10 | G:G | 0.42886 | 3.74216 |
| 2031012 | Vlasi-3 | G10 | G:G | 0.40894 | 3.55597 |
| 2031012 | Vlasi-3 | H10 | G:G | 0.46127 | 3.82931 |
| 2031012 | Vlasi-3 | A11 | G:G | 0.43181 | 3.64234 |
| 2031012 | Vlasi-3 | B11 | G:G | 0.4191 | 3.63858 |
| 2031012 | Vlasi-3 | C11 | G:G | 0.40257 | 3.711 |
| 2031012 | Vlasi-3 | D11 | G:G | 0.42812 | 3.61656 |
| 2031012 | Vlasi-3 | E11 | G:A | 2.07628 | 2.64234 |
| 2031012 | Vlasi-3 | F11 | G:G | 0.40117 | 3.5606 |
| 2031012 | Vlasi-3 | G11 | A:A | 3.88109 | 0.52053 |
| 2031012 | Vlasi-3 | H11 | G:A | 2.22522 | 2.47109 |
| 2031012 | Vlasi-3 | A12 | G:G | 0.45664 | 3.16311 |
| 2031012 | Vlasi-3 | B12 | G:G | 0.43998 | 3.58909 |
| 2031012 | Vlasi-3 | C12 | G:G | 0.41915 | 3.73079 |
| 2031012 | Vlasi-3 | D12 | G:G | 0.42463 | 3.7395 |
| 2031012 | Vlasi-3 | E12 | A:A | 3.96582 | 0.56331 |
| 2031012 | Vlasi-3 | F12 | G:G | 0.47349 | 3.54407 |
| 2031012 | Vlasi-3 | G12 | G:G | 0.86301 | 3.43459 |

| | | | | | |
|---|---|---|---|---|---|
| 2031012 | Vlasi-3 | H12 | G:G | 0.46692 | 3.67907 |
| 2031012 | Vlasi-4 | A01 | G:A | 2.0866 | 2.43228 |
| 2031012 | Vlasi-4 | B01 | G:G | 0.37782 | 3.41406 |
| 2031012 | Vlasi-4 | C01 | G:A | 1.9681 | 2.19565 |
| 2031012 | Vlasi-4 | D01 | G:A | 2.08878 | 2.34541 |
| 2031012 | Vlasi-4 | E01 | G:G | 0.38553 | 3.46588 |
| 2031012 | Vlasi-4 | F01 | G:A | 2.07473 | 2.30964 |
| 2031012 | Vlasi-4 | G01 | G:A | 2.11188 | 2.42194 |
| 2031012 | Vlasi-4 | H01 | A:A | 3.81801 | 0.50151 |
| 2031012 | Vlasi-4 | A02 | G:G | 0.42277 | 3.54238 |
| 2031012 | Vlasi-4 | B02 | G:A | 2.11082 | 2.43041 |
| 2031012 | Vlasi-4 | C02 | G:A | 1.94555 | 2.43513 |
| 2031012 | Vlasi-4 | D02 | G:G | 0.43017 | 3.62161 |
| 2031012 | Vlasi-4 | E02 | G:G | 0.37986 | 3.46412 |
| 2031012 | Vlasi-4 | F02 | G:A | 1.8946 | 2.4019 |
| 2031012 | Vlasi-4 | G02 | A:A | 3.70628 | 0.50469 |
| 2031012 | Vlasi-4 | H02 | G:A | 2.25176 | 2.63603 |
| 2031012 | Vlasi-4 | A03 | G:A | 2.19569 | 2.65328 |
| 2031012 | Vlasi-4 | B03 | G:G | 0.41022 | 3.68104 |
| 2031012 | Vlasi-4 | C03 | G:A | 1.95639 | 2.3457 |
| 2031012 | Vlasi-4 | D03 | G:A | 2.00426 | 2.42079 |
| 2031012 | Vlasi-4 | E03 | G:A | 1.95153 | 2.31315 |
| 2031012 | Vlasi-4 | F03 | G:A | 1.98837 | 2.47425 |
| 2031012 | Vlasi-4 | G03 | G:G | 0.40762 | 3.64618 |
| 2031012 | Vlasi-4 | H03 | G:A | 2.10214 | 2.43835 |
| 2031012 | Vlasi-4 | A04 | G:G | 0.43179 | 3.62128 |
| 2031012 | Vlasi-4 | B04 | G:A | 1.76473 | 2.15133 |
| 2031012 | Vlasi-4 | C04 | A:A | 3.74963 | 0.50012 |
| 2031012 | Vlasi-4 | D04 | G:G | 0.40543 | 3.57231 |
| 2031012 | Vlasi-4 | E04 | G:A | 1.86258 | 2.34327 |
| 2031012 | Vlasi-4 | F04 | G:A | 2.03659 | 2.35281 |
| 2031012 | Vlasi-4 | G04 | G:G | 0.42542 | 3.74569 |
| 2031012 | Vlasi-4 | H04 | G:A | 2.15071 | 2.44825 |
| 2031012 | Vlasi-4 | A05 | ? | 0.5745 | 0.41707 |
| 2031012 | Vlasi-4 | B05 | G:G | 0.45229 | 3.60421 |
| 2031012 | Vlasi-4 | C05 | G:A | 2.07744 | 2.32208 |
| 2031012 | Vlasi-4 | D05 | G:G | 0.39171 | 3.62414 |
| 2031012 | Vlasi-4 | E05 | G:G | 0.39609 | 3.50679 |
| 2031012 | Vlasi-4 | F05 | G:G | 0.40237 | 3.67836 |
| 2031012 | Vlasi-4 | G05 | G:A | 1.93868 | 2.39723 |
| 2031012 | Vlasi-4 | H05 | G:G | 0.42715 | 3.72227 |
| 2031012 | Vlasi-4 | A06 | A:A | 3.55203 | 0.44151 |
| 2031012 | Vlasi-4 | B06 | G:A | 1.97889 | 2.32246 |
| 2031012 | Vlasi-4 | C06 | A:A | 3.64063 | 0.50383 |
| 2031012 | Vlasi-4 | D06 | G:A | 1.95659 | 2.18161 |
| 2031012 | Vlasi-4 | E06 | G:A | 2.23184 | 2.54515 |
| 2031012 | Vlasi-4 | F06 | G:G | 0.41357 | 3.75564 |
| 2031012 | Vlasi-4 | G06 | G:G | 0.4082 | 3.74324 |
| 2031012 | Vlasi-4 | H06 | G:A | 2.09829 | 2.45561 |
| 2031012 | Vlasi-4 | A07 | ? | 0.56873 | 0.70694 |

| | | | | | |
|---|---|---|---|---|---|
| 2031012 | Vlasi-4 | B07 | ? | 0.57259 | 0.43742 |
| 2031012 | Vlasi-4 | C07 | ? | 0.5482 | 0.47336 |
| 2031012 | Vlasi-4 | D07 | ? | 0.55922 | 0.50974 |
| 2031012 | Vlasi-4 | E07 | ? | 0.56583 | 0.46867 |
| 2031012 | Vlasi-4 | F07 | A:A | 3.48285 | 0.75171 |
| 2031012 | Vlasi-4 | G07 | A:A | 3.87127 | 0.70021 |
| 2031012 | Vlasi-4 | H07 | A:A | 3.79678 | 0.81409 |
| 2031012 | Vlasi-4 | A08 | A:A | 3.58807 | 0.75463 |
| 2031012 | Vlasi-4 | B08 | A:A | 3.44363 | 0.91941 |
| 2031012 | Vlasi-4 | C08 | A:A | 3.50089 | 0.87964 |
| 2031012 | Vlasi-4 | D08 | A:A | 3.63663 | 0.78833 |
| 2031012 | Vlasi-4 | E08 | A:A | 3.58855 | 0.8631 |
| 2031012 | Vlasi-4 | F08 | A:A | 3.61154 | 0.82252 |
| 2031012 | Vlasi-4 | G08 | A:A | 3.65671 | 0.74455 |
| 2031012 | Vlasi-4 | H08 | A:A | 3.60648 | 0.86417 |
| 2031012 | Vlasi-4 | A09 | A:A | 3.58054 | 0.91006 |
| 2031012 | Vlasi-4 | B09 | A:A | 3.51281 | 0.7887 |
| 2031012 | Vlasi-4 | C09 | A:A | 3.70318 | 0.8553 |
| 2031012 | Vlasi-4 | D09 | A:A | 3.62156 | 0.87236 |
| 2031012 | Vlasi-4 | E09 | A:A | 3.62041 | 0.84997 |
| 2031012 | Vlasi-4 | F09 | A:A | 3.62171 | 0.82398 |
| 2031012 | Vlasi-4 | G09 | A:A | 3.64648 | 0.85978 |
| 2031012 | Vlasi-4 | H09 | A:A | 3.7144 | 0.8186 |
| 2031012 | Vlasi-4 | A10 | A:A | 3.35728 | 0.9146 |
| 2031012 | Vlasi-4 | B10 | A:A | 3.41878 | 0.84236 |
| 2031012 | Vlasi-4 | C10 | G:G | 0.39798 | 3.48976 |
| 2031012 | Vlasi-4 | D10 | G:G | 0.39428 | 3.60842 |
| 2031012 | Vlasi-4 | E10 | G:G | 0.44655 | 3.63469 |
| 2031012 | Vlasi-4 | F10 | G:G | 0.42456 | 3.55134 |
| 2031012 | Vlasi-4 | G10 | G:G | 0.49873 | 3.66583 |
| 2031012 | Vlasi-4 | H10 | G:G | 0.46353 | 3.82223 |
| 2031012 | Vlasi-4 | A11 | G:G | 0.56129 | 3.30823 |
| 2031012 | Vlasi-4 | B11 | G:G | 0.48261 | 3.4891 |
| 2031012 | Vlasi-4 | C11 | G:G | 0.61639 | 3.64176 |
| 2031012 | Vlasi-4 | D11 | G:G | 0.4563 | 3.72047 |
| 2031012 | Vlasi-4 | E11 | G:G | 0.47923 | 3.65682 |
| 2031012 | Vlasi-4 | F11 | G:G | 0.45275 | 3.6047 |
| 2031012 | Vlasi-4 | G11 | G:G | 0.55143 | 3.49675 |
| 2031012 | Vlasi-4 | H11 | G:G | 0.48591 | 3.79203 |
| 2031012 | Vlasi-4 | A12 | G:G | 0.6202 | 3.40978 |
| 2031012 | Vlasi-4 | B12 | G:G | 0.56223 | 3.52051 |
| 2031012 | Vlasi-4 | C12 | G:G | 0.57191 | 3.37556 |
| 2031012 | Vlasi-4 | D12 | G:G | 0.61919 | 3.56655 |
| 2031012 | Vlasi-4 | E12 | G:G | 0.56837 | 3.61481 |
| 2031012 | Vlasi-4 | F12 | G:G | 0.56314 | 3.53463 |
| 2031012 | Vlasi-4 | G12 | G:G | 0.54309 | 3.58935 |
| 2031012 | Vlasi-4 | H12 | NTC | 0.68761 | 0.56615 |
| 2031012 | Vlasi-1 | A01 | T:T | 0.3611 | 3.64806 |
| 2031012 | Vlasi-1 | B01 | T:G | 3.26176 | 1.29311 |
| 2031012 | Vlasi-1 | C01 | T:T | 0.34677 | 3.77859 |

| | | | | | |
|---|---|---|---|---|---|
| 2031012 | Vlasi-1 | D01 | T:T | 0.32993 | 3.87251 |
| 2031012 | Vlasi-1 | E01 | T:G | 3.4337 | 1.21688 |
| 2031012 | Vlasi-1 | F01 | G:G | 4.05874 | 0.45862 |
| 2031012 | Vlasi-1 | G01 | G:G | 4.02557 | 0.48518 |
| 2031012 | Vlasi-1 | H01 | G:G | 4.07803 | 0.53156 |
| 2031012 | Vlasi-1 | A02 | G:G | 3.90296 | 0.48863 |
| 2031012 | Vlasi-1 | B02 | G:G | 3.95458 | 0.477 |
| 2031012 | Vlasi-1 | C02 | T:G | 3.02869 | 1.0349 |
| 2031012 | Vlasi-1 | D02 | G:G | 4.0271 | 0.50113 |
| 2031012 | Vlasi-1 | E02 | T:G | 2.78737 | 1.19493 |
| 2031012 | Vlasi-1 | F02 | T:T | 0.39967 | 3.74022 |
| 2031012 | Vlasi-1 | G02 | T:T | 0.41468 | 3.79421 |
| 2031012 | Vlasi-1 | H02 | T:G | 3.11338 | 1.24612 |
| 2031012 | Vlasi-1 | A03 | T:T | 0.40173 | 3.64271 |
| 2031012 | Vlasi-1 | B03 | T:T | 0.42027 | 3.67574 |
| 2031012 | Vlasi-1 | C03 | T:T | 0.40179 | 3.70956 |
| 2031012 | Vlasi-1 | D03 | T:T | 0.45133 | 3.65775 |
| 2031012 | Vlasi-1 | E03 | T:G | 2.81815 | 1.17445 |
| 2031012 | Vlasi-1 | F03 | G:G | 3.72638 | 0.47985 |
| 2031012 | Vlasi-1 | G03 | G:G | 3.83646 | 0.50533 |
| 2031012 | Vlasi-1 | H03 | T:G | 2.89935 | 1.29813 |
| 2031012 | Vlasi-1 | A04 | T:T | 0.41522 | 3.84191 |
| 2031012 | Vlasi-1 | B04 | T:G | 2.83779 | 1.41253 |
| 2031012 | Vlasi-1 | C04 | T:T | 0.4018 | 3.61597 |
| 2031012 | Vlasi-1 | D04 | T:T | 0.3881 | 3.72665 |
| 2031012 | Vlasi-1 | E04 | T:G | 2.84053 | 1.20208 |
| 2031012 | Vlasi-1 | F04 | T:G | 3.15804 | 1.21897 |
| 2031012 | Vlasi-1 | G04 | G:G | 3.88212 | 0.50158 |
| 2031012 | Vlasi-1 | H04 | T:T | 0.38849 | 3.55998 |
| 2031012 | Vlasi-1 | A05 | G:G | 3.81276 | 0.49459 |
| 2031012 | Vlasi-1 | B05 | T:T | 0.38899 | 3.60359 |
| 2031012 | Vlasi-1 | C05 | T:G | 2.81801 | 1.05577 |
| 2031012 | Vlasi-1 | D05 | G:G | 3.90496 | 0.50912 |
| 2031012 | Vlasi-1 | E05 | T:T | 0.37561 | 3.58603 |
| 2031012 | Vlasi-1 | F05 | G:G | 4.04315 | 0.51178 |
| 2031012 | Vlasi-1 | G05 | T:G | 3.01318 | 1.05387 |
| 2031012 | Vlasi-1 | H05 | G:G | 3.87755 | 0.48944 |
| 2031012 | Vlasi-1 | A06 | T:G | 2.85454 | 1.65495 |
| 2031012 | Vlasi-1 | B06 | T:G | 3.03201 | 1.00019 |
| 2031012 | Vlasi-1 | C06 | T:T | 0.40129 | 3.80537 |
| 2031012 | Vlasi-1 | D06 | T:G | 3.01889 | 1.2509 |
| 2031012 | Vlasi-1 | E06 | T:G | 2.87831 | 1.16447 |
| 2031012 | Vlasi-1 | F06 | G:G | 3.93631 | 0.51343 |
| 2031012 | Vlasi-1 | G06 | G:G | 3.92091 | 0.50597 |
| 2031012 | Vlasi-1 | H06 | T:T | 0.39105 | 3.70413 |
| 2031012 | Vlasi-1 | A07 | T:G | 2.787 | 1.49106 |
| 2031012 | Vlasi-1 | B07 | T:G | 2.89453 | 1.20838 |
| 2031012 | Vlasi-1 | C07 | G:G | 3.88701 | 0.47861 |
| 2031012 | Vlasi-1 | D07 | T:G | 2.85163 | 1.13227 |
| 2031012 | Vlasi-1 | E07 | T:G | 2.76248 | 1.21811 |

| | | | | | |
|---|---|---|---|---|---|
| 2031012 | Vlasi-1 | F07 | T:G | 2.75623 | 1.28122 |
| 2031012 | Vlasi-1 | G07 | T:T | 0.38852 | 3.69636 |
| 2031012 | Vlasi-1 | H07 | T:G | 2.95738 | 1.28938 |
| 2031012 | Vlasi-1 | A08 | G:G | 3.77997 | 0.50239 |
| 2031012 | Vlasi-1 | B08 | G:G | 3.96893 | 0.4603 |
| 2031012 | Vlasi-1 | C08 | T:T | 0.3931 | 3.63094 |
| 2031012 | Vlasi-1 | D08 | T:T | 0.41741 | 3.58 |
| 2031012 | Vlasi-1 | E08 | T:T | 0.42284 | 3.64421 |
| 2031012 | Vlasi-1 | F08 | T:G | 2.88525 | 1.17092 |
| 2031012 | Vlasi-1 | G08 | T:G | 2.90915 | 1.18446 |
| 2031012 | Vlasi-1 | H08 | T:G | 2.9492 | 1.23645 |
| 2031012 | Vlasi-1 | A09 | T:G | 3.06665 | 1.47376 |
| 2031012 | Vlasi-1 | B09 | G:G | 3.90249 | 0.48843 |
| 2031012 | Vlasi-1 | C09 | T:G | 3.11522 | 1.2862 |
| 2031012 | Vlasi-1 | D09 | T:G | 2.99555 | 1.29428 |
| 2031012 | Vlasi-1 | E09 | T:G | 2.91771 | 1.26896 |
| 2031012 | Vlasi-1 | F09 | T:T | 0.41738 | 3.8025 |
| 2031012 | Vlasi-1 | G09 | T:G | 2.85615 | 1.22839 |
| 2031012 | Vlasi-1 | H09 | G:G | 4.02335 | 0.49787 |
| 2031012 | Vlasi-1 | A10 | T:G | 2.92929 | 1.2557 |
| 2031012 | Vlasi-1 | B10 | T:G | 2.95443 | 1.14432 |
| 2031012 | Vlasi-1 | C10 | T:G | 2.9892 | 1.34033 |
| 2031012 | Vlasi-1 | D10 | T:G | 2.92121 | 1.31635 |
| 2031012 | Vlasi-1 | E10 | T:G | 2.7035 | 1.29338 |
| 2031012 | Vlasi-1 | F10 | T:G | 2.81449 | 1.33503 |
| 2031012 | Vlasi-1 | G10 | T:T | 0.38177 | 3.75259 |
| 2031012 | Vlasi-1 | H10 | T:G | 3.16243 | 1.25574 |
| 2031012 | Vlasi-1 | A11 | T:G | 2.73774 | 1.47932 |
| 2031012 | Vlasi-1 | B11 | G:G | 3.8587 | 0.49354 |
| 2031012 | Vlasi-1 | C11 | T:T | 0.39515 | 3.5787 |
| 2031012 | Vlasi-1 | D11 | G:G | 3.71045 | 0.46191 |
| 2031012 | Vlasi-1 | E11 | T:G | 2.97596 | 1.19775 |
| 2031012 | Vlasi-1 | F11 | T:T | 0.40228 | 3.78237 |
| 2031012 | Vlasi-1 | G11 | T:T | 0.39956 | 3.86411 |
| 2031012 | Vlasi-1 | H11 | T:G | 2.99266 | 1.30436 |
| 2031012 | Vlasi-1 | A12 | T:G | 2.87121 | 1.42718 |
| 2031012 | Vlasi-1 | B12 | T:G | 2.83887 | 1.39036 |
| 2031012 | Vlasi-1 | C12 | G:G | 3.92538 | 0.47623 |
| 2031012 | Vlasi-1 | D12 | T:G | 2.97935 | 1.35447 |
| 2031012 | Vlasi-1 | E12 | T:G | 3.00542 | 1.41222 |
| 2031012 | Vlasi-1 | F12 | G:G | 3.93041 | 0.44717 |
| 2031012 | Vlasi-1 | G12 | T:G | 2.95883 | 1.20976 |
| 2031012 | Vlasi-1 | H12 | G:G | 4.01364 | 0.51208 |
| 2031012 | Vlasi-2 | A01 | T:T | 0.3878 | 3.67425 |
| 2031012 | Vlasi-2 | B01 | T:G | 2.88522 | 1.21239 |
| 2031012 | Vlasi-2 | C01 | T:T | 0.3748 | 3.67523 |
| 2031012 | Vlasi-2 | D01 | T:T | 0.3884 | 3.82622 |
| 2031012 | Vlasi-2 | E01 | T:T | 0.35945 | 3.62232 |
| 2031012 | Vlasi-2 | F01 | T:G | 2.50964 | 0.94719 |
| 2031012 | Vlasi-2 | G01 | T:G | 3.15356 | 1.09736 |

| | | | | | |
|---|---|---|---|---|---|
| 2031012 | Vlasi-2 | H01 | G:G | 4.09359 | 0.47362 |
| 2031012 | Vlasi-2 | A02 | T:G | 2.88394 | 1.71032 |
| 2031012 | Vlasi-2 | B02 | G:G | 3.85286 | 0.42288 |
| 2031012 | Vlasi-2 | C02 | T:G | 2.91198 | 1.18657 |
| 2031012 | Vlasi-2 | D02 | T:T | 0.39803 | 3.66093 |
| 2031012 | Vlasi-2 | E02 | T:T | 0.36164 | 3.49782 |
| 2031012 | Vlasi-2 | F02 | T:G | 2.78146 | 1.23988 |
| 2031012 | Vlasi-2 | G02 | T:G | 3.01324 | 1.09563 |
| 2031012 | Vlasi-2 | H02 | G:G | 3.98748 | 0.48884 |
| 2031012 | Vlasi-2 | A03 | G:G | 3.87731 | 0.53646 |
| 2031012 | Vlasi-2 | B03 | T:T | 0.39066 | 3.69016 |
| 2031012 | Vlasi-2 | C03 | G:G | 3.80904 | 0.47179 |
| 2031012 | Vlasi-2 | D03 | T:G | 3.07721 | 1.11877 |
| 2031012 | Vlasi-2 | E03 | G:G | 3.82227 | 0.53818 |
| 2031012 | Vlasi-2 | F03 | T:T | 0.40942 | 3.69485 |
| 2031012 | Vlasi-2 | G03 | G:G | 3.81681 | 0.53688 |
| 2031012 | Vlasi-2 | H03 | T:T | 0.44286 | 3.67076 |
| 2031012 | Vlasi-2 | A04 | T:T | 0.44192 | 3.63307 |
| 2031012 | Vlasi-2 | B04 | T:G | 2.91766 | 1.20026 |
| 2031012 | Vlasi-2 | C04 | T:G | 2.76489 | 1.15469 |
| 2031012 | Vlasi-2 | D04 | T:G | 2.70489 | 1.24524 |
| 2031012 | Vlasi-2 | E04 | G:G | 3.64518 | 0.42381 |
| 2031012 | Vlasi-2 | F04 | G:G | 3.91386 | 0.52276 |
| 2031012 | Vlasi-2 | G04 | T:G | 3.09554 | 1.03564 |
| 2031012 | Vlasi-2 | H04 | T:G | 2.60363 | 1.13346 |
| 2031012 | Vlasi-2 | A05 | T:G | 3.09195 | 1.48722 |
| 2031012 | Vlasi-2 | B05 | T:G | 2.8948 | 1.04248 |
| 2031012 | Vlasi-2 | C05 | T:G | 2.91818 | 1.15211 |
| 2031012 | Vlasi-2 | D05 | T:T | 0.39096 | 3.72775 |
| 2031012 | Vlasi-2 | E05 | T:G | 2.83758 | 1.14467 |
| 2031012 | Vlasi-2 | F05 | T:G | 2.76012 | 1.17485 |
| 2031012 | Vlasi-2 | G05 | T:G | 3.03834 | 1.10952 |
| 2031012 | Vlasi-2 | H05 | T:G | 3.00768 | 1.3312 |
| 2031012 | Vlasi-2 | A06 | G:G | 3.9057 | 0.47168 |
| 2031012 | Vlasi-2 | B06 | T:G | 2.87614 | 1.18495 |
| 2031012 | Vlasi-2 | C06 | T:T | 0.41564 | 3.79987 |
| 2031012 | Vlasi-2 | D06 | T:G | 2.99822 | 1.1876 |
| 2031012 | Vlasi-2 | E06 | G:G | 3.891 | 0.49151 |
| 2031012 | Vlasi-2 | F06 | T:G | 2.75086 | 1.17398 |
| 2031012 | Vlasi-2 | G06 | T:G | 2.92263 | 1.16056 |
| 2031012 | Vlasi-2 | H06 | T:G | 2.88252 | 1.27016 |
| 2031012 | Vlasi-2 | A07 | T:G | 2.97608 | 1.26839 |
| 2031012 | Vlasi-2 | B07 | T:G | 2.74418 | 1.16691 |
| 2031012 | Vlasi-2 | C07 | T:G | 3.16865 | 1.26038 |
| 2031012 | Vlasi-2 | D07 | T:T | 0.38014 | 3.72623 |
| 2031012 | Vlasi-2 | E07 | T:G | 2.74897 | 1.09959 |
| 2031012 | Vlasi-2 | F07 | T:G | 2.91567 | 1.14978 |
| 2031012 | Vlasi-2 | G07 | T:G | 3.02389 | 1.11674 |
| 2031012 | Vlasi-2 | H07 | T:G | 3.07998 | 1.32431 |
| 2031012 | Vlasi-2 | A08 | T:T | 0.42869 | 3.53087 |

| | | | | | |
|---|---|---|---|---|---|
| 2031012 | Vlasi-2 | B08 | T:G | 2.95014 | 1.24401 |
| 2031012 | Vlasi-2 | C08 | T:T | 0.49226 | 3.55125 |
| 2031012 | Vlasi-2 | D08 | T:G | 2.87648 | 1.30765 |
| 2031012 | Vlasi-2 | E08 | T:T | 0.38915 | 3.70272 |
| 2031012 | Vlasi-2 | F08 | G:G | 3.85862 | 0.46386 |
| 2031012 | Vlasi-2 | G08 | T:G | 3.02586 | 1.14306 |
| 2031012 | Vlasi-2 | H08 | G:G | 3.94186 | 0.4556 |
| 2031012 | Vlasi-2 | A09 | T:T | 0.47605 | 3.63241 |
| 2031012 | Vlasi-2 | B09 | T:G | 3.04626 | 1.24524 |
| 2031012 | Vlasi-2 | C09 | T:G | 2.90549 | 0.94981 |
| 2031012 | Vlasi-2 | D09 | T:G | 2.88993 | 1.31378 |
| 2031012 | Vlasi-2 | E09 | T:T | 0.42026 | 3.74221 |
| 2031012 | Vlasi-2 | F09 | T:G | 2.4436 | 0.91733 |
| 2031012 | Vlasi-2 | G09 | T:G | 3.02881 | 1.25376 |
| 2031012 | Vlasi-2 | H09 | T:T | 0.41665 | 3.84233 |
| 2031012 | Vlasi-2 | A10 | T:G | 3.01905 | 1.37488 |
| 2031012 | Vlasi-2 | B10 | T:G | 3.02141 | 1.10523 |
| 2031012 | Vlasi-2 | C10 | G:G | 3.99361 | 0.47371 |
| 2031012 | Vlasi-2 | D10 | T:G | 2.96302 | 1.09527 |
| 2031012 | Vlasi-2 | E10 | G:G | 4.08935 | 0.45719 |
| 2031012 | Vlasi-2 | F10 | T:G | 3.07392 | 1.16403 |
| 2031012 | Vlasi-2 | G10 | G:G | 4.15705 | 0.5358 |
| 2031012 | Vlasi-2 | H10 | T:T | 0.4118 | 3.7982 |
| 2031012 | Vlasi-2 | A11 | T:G | 2.87983 | 1.43746 |
| 2031012 | Vlasi-2 | B11 | T:G | 2.84948 | 1.34568 |
| 2031012 | Vlasi-2 | C11 | T:G | 2.97171 | 1.24116 |
| 2031012 | Vlasi-2 | D11 | T:G | 2.96175 | 1.38462 |
| 2031012 | Vlasi-2 | E11 | G:G | 3.90275 | 0.49247 |
| 2031012 | Vlasi-2 | F11 | T:G | 2.94734 | 1.27885 |
| 2031012 | Vlasi-2 | G11 | G:G | 3.84831 | 0.50876 |
| 2031012 | Vlasi-2 | H11 | T:G | 3.09151 | 1.53765 |
| 2031012 | Vlasi-2 | A12 | G:G | 3.75471 | 0.462 |
| 2031012 | Vlasi-2 | B12 | T:T | 0.41683 | 3.71898 |
| 2031012 | Vlasi-2 | C12 | G:G | 3.76626 | 0.49287 |
| 2031012 | Vlasi-2 | D12 | T:G | 3.00315 | 1.33587 |
| 2031012 | Vlasi-2 | E12 | T:G | 3.0634 | 1.3 |
| 2031012 | Vlasi-2 | F12 | T:T | 0.39654 | 3.70857 |
| 2031012 | Vlasi-2 | G12 | G:G | 3.87646 | 0.48741 |
| 2031012 | Vlasi-2 | H12 | G:G | 4.16661 | 0.53895 |
| 2031012 | Vlasi-3 | A01 | T:G | 3.28961 | 1.38084 |
| 2031012 | Vlasi-3 | B01 | G:G | 4.10199 | 0.48689 |
| 2031012 | Vlasi-3 | C01 | T:T | 0.31979 | 3.61165 |
| 2031012 | Vlasi-3 | D01 | G:G | 4.0303 | 0.44312 |
| 2031012 | Vlasi-3 | E01 | G:G | 4.14107 | 0.44477 |
| 2031012 | Vlasi-3 | F01 | T:G | 3.37566 | 1.1427 |
| 2031012 | Vlasi-3 | G01 | T:T | 0.39979 | 3.79292 |
| 2031012 | Vlasi-3 | H01 | T:G | 3.1416 | 1.54518 |
| 2031012 | Vlasi-3 | A02 | G:G | 3.97699 | 0.47023 |
| 2031012 | Vlasi-3 | B02 | T:G | 2.97008 | 1.17722 |
| 2031012 | Vlasi-3 | C02 | T:G | 2.8751 | 1.28066 |

| | | | | | |
|---|---|---|---|---|---|
| 2031012 | Vlasi-3 | D02 | T:G | 3.10448 | 1.21336 |
| 2031012 | Vlasi-3 | E02 | T:G | 2.91633 | 1.14168 |
| 2031012 | Vlasi-3 | F02 | T:T | 0.39843 | 3.70012 |
| 2031012 | Vlasi-3 | G02 | T:G | 3.07298 | 1.16407 |
| 2031012 | Vlasi-3 | H02 | T:G | 3.24665 | 1.24509 |
| 2031012 | Vlasi-3 | A03 | T:T | 0.37339 | 3.54109 |
| 2031012 | Vlasi-3 | B03 | T:T | 0.38572 | 3.79696 |
| 2031012 | Vlasi-3 | C03 | T:G | 2.82121 | 1.11673 |
| 2031012 | Vlasi-3 | D03 | T:T | 0.39896 | 3.67424 |
| 2031012 | Vlasi-3 | E03 | T:G | 2.92105 | 1.13865 |
| 2031012 | Vlasi-3 | F03 | G:G | 3.83571 | 0.48773 |
| 2031012 | Vlasi-3 | G03 | T:G | 3.00265 | 1.11821 |
| 2031012 | Vlasi-3 | H03 | T:G | 3.39925 | 1.29049 |
| 2031012 | Vlasi-3 | A04 | T:T | 0.39575 | 3.81187 |
| 2031012 | Vlasi-3 | B04 | G:G | 3.78987 | 0.44661 |
| 2031012 | Vlasi-3 | C04 | T:T | 0.40314 | 3.8284 |
| 2031012 | Vlasi-3 | D04 | T:G | 2.77997 | 1.16548 |
| 2031012 | Vlasi-3 | E04 | T:T | 0.37996 | 3.53877 |
| 2031012 | Vlasi-3 | F04 | T:G | 2.85442 | 1.18958 |
| 2031012 | Vlasi-3 | G04 | T:G | 2.93514 | 1.01484 |
| 2031012 | Vlasi-3 | H04 | T:G | 3.21762 | 1.31181 |
| 2031012 | Vlasi-3 | A05 | T:G | 2.99957 | 1.27992 |
| 2031012 | Vlasi-3 | B05 | T:T | 0.36597 | 3.43884 |
| 2031012 | Vlasi-3 | C05 | G:G | 3.93494 | 0.51174 |
| 2031012 | Vlasi-3 | D05 | T:T | 0.39188 | 3.57964 |
| 2031012 | Vlasi-3 | E05 | T:T | 0.4016 | 3.78413 |
| 2031012 | Vlasi-3 | F05 | T:G | 3.01469 | 1.20451 |
| 2031012 | Vlasi-3 | G05 | T:T | 0.41182 | 3.50694 |
| 2031012 | Vlasi-3 | H05 | T:T | 0.40436 | 3.80284 |
| 2031012 | Vlasi-3 | A06 | G:G | 3.73268 | 0.48408 |
| 2031012 | Vlasi-3 | B06 | T:T | 0.39123 | 3.66128 |
| 2031012 | Vlasi-3 | C06 | G:G | 3.8845 | 0.49377 |
| 2031012 | Vlasi-3 | D06 | T:G | 2.85848 | 1.25042 |
| 2031012 | Vlasi-3 | E06 | T:G | 2.83464 | 1.20284 |
| 2031012 | Vlasi-3 | F06 | T:T | 0.43059 | 3.54406 |
| 2031012 | Vlasi-3 | G06 | T:G | 2.98301 | 1.24263 |
| 2031012 | Vlasi-3 | H06 | T:G | 3.10156 | 1.25357 |
| 2031012 | Vlasi-3 | A07 | T:G | 2.94669 | 1.18555 |
| 2031012 | Vlasi-3 | B07 | G:G | 3.76156 | 0.51186 |
| 2031012 | Vlasi-3 | C07 | G:G | 4.0687 | 0.44869 |
| 2031012 | Vlasi-3 | D07 | G:G | 3.72097 | 0.50108 |
| 2031012 | Vlasi-3 | E07 | G:G | 3.67858 | 0.43825 |
| 2031012 | Vlasi-3 | F07 | G:G | 3.81975 | 0.51981 |
| 2031012 | Vlasi-3 | G07 | T:T | 0.41012 | 3.6434 |
| 2031012 | Vlasi-3 | H07 | T:G | 3.13781 | 1.44914 |
| 2031012 | Vlasi-3 | A08 | T:T | 0.38223 | 3.71431 |
| 2031012 | Vlasi-3 | B08 | T:G | 2.89694 | 1.12182 |
| 2031012 | Vlasi-3 | C08 | G:G | 4.00097 | 0.53003 |
| 2031012 | Vlasi-3 | D08 | T:T | 0.36347 | 3.43246 |
| 2031012 | Vlasi-3 | E08 | T:G | 2.86032 | 1.09018 |

| | | | | | |
|---|---|---|---|---|---|
| 2031012 | Vlasi-3 | F08 | G:G | 3.99566 | 0.51077 |
| 2031012 | Vlasi-3 | G08 | T:G | 2.94462 | 1.16512 |
| 2031012 | Vlasi-3 | H08 | T:G | 3.1353 | 1.59058 |
| 2031012 | Vlasi-3 | A09 | T:G | 2.76232 | 1.37444 |
| 2031012 | Vlasi-3 | B09 | T:G | 2.85349 | 1.23916 |
| 2031012 | Vlasi-3 | C09 | T:G | 2.79717 | 1.0641 |
| 2031012 | Vlasi-3 | D09 | T:T | 0.36773 | 3.59658 |
| 2031012 | Vlasi-3 | E09 | T:T | 0.36128 | 3.50018 |
| 2031012 | Vlasi-3 | F09 | T:G | 3.00032 | 1.22613 |
| 2031012 | Vlasi-3 | G09 | T:T | 0.46367 | 3.69362 |
| 2031012 | Vlasi-3 | H09 | G:G | 4.09698 | 0.51738 |
| 2031012 | Vlasi-3 | A10 | G:G | 3.83196 | 0.47954 |
| 2031012 | Vlasi-3 | B10 | G:G | 3.84618 | 0.50221 |
| 2031012 | Vlasi-3 | C10 | T:G | 2.97348 | 1.13494 |
| 2031012 | Vlasi-3 | D10 | G:G | 3.85821 | 0.51179 |
| 2031012 | Vlasi-3 | E10 | T:T | 0.37771 | 3.74066 |
| 2031012 | Vlasi-3 | F10 | T:T | 0.39541 | 3.81191 |
| 2031012 | Vlasi-3 | G10 | G:G | 4.08255 | 0.52518 |
| 2031012 | Vlasi-3 | H10 | G:G | 4.18354 | 0.5507 |
| 2031012 | Vlasi-3 | A11 | T:T | 0.40365 | 3.71643 |
| 2031012 | Vlasi-3 | B11 | G:G | 3.85149 | 0.46077 |
| 2031012 | Vlasi-3 | C11 | G:G | 3.89835 | 0.52609 |
| 2031012 | Vlasi-3 | D11 | T:T | 0.38007 | 3.85985 |
| 2031012 | Vlasi-3 | E11 | T:T | 0.37963 | 3.70988 |
| 2031012 | Vlasi-3 | F11 | T:T | 0.46498 | 3.73608 |
| 2031012 | Vlasi-3 | G11 | T:G | 3.1406 | 1.26596 |
| 2031012 | Vlasi-3 | H11 | T:G | 3.33544 | 1.37505 |
| 2031012 | Vlasi-3 | A12 | T:G | 2.78209 | 1.39155 |
| 2031012 | Vlasi-3 | B12 | T:T | 0.43508 | 3.56869 |
| 2031012 | Vlasi-3 | C12 | T:T | 0.37193 | 3.6694 |
| 2031012 | Vlasi-3 | D12 | T:G | 3.13073 | 1.32521 |
| 2031012 | Vlasi-3 | E12 | T:G | 3.00871 | 1.40176 |
| 2031012 | Vlasi-3 | F12 | T:G | 2.86579 | 1.40341 |
| 2031012 | Vlasi-3 | G12 | T:G | 3.13603 | 1.22626 |
| 2031012 | Vlasi-3 | H12 | T:G | 2.94103 | 1.81898 |
| 2031012 | Vlasi-4 | A01 | T:G | 2.9727 | 1.18056 |
| 2031012 | Vlasi-4 | B01 | T:T | 0.36209 | 3.64525 |
| 2031012 | Vlasi-4 | C01 | T:G | 2.85142 | 1.11603 |
| 2031012 | Vlasi-4 | D01 | G:G | 3.8407 | 0.46461 |
| 2031012 | Vlasi-4 | E01 | T:G | 2.68655 | 1.12228 |
| 2031012 | Vlasi-4 | F01 | T:G | 3.0249 | 1.07405 |
| 2031012 | Vlasi-4 | G01 | T:G | 3.063 | 1.12566 |
| 2031012 | Vlasi-4 | H01 | G:G | 3.98468 | 0.51862 |
| 2031012 | Vlasi-4 | A02 | T:G | 3.10939 | 1.27316 |
| 2031012 | Vlasi-4 | B02 | T:G | 2.8285 | 1.28649 |
| 2031012 | Vlasi-4 | C02 | G:G | 4.03617 | 0.49436 |
| 2031012 | Vlasi-4 | D02 | G:G | 4.01494 | 0.4644 |
| 2031012 | Vlasi-4 | E02 | T:G | 2.81093 | 1.18769 |
| 2031012 | Vlasi-4 | F02 | T:G | 2.89236 | 1.18146 |
| 2031012 | Vlasi-4 | G02 | T:G | 3.11271 | 0.97821 |

| | | | | | |
|---|---|---|---|---|---|
| 2031012 | Vlasi-4 | H02 | G:G | 4.04736 | 0.5272 |
| 2031012 | Vlasi-4 | A03 | T:G | 3.03305 | 1.39446 |
| 2031012 | Vlasi-4 | B03 | T:G | 2.92727 | 1.14072 |
| 2031012 | Vlasi-4 | C03 | T:T | 0.39018 | 3.6952 |
| 2031012 | Vlasi-4 | D03 | T:G | 2.80669 | 1.20787 |
| 2031012 | Vlasi-4 | E03 | T:T | 0.3901 | 3.92296 |
| 2031012 | Vlasi-4 | F03 | T:G | 2.87282 | 1.15699 |
| 2031012 | Vlasi-4 | G03 | T:G | 2.92717 | 1.21506 |
| 2031012 | Vlasi-4 | H03 | T:T | 0.41928 | 3.81519 |
| 2031012 | Vlasi-4 | A04 | T:G | 2.72773 | 1.17768 |
| 2031012 | Vlasi-4 | B04 | T:G | 2.71196 | 1.07677 |
| 2031012 | Vlasi-4 | C04 | T:G | 2.82288 | 1.20914 |
| 2031012 | Vlasi-4 | D04 | T:G | 2.75012 | 1.12001 |
| 2031012 | Vlasi-4 | E04 | T:G | 2.8472 | 1.06619 |
| 2031012 | Vlasi-4 | F04 | G:G | 3.91938 | 0.55408 |
| 2031012 | Vlasi-4 | G04 | G:G | 4.15879 | 0.50556 |
| 2031012 | Vlasi-4 | H04 | T:T | 0.436 | 3.82788 |
| 2031012 | Vlasi-4 | A05 | T:G | 2.81772 | 1.21918 |
| 2031012 | Vlasi-4 | B05 | T:G | 2.78787 | 1.21178 |
| 2031012 | Vlasi-4 | C05 | T:T | 0.38494 | 3.72829 |
| 2031012 | Vlasi-4 | D05 | G:G | 3.82293 | 0.53038 |
| 2031012 | Vlasi-4 | E05 | T:G | 2.83065 | 1.10484 |
| 2031012 | Vlasi-4 | F05 | T:T | 0.37566 | 3.45995 |
| 2031012 | Vlasi-4 | G05 | G:G | 4.0098 | 0.4652 |
| 2031012 | Vlasi-4 | H05 | T:G | 3.1558 | 1.29663 |
| 2031012 | Vlasi-4 | A06 | G:G | 3.82436 | 0.44021 |
| 2031012 | Vlasi-4 | B06 | G:G | 3.66778 | 0.43137 |
| 2031012 | Vlasi-4 | C06 | T:G | 2.81044 | 1.22089 |
| 2031012 | Vlasi-4 | D06 | T:G | 2.79029 | 0.99756 |
| 2031012 | Vlasi-4 | E06 | T:G | 3.07253 | 1.16318 |
| 2031012 | Vlasi-4 | F06 | T:G | 2.97223 | 1.14 |
| 2031012 | Vlasi-4 | G06 | G:G | 3.86783 | 0.47735 |
| 2031012 | Vlasi-4 | H06 | G:G | 4.08455 | 0.55924 |
| 2031012 | Vlasi-4 | A07 | G:G | 3.19848 | 0.47219 |
| 2031012 | Vlasi-4 | B07 | ? | 0.54814 | 0.51366 |
| 2031012 | Vlasi-4 | C07 | ? | 0.58897 | 0.51255 |
| 2031012 | Vlasi-4 | D07 | ? | 0.52876 | 1.1418 |
| 2031012 | Vlasi-4 | E07 | ? | 0.63038 | 0.51018 |
| 2031012 | Vlasi-4 | F07 | G:G | 3.76124 | 0.52248 |
| 2031012 | Vlasi-4 | G07 | G:G | 3.97558 | 0.52627 |
| 2031012 | Vlasi-4 | H07 | G:G | 3.90093 | 0.5263 |
| 2031012 | Vlasi-4 | A08 | G:G | 3.78914 | 0.55961 |
| 2031012 | Vlasi-4 | B08 | G:G | 4.03241 | 0.55562 |
| 2031012 | Vlasi-4 | C08 | G:G | 3.80445 | 0.54803 |
| 2031012 | Vlasi-4 | D08 | G:G | 3.94147 | 0.50457 |
| 2031012 | Vlasi-4 | E08 | G:G | 3.86128 | 0.53596 |
| 2031012 | Vlasi-4 | F08 | G:G | 3.94578 | 0.51516 |
| 2031012 | Vlasi-4 | G08 | G:G | 3.96117 | 0.51217 |
| 2031012 | Vlasi-4 | H08 | G:G | 4.11174 | 0.59229 |
| 2031012 | Vlasi-4 | A09 | G:G | 3.8212 | 0.54516 |

| | | | | | |
|---|---|---|---|---|---|
| 2031012 | Vlasi-4 | B09 | G:G | 3.853 | 0.53122 |
| 2031012 | Vlasi-4 | C09 | G:G | 3.76332 | 0.54086 |
| 2031012 | Vlasi-4 | D09 | G:G | 3.93735 | 0.53089 |
| 2031012 | Vlasi-4 | E09 | G:G | 3.81717 | 0.52675 |
| 2031012 | Vlasi-4 | F09 | G:G | 3.82159 | 0.52749 |
| 2031012 | Vlasi-4 | G09 | G:G | 3.79846 | 0.50966 |
| 2031012 | Vlasi-4 | H09 | G:G | 3.98362 | 0.55958 |
| 2031012 | Vlasi-4 | A10 | G:G | 3.78497 | 0.55934 |
| 2031012 | Vlasi-4 | B10 | G:G | 3.84506 | 0.50588 |
| 2031012 | Vlasi-4 | C10 | T:T | 0.37645 | 3.71284 |
| 2031012 | Vlasi-4 | D10 | T:T | 0.40596 | 3.88389 |
| 2031012 | Vlasi-4 | E10 | T:T | 0.39785 | 3.57976 |
| 2031012 | Vlasi-4 | F10 | T:T | 0.40577 | 3.69674 |
| 2031012 | Vlasi-4 | G10 | T:T | 0.40033 | 3.72779 |
| 2031012 | Vlasi-4 | H10 | T:T | 0.43819 | 3.73509 |
| 2031012 | Vlasi-4 | A11 | T:T | 0.40151 | 3.56516 |
| 2031012 | Vlasi-4 | B11 | T:T | 0.42173 | 3.42453 |
| 2031012 | Vlasi-4 | C11 | T:T | 0.41387 | 3.78933 |
| 2031012 | Vlasi-4 | D11 | T:T | 0.38645 | 3.42123 |
| 2031012 | Vlasi-4 | E11 | T:T | 0.40576 | 3.57786 |
| 2031012 | Vlasi-4 | F11 | T:T | 0.41418 | 3.7844 |
| 2031012 | Vlasi-4 | G11 | T:T | 0.42563 | 3.82495 |
| 2031012 | Vlasi-4 | H11 | T:T | 0.44135 | 3.66936 |
| 2031012 | Vlasi-4 | A12 | T:T | 0.42979 | 3.54853 |
| 2031012 | Vlasi-4 | B12 | T:T | 0.39901 | 3.55169 |
| 2031012 | Vlasi-4 | C12 | T:T | 0.40363 | 3.64637 |
| 2031012 | Vlasi-4 | D12 | T:T | 0.41551 | 3.51778 |
| 2031012 | Vlasi-4 | E12 | T:T | 0.40953 | 3.55669 |
| 2031012 | Vlasi-4 | F12 | T:T | 0.42358 | 3.69638 |
| 2031012 | Vlasi-4 | G12 | T:T | 0.39498 | 3.61842 |
| 2031012 | Vlasi-4 | H12 | NTC | 0.61422 | 0.7221 |
| 2031012 | Vlasi-1 | A01 | T:T | 0.36438 | 3.68969 |
| 2031012 | Vlasi-1 | B01 | T:T | 0.34712 | 3.66873 |
| 2031012 | Vlasi-1 | C01 | T:C | 2.32447 | 2.21803 |
| 2031012 | Vlasi-1 | D01 | T:C | 2.31232 | 2.20974 |
| 2031012 | Vlasi-1 | E01 | T:C | 2.31058 | 2.12188 |
| 2031012 | Vlasi-1 | F01 | T:C | 2.44352 | 1.93629 |
| 2031012 | Vlasi-1 | G01 | C:C | 4.07467 | 0.45517 |
| 2031012 | Vlasi-1 | H01 | T:C | 2.47771 | 2.18996 |
| 2031012 | Vlasi-1 | A02 | T:C | 2.50218 | 2.31918 |
| 2031012 | Vlasi-1 | B02 | T:C | 2.25939 | 2.28036 |
| 2031012 | Vlasi-1 | C02 | T:T | 0.37264 | 3.72088 |
| 2031012 | Vlasi-1 | D02 | T:C | 2.30345 | 2.07802 |
| 2031012 | Vlasi-1 | E02 | T:T | 0.37073 | 3.61572 |
| 2031012 | Vlasi-1 | F02 | T:C | 2.44249 | 2.02707 |
| 2031012 | Vlasi-1 | G02 | T:C | 2.45815 | 2.10046 |
| 2031012 | Vlasi-1 | H02 | C:C | 3.83017 | 0.47873 |
| 2031012 | Vlasi-1 | A03 | T:C | 2.36422 | 2.1991 |
| 2031012 | Vlasi-1 | B03 | T:C | 2.3842 | 1.92733 |
| 2031012 | Vlasi-1 | C03 | T:C | 2.32598 | 1.96422 |

| | | | | | |
|---|---|---|---|---|---|
| 2031012 | Vlasi-1 | D03 | T:T | 0.38092 | 3.85298 |
| 2031012 | Vlasi-1 | E03 | T:T | 0.37128 | 3.5479 |
| 2031012 | Vlasi-1 | F03 | T:T | 0.35843 | 3.64179 |
| 2031012 | Vlasi-1 | G03 | T:C | 2.29835 | 2.02925 |
| 2031012 | Vlasi-1 | H03 | T:T | 0.38422 | 3.68965 |
| 2031012 | Vlasi-1 | A04 | T:T | 0.36454 | 3.55745 |
| 2031012 | Vlasi-1 | B04 | C:C | 3.86605 | 0.46768 |
| 2031012 | Vlasi-1 | C04 | T:C | 2.35934 | 1.95533 |
| 2031012 | Vlasi-1 | D04 | T:C | 2.19747 | 2.0418 |
| 2031012 | Vlasi-1 | E04 | T:T | 0.35744 | 3.61075 |
| 2031012 | Vlasi-1 | F04 | C:C | 4.07131 | 0.46966 |
| 2031012 | Vlasi-1 | G04 | T:C | 2.20894 | 2.10134 |
| 2031012 | Vlasi-1 | H04 | T:C | 2.48428 | 2.13787 |
| 2031012 | Vlasi-1 | A05 | T:T | 0.37105 | 3.58184 |
| 2031012 | Vlasi-1 | B05 | T:T | 0.35778 | 3.60056 |
| 2031012 | Vlasi-1 | C05 | C:C | 4.09281 | 0.48887 |
| 2031012 | Vlasi-1 | D05 | C:C | 3.89995 | 0.43665 |
| 2031012 | Vlasi-1 | E05 | C:C | 3.78442 | 0.45053 |
| 2031012 | Vlasi-1 | F05 | T:C | 2.3202 | 2.06999 |
| 2031012 | Vlasi-1 | G05 | C:C | 4.13849 | 0.47508 |
| 2031012 | Vlasi-1 | H05 | T:T | 0.38228 | 3.6449 |
| 2031012 | Vlasi-1 | A06 | T:T | 0.39657 | 3.7302 |
| 2031012 | Vlasi-1 | B06 | T:T | 0.37605 | 3.69529 |
| 2031012 | Vlasi-1 | C06 | T:C | 2.1356 | 2.258 |
| 2031012 | Vlasi-1 | D06 | C:C | 3.93997 | 0.44455 |
| 2031012 | Vlasi-1 | E06 | T:C | 2.44452 | 1.91121 |
| 2031012 | Vlasi-1 | F06 | T:C | 2.2434 | 2.24809 |
| 2031012 | Vlasi-1 | G06 | T:C | 2.34228 | 2.00777 |
| 2031012 | Vlasi-1 | H06 | T:T | 0.35847 | 3.51326 |
| 2031012 | Vlasi-1 | A07 | C:C | 3.79591 | 0.52839 |
| 2031012 | Vlasi-1 | B07 | T:C | 2.48664 | 2.1054 |
| 2031012 | Vlasi-1 | C07 | T:C | 2.3945 | 2.16953 |
| 2031012 | Vlasi-1 | D07 | C:C | 3.79904 | 0.45449 |
| 2031012 | Vlasi-1 | E07 | T:C | 2.424 | 2.15505 |
| 2031012 | Vlasi-1 | F07 | C:C | 3.85193 | 0.46708 |
| 2031012 | Vlasi-1 | G07 | C:C | 3.97613 | 0.47869 |
| 2031012 | Vlasi-1 | H07 | T:T | 0.37427 | 3.83989 |
| 2031012 | Vlasi-1 | A08 | T:C | 2.26491 | 2.10507 |
| 2031012 | Vlasi-1 | B08 | T:C | 2.3018 | 2.1775 |
| 2031012 | Vlasi-1 | C08 | C:C | 3.99244 | 0.46681 |
| 2031012 | Vlasi-1 | D08 | T:C | 2.47472 | 1.95904 |
| 2031012 | Vlasi-1 | E08 | T:C | 2.26223 | 2.06555 |
| 2031012 | Vlasi-1 | F08 | T:T | 0.36595 | 3.75706 |
| 2031012 | Vlasi-1 | G08 | T:T | 0.36756 | 3.83205 |
| 2031012 | Vlasi-1 | H08 | T:C | 2.41316 | 2.12493 |
| 2031012 | Vlasi-1 | A09 | T:C | 2.2177 | 2.3453 |
| 2031012 | Vlasi-1 | B09 | T:T | 0.37784 | 3.62755 |
| 2031012 | Vlasi-1 | C09 | C:C | 3.81692 | 0.42651 |
| 2031012 | Vlasi-1 | D09 | C:C | 3.94602 | 0.49421 |
| 2031012 | Vlasi-1 | E09 | C:C | 3.88251 | 0.42388 |

| | | | | | |
|---|---|---|---|---|---|
| 2031012 | Vlasi-1 | F09 | T:T | 0.36207 | 3.64762 |
| 2031012 | Vlasi-1 | G09 | T:C | 2.25868 | 2.09498 |
| 2031012 | Vlasi-1 | H09 | C:C | 3.99383 | 0.47312 |
| 2031012 | Vlasi-1 | A10 | C:C | 3.85318 | 0.42827 |
| 2031012 | Vlasi-1 | B10 | T:C | 2.21132 | 2.32353 |
| 2031012 | Vlasi-1 | C10 | T:C | 2.20791 | 2.12292 |
| 2031012 | Vlasi-1 | D10 | C:C | 3.74648 | 0.39498 |
| 2031012 | Vlasi-1 | E10 | C:C | 3.97784 | 0.46333 |
| 2031012 | Vlasi-1 | F10 | C:C | 4.08019 | 0.45089 |
| 2031012 | Vlasi-1 | G10 | T:T | 0.35385 | 3.53376 |
| 2031012 | Vlasi-1 | H10 | C:C | 3.93452 | 0.47176 |
| 2031012 | Vlasi-1 | A11 | T:C | 2.51256 | 1.80567 |
| 2031012 | Vlasi-1 | B11 | T:C | 2.36157 | 2.23133 |
| 2031012 | Vlasi-1 | C11 | T:C | 2.38252 | 2.26093 |
| 2031012 | Vlasi-1 | D11 | T:C | 2.41432 | 2.1679 |
| 2031012 | Vlasi-1 | E11 | T:C | 2.23619 | 2.13347 |
| 2031012 | Vlasi-1 | F11 | T:T | 0.38536 | 3.98488 |
| 2031012 | Vlasi-1 | G11 | T:T | 0.37873 | 3.64178 |
| 2031012 | Vlasi-1 | H11 | T:C | 2.38683 | 2.34937 |
| 2031012 | Vlasi-1 | A12 | T:C | 2.36174 | 2.23287 |
| 2031012 | Vlasi-1 | B12 | T:T | 0.36608 | 3.68248 |
| 2031012 | Vlasi-1 | C12 | T:C | 2.47283 | 2.34366 |
| 2031012 | Vlasi-1 | D12 | T:T | 0.37545 | 3.70194 |
| 2031012 | Vlasi-1 | E12 | T:T | 0.37678 | 3.79428 |
| 2031012 | Vlasi-1 | F12 | T:C | 2.45143 | 2.25488 |
| 2031012 | Vlasi-1 | G12 | T:T | 0.37207 | 3.62402 |
| 2031012 | Vlasi-1 | H12 | T:C | 2.51456 | 2.16057 |
| 2031012 | Vlasi-2 | A01 | T:T | 0.37886 | 3.72435 |
| 2031012 | Vlasi-2 | B01 | C:C | 3.94698 | 0.43615 |
| 2031012 | Vlasi-2 | C01 | T:C | 2.40161 | 2.15659 |
| 2031012 | Vlasi-2 | D01 | C:C | 4.01805 | 0.44663 |
| 2031012 | Vlasi-2 | E01 | C:C | 3.9421 | 0.43198 |
| 2031012 | Vlasi-2 | F01 | C:C | 3.82789 | 0.4094 |
| 2031012 | Vlasi-2 | G01 | T:C | 2.51266 | 2.05976 |
| 2031012 | Vlasi-2 | H01 | T:C | 2.39246 | 2.17011 |
| 2031012 | Vlasi-2 | A02 | T:T | 0.37189 | 3.56648 |
| 2031012 | Vlasi-2 | B02 | C:C | 4.0601 | 0.41175 |
| 2031012 | Vlasi-2 | C02 | T:T | 0.37381 | 3.74048 |
| 2031012 | Vlasi-2 | D02 | T:C | 2.17165 | 2.12047 |
| 2031012 | Vlasi-2 | E02 | C:C | 3.8946 | 0.48117 |
| 2031012 | Vlasi-2 | F02 | T:T | 0.36994 | 3.70315 |
| 2031012 | Vlasi-2 | G02 | T:T | 0.3785 | 3.77818 |
| 2031012 | Vlasi-2 | H02 | T:C | 2.42826 | 2.03653 |
| 2031012 | Vlasi-2 | A03 | C:C | 3.92298 | 0.52529 |
| 2031012 | Vlasi-2 | B03 | C:C | 3.9151 | 0.45305 |
| 2031012 | Vlasi-2 | C03 | T:C | 2.42329 | 2.22947 |
| 2031012 | Vlasi-2 | D03 | T:C | 2.4731 | 2.09946 |
| 2031012 | Vlasi-2 | E03 | T:T | 0.3682 | 3.75884 |
| 2031012 | Vlasi-2 | F03 | T:C | 2.17879 | 1.95848 |
| 2031012 | Vlasi-2 | G03 | T:C | 2.44323 | 1.94979 |

| | | | | | |
|---|---|---|---|---|---|
| 2031012 | Vlasi-2 | H03 | T:C | 2.33031 | 2.20446 |
| 2031012 | Vlasi-2 | A04 | T:C | 2.42195 | 2.21168 |
| 2031012 | Vlasi-2 | B04 | T:C | 2.34561 | 2.10117 |
| 2031012 | Vlasi-2 | C04 | T:T | 0.35543 | 3.60419 |
| 2031012 | Vlasi-2 | D04 | T:C | 2.37109 | 2.23144 |
| 2031012 | Vlasi-2 | E04 | T:T | 0.40973 | 3.86048 |
| 2031012 | Vlasi-2 | F04 | T:C | 2.35732 | 2.18054 |
| 2031012 | Vlasi-2 | G04 | T:T | 0.42331 | 4.01285 |
| 2031012 | Vlasi-2 | H04 | T:T | 0.37575 | 3.62759 |
| 2031012 | Vlasi-2 | A05 | T:C | 2.38955 | 2.24296 |
| 2031012 | Vlasi-2 | B05 | T:C | 2.23322 | 2.21534 |
| 2031012 | Vlasi-2 | C05 | T:C | 2.34339 | 2.27696 |
| 2031012 | Vlasi-2 | D05 | T:C | 2.39475 | 2.06415 |
| 2031012 | Vlasi-2 | E05 | T:T | 0.35962 | 3.6386 |
| 2031012 | Vlasi-2 | F05 | T:C | 2.30802 | 2.24239 |
| 2031012 | Vlasi-2 | G05 | C:C | 3.92172 | 0.45993 |
| 2031012 | Vlasi-2 | H05 | T:C | 2.59539 | 2.23279 |
| 2031012 | Vlasi-2 | A06 | T:T | 0.37588 | 3.64891 |
| 2031012 | Vlasi-2 | B06 | T:C | 2.52437 | 2.05858 |
| 2031012 | Vlasi-2 | C06 | T:C | 2.3542 | 2.23183 |
| 2031012 | Vlasi-2 | D06 | T:C | 2.36977 | 2.20928 |
| 2031012 | Vlasi-2 | E06 | T:T | 0.37796 | 3.62529 |
| 2031012 | Vlasi-2 | F06 | T:T | 0.37676 | 3.66475 |
| 2031012 | Vlasi-2 | G06 | T:C | 2.49067 | 1.99948 |
| 2031012 | Vlasi-2 | H06 | T:C | 2.43478 | 2.15682 |
| 2031012 | Vlasi-2 | A07 | T:T | 0.36498 | 3.54509 |
| 2031012 | Vlasi-2 | B07 | T:C | 2.3055 | 2.27315 |
| 2031012 | Vlasi-2 | C07 | T:T | 0.37385 | 3.75846 |
| 2031012 | Vlasi-2 | D07 | C:C | 3.87649 | 0.46171 |
| 2031012 | Vlasi-2 | E07 | T:C | 2.34439 | 2.26142 |
| 2031012 | Vlasi-2 | F07 | C:C | 3.83935 | 0.44608 |
| 2031012 | Vlasi-2 | G07 | T:C | 2.4781 | 2.21635 |
| 2031012 | Vlasi-2 | H07 | T:T | 0.3776 | 3.79258 |
| 2031012 | Vlasi-2 | A08 | C:C | 3.96011 | 0.50935 |
| 2031012 | Vlasi-2 | B08 | T:C | 2.3789 | 2.07202 |
| 2031012 | Vlasi-2 | C08 | T:T | 0.39512 | 3.60428 |
| 2031012 | Vlasi-2 | D08 | T:C | 2.16031 | 2.12206 |
| 2031012 | Vlasi-2 | E08 | C:C | 3.92789 | 0.46107 |
| 2031012 | Vlasi-2 | F08 | T:C | 2.33362 | 1.97266 |
| 2031012 | Vlasi-2 | G08 | T:T | 0.37174 | 3.54069 |
| 2031012 | Vlasi-2 | H08 | T:T | 0.38746 | 3.78054 |
| 2031012 | Vlasi-2 | A09 | T:C | 2.30868 | 2.16646 |
| 2031012 | Vlasi-2 | B09 | T:T | 0.4114 | 3.88518 |
| 2031012 | Vlasi-2 | C09 | T:T | 0.39281 | 3.87739 |
| 2031012 | Vlasi-2 | D09 | T:C | 2.33141 | 2.14804 |
| 2031012 | Vlasi-2 | E09 | T:C | 2.42595 | 2.13849 |
| 2031012 | Vlasi-2 | F09 | T:T | 0.361 | 3.81638 |
| 2031012 | Vlasi-2 | G09 | T:T | 0.38506 | 3.89271 |
| 2031012 | Vlasi-2 | H09 | C:C | 4.03502 | 0.4449 |
| 2031012 | Vlasi-2 | A10 | T:C | 2.37611 | 2.19387 |

| | | | | | |
|---|---|---|---|---|---|
| 2031012 | Vlasi-2 | B10 | C:C | 4.12078 | 0.43753 |
| 2031012 | Vlasi-2 | C10 | T:C | 2.42471 | 2.23409 |
| 2031012 | Vlasi-2 | D10 | T:C | 2.174 | 2.17825 |
| 2031012 | Vlasi-2 | E10 | T:C | 2.23377 | 2.15479 |
| 2031012 | Vlasi-2 | F10 | C:C | 4.11059 | 0.44607 |
| 2031012 | Vlasi-2 | G10 | T:T | 0.37977 | 3.70553 |
| 2031012 | Vlasi-2 | H10 | T:C | 2.46802 | 2.12178 |
| 2031012 | Vlasi-2 | A11 | ? | 0.53779 | 0.45498 |
| 2031012 | Vlasi-2 | B11 | T:T | 0.3814 | 3.87829 |
| 2031012 | Vlasi-2 | C11 | T:T | 0.36517 | 3.6415 |
| 2031012 | Vlasi-2 | D11 | T:C | 2.28618 | 2.24255 |
| 2031012 | Vlasi-2 | E11 | T:C | 2.43486 | 2.17561 |
| 2031012 | Vlasi-2 | F11 | C:C | 4.29064 | 0.45792 |
| 2031012 | Vlasi-2 | G11 | C:C | 3.93247 | 0.45838 |
| 2031012 | Vlasi-2 | H11 | T:C | 2.33343 | 2.27683 |
| 2031012 | Vlasi-2 | A12 | T:C | 2.17731 | 2.30246 |
| 2031012 | Vlasi-2 | B12 | C:C | 4.00137 | 0.49141 |
| 2031012 | Vlasi-2 | C12 | T:T | 0.41389 | 3.72838 |
| 2031012 | Vlasi-2 | D12 | C:C | 3.95386 | 0.51469 |
| 2031012 | Vlasi-2 | E12 | T:C | 2.50023 | 2.44905 |
| 2031012 | Vlasi-2 | F12 | T:T | 0.41896 | 3.69008 |
| 2031012 | Vlasi-2 | G12 | T:C | 2.42227 | 2.16653 |
| 2031012 | Vlasi-2 | H12 | T:C | 2.4739 | 2.26472 |
| 2031012 | Vlasi-3 | A01 | T:T | 0.35649 | 3.81782 |
| 2031012 | Vlasi-3 | B01 | T:C | 2.28655 | 2.22388 |
| 2031012 | Vlasi-3 | C01 | T:T | 0.35516 | 3.68061 |
| 2031012 | Vlasi-3 | D01 | T:C | 2.57045 | 1.8712 |
| 2031012 | Vlasi-3 | E01 | T:C | 2.33191 | 2.10436 |
| 2031012 | Vlasi-3 | F01 | T:T | 0.34176 | 3.84316 |
| 2031012 | Vlasi-3 | G01 | T:C | 2.32164 | 2.20026 |
| 2031012 | Vlasi-3 | H01 | T:C | 2.43195 | 2.22996 |
| 2031012 | Vlasi-3 | A02 | T:C | 2.33338 | 2.44036 |
| 2031012 | Vlasi-3 | B02 | T:C | 2.30441 | 2.27291 |
| 2031012 | Vlasi-3 | C02 | T:C | 2.14905 | 2.09072 |
| 2031012 | Vlasi-3 | D02 | T:C | 2.33037 | 2.14479 |
| 2031012 | Vlasi-3 | E02 | T:C | 2.37614 | 2.1294 |
| 2031012 | Vlasi-3 | F02 | T:C | 2.37557 | 1.99527 |
| 2031012 | Vlasi-3 | G02 | T:T | 0.38397 | 3.65017 |
| 2031012 | Vlasi-3 | H02 | T:T | 0.40886 | 3.66871 |
| 2031012 | Vlasi-3 | A03 | T:C | 2.30458 | 2.18959 |
| 2031012 | Vlasi-3 | B03 | T:C | 2.42492 | 2.25551 |
| 2031012 | Vlasi-3 | C03 | C:C | 3.79376 | 0.43855 |
| 2031012 | Vlasi-3 | D03 | C:C | 3.77159 | 0.45258 |
| 2031012 | Vlasi-3 | E03 | T:C | 2.43054 | 1.92142 |
| 2031012 | Vlasi-3 | F03 | C:C | 3.87604 | 0.40943 |
| 2031012 | Vlasi-3 | G03 | T:C | 2.40693 | 2.06875 |
| 2031012 | Vlasi-3 | H03 | T:C | 2.21707 | 2.43683 |
| 2031012 | Vlasi-3 | A04 | T:C | 2.36308 | 1.96807 |
| 2031012 | Vlasi-3 | B04 | T:C | 2.40842 | 2.20636 |
| 2031012 | Vlasi-3 | C04 | T:T | 0.36848 | 3.46839 |

| | | | | | |
|---|---|---|---|---|---|
| 2031012 | Vlasi-3 | D04 | T:C | 2.25806 | 2.15788 |
| 2031012 | Vlasi-3 | E04 | T:C | 2.28151 | 2.10664 |
| 2031012 | Vlasi-3 | F04 | T:T | 0.38121 | 3.86896 |
| 2031012 | Vlasi-3 | G04 | C:C | 4.01295 | 0.422 |
| 2031012 | Vlasi-3 | H04 | C:C | 3.92514 | 0.5193 |
| 2031012 | Vlasi-3 | A05 | C:C | 3.87036 | 0.43822 |
| 2031012 | Vlasi-3 | B05 | C:C | 3.87273 | 0.49985 |
| 2031012 | Vlasi-3 | C05 | T:T | 0.37298 | 3.72616 |
| 2031012 | Vlasi-3 | D05 | C:C | 3.95634 | 0.45422 |
| 2031012 | Vlasi-3 | E05 | C:C | 3.99052 | 0.43191 |
| 2031012 | Vlasi-3 | F05 | T:C | 2.23534 | 2.19665 |
| 2031012 | Vlasi-3 | G05 | T:T | 0.40085 | 3.88455 |
| 2031012 | Vlasi-3 | H05 | T:C | 2.42097 | 2.29424 |
| 2031012 | Vlasi-3 | A06 | T:T | 0.38875 | 3.74746 |
| 2031012 | Vlasi-3 | B06 | T:C | 2.37813 | 2.16017 |
| 2031012 | Vlasi-3 | C06 | T:T | 0.36341 | 3.57538 |
| 2031012 | Vlasi-3 | D06 | C:C | 3.96595 | 0.43992 |
| 2031012 | Vlasi-3 | E06 | T:T | 0.3679 | 3.88237 |
| 2031012 | Vlasi-3 | F06 | C:C | 3.96127 | 0.46592 |
| 2031012 | Vlasi-3 | G06 | T:C | 2.24656 | 2.21189 |
| 2031012 | Vlasi-3 | H06 | T:C | 2.38231 | 2.39215 |
| 2031012 | Vlasi-3 | A07 | C:C | 3.85596 | 0.43462 |
| 2031012 | Vlasi-3 | B07 | T:C | 2.41028 | 2.10809 |
| 2031012 | Vlasi-3 | C07 | T:C | 2.27266 | 1.96353 |
| 2031012 | Vlasi-3 | D07 | T:C | 2.18926 | 2.1344 |
| 2031012 | Vlasi-3 | E07 | C:C | 3.88985 | 0.44902 |
| 2031012 | Vlasi-3 | F07 | T:C | 2.21333 | 2.2105 |
| 2031012 | Vlasi-3 | G07 | C:C | 3.86264 | 0.50587 |
| 2031012 | Vlasi-3 | H07 | T:C | 2.38412 | 2.33466 |
| 2031012 | Vlasi-3 | A08 | T:T | 0.3749 | 3.77852 |
| 2031012 | Vlasi-3 | B08 | T:C | 2.35699 | 2.11559 |
| 2031012 | Vlasi-3 | C08 | T:C | 2.32998 | 1.98964 |
| 2031012 | Vlasi-3 | D08 | T:C | 2.25861 | 2.12199 |
| 2031012 | Vlasi-3 | E08 | T:T | 0.33984 | 3.5405 |
| 2031012 | Vlasi-3 | F08 | T:C | 2.4556 | 2.09554 |
| 2031012 | Vlasi-3 | G08 | T:T | 0.37335 | 3.7331 |
| 2031012 | Vlasi-3 | H08 | T:T | 0.40697 | 3.7757 |
| 2031012 | Vlasi-3 | A09 | C:C | 3.83984 | 0.45167 |
| 2031012 | Vlasi-3 | B09 | C:C | 3.83869 | 0.44836 |
| 2031012 | Vlasi-3 | C09 | T:C | 2.3588 | 2.08922 |
| 2031012 | Vlasi-3 | D09 | T:T | 0.37093 | 3.61215 |
| 2031012 | Vlasi-3 | E09 | T:C | 2.43934 | 2.15465 |
| 2031012 | Vlasi-3 | F09 | T:C | 2.30629 | 2.18552 |
| 2031012 | Vlasi-3 | G09 | T:C | 2.43192 | 2.03831 |
| 2031012 | Vlasi-3 | H09 | T:C | 2.38539 | 2.36222 |
| 2031012 | Vlasi-3 | A10 | T:C | 2.41381 | 2.08737 |
| 2031012 | Vlasi-3 | B10 | T:T | 0.36417 | 3.7682 |
| 2031012 | Vlasi-3 | C10 | T:C | 2.27647 | 2.19101 |
| 2031012 | Vlasi-3 | D10 | T:C | 2.2361 | 2.26182 |
| 2031012 | Vlasi-3 | E10 | C:C | 4.10561 | 0.46386 |

| | | | | | |
|---|---|---|---|---|---|
| 2031012 | Vlasi-3 | F10 | T:C | 2.38373 | 1.98686 |
| 2031012 | Vlasi-3 | G10 | T:T | 0.3776 | 3.76719 |
| 2031012 | Vlasi-3 | H10 | T:T | 0.41704 | 3.84209 |
| 2031012 | Vlasi-3 | A11 | T:T | 0.37669 | 3.74407 |
| 2031012 | Vlasi-3 | B11 | T:C | 2.55644 | 2.15015 |
| 2031012 | Vlasi-3 | C11 | T:T | 0.35604 | 3.73618 |
| 2031012 | Vlasi-3 | D11 | T:T | 0.37963 | 3.8485 |
| 2031012 | Vlasi-3 | E11 | T:C | 2.24559 | 2.12913 |
| 2031012 | Vlasi-3 | F11 | C:C | 4.08735 | 0.483 |
| 2031012 | Vlasi-3 | G11 | C:C | 4.06511 | 0.47949 |
| 2031012 | Vlasi-3 | H11 | C:C | 3.95601 | 0.513 |
| 2031012 | Vlasi-3 | A12 | T:T | 0.37838 | 3.7793 |
| 2031012 | Vlasi-3 | B12 | T:C | 2.23208 | 2.27316 |
| 2031012 | Vlasi-3 | C12 | T:C | 2.1479 | 2.52244 |
| 2031012 | Vlasi-3 | D12 | T:T | 0.3759 | 3.71228 |
| 2031012 | Vlasi-3 | E12 | T:T | 0.36409 | 3.65534 |
| 2031012 | Vlasi-3 | F12 | T:T | 0.37786 | 3.64025 |
| 2031012 | Vlasi-3 | G12 | T:C | 2.20159 | 2.32848 |
| 2031012 | Vlasi-3 | H12 | T:T | 0.60619 | 3.5516 |
| 2031012 | Vlasi-4 | A01 | C:C | 3.96946 | 0.42684 |
| 2031012 | Vlasi-4 | B01 | T:C | 2.24406 | 2.05378 |
| 2031012 | Vlasi-4 | C01 | T:C | 2.34832 | 1.99285 |
| 2031012 | Vlasi-4 | D01 | T:C | 2.29566 | 2.08718 |
| 2031012 | Vlasi-4 | E01 | T:C | 2.29711 | 1.91757 |
| 2031012 | Vlasi-4 | F01 | T:T | 0.37608 | 3.74138 |
| 2031012 | Vlasi-4 | G01 | T:C | 2.47559 | 2.00093 |
| 2031012 | Vlasi-4 | H01 | T:T | 0.40686 | 3.63652 |
| 2031012 | Vlasi-4 | A02 | T:C | 2.36994 | 2.17799 |
| 2031012 | Vlasi-4 | B02 | T:C | 2.27902 | 2.07436 |
| 2031012 | Vlasi-4 | C02 | T:C | 2.39989 | 2.03065 |
| 2031012 | Vlasi-4 | D02 | T:T | 0.39002 | 3.82288 |
| 2031012 | Vlasi-4 | E02 | T:C | 2.42463 | 2.00845 |
| 2031012 | Vlasi-4 | F02 | T:T | 0.38112 | 3.72825 |
| 2031012 | Vlasi-4 | G02 | C:C | 3.84979 | 0.44565 |
| 2031012 | Vlasi-4 | H02 | C:C | 4.05217 | 0.49794 |
| 2031012 | Vlasi-4 | A03 | T:T | 0.37887 | 3.8261 |
| 2031012 | Vlasi-4 | B03 | C:C | 3.89844 | 0.44361 |
| 2031012 | Vlasi-4 | C03 | T:T | 0.35705 | 3.77877 |
| 2031012 | Vlasi-4 | D03 | C:C | 3.85112 | 0.45523 |
| 2031012 | Vlasi-4 | E03 | T:C | 2.29259 | 2.13736 |
| 2031012 | Vlasi-4 | F03 | C:C | 3.77935 | 0.43353 |
| 2031012 | Vlasi-4 | G03 | T:C | 2.4216 | 2.06385 |
| 2031012 | Vlasi-4 | H03 | T:T | 0.38704 | 3.85786 |
| 2031012 | Vlasi-4 | A04 | T:T | 0.36081 | 3.72178 |
| 2031012 | Vlasi-4 | B04 | C:C | 3.85694 | 0.4207 |
| 2031012 | Vlasi-4 | C04 | T:T | 0.37156 | 3.62537 |
| 2031012 | Vlasi-4 | D04 | T:T | 0.37013 | 3.80543 |
| 2031012 | Vlasi-4 | E04 | T:T | 0.36566 | 3.6298 |
| 2031012 | Vlasi-4 | F04 | T:T | 0.35605 | 3.50674 |
| 2031012 | Vlasi-4 | G04 | T:T | 0.38981 | 3.66972 |

| | | | | | |
|---|---|---|---|---|---|
| 2031012 | Vlasi-4 | H04 | T:C | 2.48171 | 2.113 |
| 2031012 | Vlasi-4 | A05 | T:T | 0.36764 | 3.54154 |
| 2031012 | Vlasi-4 | B05 | T:C | 2.40664 | 1.89738 |
| 2031012 | Vlasi-4 | C05 | T:C | 2.42423 | 2.02134 |
| 2031012 | Vlasi-4 | D05 | T:T | 0.35911 | 3.5784 |
| 2031012 | Vlasi-4 | E05 | T:T | 0.3694 | 3.73667 |
| 2031012 | Vlasi-4 | F05 | C:C | 3.84271 | 0.47244 |
| 2031012 | Vlasi-4 | G05 | T:C | 2.1989 | 2.09192 |
| 2031012 | Vlasi-4 | H05 | C:C | 3.77582 | 0.44574 |
| 2031012 | Vlasi-4 | A06 | T:C | 2.50135 | 1.83991 |
| 2031012 | Vlasi-4 | B06 | T:C | 2.38665 | 1.97695 |
| 2031012 | Vlasi-4 | C06 | T:T | 0.37548 | 3.59608 |
| 2031012 | Vlasi-4 | D06 | T:C | 2.31256 | 1.95941 |
| 2031012 | Vlasi-4 | E06 | T:C | 2.40671 | 1.88933 |
| 2031012 | Vlasi-4 | F06 | T:T | 0.38598 | 3.87628 |
| 2031012 | Vlasi-4 | G06 | C:C | 3.69578 | 0.42614 |
| 2031012 | Vlasi-4 | H06 | T:C | 2.52911 | 2.07996 |
| 2031012 | Vlasi-4 | A07 | ? | 0.48419 | 1.08564 |
| 2031012 | Vlasi-4 | B07 | ? | 0.54827 | 0.4175 |
| 2031012 | Vlasi-4 | C07 | ? | 0.51278 | 0.40634 |
| 2031012 | Vlasi-4 | D07 | ? | 0.56681 | 0.43109 |
| 2031012 | Vlasi-4 | E07 | ? | 0.53947 | 0.41379 |
| 2031012 | Vlasi-4 | F07 | C:C | 3.71746 | 0.57328 |
| 2031012 | Vlasi-4 | G07 | C:C | 3.84344 | 0.56785 |
| 2031012 | Vlasi-4 | H07 | C:C | 3.76247 | 0.92452 |
| 2031012 | Vlasi-4 | A08 | C:C | 3.62363 | 0.74712 |
| 2031012 | Vlasi-4 | B08 | C:C | 3.53613 | 0.68726 |
| 2031012 | Vlasi-4 | C08 | C:C | 3.76534 | 0.64324 |
| 2031012 | Vlasi-4 | D08 | C:C | 3.74453 | 0.61101 |
| 2031012 | Vlasi-4 | E08 | C:C | 3.77954 | 0.63578 |
| 2031012 | Vlasi-4 | F08 | C:C | 3.88304 | 0.61456 |
| 2031012 | Vlasi-4 | G08 | C:C | 3.74215 | 0.60665 |
| 2031012 | Vlasi-4 | H08 | C:C | 3.6843 | 0.86499 |
| 2031012 | Vlasi-4 | A09 | C:C | 3.62273 | 0.75719 |
| 2031012 | Vlasi-4 | B09 | C:C | 3.59871 | 0.62439 |
| 2031012 | Vlasi-4 | C09 | C:C | 3.70169 | 0.68856 |
| 2031012 | Vlasi-4 | D09 | C:C | 3.60148 | 0.63038 |
| 2031012 | Vlasi-4 | E09 | C:C | 3.70549 | 0.63933 |
| 2031012 | Vlasi-4 | F09 | C:C | 3.82833 | 0.59605 |
| 2031012 | Vlasi-4 | G09 | C:C | 3.43744 | 0.65566 |
| 2031012 | Vlasi-4 | H09 | C:C | 3.53387 | 1.04174 |
| 2031012 | Vlasi-4 | A10 | C:C | 3.69669 | 0.77047 |
| 2031012 | Vlasi-4 | B10 | C:C | 3.73369 | 0.59264 |
| 2031012 | Vlasi-4 | C10 | T:T | 0.36891 | 3.54866 |
| 2031012 | Vlasi-4 | D10 | T:T | 0.37853 | 3.72671 |
| 2031012 | Vlasi-4 | E10 | T:T | 0.47787 | 3.73683 |
| 2031012 | Vlasi-4 | F10 | T:T | 0.4962 | 3.60863 |
| 2031012 | Vlasi-4 | G10 | T:T | 0.42899 | 3.56368 |
| 2031012 | Vlasi-4 | H10 | T:T | 0.65325 | 3.63268 |
| 2031012 | Vlasi-4 | A11 | T:T | 0.54674 | 3.5776 |

| | | | | | |
|---|---|---|---|---|---|
| 2031012 | Vlasi-4 | B11 | T:T | 0.50084 | 3.43338 |
| 2031012 | Vlasi-4 | C11 | T:T | 0.50332 | 3.53351 |
| 2031012 | Vlasi-4 | D11 | T:T | 0.44217 | 3.5507 |
| 2031012 | Vlasi-4 | E11 | T:T | 0.52061 | 3.69406 |
| 2031012 | Vlasi-4 | F11 | T:T | 0.46078 | 3.60184 |
| 2031012 | Vlasi-4 | G11 | T:T | 0.49932 | 3.67781 |
| 2031012 | Vlasi-4 | H11 | T:T | 0.59566 | 3.48768 |
| 2031012 | Vlasi-4 | A12 | T:T | 0.77716 | 3.55527 |
| 2031012 | Vlasi-4 | B12 | T:T | 0.74716 | 3.3902 |
| 2031012 | Vlasi-4 | C12 | T:T | 0.71402 | 3.44842 |
| 2031012 | Vlasi-4 | D12 | T:T | 0.78298 | 3.49388 |
| 2031012 | Vlasi-4 | E12 | T:T | 0.71343 | 3.67443 |
| 2031012 | Vlasi-4 | F12 | T:T | 0.61319 | 3.61213 |
| 2031012 | Vlasi-4 | G12 | T:T | 0.64089 | 3.71078 |
| 2031012 | Vlasi-4 | H12 | NTC | 0.63826 | 0.56011 |
| 2031014 | Vlasi-1 | A01 | T:C | 1.70132 | 2.15649 |
| 2031014 | Vlasi-1 | B01 | T:C | 1.80485 | 2.03867 |
| 2031014 | Vlasi-1 | C01 | T:T | 0.3816 | 3.4543 |
| 2031014 | Vlasi-1 | D01 | T:C | 1.70891 | 1.79624 |
| 2031014 | Vlasi-1 | E01 | T:C | 1.73506 | 1.82341 |
| 2031014 | Vlasi-1 | F01 | T:T | 0.42277 | 3.63144 |
| 2031014 | Vlasi-1 | G01 | T:T | 0.40722 | 3.59878 |
| 2031014 | Vlasi-1 | H01 | T:C | 1.73669 | 1.94892 |
| 2031014 | Vlasi-1 | A02 | T:C | 1.42159 | 1.81486 |
| 2031014 | Vlasi-1 | B02 | T:T | 0.45852 | 3.30371 |
| 2031014 | Vlasi-1 | C02 | T:C | 1.65952 | 2.05009 |
| 2031014 | Vlasi-1 | D02 | T:T | 0.44712 | 3.76062 |
| 2031014 | Vlasi-1 | E02 | T:C | 1.66451 | 1.94691 |
| 2031014 | Vlasi-1 | F02 | T:C | 1.59217 | 1.89745 |
| 2031014 | Vlasi-1 | G02 | T:T | 0.44904 | 3.61396 |
| 2031014 | Vlasi-1 | H02 | T:C | 1.62128 | 1.72677 |
| 2031014 | Vlasi-1 | A03 | T:T | 0.44404 | 3.44335 |
| 2031014 | Vlasi-1 | B03 | ? | 0.7539 | 0.42513 |
| 2031014 | Vlasi-1 | C03 | T:T | 0.41779 | 3.42343 |
| 2031014 | Vlasi-1 | D03 | T:T | 0.43829 | 3.59166 |
| 2031014 | Vlasi-1 | E03 | T:C | 1.64575 | 1.78336 |
| 2031014 | Vlasi-1 | F03 | ? | 0.49266 | 2.36127 |
| 2031014 | Vlasi-1 | G03 | C:C | 3.42471 | 0.43253 |
| 2031014 | Vlasi-1 | H03 | T:C | 1.52596 | 1.87783 |
| 2031014 | Vlasi-1 | A04 | C:C | 3.01575 | 0.40759 |
| 2031014 | Vlasi-1 | B04 | T:C | 1.34697 | 1.75981 |
| 2031014 | Vlasi-1 | C04 | T:C | 1.70406 | 1.83134 |
| 2031014 | Vlasi-1 | D04 | T:T | 0.6384 | 3.16274 |
| 2031014 | Vlasi-1 | E04 | C:C | 3.53262 | 0.43488 |
| 2031014 | Vlasi-1 | F04 | T:C | 1.60906 | 1.95224 |
| 2031014 | Vlasi-1 | G04 | T:C | 1.83299 | 1.72404 |
| 2031014 | Vlasi-1 | H04 | T:C | 1.57908 | 1.71349 |
| 2031014 | Vlasi-1 | A05 | T:C | 1.56213 | 1.87903 |
| 2031014 | Vlasi-1 | B05 | ? | 0.87598 | 1.68445 |
| 2031014 | Vlasi-1 | C05 | T:T | 0.54348 | 3.2718 |

| | | | | | |
|---|---|---|---|---|---|
| 2031014 | Vlasi-1 | D05 | T:C | 1.44811 | 1.89188 |
| 2031014 | Vlasi-1 | E05 | T:C | 1.55675 | 1.76938 |
| 2031014 | Vlasi-1 | F05 | ? | 1.08328 | 0.43769 |
| 2031014 | Vlasi-1 | G05 | T:C | 1.33769 | 1.63048 |
| 2031014 | Vlasi-1 | H05 | C:C | 3.54771 | 0.47035 |
| 2031014 | Vlasi-1 | A06 | T:T | 0.45818 | 3.23064 |
| 2031014 | Vlasi-1 | B06 | T:C | 1.55175 | 1.84408 |
| 2031014 | Vlasi-1 | C06 | T:T | 0.44846 | 3.56882 |
| 2031014 | Vlasi-1 | D06 | C:C | 3.4361 | 0.43149 |
| 2031014 | Vlasi-1 | E06 | T:C | 1.53499 | 1.6515 |
| 2031014 | Vlasi-1 | F06 | T:T | 0.44266 | 3.51615 |
| 2031014 | Vlasi-1 | G06 | T:C | 1.4884 | 1.78346 |
| 2031014 | Vlasi-1 | H06 | T:T | 0.41874 | 3.40992 |
| 2031014 | Vlasi-1 | A07 | C:C | 2.89604 | 0.4311 |
| 2031014 | Vlasi-1 | B07 | T:T | 0.52938 | 3.48595 |
| 2031014 | Vlasi-1 | C07 | C:C | 3.52465 | 0.42493 |
| 2031014 | Vlasi-1 | D07 | ? | 0.6286 | 0.45766 |
| 2031014 | Vlasi-1 | E07 | T:T | 0.53674 | 3.23632 |
| 2031014 | Vlasi-1 | F07 | T:C | 1.27172 | 1.97756 |
| 2031014 | Vlasi-1 | G07 | T:T | 0.42473 | 3.62595 |
| 2031014 | Vlasi-1 | H07 | T:T | 0.44632 | 3.45486 |
| 2031014 | Vlasi-1 | A08 | T:T | 0.43496 | 2.93333 |
| 2031014 | Vlasi-1 | B08 | T:T | 0.42206 | 3.34394 |
| 2031014 | Vlasi-1 | C08 | T:T | 0.42879 | 3.34303 |
| 2031014 | Vlasi-1 | D08 | T:T | 0.44463 | 3.5173 |
| 2031014 | Vlasi-1 | E08 | T:T | 0.40566 | 3.41337 |
| 2031014 | Vlasi-1 | F08 | T:C | 1.57851 | 1.7512 |
| 2031014 | Vlasi-1 | G08 | T:T | 0.50645 | 3.71191 |
| 2031014 | Vlasi-1 | H08 | T:T | 0.44205 | 3.56282 |
| 2031014 | Vlasi-1 | A09 | T:T | 0.45674 | 2.81122 |
| 2031014 | Vlasi-1 | B09 | T:T | 0.44571 | 3.60081 |
| 2031014 | Vlasi-1 | C09 | T:T | 0.49171 | 3.52515 |
| 2031014 | Vlasi-1 | D09 | T:C | 1.43031 | 1.74612 |
| 2031014 | Vlasi-1 | E09 | T:T | 0.41214 | 3.51393 |
| 2031014 | Vlasi-1 | F09 | T:T | 0.48026 | 3.44588 |
| 2031014 | Vlasi-1 | G09 | C:C | 3.29517 | 0.49466 |
| 2031014 | Vlasi-1 | H09 | T:C | 1.58936 | 1.78861 |
| 2031014 | Vlasi-1 | A10 | T:T | 0.44227 | 3.26221 |
| 2031014 | Vlasi-1 | B10 | T:C | 1.3362 | 1.94174 |
| 2031014 | Vlasi-1 | C10 | T:T | 0.3749 | 3.09145 |
| 2031014 | Vlasi-1 | D10 | T:C | 1.49793 | 1.9044 |
| 2031014 | Vlasi-1 | E10 | T:T | 0.42474 | 3.442 |
| 2031014 | Vlasi-1 | F10 | T:T | 0.4177 | 3.38527 |
| 2031014 | Vlasi-1 | G10 | T:C | 1.58535 | 1.75962 |
| 2031014 | Vlasi-1 | H10 | T:C | 1.42438 | 1.75358 |
| 2031014 | Vlasi-1 | A11 | T:C | 1.47123 | 1.98961 |
| 2031014 | Vlasi-1 | B11 | T:T | 0.39744 | 3.44276 |
| 2031014 | Vlasi-1 | C11 | T:T | 0.4222 | 3.50809 |
| 2031014 | Vlasi-1 | D11 | T:T | 0.43618 | 3.589 |
| 2031014 | Vlasi-1 | E11 | C:C | 3.45533 | 0.43623 |

| | | | | | |
|---|---|---|---|---|---|
| 2031014 | Vlasi-1 | F11 | T:C | 1.44238 | 1.94109 |
| 2031014 | Vlasi-1 | G11 | T:C | 1.52335 | 1.88696 |
| 2031014 | Vlasi-1 | H11 | T:T | 0.43512 | 3.53173 |
| 2031014 | Vlasi-1 | A12 | T:C | 1.4599 | 1.96378 |
| 2031014 | Vlasi-1 | B12 | T:C | 1.50892 | 1.90926 |
| 2031014 | Vlasi-1 | C12 | C:C | 3.43169 | 0.43696 |
| 2031014 | Vlasi-1 | D12 | T:C | 1.38356 | 1.8861 |
| 2031014 | Vlasi-1 | E12 | T:T | 0.40337 | 3.54449 |
| 2031014 | Vlasi-1 | F12 | T:T | 0.42865 | 3.39761 |
| 2031014 | Vlasi-1 | G12 | T:T | 0.42692 | 3.45777 |
| 2031014 | Vlasi-1 | H12 | T:C | 1.53269 | 1.70096 |
| 2031014 | Vlasi-2 | A01 | T:T | 0.44543 | 3.43122 |
| 2031014 | Vlasi-2 | B01 | T:T | 0.4033 | 3.45408 |
| 2031014 | Vlasi-2 | C01 | T:C | 1.77611 | 1.79097 |
| 2031014 | Vlasi-2 | D01 | ? | 0.79064 | 0.48943 |
| 2031014 | Vlasi-2 | E01 | C:C | 3.63899 | 0.45259 |
| 2031014 | Vlasi-2 | F01 | T:T | 0.52557 | 3.64504 |
| 2031014 | Vlasi-2 | G01 | C:C | 3.48309 | 0.39055 |
| 2031014 | Vlasi-2 | H01 | T:C | 1.56938 | 1.96623 |
| 2031014 | Vlasi-2 | A02 | T:T | 0.45311 | 3.27437 |
| 2031014 | Vlasi-2 | B02 | T:T | 0.53949 | 3.5405 |
| 2031014 | Vlasi-2 | C02 | T:T | 0.4164 | 3.44816 |
| 2031014 | Vlasi-2 | D02 | T:C | 1.68415 | 1.85126 |
| 2031014 | Vlasi-2 | E02 | T:C | 1.52132 | 1.7573 |
| 2031014 | Vlasi-2 | F02 | T:C | 1.65692 | 1.87873 |
| 2031014 | Vlasi-2 | G02 | T:T | 0.47135 | 3.56809 |
| 2031014 | Vlasi-2 | H02 | T:T | 0.44553 | 3.67114 |
| 2031014 | Vlasi-2 | A03 | T:T | 0.55273 | 3.33096 |
| 2031014 | Vlasi-2 | B03 | C:C | 3.45688 | 0.43777 |
| 2031014 | Vlasi-2 | C03 | T:T | 0.4616 | 3.35792 |
| 2031014 | Vlasi-2 | D03 | T:T | 0.4537 | 3.75853 |
| 2031014 | Vlasi-2 | E03 | T:C | 1.62172 | 1.90717 |
| 2031014 | Vlasi-2 | F03 | T:C | 1.30371 | 1.72219 |
| 2031014 | Vlasi-2 | G03 | T:C | 1.57326 | 1.89379 |
| 2031014 | Vlasi-2 | H03 | T:C | 1.52006 | 1.78072 |
| 2031014 | Vlasi-2 | A04 | T:T | 0.47294 | 3.19148 |
| 2031014 | Vlasi-2 | B04 | T:C | 1.63561 | 1.83243 |
| 2031014 | Vlasi-2 | C04 | T:C | 1.57345 | 1.67721 |
| 2031014 | Vlasi-2 | D04 | C:C | 3.30868 | 0.40585 |
| 2031014 | Vlasi-2 | E04 | ? | 1.08255 | 0.41338 |
| 2031014 | Vlasi-2 | F04 | ? | 1.71695 | 0.44743 |
| 2031014 | Vlasi-2 | G04 | ? | 1.04797 | 0.444 |
| 2031014 | Vlasi-2 | H04 | T:C | 1.60809 | 1.75579 |
| 2031014 | Vlasi-2 | A05 | T:T | 0.45717 | 3.51499 |
| 2031014 | Vlasi-2 | B05 | T:T | 0.42964 | 3.44655 |
| 2031014 | Vlasi-2 | C05 | T:T | 0.50872 | 3.4097 |
| 2031014 | Vlasi-2 | D05 | T:T | 0.53632 | 3.2044 |
| 2031014 | Vlasi-2 | E05 | T:T | 0.5007 | 3.46802 |
| 2031014 | Vlasi-2 | F05 | T:C | 1.42576 | 1.77967 |
| 2031014 | Vlasi-2 | G05 | T:C | 1.47272 | 1.76154 |

| | | | | | |
|---|---|---|---|---|---|
| 2031014 | Vlasi-2 | H05 | T:T | 0.52292 | 3.56017 |
| 2031014 | Vlasi-2 | A06 | T:T | 0.46652 | 3.45787 |
| 2031014 | Vlasi-2 | B06 | T:T | 0.39292 | 3.292 |
| 2031014 | Vlasi-2 | C06 | ? | 0.89183 | 1.47132 |
| 2031014 | Vlasi-2 | D06 | T:T | 0.43522 | 3.44248 |
| 2031014 | Vlasi-2 | E06 | T:C | 1.61009 | 1.88312 |
| 2031014 | Vlasi-2 | F06 | T:T | 0.42782 | 3.64485 |
| 2031014 | Vlasi-2 | G06 | T:T | 0.46435 | 3.45106 |
| 2031014 | Vlasi-2 | H06 | T:T | 0.42892 | 3.48651 |
| 2031014 | Vlasi-2 | A07 | T:T | 0.42102 | 3.40716 |
| 2031014 | Vlasi-2 | B07 | ? | 0.99506 | 0.42106 |
| 2031014 | Vlasi-2 | C07 | C:C | 3.13838 | 0.44924 |
| 2031014 | Vlasi-2 | D07 | ? | 0.96895 | 0.95639 |
| 2031014 | Vlasi-2 | E07 | T:T | 0.52753 | 3.40868 |
| 2031014 | Vlasi-2 | F07 | T:T | 0.40045 | 3.51998 |
| 2031014 | Vlasi-2 | G07 | T:C | 1.53286 | 1.74507 |
| 2031014 | Vlasi-2 | H07 | T:T | 0.67569 | 2.84993 |
| 2031014 | Vlasi-2 | A08 | T:C | 1.68975 | 1.94033 |
| 2031014 | Vlasi-2 | B08 | T:T | 0.46697 | 3.63602 |
| 2031014 | Vlasi-2 | C08 | ? | 1.25022 | 0.33724 |
| 2031014 | Vlasi-2 | D08 | T:C | 1.53041 | 1.95204 |
| 2031014 | Vlasi-2 | E08 | T:T | 0.51246 | 2.71755 |
| 2031014 | Vlasi-2 | F08 | T:T | 0.41063 | 3.50805 |
| 2031014 | Vlasi-2 | G08 | T:T | 0.45568 | 3.68296 |
| 2031014 | Vlasi-2 | H08 | T:C | 1.56133 | 1.87422 |
| 2031014 | Vlasi-2 | A09 | ? | 0.58246 | 0.39911 |
| 2031014 | Vlasi-2 | B09 | C:C | 3.25386 | 0.45404 |
| 2031014 | Vlasi-2 | C09 | ? | 0.97146 | 0.46255 |
| 2031014 | Vlasi-2 | D09 | T:T | 0.47432 | 3.61465 |
| 2031014 | Vlasi-2 | E09 | ? | 1.19648 | 0.41278 |
| 2031014 | Vlasi-2 | F09 | T:T | 0.45468 | 2.99735 |
| 2031014 | Vlasi-2 | G09 | C:C | 3.22846 | 0.44288 |
| 2031014 | Vlasi-2 | H09 | T:C | 1.67424 | 1.84812 |
| 2031014 | Vlasi-2 | A10 | T:C | 1.4968 | 1.89452 |
| 2031014 | Vlasi-2 | B10 | T:C | 1.52019 | 1.90939 |
| 2031014 | Vlasi-2 | C10 | T:C | 1.5563 | 1.8401 |
| 2031014 | Vlasi-2 | D10 | T:T | 0.4502 | 3.56749 |
| 2031014 | Vlasi-2 | E10 | T:C | 1.50279 | 1.73384 |
| 2031014 | Vlasi-2 | F10 | T:T | 0.45728 | 3.70486 |
| 2031014 | Vlasi-2 | G10 | C:C | 3.05048 | 0.42315 |
| 2031014 | Vlasi-2 | H10 | T:C | 1.57266 | 1.70919 |
| 2031014 | Vlasi-2 | A11 | T:C | 1.8238 | 1.9614 |
| 2031014 | Vlasi-2 | B11 | C:C | 3.28917 | 0.42529 |
| 2031014 | Vlasi-2 | C11 | T:C | 1.55281 | 1.86185 |
| 2031014 | Vlasi-2 | D11 | T:C | 1.43606 | 1.83589 |
| 2031014 | Vlasi-2 | E11 | T:T | 0.43171 | 3.49043 |
| 2031014 | Vlasi-2 | F11 | T:T | 0.41254 | 3.35811 |
| 2031014 | Vlasi-2 | G11 | T:T | 0.42659 | 3.38904 |
| 2031014 | Vlasi-2 | H11 | T:T | 0.42344 | 3.54002 |
| 2031014 | Vlasi-2 | A12 | T:C | 1.68414 | 2.14627 |

| | | | | | |
|---|---|---|---|---|---|
| 2031014 | Vlasi-2 | B12 | T:C | 1.67007 | 1.98295 |
| 2031014 | Vlasi-2 | C12 | T:C | 1.69546 | 1.97262 |
| 2031014 | Vlasi-2 | D12 | T:C | 1.64955 | 1.81767 |
| 2031014 | Vlasi-2 | E12 | T:T | 0.43075 | 3.55933 |
| 2031014 | Vlasi-2 | F12 | T:T | 0.44161 | 3.18508 |
| 2031014 | Vlasi-2 | G12 | T:T | 0.44049 | 3.48557 |
| 2031014 | Vlasi-2 | H12 | T:T | 0.43448 | 3.4513 |
| 2031014 | Vlasi-3 | A01 | T:C | 1.86388 | 1.90806 |
| 2031014 | Vlasi-3 | B01 | T:C | 1.72744 | 2.00674 |
| 2031014 | Vlasi-3 | C01 | T:C | 1.421 | 1.83313 |
| 2031014 | Vlasi-3 | D01 | T:T | 0.37903 | 3.45461 |
| 2031014 | Vlasi-3 | E01 | C:C | 3.63179 | 0.42132 |
| 2031014 | Vlasi-3 | F01 | T:T | 0.41692 | 3.58771 |
| 2031014 | Vlasi-3 | G01 | T:T | 0.41496 | 3.62939 |
| 2031014 | Vlasi-3 | H01 | T:T | 0.43362 | 3.73645 |
| 2031014 | Vlasi-3 | A02 | T:T | 0.44538 | 3.30586 |
| 2031014 | Vlasi-3 | B02 | T:C | 1.52019 | 1.93739 |
| 2031014 | Vlasi-3 | C02 | T:C | 1.47002 | 1.79916 |
| 2031014 | Vlasi-3 | D02 | T:T | 0.39739 | 3.25316 |
| 2031014 | Vlasi-3 | E02 | T:T | 0.38663 | 3.37397 |
| 2031014 | Vlasi-3 | F02 | T:C | 1.45689 | 1.68694 |
| 2031014 | Vlasi-3 | G02 | T:C | 1.58343 | 1.87709 |
| 2031014 | Vlasi-3 | H02 | T:T | 0.42116 | 3.24545 |
| 2031014 | Vlasi-3 | A03 | T:C | 1.71863 | 1.78365 |
| 2031014 | Vlasi-3 | B03 | T:T | 0.42075 | 3.58658 |
| 2031014 | Vlasi-3 | C03 | T:C | 1.68078 | 1.65081 |
| 2031014 | Vlasi-3 | D03 | T:T | 0.39788 | 3.25565 |
| 2031014 | Vlasi-3 | E03 | T:C | 1.40726 | 1.79923 |
| 2031014 | Vlasi-3 | F03 | ? | 0.82579 | 1.77348 |
| 2031014 | Vlasi-3 | G03 | ? | 0.98665 | 0.52255 |
| 2031014 | Vlasi-3 | H03 | T:C | 1.60467 | 1.8811 |
| 2031014 | Vlasi-3 | A04 | T:T | 0.422 | 3.42237 |
| 2031014 | Vlasi-3 | B04 | T:C | 1.38935 | 1.88069 |
| 2031014 | Vlasi-3 | C04 | T:T | 0.41527 | 3.30956 |
| 2031014 | Vlasi-3 | D04 | T:C | 1.45476 | 1.93459 |
| 2031014 | Vlasi-3 | E04 | T:C | 1.49907 | 1.95421 |
| 2031014 | Vlasi-3 | F04 | T:T | 0.41377 | 3.54647 |
| 2031014 | Vlasi-3 | G04 | ? | 1.80657 | 0.47312 |
| 2031014 | Vlasi-3 | H04 | T:T | 0.42396 | 3.38148 |
| 2031014 | Vlasi-3 | A05 | T:T | 0.42502 | 3.4099 |
| 2031014 | Vlasi-3 | B05 | T:C | 1.39811 | 1.77466 |
| 2031014 | Vlasi-3 | C05 | T:C | 1.52068 | 1.81657 |
| 2031014 | Vlasi-3 | D05 | T:C | 1.29744 | 1.95264 |
| 2031014 | Vlasi-3 | E05 | C:C | 3.50941 | 0.43559 |
| 2031014 | Vlasi-3 | F05 | T:C | 1.49586 | 1.75993 |
| 2031014 | Vlasi-3 | G05 | T:T | 0.4202 | 3.49397 |
| 2031014 | Vlasi-3 | H05 | T:T | 0.40136 | 3.44945 |
| 2031014 | Vlasi-3 | A06 | T:T | 0.40001 | 3.27788 |
| 2031014 | Vlasi-3 | B06 | T:T | 0.42907 | 3.44555 |
| 2031014 | Vlasi-3 | C06 | T:C | 1.61958 | 1.7369 |

| | | | | | |
|---|---|---|---|---|---|
| 2031014 | Vlasi-3 | D06 | T:T | 0.42108 | 3.50986 |
| 2031014 | Vlasi-3 | E06 | T:C | 1.59197 | 1.99852 |
| 2031014 | Vlasi-3 | F06 | T:T | 0.41149 | 3.41559 |
| 2031014 | Vlasi-3 | G06 | T:C | 1.51881 | 1.71594 |
| 2031014 | Vlasi-3 | H06 | T:T | 0.42372 | 3.55997 |
| 2031014 | Vlasi-3 | A07 | T:T | 0.41757 | 3.35601 |
| 2031014 | Vlasi-3 | B07 | T:C | 1.50866 | 1.76878 |
| 2031014 | Vlasi-3 | C07 | T:T | 0.43219 | 3.44344 |
| 2031014 | Vlasi-3 | D07 | T:T | 0.38898 | 3.42704 |
| 2031014 | Vlasi-3 | E07 | T:C | 1.51131 | 1.94137 |
| 2031014 | Vlasi-3 | F07 | T:C | 1.53652 | 1.67206 |
| 2031014 | Vlasi-3 | G07 | T:T | 0.43241 | 3.3782 |
| 2031014 | Vlasi-3 | H07 | T:C | 1.71599 | 1.92118 |
| 2031014 | Vlasi-3 | A08 | T:C | 1.62137 | 1.80679 |
| 2031014 | Vlasi-3 | B08 | T:C | 1.30265 | 1.71252 |
| 2031014 | Vlasi-3 | C08 | T:T | 0.40049 | 3.37814 |
| 2031014 | Vlasi-3 | D08 | T:C | 1.60159 | 1.92184 |
| 2031014 | Vlasi-3 | E08 | T:C | 1.52875 | 1.81833 |
| 2031014 | Vlasi-3 | F08 | T:C | 1.63905 | 1.74676 |
| 2031014 | Vlasi-3 | G08 | T:T | 0.42576 | 3.537 |
| 2031014 | Vlasi-3 | H08 | T:C | 1.5255 | 1.72692 |
| 2031014 | Vlasi-3 | A09 | T:T | 0.43641 | 3.52927 |
| 2031014 | Vlasi-3 | B09 | T:T | 0.4035 | 3.42177 |
| 2031014 | Vlasi-3 | C09 | T:T | 0.40714 | 3.41157 |
| 2031014 | Vlasi-3 | D09 | T:T | 0.38136 | 3.35423 |
| 2031014 | Vlasi-3 | E09 | T:T | 0.39567 | 3.30266 |
| 2031014 | Vlasi-3 | F09 | T:C | 1.63935 | 1.62402 |
| 2031014 | Vlasi-3 | G09 | T:C | 1.47091 | 1.78309 |
| 2031014 | Vlasi-3 | H09 | T:C | 1.70903 | 1.84039 |
| 2031014 | Vlasi-3 | A10 | T:C | 1.30061 | 1.67039 |
| 2031014 | Vlasi-3 | B10 | T:C | 1.65654 | 1.48763 |
| 2031014 | Vlasi-3 | C10 | T:C | 1.59099 | 1.81963 |
| 2031014 | Vlasi-3 | D10 | C:C | 3.27554 | 0.46002 |
| 2031014 | Vlasi-3 | E10 | T:T | 0.44113 | 3.48554 |
| 2031014 | Vlasi-3 | F10 | T:T | 0.42135 | 3.4383 |
| 2031014 | Vlasi-3 | G10 | T:T | 0.42021 | 3.41829 |
| 2031014 | Vlasi-3 | H10 | T:T | 0.43909 | 3.56592 |
| 2031014 | Vlasi-3 | A11 | T:C | 1.646 | 2.03926 |
| 2031014 | Vlasi-3 | B11 | T:T | 0.41051 | 3.30844 |
| 2031014 | Vlasi-3 | C11 | T:T | 0.41412 | 3.40255 |
| 2031014 | Vlasi-3 | D11 | T:C | 1.44826 | 1.8288 |
| 2031014 | Vlasi-3 | E11 | T:C | 1.54429 | 1.71088 |
| 2031014 | Vlasi-3 | F11 | T:C | 1.5386 | 1.68441 |
| 2031014 | Vlasi-3 | G11 | T:C | 1.46332 | 1.88046 |
| 2031014 | Vlasi-3 | H11 | C:C | 3.50181 | 0.47604 |
| 2031014 | Vlasi-3 | A12 | T:C | 1.54238 | 2.11233 |
| 2031014 | Vlasi-3 | B12 | T:C | 1.66322 | 1.94119 |
| 2031014 | Vlasi-3 | C12 | T:T | 0.3859 | 3.36756 |
| 2031014 | Vlasi-3 | D12 | T:C | 1.58277 | 1.86529 |
| 2031014 | Vlasi-3 | E12 | T:C | 1.61316 | 1.82638 |

| | | | | | |
|---|---|---|---|---|---|
| 2031014 | Vlasi-3 | F12 | T:T | 0.41008 | 3.3594 |
| 2031014 | Vlasi-3 | G12 | T:C | 1.60353 | 1.80251 |
| 2031014 | Vlasi-3 | H12 | T:C | 1.72327 | 2.10667 |
| 2031014 | Vlasi-4 | A01 | ? | 0.59244 | 0.43242 |
| 2031014 | Vlasi-4 | B01 | T:C | 1.81082 | 1.85666 |
| 2031014 | Vlasi-4 | C01 | T:C | 1.60469 | 1.76907 |
| 2031014 | Vlasi-4 | D01 | T:T | 0.4026 | 3.33631 |
| 2031014 | Vlasi-4 | E01 | T:C | 1.37851 | 1.64679 |
| 2031014 | Vlasi-4 | F01 | T:C | 1.59815 | 1.81394 |
| 2031014 | Vlasi-4 | G01 | T:T | 0.41608 | 3.46374 |
| 2031014 | Vlasi-4 | H01 | T:T | 0.44324 | 3.47839 |
| 2031014 | Vlasi-4 | A02 | T:C | 1.72823 | 1.96619 |
| 2031014 | Vlasi-4 | B02 | T:T | 0.45759 | 3.30882 |
| 2031014 | Vlasi-4 | C02 | ? | 0.93389 | 0.57354 |
| 2031014 | Vlasi-4 | D02 | T:C | 1.71085 | 1.92318 |
| 2031014 | Vlasi-4 | E02 | C:C | 3.3444 | 0.42959 |
| 2031014 | Vlasi-4 | F02 | T:T | 0.43602 | 3.60086 |
| 2031014 | Vlasi-4 | G02 | T:C | 1.62906 | 1.65665 |
| 2031014 | Vlasi-4 | H02 | T:C | 1.76182 | 1.87929 |
| 2031014 | Vlasi-4 | A03 | T:C | 1.78925 | 1.86259 |
| 2031014 | Vlasi-4 | B03 | T:C | 1.67344 | 1.86361 |
| 2031014 | Vlasi-4 | C03 | T:T | 0.39155 | 3.36427 |
| 2031014 | Vlasi-4 | D03 | T:T | 0.41461 | 3.42903 |
| 2031014 | Vlasi-4 | E03 | ? | 0.66832 | 0.38391 |
| 2031014 | Vlasi-4 | F03 | T:C | 1.72949 | 1.67856 |
| 2031014 | Vlasi-4 | G03 | T:T | 0.42395 | 3.44809 |
| 2031014 | Vlasi-4 | H03 | T:T | 0.4225 | 3.52154 |
| 2031014 | Vlasi-4 | A04 | C:C | 3.23289 | 0.41501 |
| 2031014 | Vlasi-4 | B04 | T:C | 1.39584 | 1.58326 |
| 2031014 | Vlasi-4 | C04 | C:C | 3.19853 | 0.4525 |
| 2031014 | Vlasi-4 | D04 | T:C | 1.3982 | 1.73864 |
| 2031014 | Vlasi-4 | E04 | T:T | 0.39908 | 3.11147 |
| 2031014 | Vlasi-4 | F04 | T:C | 1.52562 | 1.81713 |
| 2031014 | Vlasi-4 | G04 | T:T | 0.42569 | 3.37377 |
| 2031014 | Vlasi-4 | H04 | T:T | 0.43204 | 3.50517 |
| 2031014 | Vlasi-4 | A05 | T:T | 0.39254 | 3.26414 |
| 2031014 | Vlasi-4 | B05 | T:T | 0.38755 | 3.29714 |
| 2031014 | Vlasi-4 | C05 | T:C | 1.544 | 1.67999 |
| 2031014 | Vlasi-4 | D05 | T:C | 1.50539 | 1.74704 |
| 2031014 | Vlasi-4 | E05 | T:T | 0.394 | 3.38776 |
| 2031014 | Vlasi-4 | F05 | C:C | 3.24301 | 0.45105 |
| 2031014 | Vlasi-4 | G05 | T:T | 0.42842 | 3.55102 |
| 2031014 | Vlasi-4 | H05 | T:C | 1.69127 | 1.82607 |
| 2031014 | Vlasi-4 | A06 | T:T | 0.4229 | 3.15826 |
| 2031014 | Vlasi-4 | B06 | C:C | 3.20724 | 0.4282 |
| 2031014 | Vlasi-4 | C06 | C:C | 3.24592 | 0.47679 |
| 2031014 | Vlasi-4 | D06 | T:C | 1.41567 | 1.67025 |
| 2031014 | Vlasi-4 | E06 | T:C | 1.48768 | 1.80062 |
| 2031014 | Vlasi-4 | F06 | T:T | 0.45924 | 3.52228 |
| 2031014 | Vlasi-4 | G06 | T:T | 0.42878 | 3.3356 |

| | | | | | |
|---|---|---|---|---|---|
| 2031014 | Vlasi-4 | H06 | C:C | 3.39183 | 0.45992 |
| 2031014 | Vlasi-4 | A07 | ? | 0.82397 | 0.76273 |
| 2031014 | Vlasi-4 | B07 | ? | 0.52381 | 0.73658 |
| 2031014 | Vlasi-4 | C07 | ? | 0.56814 | 1.36048 |
| 2031014 | Vlasi-4 | D07 | ? | 0.57385 | 0.68429 |
| 2031014 | Vlasi-4 | E07 | ? | 0.59689 | 1.12091 |
| 2031014 | Vlasi-4 | F07 | C:C | 2.99639 | 0.64142 |
| 2031014 | Vlasi-4 | G07 | C:C | 3.31557 | 0.62949 |
| 2031014 | Vlasi-4 | H07 | C:C | 3.12435 | 0.72503 |
| 2031014 | Vlasi-4 | A08 | C:C | 3.12461 | 0.70601 |
| 2031014 | Vlasi-4 | B08 | C:C | 2.77615 | 0.70531 |
| 2031014 | Vlasi-4 | C08 | C:C | 3.09876 | 0.70992 |
| 2031014 | Vlasi-4 | D08 | C:C | 3.11599 | 0.66412 |
| 2031014 | Vlasi-4 | E08 | C:C | 3.10652 | 0.72604 |
| 2031014 | Vlasi-4 | F08 | C:C | 3.04294 | 0.7079 |
| 2031014 | Vlasi-4 | G08 | C:C | 3.08426 | 0.60313 |
| 2031014 | Vlasi-4 | H08 | C:C | 2.90978 | 0.79888 |
| 2031014 | Vlasi-4 | A09 | C:C | 2.96851 | 0.65137 |
| 2031014 | Vlasi-4 | B09 | C:C | 3.02932 | 0.68615 |
| 2031014 | Vlasi-4 | C09 | C:C | 2.95497 | 0.69436 |
| 2031014 | Vlasi-4 | D09 | C:C | 3.19057 | 0.72664 |
| 2031014 | Vlasi-4 | E09 | C:C | 2.9271 | 0.68811 |
| 2031014 | Vlasi-4 | F09 | C:C | 2.90071 | 0.62984 |
| 2031014 | Vlasi-4 | G09 | C:C | 2.7033 | 0.74332 |
| 2031014 | Vlasi-4 | H09 | C:C | 3.12153 | 0.78748 |
| 2031014 | Vlasi-4 | A10 | C:C | 2.85987 | 0.74072 |
| 2031014 | Vlasi-4 | B10 | C:C | 2.76873 | 0.83304 |
| 2031014 | Vlasi-4 | C10 | T:T | 0.46759 | 3.19134 |
| 2031014 | Vlasi-4 | D10 | T:T | 0.44087 | 3.11456 |
| 2031014 | Vlasi-4 | E10 | T:T | 0.64559 | 3.15671 |
| 2031014 | Vlasi-4 | F10 | T:T | 0.66796 | 2.98975 |
| 2031014 | Vlasi-4 | G10 | T:T | 0.64333 | 3.01715 |
| 2031014 | Vlasi-4 | H10 | T:T | 0.79213 | 2.94875 |
| 2031014 | Vlasi-4 | A11 | T:T | 0.78962 | 3.05458 |
| 2031014 | Vlasi-4 | B11 | T:T | 0.78073 | 2.99973 |
| 2031014 | Vlasi-4 | C11 | T:T | 0.74454 | 3.12317 |
| 2031014 | Vlasi-4 | D11 | T:T | 0.75901 | 2.94703 |
| 2031014 | Vlasi-4 | E11 | T:T | 0.77811 | 2.89822 |
| 2031014 | Vlasi-4 | F11 | T:T | 0.6674 | 2.69474 |
| 2031014 | Vlasi-4 | G11 | T:T | 0.72441 | 3.02157 |
| 2031014 | Vlasi-4 | H11 | T:T | 0.79155 | 3.01908 |
| 2031014 | Vlasi-4 | A12 | T:T | 0.89829 | 3.05367 |
| 2031014 | Vlasi-4 | B12 | T:T | 1.03525 | 2.80445 |
| 2031014 | Vlasi-4 | C12 | T:T | 1.02896 | 2.88355 |
| 2031014 | Vlasi-4 | D12 | T:T | 1.06558 | 2.97998 |
| 2031014 | Vlasi-4 | E12 | T:T | 0.859 | 3.03251 |
| 2031014 | Vlasi-4 | F12 | T:T | 0.85478 | 3.01606 |
| 2031014 | Vlasi-4 | G12 | T:T | 0.92486 | 3.01676 |
| 2031014 | Vlasi-4 | H12 | NTC | 0.60789 | 0.53859 |
| 2031014 | Vlasi-1 | A01 | ? | 0.59568 | 0.43231 |

| | | | | | |
|---|---|---|---|---|---|
| 2031014 | Vlasi-1 | B01 | A:A | 0.61051 | 3.47684 |
| 2031014 | Vlasi-1 | C01 | A:G | 2.3357 | 2.10405 |
| 2031014 | Vlasi-1 | D01 | A:G | 2.50129 | 1.88359 |
| 2031014 | Vlasi-1 | E01 | A:G | 2.63537 | 1.83981 |
| 2031014 | Vlasi-1 | F01 | A:A | 0.57845 | 3.71697 |
| 2031014 | Vlasi-1 | G01 | A:G | 2.69282 | 1.73664 |
| 2031014 | Vlasi-1 | H01 | A:G | 2.52738 | 2.12302 |
| 2031014 | Vlasi-1 | A02 | ? | 0.61962 | 0.4601 |
| 2031014 | Vlasi-1 | B02 | A:G | 2.40198 | 2.06794 |
| 2031014 | Vlasi-1 | C02 | A:G | 2.35777 | 2.10485 |
| 2031014 | Vlasi-1 | D02 | A:G | 2.5253 | 2.03154 |
| 2031014 | Vlasi-1 | E02 | A:A | 0.86808 | 3.10807 |
| 2031014 | Vlasi-1 | F02 | A:A | 0.46789 | 3.93775 |
| 2031014 | Vlasi-1 | G02 | A:A | 0.64677 | 3.46436 |
| 2031014 | Vlasi-1 | H02 | A:G | 2.30827 | 2.26626 |
| 2031014 | Vlasi-1 | A03 | ? | 1.99702 | 2.42466 |
| 2031014 | Vlasi-1 | B03 | A:G | 2.20459 | 2.22108 |
| 2031014 | Vlasi-1 | C03 | A:A | 0.68676 | 3.45737 |
| 2031014 | Vlasi-1 | D03 | A:G | 2.18463 | 2.29818 |
| 2031014 | Vlasi-1 | E03 | A:G | 2.39111 | 2.0211 |
| 2031014 | Vlasi-1 | F03 | A:A | 0.42121 | 3.42956 |
| 2031014 | Vlasi-1 | G03 | A:G | 2.49911 | 1.77924 |
| 2031014 | Vlasi-1 | H03 | A:A | 0.54525 | 3.60282 |
| 2031014 | Vlasi-1 | A04 | A:A | 0.54122 | 3.36737 |
| 2031014 | Vlasi-1 | B04 | A:G | 2.30579 | 2.33985 |
| 2031014 | Vlasi-1 | C04 | G:G | 3.80426 | 0.51645 |
| 2031014 | Vlasi-1 | D04 | A:A | 0.56662 | 3.35693 |
| 2031014 | Vlasi-1 | E04 | A:G | 2.19631 | 2.32193 |
| 2031014 | Vlasi-1 | F04 | A:A | 1.0452 | 2.97848 |
| 2031014 | Vlasi-1 | G04 | A:A | 0.50669 | 3.62474 |
| 2031014 | Vlasi-1 | H04 | G:G | 3.75162 | 0.50593 |
| 2031014 | Vlasi-1 | A05 | ? | 1.56689 | 2.55172 |
| 2031014 | Vlasi-1 | B05 | ? | 1.39988 | 2.78617 |
| 2031014 | Vlasi-1 | C05 | A:A | 0.52081 | 3.86332 |
| 2031014 | Vlasi-1 | D05 | ? | 0.81665 | 1.93199 |
| 2031014 | Vlasi-1 | E05 | ? | 0.75562 | 0.41506 |
| 2031014 | Vlasi-1 | F05 | ? | 1.02102 | 0.43986 |
| 2031014 | Vlasi-1 | G05 | ? | 0.97469 | 0.50365 |
| 2031014 | Vlasi-1 | H05 | A:A | 0.4534 | 3.7035 |
| 2031014 | Vlasi-1 | A06 | A:A | 0.75005 | 3.37753 |
| 2031014 | Vlasi-1 | B06 | A:G | 2.4624 | 1.91786 |
| 2031014 | Vlasi-1 | C06 | ? | 1.22994 | 2.79218 |
| 2031014 | Vlasi-1 | D06 | ? | 0.87768 | 0.8795 |
| 2031014 | Vlasi-1 | E06 | A:G | 2.20691 | 1.99484 |
| 2031014 | Vlasi-1 | F06 | ? | 0.80544 | 1.80684 |
| 2031014 | Vlasi-1 | G06 | A:G | 2.48381 | 1.87112 |
| 2031014 | Vlasi-1 | H06 | A:G | 2.42195 | 1.85948 |
| 2031014 | Vlasi-1 | A07 | A:A | 0.74169 | 3.26565 |
| 2031014 | Vlasi-1 | B07 | G:G | 3.5521 | 0.50295 |
| 2031014 | Vlasi-1 | C07 | ? | 0.95 | 0.45706 |

| | | | | | |
|---|---|---|---|---|---|
| 2031014 | Vlasi-1 | D07 | ? | 0.73611 | 2.56628 |
| 2031014 | Vlasi-1 | E07 | ? | 1.00769 | 0.44875 |
| 2031014 | Vlasi-1 | F07 | A:A | 0.52957 | 3.49047 |
| 2031014 | Vlasi-1 | G07 | G:G | 3.77615 | 0.51623 |
| 2031014 | Vlasi-1 | H07 | A:A | 0.51973 | 3.62216 |
| 2031014 | Vlasi-1 | A08 | G:G | 3.51654 | 0.53155 |
| 2031014 | Vlasi-1 | B08 | A:A | 0.53764 | 3.45337 |
| 2031014 | Vlasi-1 | C08 | A:G | 2.19337 | 1.9844 |
| 2031014 | Vlasi-1 | D08 | A:A | 0.45844 | 3.64697 |
| 2031014 | Vlasi-1 | E08 | A:A | 0.4873 | 3.72673 |
| 2031014 | Vlasi-1 | F08 | G:G | 3.55314 | 0.48378 |
| 2031014 | Vlasi-1 | G08 | G:G | 3.87607 | 0.54316 |
| 2031014 | Vlasi-1 | H08 | A:G | 2.60856 | 1.8665 |
| 2031014 | Vlasi-1 | A09 | A:A | 0.57271 | 3.2438 |
| 2031014 | Vlasi-1 | B09 | A:A | 0.60557 | 3.33243 |
| 2031014 | Vlasi-1 | C09 | A:G | 2.42151 | 1.88567 |
| 2031014 | Vlasi-1 | D09 | A:A | 0.502 | 3.81566 |
| 2031014 | Vlasi-1 | E09 | A:A | 0.47403 | 3.61102 |
| 2031014 | Vlasi-1 | F09 | A:G | 2.48259 | 1.88503 |
| 2031014 | Vlasi-1 | G09 | A:G | 2.64735 | 1.80063 |
| 2031014 | Vlasi-1 | H09 | A:G | 2.61808 | 1.8574 |
| 2031014 | Vlasi-1 | A10 | ? | 1.57946 | 2.18727 |
| 2031014 | Vlasi-1 | B10 | A:G | 2.16858 | 2.03841 |
| 2031014 | Vlasi-1 | C10 | A:A | 0.56066 | 3.46423 |
| 2031014 | Vlasi-1 | D10 | A:G | 2.4811 | 1.92985 |
| 2031014 | Vlasi-1 | E10 | A:A | 0.56877 | 3.50255 |
| 2031014 | Vlasi-1 | F10 | A:A | 0.52933 | 3.49447 |
| 2031014 | Vlasi-1 | G10 | G:G | 3.67915 | 0.52308 |
| 2031014 | Vlasi-1 | H10 | A:G | 2.44592 | 1.88073 |
| 2031014 | Vlasi-1 | A11 | A:A | 0.41176 | 3.48218 |
| 2031014 | Vlasi-1 | B11 | G:G | 3.55092 | 0.50376 |
| 2031014 | Vlasi-1 | C11 | A:G | 2.47027 | 1.88958 |
| 2031014 | Vlasi-1 | D11 | A:A | 0.68053 | 3.46035 |
| 2031014 | Vlasi-1 | E11 | A:G | 2.43168 | 1.94896 |
| 2031014 | Vlasi-1 | F11 | A:A | 0.51162 | 3.5613 |
| 2031014 | Vlasi-1 | G11 | A:A | 0.51969 | 3.36227 |
| 2031014 | Vlasi-1 | H11 | A:A | 0.49324 | 3.3947 |
| 2031014 | Vlasi-1 | A12 | A:A | 0.55729 | 3.50116 |
| 2031014 | Vlasi-1 | B12 | A:G | 2.47756 | 2.01703 |
| 2031014 | Vlasi-1 | C12 | G:G | 3.58679 | 0.53838 |
| 2031014 | Vlasi-1 | D12 | A:G | 2.44534 | 2.22805 |
| 2031014 | Vlasi-1 | E12 | G:G | 3.70066 | 0.50798 |
| 2031014 | Vlasi-1 | F12 | A:A | 0.57291 | 3.37646 |
| 2031014 | Vlasi-1 | G12 | A:G | 2.20307 | 2.23946 |
| 2031014 | Vlasi-1 | H12 | G:G | 3.9055 | 0.58113 |
| 2031014 | Vlasi-2 | A01 | A:A | 0.57149 | 3.38281 |
| 2031014 | Vlasi-2 | B01 | A:A | 0.52029 | 3.57296 |
| 2031014 | Vlasi-2 | C01 | A:A | 0.509 | 3.55108 |
| 2031014 | Vlasi-2 | D01 | ? | 0.75692 | 0.47053 |
| 2031014 | Vlasi-2 | E01 | A:A | 0.48659 | 3.81919 |

| | | | | | |
|---|---|---|---|---|---|
| 2031014 | Vlasi-2 | F01 | A:A | 0.49994 | 4.02174 |
| 2031014 | Vlasi-2 | G01 | A:G | 2.53881 | 1.84046 |
| 2031014 | Vlasi-2 | H01 | A:G | 2.35888 | 2.20876 |
| 2031014 | Vlasi-2 | A02 | A:G | 2.38301 | 2.03478 |
| 2031014 | Vlasi-2 | B02 | G:G | 3.75667 | 0.48317 |
| 2031014 | Vlasi-2 | C02 | G:G | 3.65565 | 0.55078 |
| 2031014 | Vlasi-2 | D02 | A:A | 0.54854 | 3.62615 |
| 2031014 | Vlasi-2 | E02 | A:G | 2.42968 | 2.0509 |
| 2031014 | Vlasi-2 | F02 | G:G | 3.60103 | 0.45603 |
| 2031014 | Vlasi-2 | G02 | A:A | 0.48297 | 3.66443 |
| 2031014 | Vlasi-2 | H02 | A:A | 0.4851 | 3.78776 |
| 2031014 | Vlasi-2 | A03 | A:A | 0.8478 | 3.15676 |
| 2031014 | Vlasi-2 | B03 | G:G | 3.59263 | 0.48556 |
| 2031014 | Vlasi-2 | C03 | A:G | 2.58772 | 2.10157 |
| 2031014 | Vlasi-2 | D03 | ? | 1.95247 | 2.31607 |
| 2031014 | Vlasi-2 | E03 | A:G | 2.56022 | 2.02856 |
| 2031014 | Vlasi-2 | F03 | A:A | 0.46467 | 3.68269 |
| 2031014 | Vlasi-2 | G03 | A:G | 2.29632 | 2.03469 |
| 2031014 | Vlasi-2 | H03 | A:G | 2.56147 | 1.8689 |
| 2031014 | Vlasi-2 | A04 | A:A | 0.79384 | 3.44274 |
| 2031014 | Vlasi-2 | B04 | A:A | 0.54989 | 3.47098 |
| 2031014 | Vlasi-2 | C04 | ? | 0.58105 | 0.41171 |
| 2031014 | Vlasi-2 | D04 | A:G | 2.41002 | 1.9028 |
| 2031014 | Vlasi-2 | E04 | ? | 1.06921 | 0.42765 |
| 2031014 | Vlasi-2 | F04 | ? | 0.80287 | 0.4827 |
| 2031014 | Vlasi-2 | G04 | ? | 0.98463 | 0.50828 |
| 2031014 | Vlasi-2 | H04 | A:G | 2.51155 | 1.99838 |
| 2031014 | Vlasi-2 | A05 | A:G | 2.27297 | 2.40078 |
| 2031014 | Vlasi-2 | B05 | A:A | 0.55914 | 3.4994 |
| 2031014 | Vlasi-2 | C05 | A:A | 0.89478 | 3.45637 |
| 2031014 | Vlasi-2 | D05 | ? | 0.85567 | 1.45175 |
| 2031014 | Vlasi-2 | E05 | A:A | 0.51212 | 3.56596 |
| 2031014 | Vlasi-2 | F05 | A:A | 0.46687 | 3.73614 |
| 2031014 | Vlasi-2 | G05 | ? | 1.27294 | 2.66518 |
| 2031014 | Vlasi-2 | H05 | A:G | 2.10019 | 2.19467 |
| 2031014 | Vlasi-2 | A06 | A:G | 2.30199 | 2.1202 |
| 2031014 | Vlasi-2 | B06 | A:G | 2.33481 | 1.99859 |
| 2031014 | Vlasi-2 | C06 | A:A | 0.53946 | 3.79207 |
| 2031014 | Vlasi-2 | D06 | A:G | 2.2716 | 2.30277 |
| 2031014 | Vlasi-2 | E06 | A:G | 2.57142 | 1.95772 |
| 2031014 | Vlasi-2 | F06 | A:G | 2.44933 | 2.10143 |
| 2031014 | Vlasi-2 | G06 | A:A | 0.53619 | 3.98383 |
| 2031014 | Vlasi-2 | H06 | A:A | 0.59535 | 3.58675 |
| 2031014 | Vlasi-2 | A07 | A:G | 2.36376 | 2.01152 |
| 2031014 | Vlasi-2 | B07 | ? | 0.67566 | 0.37723 |
| 2031014 | Vlasi-2 | C07 | G:G | 3.73907 | 0.51335 |
| 2031014 | Vlasi-2 | D07 | ? | 0.89521 | 0.51103 |
| 2031014 | Vlasi-2 | E07 | ? | 0.87538 | 0.46862 |
| 2031014 | Vlasi-2 | F07 | A:G | 2.6055 | 1.77943 |
| 2031014 | Vlasi-2 | G07 | A:G | 2.44025 | 1.91065 |

| | | | | | |
|---|---|---|---|---|---|
| 2031014 | Vlasi-2 | H07 | A:G | 2.45195 | 2.03225 |
| 2031014 | Vlasi-2 | A08 | A:A | 0.69859 | 3.44059 |
| 2031014 | Vlasi-2 | B08 | A:A | 0.44785 | 3.61705 |
| 2031014 | Vlasi-2 | C08 | A:A | 0.71841 | 2.979 |
| 2031014 | Vlasi-2 | D08 | A:G | 2.26675 | 2.06818 |
| 2031014 | Vlasi-2 | E08 | G:G | 3.85101 | 0.48601 |
| 2031014 | Vlasi-2 | F08 | A:G | 2.44736 | 1.87575 |
| 2031014 | Vlasi-2 | G08 | A:A | 0.57505 | 3.6097 |
| 2031014 | Vlasi-2 | H08 | A:G | 2.68651 | 1.86239 |
| 2031014 | Vlasi-2 | A09 | ? | 0.61043 | 0.42992 |
| 2031014 | Vlasi-2 | B09 | ? | 1.88023 | 2.25478 |
| 2031014 | Vlasi-2 | C09 | ? | 1.09294 | 0.50298 |
| 2031014 | Vlasi-2 | D09 | ? | 1.77073 | 2.5329 |
| 2031014 | Vlasi-2 | E09 | A:A | 0.57776 | 3.51206 |
| 2031014 | Vlasi-2 | F09 | ? | 0.55095 | 1.61535 |
| 2031014 | Vlasi-2 | G09 | A:A | 0.46615 | 3.72892 |
| 2031014 | Vlasi-2 | H09 | A:A | 0.50578 | 3.56037 |
| 2031014 | Vlasi-2 | A10 | G:G | 3.33324 | 0.66668 |
| 2031014 | Vlasi-2 | B10 | A:G | 2.63922 | 1.8967 |
| 2031014 | Vlasi-2 | C10 | G:G | 3.63366 | 0.48304 |
| 2031014 | Vlasi-2 | D10 | A:G | 2.30925 | 2.01433 |
| 2031014 | Vlasi-2 | E10 | A:G | 2.48329 | 1.94428 |
| 2031014 | Vlasi-2 | F10 | A:A | 1.09639 | 3.16821 |
| 2031014 | Vlasi-2 | G10 | G:G | 3.73453 | 0.55523 |
| 2031014 | Vlasi-2 | H10 | A:G | 2.25902 | 2.06348 |
| 2031014 | Vlasi-2 | A11 | A:A | 0.99242 | 3.2125 |
| 2031014 | Vlasi-2 | B11 | ? | 1.54224 | 2.86107 |
| 2031014 | Vlasi-2 | C11 | A:G | 2.1594 | 2.04162 |
| 2031014 | Vlasi-2 | D11 | A:A | 0.53823 | 3.47309 |
| 2031014 | Vlasi-2 | E11 | G:G | 3.67343 | 0.50979 |
| 2031014 | Vlasi-2 | F11 | A:A | 0.55147 | 3.67991 |
| 2031014 | Vlasi-2 | G11 | A:G | 2.46657 | 2.02611 |
| 2031014 | Vlasi-2 | H11 | A:A | 0.58133 | 3.47633 |
| 2031014 | Vlasi-2 | A12 | G:G | 3.59759 | 0.63127 |
| 2031014 | Vlasi-2 | B12 | G:G | 3.78656 | 0.59826 |
| 2031014 | Vlasi-2 | C12 | A:A | 0.69494 | 3.29779 |
| 2031014 | Vlasi-2 | D12 | A:G | 2.55389 | 2.17939 |
| 2031014 | Vlasi-2 | E12 | A:A | 0.62112 | 3.51373 |
| 2031014 | Vlasi-2 | F12 | A:A | 0.68507 | 3.57415 |
| 2031014 | Vlasi-2 | G12 | A:G | 2.38143 | 2.05182 |
| 2031014 | Vlasi-2 | H12 | A:A | 0.72952 | 3.69826 |
| 2031014 | Vlasi-3 | A01 | G:G | 3.80522 | 0.5344 |
| 2031014 | Vlasi-3 | B01 | G:G | 3.95232 | 0.51408 |
| 2031014 | Vlasi-3 | C01 | G:G | 3.78218 | 0.5087 |
| 2031014 | Vlasi-3 | D01 | A:G | 2.43884 | 1.9367 |
| 2031014 | Vlasi-3 | E01 | A:A | 0.56309 | 3.53668 |
| 2031014 | Vlasi-3 | F01 | A:A | 0.51998 | 3.58978 |
| 2031014 | Vlasi-3 | G01 | G:G | 3.81215 | 0.49221 |
| 2031014 | Vlasi-3 | H01 | A:G | 2.57044 | 1.94539 |
| 2031014 | Vlasi-3 | A02 | A:G | 2.58677 | 2.05597 |

| | | | | | |
|---|---|---|---|---|---|
| 2031014 | Vlasi-3 | B02 | A:A | 0.52094 | 3.53818 |
| 2031014 | Vlasi-3 | C02 | A:G | 2.63082 | 2.00125 |
| 2031014 | Vlasi-3 | D02 | A:G | 2.41172 | 1.94572 |
| 2031014 | Vlasi-3 | E02 | A:A | 0.52272 | 3.50391 |
| 2031014 | Vlasi-3 | F02 | A:A | 0.588 | 3.53278 |
| 2031014 | Vlasi-3 | G02 | A:A | 0.51878 | 3.41219 |
| 2031014 | Vlasi-3 | H02 | A:G | 2.66159 | 1.89954 |
| 2031014 | Vlasi-3 | A03 | A:G | 2.39947 | 2.05347 |
| 2031014 | Vlasi-3 | B03 | A:A | 0.49855 | 3.57959 |
| 2031014 | Vlasi-3 | C03 | A:G | 2.43728 | 1.97147 |
| 2031014 | Vlasi-3 | D03 | A:G | 2.55354 | 2.00002 |
| 2031014 | Vlasi-3 | E03 | A:G | 2.5211 | 1.79356 |
| 2031014 | Vlasi-3 | F03 | ? | 1.90982 | 2.18406 |
| 2031014 | Vlasi-3 | G03 | ? | 0.82545 | 0.53701 |
| 2031014 | Vlasi-3 | H03 | A:A | 0.63545 | 3.68962 |
| 2031014 | Vlasi-3 | A04 | A:G | 2.32184 | 2.09467 |
| 2031014 | Vlasi-3 | B04 | A:A | 0.48237 | 3.55332 |
| 2031014 | Vlasi-3 | C04 | A:A | 0.51948 | 3.51345 |
| 2031014 | Vlasi-3 | D04 | A:G | 2.30466 | 1.80892 |
| 2031014 | Vlasi-3 | E04 | A:G | 2.39653 | 1.74558 |
| 2031014 | Vlasi-3 | F04 | A:G | 2.16747 | 2.01411 |
| 2031014 | Vlasi-3 | G04 | ? | 1.50056 | 2.51529 |
| 2031014 | Vlasi-3 | H04 | A:A | 0.60388 | 3.60379 |
| 2031014 | Vlasi-3 | A05 | G:G | 3.48898 | 0.62149 |
| 2031014 | Vlasi-3 | B05 | A:G | 2.41815 | 1.90934 |
| 2031014 | Vlasi-3 | C05 | A:G | 2.55906 | 1.79643 |
| 2031014 | Vlasi-3 | D05 | A:A | 0.64081 | 3.17358 |
| 2031014 | Vlasi-3 | E05 | ? | 1.35754 | 2.6468 |
| 2031014 | Vlasi-3 | F05 | A:G | 2.62583 | 1.7331 |
| 2031014 | Vlasi-3 | G05 | A:A | 0.47223 | 3.36334 |
| 2031014 | Vlasi-3 | H05 | A:A | 0.51871 | 3.59788 |
| 2031014 | Vlasi-3 | A06 | A:A | 0.54971 | 3.39015 |
| 2031014 | Vlasi-3 | B06 | A:G | 2.21677 | 2.03491 |
| 2031014 | Vlasi-3 | C06 | A:A | 0.48167 | 3.55849 |
| 2031014 | Vlasi-3 | D06 | A:G | 2.42353 | 1.91858 |
| 2031014 | Vlasi-3 | E06 | A:A | 0.5099 | 3.68337 |
| 2031014 | Vlasi-3 | F06 | G:G | 3.91516 | 0.51811 |
| 2031014 | Vlasi-3 | G06 | A:G | 2.66198 | 1.70608 |
| 2031014 | Vlasi-3 | H06 | A:G | 2.29957 | 2.27267 |
| 2031014 | Vlasi-3 | A07 | A:G | 2.50946 | 2.06763 |
| 2031014 | Vlasi-3 | B07 | A:G | 2.19086 | 2.05755 |
| 2031014 | Vlasi-3 | C07 | A:A | 0.47124 | 3.72242 |
| 2031014 | Vlasi-3 | D07 | A:A | 0.43897 | 3.66596 |
| 2031014 | Vlasi-3 | E07 | A:A | 0.44158 | 3.54856 |
| 2031014 | Vlasi-3 | F07 | A:G | 2.23861 | 1.95783 |
| 2031014 | Vlasi-3 | G07 | A:A | 0.50427 | 3.60156 |
| 2031014 | Vlasi-3 | H07 | G:G | 3.78769 | 0.51688 |
| 2031014 | Vlasi-3 | A08 | A:G | 2.27062 | 2.06955 |
| 2031014 | Vlasi-3 | B08 | A:A | 0.4862 | 3.49527 |
| 2031014 | Vlasi-3 | C08 | A:A | 0.42383 | 3.57399 |

| | | | | | |
|---|---|---|---|---|---|
| 2031014 | Vlasi-3 | D08 | A:G | 2.14383 | 2.15124 |
| 2031014 | Vlasi-3 | E08 | A:G | 2.15322 | 2.02915 |
| 2031014 | Vlasi-3 | F08 | A:G | 2.60764 | 1.82562 |
| 2031014 | Vlasi-3 | G08 | A:A | 0.46706 | 3.6978 |
| 2031014 | Vlasi-3 | H08 | A:A | 0.5707 | 3.64296 |
| 2031014 | Vlasi-3 | A09 | A:G | 2.16464 | 2.14261 |
| 2031014 | Vlasi-3 | B09 | A:A | 0.48028 | 3.54957 |
| 2031014 | Vlasi-3 | C09 | A:G | 2.39191 | 1.941 |
| 2031014 | Vlasi-3 | D09 | A:A | 0.51586 | 3.69754 |
| 2031014 | Vlasi-3 | E09 | A:A | 0.51599 | 3.23895 |
| 2031014 | Vlasi-3 | F09 | A:G | 2.48687 | 1.91487 |
| 2031014 | Vlasi-3 | G09 | G:G | 3.72718 | 0.49734 |
| 2031014 | Vlasi-3 | H09 | A:A | 0.58051 | 3.57162 |
| 2031014 | Vlasi-3 | A10 | ? | 0.59989 | 0.44564 |
| 2031014 | Vlasi-3 | B10 | A:G | 2.38632 | 2.02483 |
| 2031014 | Vlasi-3 | C10 | A:G | 2.43841 | 1.9596 |
| 2031014 | Vlasi-3 | D10 | A:A | 0.53061 | 3.81559 |
| 2031014 | Vlasi-3 | E10 | A:A | 0.53984 | 3.43234 |
| 2031014 | Vlasi-3 | F10 | A:A | 0.48507 | 3.73811 |
| 2031014 | Vlasi-3 | G10 | A:G | 2.52053 | 1.89353 |
| 2031014 | Vlasi-3 | H10 | A:A | 0.54936 | 3.71842 |
| 2031014 | Vlasi-3 | A11 | A:A | 0.59676 | 3.51562 |
| 2031014 | Vlasi-3 | B11 | A:G | 2.51531 | 1.90943 |
| 2031014 | Vlasi-3 | C11 | A:A | 0.52834 | 3.6298 |
| 2031014 | Vlasi-3 | D11 | G:G | 3.71966 | 0.51696 |
| 2031014 | Vlasi-3 | E11 | A:G | 2.74408 | 1.94401 |
| 2031014 | Vlasi-3 | F11 | A:G | 2.51428 | 2.08218 |
| 2031014 | Vlasi-3 | G11 | A:G | 2.53612 | 1.90819 |
| 2031014 | Vlasi-3 | H11 | A:A | 0.54332 | 3.60205 |
| 2031014 | Vlasi-3 | A12 | A:A | 0.63186 | 3.50006 |
| 2031014 | Vlasi-3 | B12 | G:G | 3.77711 | 0.57355 |
| 2031014 | Vlasi-3 | C12 | A:A | 0.60416 | 3.43295 |
| 2031014 | Vlasi-3 | D12 | G:G | 3.68591 | 0.57548 |
| 2031014 | Vlasi-3 | E12 | A:G | 2.51592 | 1.87651 |
| 2031014 | Vlasi-3 | F12 | A:G | 2.27373 | 2.14767 |
| 2031014 | Vlasi-3 | G12 | A:G | 2.48026 | 1.93592 |
| 2031014 | Vlasi-3 | H12 | A:A | 1.07277 | 3.45475 |
| 2031014 | Vlasi-4 | A01 | A:A | 0.46533 | 3.65669 |
| 2031014 | Vlasi-4 | B01 | ? | 1.88275 | 2.44451 |
| 2031014 | Vlasi-4 | C01 | A:G | 2.1259 | 2.14134 |
| 2031014 | Vlasi-4 | D01 | A:G | 1.9916 | 2.08242 |
| 2031014 | Vlasi-4 | E01 | A:A | 0.47125 | 3.48266 |
| 2031014 | Vlasi-4 | F01 | G:G | 3.81094 | 0.54191 |
| 2031014 | Vlasi-4 | G01 | ? | 1.68518 | 2.67488 |
| 2031014 | Vlasi-4 | H01 | A:A | 0.68364 | 3.44137 |
| 2031014 | Vlasi-4 | A02 | A:A | 0.64461 | 3.4322 |
| 2031014 | Vlasi-4 | B02 | A:G | 2.67185 | 2.03195 |
| 2031014 | Vlasi-4 | C02 | ? | 0.54754 | 2.86394 |
| 2031014 | Vlasi-4 | D02 | A:G | 2.2885 | 2.13879 |
| 2031014 | Vlasi-4 | E02 | A:G | 2.51642 | 1.92422 |

| | | | | | |
|---|---|---|---|---|---|
| 2031014 | Vlasi-4 | F02 | A:A | 0.4644 | 3.64318 |
| 2031014 | Vlasi-4 | G02 | A:A | 0.45799 | 3.66762 |
| 2031014 | Vlasi-4 | H02 | A:G | 2.3394 | 2.26241 |
| 2031014 | Vlasi-4 | A03 | A:G | 2.30866 | 2.16385 |
| 2031014 | Vlasi-4 | B03 | A:G | 2.40168 | 2.03012 |
| 2031014 | Vlasi-4 | C03 | ? | 1.64882 | 2.32656 |
| 2031014 | Vlasi-4 | D03 | A:A | 0.45029 | 3.64219 |
| 2031014 | Vlasi-4 | E03 | G:G | 3.65603 | 0.49841 |
| 2031014 | Vlasi-4 | F03 | A:A | 0.45919 | 3.54626 |
| 2031014 | Vlasi-4 | G03 | A:G | 2.78925 | 1.75207 |
| 2031014 | Vlasi-4 | H03 | G:G | 3.76731 | 0.52005 |
| 2031014 | Vlasi-4 | A04 | A:G | 2.24532 | 2.16622 |
| 2031014 | Vlasi-4 | B04 | A:A | 0.52494 | 3.544 |
| 2031014 | Vlasi-4 | C04 | A:A | 0.69098 | 3.59237 |
| 2031014 | Vlasi-4 | D04 | A:A | 0.42607 | 3.66486 |
| 2031014 | Vlasi-4 | E04 | G:G | 3.72833 | 0.53914 |
| 2031014 | Vlasi-4 | F04 | A:G | 2.51467 | 2.11561 |
| 2031014 | Vlasi-4 | G04 | G:G | 3.71651 | 0.53612 |
| 2031014 | Vlasi-4 | H04 | G:G | 3.63164 | 0.55638 |
| 2031014 | Vlasi-4 | A05 | A:A | 0.53562 | 3.47439 |
| 2031014 | Vlasi-4 | B05 | A:A | 0.5371 | 3.4181 |
| 2031014 | Vlasi-4 | C05 | A:A | 0.51021 | 3.42651 |
| 2031014 | Vlasi-4 | D05 | A:A | 0.46229 | 3.57587 |
| 2031014 | Vlasi-4 | E05 | A:G | 2.45564 | 1.8841 |
| 2031014 | Vlasi-4 | F05 | G:G | 3.69737 | 0.47884 |
| 2031014 | Vlasi-4 | G05 | A:A | 0.44692 | 3.55007 |
| 2031014 | Vlasi-4 | H05 | A:G | 2.59077 | 1.98697 |
| 2031014 | Vlasi-4 | A06 | ? | 1.80831 | 2.11692 |
| 2031014 | Vlasi-4 | B06 | A:G | 2.18048 | 2.11073 |
| 2031014 | Vlasi-4 | C06 | A:G | 2.54377 | 1.91668 |
| 2031014 | Vlasi-4 | D06 | ? | 1.81007 | 2.1379 |
| 2031014 | Vlasi-4 | E06 | A:A | 0.74317 | 3.33192 |
| 2031014 | Vlasi-4 | F06 | G:G | 3.71636 | 0.55407 |
| 2031014 | Vlasi-4 | G06 | G:G | 3.53476 | 0.48794 |
| 2031014 | Vlasi-4 | H06 | A:G | 2.43539 | 2.09622 |
| 2031014 | Vlasi-4 | A07 | G:G | 3.27354 | 0.56853 |
| 2031014 | Vlasi-4 | B07 | G:G | 3.34206 | 0.61013 |
| 2031014 | Vlasi-4 | C07 | G:G | 3.46674 | 0.51088 |
| 2031014 | Vlasi-4 | D07 | G:G | 3.53003 | 0.58013 |
| 2031014 | Vlasi-4 | E07 | G:G | 3.69182 | 0.60004 |
| 2031014 | Vlasi-4 | F07 | G:G | 3.6481 | 0.54475 |
| 2031014 | Vlasi-4 | G07 | G:G | 3.70023 | 0.59611 |
| 2031014 | Vlasi-4 | H07 | G:G | 3.87873 | 0.66395 |
| 2031014 | Vlasi-4 | A08 | G:G | 3.59026 | 0.51554 |
| 2031014 | Vlasi-4 | B08 | G:G | 3.42799 | 0.85425 |
| 2031014 | Vlasi-4 | C08 | G:G | 3.45425 | 0.62179 |
| 2031014 | Vlasi-4 | D08 | G:G | 3.6493 | 0.56595 |
| 2031014 | Vlasi-4 | E08 | G:G | 3.72353 | 0.56446 |
| 2031014 | Vlasi-4 | F08 | G:G | 3.78397 | 0.59826 |
| 2031014 | Vlasi-4 | G08 | G:G | 3.72583 | 0.59859 |

| | | | | | |
|---|---|---|---|---|---|
| 2031014 | Vlasi-4 | H08 | G:G | 3.75996 | 0.62893 |
| 2031014 | Vlasi-4 | A09 | G:G | 3.62579 | 0.59394 |
| 2031014 | Vlasi-4 | B09 | G:G | 3.50634 | 0.54894 |
| 2031014 | Vlasi-4 | C09 | G:G | 3.75709 | 0.70888 |
| 2031014 | Vlasi-4 | D09 | G:G | 3.64385 | 0.58428 |
| 2031014 | Vlasi-4 | E09 | G:G | 3.78521 | 0.57545 |
| 2031014 | Vlasi-4 | F09 | G:G | 3.63803 | 0.52415 |
| 2031014 | Vlasi-4 | G09 | G:G | 3.74639 | 0.55325 |
| 2031014 | Vlasi-4 | H09 | G:G | 3.74107 | 0.57736 |
| 2031014 | Vlasi-4 | A10 | G:G | 3.53965 | 0.56282 |
| 2031014 | Vlasi-4 | B10 | G:G | 3.51366 | 0.58415 |
| 2031014 | Vlasi-4 | C10 | ? | 1.57632 | 2.69874 |
| 2031014 | Vlasi-4 | D10 | ? | 1.73382 | 2.62845 |
| 2031014 | Vlasi-4 | E10 | ? | 2.22133 | 2.51742 |
| 2031014 | Vlasi-4 | F10 | ? | 1.89717 | 2.69914 |
| 2031014 | Vlasi-4 | G10 | ? | 2.05329 | 2.54711 |
| 2031014 | Vlasi-4 | H10 | ? | 2.09112 | 2.70071 |
| 2031014 | Vlasi-4 | A11 | ? | 1.85916 | 2.67458 |
| 2031014 | Vlasi-4 | B11 | A:A | 1.18211 | 3.05702 |
| 2031014 | Vlasi-4 | C11 | ? | 2.16771 | 2.56931 |
| 2031014 | Vlasi-4 | D11 | A:A | 1.04598 | 3.20577 |
| 2031014 | Vlasi-4 | E11 | ? | 1.92047 | 2.52664 |
| 2031014 | Vlasi-4 | F11 | ? | 1.64622 | 2.77057 |
| 2031014 | Vlasi-4 | G11 | A:A | 1.13971 | 3.08199 |
| 2031014 | Vlasi-4 | H11 | A:A | 1.11206 | 3.20472 |
| 2031014 | Vlasi-4 | A12 | ? | 1.75717 | 2.83583 |
| 2031014 | Vlasi-4 | B12 | ? | 2.03267 | 2.45977 |
| 2031014 | Vlasi-4 | C12 | ? | 2.11188 | 2.58549 |
| 2031014 | Vlasi-4 | D12 | ? | 2.13971 | 2.51862 |
| 2031014 | Vlasi-4 | E12 | ? | 1.8976 | 2.72686 |
| 2031014 | Vlasi-4 | F12 | ? | 2.02659 | 2.5371 |
| 2031014 | Vlasi-4 | G12 | ? | 1.37207 | 3.11638 |
| 2031014 | Vlasi-4 | H12 | NTC | 3.86836 | 0.58675 |
| 2031014 | Vlasi-1 | A01 | T:T | 0.65459 | 3.55991 |
| 2031014 | Vlasi-1 | B01 | T:A | 2.3649 | 2.57413 |
| 2031014 | Vlasi-1 | C01 | T:A | 2.29049 | 2.61571 |
| 2031014 | Vlasi-1 | D01 | T:A | 2.21552 | 2.59223 |
| 2031014 | Vlasi-1 | E01 | T:T | 0.57942 | 3.7596 |
| 2031014 | Vlasi-1 | F01 | T:A | 2.3212 | 2.68175 |
| 2031014 | Vlasi-1 | G01 | T:A | 2.36558 | 2.61223 |
| 2031014 | Vlasi-1 | H01 | T:T | 0.52078 | 3.72891 |
| 2031014 | Vlasi-1 | A02 | T:T | 0.60642 | 3.42267 |
| 2031014 | Vlasi-1 | B02 | T:A | 2.25441 | 2.58183 |
| 2031014 | Vlasi-1 | C02 | T:T | 0.52169 | 3.72847 |
| 2031014 | Vlasi-1 | D02 | T:T | 0.5486 | 3.96345 |
| 2031014 | Vlasi-1 | E02 | T:A | 2.10152 | 2.47625 |
| 2031014 | Vlasi-1 | F02 | T:A | 2.27356 | 2.66836 |
| 2031014 | Vlasi-1 | G02 | T:T | 0.47159 | 3.57235 |
| 2031014 | Vlasi-1 | H02 | T:A | 2.24071 | 2.58107 |
| 2031014 | Vlasi-1 | A03 | A:A | 3.65806 | 0.71246 |

| | | | | | |
|---|---|---|---|---|---|
| 2031014 | Vlasi-1 | B03 | A:A | 3.89426 | 0.58316 |
| 2031014 | Vlasi-1 | C03 | T:T | 0.53465 | 3.71095 |
| 2031014 | Vlasi-1 | D03 | T:A | 1.5464 | 2.32214 |
| 2031014 | Vlasi-1 | E03 | T:A | 2.19202 | 2.55051 |
| 2031014 | Vlasi-1 | F03 | T:T | 0.46045 | 3.56749 |
| 2031014 | Vlasi-1 | G03 | T:A | 2.0581 | 2.49316 |
| 2031014 | Vlasi-1 | H03 | T:A | 2.15358 | 2.42717 |
| 2031014 | Vlasi-1 | A04 | T:A | 2.35662 | 2.52631 |
| 2031014 | Vlasi-1 | B04 | T:T | 0.53826 | 3.51325 |
| 2031014 | Vlasi-1 | C04 | T:T | 0.5806 | 3.90201 |
| 2031014 | Vlasi-1 | D04 | T:A | 1.74272 | 2.41267 |
| 2031014 | Vlasi-1 | E04 | T:A | 2.26343 | 2.59292 |
| 2031014 | Vlasi-1 | F04 | T:A | 2.20783 | 2.44388 |
| 2031014 | Vlasi-1 | G04 | T:A | 2.23494 | 2.52855 |
| 2031014 | Vlasi-1 | H04 | T:T | 0.47596 | 3.68322 |
| 2031014 | Vlasi-1 | A05 | T:T | 0.56128 | 3.52114 |
| 2031014 | Vlasi-1 | B05 | T:A | 1.79257 | 2.57019 |
| 2031014 | Vlasi-1 | C05 | T:A | 1.90053 | 2.54235 |
| 2031014 | Vlasi-1 | D05 | T:A | 1.84379 | 2.47562 |
| 2031014 | Vlasi-1 | E05 | T:T | 0.61616 | 3.73538 |
| 2031014 | Vlasi-1 | F05 | ? | 1.05009 | 0.48965 |
| 2031014 | Vlasi-1 | G05 | T:A | 1.72407 | 2.56715 |
| 2031014 | Vlasi-1 | H05 | T:T | 0.49081 | 3.60355 |
| 2031014 | Vlasi-1 | A06 | A:A | 3.69478 | 0.64337 |
| 2031014 | Vlasi-1 | B06 | T:A | 1.9548 | 2.5536 |
| 2031014 | Vlasi-1 | C06 | T:A | 2.0999 | 2.62692 |
| 2031014 | Vlasi-1 | D06 | T:T | 0.63766 | 3.66743 |
| 2031014 | Vlasi-1 | E06 | T:T | 0.59018 | 3.7202 |
| 2031014 | Vlasi-1 | F06 | T:A | 1.99403 | 2.72558 |
| 2031014 | Vlasi-1 | G06 | A:A | 4.03488 | 0.56879 |
| 2031014 | Vlasi-1 | H06 | T:T | 0.4814 | 3.76643 |
| 2031014 | Vlasi-1 | A07 | A:A | 3.6936 | 0.666 |
| 2031014 | Vlasi-1 | B07 | T:T | 0.55655 | 3.73882 |
| 2031014 | Vlasi-1 | C07 | T:A | 2.14952 | 2.62579 |
| 2031014 | Vlasi-1 | D07 | T:T | 0.56739 | 3.91869 |
| 2031014 | Vlasi-1 | E07 | T:T | 0.74697 | 3.38056 |
| 2031014 | Vlasi-1 | F07 | T:T | 0.48422 | 3.75275 |
| 2031014 | Vlasi-1 | G07 | T:A | 2.08339 | 2.53961 |
| 2031014 | Vlasi-1 | H07 | T:T | 0.50601 | 3.6746 |
| 2031014 | Vlasi-1 | A08 | T:A | 2.19931 | 2.56243 |
| 2031014 | Vlasi-1 | B08 | T:T | 0.48081 | 3.54666 |
| 2031014 | Vlasi-1 | C08 | T:T | 0.50499 | 3.70154 |
| 2031014 | Vlasi-1 | D08 | T:A | 2.12885 | 2.76453 |
| 2031014 | Vlasi-1 | E08 | T:A | 2.03681 | 2.55071 |
| 2031014 | Vlasi-1 | F08 | T:A | 2.05661 | 2.69323 |
| 2031014 | Vlasi-1 | G08 | T:T | 0.50318 | 3.81367 |
| 2031014 | Vlasi-1 | H08 | T:A | 2.22463 | 2.56482 |
| 2031014 | Vlasi-1 | A09 | T:A | 2.06773 | 2.48245 |
| 2031014 | Vlasi-1 | B09 | T:T | 0.50294 | 3.57483 |
| 2031014 | Vlasi-1 | C09 | T:A | 2.24546 | 2.70395 |

| | | | | | |
|---|---|---|---|---|---|
| 2031014 | Vlasi-1 | D09 | T:T | 0.55051 | 3.84211 |
| 2031014 | Vlasi-1 | E09 | T:A | 2.17828 | 2.58478 |
| 2031014 | Vlasi-1 | F09 | T:A | 2.18141 | 2.69249 |
| 2031014 | Vlasi-1 | G09 | A:A | 3.61054 | 0.81743 |
| 2031014 | Vlasi-1 | H09 | T:T | 0.49757 | 3.65055 |
| 2031014 | Vlasi-1 | A10 | T:A | 2.05124 | 2.53561 |
| 2031014 | Vlasi-1 | B10 | T:A | 2.17469 | 2.44511 |
| 2031014 | Vlasi-1 | C10 | T:A | 2.17883 | 2.65171 |
| 2031014 | Vlasi-1 | D10 | A:A | 3.49242 | 0.6745 |
| 2031014 | Vlasi-1 | E10 | A:A | 3.75229 | 0.60105 |
| 2031014 | Vlasi-1 | F10 | A:A | 3.79305 | 0.55623 |
| 2031014 | Vlasi-1 | G10 | T:A | 2.02326 | 2.54024 |
| 2031014 | Vlasi-1 | H10 | T:T | 0.49202 | 3.72473 |
| 2031014 | Vlasi-1 | A11 | T:T | 0.55383 | 3.68165 |
| 2031014 | Vlasi-1 | B11 | T:A | 2.16542 | 2.53222 |
| 2031014 | Vlasi-1 | C11 | T:T | 0.49247 | 3.71816 |
| 2031014 | Vlasi-1 | D11 | T:A | 2.17612 | 2.4866 |
| 2031014 | Vlasi-1 | E11 | T:T | 0.48301 | 3.71324 |
| 2031014 | Vlasi-1 | F11 | T:A | 2.07663 | 2.52134 |
| 2031014 | Vlasi-1 | G11 | T:T | 0.45821 | 3.4776 |
| 2031014 | Vlasi-1 | H11 | T:T | 0.4946 | 3.8519 |
| 2031014 | Vlasi-1 | A12 | T:T | 0.53399 | 3.45135 |
| 2031014 | Vlasi-1 | B12 | T:T | 0.50892 | 3.52115 |
| 2031014 | Vlasi-1 | C12 | T:A | 2.154 | 2.55992 |
| 2031014 | Vlasi-1 | D12 | T:A | 2.21642 | 2.73863 |
| 2031014 | Vlasi-1 | E12 | T:A | 2.26248 | 2.57735 |
| 2031014 | Vlasi-1 | F12 | T:T | 0.49446 | 3.65964 |
| 2031014 | Vlasi-1 | G12 | T:T | 0.48346 | 3.56995 |
| 2031014 | Vlasi-1 | H12 | T:T | 0.47741 | 3.57195 |
| 2031014 | Vlasi-2 | A01 | A:A | 3.72872 | 0.70824 |
| 2031014 | Vlasi-2 | B01 | A:A | 3.84178 | 0.60804 |
| 2031014 | Vlasi-2 | C01 | T:A | 2.36131 | 2.60924 |
| 2031014 | Vlasi-2 | D01 | T:T | 0.58782 | 3.79414 |
| 2031014 | Vlasi-2 | E01 | T:T | 0.52307 | 3.75202 |
| 2031014 | Vlasi-2 | F01 | T:A | 2.18845 | 2.70243 |
| 2031014 | Vlasi-2 | G01 | T:A | 2.23545 | 2.50197 |
| 2031014 | Vlasi-2 | H01 | T:T | 0.53205 | 3.80411 |
| 2031014 | Vlasi-2 | A02 | T:T | 0.56942 | 3.42631 |
| 2031014 | Vlasi-2 | B02 | T:A | 2.1985 | 2.62116 |
| 2031014 | Vlasi-2 | C02 | T:A | 2.31868 | 2.67939 |
| 2031014 | Vlasi-2 | D02 | T:T | 0.52195 | 3.71853 |
| 2031014 | Vlasi-2 | E02 | T:T | 0.46887 | 3.5572 |
| 2031014 | Vlasi-2 | F02 | T:T | 0.45839 | 3.64977 |
| 2031014 | Vlasi-2 | G02 | T:A | 2.16171 | 2.57974 |
| 2031014 | Vlasi-2 | H02 | T:T | 0.53566 | 3.76495 |
| 2031014 | Vlasi-2 | A03 | T:A | 2.27858 | 2.40306 |
| 2031014 | Vlasi-2 | B03 | T:A | 2.16915 | 2.57663 |
| 2031014 | Vlasi-2 | C03 | A:A | 4.112 | 0.64535 |
| 2031014 | Vlasi-2 | D03 | T:A | 2.13278 | 2.72037 |
| 2031014 | Vlasi-2 | E03 | T:A | 2.23487 | 2.67362 |

| | | | | | |
|---|---|---|---|---|---|
| 2031014 | Vlasi-2 | F03 | T:A | 2.10588 | 2.45593 |
| 2031014 | Vlasi-2 | G03 | T:A | 2.20543 | 2.61549 |
| 2031014 | Vlasi-2 | H03 | T:A | 2.24806 | 2.46819 |
| 2031014 | Vlasi-2 | A04 | T:A | 2.18799 | 2.41589 |
| 2031014 | Vlasi-2 | B04 | T:A | 2.21635 | 2.55265 |
| 2031014 | Vlasi-2 | C04 | T:T | 0.49889 | 3.71975 |
| 2031014 | Vlasi-2 | D04 | T:A | 2.08543 | 2.45872 |
| 2031014 | Vlasi-2 | E04 | ? | 1.09729 | 0.47483 |
| 2031014 | Vlasi-2 | F04 | A:A | 3.68872 | 0.61569 |
| 2031014 | Vlasi-2 | G04 | T:T | 0.70523 | 3.82929 |
| 2031014 | Vlasi-2 | H04 | T:A | 2.20564 | 2.52434 |
| 2031014 | Vlasi-2 | A05 | T:A | 2.25487 | 2.63922 |
| 2031014 | Vlasi-2 | B05 | A:A | 3.41319 | 0.67807 |
| 2031014 | Vlasi-2 | C05 | T:T | 0.62536 | 3.97114 |
| 2031014 | Vlasi-2 | D05 | T:T | 0.53497 | 3.84531 |
| 2031014 | Vlasi-2 | E05 | T:A | 2.066 | 2.64905 |
| 2031014 | Vlasi-2 | F05 | T:A | 2.02754 | 2.58085 |
| 2031014 | Vlasi-2 | G05 | T:T | 0.55368 | 3.83868 |
| 2031014 | Vlasi-2 | H05 | T:T | 0.5937 | 3.96088 |
| 2031014 | Vlasi-2 | A06 | A:A | 3.67531 | 0.64537 |
| 2031014 | Vlasi-2 | B06 | T:A | 2.2062 | 2.72313 |
| 2031014 | Vlasi-2 | C06 | T:T | 0.66827 | 3.58793 |
| 2031014 | Vlasi-2 | D06 | T:T | 0.51865 | 3.81941 |
| 2031014 | Vlasi-2 | E06 | T:A | 2.25941 | 2.57675 |
| 2031014 | Vlasi-2 | F06 | T:A | 2.17519 | 2.63655 |
| 2031014 | Vlasi-2 | G06 | T:T | 0.53463 | 3.86076 |
| 2031014 | Vlasi-2 | H06 | T:A | 2.24817 | 2.46544 |
| 2031014 | Vlasi-2 | A07 | T:T | 0.47619 | 3.45219 |
| 2031014 | Vlasi-2 | B07 | T:A | 1.99842 | 2.37579 |
| 2031014 | Vlasi-2 | C07 | T:A | 1.79123 | 2.31851 |
| 2031014 | Vlasi-2 | D07 | A:A | 3.35242 | 0.50341 |
| 2031014 | Vlasi-2 | E07 | T:T | 0.61704 | 3.91408 |
| 2031014 | Vlasi-2 | F07 | T:A | 2.09705 | 2.61108 |
| 2031014 | Vlasi-2 | G07 | T:A | 2.12652 | 2.54093 |
| 2031014 | Vlasi-2 | H07 | A:A | 3.95722 | 0.62934 |
| 2031014 | Vlasi-2 | A08 | T:A | 1.98501 | 2.37948 |
| 2031014 | Vlasi-2 | B08 | T:T | 0.51385 | 3.62211 |
| 2031014 | Vlasi-2 | C08 | A:A | 3.22208 | 0.45214 |
| 2031014 | Vlasi-2 | D08 | T:A | 2.12637 | 2.53038 |
| 2031014 | Vlasi-2 | E08 | A:A | 3.86577 | 0.6044 |
| 2031014 | Vlasi-2 | F08 | T:A | 2.14436 | 2.52802 |
| 2031014 | Vlasi-2 | G08 | T:T | 0.53472 | 3.7517 |
| 2031014 | Vlasi-2 | H08 | T:T | 0.542 | 3.70842 |
| 2031014 | Vlasi-2 | A09 | T:A | 2.06028 | 2.35699 |
| 2031014 | Vlasi-2 | B09 | T:A | 2.19379 | 2.49729 |
| 2031014 | Vlasi-2 | C09 | ? | 0.91809 | 0.47149 |
| 2031014 | Vlasi-2 | D09 | T:A | 2.04566 | 2.66769 |
| 2031014 | Vlasi-2 | E09 | A:A | 3.68402 | 0.54076 |
| 2031014 | Vlasi-2 | F09 | T:A | 1.79428 | 2.4925 |
| 2031014 | Vlasi-2 | G09 | T:T | 0.47878 | 3.75541 |

| | | | | | |
|---|---|---|---|---|---|
| 2031014 | Vlasi-2 | H09 | T:T | 0.47968 | 3.55959 |
| 2031014 | Vlasi-2 | A10 | T:T | 0.54037 | 3.31125 |
| 2031014 | Vlasi-2 | B10 | T:T | 0.5265 | 3.42364 |
| 2031014 | Vlasi-2 | C10 | T:T | 0.51143 | 3.66182 |
| 2031014 | Vlasi-2 | D10 | T:A | 2.19584 | 2.5579 |
| 2031014 | Vlasi-2 | E10 | A:A | 3.98794 | 0.6142 |
| 2031014 | Vlasi-2 | F10 | T:A | 2.2333 | 2.65061 |
| 2031014 | Vlasi-2 | G10 | T:A | 2.12279 | 2.48228 |
| 2031014 | Vlasi-2 | H10 | T:T | 0.51638 | 3.69249 |
| 2031014 | Vlasi-2 | A11 | T:T | 0.56128 | 3.42108 |
| 2031014 | Vlasi-2 | B11 | T:A | 2.08435 | 2.52915 |
| 2031014 | Vlasi-2 | C11 | T:T | 0.49204 | 3.55698 |
| 2031014 | Vlasi-2 | D11 | T:A | 2.2084 | 2.52374 |
| 2031014 | Vlasi-2 | E11 | T:T | 0.49905 | 3.68795 |
| 2031014 | Vlasi-2 | F11 | T:A | 2.18759 | 2.62168 |
| 2031014 | Vlasi-2 | G11 | T:T | 0.49106 | 3.66442 |
| 2031014 | Vlasi-2 | H11 | T:A | 2.28754 | 2.59639 |
| 2031014 | Vlasi-2 | A12 | T:T | 0.57477 | 3.50629 |
| 2031014 | Vlasi-2 | B12 | T:A | 2.27671 | 2.52821 |
| 2031014 | Vlasi-2 | C12 | T:T | 0.53859 | 3.43134 |
| 2031014 | Vlasi-2 | D12 | T:T | 0.54013 | 3.60089 |
| 2031014 | Vlasi-2 | E12 | T:A | 2.22727 | 2.55935 |
| 2031014 | Vlasi-2 | F12 | T:T | 0.52285 | 3.42964 |
| 2031014 | Vlasi-2 | G12 | T:T | 0.55209 | 3.93839 |
| 2031014 | Vlasi-2 | H12 | T:A | 2.30202 | 2.6706 |
| 2031014 | Vlasi-3 | A01 | T:A | 2.22156 | 2.70666 |
| 2031014 | Vlasi-3 | B01 | T:T | 0.52877 | 3.65705 |
| 2031014 | Vlasi-3 | C01 | T:T | 0.49642 | 3.69723 |
| 2031014 | Vlasi-3 | D01 | T:T | 0.50663 | 3.75783 |
| 2031014 | Vlasi-3 | E01 | T:T | 0.48267 | 3.53869 |
| 2031014 | Vlasi-3 | F01 | T:T | 0.49297 | 3.68049 |
| 2031014 | Vlasi-3 | G01 | T:T | 0.48696 | 3.48965 |
| 2031014 | Vlasi-3 | H01 | T:T | 0.64383 | 3.67799 |
| 2031014 | Vlasi-3 | A02 | T:T | 0.57623 | 3.45798 |
| 2031014 | Vlasi-3 | B02 | T:A | 2.20561 | 2.59127 |
| 2031014 | Vlasi-3 | C02 | T:T | 0.48891 | 3.7378 |
| 2031014 | Vlasi-3 | D02 | T:T | 0.53043 | 3.71447 |
| 2031014 | Vlasi-3 | E02 | T:A | 2.20786 | 2.53847 |
| 2031014 | Vlasi-3 | F02 | T:T | 0.47519 | 3.5501 |
| 2031014 | Vlasi-3 | G02 | T:T | 0.46858 | 3.47195 |
| 2031014 | Vlasi-3 | H02 | T:A | 2.29846 | 2.71504 |
| 2031014 | Vlasi-3 | A03 | A:A | 3.7302 | 0.63306 |
| 2031014 | Vlasi-3 | B03 | T:T | 0.53801 | 3.56717 |
| 2031014 | Vlasi-3 | C03 | T:A | 2.29332 | 2.52928 |
| 2031014 | Vlasi-3 | D03 | T:A | 2.26006 | 2.46586 |
| 2031014 | Vlasi-3 | E03 | A:A | 3.88059 | 0.55482 |
| 2031014 | Vlasi-3 | F03 | T:T | 0.5734 | 3.85595 |
| 2031014 | Vlasi-3 | G03 | T:A | 1.56046 | 2.17829 |
| 2031014 | Vlasi-3 | H03 | T:A | 2.24664 | 2.59188 |
| 2031014 | Vlasi-3 | A04 | T:T | 0.49635 | 3.44203 |

| | | | | | |
|---|---|---|---|---|---|
| 2031014 | Vlasi-3 | B04 | T:T | 0.50885 | 3.57601 |
| 2031014 | Vlasi-3 | C04 | T:A | 2.20928 | 2.48517 |
| 2031014 | Vlasi-3 | D04 | T:T | 0.47773 | 3.61348 |
| 2031014 | Vlasi-3 | E04 | T:A | 2.09945 | 2.40487 |
| 2031014 | Vlasi-3 | F04 | T:T | 0.45851 | 3.61446 |
| 2031014 | Vlasi-3 | G04 | T:A | 2.19434 | 2.48656 |
| 2031014 | Vlasi-3 | H04 | T:T | 0.65813 | 3.59029 |
| 2031014 | Vlasi-3 | A05 | T:A | 2.21803 | 2.49353 |
| 2031014 | Vlasi-3 | B05 | T:A | 2.15672 | 2.61483 |
| 2031014 | Vlasi-3 | C05 | T:A | 2.09371 | 2.50299 |
| 2031014 | Vlasi-3 | D05 | T:T | 0.58559 | 3.90363 |
| 2031014 | Vlasi-3 | E05 | T:A | 2.02919 | 2.61956 |
| 2031014 | Vlasi-3 | F05 | T:T | 0.48272 | 3.72883 |
| 2031014 | Vlasi-3 | G05 | A:A | 3.76942 | 0.56936 |
| 2031014 | Vlasi-3 | H05 | T:T | 0.69344 | 3.56337 |
| 2031014 | Vlasi-3 | A06 | T:T | 0.51391 | 3.56735 |
| 2031014 | Vlasi-3 | B06 | T:A | 2.12273 | 2.41893 |
| 2031014 | Vlasi-3 | C06 | A:A | 3.63188 | 0.67406 |
| 2031014 | Vlasi-3 | D06 | T:A | 2.15312 | 2.54702 |
| 2031014 | Vlasi-3 | E06 | T:A | 2.17945 | 2.59649 |
| 2031014 | Vlasi-3 | F06 | T:A | 2.26102 | 2.6642 |
| 2031014 | Vlasi-3 | G06 | T:T | 0.46741 | 3.61947 |
| 2031014 | Vlasi-3 | H06 | T:A | 2.3044 | 2.7425 |
| 2031014 | Vlasi-3 | A07 | T:A | 2.05136 | 2.46443 |
| 2031014 | Vlasi-3 | B07 | T:A | 2.19072 | 2.59508 |
| 2031014 | Vlasi-3 | C07 | ? | 0.8295 | 0.44814 |
| 2031014 | Vlasi-3 | D07 | T:T | 0.4674 | 3.79538 |
| 2031014 | Vlasi-3 | E07 | T:A | 2.06791 | 2.45496 |
| 2031014 | Vlasi-3 | F07 | A:A | 3.84511 | 0.58645 |
| 2031014 | Vlasi-3 | G07 | T:T | 0.46607 | 3.57602 |
| 2031014 | Vlasi-3 | H07 | T:T | 0.6323 | 3.52257 |
| 2031014 | Vlasi-3 | A08 | T:A | 2.13851 | 2.53108 |
| 2031014 | Vlasi-3 | B08 | T:A | 2.1443 | 2.61393 |
| 2031014 | Vlasi-3 | C08 | T:A | 2.17917 | 2.42709 |
| 2031014 | Vlasi-3 | D08 | A:A | 3.82377 | 0.63156 |
| 2031014 | Vlasi-3 | E08 | T:T | 0.46976 | 3.67193 |
| 2031014 | Vlasi-3 | F08 | A:A | 3.7505 | 0.57453 |
| 2031014 | Vlasi-3 | G08 | T:A | 2.18827 | 2.58956 |
| 2031014 | Vlasi-3 | H08 | T:A | 2.26465 | 2.56676 |
| 2031014 | Vlasi-3 | A09 | T:A | 2.17174 | 2.4007 |
| 2031014 | Vlasi-3 | B09 | T:A | 2.17018 | 2.58035 |
| 2031014 | Vlasi-3 | C09 | T:A | 2.19276 | 2.55834 |
| 2031014 | Vlasi-3 | D09 | T:T | 0.45039 | 3.51902 |
| 2031014 | Vlasi-3 | E09 | T:A | 2.24265 | 2.58291 |
| 2031014 | Vlasi-3 | F09 | T:A | 2.18893 | 2.52585 |
| 2031014 | Vlasi-3 | G09 | T:T | 0.47957 | 3.57565 |
| 2031014 | Vlasi-3 | H09 | T:A | 2.34807 | 2.60379 |
| 2031014 | Vlasi-3 | A10 | T:A | 1.96846 | 2.42573 |
| 2031014 | Vlasi-3 | B10 | T:A | 2.24521 | 2.4482 |
| 2031014 | Vlasi-3 | C10 | T:A | 2.04866 | 2.45122 |

| | | | | | |
|---|---|---|---|---|---|
| 2031014 | Vlasi-3 | D10 | T:A | 2.19476 | 2.48765 |
| 2031014 | Vlasi-3 | E10 | T:T | 0.45696 | 3.68634 |
| 2031014 | Vlasi-3 | F10 | T:A | 2.18774 | 2.52812 |
| 2031014 | Vlasi-3 | G10 | T:T | 0.47026 | 3.61155 |
| 2031014 | Vlasi-3 | H10 | T:A | 2.38818 | 2.60382 |
| 2031014 | Vlasi-3 | A11 | T:A | 2.31818 | 2.63418 |
| 2031014 | Vlasi-3 | B11 | T:T | 0.47323 | 3.40146 |
| 2031014 | Vlasi-3 | C11 | T:T | 0.47334 | 3.60298 |
| 2031014 | Vlasi-3 | D11 | ? | 0.6321 | 0.42414 |
| 2031014 | Vlasi-3 | E11 | T:A | 2.12984 | 2.53023 |
| 2031014 | Vlasi-3 | F11 | T:A | 2.08056 | 2.61212 |
| 2031014 | Vlasi-3 | G11 | T:A | 2.20519 | 2.59175 |
| 2031014 | Vlasi-3 | H11 | A:A | 3.94827 | 0.76738 |
| 2031014 | Vlasi-3 | A12 | A:A | 3.63193 | 0.52491 |
| 2031014 | Vlasi-3 | B12 | T:T | 0.5363 | 3.61028 |
| 2031014 | Vlasi-3 | C12 | T:T | 0.49848 | 3.51044 |
| 2031014 | Vlasi-3 | D12 | T:A | 2.16818 | 2.65456 |
| 2031014 | Vlasi-3 | E12 | T:A | 2.25263 | 2.66312 |
| 2031014 | Vlasi-3 | F12 | T:A | 2.1804 | 2.50787 |
| 2031014 | Vlasi-3 | G12 | T:A | 2.11511 | 2.54411 |
| 2031014 | Vlasi-3 | H12 | T:T | 0.63531 | 3.37545 |
| 2031014 | Vlasi-4 | A01 | A:A | 3.64104 | 0.62893 |
| 2031014 | Vlasi-4 | B01 | A:A | 3.80748 | 0.58923 |
| 2031014 | Vlasi-4 | C01 | T:T | 0.47325 | 3.70047 |
| 2031014 | Vlasi-4 | D01 | T:A | 2.3106 | 2.64414 |
| 2031014 | Vlasi-4 | E01 | A:A | 3.85475 | 0.53987 |
| 2031014 | Vlasi-4 | F01 | T:T | 0.46231 | 3.67686 |
| 2031014 | Vlasi-4 | G01 | T:T | 0.4983 | 3.80681 |
| 2031014 | Vlasi-4 | H01 | T:A | 2.20592 | 2.55836 |
| 2031014 | Vlasi-4 | A02 | T:A | 2.24147 | 2.56128 |
| 2031014 | Vlasi-4 | B02 | T:T | 0.49273 | 3.58325 |
| 2031014 | Vlasi-4 | C02 | T:A | 1.74008 | 2.6457 |
| 2031014 | Vlasi-4 | D02 | T:T | 0.55383 | 4.09822 |
| 2031014 | Vlasi-4 | E02 | T:A | 2.23863 | 2.61747 |
| 2031014 | Vlasi-4 | F02 | T:A | 2.12999 | 2.47424 |
| 2031014 | Vlasi-4 | G02 | T:T | 0.4543 | 3.50451 |
| 2031014 | Vlasi-4 | H02 | T:A | 2.30819 | 2.59028 |
| 2031014 | Vlasi-4 | A03 | T:A | 2.18948 | 2.45589 |
| 2031014 | Vlasi-4 | B03 | T:A | 2.18007 | 2.5485 |
| 2031014 | Vlasi-4 | C03 | T:A | 2.15207 | 2.48102 |
| 2031014 | Vlasi-4 | D03 | T:A | 2.24926 | 2.614 |
| 2031014 | Vlasi-4 | E03 | T:A | 2.14363 | 2.51222 |
| 2031014 | Vlasi-4 | F03 | T:A | 2.11417 | 2.69019 |
| 2031014 | Vlasi-4 | G03 | T:A | 1.81197 | 2.33791 |
| 2031014 | Vlasi-4 | H03 | T:A | 2.35846 | 2.68546 |
| 2031014 | Vlasi-4 | A04 | T:A | 2.07932 | 2.44039 |
| 2031014 | Vlasi-4 | B04 | T:A | 2.12577 | 2.562 |
| 2031014 | Vlasi-4 | C04 | T:A | 2.05789 | 2.5977 |
| 2031014 | Vlasi-4 | D04 | T:T | 0.45722 | 3.65647 |
| 2031014 | Vlasi-4 | E04 | T:A | 2.09645 | 2.50325 |

| | | | | | |
|---|---|---|---|---|---|
| 2031014 | Vlasi-4 | F04 | T:T | 0.47358 | 3.68645 |
| 2031014 | Vlasi-4 | G04 | T:A | 2.09338 | 2.52319 |
| 2031014 | Vlasi-4 | H04 | T:T | 0.5952 | 3.65945 |
| 2031014 | Vlasi-4 | A05 | T:T | 0.50082 | 3.72203 |
| 2031014 | Vlasi-4 | B05 | T:A | 2.07992 | 2.48324 |
| 2031014 | Vlasi-4 | C05 | T:A | 2.01065 | 2.57265 |
| 2031014 | Vlasi-4 | D05 | T:A | 2.10093 | 2.52779 |
| 2031014 | Vlasi-4 | E05 | T:A | 2.19635 | 2.52675 |
| 2031014 | Vlasi-4 | F05 | T:T | 0.45961 | 3.76437 |
| 2031014 | Vlasi-4 | G05 | A:A | 3.88686 | 0.60798 |
| 2031014 | Vlasi-4 | H05 | T:A | 2.12781 | 2.53227 |
| 2031014 | Vlasi-4 | A06 | A:A | 3.59667 | 0.57669 |
| 2031014 | Vlasi-4 | B06 | T:A | 2.10147 | 2.56052 |
| 2031014 | Vlasi-4 | C06 | T:A | 2.0534 | 2.48719 |
| 2031014 | Vlasi-4 | D06 | T:A | 2.21459 | 2.39859 |
| 2031014 | Vlasi-4 | E06 | T:T | 0.45726 | 3.5593 |
| 2031014 | Vlasi-4 | F06 | T:T | 0.54817 | 3.99603 |
| 2031014 | Vlasi-4 | G06 | T:T | 0.45657 | 3.63485 |
| 2031014 | Vlasi-4 | H06 | T:T | 0.62025 | 3.65902 |
| 2031014 | Vlasi-4 | A07 | ? | 0.67083 | 0.76702 |
| 2031014 | Vlasi-4 | B07 | ? | 0.46133 | 2.38333 |
| 2031014 | Vlasi-4 | C07 | ? | 0.62385 | 1.08571 |
| 2031014 | Vlasi-4 | D07 | ? | 0.77894 | 1.71014 |
| 2031014 | Vlasi-4 | E07 | ? | 0.73915 | 1.27432 |
| 2031014 | Vlasi-4 | F07 | A:A | 3.62675 | 0.72653 |
| 2031014 | Vlasi-4 | G07 | A:A | 3.81936 | 0.70775 |
| 2031014 | Vlasi-4 | H07 | A:A | 3.73266 | 0.863 |
| 2031014 | Vlasi-4 | A08 | A:A | 3.62211 | 0.71027 |
| 2031014 | Vlasi-4 | B08 | A:A | 3.64371 | 0.75785 |
| 2031014 | Vlasi-4 | C08 | A:A | 3.69818 | 0.67388 |
| 2031014 | Vlasi-4 | D08 | A:A | 3.72557 | 0.77923 |
| 2031014 | Vlasi-4 | E08 | A:A | 3.64197 | 0.93006 |
| 2031014 | Vlasi-4 | F08 | A:A | 3.62627 | 0.71382 |
| 2031014 | Vlasi-4 | G08 | A:A | 3.8174 | 0.70956 |
| 2031014 | Vlasi-4 | H08 | A:A | 3.72619 | 0.85499 |
| 2031014 | Vlasi-4 | A09 | A:A | 3.5046 | 0.76903 |
| 2031014 | Vlasi-4 | B09 | A:A | 3.56667 | 0.86738 |
| 2031014 | Vlasi-4 | C09 | A:A | 3.62055 | 0.74955 |
| 2031014 | Vlasi-4 | D09 | A:A | 3.69053 | 0.71722 |
| 2031014 | Vlasi-4 | E09 | A:A | 3.602 | 0.71747 |
| 2031014 | Vlasi-4 | F09 | A:A | 3.73439 | 0.70459 |
| 2031014 | Vlasi-4 | G09 | A:A | 3.70178 | 0.68628 |
| 2031014 | Vlasi-4 | H09 | A:A | 3.81091 | 0.92024 |
| 2031014 | Vlasi-4 | A10 | A:A | 3.59554 | 0.75075 |
| 2031014 | Vlasi-4 | B10 | A:A | 3.61264 | 0.71838 |
| 2031014 | Vlasi-4 | C10 | T:T | 0.46769 | 3.49854 |
| 2031014 | Vlasi-4 | D10 | T:T | 0.46753 | 3.52735 |
| 2031014 | Vlasi-4 | E10 | T:T | 0.50313 | 3.50528 |
| 2031014 | Vlasi-4 | F10 | T:T | 0.47528 | 3.42614 |
| 2031014 | Vlasi-4 | G10 | T:T | 0.50659 | 3.53147 |

| | | | | | |
|---|---|---|---|---|---|
| 2031014 | Vlasi-4 | H10 | T:T | 0.66494 | 3.56431 |
| 2031014 | Vlasi-4 | A11 | T:T | 0.58434 | 3.4944 |
| 2031014 | Vlasi-4 | B11 | T:T | 0.55244 | 3.49578 |
| 2031014 | Vlasi-4 | C11 | T:T | 0.54188 | 3.50492 |
| 2031014 | Vlasi-4 | D11 | T:T | 0.52536 | 3.48712 |
| 2031014 | Vlasi-4 | E11 | T:T | 0.51112 | 3.42504 |
| 2031014 | Vlasi-4 | F11 | T:T | 0.49389 | 3.54909 |
| 2031014 | Vlasi-4 | G11 | T:T | 0.52325 | 3.53358 |
| 2031014 | Vlasi-4 | H11 | T:T | 0.81481 | 3.62345 |
| 2031014 | Vlasi-4 | A12 | T:T | 0.60197 | 3.3209 |
| 2031014 | Vlasi-4 | B12 | T:T | 0.63399 | 3.58204 |
| 2031014 | Vlasi-4 | C12 | T:T | 0.70107 | 3.55008 |
| 2031014 | Vlasi-4 | D12 | T:T | 0.62481 | 3.49078 |
| 2031014 | Vlasi-4 | E12 | T:T | 0.64086 | 3.68564 |
| 2031014 | Vlasi-4 | F12 | T:T | 0.60974 | 3.53764 |
| 2031014 | Vlasi-4 | G12 | T:T | 0.60768 | 3.70168 |
| 2031014 | Vlasi-4 | H12 | NTC | 0.74838 | 2.01161 |
| 2031014 | Vlasi-1 | A01 | C:A | 2.14651 | 2.16763 |
| 2031014 | Vlasi-1 | B01 | C:A | 2.05992 | 2.18014 |
| 2031014 | Vlasi-1 | C01 | C:C | 0.4223 | 3.44354 |
| 2031014 | Vlasi-1 | D01 | C:A | 2.04252 | 2.14169 |
| 2031014 | Vlasi-1 | E01 | C:A | 2.0205 | 2.11119 |
| 2031014 | Vlasi-1 | F01 | C:A | 1.94397 | 2.14099 |
| 2031014 | Vlasi-1 | G01 | C:A | 2.09896 | 1.95117 |
| 2031014 | Vlasi-1 | H01 | C:A | 1.7474 | 2.01463 |
| 2031014 | Vlasi-1 | A02 | C:C | 0.46234 | 3.21622 |
| 2031014 | Vlasi-1 | B02 | A:A | 3.58824 | 0.57614 |
| 2031014 | Vlasi-1 | C02 | C:C | 0.42085 | 3.4153 |
| 2031014 | Vlasi-1 | D02 | C:C | 0.45384 | 3.75683 |
| 2031014 | Vlasi-1 | E02 | C:A | 2.07808 | 2.0329 |
| 2031014 | Vlasi-1 | F02 | A:A | 3.64492 | 0.51762 |
| 2031014 | Vlasi-1 | G02 | C:A | 1.87401 | 1.95745 |
| 2031014 | Vlasi-1 | H02 | C:C | 0.44642 | 3.51489 |
| 2031014 | Vlasi-1 | A03 | A:A | 3.72149 | 0.55292 |
| 2031014 | Vlasi-1 | B03 | A:A | 3.63328 | 0.49633 |
| 2031014 | Vlasi-1 | C03 | C:A | 1.8161 | 2.02485 |
| 2031014 | Vlasi-1 | D03 | C:A | 2.06724 | 2.11663 |
| 2031014 | Vlasi-1 | E03 | C:A | 1.91658 | 1.85825 |
| 2031014 | Vlasi-1 | F03 | C:A | 1.42604 | 1.90085 |
| 2031014 | Vlasi-1 | G03 | C:C | 0.43014 | 3.56573 |
| 2031014 | Vlasi-1 | H03 | C:C | 0.45504 | 3.56765 |
| 2031014 | Vlasi-1 | A04 | C:A | 2.00952 | 2.20984 |
| 2031014 | Vlasi-1 | B04 | C:C | 0.47719 | 3.63259 |
| 2031014 | Vlasi-1 | C04 | C:C | 0.44749 | 3.4855 |
| 2031014 | Vlasi-1 | D04 | C:A | 1.97132 | 2.07183 |
| 2031014 | Vlasi-1 | E04 | A:A | 3.73632 | 0.53841 |
| 2031014 | Vlasi-1 | F04 | A:A | 3.90637 | 0.55246 |
| 2031014 | Vlasi-1 | G04 | C:A | 1.93303 | 2.07333 |
| 2031014 | Vlasi-1 | H04 | C:A | 1.93732 | 2.0284 |
| 2031014 | Vlasi-1 | A05 | C:A | 1.99148 | 2.1184 |

| | | | | | |
|---|---|---|---|---|---|
| 2031014 | Vlasi-1 | B05 | C:A | 1.88526 | 2.02251 |
| 2031014 | Vlasi-1 | C05 | C:C | 0.43235 | 3.46894 |
| 2031014 | Vlasi-1 | D05 | C:A | 1.92101 | 2.28585 |
| 2031014 | Vlasi-1 | E05 | C:A | 1.97955 | 2.09511 |
| 2031014 | Vlasi-1 | F05 | ? | 0.98656 | 0.53926 |
| 2031014 | Vlasi-1 | G05 | C:C | 0.72504 | 2.88039 |
| 2031014 | Vlasi-1 | H05 | A:A | 3.66482 | 0.51148 |
| 2031014 | Vlasi-1 | A06 | C:A | 1.78246 | 2.06515 |
| 2031014 | Vlasi-1 | B06 | C:C | 0.40841 | 3.41662 |
| 2031014 | Vlasi-1 | C06 | A:A | 3.89387 | 0.52247 |
| 2031014 | Vlasi-1 | D06 | A:A | 3.84969 | 0.4763 |
| 2031014 | Vlasi-1 | E06 | C:C | 0.49414 | 3.69569 |
| 2031014 | Vlasi-1 | F06 | A:A | 3.80978 | 0.55019 |
| 2031014 | Vlasi-1 | G06 | C:A | 1.77432 | 2.13248 |
| 2031014 | Vlasi-1 | H06 | C:C | 0.41902 | 3.3326 |
| 2031014 | Vlasi-1 | A07 | C:C | 0.42633 | 3.31576 |
| 2031014 | Vlasi-1 | B07 | A:A | 3.63281 | 0.46886 |
| 2031014 | Vlasi-1 | C07 | A:A | 3.65372 | 0.50846 |
| 2031014 | Vlasi-1 | D07 | C:C | 0.4381 | 3.51591 |
| 2031014 | Vlasi-1 | E07 | C:A | 1.87427 | 2.01597 |
| 2031014 | Vlasi-1 | F07 | C:C | 0.39099 | 3.429 |
| 2031014 | Vlasi-1 | G07 | C:A | 2.00657 | 2.2402 |
| 2031014 | Vlasi-1 | H07 | C:A | 2.01574 | 2.0334 |
| 2031014 | Vlasi-1 | A08 | C:C | 0.43484 | 3.30976 |
| 2031014 | Vlasi-1 | B08 | C:C | 0.46338 | 3.46267 |
| 2031014 | Vlasi-1 | C08 | C:A | 2.06352 | 2.17393 |
| 2031014 | Vlasi-1 | D08 | C:C | 0.46327 | 3.71491 |
| 2031014 | Vlasi-1 | E08 | C:C | 0.42218 | 3.46786 |
| 2031014 | Vlasi-1 | F08 | C:A | 2.00815 | 2.09664 |
| 2031014 | Vlasi-1 | G08 | A:A | 3.76614 | 0.57237 |
| 2031014 | Vlasi-1 | H08 | C:A | 2.00024 | 2.00773 |
| 2031014 | Vlasi-1 | A09 | C:C | 0.40679 | 3.19998 |
| 2031014 | Vlasi-1 | B09 | C:A | 2.01157 | 2.0921 |
| 2031014 | Vlasi-1 | C09 | C:C | 0.40917 | 3.42158 |
| 2031014 | Vlasi-1 | D09 | C:A | 2.14069 | 2.19554 |
| 2031014 | Vlasi-1 | E09 | C:A | 1.99212 | 2.16459 |
| 2031014 | Vlasi-1 | F09 | C:A | 2.1355 | 2.21068 |
| 2031014 | Vlasi-1 | G09 | C:A | 1.91435 | 2.03963 |
| 2031014 | Vlasi-1 | H09 | A:A | 3.53395 | 0.55375 |
| 2031014 | Vlasi-1 | A10 | A:A | 3.55207 | 0.57219 |
| 2031014 | Vlasi-1 | B10 | A:A | 3.55154 | 0.54718 |
| 2031014 | Vlasi-1 | C10 | C:A | 1.8356 | 1.95308 |
| 2031014 | Vlasi-1 | D10 | C:A | 1.86189 | 2.23277 |
| 2031014 | Vlasi-1 | E10 | C:C | 0.41491 | 3.47514 |
| 2031014 | Vlasi-1 | F10 | C:A | 1.96193 | 1.98298 |
| 2031014 | Vlasi-1 | G10 | C:C | 0.40828 | 3.41036 |
| 2031014 | Vlasi-1 | H10 | C:A | 2.04523 | 2.06747 |
| 2031014 | Vlasi-1 | A11 | C:A | 1.97306 | 2.08217 |
| 2031014 | Vlasi-1 | B11 | C:C | 0.43595 | 3.60791 |
| 2031014 | Vlasi-1 | C11 | C:C | 0.40706 | 3.39608 |

| | | | | | |
|---|---|---|---|---|---|
| 2031014 | Vlasi-1 | D11 | C:C | 0.48303 | 3.39461 |
| 2031014 | Vlasi-1 | E11 | C:C | 0.46329 | 3.54216 |
| 2031014 | Vlasi-1 | F11 | C:A | 1.95729 | 2.09033 |
| 2031014 | Vlasi-1 | G11 | C:A | 1.99197 | 1.95111 |
| 2031014 | Vlasi-1 | H11 | C:C | 0.47014 | 3.54702 |
| 2031014 | Vlasi-1 | A12 | C:C | 0.44167 | 3.45342 |
| 2031014 | Vlasi-1 | B12 | C:C | 0.42122 | 3.47121 |
| 2031014 | Vlasi-1 | C12 | C:A | 2.06398 | 2.08373 |
| 2031014 | Vlasi-1 | D12 | A:A | 3.50385 | 0.52661 |
| 2031014 | Vlasi-1 | E12 | C:A | 1.89653 | 2.18461 |
| 2031014 | Vlasi-1 | F12 | C:A | 1.98149 | 1.99453 |
| 2031014 | Vlasi-1 | G12 | C:C | 0.4283 | 3.6551 |
| 2031014 | Vlasi-1 | H12 | C:C | 0.41318 | 3.43159 |
| 2031014 | Vlasi-2 | A01 | C:A | 2.20507 | 2.20532 |
| 2031014 | Vlasi-2 | B01 | C:C | 0.42259 | 3.51408 |
| 2031014 | Vlasi-2 | C01 | C:C | 0.4178 | 3.47544 |
| 2031014 | Vlasi-2 | D01 | C:C | 0.46153 | 3.67866 |
| 2031014 | Vlasi-2 | E01 | C:C | 0.43004 | 3.55733 |
| 2031014 | Vlasi-2 | F01 | C:A | 2.09589 | 2.16629 |
| 2031014 | Vlasi-2 | G01 | C:A | 2.04532 | 2.05845 |
| 2031014 | Vlasi-2 | H01 | C:A | 2.01415 | 2.22603 |
| 2031014 | Vlasi-2 | A02 | C:A | 2.08463 | 2.02249 |
| 2031014 | Vlasi-2 | B02 | C:C | 0.46855 | 3.69792 |
| 2031014 | Vlasi-2 | C02 | C:A | 2.05012 | 1.98332 |
| 2031014 | Vlasi-2 | D02 | C:A | 1.96694 | 2.09123 |
| 2031014 | Vlasi-2 | E02 | C:A | 1.99731 | 2.07412 |
| 2031014 | Vlasi-2 | F02 | A:A | 3.52004 | 0.5192 |
| 2031014 | Vlasi-2 | G02 | C:C | 0.48865 | 3.74754 |
| 2031014 | Vlasi-2 | H02 | C:C | 0.47996 | 3.6418 |
| 2031014 | Vlasi-2 | A03 | C:A | 1.99737 | 2.0739 |
| 2031014 | Vlasi-2 | B03 | C:C | 0.47484 | 3.62973 |
| 2031014 | Vlasi-2 | C03 | C:A | 1.92759 | 1.9343 |
| 2031014 | Vlasi-2 | D03 | C:C | 0.43752 | 3.59053 |
| 2031014 | Vlasi-2 | E03 | C:A | 1.9596 | 2.15772 |
| 2031014 | Vlasi-2 | F03 | A:A | 3.61622 | 0.5477 |
| 2031014 | Vlasi-2 | G03 | C:A | 2.05609 | 2.19072 |
| 2031014 | Vlasi-2 | H03 | C:A | 2.02041 | 2.05782 |
| 2031014 | Vlasi-2 | A04 | A:A | 3.62432 | 0.56275 |
| 2031014 | Vlasi-2 | B04 | C:A | 2.04815 | 2.08792 |
| 2031014 | Vlasi-2 | C04 | C:A | 1.87014 | 2.019 |
| 2031014 | Vlasi-2 | D04 | A:A | 3.59886 | 0.53584 |
| 2031014 | Vlasi-2 | E04 | ? | 1.05812 | 0.8183 |
| 2031014 | Vlasi-2 | F04 | A:A | 3.70561 | 0.50972 |
| 2031014 | Vlasi-2 | G04 | ? | 0.97849 | 0.52998 |
| 2031014 | Vlasi-2 | H04 | C:C | 0.43076 | 3.52456 |
| 2031014 | Vlasi-2 | A05 | C:A | 2.15626 | 2.1404 |
| 2031014 | Vlasi-2 | B05 | C:C | 0.40592 | 3.33177 |
| 2031014 | Vlasi-2 | C05 | C:C | 0.48732 | 3.52615 |
| 2031014 | Vlasi-2 | D05 | A:A | 3.23321 | 0.47143 |
| 2031014 | Vlasi-2 | E05 | C:A | 2.1562 | 2.26283 |

| | | | | | |
|---|---|---|---|---|---|
| 2031014 | Vlasi-2 | F05 | C:C | 0.51035 | 3.74159 |
| 2031014 | Vlasi-2 | G05 | C:C | 0.51152 | 3.77181 |
| 2031014 | Vlasi-2 | H05 | C:A | 2.13281 | 2.28649 |
| 2031014 | Vlasi-2 | A06 | C:A | 2.01286 | 2.07746 |
| 2031014 | Vlasi-2 | B06 | C:A | 1.96074 | 2.11545 |
| 2031014 | Vlasi-2 | C06 | C:A | 1.98815 | 2.08335 |
| 2031014 | Vlasi-2 | D06 | C:A | 2.0355 | 2.20393 |
| 2031014 | Vlasi-2 | E06 | C:A | 1.9444 | 2.08289 |
| 2031014 | Vlasi-2 | F06 | C:C | 0.43366 | 3.53432 |
| 2031014 | Vlasi-2 | G06 | C:C | 0.53468 | 3.60309 |
| 2031014 | Vlasi-2 | H06 | C:C | 0.42445 | 3.30293 |
| 2031014 | Vlasi-2 | A07 | C:A | 2.04454 | 2.05861 |
| 2031014 | Vlasi-2 | B07 | C:A | 2.01886 | 2.0533 |
| 2031014 | Vlasi-2 | C07 | C:A | 1.50651 | 1.75985 |
| 2031014 | Vlasi-2 | D07 | C:A | 1.92108 | 2.16462 |
| 2031014 | Vlasi-2 | E07 | C:A | 1.60712 | 2.09894 |
| 2031014 | Vlasi-2 | F07 | A:A | 3.65751 | 0.53164 |
| 2031014 | Vlasi-2 | G07 | C:A | 2.06191 | 2.17215 |
| 2031014 | Vlasi-2 | H07 | C:A | 2.00608 | 2.10151 |
| 2031014 | Vlasi-2 | A08 | C:C | 0.47944 | 3.46973 |
| 2031014 | Vlasi-2 | B08 | C:C | 0.44645 | 3.62516 |
| 2031014 | Vlasi-2 | C08 | A:A | 2.6504 | 0.38444 |
| 2031014 | Vlasi-2 | D08 | A:A | 3.63214 | 0.47121 |
| 2031014 | Vlasi-2 | E08 | C:C | 0.42062 | 3.40841 |
| 2031014 | Vlasi-2 | F08 | C:A | 1.88576 | 2.09676 |
| 2031014 | Vlasi-2 | G08 | C:C | 0.48942 | 3.4363 |
| 2031014 | Vlasi-2 | H08 | C:C | 0.44425 | 3.55188 |
| 2031014 | Vlasi-2 | A09 | ? | 0.70018 | 0.52552 |
| 2031014 | Vlasi-2 | B09 | C:A | 1.97419 | 2.02607 |
| 2031014 | Vlasi-2 | C09 | ? | 0.92549 | 0.42771 |
| 2031014 | Vlasi-2 | D09 | C:C | 0.46475 | 3.56165 |
| 2031014 | Vlasi-2 | E09 | C:C | 0.56047 | 3.61702 |
| 2031014 | Vlasi-2 | F09 | A:A | 2.70469 | 0.41289 |
| 2031014 | Vlasi-2 | G09 | A:A | 3.72713 | 0.53099 |
| 2031014 | Vlasi-2 | H09 | C:C | 0.4386 | 3.43647 |
| 2031014 | Vlasi-2 | A10 | C:C | 0.50693 | 3.3522 |
| 2031014 | Vlasi-2 | B10 | C:C | 0.43397 | 3.44451 |
| 2031014 | Vlasi-2 | C10 | C:A | 2.00627 | 2.10668 |
| 2031014 | Vlasi-2 | D10 | C:A | 1.97879 | 2.09988 |
| 2031014 | Vlasi-2 | E10 | C:A | 1.94502 | 2.19724 |
| 2031014 | Vlasi-2 | F10 | C:C | 0.58418 | 3.66359 |
| 2031014 | Vlasi-2 | G10 | C:A | 2.00884 | 2.16685 |
| 2031014 | Vlasi-2 | H10 | C:A | 1.96379 | 2.21864 |
| 2031014 | Vlasi-2 | A11 | C:A | 1.67396 | 1.66556 |
| 2031014 | Vlasi-2 | B11 | A:A | 3.63755 | 0.56215 |
| 2031014 | Vlasi-2 | C11 | C:A | 2.0405 | 2.142 |
| 2031014 | Vlasi-2 | D11 | C:A | 2.03059 | 2.14547 |
| 2031014 | Vlasi-2 | E11 | C:C | 0.42948 | 3.53725 |
| 2031014 | Vlasi-2 | F11 | A:A | 3.80529 | 0.55174 |
| 2031014 | Vlasi-2 | G11 | C:A | 1.92362 | 2.04845 |

| | | | | | |
|---|---|---|---|---|---|
| 2031014 | Vlasi-2 | H11 | C:C | 0.43832 | 3.17545 |
| 2031014 | Vlasi-2 | A12 | C:C | 0.48162 | 3.3656 |
| 2031014 | Vlasi-2 | B12 | A:A | 3.68587 | 0.59448 |
| 2031014 | Vlasi-2 | C12 | C:A | 2.15699 | 2.09128 |
| 2031014 | Vlasi-2 | D12 | C:A | 2.10423 | 2.10435 |
| 2031014 | Vlasi-2 | E12 | C:C | 0.47122 | 3.54086 |
| 2031014 | Vlasi-2 | F12 | C:A | 2.04606 | 2.17789 |
| 2031014 | Vlasi-2 | G12 | C:A | 2.02389 | 2.14264 |
| 2031014 | Vlasi-2 | H12 | C:C | 0.4706 | 3.66067 |
| 2031014 | Vlasi-3 | A01 | C:C | 0.43473 | 3.56048 |
| 2031014 | Vlasi-3 | B01 | C:A | 2.0704 | 1.80696 |
| 2031014 | Vlasi-3 | C01 | A:A | 3.56525 | 0.52036 |
| 2031014 | Vlasi-3 | D01 | C:C | 0.41694 | 3.51091 |
| 2031014 | Vlasi-3 | E01 | C:A | 2.1654 | 1.88013 |
| 2031014 | Vlasi-3 | F01 | A:A | 3.88084 | 0.5438 |
| 2031014 | Vlasi-3 | G01 | C:C | 0.40887 | 3.40844 |
| 2031014 | Vlasi-3 | H01 | A:A | 3.57122 | 0.56295 |
| 2031014 | Vlasi-3 | A02 | C:A | 2.03304 | 2.09743 |
| 2031014 | Vlasi-3 | B02 | ? | 0.65311 | 0.44028 |
| 2031014 | Vlasi-3 | C02 | C:A | 1.78596 | 2.12858 |
| 2031014 | Vlasi-3 | D02 | C:C | 0.41475 | 3.50606 |
| 2031014 | Vlasi-3 | E02 | C:A | 1.93802 | 1.97024 |
| 2031014 | Vlasi-3 | F02 | C:A | 1.88432 | 2.01845 |
| 2031014 | Vlasi-3 | G02 | C:A | 2.00611 | 1.99062 |
| 2031014 | Vlasi-3 | H02 | C:C | 0.4637 | 3.5166 |
| 2031014 | Vlasi-3 | A03 | C:A | 1.89227 | 2.01404 |
| 2031014 | Vlasi-3 | B03 | A:A | 4.10881 | 0.56147 |
| 2031014 | Vlasi-3 | C03 | C:C | 0.46043 | 3.1199 |
| 2031014 | Vlasi-3 | D03 | C:A | 1.97553 | 1.96779 |
| 2031014 | Vlasi-3 | E03 | C:C | 0.41184 | 3.5282 |
| 2031014 | Vlasi-3 | F03 | C:C | 0.49966 | 3.69425 |
| 2031014 | Vlasi-3 | G03 | C:A | 1.51667 | 2.04487 |
| 2031014 | Vlasi-3 | H03 | A:A | 3.59504 | 0.611 |
| 2031014 | Vlasi-3 | A04 | A:A | 3.31031 | 0.49622 |
| 2031014 | Vlasi-3 | B04 | C:A | 1.94507 | 2.04268 |
| 2031014 | Vlasi-3 | C04 | A:A | 3.70194 | 0.56253 |
| 2031014 | Vlasi-3 | D04 | C:A | 1.92715 | 2.0785 |
| 2031014 | Vlasi-3 | E04 | C:A | 1.98187 | 1.93051 |
| 2031014 | Vlasi-3 | F04 | C:C | 0.41511 | 3.54153 |
| 2031014 | Vlasi-3 | G04 | C:C | 0.48124 | 3.65733 |
| 2031014 | Vlasi-3 | H04 | C:C | 0.48151 | 3.63648 |
| 2031014 | Vlasi-3 | A05 | A:A | 3.53245 | 0.52489 |
| 2031014 | Vlasi-3 | B05 | C:A | 1.92448 | 1.9749 |
| 2031014 | Vlasi-3 | C05 | C:C | 0.40921 | 3.40042 |
| 2031014 | Vlasi-3 | D05 | C:A | 2.06716 | 2.30633 |
| 2031014 | Vlasi-3 | E05 | C:A | 2.06247 | 2.07916 |
| 2031014 | Vlasi-3 | F05 | C:A | 1.95532 | 1.89336 |
| 2031014 | Vlasi-3 | G05 | A:A | 3.58895 | 0.54477 |
| 2031014 | Vlasi-3 | H05 | C:A | 2.05309 | 2.19831 |
| 2031014 | Vlasi-3 | A06 | C:A | 2.10768 | 1.98912 |

| | | | | | |
|---|---|---|---|---|---|
| 2031014 | Vlasi-3 | B06 | A:A | 3.54456 | 0.53096 |
| 2031014 | Vlasi-3 | C06 | C:A | 1.94134 | 2.07313 |
| 2031014 | Vlasi-3 | D06 | C:C | 0.41466 | 3.48814 |
| 2031014 | Vlasi-3 | E06 | C:A | 2.01678 | 2.00733 |
| 2031014 | Vlasi-3 | F06 | C:C | 0.41813 | 3.54957 |
| 2031014 | Vlasi-3 | G06 | C:A | 1.8875 | 1.99536 |
| 2031014 | Vlasi-3 | H06 | C:A | 1.99967 | 2.20202 |
| 2031014 | Vlasi-3 | A07 | C:A | 2.06833 | 2.02126 |
| 2031014 | Vlasi-3 | B07 | C:C | 0.4074 | 3.40745 |
| 2031014 | Vlasi-3 | C07 | C:A | 1.97011 | 1.99384 |
| 2031014 | Vlasi-3 | D07 | C:C | 0.41927 | 3.65407 |
| 2031014 | Vlasi-3 | E07 | C:A | 1.81491 | 2.1377 |
| 2031014 | Vlasi-3 | F07 | C:C | 0.42694 | 3.58091 |
| 2031014 | Vlasi-3 | G07 | C:C | 0.45124 | 3.31847 |
| 2031014 | Vlasi-3 | H07 | C:C | 0.46816 | 3.49381 |
| 2031014 | Vlasi-3 | A08 | C:C | 0.43591 | 3.47617 |
| 2031014 | Vlasi-3 | B08 | C:A | 2.02896 | 2.07471 |
| 2031014 | Vlasi-3 | C08 | C:A | 1.94358 | 2.09792 |
| 2031014 | Vlasi-3 | D08 | A:A | 3.68817 | 0.54451 |
| 2031014 | Vlasi-3 | E08 | C:A | 1.94603 | 2.113 |
| 2031014 | Vlasi-3 | F08 | C:C | 0.40659 | 3.5268 |
| 2031014 | Vlasi-3 | G08 | A:A | 3.59541 | 0.49005 |
| 2031014 | Vlasi-3 | H08 | C:A | 2.16818 | 2.37635 |
| 2031014 | Vlasi-3 | A09 | A:A | 3.6662 | 0.54631 |
| 2031014 | Vlasi-3 | B09 | C:C | 0.39798 | 3.33108 |
| 2031014 | Vlasi-3 | C09 | C:A | 2.1067 | 2.11587 |
| 2031014 | Vlasi-3 | D09 | C:A | 1.79707 | 2.12589 |
| 2031014 | Vlasi-3 | E09 | C:A | 1.89855 | 1.96768 |
| 2031014 | Vlasi-3 | F09 | C:C | 0.41712 | 3.6363 |
| 2031014 | Vlasi-3 | G09 | A:A | 3.7009 | 0.50733 |
| 2031014 | Vlasi-3 | H09 | A:A | 3.78341 | 0.60108 |
| 2031014 | Vlasi-3 | A10 | C:C | 0.41072 | 3.30492 |
| 2031014 | Vlasi-3 | B10 | C:A | 1.81898 | 2.05249 |
| 2031014 | Vlasi-3 | C10 | C:A | 1.9562 | 1.96029 |
| 2031014 | Vlasi-3 | D10 | C:C | 0.41929 | 3.49593 |
| 2031014 | Vlasi-3 | E10 | C:C | 0.41267 | 3.44818 |
| 2031014 | Vlasi-3 | F10 | C:A | 2.02732 | 2.08191 |
| 2031014 | Vlasi-3 | G10 | C:A | 1.83757 | 2.1047 |
| 2031014 | Vlasi-3 | H10 | C:C | 0.4682 | 3.51112 |
| 2031014 | Vlasi-3 | A11 | C:A | 1.91087 | 1.97894 |
| 2031014 | Vlasi-3 | B11 | C:C | 0.40657 | 3.30187 |
| 2031014 | Vlasi-3 | C11 | C:A | 1.82625 | 2.13772 |
| 2031014 | Vlasi-3 | D11 | A:A | 3.79478 | 0.60848 |
| 2031014 | Vlasi-3 | E11 | C:A | 1.98939 | 2.15289 |
| 2031014 | Vlasi-3 | F11 | C:A | 1.98292 | 2.10144 |
| 2031014 | Vlasi-3 | G11 | C:C | 0.41384 | 3.43737 |
| 2031014 | Vlasi-3 | H11 | C:A | 2.10267 | 2.22844 |
| 2031014 | Vlasi-3 | A12 | A:A | 3.47774 | 0.51716 |
| 2031014 | Vlasi-3 | B12 | C:A | 2.06734 | 2.00713 |
| 2031014 | Vlasi-3 | C12 | C:C | 0.42832 | 3.48628 |

| | | | | | |
|---|---|---|---|---|---|
| 2031014 | Vlasi-3 | D12 | A:A | 3.60882 | 0.53139 |
| 2031014 | Vlasi-3 | E12 | A:A | 3.63131 | 0.52913 |
| 2031014 | Vlasi-3 | F12 | C:A | 2.07671 | 2.16734 |
| 2031014 | Vlasi-3 | G12 | C:C | 0.69816 | 3.23541 |
| 2031014 | Vlasi-3 | H12 | C:A | 1.98849 | 2.09136 |
| 2031014 | Vlasi-4 | A01 | C:C | 0.42934 | 3.50702 |
| 2031014 | Vlasi-4 | B01 | C:A | 2.04853 | 2.1394 |
| 2031014 | Vlasi-4 | C01 | C:A | 2.02051 | 2.10793 |
| 2031014 | Vlasi-4 | D01 | C:A | 1.90246 | 2.10133 |
| 2031014 | Vlasi-4 | E01 | C:C | 0.40044 | 3.35718 |
| 2031014 | Vlasi-4 | F01 | A:A | 3.65706 | 0.49576 |
| 2031014 | Vlasi-4 | G01 | C:C | 0.42645 | 3.40746 |
| 2031014 | Vlasi-4 | H01 | C:A | 2.06633 | 2.04148 |
| 2031014 | Vlasi-4 | A02 | C:A | 2.03666 | 2.1005 |
| 2031014 | Vlasi-4 | B02 | C:A | 2.01418 | 2.02257 |
| 2031014 | Vlasi-4 | C02 | C:A | 2.15508 | 2.4259 |
| 2031014 | Vlasi-4 | D02 | C:C | 0.46417 | 3.66774 |
| 2031014 | Vlasi-4 | E02 | C:A | 1.93806 | 2.1809 |
| 2031014 | Vlasi-4 | F02 | C:A | 2.02343 | 1.98307 |
| 2031014 | Vlasi-4 | G02 | C:A | 1.95873 | 2.07057 |
| 2031014 | Vlasi-4 | H02 | C:C | 0.4569 | 3.39929 |
| 2031014 | Vlasi-4 | A03 | C:A | 1.98495 | 1.85512 |
| 2031014 | Vlasi-4 | B03 | C:A | 1.99151 | 2.06704 |
| 2031014 | Vlasi-4 | C03 | C:C | 0.52494 | 3.42109 |
| 2031014 | Vlasi-4 | D03 | A:A | 3.54854 | 0.55734 |
| 2031014 | Vlasi-4 | E03 | C:A | 1.9314 | 2.07683 |
| 2031014 | Vlasi-4 | F03 | C:C | 0.42577 | 3.64575 |
| 2031014 | Vlasi-4 | G03 | C:C | 0.41489 | 3.46826 |
| 2031014 | Vlasi-4 | H03 | C:A | 2.2002 | 2.06379 |
| 2031014 | Vlasi-4 | A04 | C:A | 2.04962 | 2.13772 |
| 2031014 | Vlasi-4 | B04 | C:C | 0.41055 | 3.54997 |
| 2031014 | Vlasi-4 | C04 | C:C | 0.40536 | 3.38373 |
| 2031014 | Vlasi-4 | D04 | C:C | 0.41648 | 3.5667 |
| 2031014 | Vlasi-4 | E04 | C:A | 1.93305 | 2.05196 |
| 2031014 | Vlasi-4 | F04 | C:A | 1.71175 | 1.99536 |
| 2031014 | Vlasi-4 | G04 | C:C | 0.42684 | 3.49449 |
| 2031014 | Vlasi-4 | H04 | C:C | 0.46245 | 3.34959 |
| 2031014 | Vlasi-4 | A05 | C:A | 1.87263 | 2.09787 |
| 2031014 | Vlasi-4 | B05 | C:C | 0.3893 | 3.4293 |
| 2031014 | Vlasi-4 | C05 | C:C | 0.40688 | 3.43418 |
| 2031014 | Vlasi-4 | D05 | C:C | 0.37454 | 3.29153 |
| 2031014 | Vlasi-4 | E05 | A:A | 3.55537 | 0.50175 |
| 2031014 | Vlasi-4 | F05 | C:C | 0.39363 | 3.36103 |
| 2031014 | Vlasi-4 | G05 | C:A | 1.94277 | 1.96155 |
| 2031014 | Vlasi-4 | H05 | C:C | 0.47161 | 3.4809 |
| 2031014 | Vlasi-4 | A06 | C:C | 0.43132 | 3.36787 |
| 2031014 | Vlasi-4 | B06 | C:A | 2.01865 | 2.05308 |
| 2031014 | Vlasi-4 | C06 | C:C | 0.40876 | 3.53579 |
| 2031014 | Vlasi-4 | D06 | C:C | 0.40556 | 3.42555 |
| 2031014 | Vlasi-4 | E06 | C:A | 1.78849 | 1.90825 |

| | | | | | |
|---|---|---|---|---|---|
| 2031014 | Vlasi-4 | F06 | C:C | 0.56225 | 3.8175 |
| 2031014 | Vlasi-4 | G06 | C:A | 1.83178 | 2.10866 |
| 2031014 | Vlasi-4 | H06 | C:A | 2.11273 | 2.23574 |
| 2031014 | Vlasi-4 | A07 | ? | 0.54515 | 0.45901 |
| 2031014 | Vlasi-4 | B07 | ? | 0.58127 | 0.46901 |
| 2031014 | Vlasi-4 | C07 | ? | 0.56644 | 0.45613 |
| 2031014 | Vlasi-4 | D07 | ? | 0.5816 | 0.46343 |
| 2031014 | Vlasi-4 | E07 | ? | 0.57219 | 0.48522 |
| 2031014 | Vlasi-4 | F07 | A:A | 3.14959 | 0.54452 |
| 2031014 | Vlasi-4 | G07 | A:A | 3.53537 | 0.53865 |
| 2031014 | Vlasi-4 | H07 | A:A | 3.53397 | 0.59659 |
| 2031014 | Vlasi-4 | A08 | A:A | 3.22213 | 0.50897 |
| 2031014 | Vlasi-4 | B08 | A:A | 2.80088 | 0.50794 |
| 2031014 | Vlasi-4 | C08 | A:A | 3.17645 | 0.47402 |
| 2031014 | Vlasi-4 | D08 | A:A | 3.29342 | 0.52647 |
| 2031014 | Vlasi-4 | E08 | A:A | 3.12626 | 0.49523 |
| 2031014 | Vlasi-4 | F08 | A:A | 2.84022 | 0.52478 |
| 2031014 | Vlasi-4 | G08 | A:A | 3.21741 | 0.51617 |
| 2031014 | Vlasi-4 | H08 | A:A | 3.12636 | 0.60195 |
| 2031014 | Vlasi-4 | A09 | A:A | 3.14355 | 0.51067 |
| 2031014 | Vlasi-4 | B09 | A:A | 3.22486 | 0.50796 |
| 2031014 | Vlasi-4 | C09 | A:A | 3.0726 | 0.56404 |
| 2031014 | Vlasi-4 | D09 | A:A | 3.01249 | 0.50475 |
| 2031014 | Vlasi-4 | E09 | A:A | 2.94262 | 0.50941 |
| 2031014 | Vlasi-4 | F09 | A:A | 3.00216 | 0.54607 |
| 2031014 | Vlasi-4 | G09 | A:A | 2.80674 | 0.50867 |
| 2031014 | Vlasi-4 | H09 | A:A | 3.11619 | 0.57678 |
| 2031014 | Vlasi-4 | A10 | A:A | 3.09436 | 0.47701 |
| 2031014 | Vlasi-4 | B10 | A:A | 2.84715 | 0.52091 |
| 2031014 | Vlasi-4 | C10 | C:C | 0.40711 | 3.1026 |
| 2031014 | Vlasi-4 | D10 | C:C | 0.40072 | 3.19614 |
| 2031014 | Vlasi-4 | E10 | C:C | 0.44747 | 3.28797 |
| 2031014 | Vlasi-4 | F10 | C:C | 0.42499 | 3.08376 |
| 2031014 | Vlasi-4 | G10 | C:C | 0.42144 | 3.10245 |
| 2031014 | Vlasi-4 | H10 | C:C | 0.46731 | 3.12033 |
| 2031014 | Vlasi-4 | A11 | C:C | 0.42056 | 3.30761 |
| 2031014 | Vlasi-4 | B11 | C:C | 0.42651 | 3.08884 |
| 2031014 | Vlasi-4 | C11 | C:C | 0.41294 | 3.3185 |
| 2031014 | Vlasi-4 | D11 | C:C | 0.42121 | 2.85808 |
| 2031014 | Vlasi-4 | E11 | C:C | 0.43211 | 3.09616 |
| 2031014 | Vlasi-4 | F11 | C:C | 0.42325 | 2.91251 |
| 2031014 | Vlasi-4 | G11 | C:C | 0.41714 | 3.07363 |
| 2031014 | Vlasi-4 | H11 | C:C | 0.4712 | 3.18101 |
| 2031014 | Vlasi-4 | A12 | C:C | 0.46374 | 3.18768 |
| 2031014 | Vlasi-4 | B12 | C:C | 0.4302 | 3.12115 |
| 2031014 | Vlasi-4 | C12 | C:C | 0.42693 | 3.28937 |
| 2031014 | Vlasi-4 | D12 | C:C | 0.45647 | 3.0185 |
| 2031014 | Vlasi-4 | E12 | ? | 0.47595 | 2.18433 |
| 2031014 | Vlasi-4 | F12 | C:C | 0.44809 | 3.29227 |
| 2031014 | Vlasi-4 | G12 | C:C | 0.45509 | 3.272 |

| | | | | | |
|---|---|---|---|---|---|
| 2031014 | Vlasi-4 | H12 | NTC | 0.6684 | 0.59769 |
| 2031015 | Vlasi-1 | A01 | G:G | 0.43298 | 3.59501 |
| 2031015 | Vlasi-1 | B01 | G:G | 0.3862 | 3.64495 |
| 2031015 | Vlasi-1 | C01 | G:G | 0.37863 | 3.66597 |
| 2031015 | Vlasi-1 | D01 | G:A | 2.09829 | 2.23126 |
| 2031015 | Vlasi-1 | E01 | A:A | 3.65907 | 0.47756 |
| 2031015 | Vlasi-1 | F01 | G:G | 0.39351 | 3.56378 |
| 2031015 | Vlasi-1 | G01 | G:G | 0.3859 | 3.60832 |
| 2031015 | Vlasi-1 | H01 | G:A | 2.01789 | 2.15981 |
| 2031015 | Vlasi-1 | A02 | G:A | 2.19352 | 2.20348 |
| 2031015 | Vlasi-1 | B02 | G:G | 0.53142 | 3.44735 |
| 2031015 | Vlasi-1 | C02 | G:G | 0.40005 | 3.55026 |
| 2031015 | Vlasi-1 | D02 | G:G | 0.39881 | 3.60358 |
| 2031015 | Vlasi-1 | E02 | G:A | 1.92931 | 1.92376 |
| 2031015 | Vlasi-1 | F02 | G:A | 2.22737 | 2.06308 |
| 2031015 | Vlasi-1 | G02 | G:A | 2.14054 | 2.16236 |
| 2031015 | Vlasi-1 | H02 | G:G | 0.4008 | 3.47967 |
| 2031015 | Vlasi-1 | A03 | G:A | 2.08817 | 2.14855 |
| 2031015 | Vlasi-1 | B03 | ? | 0.92064 | 0.41468 |
| 2031015 | Vlasi-1 | C03 | G:G | 0.37785 | 3.55568 |
| 2031015 | Vlasi-1 | D03 | G:A | 2.02075 | 2.12012 |
| 2031015 | Vlasi-1 | E03 | G:G | 0.37978 | 3.44596 |
| 2031015 | Vlasi-1 | F03 | G:G | 0.39618 | 3.71172 |
| 2031015 | Vlasi-1 | G03 | G:A | 2.03399 | 2.26865 |
| 2031015 | Vlasi-1 | H03 | G:G | 0.40726 | 3.74222 |
| 2031015 | Vlasi-1 | A04 | G:G | 0.42659 | 3.57194 |
| 2031015 | Vlasi-1 | B04 | G:G | 0.40724 | 3.55345 |
| 2031015 | Vlasi-1 | C04 | G:A | 2.19209 | 2.19434 |
| 2031015 | Vlasi-1 | D04 | G:G | 0.39107 | 3.47629 |
| 2031015 | Vlasi-1 | E04 | G:A | 2.05234 | 2.18625 |
| 2031015 | Vlasi-1 | F04 | G:A | 2.08872 | 2.20188 |
| 2031015 | Vlasi-1 | G04 | G:A | 2.01297 | 2.01892 |
| 2031015 | Vlasi-1 | H04 | G:A | 2.19238 | 2.19361 |
| 2031015 | Vlasi-1 | A05 | G:A | 2.06971 | 2.28195 |
| 2031015 | Vlasi-1 | B05 | G:G | 0.47049 | 3.493 |
| 2031015 | Vlasi-1 | C05 | G:G | 0.41512 | 3.73383 |
| 2031015 | Vlasi-1 | D05 | G:G | 0.4036 | 3.65518 |
| 2031015 | Vlasi-1 | E05 | G:G | 0.38192 | 3.51161 |
| 2031015 | Vlasi-1 | F05 | ? | 0.99548 | 0.44075 |
| 2031015 | Vlasi-1 | G05 | G:G | 0.47925 | 3.68735 |
| 2031015 | Vlasi-1 | H05 | G:G | 0.41714 | 3.71342 |
| 2031015 | Vlasi-1 | A06 | G:A | 2.01338 | 2.24742 |
| 2031015 | Vlasi-1 | B06 | G:G | 0.42128 | 3.53469 |
| 2031015 | Vlasi-1 | C06 | G:G | 0.41451 | 3.8167 |
| 2031015 | Vlasi-1 | D06 | G:A | 2.19053 | 1.89635 |
| 2031015 | Vlasi-1 | E06 | G:A | 2.02581 | 2.30257 |
| 2031015 | Vlasi-1 | F06 | G:A | 1.94521 | 2.11829 |
| 2031015 | Vlasi-1 | G06 | G:A | 1.99629 | 2.21709 |
| 2031015 | Vlasi-1 | H06 | G:A | 2.17143 | 2.14084 |
| 2031015 | Vlasi-1 | A07 | G:G | 0.42595 | 3.24046 |

| | | | | | |
|---|---|---|---|---|---|
| 2031015 | Vlasi-1 | B07 | G:G | 0.46328 | 3.5299 |
| 2031015 | Vlasi-1 | C07 | G:A | 2.04245 | 2.07284 |
| 2031015 | Vlasi-1 | D07 | G:A | 2.1228 | 1.92058 |
| 2031015 | Vlasi-1 | E07 | G:A | 2.07595 | 2.23278 |
| 2031015 | Vlasi-1 | F07 | G:G | 0.39082 | 3.72393 |
| 2031015 | Vlasi-1 | G07 | A:A | 3.42457 | 0.53345 |
| 2031015 | Vlasi-1 | H07 | A:A | 3.48593 | 0.50652 |
| 2031015 | Vlasi-1 | A08 | G:A | 2.2612 | 2.12052 |
| 2031015 | Vlasi-1 | B08 | G:G | 0.47318 | 3.51274 |
| 2031015 | Vlasi-1 | C08 | G:G | 0.42263 | 3.59198 |
| 2031015 | Vlasi-1 | D08 | G:G | 0.39786 | 3.61087 |
| 2031015 | Vlasi-1 | E08 | G:G | 0.39189 | 3.69773 |
| 2031015 | Vlasi-1 | F08 | G:G | 0.38436 | 3.55234 |
| 2031015 | Vlasi-1 | G08 | G:G | 0.43996 | 3.63892 |
| 2031015 | Vlasi-1 | H08 | G:A | 2.15874 | 2.14929 |
| 2031015 | Vlasi-1 | A09 | G:A | 2.13844 | 2.27762 |
| 2031015 | Vlasi-1 | B09 | G:G | 0.38513 | 3.54863 |
| 2031015 | Vlasi-1 | C09 | G:A | 2.01301 | 2.11162 |
| 2031015 | Vlasi-1 | D09 | A:A | 2.98349 | 0.40097 |
| 2031015 | Vlasi-1 | E09 | G:G | 0.38668 | 3.60681 |
| 2031015 | Vlasi-1 | F09 | G:A | 1.9683 | 2.13095 |
| 2031015 | Vlasi-1 | G09 | G:G | 0.39528 | 3.7186 |
| 2031015 | Vlasi-1 | H09 | G:G | 0.39938 | 3.6317 |
| 2031015 | Vlasi-1 | A10 | G:A | 2.22398 | 2.04949 |
| 2031015 | Vlasi-1 | B10 | G:G | 0.3953 | 3.59707 |
| 2031015 | Vlasi-1 | C10 | G:G | 0.40198 | 3.55951 |
| 2031015 | Vlasi-1 | D10 | G:G | 0.39003 | 3.55609 |
| 2031015 | Vlasi-1 | E10 | G:G | 0.39435 | 3.77702 |
| 2031015 | Vlasi-1 | F10 | G:A | 2.04602 | 2.29801 |
| 2031015 | Vlasi-1 | G10 | G:G | 0.40147 | 3.56934 |
| 2031015 | Vlasi-1 | H10 | G:G | 0.4092 | 3.54764 |
| 2031015 | Vlasi-1 | A11 | G:G | 0.47625 | 3.44569 |
| 2031015 | Vlasi-1 | B11 | G:G | 0.40385 | 3.67965 |
| 2031015 | Vlasi-1 | C11 | G:A | 2.31908 | 2.04403 |
| 2031015 | Vlasi-1 | D11 | G:A | 2.07465 | 2.2448 |
| 2031015 | Vlasi-1 | E11 | G:G | 0.42608 | 3.70932 |
| 2031015 | Vlasi-1 | F11 | G:G | 0.39824 | 3.79801 |
| 2031015 | Vlasi-1 | G11 | G:G | 0.39217 | 3.57935 |
| 2031015 | Vlasi-1 | H11 | G:G | 0.40599 | 3.65721 |
| 2031015 | Vlasi-1 | A12 | G:G | 0.41926 | 3.42992 |
| 2031015 | Vlasi-1 | B12 | G:G | 0.37447 | 3.53218 |
| 2031015 | Vlasi-1 | C12 | G:G | 0.38138 | 3.58233 |
| 2031015 | Vlasi-1 | D12 | G:G | 0.40285 | 3.60781 |
| 2031015 | Vlasi-1 | E12 | G:A | 2.16997 | 2.23623 |
| 2031015 | Vlasi-1 | F12 | G:A | 2.28212 | 2.09249 |
| 2031015 | Vlasi-1 | G12 | G:A | 2.13372 | 2.3034 |
| 2031015 | Vlasi-1 | H12 | G:G | 0.41515 | 3.73879 |
| 2031015 | Vlasi-2 | A01 | G:G | 0.45586 | 3.59794 |
| 2031015 | Vlasi-2 | B01 | G:G | 0.40783 | 3.51618 |
| 2031015 | Vlasi-2 | C01 | G:A | 2.15154 | 2.24712 |

| | | | | | |
|---|---|---|---|---|---|
| 2031015 | Vlasi-2 | D01 | ? | 0.7681 | 0.45844 |
| 2031015 | Vlasi-2 | E01 | G:G | 0.54685 | 3.44689 |
| 2031015 | Vlasi-2 | F01 | G:G | 0.51942 | 3.71534 |
| 2031015 | Vlasi-2 | G01 | G:G | 0.50732 | 3.6545 |
| 2031015 | Vlasi-2 | H01 | G:A | 2.1034 | 2.11884 |
| 2031015 | Vlasi-2 | A02 | G:G | 0.43374 | 3.5301 |
| 2031015 | Vlasi-2 | B02 | G:G | 0.52079 | 3.48369 |
| 2031015 | Vlasi-2 | C02 | G:A | 2.1474 | 2.21926 |
| 2031015 | Vlasi-2 | D02 | G:G | 0.4129 | 3.53584 |
| 2031015 | Vlasi-2 | E02 | G:A | 2.18934 | 2.01761 |
| 2031015 | Vlasi-2 | F02 | G:G | 0.45265 | 3.7182 |
| 2031015 | Vlasi-2 | G02 | G:A | 1.96112 | 2.15614 |
| 2031015 | Vlasi-2 | H02 | G:G | 0.45195 | 3.68706 |
| 2031015 | Vlasi-2 | A03 | G:A | 2.21525 | 2.20597 |
| 2031015 | Vlasi-2 | B03 | A:A | 3.48161 | 0.49796 |
| 2031015 | Vlasi-2 | C03 | A:A | 3.64662 | 0.5473 |
| 2031015 | Vlasi-2 | D03 | G:G | 0.40306 | 3.51958 |
| 2031015 | Vlasi-2 | E03 | G:G | 0.39223 | 3.49994 |
| 2031015 | Vlasi-2 | F03 | G:G | 0.40186 | 3.51114 |
| 2031015 | Vlasi-2 | G03 | G:G | 0.71858 | 3.31446 |
| 2031015 | Vlasi-2 | H03 | G:A | 2.12073 | 1.85223 |
| 2031015 | Vlasi-2 | A04 | A:A | 3.48743 | 0.49952 |
| 2031015 | Vlasi-2 | B04 | G:A | 2.1646 | 2.09992 |
| 2031015 | Vlasi-2 | C04 | G:G | 0.38972 | 3.53869 |
| 2031015 | Vlasi-2 | D04 | G:G | 0.39352 | 3.51557 |
| 2031015 | Vlasi-2 | E04 | ? | 0.92546 | 0.50276 |
| 2031015 | Vlasi-2 | F04 | ? | 1.05711 | 0.454 |
| 2031015 | Vlasi-2 | G04 | ? | 1.04258 | 0.40614 |
| 2031015 | Vlasi-2 | H04 | G:G | 0.41316 | 3.64118 |
| 2031015 | Vlasi-2 | A05 | G:G | 0.48156 | 3.60729 |
| 2031015 | Vlasi-2 | B05 | G:A | 2.33084 | 2.02785 |
| 2031015 | Vlasi-2 | C05 | G:G | 0.43558 | 3.69533 |
| 2031015 | Vlasi-2 | D05 | ? | 1.03594 | 0.40002 |
| 2031015 | Vlasi-2 | E05 | G:A | 2.16359 | 2.1085 |
| 2031015 | Vlasi-2 | F05 | G:A | 2.06902 | 1.95321 |
| 2031015 | Vlasi-2 | G05 | G:G | 0.47966 | 3.51622 |
| 2031015 | Vlasi-2 | H05 | G:A | 2.0706 | 1.98618 |
| 2031015 | Vlasi-2 | A06 | G:A | 1.83382 | 2.29044 |
| 2031015 | Vlasi-2 | B06 | G:A | 2.23313 | 2.01993 |
| 2031015 | Vlasi-2 | C06 | G:A | 2.15654 | 1.88333 |
| 2031015 | Vlasi-2 | D06 | G:G | 0.40208 | 3.59146 |
| 2031015 | Vlasi-2 | E06 | G:G | 0.40273 | 3.36004 |
| 2031015 | Vlasi-2 | F06 | G:G | 0.39711 | 3.52735 |
| 2031015 | Vlasi-2 | G06 | G:G | 0.43065 | 3.61382 |
| 2031015 | Vlasi-2 | H06 | G:A | 2.08433 | 2.08221 |
| 2031015 | Vlasi-2 | A07 | G:G | 0.41772 | 3.4217 |
| 2031015 | Vlasi-2 | B07 | ? | 0.96016 | 1.43059 |
| 2031015 | Vlasi-2 | C07 | G:G | 0.41451 | 3.61973 |
| 2031015 | Vlasi-2 | D07 | G:A | 2.17981 | 1.99557 |
| 2031015 | Vlasi-2 | E07 | G:G | 0.42413 | 3.7419 |

| | | | | | |
|---|---|---|---|---|---|
| 2031015 | Vlasi-2 | F07 | G:G | 0.38374 | 3.49267 |
| 2031015 | Vlasi-2 | G07 | A:A | 3.56083 | 0.48802 |
| 2031015 | Vlasi-2 | H07 | G:G | 0.44729 | 3.62547 |
| 2031015 | Vlasi-2 | A08 | G:G | 0.48089 | 3.4433 |
| 2031015 | Vlasi-2 | B08 | G:G | 0.43429 | 3.50936 |
| 2031015 | Vlasi-2 | C08 | ? | 0.99866 | 0.33784 |
| 2031015 | Vlasi-2 | D08 | G:G | 0.42627 | 3.51336 |
| 2031015 | Vlasi-2 | E08 | G:G | 0.3709 | 3.54076 |
| 2031015 | Vlasi-2 | F08 | G:G | 0.39297 | 3.70754 |
| 2031015 | Vlasi-2 | G08 | G:G | 0.43803 | 3.95089 |
| 2031015 | Vlasi-2 | H08 | G:A | 2.1776 | 2.13004 |
| 2031015 | Vlasi-2 | A09 | G:A | 2.20013 | 2.16872 |
| 2031015 | Vlasi-2 | B09 | ? | 1.04742 | 0.39463 |
| 2031015 | Vlasi-2 | C09 | ? | 1.0677 | 0.35976 |
| 2031015 | Vlasi-2 | D09 | G:G | 0.43959 | 3.49586 |
| 2031015 | Vlasi-2 | E09 | G:G | 0.53378 | 3.49983 |
| 2031015 | Vlasi-2 | F09 | G:A | 1.88983 | 1.98534 |
| 2031015 | Vlasi-2 | G09 | G:G | 0.40192 | 3.72696 |
| 2031015 | Vlasi-2 | H09 | G:G | 0.44649 | 3.85002 |
| 2031015 | Vlasi-2 | A10 | G:G | 0.4549 | 3.60883 |
| 2031015 | Vlasi-2 | B10 | G:G | 0.38976 | 3.52081 |
| 2031015 | Vlasi-2 | C10 | G:A | 2.00373 | 2.1407 |
| 2031015 | Vlasi-2 | D10 | G:G | 0.43515 | 3.68648 |
| 2031015 | Vlasi-2 | E10 | G:G | 0.40087 | 3.45936 |
| 2031015 | Vlasi-2 | F10 | G:A | 1.53817 | 1.99589 |
| 2031015 | Vlasi-2 | G10 | G:A | 1.85899 | 1.9632 |
| 2031015 | Vlasi-2 | H10 | G:A | 2.03307 | 2.18536 |
| 2031015 | Vlasi-2 | A11 | G:G | 0.45357 | 3.48383 |
| 2031015 | Vlasi-2 | B11 | G:G | 0.40237 | 3.60846 |
| 2031015 | Vlasi-2 | C11 | G:G | 0.41389 | 3.575 |
| 2031015 | Vlasi-2 | D11 | G:A | 2.18209 | 2.12049 |
| 2031015 | Vlasi-2 | E11 | G:A | 2.12114 | 2.09383 |
| 2031015 | Vlasi-2 | F11 | G:G | 0.46696 | 3.72573 |
| 2031015 | Vlasi-2 | G11 | G:A | 2.2052 | 2.23931 |
| 2031015 | Vlasi-2 | H11 | G:G | 0.41237 | 3.6635 |
| 2031015 | Vlasi-2 | A12 | G:G | 0.47533 | 3.51731 |
| 2031015 | Vlasi-2 | B12 | G:G | 0.4346 | 3.55617 |
| 2031015 | Vlasi-2 | C12 | G:G | 0.41872 | 3.76446 |
| 2031015 | Vlasi-2 | D12 | G:G | 0.40681 | 3.62509 |
| 2031015 | Vlasi-2 | E12 | G:A | 2.3442 | 2.37874 |
| 2031015 | Vlasi-2 | F12 | G:G | 0.41558 | 3.70403 |
| 2031015 | Vlasi-2 | G12 | G:A | 2.23977 | 2.21189 |
| 2031015 | Vlasi-2 | H12 | G:A | 2.27435 | 2.41608 |
| 2031015 | Vlasi-3 | A01 | G:G | 0.40473 | 3.53173 |
| 2031015 | Vlasi-3 | B01 | G:A | 2.32166 | 2.07665 |
| 2031015 | Vlasi-3 | C01 | G:G | 0.37845 | 3.60195 |
| 2031015 | Vlasi-3 | D01 | G:A | 2.18153 | 1.94014 |
| 2031015 | Vlasi-3 | E01 | A:A | 3.61643 | 0.48139 |
| 2031015 | Vlasi-3 | F01 | G:A | 2.30992 | 1.92204 |
| 2031015 | Vlasi-3 | G01 | G:G | 0.40522 | 3.70878 |

| | | | | | |
|---|---|---|---|---|---|
| 2031015 | Vlasi-3 | H01 | G:A | 2.03813 | 1.90071 |
| 2031015 | Vlasi-3 | A02 | G:G | 0.43059 | 3.60184 |
| 2031015 | Vlasi-3 | B02 | G:G | 0.43642 | 3.58044 |
| 2031015 | Vlasi-3 | C02 | G:G | 0.39771 | 3.72759 |
| 2031015 | Vlasi-3 | D02 | G:G | 0.37335 | 3.54314 |
| 2031015 | Vlasi-3 | E02 | G:A | 2.20273 | 2.02679 |
| 2031015 | Vlasi-3 | F02 | G:A | 2.09518 | 2.1897 |
| 2031015 | Vlasi-3 | G02 | G:G | 0.40996 | 2.88271 |
| 2031015 | Vlasi-3 | H02 | G:G | 0.40802 | 3.6058 |
| 2031015 | Vlasi-3 | A03 | G:G | 0.42204 | 3.48593 |
| 2031015 | Vlasi-3 | B03 | G:G | 0.43662 | 3.52745 |
| 2031015 | Vlasi-3 | C03 | G:G | 0.3835 | 3.43557 |
| 2031015 | Vlasi-3 | D03 | G:G | 0.37497 | 3.52329 |
| 2031015 | Vlasi-3 | E03 | G:G | 0.37233 | 3.44873 |
| 2031015 | Vlasi-3 | F03 | G:G | 0.47246 | 3.68085 |
| 2031015 | Vlasi-3 | G03 | ? | 1.02694 | 0.49333 |
| 2031015 | Vlasi-3 | H03 | G:G | 0.42662 | 3.69465 |
| 2031015 | Vlasi-3 | A04 | G:G | 0.41539 | 3.44495 |
| 2031015 | Vlasi-3 | B04 | G:A | 2.00742 | 2.29623 |
| 2031015 | Vlasi-3 | C04 | G:G | 0.39239 | 3.4899 |
| 2031015 | Vlasi-3 | D04 | G:A | 2.136 | 2.0066 |
| 2031015 | Vlasi-3 | E04 | G:G | 0.39485 | 3.55217 |
| 2031015 | Vlasi-3 | F04 | G:G | 0.38706 | 3.56573 |
| 2031015 | Vlasi-3 | G04 | G:G | 0.43865 | 3.76847 |
| 2031015 | Vlasi-3 | H04 | G:A | 2.25175 | 2.00842 |
| 2031015 | Vlasi-3 | A05 | G:A | 2.21949 | 2.23784 |
| 2031015 | Vlasi-3 | B05 | G:G | 0.39742 | 3.42564 |
| 2031015 | Vlasi-3 | C05 | A:A | 3.48765 | 0.52182 |
| 2031015 | Vlasi-3 | D05 | G:A | 2.14175 | 2.00658 |
| 2031015 | Vlasi-3 | E05 | G:G | 0.42498 | 3.61379 |
| 2031015 | Vlasi-3 | F05 | G:A | 2.0968 | 2.07121 |
| 2031015 | Vlasi-3 | G05 | G:A | 1.98314 | 2.26368 |
| 2031015 | Vlasi-3 | H05 | G:G | 0.46728 | 3.73816 |
| 2031015 | Vlasi-3 | A06 | G:A | 2.3127 | 2.23718 |
| 2031015 | Vlasi-3 | B06 | G:G | 0.39918 | 3.40011 |
| 2031015 | Vlasi-3 | C06 | G:G | 0.38535 | 3.51212 |
| 2031015 | Vlasi-3 | D06 | G:A | 2.12902 | 2.24397 |
| 2031015 | Vlasi-3 | E06 | G:A | 2.07411 | 2.12159 |
| 2031015 | Vlasi-3 | F06 | G:G | 0.38803 | 3.54122 |
| 2031015 | Vlasi-3 | G06 | G:G | 0.37855 | 3.48434 |
| 2031015 | Vlasi-3 | H06 | G:A | 2.09456 | 2.0399 |
| 2031015 | Vlasi-3 | A07 | G:A | 2.04794 | 2.27873 |
| 2031015 | Vlasi-3 | B07 | G:G | 0.36821 | 3.51691 |
| 2031015 | Vlasi-3 | C07 | G:A | 1.93374 | 2.11958 |
| 2031015 | Vlasi-3 | D07 | G:G | 0.368 | 3.46335 |
| 2031015 | Vlasi-3 | E07 | G:G | 0.40849 | 3.60234 |
| 2031015 | Vlasi-3 | F07 | G:A | 2.02805 | 1.95372 |
| 2031015 | Vlasi-3 | G07 | G:A | 2.0258 | 2.36973 |
| 2031015 | Vlasi-3 | H07 | G:G | 0.42308 | 3.56658 |
| 2031015 | Vlasi-3 | A08 | A:A | 3.50027 | 0.51678 |

| | | | | | |
|---|---|---|---|---|---|
| 2031015 | Vlasi-3 | B08 | G:G | 0.37059 | 3.53868 |
| 2031015 | Vlasi-3 | C08 | G:A | 2.14409 | 1.95329 |
| 2031015 | Vlasi-3 | D08 | G:A | 2.12794 | 2.15123 |
| 2031015 | Vlasi-3 | E08 | G:G | 0.36562 | 3.62738 |
| 2031015 | Vlasi-3 | F08 | G:A | 2.00887 | 2.06669 |
| 2031015 | Vlasi-3 | G08 | G:A | 1.92586 | 2.02452 |
| 2031015 | Vlasi-3 | H08 | G:A | 2.20188 | 2.03615 |
| 2031015 | Vlasi-3 | A09 | G:A | 2.16987 | 2.20071 |
| 2031015 | Vlasi-3 | B09 | G:G | 0.38158 | 3.59722 |
| 2031015 | Vlasi-3 | C09 | G:G | 0.38968 | 3.552 |
| 2031015 | Vlasi-3 | D09 | G:G | 0.37126 | 3.4108 |
| 2031015 | Vlasi-3 | E09 | G:G | 0.37809 | 3.66964 |
| 2031015 | Vlasi-3 | F09 | G:G | 0.39212 | 3.50245 |
| 2031015 | Vlasi-3 | G09 | G:A | 2.00722 | 2.25511 |
| 2031015 | Vlasi-3 | H09 | G:G | 0.41602 | 3.49719 |
| 2031015 | Vlasi-3 | A10 | G:G | 0.43964 | 3.55009 |
| 2031015 | Vlasi-3 | B10 | G:G | 0.38515 | 3.53692 |
| 2031015 | Vlasi-3 | C10 | G:A | 2.04683 | 2.21407 |
| 2031015 | Vlasi-3 | D10 | G:G | 0.38668 | 3.58886 |
| 2031015 | Vlasi-3 | E10 | G:G | 0.40041 | 3.81798 |
| 2031015 | Vlasi-3 | F10 | G:G | 0.3849 | 3.63687 |
| 2031015 | Vlasi-3 | G10 | G:G | 0.40619 | 3.69088 |
| 2031015 | Vlasi-3 | H10 | G:G | 0.45623 | 3.75917 |
| 2031015 | Vlasi-3 | A11 | G:A | 2.0288 | 2.26466 |
| 2031015 | Vlasi-3 | B11 | G:G | 0.37616 | 3.59314 |
| 2031015 | Vlasi-3 | C11 | A:A | 3.49325 | 0.49062 |
| 2031015 | Vlasi-3 | D11 | G:A | 2.06967 | 2.16605 |
| 2031015 | Vlasi-3 | E11 | G:G | 0.41019 | 3.6593 |
| 2031015 | Vlasi-3 | F11 | G:G | 0.39729 | 3.79257 |
| 2031015 | Vlasi-3 | G11 | A:A | 3.6231 | 0.50822 |
| 2031015 | Vlasi-3 | H11 | G:G | 0.42537 | 3.48945 |
| 2031015 | Vlasi-3 | A12 | G:A | 2.06849 | 2.12783 |
| 2031015 | Vlasi-3 | B12 | G:A | 2.15424 | 2.32043 |
| 2031015 | Vlasi-3 | C12 | G:G | 0.37776 | 3.51542 |
| 2031015 | Vlasi-3 | D12 | G:A | 2.18302 | 2.15623 |
| 2031015 | Vlasi-3 | E12 | G:G | 0.39949 | 3.75055 |
| 2031015 | Vlasi-3 | F12 | A:A | 3.76864 | 0.53754 |
| 2031015 | Vlasi-3 | G12 | G:G | 0.39838 | 3.60685 |
| 2031015 | Vlasi-3 | H12 | G:A | 2.41015 | 2.12491 |
| 2031015 | Vlasi-4 | A01 | G:G | 0.40494 | 3.61603 |
| 2031015 | Vlasi-4 | B01 | G:G | 0.38494 | 3.54354 |
| 2031015 | Vlasi-4 | C01 | G:A | 2.081 | 2.03128 |
| 2031015 | Vlasi-4 | D01 | G:G | 0.37398 | 3.63673 |
| 2031015 | Vlasi-4 | E01 | G:A | 2.03565 | 2.26698 |
| 2031015 | Vlasi-4 | F01 | A:A | 3.59595 | 0.49031 |
| 2031015 | Vlasi-4 | G01 | A:A | 3.50184 | 0.50093 |
| 2031015 | Vlasi-4 | H01 | ? | 0.71072 | 0.51824 |
| 2031015 | Vlasi-4 | A02 | G:A | 2.25825 | 2.18568 |
| 2031015 | Vlasi-4 | B02 | G:G | 0.40954 | 3.56937 |
| 2031015 | Vlasi-4 | C02 | G:G | 0.42552 | 3.74616 |

| | | | | | |
|---|---|---|---|---|---|
| 2031015 | Vlasi-4 | D02 | G:A | 2.16004 | 2.05422 |
| 2031015 | Vlasi-4 | E02 | G:G | 0.38881 | 3.57072 |
| 2031015 | Vlasi-4 | F02 | G:A | 2.15049 | 2.15723 |
| 2031015 | Vlasi-4 | G02 | G:G | 0.41195 | 3.48908 |
| 2031015 | Vlasi-4 | H02 | G:A | 2.23202 | 2.09929 |
| 2031015 | Vlasi-4 | A03 | G:G | 0.42234 | 3.47026 |
| 2031015 | Vlasi-4 | B03 | G:A | 2.19505 | 2.04494 |
| 2031015 | Vlasi-4 | C03 | A:A | 3.58818 | 0.50199 |
| 2031015 | Vlasi-4 | D03 | G:A | 2.02134 | 2.16012 |
| 2031015 | Vlasi-4 | E03 | G:G | 0.37823 | 3.53327 |
| 2031015 | Vlasi-4 | F03 | G:G | 0.38465 | 3.49562 |
| 2031015 | Vlasi-4 | G03 | G:G | 0.39815 | 3.60169 |
| 2031015 | Vlasi-4 | H03 | G:G | 0.41112 | 3.51897 |
| 2031015 | Vlasi-4 | A04 | G:G | 0.42595 | 3.50514 |
| 2031015 | Vlasi-4 | B04 | G:A | 2.20078 | 2.01619 |
| 2031015 | Vlasi-4 | C04 | G:G | 0.38995 | 3.60377 |
| 2031015 | Vlasi-4 | D04 | G:A | 1.99913 | 1.98889 |
| 2031015 | Vlasi-4 | E04 | G:A | 1.91046 | 1.98406 |
| 2031015 | Vlasi-4 | F04 | G:G | 0.37747 | 3.49067 |
| 2031015 | Vlasi-4 | G04 | G:G | 0.40554 | 3.54886 |
| 2031015 | Vlasi-4 | H04 | G:A | 2.02994 | 2.07091 |
| 2031015 | Vlasi-4 | A05 | G:A | 2.12733 | 2.23813 |
| 2031015 | Vlasi-4 | B05 | G:G | 0.3547 | 3.46315 |
| 2031015 | Vlasi-4 | C05 | G:A | 2.05643 | 2.04839 |
| 2031015 | Vlasi-4 | D05 | G:G | 0.3884 | 3.60029 |
| 2031015 | Vlasi-4 | E05 | G:G | 0.38155 | 3.6478 |
| 2031015 | Vlasi-4 | F05 | G:G | 0.38268 | 3.62127 |
| 2031015 | Vlasi-4 | G05 | G:A | 2.03357 | 2.05085 |
| 2031015 | Vlasi-4 | H05 | G:G | 0.42014 | 3.55096 |
| 2031015 | Vlasi-4 | A06 | G:A | 2.02444 | 2.02925 |
| 2031015 | Vlasi-4 | B06 | G:G | 0.39742 | 3.54221 |
| 2031015 | Vlasi-4 | C06 | G:A | 2.08691 | 2.10914 |
| 2031015 | Vlasi-4 | D06 | G:A | 2.08364 | 1.87967 |
| 2031015 | Vlasi-4 | E06 | G:A | 1.85779 | 2.22208 |
| 2031015 | Vlasi-4 | F06 | G:A | 2.01546 | 2.19871 |
| 2031015 | Vlasi-4 | G06 | G:G | 0.45359 | 3.65739 |
| 2031015 | Vlasi-4 | H06 | G:G | 0.42591 | 3.60295 |
| 2031015 | Vlasi-4 | A07 | A:A | 3.23592 | 0.59075 |
| 2031015 | Vlasi-4 | B07 | ? | 0.57412 | 0.74185 |
| 2031015 | Vlasi-4 | C07 | ? | 0.59649 | 0.55855 |
| 2031015 | Vlasi-4 | D07 | ? | 0.62289 | 0.61023 |
| 2031015 | Vlasi-4 | E07 | ? | 0.62639 | 0.60445 |
| 2031015 | Vlasi-4 | F07 | A:A | 3.53049 | 0.5873 |
| 2031015 | Vlasi-4 | G07 | A:A | 3.52799 | 0.54093 |
| 2031015 | Vlasi-4 | H07 | A:A | 3.5364 | 0.63268 |
| 2031015 | Vlasi-4 | A08 | A:A | 3.62539 | 0.64268 |
| 2031015 | Vlasi-4 | B08 | A:A | 3.57242 | 0.59253 |
| 2031015 | Vlasi-4 | C08 | A:A | 3.57525 | 0.53306 |
| 2031015 | Vlasi-4 | D08 | A:A | 3.64438 | 0.59786 |
| 2031015 | Vlasi-4 | E08 | A:A | 3.54865 | 0.60015 |

| | | | | | |
|---|---|---|---|---|---|
| 2031015 | Vlasi-4 | F08 | A:A | 3.60697 | 0.58399 |
| 2031015 | Vlasi-4 | G08 | A:A | 3.66393 | 0.62611 |
| 2031015 | Vlasi-4 | H08 | A:A | 3.50274 | 0.61076 |
| 2031015 | Vlasi-4 | A09 | A:A | 3.46444 | 0.61265 |
| 2031015 | Vlasi-4 | B09 | A:A | 3.53525 | 0.56752 |
| 2031015 | Vlasi-4 | C09 | A:A | 3.50417 | 0.61373 |
| 2031015 | Vlasi-4 | D09 | A:A | 3.42544 | 0.56576 |
| 2031015 | Vlasi-4 | E09 | A:A | 3.58347 | 0.59417 |
| 2031015 | Vlasi-4 | F09 | A:A | 3.5478 | 0.59951 |
| 2031015 | Vlasi-4 | G09 | A:A | 3.56053 | 0.60924 |
| 2031015 | Vlasi-4 | H09 | A:A | 3.54248 | 0.59176 |
| 2031015 | Vlasi-4 | A10 | A:A | 3.43664 | 0.61962 |
| 2031015 | Vlasi-4 | B10 | A:A | 3.55028 | 0.56777 |
| 2031015 | Vlasi-4 | C10 | G:G | 0.3812 | 3.61074 |
| 2031015 | Vlasi-4 | D10 | G:G | 0.3659 | 3.54116 |
| 2031015 | Vlasi-4 | E10 | G:G | 0.39736 | 3.61761 |
| 2031015 | Vlasi-4 | F10 | G:G | 0.3989 | 3.51144 |
| 2031015 | Vlasi-4 | G10 | G:G | 0.47175 | 3.49705 |
| 2031015 | Vlasi-4 | H10 | G:G | 0.40035 | 3.51435 |
| 2031015 | Vlasi-4 | A11 | G:G | 0.47355 | 3.65599 |
| 2031015 | Vlasi-4 | B11 | G:G | 0.41609 | 3.42673 |
| 2031015 | Vlasi-4 | C11 | G:G | 0.43164 | 3.58236 |
| 2031015 | Vlasi-4 | D11 | G:G | 0.40272 | 3.70776 |
| 2031015 | Vlasi-4 | E11 | G:G | 0.40632 | 3.68785 |
| 2031015 | Vlasi-4 | F11 | G:G | 0.41663 | 3.74914 |
| 2031015 | Vlasi-4 | G11 | G:G | 0.41143 | 3.57326 |
| 2031015 | Vlasi-4 | H11 | G:G | 0.48192 | 3.48399 |
| 2031015 | Vlasi-4 | A12 | G:G | 0.52525 | 3.54863 |
| 2031015 | Vlasi-4 | B12 | G:G | 0.51527 | 3.45342 |
| 2031015 | Vlasi-4 | C12 | G:G | 0.42429 | 3.5288 |
| 2031015 | Vlasi-4 | D12 | G:G | 0.4541 | 3.44986 |
| 2031015 | Vlasi-4 | E12 | G:G | 0.43421 | 3.46239 |
| 2031015 | Vlasi-4 | F12 | G:G | 0.4658 | 3.7638 |
| 2031015 | Vlasi-4 | G12 | G:G | 0.49395 | 3.42137 |
| 2031015 | Vlasi-4 | H12 | NTC | 0.69408 | 0.78333 |
| 2031015 | Vlasi-1 | A01 | C:C | 0.44214 | 3.658 |
| 2031015 | Vlasi-1 | B01 | C:C | 0.38524 | 3.64724 |
| 2031015 | Vlasi-1 | C01 | C:T | 1.73483 | 2.73323 |
| 2031015 | Vlasi-1 | D01 | C:C | 0.39568 | 3.69872 |
| 2031015 | Vlasi-1 | E01 | C:C | 0.38429 | 3.78972 |
| 2031015 | Vlasi-1 | F01 | C:C | 0.39187 | 3.89136 |
| 2031015 | Vlasi-1 | G01 | C:T | 1.29859 | 2.37422 |
| 2031015 | Vlasi-1 | H01 | C:T | 1.76078 | 2.59769 |
| 2031015 | Vlasi-1 | A02 | C:C | 0.46303 | 3.53879 |
| 2031015 | Vlasi-1 | B02 | C:C | 0.55223 | 3.12908 |
| 2031015 | Vlasi-1 | C02 | C:T | 1.69266 | 2.40862 |
| 2031015 | Vlasi-1 | D02 | C:T | 1.68134 | 2.4039 |
| 2031015 | Vlasi-1 | E02 | C:C | 0.48324 | 3.36361 |
| 2031015 | Vlasi-1 | F02 | C:C | 0.42529 | 3.40066 |
| 2031015 | Vlasi-1 | G02 | C:C | 0.43499 | 3.60929 |

| | | | | | |
|---|---|---|---|---|---|
| 2031015 | Vlasi-1 | H02 | C:T | 1.56084 | 2.51005 |
| 2031015 | Vlasi-1 | A03 | C:T | 1.78247 | 2.66565 |
| 2031015 | Vlasi-1 | B03 | C:C | 0.43829 | 3.53012 |
| 2031015 | Vlasi-1 | C03 | C:C | 0.4936 | 3.55887 |
| 2031015 | Vlasi-1 | D03 | C:T | 1.60618 | 2.48874 |
| 2031015 | Vlasi-1 | E03 | T:T | 3.31898 | 0.53546 |
| 2031015 | Vlasi-1 | F03 | C:C | 0.44134 | 3.55361 |
| 2031015 | Vlasi-1 | G03 | C:C | 0.4323 | 3.4488 |
| 2031015 | Vlasi-1 | H03 | C:C | 0.43345 | 3.44658 |
| 2031015 | Vlasi-1 | A04 | C:C | 0.4719 | 3.48796 |
| 2031015 | Vlasi-1 | B04 | T:T | 3.22408 | 0.55044 |
| 2031015 | Vlasi-1 | C04 | C:T | 1.60953 | 2.53372 |
| 2031015 | Vlasi-1 | D04 | C:C | 0.41498 | 3.51618 |
| 2031015 | Vlasi-1 | E04 | C:C | 0.47575 | 3.48993 |
| 2031015 | Vlasi-1 | F04 | C:C | 0.44897 | 3.40636 |
| 2031015 | Vlasi-1 | G04 | C:T | 1.51967 | 2.6417 |
| 2031015 | Vlasi-1 | H04 | C:C | 0.42583 | 3.53419 |
| 2031015 | Vlasi-1 | A05 | C:C | 0.49879 | 3.62364 |
| 2031015 | Vlasi-1 | B05 | ? | 0.89869 | 1.70044 |
| 2031015 | Vlasi-1 | C05 | C:T | 1.48768 | 2.62899 |
| 2031015 | Vlasi-1 | D05 | C:C | 0.44054 | 3.58222 |
| 2031015 | Vlasi-1 | E05 | C:C | 0.43251 | 3.41288 |
| 2031015 | Vlasi-1 | F05 | ? | 0.60579 | 1.88682 |
| 2031015 | Vlasi-1 | G05 | C:T | 1.41262 | 2.26568 |
| 2031015 | Vlasi-1 | H05 | C:C | 0.43097 | 3.49533 |
| 2031015 | Vlasi-1 | A06 | C:C | 0.48078 | 3.61079 |
| 2031015 | Vlasi-1 | B06 | C:C | 0.43746 | 3.61691 |
| 2031015 | Vlasi-1 | C06 | C:C | 0.47096 | 3.54735 |
| 2031015 | Vlasi-1 | D06 | C:C | 0.4473 | 3.43444 |
| 2031015 | Vlasi-1 | E06 | C:C | 0.46052 | 3.59211 |
| 2031015 | Vlasi-1 | F06 | C:C | 0.43431 | 3.32777 |
| 2031015 | Vlasi-1 | G06 | C:C | 0.46317 | 3.3598 |
| 2031015 | Vlasi-1 | H06 | C:C | 0.43935 | 3.64001 |
| 2031015 | Vlasi-1 | A07 | C:T | 1.70477 | 2.73137 |
| 2031015 | Vlasi-1 | B07 | C:C | 0.48022 | 3.24474 |
| 2031015 | Vlasi-1 | C07 | C:T | 1.57595 | 2.26359 |
| 2031015 | Vlasi-1 | D07 | C:C | 0.46915 | 3.44122 |
| 2031015 | Vlasi-1 | E07 | C:C | 0.42537 | 3.34294 |
| 2031015 | Vlasi-1 | F07 | T:T | 3.15446 | 0.55914 |
| 2031015 | Vlasi-1 | G07 | C:C | 0.44452 | 3.47119 |
| 2031015 | Vlasi-1 | H07 | C:C | 0.46378 | 3.45205 |
| 2031015 | Vlasi-1 | A08 | C:T | 1.87153 | 2.56246 |
| 2031015 | Vlasi-1 | B08 | ? | 0.84009 | 0.55372 |
| 2031015 | Vlasi-1 | C08 | C:C | 0.50878 | 3.5371 |
| 2031015 | Vlasi-1 | D08 | C:C | 0.44212 | 3.70126 |
| 2031015 | Vlasi-1 | E08 | C:C | 0.41978 | 3.62052 |
| 2031015 | Vlasi-1 | F08 | C:C | 0.42719 | 3.53609 |
| 2031015 | Vlasi-1 | G08 | C:C | 0.49676 | 3.65145 |
| 2031015 | Vlasi-1 | H08 | C:C | 0.43777 | 3.51538 |
| 2031015 | Vlasi-1 | A09 | C:C | 0.47304 | 3.51395 |

| | | | | | |
|---|---|---|---|---|---|
| 2031015 | Vlasi-1 | B09 | C:C | 0.4524 | 3.55098 |
| 2031015 | Vlasi-1 | C09 | C:T | 1.38809 | 2.12868 |
| 2031015 | Vlasi-1 | D09 | ? | 0.80135 | 0.53867 |
| 2031015 | Vlasi-1 | E09 | C:C | 0.44489 | 3.71706 |
| 2031015 | Vlasi-1 | F09 | C:T | 1.5244 | 2.2634 |
| 2031015 | Vlasi-1 | G09 | T:T | 3.43441 | 0.56894 |
| 2031015 | Vlasi-1 | H09 | C:T | 1.78876 | 2.58796 |
| 2031015 | Vlasi-1 | A10 | C:C | 0.48118 | 3.69736 |
| 2031015 | Vlasi-1 | B10 | C:C | 0.42598 | 3.46725 |
| 2031015 | Vlasi-1 | C10 | C:C | 0.409 | 3.56981 |
| 2031015 | Vlasi-1 | D10 | C:T | 1.54332 | 2.63551 |
| 2031015 | Vlasi-1 | E10 | C:C | 0.41888 | 2.92135 |
| 2031015 | Vlasi-1 | F10 | C:C | 0.42867 | 3.75237 |
| 2031015 | Vlasi-1 | G10 | C:C | 0.44191 | 3.59734 |
| 2031015 | Vlasi-1 | H10 | C:T | 1.48456 | 2.66556 |
| 2031015 | Vlasi-1 | A11 | T:T | 3.40284 | 0.60927 |
| 2031015 | Vlasi-1 | B11 | C:C | 0.41346 | 3.53824 |
| 2031015 | Vlasi-1 | C11 | C:T | 1.6492 | 2.49136 |
| 2031015 | Vlasi-1 | D11 | C:T | 1.68593 | 2.49167 |
| 2031015 | Vlasi-1 | E11 | C:T | 1.67603 | 2.35631 |
| 2031015 | Vlasi-1 | F11 | C:C | 0.45512 | 3.68311 |
| 2031015 | Vlasi-1 | G11 | C:T | 1.71277 | 2.66545 |
| 2031015 | Vlasi-1 | H11 | C:C | 0.44381 | 3.69344 |
| 2031015 | Vlasi-1 | A12 | C:C | 0.45577 | 3.49992 |
| 2031015 | Vlasi-1 | B12 | C:C | 0.42706 | 3.49155 |
| 2031015 | Vlasi-1 | C12 | C:C | 0.41619 | 3.45832 |
| 2031015 | Vlasi-1 | D12 | C:C | 0.41078 | 3.51764 |
| 2031015 | Vlasi-1 | E12 | C:C | 0.41815 | 3.66516 |
| 2031015 | Vlasi-1 | F12 | C:C | 0.42802 | 3.46019 |
| 2031015 | Vlasi-1 | G12 | C:T | 1.64613 | 2.77198 |
| 2031015 | Vlasi-1 | H12 | C:T | 1.54384 | 2.65596 |
| 2031015 | Vlasi-2 | A01 | C:C | 0.47309 | 3.61163 |
| 2031015 | Vlasi-2 | B01 | C:C | 0.44761 | 3.56281 |
| 2031015 | Vlasi-2 | C01 | C:C | 0.4313 | 3.65259 |
| 2031015 | Vlasi-2 | D01 | ? | 0.70747 | 0.51111 |
| 2031015 | Vlasi-2 | E01 | C:C | 0.46323 | 3.37267 |
| 2031015 | Vlasi-2 | F01 | ? | 0.81402 | 0.65106 |
| 2031015 | Vlasi-2 | G01 | ? | 0.76485 | 0.59612 |
| 2031015 | Vlasi-2 | H01 | C:T | 1.56681 | 2.03149 |
| 2031015 | Vlasi-2 | A02 | C:C | 0.47122 | 3.70046 |
| 2031015 | Vlasi-2 | B02 | ? | 0.7737 | 0.8219 |
| 2031015 | Vlasi-2 | C02 | T:T | 3.50236 | 0.58291 |
| 2031015 | Vlasi-2 | D02 | C:C | 0.44689 | 3.68995 |
| 2031015 | Vlasi-2 | E02 | C:C | 0.42122 | 3.55645 |
| 2031015 | Vlasi-2 | F02 | C:C | 0.43564 | 3.35105 |
| 2031015 | Vlasi-2 | G02 | C:C | 0.45514 | 3.56808 |
| 2031015 | Vlasi-2 | H02 | C:T | 1.32633 | 2.18307 |
| 2031015 | Vlasi-2 | A03 | C:T | 1.92308 | 2.87119 |
| 2031015 | Vlasi-2 | B03 | C:T | 1.75223 | 2.56119 |
| 2031015 | Vlasi-2 | C03 | T:T | 3.3812 | 0.57982 |

| | | | | | |
|---|---|---|---|---|---|
| 2031015 | Vlasi-2 | D03 | C:C | 0.4476 | 3.63378 |
| 2031015 | Vlasi-2 | E03 | C:C | 0.42337 | 3.479 |
| 2031015 | Vlasi-2 | F03 | C:C | 0.43441 | 3.46018 |
| 2031015 | Vlasi-2 | G03 | ? | 0.71115 | 0.51015 |
| 2031015 | Vlasi-2 | H03 | C:C | 0.44103 | 3.39279 |
| 2031015 | Vlasi-2 | A04 | C:C | 0.48815 | 3.56298 |
| 2031015 | Vlasi-2 | B04 | C:T | 1.54516 | 2.27031 |
| 2031015 | Vlasi-2 | C04 | C:C | 0.41403 | 3.52636 |
| 2031015 | Vlasi-2 | D04 | C:T | 1.56667 | 2.40558 |
| 2031015 | Vlasi-2 | E04 | ? | 0.94757 | 0.50294 |
| 2031015 | Vlasi-2 | F04 | ? | 0.84967 | 0.55086 |
| 2031015 | Vlasi-2 | G04 | ? | 0.98225 | 0.59898 |
| 2031015 | Vlasi-2 | H04 | C:C | 0.45294 | 3.60319 |
| 2031015 | Vlasi-2 | A05 | C:T | 1.74415 | 2.6196 |
| 2031015 | Vlasi-2 | B05 | C:C | 0.41993 | 3.46867 |
| 2031015 | Vlasi-2 | C05 | ? | 0.51748 | 2.60162 |
| 2031015 | Vlasi-2 | D05 | C:C | 0.44037 | 3.61415 |
| 2031015 | Vlasi-2 | E05 | ? | 1.0592 | 1.56927 |
| 2031015 | Vlasi-2 | F05 | C:T | 1.29026 | 2.00811 |
| 2031015 | Vlasi-2 | G05 | C:T | 1.41751 | 2.11211 |
| 2031015 | Vlasi-2 | H05 | C:C | 0.51241 | 3.47182 |
| 2031015 | Vlasi-2 | A06 | C:C | 0.4844 | 3.58913 |
| 2031015 | Vlasi-2 | B06 | C:T | 1.6327 | 2.36189 |
| 2031015 | Vlasi-2 | C06 | C:T | 1.32077 | 1.90221 |
| 2031015 | Vlasi-2 | D06 | C:C | 0.45024 | 3.48844 |
| 2031015 | Vlasi-2 | E06 | C:C | 0.45342 | 3.6184 |
| 2031015 | Vlasi-2 | F06 | C:C | 0.46326 | 3.76632 |
| 2031015 | Vlasi-2 | G06 | C:C | 0.45711 | 3.55228 |
| 2031015 | Vlasi-2 | H06 | C:C | 0.44828 | 3.61617 |
| 2031015 | Vlasi-2 | A07 | C:T | 1.77634 | 2.59762 |
| 2031015 | Vlasi-2 | B07 | ? | 0.86965 | 0.50417 |
| 2031015 | Vlasi-2 | C07 | C:C | 0.49237 | 3.25929 |
| 2031015 | Vlasi-2 | D07 | C:C | 0.50312 | 3.50796 |
| 2031015 | Vlasi-2 | E07 | ? | 1.02206 | 0.53636 |
| 2031015 | Vlasi-2 | F07 | C:C | 0.42849 | 3.56365 |
| 2031015 | Vlasi-2 | G07 | C:C | 0.46327 | 3.44223 |
| 2031015 | Vlasi-2 | H07 | C:C | 0.46678 | 3.49956 |
| 2031015 | Vlasi-2 | A08 | C:C | 0.50048 | 3.49961 |
| 2031015 | Vlasi-2 | B08 | C:C | 0.45469 | 3.33173 |
| 2031015 | Vlasi-2 | C08 | ? | 0.92131 | 0.46573 |
| 2031015 | Vlasi-2 | D08 | C:T | 1.54106 | 2.41829 |
| 2031015 | Vlasi-2 | E08 | C:T | 1.75775 | 2.4971 |
| 2031015 | Vlasi-2 | F08 | C:C | 0.44012 | 3.36655 |
| 2031015 | Vlasi-2 | G08 | C:C | 0.42074 | 3.54235 |
| 2031015 | Vlasi-2 | H08 | C:C | 0.44564 | 3.43454 |
| 2031015 | Vlasi-2 | A09 | C:C | 0.44785 | 3.5221 |
| 2031015 | Vlasi-2 | B09 | ? | 0.84938 | 0.51098 |
| 2031015 | Vlasi-2 | C09 | ? | 0.896 | 0.49482 |
| 2031015 | Vlasi-2 | D09 | C:C | 0.51222 | 3.21766 |
| 2031015 | Vlasi-2 | E09 | ? | 0.59137 | 2.54243 |

| | | | | | |
|---|---|---|---|---|---|
| 2031015 | Vlasi-2 | F09 | ? | 0.48601 | 2.46138 |
| 2031015 | Vlasi-2 | G09 | C:C | 0.44214 | 3.4499 |
| 2031015 | Vlasi-2 | H09 | C:T | 1.55591 | 2.56759 |
| 2031015 | Vlasi-2 | A10 | ? | 0.60431 | 2.04544 |
| 2031015 | Vlasi-2 | B10 | C:C | 0.43507 | 3.53515 |
| 2031015 | Vlasi-2 | C10 | ? | 0.6208 | 1.57513 |
| 2031015 | Vlasi-2 | D10 | C:C | 0.47896 | 3.70494 |
| 2031015 | Vlasi-2 | E10 | C:C | 0.4982 | 3.29636 |
| 2031015 | Vlasi-2 | F10 | C:C | 0.48296 | 3.34912 |
| 2031015 | Vlasi-2 | G10 | ? | 0.72538 | 0.7712 |
| 2031015 | Vlasi-2 | H10 | C:C | 0.5155 | 3.39332 |
| 2031015 | Vlasi-2 | A11 | C:C | 0.45423 | 3.45474 |
| 2031015 | Vlasi-2 | B11 | C:C | 0.43536 | 3.52256 |
| 2031015 | Vlasi-2 | C11 | C:C | 0.46039 | 3.47253 |
| 2031015 | Vlasi-2 | D11 | C:C | 0.41073 | 3.52039 |
| 2031015 | Vlasi-2 | E11 | C:C | 0.43691 | 3.4158 |
| 2031015 | Vlasi-2 | F11 | C:C | 0.4702 | 3.46693 |
| 2031015 | Vlasi-2 | G11 | C:T | 1.52263 | 2.76232 |
| 2031015 | Vlasi-2 | H11 | C:C | 0.47387 | 3.67095 |
| 2031015 | Vlasi-2 | A12 | C:C | 0.48256 | 3.68577 |
| 2031015 | Vlasi-2 | B12 | C:C | 0.4531 | 3.58972 |
| 2031015 | Vlasi-2 | C12 | C:T | 1.82214 | 2.82233 |
| 2031015 | Vlasi-2 | D12 | C:C | 0.45128 | 3.77574 |
| 2031015 | Vlasi-2 | E12 | C:C | 0.45446 | 3.44118 |
| 2031015 | Vlasi-2 | F12 | C:C | 0.45958 | 3.81898 |
| 2031015 | Vlasi-2 | G12 | C:T | 1.70906 | 2.57598 |
| 2031015 | Vlasi-2 | H12 | C:C | 0.50666 | 3.65761 |
| 2031015 | Vlasi-3 | A01 | C:C | 0.40383 | 3.64 |
| 2031015 | Vlasi-3 | B01 | C:C | 0.36981 | 3.6129 |
| 2031015 | Vlasi-3 | C01 | C:C | 0.37217 | 3.66989 |
| 2031015 | Vlasi-3 | D01 | C:C | 0.35636 | 3.65936 |
| 2031015 | Vlasi-3 | E01 | C:C | 0.38948 | 3.69653 |
| 2031015 | Vlasi-3 | F01 | C:C | 0.39951 | 3.73164 |
| 2031015 | Vlasi-3 | G01 | C:T | 1.85183 | 2.62524 |
| 2031015 | Vlasi-3 | H01 | C:C | 0.42889 | 3.71716 |
| 2031015 | Vlasi-3 | A02 | C:T | 1.86861 | 2.60114 |
| 2031015 | Vlasi-3 | B02 | C:C | 0.4224 | 3.7058 |
| 2031015 | Vlasi-3 | C02 | C:C | 0.39487 | 3.41328 |
| 2031015 | Vlasi-3 | D02 | C:C | 0.41206 | 3.55552 |
| 2031015 | Vlasi-3 | E02 | C:C | 0.40388 | 3.51367 |
| 2031015 | Vlasi-3 | F02 | T:T | 3.3253 | 0.5663 |
| 2031015 | Vlasi-3 | G02 | C:C | 0.41555 | 3.54139 |
| 2031015 | Vlasi-3 | H02 | C:C | 0.47916 | 3.7343 |
| 2031015 | Vlasi-3 | A03 | C:C | 0.45443 | 3.63816 |
| 2031015 | Vlasi-3 | B03 | C:C | 0.41956 | 3.52889 |
| 2031015 | Vlasi-3 | C03 | C:C | 0.40369 | 3.55739 |
| 2031015 | Vlasi-3 | D03 | C:C | 0.41162 | 3.36711 |
| 2031015 | Vlasi-3 | E03 | C:C | 0.436 | 3.67187 |
| 2031015 | Vlasi-3 | F03 | C:C | 0.47146 | 3.43195 |
| 2031015 | Vlasi-3 | G03 | ? | 0.72875 | 0.55287 |

| | | | | | |
|---|---|---|---|---|---|
| 2031015 | Vlasi-3 | H03 | C:T | 1.8817 | 2.72491 |
| 2031015 | Vlasi-3 | A04 | C:C | 0.42518 | 3.42116 |
| 2031015 | Vlasi-3 | B04 | T:T | 3.17545 | 0.55403 |
| 2031015 | Vlasi-3 | C04 | C:C | 0.43987 | 3.54784 |
| 2031015 | Vlasi-3 | D04 | C:C | 0.44626 | 3.67486 |
| 2031015 | Vlasi-3 | E04 | C:T | 1.50754 | 2.53909 |
| 2031015 | Vlasi-3 | F04 | C:C | 0.43665 | 3.44651 |
| 2031015 | Vlasi-3 | G04 | C:C | 0.47341 | 3.39413 |
| 2031015 | Vlasi-3 | H04 | C:T | 1.28725 | 2.39235 |
| 2031015 | Vlasi-3 | A05 | C:C | 0.45426 | 3.61301 |
| 2031015 | Vlasi-3 | B05 | C:C | 0.41458 | 3.49818 |
| 2031015 | Vlasi-3 | C05 | C:T | 1.60597 | 2.67007 |
| 2031015 | Vlasi-3 | D05 | C:T | 1.21262 | 2.07558 |
| 2031015 | Vlasi-3 | E05 | C:C | 0.46001 | 3.59202 |
| 2031015 | Vlasi-3 | F05 | T:T | 3.18253 | 0.54791 |
| 2031015 | Vlasi-3 | G05 | C:C | 0.44264 | 3.58887 |
| 2031015 | Vlasi-3 | H05 | C:T | 1.81458 | 2.6625 |
| 2031015 | Vlasi-3 | A06 | C:C | 0.41962 | 3.47518 |
| 2031015 | Vlasi-3 | B06 | C:C | 0.41892 | 3.38504 |
| 2031015 | Vlasi-3 | C06 | C:T | 1.53592 | 2.51299 |
| 2031015 | Vlasi-3 | D06 | C:T | 1.51959 | 2.42707 |
| 2031015 | Vlasi-3 | E06 | C:C | 0.43139 | 3.56817 |
| 2031015 | Vlasi-3 | F06 | C:C | 0.43819 | 3.46663 |
| 2031015 | Vlasi-3 | G06 | C:T | 1.51314 | 2.47215 |
| 2031015 | Vlasi-3 | H06 | C:C | 0.49797 | 3.24537 |
| 2031015 | Vlasi-3 | A07 | C:C | 0.44312 | 3.44561 |
| 2031015 | Vlasi-3 | B07 | C:T | 1.65868 | 2.44043 |
| 2031015 | Vlasi-3 | C07 | C:T | 1.3031 | 1.90283 |
| 2031015 | Vlasi-3 | D07 | C:C | 0.42109 | 3.67428 |
| 2031015 | Vlasi-3 | E07 | C:C | 0.42778 | 3.40238 |
| 2031015 | Vlasi-3 | F07 | C:C | 0.43313 | 3.50145 |
| 2031015 | Vlasi-3 | G07 | C:T | 1.70914 | 2.4883 |
| 2031015 | Vlasi-3 | H07 | T:T | 3.51717 | 0.78162 |
| 2031015 | Vlasi-3 | A08 | C:T | 1.81207 | 2.59457 |
| 2031015 | Vlasi-3 | B08 | C:C | 0.40195 | 3.41243 |
| 2031015 | Vlasi-3 | C08 | C:C | 0.41867 | 3.48664 |
| 2031015 | Vlasi-3 | D08 | C:T | 1.63585 | 2.32138 |
| 2031015 | Vlasi-3 | E08 | C:C | 0.4148 | 3.40763 |
| 2031015 | Vlasi-3 | F08 | C:T | 1.39453 | 2.28718 |
| 2031015 | Vlasi-3 | G08 | C:T | 1.59301 | 2.58316 |
| 2031015 | Vlasi-3 | H08 | C:C | 0.48416 | 3.5669 |
| 2031015 | Vlasi-3 | A09 | C:C | 0.42562 | 3.39843 |
| 2031015 | Vlasi-3 | B09 | C:C | 0.44171 | 3.72527 |
| 2031015 | Vlasi-3 | C09 | C:C | 0.42663 | 3.49754 |
| 2031015 | Vlasi-3 | D09 | C:C | 0.42961 | 3.62392 |
| 2031015 | Vlasi-3 | E09 | C:C | 0.4386 | 3.63351 |
| 2031015 | Vlasi-3 | F09 | C:C | 0.42067 | 3.49594 |
| 2031015 | Vlasi-3 | G09 | C:T | 1.67749 | 2.43028 |
| 2031015 | Vlasi-3 | H09 | C:C | 0.49736 | 3.7009 |
| 2031015 | Vlasi-3 | A10 | C:T | 1.66306 | 2.59546 |

| | | | | | |
|---|---|---|---|---|---|
| 2031015 | Vlasi-3 | B10 | C:T | 1.56203 | 2.71615 |
| 2031015 | Vlasi-3 | C10 | C:C | 0.42077 | 3.5624 |
| 2031015 | Vlasi-3 | D10 | C:T | 1.66751 | 2.57307 |
| 2031015 | Vlasi-3 | E10 | C:C | 0.41067 | 3.56657 |
| 2031015 | Vlasi-3 | F10 | C:T | 1.62453 | 2.37216 |
| 2031015 | Vlasi-3 | G10 | C:C | 0.45408 | 3.65883 |
| 2031015 | Vlasi-3 | H10 | C:C | 0.50091 | 3.86745 |
| 2031015 | Vlasi-3 | A11 | C:T | 1.74448 | 2.83937 |
| 2031015 | Vlasi-3 | B11 | C:C | 0.41122 | 3.55565 |
| 2031015 | Vlasi-3 | C11 | C:C | 0.42869 | 3.56656 |
| 2031015 | Vlasi-3 | D11 | C:T | 1.64207 | 2.50305 |
| 2031015 | Vlasi-3 | E11 | C:C | 0.45507 | 3.71951 |
| 2031015 | Vlasi-3 | F11 | C:C | 0.44133 | 3.73552 |
| 2031015 | Vlasi-3 | G11 | C:C | 0.43919 | 3.68978 |
| 2031015 | Vlasi-3 | H11 | C:C | 0.50106 | 3.85087 |
| 2031015 | Vlasi-3 | A12 | C:T | 1.65063 | 2.73435 |
| 2031015 | Vlasi-3 | B12 | C:T | 1.80074 | 2.6456 |
| 2031015 | Vlasi-3 | C12 | C:C | 0.40394 | 3.58766 |
| 2031015 | Vlasi-3 | D12 | C:C | 0.46378 | 3.87065 |
| 2031015 | Vlasi-3 | E12 | C:C | 0.42739 | 3.67581 |
| 2031015 | Vlasi-3 | F12 | C:C | 0.41935 | 3.6496 |
| 2031015 | Vlasi-3 | G12 | C:C | 0.48512 | 3.59184 |
| 2031015 | Vlasi-3 | H12 | C:C | 0.52962 | 3.57615 |
| 2031015 | Vlasi-4 | A01 | C:C | 0.48867 | 3.63443 |
| 2031015 | Vlasi-4 | B01 | C:C | 0.43323 | 3.69933 |
| 2031015 | Vlasi-4 | C01 | T:T | 3.19522 | 0.52407 |
| 2031015 | Vlasi-4 | D01 | C:T | 1.42297 | 2.24211 |
| 2031015 | Vlasi-4 | E01 | C:C | 0.42593 | 3.35299 |
| 2031015 | Vlasi-4 | F01 | C:T | 1.69013 | 2.31402 |
| 2031015 | Vlasi-4 | G01 | C:C | 0.48048 | 3.23936 |
| 2031015 | Vlasi-4 | H01 | C:C | 0.45384 | 3.48754 |
| 2031015 | Vlasi-4 | A02 | C:T | 1.81925 | 2.59627 |
| 2031015 | Vlasi-4 | B02 | C:C | 0.43182 | 3.50441 |
| 2031015 | Vlasi-4 | C02 | ? | 0.53105 | 2.31633 |
| 2031015 | Vlasi-4 | D02 | C:C | 0.49111 | 3.78158 |
| 2031015 | Vlasi-4 | E02 | C:T | 1.60472 | 2.44439 |
| 2031015 | Vlasi-4 | F02 | C:C | 0.4286 | 3.43788 |
| 2031015 | Vlasi-4 | G02 | C:C | 0.4559 | 3.54787 |
| 2031015 | Vlasi-4 | H02 | C:T | 1.77294 | 2.67801 |
| 2031015 | Vlasi-4 | A03 | C:C | 0.45648 | 3.44923 |
| 2031015 | Vlasi-4 | B03 | C:C | 0.41879 | 3.47825 |
| 2031015 | Vlasi-4 | C03 | C:C | 0.42436 | 3.55082 |
| 2031015 | Vlasi-4 | D03 | C:T | 1.58142 | 2.37935 |
| 2031015 | Vlasi-4 | E03 | C:C | 0.4258 | 3.24497 |
| 2031015 | Vlasi-4 | F03 | C:C | 0.4319 | 3.54591 |
| 2031015 | Vlasi-4 | G03 | T:T | 3.08821 | 0.56484 |
| 2031015 | Vlasi-4 | H03 | C:C | 0.46851 | 3.71218 |
| 2031015 | Vlasi-4 | A04 | T:T | 3.37586 | 0.57185 |
| 2031015 | Vlasi-4 | B04 | C:C | 0.4201 | 3.55528 |
| 2031015 | Vlasi-4 | C04 | C:C | 0.42278 | 3.58142 |

| | | | | | |
|---|---|---|---|---|---|
| 2031015 | Vlasi-4 | D04 | ? | 0.91357 | 1.60802 |
| 2031015 | Vlasi-4 | E04 | C:C | 0.41989 | 3.32485 |
| 2031015 | Vlasi-4 | F04 | C:C | 0.43382 | 3.57051 |
| 2031015 | Vlasi-4 | G04 | C:T | 1.62194 | 2.5456 |
| 2031015 | Vlasi-4 | H04 | C:T | 1.80421 | 2.80084 |
| 2031015 | Vlasi-4 | A05 | C:C | 0.41267 | 3.49252 |
| 2031015 | Vlasi-4 | B05 | C:T | 1.65553 | 2.41249 |
| 2031015 | Vlasi-4 | C05 | C:C | 0.42894 | 3.29886 |
| 2031015 | Vlasi-4 | D05 | C:C | 0.42541 | 3.49772 |
| 2031015 | Vlasi-4 | E05 | C:C | 0.41694 | 3.34524 |
| 2031015 | Vlasi-4 | F05 | C:C | 0.428 | 3.16966 |
| 2031015 | Vlasi-4 | G05 | C:C | 0.45057 | 3.36022 |
| 2031015 | Vlasi-4 | H05 | C:C | 0.46837 | 3.67446 |
| 2031015 | Vlasi-4 | A06 | C:C | 0.44609 | 3.33889 |
| 2031015 | Vlasi-4 | B06 | C:C | 0.42519 | 3.36626 |
| 2031015 | Vlasi-4 | C06 | C:C | 0.41911 | 3.59565 |
| 2031015 | Vlasi-4 | D06 | C:C | 0.45581 | 2.94116 |
| 2031015 | Vlasi-4 | E06 | C:C | 0.44529 | 3.48874 |
| 2031015 | Vlasi-4 | F06 | C:C | 0.48711 | 3.16693 |
| 2031015 | Vlasi-4 | G06 | T:T | 3.10983 | 0.60018 |
| 2031015 | Vlasi-4 | H06 | C:C | 0.47979 | 3.50358 |
| 2031015 | Vlasi-4 | A07 | T:T | 2.92422 | 0.95666 |
| 2031015 | Vlasi-4 | B07 | ? | 0.62536 | 0.6112 |
| 2031015 | Vlasi-4 | C07 | ? | 0.63596 | 0.67488 |
| 2031015 | Vlasi-4 | D07 | ? | 0.66187 | 0.66237 |
| 2031015 | Vlasi-4 | E07 | ? | 0.66919 | 0.65561 |
| 2031015 | Vlasi-4 | F07 | T:T | 3.05967 | 0.94138 |
| 2031015 | Vlasi-4 | G07 | T:T | 3.24288 | 0.93166 |
| 2031015 | Vlasi-4 | H07 | T:T | 3.17178 | 1.04245 |
| 2031015 | Vlasi-4 | A08 | T:T | 3.30779 | 1.14013 |
| 2031015 | Vlasi-4 | B08 | T:T | 3.14728 | 1.01227 |
| 2031015 | Vlasi-4 | C08 | T:T | 3.07828 | 0.98541 |
| 2031015 | Vlasi-4 | D08 | T:T | 3.23679 | 1.00291 |
| 2031015 | Vlasi-4 | E08 | T:T | 3.14851 | 0.96227 |
| 2031015 | Vlasi-4 | F08 | T:T | 3.06887 | 0.99937 |
| 2031015 | Vlasi-4 | G08 | T:T | 3.27491 | 1.01284 |
| 2031015 | Vlasi-4 | H08 | T:T | 3.14592 | 1.14106 |
| 2031015 | Vlasi-4 | A09 | T:T | 3.15055 | 1.14825 |
| 2031015 | Vlasi-4 | B09 | T:T | 3.25535 | 1.0828 |
| 2031015 | Vlasi-4 | C09 | T:T | 3.12249 | 1.19668 |
| 2031015 | Vlasi-4 | D09 | T:T | 3.06672 | 1.00552 |
| 2031015 | Vlasi-4 | E09 | T:T | 3.04255 | 0.98699 |
| 2031015 | Vlasi-4 | F09 | T:T | 3.1271 | 1.06499 |
| 2031015 | Vlasi-4 | G09 | T:T | 2.96912 | 1.06799 |
| 2031015 | Vlasi-4 | H09 | T:T | 3.24075 | 1.22737 |
| 2031015 | Vlasi-4 | A10 | T:T | 3.18714 | 1.06589 |
| 2031015 | Vlasi-4 | B10 | T:T | 3.22617 | 0.97401 |
| 2031015 | Vlasi-4 | C10 | C:C | 0.43878 | 3.4008 |
| 2031015 | Vlasi-4 | D10 | C:C | 0.41993 | 3.63169 |
| 2031015 | Vlasi-4 | E10 | C:C | 0.47383 | 3.60353 |

| | | | | | |
|---|---|---|---|---|---|
| 2031015 | Vlasi-4 | F10 | C:C | 0.45697 | 3.38903 |
| 2031015 | Vlasi-4 | G10 | C:C | 0.50324 | 3.66912 |
| 2031015 | Vlasi-4 | H10 | C:C | 0.53144 | 3.40951 |
| 2031015 | Vlasi-4 | A11 | C:C | 0.56175 | 3.51591 |
| 2031015 | Vlasi-4 | B11 | C:C | 0.48774 | 3.49465 |
| 2031015 | Vlasi-4 | C11 | C:C | 0.47963 | 3.60959 |
| 2031015 | Vlasi-4 | D11 | C:C | 0.48113 | 3.56424 |
| 2031015 | Vlasi-4 | E11 | C:C | 0.50249 | 3.40589 |
| 2031015 | Vlasi-4 | F11 | C:C | 0.46153 | 3.47988 |
| 2031015 | Vlasi-4 | G11 | C:C | 0.5114 | 3.49053 |
| 2031015 | Vlasi-4 | H11 | C:C | 0.52407 | 3.55651 |
| 2031015 | Vlasi-4 | A12 | C:C | 0.60863 | 3.54003 |
| 2031015 | Vlasi-4 | B12 | C:C | 0.48456 | 3.53361 |
| 2031015 | Vlasi-4 | C12 | C:C | 0.56454 | 3.53337 |
| 2031015 | Vlasi-4 | D12 | C:C | 0.5594 | 3.4305 |
| 2031015 | Vlasi-4 | E12 | C:C | 0.53137 | 3.5917 |
| 2031015 | Vlasi-4 | F12 | C:C | 0.53325 | 3.50788 |
| 2031015 | Vlasi-4 | G12 | C:C | 0.59264 | 3.50059 |
| 2031015 | Vlasi-4 | H12 | NTC | 0.77437 | 0.76139 |
| 2031015 | Vlasi-1 | A01 | T:T | 0.54259 | 1.56618 |
| 2031015 | Vlasi-1 | B01 | T:T | 0.53003 | 1.89689 |
| 2031015 | Vlasi-1 | C01 | T:T | 0.49668 | 2.2528 |
| 2031015 | Vlasi-1 | D01 | T:T | 0.48438 | 2.18195 |
| 2031015 | Vlasi-1 | E01 | T:T | 0.4852 | 2.15405 |
| 2031015 | Vlasi-1 | F01 | T:T | 0.46845 | 2.46877 |
| 2031015 | Vlasi-1 | G01 | T:T | 0.51678 | 2.03037 |
| 2031015 | Vlasi-1 | H01 | T:T | 0.54113 | 2.19133 |
| 2031015 | Vlasi-1 | A02 | T:T | 0.45994 | 3.08011 |
| 2031015 | Vlasi-1 | B02 | ? | 0.83054 | 0.51861 |
| 2031015 | Vlasi-1 | C02 | T:T | 0.51585 | 1.87477 |
| 2031015 | Vlasi-1 | D02 | ? | 0.64557 | 0.64003 |
| 2031015 | Vlasi-1 | E02 | ? | 0.63842 | 0.43834 |
| 2031015 | Vlasi-1 | F02 | T:T | 0.48847 | 2.17566 |
| 2031015 | Vlasi-1 | G02 | T:T | 0.46998 | 2.80395 |
| 2031015 | Vlasi-1 | H02 | T:T | 0.4756 | 2.58802 |
| 2031015 | Vlasi-1 | A03 | ? | 0.60456 | 0.55543 |
| 2031015 | Vlasi-1 | B03 | ? | 0.90956 | 0.42934 |
| 2031015 | Vlasi-1 | C03 | T:T | 0.48182 | 2.68043 |
| 2031015 | Vlasi-1 | D03 | T:T | 0.52479 | 1.44645 |
| 2031015 | Vlasi-1 | E03 | T:T | 0.48386 | 2.92259 |
| 2031015 | Vlasi-1 | F03 | T:T | 0.50319 | 2.13815 |
| 2031015 | Vlasi-1 | G03 | T:T | 0.48076 | 2.24922 |
| 2031015 | Vlasi-1 | H03 | T:T | 0.48665 | 2.39837 |
| 2031015 | Vlasi-1 | A04 | ? | 0.6122 | 0.49847 |
| 2031015 | Vlasi-1 | B04 | T:T | 0.50815 | 2.57375 |
| 2031015 | Vlasi-1 | C04 | T:T | 0.60276 | 1.61785 |
| 2031015 | Vlasi-1 | D04 | ? | 0.73026 | 0.46801 |
| 2031015 | Vlasi-1 | E04 | ? | 0.68708 | 0.48345 |
| 2031015 | Vlasi-1 | F04 | T:T | 0.60412 | 1.1128 |
| 2031015 | Vlasi-1 | G04 | T:T | 0.60059 | 1.67626 |

| | | | | | |
|---|---|---|---|---|---|
| 2031015 | Vlasi-1 | H04 | T:T | 0.49141 | 2.44556 |
| 2031015 | Vlasi-1 | A05 | ? | 0.60794 | 0.54705 |
| 2031015 | Vlasi-1 | B05 | ? | 0.72175 | 0.47629 |
| 2031015 | Vlasi-1 | C05 | ? | 0.79724 | 0.52981 |
| 2031015 | Vlasi-1 | D05 | ? | 0.68694 | 0.48261 |
| 2031015 | Vlasi-1 | E05 | T:T | 0.57528 | 1.34955 |
| 2031015 | Vlasi-1 | F05 | ? | 1.06015 | 0.41329 |
| 2031015 | Vlasi-1 | G05 | ? | 0.84756 | 0.53464 |
| 2031015 | Vlasi-1 | H05 | T:T | 0.50803 | 2.51384 |
| 2031015 | Vlasi-1 | A06 | T:T | 0.47844 | 2.76608 |
| 2031015 | Vlasi-1 | B06 | T:T | 0.52648 | 2.34072 |
| 2031015 | Vlasi-1 | C06 | T:T | 0.50483 | 2.17736 |
| 2031015 | Vlasi-1 | D06 | ? | 0.65873 | 0.76973 |
| 2031015 | Vlasi-1 | E06 | T:T | 0.56066 | 1.61197 |
| 2031015 | Vlasi-1 | F06 | T:T | 0.57087 | 1.39792 |
| 2031015 | Vlasi-1 | G06 | T:T | 0.53476 | 1.97038 |
| 2031015 | Vlasi-1 | H06 | T:T | 0.57039 | 1.59339 |
| 2031015 | Vlasi-1 | A07 | T:T | 0.46256 | 3.42658 |
| 2031015 | Vlasi-1 | B07 | ? | 0.75538 | 0.46014 |
| 2031015 | Vlasi-1 | C07 | T:T | 0.61736 | 1.77996 |
| 2031015 | Vlasi-1 | D07 | T:T | 0.51518 | 1.60982 |
| 2031015 | Vlasi-1 | E07 | ? | 0.691 | 0.47116 |
| 2031015 | Vlasi-1 | F07 | T:T | 0.44147 | 2.33636 |
| 2031015 | Vlasi-1 | G07 | T:T | 0.53495 | 2.4687 |
| 2031015 | Vlasi-1 | H07 | T:T | 0.53636 | 2.50216 |
| 2031015 | Vlasi-1 | A08 | T:T | 0.51542 | 2.68706 |
| 2031015 | Vlasi-1 | B08 | T:T | 0.63597 | 1.67137 |
| 2031015 | Vlasi-1 | C08 | T:T | 0.60421 | 1.5406 |
| 2031015 | Vlasi-1 | D08 | ? | 0.64251 | 0.49776 |
| 2031015 | Vlasi-1 | E08 | T:T | 0.46272 | 2.49755 |
| 2031015 | Vlasi-1 | F08 | T:T | 0.42031 | 2.65751 |
| 2031015 | Vlasi-1 | G08 | ? | 0.66924 | 0.48496 |
| 2031015 | Vlasi-1 | H08 | T:T | 0.52327 | 2.46514 |
| 2031015 | Vlasi-1 | A09 | T:T | 0.45989 | 3.06692 |
| 2031015 | Vlasi-1 | B09 | T:T | 0.49194 | 2.52475 |
| 2031015 | Vlasi-1 | C09 | ? | 0.71993 | 0.48449 |
| 2031015 | Vlasi-1 | D09 | ? | 0.96598 | 0.41856 |
| 2031015 | Vlasi-1 | E09 | T:T | 0.46684 | 2.60581 |
| 2031015 | Vlasi-1 | F09 | ? | 0.67854 | 0.44481 |
| 2031015 | Vlasi-1 | G09 | T:T | 0.43594 | 2.70389 |
| 2031015 | Vlasi-1 | H09 | T:T | 0.48889 | 3.1495 |
| 2031015 | Vlasi-1 | A10 | T:T | 0.51783 | 2.31597 |
| 2031015 | Vlasi-1 | B10 | ? | 0.63297 | 0.50714 |
| 2031015 | Vlasi-1 | C10 | T:T | 0.42149 | 3.13171 |
| 2031015 | Vlasi-1 | D10 | T:T | 0.50035 | 2.43899 |
| 2031015 | Vlasi-1 | E10 | T:T | 0.46739 | 2.32019 |
| 2031015 | Vlasi-1 | F10 | T:T | 0.48499 | 2.64725 |
| 2031015 | Vlasi-1 | G10 | T:T | 0.49575 | 2.59253 |
| 2031015 | Vlasi-1 | H10 | T:T | 0.51889 | 3.08484 |
| 2031015 | Vlasi-1 | A11 | T:T | 0.49126 | 3.26118 |

| | | | | | |
|---|---|---|---|---|---|
| 2031015 | Vlasi-1 | B11 | T:T | 0.46146 | 2.76411 |
| 2031015 | Vlasi-1 | C11 | T:T | 0.45727 | 2.64307 |
| 2031015 | Vlasi-1 | D11 | T:T | 0.45229 | 2.92206 |
| 2031015 | Vlasi-1 | E11 | T:T | 0.46257 | 2.72317 |
| 2031015 | Vlasi-1 | F11 | T:T | 0.47328 | 2.57864 |
| 2031015 | Vlasi-1 | G11 | T:T | 0.47175 | 2.87691 |
| 2031015 | Vlasi-1 | H11 | T:T | 0.45917 | 3.08034 |
| 2031015 | Vlasi-1 | A12 | T:T | 0.47001 | 2.9698 |
| 2031015 | Vlasi-1 | B12 | T:T | 0.43728 | 3.20002 |
| 2031015 | Vlasi-1 | C12 | T:T | 0.44573 | 2.83332 |
| 2031015 | Vlasi-1 | D12 | T:T | 0.43062 | 3.15406 |
| 2031015 | Vlasi-1 | E12 | T:T | 0.42536 | 3.19303 |
| 2031015 | Vlasi-1 | F12 | T:T | 0.43332 | 3.2533 |
| 2031015 | Vlasi-1 | G12 | T:T | 0.47241 | 3.20509 |
| 2031015 | Vlasi-1 | H12 | T:T | 0.43755 | 3.26489 |
| 2031015 | Vlasi-2 | A01 | T:T | 0.55494 | 1.17194 |
| 2031015 | Vlasi-2 | B01 | T:T | 0.55665 | 1.29348 |
| 2031015 | Vlasi-2 | C01 | T:T | 0.57449 | 1.48135 |
| 2031015 | Vlasi-2 | D01 | ? | 0.8125 | 0.45402 |
| 2031015 | Vlasi-2 | E01 | ? | 0.61766 | 0.89382 |
| 2031015 | Vlasi-2 | F01 | ? | 0.83735 | 0.40241 |
| 2031015 | Vlasi-2 | G01 | ? | 0.77266 | 0.49086 |
| 2031015 | Vlasi-2 | H01 | T:T | 0.59119 | 1.82635 |
| 2031015 | Vlasi-2 | A02 | T:T | 0.46212 | 2.97025 |
| 2031015 | Vlasi-2 | B02 | ? | 0.73587 | 0.44822 |
| 2031015 | Vlasi-2 | C02 | T:T | 0.50007 | 2.25804 |
| 2031015 | Vlasi-2 | D02 | T:T | 0.58248 | 1.13107 |
| 2031015 | Vlasi-2 | E02 | T:T | 0.51408 | 1.9269 |
| 2031015 | Vlasi-2 | F02 | ? | 0.64545 | 0.43396 |
| 2031015 | Vlasi-2 | G02 | T:T | 0.56516 | 1.66775 |
| 2031015 | Vlasi-2 | H02 | T:T | 0.60678 | 1.57837 |
| 2031015 | Vlasi-2 | A03 | T:T | 0.50463 | 3.35421 |
| 2031015 | Vlasi-2 | B03 | T:T | 0.48379 | 2.35205 |
| 2031015 | Vlasi-2 | C03 | T:T | 0.43662 | 3.05799 |
| 2031015 | Vlasi-2 | D03 | ? | 0.58088 | 0.40501 |
| 2031015 | Vlasi-2 | E03 | T:T | 0.48545 | 2.4421 |
| 2031015 | Vlasi-2 | F03 | ? | 0.71314 | 0.44281 |
| 2031015 | Vlasi-2 | G03 | T:T | 0.5571 | 1.24192 |
| 2031015 | Vlasi-2 | H03 | T:T | 0.52546 | 2.12056 |
| 2031015 | Vlasi-2 | A04 | T:T | 0.46073 | 3.05418 |
| 2031015 | Vlasi-2 | B04 | T:T | 0.56541 | 1.46672 |
| 2031015 | Vlasi-2 | C04 | T:T | 0.48121 | 2.50686 |
| 2031015 | Vlasi-2 | D04 | T:T | 0.54514 | 1.63134 |
| 2031015 | Vlasi-2 | E04 | ? | 1.03022 | 0.35981 |
| 2031015 | Vlasi-2 | F04 | ? | 0.94582 | 0.44622 |
| 2031015 | Vlasi-2 | G04 | ? | 1.05174 | 0.46288 |
| 2031015 | Vlasi-2 | H04 | T:T | 0.51944 | 2.60477 |
| 2031015 | Vlasi-2 | A05 | ? | 0.65044 | 0.73103 |
| 2031015 | Vlasi-2 | B05 | T:T | 0.47876 | 2.76187 |
| 2031015 | Vlasi-2 | C05 | ? | 0.66835 | 0.45735 |

| | | | | | |
|---|---|---|---|---|---|
| 2031015 | Vlasi-2 | D05 | ? | 0.88212 | 0.44237 |
| 2031015 | Vlasi-2 | E05 | ? | 0.68602 | 0.44697 |
| 2031015 | Vlasi-2 | F05 | ? | 0.64961 | 0.44198 |
| 2031015 | Vlasi-2 | G05 | ? | 0.99824 | 0.47688 |
| 2031015 | Vlasi-2 | H05 | ? | 0.83971 | 0.55436 |
| 2031015 | Vlasi-2 | A06 | ? | 0.73223 | 0.51296 |
| 2031015 | Vlasi-2 | B06 | T:T | 0.47106 | 2.11137 |
| 2031015 | Vlasi-2 | C06 | ? | 0.7335 | 0.47124 |
| 2031015 | Vlasi-2 | D06 | ? | 0.60002 | 0.81306 |
| 2031015 | Vlasi-2 | E06 | T:T | 0.51049 | 2.08224 |
| 2031015 | Vlasi-2 | F06 | T:T | 0.48959 | 2.08828 |
| 2031015 | Vlasi-2 | G06 | T:T | 0.59275 | 1.46601 |
| 2031015 | Vlasi-2 | H06 | T:T | 0.46824 | 3.08202 |
| 2031015 | Vlasi-2 | A07 | T:T | 0.50843 | 2.45003 |
| 2031015 | Vlasi-2 | B07 | ? | 0.97447 | 0.41027 |
| 2031015 | Vlasi-2 | C07 | ? | 0.67789 | 0.46942 |
| 2031015 | Vlasi-2 | D07 | ? | 0.8574 | 0.49187 |
| 2031015 | Vlasi-2 | E07 | ? | 0.6513 | 0.48284 |
| 2031015 | Vlasi-2 | F07 | T:T | 0.44453 | 2.64914 |
| 2031015 | Vlasi-2 | G07 | T:T | 0.56798 | 1.31962 |
| 2031015 | Vlasi-2 | H07 | ? | 0.76185 | 0.5072 |
| 2031015 | Vlasi-2 | A08 | T:T | 0.55917 | 2.31005 |
| 2031015 | Vlasi-2 | B08 | ? | 0.66779 | 0.44704 |
| 2031015 | Vlasi-2 | C08 | ? | 1.12993 | 0.3254 |
| 2031015 | Vlasi-2 | D08 | T:T | 0.57256 | 1.29633 |
| 2031015 | Vlasi-2 | E08 | T:T | 0.4688 | 2.43998 |
| 2031015 | Vlasi-2 | F08 | T:T | 0.507 | 2.1443 |
| 2031015 | Vlasi-2 | G08 | T:T | 0.51072 | 2.3289 |
| 2031015 | Vlasi-2 | H08 | T:T | 0.47709 | 2.59655 |
| 2031015 | Vlasi-2 | A09 | ? | 0.65285 | 0.56153 |
| 2031015 | Vlasi-2 | B09 | ? | 1.00752 | 0.46714 |
| 2031015 | Vlasi-2 | C09 | ? | 1.08228 | 0.37173 |
| 2031015 | Vlasi-2 | D09 | ? | 0.82626 | 0.44847 |
| 2031015 | Vlasi-2 | E09 | ? | 0.95984 | 0.45427 |
| 2031015 | Vlasi-2 | F09 | ? | 0.66322 | 0.45235 |
| 2031015 | Vlasi-2 | G09 | T:T | 0.61869 | 1.14484 |
| 2031015 | Vlasi-2 | H09 | ? | 0.66894 | 0.60735 |
| 2031015 | Vlasi-2 | A10 | T:T | 0.50155 | 3.02971 |
| 2031015 | Vlasi-2 | B10 | T:T | 0.54946 | 1.51472 |
| 2031015 | Vlasi-2 | C10 | ? | 0.71599 | 0.446 |
| 2031015 | Vlasi-2 | D10 | ? | 0.60705 | 0.69926 |
| 2031015 | Vlasi-2 | E10 | T:T | 0.60732 | 1.17008 |
| 2031015 | Vlasi-2 | F10 | ? | 0.69471 | 0.49196 |
| 2031015 | Vlasi-2 | G10 | ? | 0.92946 | 0.45549 |
| 2031015 | Vlasi-2 | H10 | T:T | 0.6064 | 2.05979 |
| 2031015 | Vlasi-2 | A11 | T:T | 0.47487 | 3.38051 |
| 2031015 | Vlasi-2 | B11 | T:T | 0.47682 | 2.47124 |
| 2031015 | Vlasi-2 | C11 | ? | 0.63382 | 0.49185 |
| 2031015 | Vlasi-2 | D11 | T:T | 0.45262 | 2.71299 |
| 2031015 | Vlasi-2 | E11 | T:T | 0.55789 | 2.18052 |

| | | | | | |
|---|---|---|---|---|---|
| 2031015 | Vlasi-2 | F11 | ? | 0.69765 | 0.48558 |
| 2031015 | Vlasi-2 | G11 | T:T | 0.46799 | 3.08365 |
| 2031015 | Vlasi-2 | H11 | T:T | 0.46967 | 3.2231 |
| 2031015 | Vlasi-2 | A12 | T:T | 0.52893 | 2.18503 |
| 2031015 | Vlasi-2 | B12 | T:T | 0.51073 | 3.4049 |
| 2031015 | Vlasi-2 | C12 | T:T | 0.47147 | 3.15958 |
| 2031015 | Vlasi-2 | D12 | T:T | 0.46749 | 3.55609 |
| 2031015 | Vlasi-2 | E12 | T:T | 0.49417 | 2.80958 |
| 2031015 | Vlasi-2 | F12 | T:T | 0.46591 | 3.26865 |
| 2031015 | Vlasi-2 | G12 | T:T | 0.46528 | 3.08305 |
| 2031015 | Vlasi-2 | H12 | T:T | 0.48033 | 3.08422 |
| 2031015 | Vlasi-3 | A01 | T:T | 0.47066 | 3.16715 |
| 2031015 | Vlasi-3 | B01 | T:T | 0.49746 | 2.67892 |
| 2031015 | Vlasi-3 | C01 | T:T | 0.461 | 2.41542 |
| 2031015 | Vlasi-3 | D01 | T:T | 0.48286 | 1.89219 |
| 2031015 | Vlasi-3 | E01 | T:T | 0.47111 | 2.4612 |
| 2031015 | Vlasi-3 | F01 | T:T | 0.48252 | 2.30934 |
| 2031015 | Vlasi-3 | G01 | T:T | 0.52434 | 2.36539 |
| 2031015 | Vlasi-3 | H01 | T:T | 0.484 | 3.22222 |
| 2031015 | Vlasi-3 | A02 | T:T | 0.45975 | 3.067 |
| 2031015 | Vlasi-3 | B02 | T:T | 0.48218 | 1.68649 |
| 2031015 | Vlasi-3 | C02 | T:T | 0.43379 | 2.56532 |
| 2031015 | Vlasi-3 | D02 | ? | 0.59846 | 0.44542 |
| 2031015 | Vlasi-3 | E02 | T:T | 0.44584 | 2.09168 |
| 2031015 | Vlasi-3 | F02 | T:T | 0.44933 | 2.46828 |
| 2031015 | Vlasi-3 | G02 | T:T | 0.52659 | 2.17107 |
| 2031015 | Vlasi-3 | H02 | T:T | 0.48935 | 3.19396 |
| 2031015 | Vlasi-3 | A03 | T:T | 0.45216 | 3.00784 |
| 2031015 | Vlasi-3 | B03 | T:T | 0.48859 | 2.28432 |
| 2031015 | Vlasi-3 | C03 | T:T | 0.48324 | 2.22792 |
| 2031015 | Vlasi-3 | D03 | T:T | 0.4666 | 2.08971 |
| 2031015 | Vlasi-3 | E03 | T:T | 0.47972 | 2.44516 |
| 2031015 | Vlasi-3 | F03 | ? | 0.86958 | 0.46816 |
| 2031015 | Vlasi-3 | G03 | ? | 1.06505 | 0.50446 |
| 2031015 | Vlasi-3 | H03 | T:T | 0.49186 | 3.22871 |
| 2031015 | Vlasi-3 | A04 | T:T | 0.51324 | 1.8602 |
| 2031015 | Vlasi-3 | B04 | T:T | 0.57185 | 1.29997 |
| 2031015 | Vlasi-3 | C04 | T:T | 0.46938 | 2.2267 |
| 2031015 | Vlasi-3 | D04 | T:T | 0.48961 | 1.6647 |
| 2031015 | Vlasi-3 | E04 | T:T | 0.47719 | 2.24761 |
| 2031015 | Vlasi-3 | F04 | T:T | 0.54615 | 1.78292 |
| 2031015 | Vlasi-3 | G04 | ? | 1.02543 | 0.55812 |
| 2031015 | Vlasi-3 | H04 | T:T | 0.46748 | 3.23232 |
| 2031015 | Vlasi-3 | A05 | T:T | 0.45481 | 2.9539 |
| 2031015 | Vlasi-3 | B05 | T:T | 0.43763 | 3.0223 |
| 2031015 | Vlasi-3 | C05 | T:T | 0.43633 | 3.30514 |
| 2031015 | Vlasi-3 | D05 | ? | 0.63398 | 0.4358 |
| 2031015 | Vlasi-3 | E05 | T:T | 0.56491 | 1.04763 |
| 2031015 | Vlasi-3 | F05 | T:T | 0.52498 | 2.00325 |
| 2031015 | Vlasi-3 | G05 | T:T | 0.47169 | 2.48616 |

| | | | | | |
|---|---|---|---|---|---|
| 2031015 | Vlasi-3 | H05 | T:T | 0.54531 | 2.9224 |
| 2031015 | Vlasi-3 | A06 | T:T | 0.41888 | 2.56593 |
| 2031015 | Vlasi-3 | B06 | ? | 0.68609 | 0.52442 |
| 2031015 | Vlasi-3 | C06 | T:T | 0.52429 | 1.71771 |
| 2031015 | Vlasi-3 | D06 | T:T | 0.4702 | 1.92716 |
| 2031015 | Vlasi-3 | E06 | T:T | 0.48214 | 2.12714 |
| 2031015 | Vlasi-3 | F06 | T:T | 0.49698 | 2.31774 |
| 2031015 | Vlasi-3 | G06 | T:T | 0.46855 | 2.81229 |
| 2031015 | Vlasi-3 | H06 | T:T | 0.61335 | 1.05532 |
| 2031015 | Vlasi-3 | A07 | T:T | 0.50319 | 2.35883 |
| 2031015 | Vlasi-3 | B07 | T:T | 0.46737 | 2.26511 |
| 2031015 | Vlasi-3 | C07 | ? | 0.61542 | 0.44244 |
| 2031015 | Vlasi-3 | D07 | T:T | 0.48999 | 2.12678 |
| 2031015 | Vlasi-3 | E07 | T:T | 0.52147 | 1.88477 |
| 2031015 | Vlasi-3 | F07 | T:T | 0.49993 | 2.03669 |
| 2031015 | Vlasi-3 | G07 | T:T | 0.47263 | 2.76971 |
| 2031015 | Vlasi-3 | H07 | T:T | 0.50614 | 3.47553 |
| 2031015 | Vlasi-3 | A08 | T:T | 0.47732 | 3.03978 |
| 2031015 | Vlasi-3 | B08 | T:T | 0.47466 | 1.98239 |
| 2031015 | Vlasi-3 | C08 | T:T | 0.49993 | 1.55347 |
| 2031015 | Vlasi-3 | D08 | T:T | 0.50268 | 2.21966 |
| 2031015 | Vlasi-3 | E08 | T:T | 0.48812 | 1.98033 |
| 2031015 | Vlasi-3 | F08 | T:T | 0.51658 | 2.27109 |
| 2031015 | Vlasi-3 | G08 | T:T | 0.55679 | 2.05774 |
| 2031015 | Vlasi-3 | H08 | T:T | 0.49615 | 3.23235 |
| 2031015 | Vlasi-3 | A09 | T:T | 0.48183 | 2.7678 |
| 2031015 | Vlasi-3 | B09 | T:T | 0.4679 | 2.21047 |
| 2031015 | Vlasi-3 | C09 | T:T | 0.5701 | 1.13884 |
| 2031015 | Vlasi-3 | D09 | T:T | 0.4512 | 2.66775 |
| 2031015 | Vlasi-3 | E09 | T:T | 0.41227 | 2.87935 |
| 2031015 | Vlasi-3 | F09 | T:T | 0.46708 | 2.21648 |
| 2031015 | Vlasi-3 | G09 | T:T | 0.46568 | 2.95186 |
| 2031015 | Vlasi-3 | H09 | T:T | 0.5151 | 3.34879 |
| 2031015 | Vlasi-3 | A10 | T:T | 0.49447 | 2.20114 |
| 2031015 | Vlasi-3 | B10 | T:T | 0.45957 | 2.99036 |
| 2031015 | Vlasi-3 | C10 | T:T | 0.47014 | 2.47107 |
| 2031015 | Vlasi-3 | D10 | T:T | 0.43863 | 2.63356 |
| 2031015 | Vlasi-3 | E10 | T:T | 0.40423 | 2.80973 |
| 2031015 | Vlasi-3 | F10 | T:T | 0.50662 | 2.08386 |
| 2031015 | Vlasi-3 | G10 | T:T | 0.49397 | 2.75903 |
| 2031015 | Vlasi-3 | H10 | T:T | 0.50576 | 3.30807 |
| 2031015 | Vlasi-3 | A11 | T:T | 0.41764 | 3.02862 |
| 2031015 | Vlasi-3 | B11 | T:T | 0.45208 | 3.31634 |
| 2031015 | Vlasi-3 | C11 | T:T | 0.43909 | 3.26421 |
| 2031015 | Vlasi-3 | D11 | T:T | 0.43134 | 3.02218 |
| 2031015 | Vlasi-3 | E11 | T:T | 0.44462 | 2.85899 |
| 2031015 | Vlasi-3 | F11 | T:T | 0.43798 | 2.93161 |
| 2031015 | Vlasi-3 | G11 | T:T | 0.46315 | 3.14047 |
| 2031015 | Vlasi-3 | H11 | T:T | 0.48126 | 3.4006 |
| 2031015 | Vlasi-3 | A12 | T:T | 0.47003 | 3.18327 |

| | | | | | |
|---|---|---|---|---|---|
| 2031015 | Vlasi-3 | B12 | T:T | 0.44313 | 3.25566 |
| 2031015 | Vlasi-3 | C12 | T:T | 0.43928 | 3.1461 |
| 2031015 | Vlasi-3 | D12 | T:T | 0.43443 | 3.51072 |
| 2031015 | Vlasi-3 | E12 | T:T | 0.42856 | 3.26714 |
| 2031015 | Vlasi-3 | F12 | ? | 0.58116 | 0.4859 |
| 2031015 | Vlasi-3 | G12 | T:T | 0.47381 | 2.88111 |
| 2031015 | Vlasi-3 | H12 | T:T | 0.68412 | 3.65535 |
| 2031015 | Vlasi-4 | A01 | ? | 0.62652 | 0.59213 |
| 2031015 | Vlasi-4 | B01 | T:T | 0.54218 | 1.13325 |
| 2031015 | Vlasi-4 | C01 | ? | 0.53288 | 0.76475 |
| 2031015 | Vlasi-4 | D01 | ? | 0.54934 | 0.87179 |
| 2031015 | Vlasi-4 | E01 | T:T | 0.54141 | 1.16942 |
| 2031015 | Vlasi-4 | F01 | T:T | 0.53888 | 2.31521 |
| 2031015 | Vlasi-4 | G01 | T:T | 0.53388 | 1.83935 |
| 2031015 | Vlasi-4 | H01 | T:T | 0.47926 | 3.36705 |
| 2031015 | Vlasi-4 | A02 | T:T | 0.5171 | 2.31072 |
| 2031015 | Vlasi-4 | B02 | T:T | 0.43106 | 3.21538 |
| 2031015 | Vlasi-4 | C02 | ? | 0.82614 | 0.49254 |
| 2031015 | Vlasi-4 | D02 | ? | 0.67968 | 0.46989 |
| 2031015 | Vlasi-4 | E02 | T:T | 0.49209 | 2.36365 |
| 2031015 | Vlasi-4 | F02 | T:T | 0.53872 | 2.04365 |
| 2031015 | Vlasi-4 | G02 | T:T | 0.53879 | 1.72261 |
| 2031015 | Vlasi-4 | H02 | T:T | 0.50802 | 2.65826 |
| 2031015 | Vlasi-4 | A03 | T:T | 0.42213 | 3.15382 |
| 2031015 | Vlasi-4 | B03 | T:T | 0.48526 | 2.1976 |
| 2031015 | Vlasi-4 | C03 | T:T | 0.5006 | 1.55472 |
| 2031015 | Vlasi-4 | D03 | T:T | 0.50159 | 1.68653 |
| 2031015 | Vlasi-4 | E03 | T:T | 0.54153 | 1.07715 |
| 2031015 | Vlasi-4 | F03 | T:T | 0.46536 | 1.83288 |
| 2031015 | Vlasi-4 | G03 | T:T | 0.45953 | 2.79956 |
| 2031015 | Vlasi-4 | H03 | T:T | 0.51723 | 2.43063 |
| 2031015 | Vlasi-4 | A04 | ? | 0.57541 | 0.56513 |
| 2031015 | Vlasi-4 | B04 | T:T | 0.48217 | 1.83532 |
| 2031015 | Vlasi-4 | C04 | T:T | 0.43313 | 3.07257 |
| 2031015 | Vlasi-4 | D04 | ? | 0.53305 | 0.74642 |
| 2031015 | Vlasi-4 | E04 | T:T | 0.50888 | 1.48955 |
| 2031015 | Vlasi-4 | F04 | T:T | 0.45556 | 2.59698 |
| 2031015 | Vlasi-4 | G04 | T:T | 0.51119 | 2.04255 |
| 2031015 | Vlasi-4 | H04 | T:T | 0.50448 | 2.82091 |
| 2031015 | Vlasi-4 | A05 | T:T | 0.51065 | 2.1835 |
| 2031015 | Vlasi-4 | B05 | T:T | 0.45356 | 2.4208 |
| 2031015 | Vlasi-4 | C05 | T:T | 0.49459 | 1.8526 |
| 2031015 | Vlasi-4 | D05 | T:T | 0.48083 | 1.72754 |
| 2031015 | Vlasi-4 | E05 | T:T | 0.51676 | 1.07331 |
| 2031015 | Vlasi-4 | F05 | T:T | 0.54044 | 0.98285 |
| 2031015 | Vlasi-4 | G05 | T:T | 0.55125 | 1.85785 |
| 2031015 | Vlasi-4 | H05 | T:T | 0.47282 | 3.09248 |
| 2031015 | Vlasi-4 | A06 | T:T | 0.56048 | 1.24101 |
| 2031015 | Vlasi-4 | B06 | ? | 0.56049 | 0.88629 |
| 2031015 | Vlasi-4 | C06 | T:T | 0.43798 | 2.84767 |

| | | | | | |
|---|---|---|---|---|---|
| 2031015 | Vlasi-4 | D06 | ? | 0.57351 | 0.45203 |
| 2031015 | Vlasi-4 | E06 | T:T | 0.4564 | 2.58575 |
| 2031015 | Vlasi-4 | F06 | ? | 0.68567 | 0.49548 |
| 2031015 | Vlasi-4 | G06 | T:T | 0.49132 | 2.38055 |
| 2031015 | Vlasi-4 | H06 | T:T | 0.49337 | 2.88886 |
| 2031015 | Vlasi-4 | A07 | T:T | 0.47119 | 3.01695 |
| 2031015 | Vlasi-4 | B07 | T:T | 0.43628 | 3.0599 |
| 2031015 | Vlasi-4 | C07 | T:T | 0.44868 | 3.24021 |
| 2031015 | Vlasi-4 | D07 | T:T | 0.45585 | 2.65469 |
| 2031015 | Vlasi-4 | E07 | ? | 0.66811 | 0.77621 |
| 2031015 | Vlasi-4 | F07 | C:C | 3.33358 | 0.61384 |
| 2031015 | Vlasi-4 | G07 | C:C | 3.30577 | 0.71476 |
| 2031015 | Vlasi-4 | H07 | C:C | 3.47174 | 0.90389 |
| 2031015 | Vlasi-4 | A08 | C:C | 3.22564 | 0.78275 |
| 2031015 | Vlasi-4 | B08 | C:C | 3.0234 | 0.85221 |
| 2031015 | Vlasi-4 | C08 | C:C | 3.2986 | 0.77043 |
| 2031015 | Vlasi-4 | D08 | C:C | 3.48925 | 0.55368 |
| 2031015 | Vlasi-4 | E08 | C:C | 3.38387 | 0.58961 |
| 2031015 | Vlasi-4 | F08 | C:C | 3.47375 | 0.65577 |
| 2031015 | Vlasi-4 | G08 | C:C | 3.44933 | 0.78499 |
| 2031015 | Vlasi-4 | H08 | C:C | 3.75023 | 0.85676 |
| 2031015 | Vlasi-4 | A09 | C:C | 3.44524 | 0.68458 |
| 2031015 | Vlasi-4 | B09 | C:C | 3.46509 | 0.63201 |
| 2031015 | Vlasi-4 | C09 | C:C | 3.48184 | 0.7329 |
| 2031015 | Vlasi-4 | D09 | C:C | 3.45245 | 0.6488 |
| 2031015 | Vlasi-4 | E09 | C:C | 3.34968 | 0.63157 |
| 2031015 | Vlasi-4 | F09 | C:C | 3.40024 | 0.68578 |
| 2031015 | Vlasi-4 | G09 | C:C | 3.49361 | 0.81922 |
| 2031015 | Vlasi-4 | H09 | C:C | 3.61529 | 0.68324 |
| 2031015 | Vlasi-4 | A10 | C:C | 3.42048 | 0.73042 |
| 2031015 | Vlasi-4 | B10 | C:C | 3.20881 | 0.75782 |
| 2031015 | Vlasi-4 | C10 | T:T | 0.40233 | 3.22497 |
| 2031015 | Vlasi-4 | D10 | T:T | 0.42958 | 3.32891 |
| 2031015 | Vlasi-4 | E10 | T:T | 0.49139 | 3.66143 |
| 2031015 | Vlasi-4 | F10 | T:T | 0.46004 | 3.55388 |
| 2031015 | Vlasi-4 | G10 | T:T | 0.51322 | 3.63742 |
| 2031015 | Vlasi-4 | H10 | T:T | 0.56304 | 3.57046 |
| 2031015 | Vlasi-4 | A11 | T:T | 0.49217 | 3.33812 |
| 2031015 | Vlasi-4 | B11 | T:T | 0.48737 | 3.52722 |
| 2031015 | Vlasi-4 | C11 | T:T | 0.46383 | 3.52251 |
| 2031015 | Vlasi-4 | D11 | T:T | 0.46407 | 3.64478 |
| 2031015 | Vlasi-4 | E11 | T:T | 0.54192 | 3.43925 |
| 2031015 | Vlasi-4 | F11 | T:T | 0.49359 | 3.53565 |
| 2031015 | Vlasi-4 | G11 | T:T | 0.55558 | 3.65734 |
| 2031015 | Vlasi-4 | H11 | T:T | 0.74161 | 3.75776 |
| 2031015 | Vlasi-4 | A12 | T:T | 0.77314 | 3.5515 |
| 2031015 | Vlasi-4 | B12 | T:T | 0.67831 | 3.49586 |
| 2031015 | Vlasi-4 | C12 | T:T | 0.65229 | 3.47063 |
| 2031015 | Vlasi-4 | D12 | T:T | 0.65765 | 3.57638 |
| 2031015 | Vlasi-4 | E12 | T:T | 0.55158 | 3.44123 |

| | | | | | |
|---|---|---|---|---|---|
| 2031015 | Vlasi-4 | F12 | T:T | 0.54898 | 3.48074 |
| 2031015 | Vlasi-4 | G12 | T:T | 0.6033 | 3.56054 |
| 2031015 | Vlasi-4 | H12 | NTC | 0.82332 | 0.81698 |

r to find out more about the syntax from http://results,lgcgenomics,com/software/snpviewer/
KlusterCaller software from http://results,lgcgenomics,com/software/klustercaller/

| Unused | Missing | Bad | Allele Y% | Allele X% | X2 | Allele Y | Allele X | |
|---|---|---|---|---|---|---|---|---|
| 0 | 0 | 0 | 63 | 54 | 36 | 46 | 0 | 60 |
| 1 | 0 | 0 | 66 | 32 | 33 | 68 | 0 | 67 |
| 0 | 0 | 0 | 66 | 67 | 33 | 33 | 1 | 15 |
| 4 | 0 | 0 | 53 | 85 | 46 | 15 | 16 | 45 |
| 0 | 0 | 0 | 60 | 42 | 39 | 58 | 2 | 85 |
| 0 | 0 | 0 | 61 | 98 | 38 | 2 | 0 | 0 |
| 1 | 0 | 0 | 60 | 53 | 39 | 47 | 4 | 25 |
| 3 | 0 | 0 | 55 | 43 | 44 | 57 | 24 | 50 |
| 1 | 0 | 0 | 42 | 11 | 57 | 89 | 2 | 61 |
| 1 | 0 | 0 | 56 | 32 | 43 | 68 | 0 | 13 |
| 1 | 0 | 0 | 53 | 68 | 46 | 32 | 0 | 45 |
| 5 | 0 | 0 | 51 | 67 | 48 | 33 | 11 | 33 |
| 0 | 0 | 0 | 66 | 15 | 33 | 85 | 0 | 21 |
| 2 | 0 | 0 | 66 | 49 | 33 | 51 | 0 | 52 |
| 1 | 0 | 0 | 66 | 32 | 33 | 68 | 0 | 31 |
| 5 | 0 | 0 | 60 | 0 | 40 | 0 | 21 | 67 |
| 1 | 0 | 0 | 71 | 58 | 28 | 42 | 0 | 12 |
| 0 | 0 | 0 | 66 | 67 | 33 | 33 | 0 | 9 |
| 1 | 0 | 0 | 65 | 26 | 34 | 74 | 0 | 4 |
| 5 | 0 | 0 | 58 | 33 | 41 | 67 | 10 | 23 |
| 0 | 0 | 0 | 62 | 50 | 37 | 50 | 0 | 43 |
| 0 | 0 | 0 | 73 | 96 | 26 | 4 | 0 | 62 |
| 0 | 0 | 0 | 66 | 67 | 33 | 33 | 0 | 2 |
| 5 | 0 | 0 | 53 | 89 | 46 | 11 | 21 | 24 |
| 1 | 0 | 0 | 46 | 84 | 53 | 16 | 2 | 51 |
| 1 | 0 | 0 | 51 | 58 | 48 | 42 | 3 | 8 |
| 2 | 0 | 0 | 52 | 66 | 47 | 34 | 0 | 73 |
| 0 | 0 | 0 | 47 | 89 | 52 | 11 | 17 | 61 |
| 0 | 0 | 0 | 71 | 35 | 28 | 65 | 3 | 75 |
| 5 | 0 | 0 | 74 | 73 | 25 | 27 | 1 | 47 |
| 0 | 0 | 0 | 75 | 0 | 25 | 0 | 4 | 74 |
| 6 | 0 | 0 | 60 | 11 | 39 | 89 | 18 | 92 |
| 0 | 0 | 0 | 73 | 96 | 26 | 4 | 1 | 77 |
| 0 | 0 | 0 | 66 | 67 | 33 | 33 | 0 | 2 |
| 0 | 0 | 0 | 71 | 35 | 28 | 65 | 2 | 43 |
| 6 | 0 | 0 | 56 | 74 | 43 | 26 | 16 | 29 |

| | | | | | | | | |
|---|---|---|---|---|---|---|---|---|
| 0 | 0 | 0 | 50 | 52 | 49 | 48 | 0 | 37 |
| 0 | 0 | 0 | 50 | 0 | 50 | 0 | 0 | 67 |
| 0 | 0 | 0 | 53 | 12 | 46 | 87 | 1 | 42 |
| 4 | 0 | 0 | 46 | 15 | 53 | 85 | 13 | 21 |
| 0 | 0 | 0 | 52 | 60 | 47 | 40 | 0 | 35 |
| 1 | 0 | 0 | 54 | 74 | 45 | 26 | 0 | 5 |
| 0 | 0 | 0 | 54 | 17 | 45 | 83 | 0 | 0 |
| 5 | 0 | 0 | 54 | 44 | 45 | 56 | 27 | 42 |
| 5 | 0 | 0 | 67 | 58 | 32 | 42 | 0 | 4 |
| 11 | 0 | 0 | 70 | 59 | 29 | 41 | 0 | 74 |
| 3 | 0 | 0 | 69 | 89 | 30 | 11 | 4 | 77 |
| 8 | 0 | 0 | 56 | 90 | 43 | 10 | 26 | 77 |
| 16 | 0 | 0 | 64 | 37 | 35 | 62 | 0 | 81 |
| 17 | 0 | 0 | 64 | 56 | 35 | 44 | 1 | 4 |
| 5 | 0 | 0 | 65 | 38 | 34 | 62 | 0 | 0 |
| 23 | 0 | 0 | 40 | 28 | 59 | 72 | 20 | 85 |
| 1 | 0 | 0 | 66 | 32 | 33 | 68 | 0 | 67 |
| 2 | 0 | 0 | 63 | 30 | 36 | 70 | 0 | 9 |
| 2 | 0 | 0 | 64 | 89 | 35 | 11 | 1 | 37 |
| 5 | 0 | 0 | 55 | 56 | 44 | 44 | 12 | 32 |
| 1 | 0 | 0 | 58 | 95 | 41 | 5 | 0 | 18 |
| 4 | 0 | 0 | 60 | 33 | 39 | 67 | 0 | 4 |
| 1 | 0 | 0 | 56 | 32 | 43 | 68 | 0 | 13 |
| 6 | 0 | 0 | 60 | 11 | 39 | 89 | 18 | 92 |
| 2 | 0 | 0 | 76 | 60 | 23 | 40 | 0 | 44 |
| 9 | 0 | 0 | 78 | 16 | 21 | 84 | 0 | 1 |
| 1 | 0 | 0 | 75 | 26 | 24 | 74 | 0 | 1 |
| 5 | 0 | 0 | 61 | 11 | 38 | 89 | 25 | 52 |
| 4 | 0 | 0 | 80 | 43 | 19 | 57 | 0 | 96 |
| 20 | 0 | 0 | 85 | 53 | 14 | 47 | 0 | 14 |
| 1 | 0 | 0 | 81 | 58 | 18 | 42 | 0 | 28 |
| 6 | 0 | 0 | 65 | 73 | 34 | 27 | 53 | 53 |
| 23 | 0 | 0 | 100 | 0 | 0 | 0 | 0 | 0 |
| 41 | 0 | 0 | 100 | 0 | 0 | 0 | 0 | 0 |
| 8 | 0 | 0 | 100 | 0 | 0 | 0 | 0 | 0 |
| 11 | 0 | 0 | 75 | 0 | 25 | 0 | 84 | 0 |

ıeader

;/C]TGGCTTCACTTCTG
']TACCTGGAATKAAT

/C]GAGGGTCCCCGYYG
/C]TTCCTAGATGTAGC
/G]TAATATATAAAGGA
/A]GGTGTATATGTGGT
/C]GAGCTGTCATCATG
/C]AGAATTTCCTATCT
/G]TGAMTGCTCTTATT
/T]SCGGTTTGTTTTTC
/T]ATAGCACTTCCCCC
C/T]RTTGGTGGTTGCCA
C/T]GATGATNNNTATGT
G/A]TCTCACACTCRGCT
/T]CAGCAGCTTCAGGA
/C]CCTCTCTTCAAAAG
/G]AATTTCAACACGGT
C]GTTTAATTCCAAAG

Notes

| SNPID | SubjectID | Norm | Carrier | DaughterW | LongID | AliquotID |
|---|---|---|---|---|---|---|
| arh_var1 | 6284 | | | A02 | | |
| arh_var1 | 6285 | | | E02 | | |
| arh_var1 | 6286 | | | I02 | | |
| arh_var1 | 6287 | | | M02 | | |
| arh_var1 | 6288 | | | Q02 | | |
| arh_var1 | 6289 | | | U02 | | |
| arh_var1 | 6290 | | | Y02 | | |
| arh_var1 | 6291 | | | AC02 | | |
| arh_var1 | 6292 | | | A06 | | |

| | | |
|---|---|---|
| arh_var1 | 6293 | E06 |
| arh_var1 | 6294 | I06 |
| arh_var1 | 6295 | M06 |
| arh_var1 | 6296 | Q06 |
| arh_var1 | 6297 | U06 |
| arh_var1 | 6298 | Y06 |
| arh_var1 | 6299 | AC06 |
| arh_var1 | 6300 | A10 |
| arh_var1 | 6301 | E10 |
| arh_var1 | 6302 | I10 |
| arh_var1 | 6303 | M10 |
| arh_var1 | 6304 | Q10 |
| arh_var1 | 6305 | U10 |
| arh_var1 | 6306 | Y10 |
| arh_var1 | 6307 | AC10 |
| arh_var1 | 6308 | A14 |
| arh_var1 | 6309 | E14 |
| arh_var1 | 6310 | I14 |
| arh_var1 | 6311 | M14 |
| arh_var1 | 6312 | Q14 |
| arh_var1 | 6313 | U14 |
| arh_var1 | 6314 | Y14 |
| arh_var1 | 6315 | AC14 |
| arh_var1 | 6316 | A18 |
| arh_var1 | 6317 | E18 |
| arh_var1 | 6318 | I18 |
| arh_var1 | 6319 | M18 |
| arh_var1 | 6320 | Q18 |
| arh_var1 | 6321 | U18 |
| arh_var1 | 6322 | Y18 |
| arh_var1 | 6323 | AC18 |
| arh_var1 | 6324 | A22 |
| arh_var1 | 6325 | E22 |
| arh_var1 | 6326 | I22 |
| arh_var1 | 6327 | M22 |
| arh_var1 | 6328 | Q22 |
| arh_var1 | 6329 | U22 |
| arh_var1 | 6330 | Y22 |
| arh_var1 | 6331 | AC22 |
| arh_var1 | 6332 | A26 |
| arh_var1 | 6333 | E26 |
| arh_var1 | 6334 | I26 |
| arh_var1 | 6335 | M26 |
| arh_var1 | 6336 | Q26 |
| arh_var1 | 6337 | U26 |
| arh_var1 | 6338 | Y26 |
| arh_var1 | 6339 | AC26 |
| arh_var1 | 6340 | A30 |
| arh_var1 | 6341 | E30 |
| arh_var1 | 6342 | I30 |

| | | |
|---|---|---|
| arh_var1 | 6343 | M30 |
| arh_var1 | 6344 | Q30 |
| arh_var1 | 6345 | U30 |
| arh_var1 | 6346 | Y30 |
| arh_var1 | 6347 | AC30 |
| arh_var1 | 6348 | A34 |
| arh_var1 | 6349 | E34 |
| arh_var1 | 6350 | I34 |
| arh_var1 | 6351 | M34 |
| arh_var1 | 6352 | Q34 |
| arh_var1 | 6353 | U34 |
| arh_var1 | 6354 | Y34 |
| arh_var1 | 6355 | AC34 |
| arh_var1 | 6356 | A38 |
| arh_var1 | 6357 | E38 |
| arh_var1 | 6358 | I38 |
| arh_var1 | 6359 | M38 |
| arh_var1 | 6360 | Q38 |
| arh_var1 | 6361 | U38 |
| arh_var1 | 6362 | Y38 |
| arh_var1 | 6363 | AC38 |
| arh_var1 | 6364 | A42 |
| arh_var1 | 6365 | E42 |
| arh_var1 | 6366 | I42 |
| arh_var1 | 6367 | M42 |
| arh_var1 | 6368 | Q42 |
| arh_var1 | 6369 | U42 |
| arh_var1 | 6370 | Y42 |
| arh_var1 | 6371 | AC42 |
| arh_var1 | 6372 | A46 |
| arh_var1 | 6373 | E46 |
| arh_var1 | 6374 | I46 |
| arh_var1 | 6375 | M46 |
| arh_var1 | 6376 | Q46 |
| arh_var1 | 6377 | U46 |
| arh_var1 | 6378 | Y46 |
| arh_var1 | 6379 | AC46 |
| arh_var1 | 6380 | A04 |
| arh_var1 | 6381 | E04 |
| arh_var1 | 6382 | I04 |
| arh_var1 | 6383 | M04 |
| arh_var1 | 6384 | Q04 |
| arh_var1 | 6385 | U04 |
| arh_var1 | 6386 | Y04 |
| arh_var1 | 6387 | AC04 |
| arh_var1 | 6388 | A08 |
| arh_var1 | 6389 | E08 |
| arh_var1 | 6390 | I08 |
| arh_var1 | 6391 | M08 |
| arh_var1 | 6392 | Q08 |

| | | |
|---|---|---|
| arh_var1 | 6393 | U08 |
| arh_var1 | 6394 | Y08 |
| arh_var1 | 6395 | AC08 |
| arh_var1 | 6396 | A12 |
| arh_var1 | 6397 | E12 |
| arh_var1 | 6398 | I12 |
| arh_var1 | 6399 | M12 |
| arh_var1 | 6400 | Q12 |
| arh_var1 | 6401 | U12 |
| arh_var1 | 6402 | Y12 |
| arh_var1 | 6403 | AC12 |
| arh_var1 | 6404 | A16 |
| arh_var1 | 6405 | E16 |
| arh_var1 | 6406 | I16 |
| arh_var1 | 6407 | M16 |
| arh_var1 | 6408 | Q16 |
| arh_var1 | 6409 | U16 |
| arh_var1 | 6410 | Y16 |
| arh_var1 | 6411 | AC16 |
| arh_var1 | 6412 | A20 |
| arh_var1 | 6413 | E20 |
| arh_var1 | 6414 | I20 |
| arh_var1 | 6415 | M20 |
| arh_var1 | 6416 | Q20 |
| arh_var1 | 6417 | U20 |
| arh_var1 | 6418 | Y20 |
| arh_var1 | 6419 | AC20 |
| arh_var1 | 6421 | A24 |
| arh_var1 | 6422 | E24 |
| arh_var1 | 6423 | I24 |
| arh_var1 | 6424 | M24 |
| arh_var1 | 6425 | Q24 |
| arh_var1 | 6426 | U24 |
| arh_var1 | 6427 | Y24 |
| arh_var1 | 6428 | AC24 |
| arh_var1 | 6429 | A28 |
| arh_var1 | 6430 | E28 |
| arh_var1 | 6431 | I28 |
| arh_var1 | 6432 | M28 |
| arh_var1 | 6433 | Q28 |
| arh_var1 | 6434 | U28 |
| arh_var1 | 6435 | Y28 |
| arh_var1 | 6436 | AC28 |
| arh_var1 | 6437 | A32 |
| arh_var1 | 6438 | E32 |
| arh_var1 | 6439 | I32 |
| arh_var1 | 6440 | M32 |
| arh_var1 | 6441 | Q32 |
| arh_var1 | 6442 | U32 |
| arh_var1 | 6443 | Y32 |

| | | |
|---|---|---|
| arh_var1 | 6444 | AC32 |
| arh_var1 | 6445 | A36 |
| arh_var1 | 6446 | E36 |
| arh_var1 | 6447 | I36 |
| arh_var1 | 6448 | M36 |
| arh_var1 | 6449 | Q36 |
| arh_var1 | 6450 | U36 |
| arh_var1 | 6451 | Y36 |
| arh_var1 | 6452 | AC36 |
| arh_var1 | 6453 | A40 |
| arh_var1 | 6454 | E40 |
| arh_var1 | 6455 | I40 |
| arh_var1 | 6456 | M40 |
| arh_var1 | 6457 | Q40 |
| arh_var1 | 6458 | U40 |
| arh_var1 | 6459 | Y40 |
| arh_var1 | 6460 | AC40 |
| arh_var1 | 6461 | A44 |
| arh_var1 | 6462 | E44 |
| arh_var1 | 6463 | I44 |
| arh_var1 | 6464 | M44 |
| arh_var1 | 6465 | Q44 |
| arh_var1 | 6466 | U44 |
| arh_var1 | 6467 | Y44 |
| arh_var1 | 6468 | AC44 |
| arh_var1 | 6469 | A48 |
| arh_var1 | 6470 | E48 |
| arh_var1 | 6471 | I48 |
| arh_var1 | 6472 | M48 |
| arh_var1 | 6473 | Q48 |
| arh_var1 | 6474 | U48 |
| arh_var1 | 6475 | Y48 |
| arh_var1 | 6476 | AC48 |
| arh_var1 | 6477 | C02 |
| arh_var1 | 6478 | G02 |
| arh_var1 | 6479 | K02 |
| arh_var1 | 6480 | O02 |
| arh_var1 | 6481 | S02 |
| arh_var1 | 6482 | W02 |
| arh_var1 | 6483 | AA02 |
| arh_var1 | 6484 | AE02 |
| arh_var1 | 6485 | C06 |
| arh_var1 | 6486 | G06 |
| arh_var1 | 6487 | K06 |
| arh_var1 | 6488 | O06 |
| arh_var1 | 6489 | S06 |
| arh_var1 | 6490 | W06 |
| arh_var1 | 6491 | AA06 |
| arh_var1 | 6492 | AE06 |
| arh_var1 | 6493 | C10 |

| | | |
|---|---|---|
| arh_var1 | 6494 | G10 |
| arh_var1 | 6495 | K10 |
| arh_var1 | 6496 | O10 |
| arh_var1 | 6497 | S10 |
| arh_var1 | 6498 | W10 |
| arh_var1 | 6499 | AA10 |
| arh_var1 | 6500 | AE10 |
| arh_var1 | 6501 | C14 |
| arh_var1 | 6502 | G14 |
| arh_var1 | 6503 | K14 |
| arh_var1 | 6504 | O14 |
| arh_var1 | 6505 | S14 |
| arh_var1 | 6506 | W14 |
| arh_var1 | 6507 | AA14 |
| arh_var1 | 6508 | AE14 |
| arh_var1 | 6509 | C18 |
| arh_var1 | 6510 | G18 |
| arh_var1 | 6511 | K18 |
| arh_var1 | 6512 | O18 |
| arh_var1 | 6513 | S18 |
| arh_var1 | 6514 | W18 |
| arh_var1 | 6515 | AA18 |
| arh_var1 | 6516 | AE18 |
| arh_var1 | 6517 | C22 |
| arh_var1 | 6518 | G22 |
| arh_var1 | 6519 | K22 |
| arh_var1 | 6520 | O22 |
| arh_var1 | 6521 | S22 |
| arh_var1 | 6522 | W22 |
| arh_var1 | 6523 | AA22 |
| arh_var1 | 6524 | AE22 |
| arh_var1 | 6525 | C26 |
| arh_var1 | 6526 | G26 |
| arh_var1 | 6527 | K26 |
| arh_var1 | 6528 | O26 |
| arh_var1 | 6529 | S26 |
| arh_var1 | 6530 | W26 |
| arh_var1 | 6531 | AA26 |
| arh_var1 | 6532 | AE26 |
| arh_var1 | 6533 | C30 |
| arh_var1 | 6534 | G30 |
| arh_var1 | 6535 | K30 |
| arh_var1 | 6536 | O30 |
| arh_var1 | 6537 | S30 |
| arh_var1 | 6538 | W30 |
| arh_var1 | 6539 | AA30 |
| arh_var1 | 6540 | AE30 |
| arh_var1 | 6541 | C34 |
| arh_var1 | 6542 | G34 |
| arh_var1 | 6543 | K34 |

| | | |
|---|---|---|
| arh_var1 | 6544 | O34 |
| arh_var1 | 6545 | S34 |
| arh_var1 | 6546 | W34 |
| arh_var1 | 6547 | AA34 |
| arh_var1 | 6548 | AE34 |
| arh_var1 | 6549 | C38 |
| arh_var1 | 6550 | G38 |
| arh_var1 | 6551 | K38 |
| arh_var1 | 6552 | O38 |
| arh_var1 | 6553 | S38 |
| arh_var1 | 6554 | W38 |
| arh_var1 | 6555 | AA38 |
| arh_var1 | 6556 | AE38 |
| arh_var1 | 6557 | C42 |
| arh_var1 | 6558 | G42 |
| arh_var1 | 6559 | K42 |
| arh_var1 | 6560 | O42 |
| arh_var1 | 6561 | S42 |
| arh_var1 | 6562 | W42 |
| arh_var1 | 6563 | AA42 |
| arh_var1 | 6564 | AE42 |
| arh_var1 | 6565 | C46 |
| arh_var1 | 6566 | G46 |
| arh_var1 | 6567 | K46 |
| arh_var1 | 6568 | O46 |
| arh_var1 | 6569 | S46 |
| arh_var1 | 6570 | W46 |
| arh_var1 | 6571 | AA46 |
| arh_var1 | 6572 | AE46 |
| arh_var1 | 6573 | C04 |
| arh_var1 | 6574 | G04 |
| arh_var1 | 6575 | K04 |
| arh_var1 | 6576 | O04 |
| arh_var1 | 6577 | S04 |
| arh_var1 | 6578 | W04 |
| arh_var1 | 6579 | AA04 |
| arh_var1 | 6580 | AE04 |
| arh_var1 | 6581 | C08 |
| arh_var1 | 6582 | G08 |
| arh_var1 | 6583 | K08 |
| arh_var1 | 6584 | O08 |
| arh_var1 | 6585 | S08 |
| arh_var1 | 6586 | W08 |
| arh_var1 | 6587 | AA08 |
| arh_var1 | 6588 | AE08 |
| arh_var1 | 6589 | C12 |
| arh_var1 | 6590 | G12 |
| arh_var1 | 6591 | K12 |
| arh_var1 | 6592 | O12 |
| arh_var1 | 6593 | S12 |

| | | |
|---|---|---|
| arh_var1 | 6594 | W12 |
| arh_var1 | 6595 | AA12 |
| arh_var1 | 6596 | AE12 |
| arh_var1 | 6597 | C16 |
| arh_var1 | 6598 | G16 |
| arh_var1 | 6599 | K16 |
| arh_var1 | 6600 | O16 |
| arh_var1 | 6601 | S16 |
| arh_var1 | 6602 | W16 |
| arh_var1 | 6603 | AA16 |
| arh_var1 | 6604 | AE16 |
| arh_var1 | 6605 | C20 |
| arh_var1 | 6606 | G20 |
| arh_var1 | 6607 | K20 |
| arh_var1 | 6608 | O20 |
| arh_var1 | 6609 | S20 |
| arh_var1 | 6610 | W20 |
| arh_var1 | 6611 | AA20 |
| arh_var1 | 6612 | AE20 |
| arh_var1 | 6613 | C24 |
| arh_var1 | 6614 | G24 |
| arh_var1 | 6615 | K24 |
| arh_var1 | 6616 | O24 |
| arh_var1 | 6617 | S24 |
| arh_var1 | 6618 | W24 |
| arh_var1 | 6619 | AA24 |
| arh_var1 | 6620 | AE24 |
| arh_var1 | 6621 | C28 |
| arh_var1 | 6622 | G28 |
| arh_var1 | 6623 | K28 |
| arh_var1 | 6624 | O28 |
| arh_var1 | 6625 | S28 |
| arh_var1 | 2815 | W28 |
| arh_var1 | 2816 | AA28 |
| arh_var1 | 2817 | AE28 |
| arh_var1 | 2820 | C32 |
| arh_var1 | 2821 | G32 |
| arh_var1 | 2822 | K32 |
| arh_var1 | 3245 | O32 |
| arh_var1 | 3246 | S32 |
| arh_var1 | 3247 | W32 |
| arh_var1 | 3472 | AA32 |
| arh_var1 | 3473 | AE32 |
| arh_var1 | 3474 | C36 |
| arh_var1 | 3327 | G36 |
| arh_var1 | 3583 | K36 |
| arh_var1 | 3584 | O36 |
| arh_var1 | 3601 | S36 |
| arh_var1 | 3602 | W36 |
| arh_var1 | 3603 | AA36 |

| | | |
|---|---|---|
| arh_var1 | 3775 | AE36 |
| arh_var1 | 3776 | C40 |
| arh_var1 | 3777 | G40 |
| arh_var1 | 1808 | K40 |
| arh_var1 | 1809 | O40 |
| arh_var1 | 1812 | S40 |
| arh_var1 | 1879 | W40 |
| arh_var1 | 1880 | AA40 |
| arh_var1 | 1881 | AE40 |
| arh_var1 | 2360 | C44 |
| arh_var1 | 2361 | G44 |
| arh_var1 | 2362 | K44 |
| arh_var1 | 2492 | O44 |
| arh_var1 | 2493 | S44 |
| arh_var1 | 2494 | W44 |
| arh_var1 | 2579 | AA44 |
| arh_var1 | 2580 | AE44 |
| arh_var1 | 2581 | C48 |
| arh_var1 | 2602 | G48 |
| arh_var1 | 2603 | K48 |
| arh_var1 | 2604 | O48 |
| arh_var1 | 2846 | S48 |
| arh_var1 | 2847 | W48 |
| arh_var1 | 2848 | AA48 |
| arh_var1 | NTC | AE48 |
| clasp2_var | 6284 | B01 |
| clasp2_var | 6285 | F01 |
| clasp2_var | 6286 | J01 |
| clasp2_var | 6287 | N01 |
| clasp2_var | 6288 | R01 |
| clasp2_var | 6289 | V01 |
| clasp2_var | 6290 | Z01 |
| clasp2_var | 6291 | AD01 |
| clasp2_var | 6292 | B05 |
| clasp2_var | 6293 | F05 |
| clasp2_var | 6294 | J05 |
| clasp2_var | 6295 | N05 |
| clasp2_var | 6296 | R05 |
| clasp2_var | 6297 | V05 |
| clasp2_var | 6298 | Z05 |
| clasp2_var | 6299 | AD05 |
| clasp2_var | 6300 | B09 |
| clasp2_var | 6301 | F09 |
| clasp2_var | 6302 | J09 |
| clasp2_var | 6303 | N09 |
| clasp2_var | 6304 | R09 |
| clasp2_var | 6305 | V09 |
| clasp2_var | 6306 | Z09 |
| clasp2_var | 6307 | AD09 |
| clasp2_var | 6308 | B13 |

| | | |
|---|---|---|
| clasp2_var: | 6309 | F13 |
| clasp2_var: | 6310 | J13 |
| clasp2_var: | 6311 | N13 |
| clasp2_var: | 6312 | R13 |
| clasp2_var: | 6313 | V13 |
| clasp2_var: | 6314 | Z13 |
| clasp2_var: | 6315 | AD13 |
| clasp2_var: | 6316 | B17 |
| clasp2_var: | 6317 | F17 |
| clasp2_var: | 6318 | J17 |
| clasp2_var: | 6319 | N17 |
| clasp2_var: | 6320 | R17 |
| clasp2_var: | 6321 | V17 |
| clasp2_var: | 6322 | Z17 |
| clasp2_var: | 6323 | AD17 |
| clasp2_var: | 6324 | B21 |
| clasp2_var: | 6325 | F21 |
| clasp2_var: | 6326 | J21 |
| clasp2_var: | 6327 | N21 |
| clasp2_var: | 6328 | R21 |
| clasp2_var: | 6329 | V21 |
| clasp2_var: | 6330 | Z21 |
| clasp2_var: | 6331 | AD21 |
| clasp2_var: | 6332 | B25 |
| clasp2_var: | 6333 | F25 |
| clasp2_var: | 6334 | J25 |
| clasp2_var: | 6335 | N25 |
| clasp2_var: | 6336 | R25 |
| clasp2_var: | 6337 | V25 |
| clasp2_var: | 6338 | Z25 |
| clasp2_var: | 6339 | AD25 |
| clasp2_var: | 6340 | B29 |
| clasp2_var: | 6341 | F29 |
| clasp2_var: | 6342 | J29 |
| clasp2_var: | 6343 | N29 |
| clasp2_var: | 6344 | R29 |
| clasp2_var: | 6345 | V29 |
| clasp2_var: | 6346 | Z29 |
| clasp2_var: | 6347 | AD29 |
| clasp2_var: | 6348 | B33 |
| clasp2_var: | 6349 | F33 |
| clasp2_var: | 6350 | J33 |
| clasp2_var: | 6351 | N33 |
| clasp2_var: | 6352 | R33 |
| clasp2_var: | 6353 | V33 |
| clasp2_var: | 6354 | Z33 |
| clasp2_var: | 6355 | AD33 |
| clasp2_var: | 6356 | B37 |
| clasp2_var: | 6357 | F37 |
| clasp2_var: | 6358 | J37 |

| | | |
|---|---|---|
| clasp2_var: | 6359 | N37 |
| clasp2_var: | 6360 | R37 |
| clasp2_var: | 6361 | V37 |
| clasp2_var: | 6362 | Z37 |
| clasp2_var: | 6363 | AD37 |
| clasp2_var: | 6364 | B41 |
| clasp2_var: | 6365 | F41 |
| clasp2_var: | 6366 | J41 |
| clasp2_var: | 6367 | N41 |
| clasp2_var: | 6368 | R41 |
| clasp2_var: | 6369 | V41 |
| clasp2_var: | 6370 | Z41 |
| clasp2_var: | 6371 | AD41 |
| clasp2_var: | 6372 | B45 |
| clasp2_var: | 6373 | F45 |
| clasp2_var: | 6374 | J45 |
| clasp2_var: | 6375 | N45 |
| clasp2_var: | 6376 | R45 |
| clasp2_var: | 6377 | V45 |
| clasp2_var: | 6378 | Z45 |
| clasp2_var: | 6379 | AD45 |
| clasp2_var: | 6380 | B03 |
| clasp2_var: | 6381 | F03 |
| clasp2_var: | 6382 | J03 |
| clasp2_var: | 6383 | N03 |
| clasp2_var: | 6384 | R03 |
| clasp2_var: | 6385 | V03 |
| clasp2_var: | 6386 | Z03 |
| clasp2_var: | 6387 | AD03 |
| clasp2_var: | 6388 | B07 |
| clasp2_var: | 6389 | F07 |
| clasp2_var: | 6390 | J07 |
| clasp2_var: | 6391 | N07 |
| clasp2_var: | 6392 | R07 |
| clasp2_var: | 6393 | V07 |
| clasp2_var: | 6394 | Z07 |
| clasp2_var: | 6395 | AD07 |
| clasp2_var: | 6396 | B11 |
| clasp2_var: | 6397 | F11 |
| clasp2_var: | 6398 | J11 |
| clasp2_var: | 6399 | N11 |
| clasp2_var: | 6400 | R11 |
| clasp2_var: | 6401 | V11 |
| clasp2_var: | 6402 | Z11 |
| clasp2_var: | 6403 | AD11 |
| clasp2_var: | 6404 | B15 |
| clasp2_var: | 6405 | F15 |
| clasp2_var: | 6406 | J15 |
| clasp2_var: | 6407 | N15 |
| clasp2_var: | 6408 | R15 |

| | | |
|---|---|---|
| clasp2_var: | 6409 | V15 |
| clasp2_var: | 6410 | Z15 |
| clasp2_var: | 6411 | AD15 |
| clasp2_var: | 6412 | B19 |
| clasp2_var: | 6413 | F19 |
| clasp2_var: | 6414 | J19 |
| clasp2_var: | 6415 | N19 |
| clasp2_var: | 6416 | R19 |
| clasp2_var: | 6417 | V19 |
| clasp2_var: | 6418 | Z19 |
| clasp2_var: | 6419 | AD19 |
| clasp2_var: | 6421 | B23 |
| clasp2_var: | 6422 | F23 |
| clasp2_var: | 6423 | J23 |
| clasp2_var: | 6424 | N23 |
| clasp2_var: | 6425 | R23 |
| clasp2_var: | 6426 | V23 |
| clasp2_var: | 6427 | Z23 |
| clasp2_var: | 6428 | AD23 |
| clasp2_var: | 6429 | B27 |
| clasp2_var: | 6430 | F27 |
| clasp2_var: | 6431 | J27 |
| clasp2_var: | 6432 | N27 |
| clasp2_var: | 6433 | R27 |
| clasp2_var: | 6434 | V27 |
| clasp2_var: | 6435 | Z27 |
| clasp2_var: | 6436 | AD27 |
| clasp2_var: | 6437 | B31 |
| clasp2_var: | 6438 | F31 |
| clasp2_var: | 6439 | J31 |
| clasp2_var: | 6440 | N31 |
| clasp2_var: | 6441 | R31 |
| clasp2_var: | 6442 | V31 |
| clasp2_var: | 6443 | Z31 |
| clasp2_var: | 6444 | AD31 |
| clasp2_var: | 6445 | B35 |
| clasp2_var: | 6446 | F35 |
| clasp2_var: | 6447 | J35 |
| clasp2_var: | 6448 | N35 |
| clasp2_var: | 6449 | R35 |
| clasp2_var: | 6450 | V35 |
| clasp2_var: | 6451 | Z35 |
| clasp2_var: | 6452 | AD35 |
| clasp2_var: | 6453 | B39 |
| clasp2_var: | 6454 | F39 |
| clasp2_var: | 6455 | J39 |
| clasp2_var: | 6456 | N39 |
| clasp2_var: | 6457 | R39 |
| clasp2_var: | 6458 | V39 |
| clasp2_var: | 6459 | Z39 |

| | | |
|---|---|---|
| clasp2_var: | 6460 | AD39 |
| clasp2_var: | 6461 | B43 |
| clasp2_var: | 6462 | F43 |
| clasp2_var: | 6463 | J43 |
| clasp2_var: | 6464 | N43 |
| clasp2_var: | 6465 | R43 |
| clasp2_var: | 6466 | V43 |
| clasp2_var: | 6467 | Z43 |
| clasp2_var: | 6468 | AD43 |
| clasp2_var: | 6469 | B47 |
| clasp2_var: | 6470 | F47 |
| clasp2_var: | 6471 | J47 |
| clasp2_var: | 6472 | N47 |
| clasp2_var: | 6473 | R47 |
| clasp2_var: | 6474 | V47 |
| clasp2_var: | 6475 | Z47 |
| clasp2_var: | 6476 | AD47 |
| clasp2_var: | 6477 | D01 |
| clasp2_var: | 6478 | H01 |
| clasp2_var: | 6479 | L01 |
| clasp2_var: | 6480 | P01 |
| clasp2_var: | 6481 | T01 |
| clasp2_var: | 6482 | X01 |
| clasp2_var: | 6483 | AB01 |
| clasp2_var: | 6484 | AF01 |
| clasp2_var: | 6485 | D05 |
| clasp2_var: | 6486 | H05 |
| clasp2_var: | 6487 | L05 |
| clasp2_var: | 6488 | P05 |
| clasp2_var: | 6489 | T05 |
| clasp2_var: | 6490 | X05 |
| clasp2_var: | 6491 | AB05 |
| clasp2_var: | 6492 | AF05 |
| clasp2_var: | 6493 | D09 |
| clasp2_var: | 6494 | H09 |
| clasp2_var: | 6495 | L09 |
| clasp2_var: | 6496 | P09 |
| clasp2_var: | 6497 | T09 |
| clasp2_var: | 6498 | X09 |
| clasp2_var: | 6499 | AB09 |
| clasp2_var: | 6500 | AF09 |
| clasp2_var: | 6501 | D13 |
| clasp2_var: | 6502 | H13 |
| clasp2_var: | 6503 | L13 |
| clasp2_var: | 6504 | P13 |
| clasp2_var: | 6505 | T13 |
| clasp2_var: | 6506 | X13 |
| clasp2_var: | 6507 | AB13 |
| clasp2_var: | 6508 | AF13 |
| clasp2_var: | 6509 | D17 |

| | | |
|---|---|---|
| clasp2_var: | 6510 | H17 |
| clasp2_var: | 6511 | L17 |
| clasp2_var: | 6512 | P17 |
| clasp2_var: | 6513 | T17 |
| clasp2_var: | 6514 | X17 |
| clasp2_var: | 6515 | AB17 |
| clasp2_var: | 6516 | AF17 |
| clasp2_var: | 6517 | D21 |
| clasp2_var: | 6518 | H21 |
| clasp2_var: | 6519 | L21 |
| clasp2_var: | 6520 | P21 |
| clasp2_var: | 6521 | T21 |
| clasp2_var: | 6522 | X21 |
| clasp2_var: | 6523 | AB21 |
| clasp2_var: | 6524 | AF21 |
| clasp2_var: | 6525 | D25 |
| clasp2_var: | 6526 | H25 |
| clasp2_var: | 6527 | L25 |
| clasp2_var: | 6528 | P25 |
| clasp2_var: | 6529 | T25 |
| clasp2_var: | 6530 | X25 |
| clasp2_var: | 6531 | AB25 |
| clasp2_var: | 6532 | AF25 |
| clasp2_var: | 6533 | D29 |
| clasp2_var: | 6534 | H29 |
| clasp2_var: | 6535 | L29 |
| clasp2_var: | 6536 | P29 |
| clasp2_var: | 6537 | T29 |
| clasp2_var: | 6538 | X29 |
| clasp2_var: | 6539 | AB29 |
| clasp2_var: | 6540 | AF29 |
| clasp2_var: | 6541 | D33 |
| clasp2_var: | 6542 | H33 |
| clasp2_var: | 6543 | L33 |
| clasp2_var: | 6544 | P33 |
| clasp2_var: | 6545 | T33 |
| clasp2_var: | 6546 | X33 |
| clasp2_var: | 6547 | AB33 |
| clasp2_var: | 6548 | AF33 |
| clasp2_var: | 6549 | D37 |
| clasp2_var: | 6550 | H37 |
| clasp2_var: | 6551 | L37 |
| clasp2_var: | 6552 | P37 |
| clasp2_var: | 6553 | T37 |
| clasp2_var: | 6554 | X37 |
| clasp2_var: | 6555 | AB37 |
| clasp2_var: | 6556 | AF37 |
| clasp2_var: | 6557 | D41 |
| clasp2_var: | 6558 | H41 |
| clasp2_var: | 6559 | L41 |

| | | |
|---|---|---|
| clasp2_var: | 6560 | P41 |
| clasp2_var: | 6561 | T41 |
| clasp2_var: | 6562 | X41 |
| clasp2_var: | 6563 | AB41 |
| clasp2_var: | 6564 | AF41 |
| clasp2_var: | 6565 | D45 |
| clasp2_var: | 6566 | H45 |
| clasp2_var: | 6567 | L45 |
| clasp2_var: | 6568 | P45 |
| clasp2_var: | 6569 | T45 |
| clasp2_var: | 6570 | X45 |
| clasp2_var: | 6571 | AB45 |
| clasp2_var: | 6572 | AF45 |
| clasp2_var: | 6573 | D03 |
| clasp2_var: | 6574 | H03 |
| clasp2_var: | 6575 | L03 |
| clasp2_var: | 6576 | P03 |
| clasp2_var: | 6577 | T03 |
| clasp2_var: | 6578 | X03 |
| clasp2_var: | 6579 | AB03 |
| clasp2_var: | 6580 | AF03 |
| clasp2_var: | 6581 | D07 |
| clasp2_var: | 6582 | H07 |
| clasp2_var: | 6583 | L07 |
| clasp2_var: | 6584 | P07 |
| clasp2_var: | 6585 | T07 |
| clasp2_var: | 6586 | X07 |
| clasp2_var: | 6587 | AB07 |
| clasp2_var: | 6588 | AF07 |
| clasp2_var: | 6589 | D11 |
| clasp2_var: | 6590 | H11 |
| clasp2_var: | 6591 | L11 |
| clasp2_var: | 6592 | P11 |
| clasp2_var: | 6593 | T11 |
| clasp2_var: | 6594 | X11 |
| clasp2_var: | 6595 | AB11 |
| clasp2_var: | 6596 | AF11 |
| clasp2_var: | 6597 | D15 |
| clasp2_var: | 6598 | H15 |
| clasp2_var: | 6599 | L15 |
| clasp2_var: | 6600 | P15 |
| clasp2_var: | 6601 | T15 |
| clasp2_var: | 6602 | X15 |
| clasp2_var: | 6603 | AB15 |
| clasp2_var: | 6604 | AF15 |
| clasp2_var: | 6605 | D19 |
| clasp2_var: | 6606 | H19 |
| clasp2_var: | 6607 | L19 |
| clasp2_var: | 6608 | P19 |
| clasp2_var: | 6609 | T19 |

| | | |
|---|---|---|
| clasp2_var: | 6610 | X19 |
| clasp2_var: | 6611 | AB19 |
| clasp2_var: | 6612 | AF19 |
| clasp2_var: | 6613 | D23 |
| clasp2_var: | 6614 | H23 |
| clasp2_var: | 6615 | L23 |
| clasp2_var: | 6616 | P23 |
| clasp2_var: | 6617 | T23 |
| clasp2_var: | 6618 | X23 |
| clasp2_var: | 6619 | AB23 |
| clasp2_var: | 6620 | AF23 |
| clasp2_var: | 6621 | D27 |
| clasp2_var: | 6622 | H27 |
| clasp2_var: | 6623 | L27 |
| clasp2_var: | 6624 | P27 |
| clasp2_var: | 6625 | T27 |
| clasp2_var: | 2815 | X27 |
| clasp2_var: | 2816 | AB27 |
| clasp2_var: | 2817 | AF27 |
| clasp2_var: | 2820 | D31 |
| clasp2_var: | 2821 | H31 |
| clasp2_var: | 2822 | L31 |
| clasp2_var: | 3245 | P31 |
| clasp2_var: | 3246 | T31 |
| clasp2_var: | 3247 | X31 |
| clasp2_var: | 3472 | AB31 |
| clasp2_var: | 3473 | AF31 |
| clasp2_var: | 3474 | D35 |
| clasp2_var: | 3327 | H35 |
| clasp2_var: | 3583 | L35 |
| clasp2_var: | 3584 | P35 |
| clasp2_var: | 3601 | T35 |
| clasp2_var: | 3602 | X35 |
| clasp2_var: | 3603 | AB35 |
| clasp2_var: | 3775 | AF35 |
| clasp2_var: | 3776 | D39 |
| clasp2_var: | 3777 | H39 |
| clasp2_var: | 1808 | L39 |
| clasp2_var: | 1809 | P39 |
| clasp2_var: | 1812 | T39 |
| clasp2_var: | 1879 | X39 |
| clasp2_var: | 1880 | AB39 |
| clasp2_var: | 1881 | AF39 |
| clasp2_var: | 2360 | D43 |
| clasp2_var: | 2361 | H43 |
| clasp2_var: | 2362 | L43 |
| clasp2_var: | 2492 | P43 |
| clasp2_var: | 2493 | T43 |
| clasp2_var: | 2494 | X43 |
| clasp2_var: | 2579 | AB43 |

| | | |
|---|---|---|
| clasp2_var: | 2580 | AF43 |
| clasp2_var: | 2581 | D47 |
| clasp2_var: | 2602 | H47 |
| clasp2_var: | 2603 | L47 |
| clasp2_var: | 2604 | P47 |
| clasp2_var: | 2846 | T47 |
| clasp2_var: | 2847 | X47 |
| clasp2_var: | 2848 | AB47 |
| clasp2_var: | NTC | AF47 |
| col18_var1 | 6284 | B02 |
| col18_var1 | 6285 | F02 |
| col18_var1 | 6286 | J02 |
| col18_var1 | 6287 | N02 |
| col18_var1 | 6288 | R02 |
| col18_var1 | 6289 | V02 |
| col18_var1 | 6290 | Z02 |
| col18_var1 | 6291 | AD02 |
| col18_var1 | 6292 | B06 |
| col18_var1 | 6293 | F06 |
| col18_var1 | 6294 | J06 |
| col18_var1 | 6295 | N06 |
| col18_var1 | 6296 | R06 |
| col18_var1 | 6297 | V06 |
| col18_var1 | 6298 | Z06 |
| col18_var1 | 6299 | AD06 |
| col18_var1 | 6300 | B10 |
| col18_var1 | 6301 | F10 |
| col18_var1 | 6302 | J10 |
| col18_var1 | 6303 | N10 |
| col18_var1 | 6304 | R10 |
| col18_var1 | 6305 | V10 |
| col18_var1 | 6306 | Z10 |
| col18_var1 | 6307 | AD10 |
| col18_var1 | 6308 | B14 |
| col18_var1 | 6309 | F14 |
| col18_var1 | 6310 | J14 |
| col18_var1 | 6311 | N14 |
| col18_var1 | 6312 | R14 |
| col18_var1 | 6313 | V14 |
| col18_var1 | 6314 | Z14 |
| col18_var1 | 6315 | AD14 |
| col18_var1 | 6316 | B18 |
| col18_var1 | 6317 | F18 |
| col18_var1 | 6318 | J18 |
| col18_var1 | 6319 | N18 |
| col18_var1 | 6320 | R18 |
| col18_var1 | 6321 | V18 |
| col18_var1 | 6322 | Z18 |
| col18_var1 | 6323 | AD18 |
| col18_var1 | 6324 | B22 |

| | | |
|---|---|---|
| col18_var1 | 6325 | F22 |
| col18_var1 | 6326 | J22 |
| col18_var1 | 6327 | N22 |
| col18_var1 | 6328 | R22 |
| col18_var1 | 6329 | V22 |
| col18_var1 | 6330 | Z22 |
| col18_var1 | 6331 | AD22 |
| col18_var1 | 6332 | B26 |
| col18_var1 | 6333 | F26 |
| col18_var1 | 6334 | J26 |
| col18_var1 | 6335 | N26 |
| col18_var1 | 6336 | R26 |
| col18_var1 | 6337 | V26 |
| col18_var1 | 6338 | Z26 |
| col18_var1 | 6339 | AD26 |
| col18_var1 | 6340 | B30 |
| col18_var1 | 6341 | F30 |
| col18_var1 | 6342 | J30 |
| col18_var1 | 6343 | N30 |
| col18_var1 | 6344 | R30 |
| col18_var1 | 6345 | V30 |
| col18_var1 | 6346 | Z30 |
| col18_var1 | 6347 | AD30 |
| col18_var1 | 6348 | B34 |
| col18_var1 | 6349 | F34 |
| col18_var1 | 6350 | J34 |
| col18_var1 | 6351 | N34 |
| col18_var1 | 6352 | R34 |
| col18_var1 | 6353 | V34 |
| col18_var1 | 6354 | Z34 |
| col18_var1 | 6355 | AD34 |
| col18_var1 | 6356 | B38 |
| col18_var1 | 6357 | F38 |
| col18_var1 | 6358 | J38 |
| col18_var1 | 6359 | N38 |
| col18_var1 | 6360 | R38 |
| col18_var1 | 6361 | V38 |
| col18_var1 | 6362 | Z38 |
| col18_var1 | 6363 | AD38 |
| col18_var1 | 6364 | B42 |
| col18_var1 | 6365 | F42 |
| col18_var1 | 6366 | J42 |
| col18_var1 | 6367 | N42 |
| col18_var1 | 6368 | R42 |
| col18_var1 | 6369 | V42 |
| col18_var1 | 6370 | Z42 |
| col18_var1 | 6371 | AD42 |
| col18_var1 | 6372 | B46 |
| col18_var1 | 6373 | F46 |
| col18_var1 | 6374 | J46 |

| | | |
|---|---|---|
| col18_var1 | 6375 | N46 |
| col18_var1 | 6376 | R46 |
| col18_var1 | 6377 | V46 |
| col18_var1 | 6378 | Z46 |
| col18_var1 | 6379 | AD46 |
| col18_var1 | 6380 | B04 |
| col18_var1 | 6381 | F04 |
| col18_var1 | 6382 | J04 |
| col18_var1 | 6383 | N04 |
| col18_var1 | 6384 | R04 |
| col18_var1 | 6385 | V04 |
| col18_var1 | 6386 | Z04 |
| col18_var1 | 6387 | AD04 |
| col18_var1 | 6388 | B08 |
| col18_var1 | 6389 | F08 |
| col18_var1 | 6390 | J08 |
| col18_var1 | 6391 | N08 |
| col18_var1 | 6392 | R08 |
| col18_var1 | 6393 | V08 |
| col18_var1 | 6394 | Z08 |
| col18_var1 | 6395 | AD08 |
| col18_var1 | 6396 | B12 |
| col18_var1 | 6397 | F12 |
| col18_var1 | 6398 | J12 |
| col18_var1 | 6399 | N12 |
| col18_var1 | 6400 | R12 |
| col18_var1 | 6401 | V12 |
| col18_var1 | 6402 | Z12 |
| col18_var1 | 6403 | AD12 |
| col18_var1 | 6404 | B16 |
| col18_var1 | 6405 | F16 |
| col18_var1 | 6406 | J16 |
| col18_var1 | 6407 | N16 |
| col18_var1 | 6408 | R16 |
| col18_var1 | 6409 | V16 |
| col18_var1 | 6410 | Z16 |
| col18_var1 | 6411 | AD16 |
| col18_var1 | 6412 | B20 |
| col18_var1 | 6413 | F20 |
| col18_var1 | 6414 | J20 |
| col18_var1 | 6415 | N20 |
| col18_var1 | 6416 | R20 |
| col18_var1 | 6417 | V20 |
| col18_var1 | 6418 | Z20 |
| col18_var1 | 6419 | AD20 |
| col18_var1 | 6421 | B24 |
| col18_var1 | 6422 | F24 |
| col18_var1 | 6423 | J24 |
| col18_var1 | 6424 | N24 |
| col18_var1 | 6425 | R24 |

| | | |
|---|---|---|
| col18_var1 | 6426 | V24 |
| col18_var1 | 6427 | Z24 |
| col18_var1 | 6428 | AD24 |
| col18_var1 | 6429 | B28 |
| col18_var1 | 6430 | F28 |
| col18_var1 | 6431 | J28 |
| col18_var1 | 6432 | N28 |
| col18_var1 | 6433 | R28 |
| col18_var1 | 6434 | V28 |
| col18_var1 | 6435 | Z28 |
| col18_var1 | 6436 | AD28 |
| col18_var1 | 6437 | B32 |
| col18_var1 | 6438 | F32 |
| col18_var1 | 6439 | J32 |
| col18_var1 | 6440 | N32 |
| col18_var1 | 6441 | R32 |
| col18_var1 | 6442 | V32 |
| col18_var1 | 6443 | Z32 |
| col18_var1 | 6444 | AD32 |
| col18_var1 | 6445 | B36 |
| col18_var1 | 6446 | F36 |
| col18_var1 | 6447 | J36 |
| col18_var1 | 6448 | N36 |
| col18_var1 | 6449 | R36 |
| col18_var1 | 6450 | V36 |
| col18_var1 | 6451 | Z36 |
| col18_var1 | 6452 | AD36 |
| col18_var1 | 6453 | B40 |
| col18_var1 | 6454 | F40 |
| col18_var1 | 6455 | J40 |
| col18_var1 | 6456 | N40 |
| col18_var1 | 6457 | R40 |
| col18_var1 | 6458 | V40 |
| col18_var1 | 6459 | Z40 |
| col18_var1 | 6460 | AD40 |
| col18_var1 | 6461 | B44 |
| col18_var1 | 6462 | F44 |
| col18_var1 | 6463 | J44 |
| col18_var1 | 6464 | N44 |
| col18_var1 | 6465 | R44 |
| col18_var1 | 6466 | V44 |
| col18_var1 | 6467 | Z44 |
| col18_var1 | 6468 | AD44 |
| col18_var1 | 6469 | B48 |
| col18_var1 | 6470 | F48 |
| col18_var1 | 6471 | J48 |
| col18_var1 | 6472 | N48 |
| col18_var1 | 6473 | R48 |
| col18_var1 | 6474 | V48 |
| col18_var1 | 6475 | Z48 |

| | | |
|---|---|---|
| col18_var1 | 6476 | AD48 |
| col18_var1 | 6477 | D02 |
| col18_var1 | 6478 | H02 |
| col18_var1 | 6479 | L02 |
| col18_var1 | 6480 | P02 |
| col18_var1 | 6481 | T02 |
| col18_var1 | 6482 | X02 |
| col18_var1 | 6483 | AB02 |
| col18_var1 | 6484 | AF02 |
| col18_var1 | 6485 | D06 |
| col18_var1 | 6486 | H06 |
| col18_var1 | 6487 | L06 |
| col18_var1 | 6488 | P06 |
| col18_var1 | 6489 | T06 |
| col18_var1 | 6490 | X06 |
| col18_var1 | 6491 | AB06 |
| col18_var1 | 6492 | AF06 |
| col18_var1 | 6493 | D10 |
| col18_var1 | 6494 | H10 |
| col18_var1 | 6495 | L10 |
| col18_var1 | 6496 | P10 |
| col18_var1 | 6497 | T10 |
| col18_var1 | 6498 | X10 |
| col18_var1 | 6499 | AB10 |
| col18_var1 | 6500 | AF10 |
| col18_var1 | 6501 | D14 |
| col18_var1 | 6502 | H14 |
| col18_var1 | 6503 | L14 |
| col18_var1 | 6504 | P14 |
| col18_var1 | 6505 | T14 |
| col18_var1 | 6506 | X14 |
| col18_var1 | 6507 | AB14 |
| col18_var1 | 6508 | AF14 |
| col18_var1 | 6509 | D18 |
| col18_var1 | 6510 | H18 |
| col18_var1 | 6511 | L18 |
| col18_var1 | 6512 | P18 |
| col18_var1 | 6513 | T18 |
| col18_var1 | 6514 | X18 |
| col18_var1 | 6515 | AB18 |
| col18_var1 | 6516 | AF18 |
| col18_var1 | 6517 | D22 |
| col18_var1 | 6518 | H22 |
| col18_var1 | 6519 | L22 |
| col18_var1 | 6520 | P22 |
| col18_var1 | 6521 | T22 |
| col18_var1 | 6522 | X22 |
| col18_var1 | 6523 | AB22 |
| col18_var1 | 6524 | AF22 |
| col18_var1 | 6525 | D26 |

| | | |
|---|---|---|
| col18_var1 | 6526 | H26 |
| col18_var1 | 6527 | L26 |
| col18_var1 | 6528 | P26 |
| col18_var1 | 6529 | T26 |
| col18_var1 | 6530 | X26 |
| col18_var1 | 6531 | AB26 |
| col18_var1 | 6532 | AF26 |
| col18_var1 | 6533 | D30 |
| col18_var1 | 6534 | H30 |
| col18_var1 | 6535 | L30 |
| col18_var1 | 6536 | P30 |
| col18_var1 | 6537 | T30 |
| col18_var1 | 6538 | X30 |
| col18_var1 | 6539 | AB30 |
| col18_var1 | 6540 | AF30 |
| col18_var1 | 6541 | D34 |
| col18_var1 | 6542 | H34 |
| col18_var1 | 6543 | L34 |
| col18_var1 | 6544 | P34 |
| col18_var1 | 6545 | T34 |
| col18_var1 | 6546 | X34 |
| col18_var1 | 6547 | AB34 |
| col18_var1 | 6548 | AF34 |
| col18_var1 | 6549 | D38 |
| col18_var1 | 6550 | H38 |
| col18_var1 | 6551 | L38 |
| col18_var1 | 6552 | P38 |
| col18_var1 | 6553 | T38 |
| col18_var1 | 6554 | X38 |
| col18_var1 | 6555 | AB38 |
| col18_var1 | 6556 | AF38 |
| col18_var1 | 6557 | D42 |
| col18_var1 | 6558 | H42 |
| col18_var1 | 6559 | L42 |
| col18_var1 | 6560 | P42 |
| col18_var1 | 6561 | T42 |
| col18_var1 | 6562 | X42 |
| col18_var1 | 6563 | AB42 |
| col18_var1 | 6564 | AF42 |
| col18_var1 | 6565 | D46 |
| col18_var1 | 6566 | H46 |
| col18_var1 | 6567 | L46 |
| col18_var1 | 6568 | P46 |
| col18_var1 | 6569 | T46 |
| col18_var1 | 6570 | X46 |
| col18_var1 | 6571 | AB46 |
| col18_var1 | 6572 | AF46 |
| col18_var1 | 6573 | D04 |
| col18_var1 | 6574 | H04 |
| col18_var1 | 6575 | L04 |

| | | |
|---|---|---|
| col18_var1 | 6576 | P04 |
| col18_var1 | 6577 | T04 |
| col18_var1 | 6578 | X04 |
| col18_var1 | 6579 | AB04 |
| col18_var1 | 6580 | AF04 |
| col18_var1 | 6581 | D08 |
| col18_var1 | 6582 | H08 |
| col18_var1 | 6583 | L08 |
| col18_var1 | 6584 | P08 |
| col18_var1 | 6585 | T08 |
| col18_var1 | 6586 | X08 |
| col18_var1 | 6587 | AB08 |
| col18_var1 | 6588 | AF08 |
| col18_var1 | 6589 | D12 |
| col18_var1 | 6590 | H12 |
| col18_var1 | 6591 | L12 |
| col18_var1 | 6592 | P12 |
| col18_var1 | 6593 | T12 |
| col18_var1 | 6594 | X12 |
| col18_var1 | 6595 | AB12 |
| col18_var1 | 6596 | AF12 |
| col18_var1 | 6597 | D16 |
| col18_var1 | 6598 | H16 |
| col18_var1 | 6599 | L16 |
| col18_var1 | 6600 | P16 |
| col18_var1 | 6601 | T16 |
| col18_var1 | 6602 | X16 |
| col18_var1 | 6603 | AB16 |
| col18_var1 | 6604 | AF16 |
| col18_var1 | 6605 | D20 |
| col18_var1 | 6606 | H20 |
| col18_var1 | 6607 | L20 |
| col18_var1 | 6608 | P20 |
| col18_var1 | 6609 | T20 |
| col18_var1 | 6610 | X20 |
| col18_var1 | 6611 | AB20 |
| col18_var1 | 6612 | AF20 |
| col18_var1 | 6613 | D24 |
| col18_var1 | 6614 | H24 |
| col18_var1 | 6615 | L24 |
| col18_var1 | 6616 | P24 |
| col18_var1 | 6617 | T24 |
| col18_var1 | 6618 | X24 |
| col18_var1 | 6619 | AB24 |
| col18_var1 | 6620 | AF24 |
| col18_var1 | 6621 | D28 |
| col18_var1 | 6622 | H28 |
| col18_var1 | 6623 | L28 |
| col18_var1 | 6624 | P28 |
| col18_var1 | 6625 | T28 |

| | | |
|---|---|---|
| col18_var1 | 2815 | X28 |
| col18_var1 | 2816 | AB28 |
| col18_var1 | 2817 | AF28 |
| col18_var1 | 2820 | D32 |
| col18_var1 | 2821 | H32 |
| col18_var1 | 2822 | L32 |
| col18_var1 | 3245 | P32 |
| col18_var1 | 3246 | T32 |
| col18_var1 | 3247 | X32 |
| col18_var1 | 3472 | AB32 |
| col18_var1 | 3473 | AF32 |
| col18_var1 | 3474 | D36 |
| col18_var1 | 3327 | H36 |
| col18_var1 | 3583 | L36 |
| col18_var1 | 3584 | P36 |
| col18_var1 | 3601 | T36 |
| col18_var1 | 3602 | X36 |
| col18_var1 | 3603 | AB36 |
| col18_var1 | 3775 | AF36 |
| col18_var1 | 3776 | D40 |
| col18_var1 | 3777 | H40 |
| col18_var1 | 1808 | L40 |
| col18_var1 | 1809 | P40 |
| col18_var1 | 1812 | T40 |
| col18_var1 | 1879 | X40 |
| col18_var1 | 1880 | AB40 |
| col18_var1 | 1881 | AF40 |
| col18_var1 | 2360 | D44 |
| col18_var1 | 2361 | H44 |
| col18_var1 | 2362 | L44 |
| col18_var1 | 2492 | P44 |
| col18_var1 | 2493 | T44 |
| col18_var1 | 2494 | X44 |
| col18_var1 | 2579 | AB44 |
| col18_var1 | 2580 | AF44 |
| col18_var1 | 2581 | D48 |
| col18_var1 | 2602 | H48 |
| col18_var1 | 2603 | L48 |
| col18_var1 | 2604 | P48 |
| col18_var1 | 2846 | T48 |
| col18_var1 | 2847 | X48 |
| col18_var1 | 2848 | AB48 |
| col18_var1 NTC | | AF48 |
| ddx17_var1 | 6284 | A01 |
| ddx17_var1 | 6285 | E01 |
| ddx17_var1 | 6286 | I01 |
| ddx17_var1 | 6287 | M01 |
| ddx17_var1 | 6288 | Q01 |
| ddx17_var1 | 6289 | U01 |
| ddx17_var1 | 6290 | Y01 |

| | | |
|---|---|---|
| ddx17_var1 | 6291 | AC01 |
| ddx17_var1 | 6292 | A05 |
| ddx17_var1 | 6293 | E05 |
| ddx17_var1 | 6294 | I05 |
| ddx17_var1 | 6295 | M05 |
| ddx17_var1 | 6296 | Q05 |
| ddx17_var1 | 6297 | U05 |
| ddx17_var1 | 6298 | Y05 |
| ddx17_var1 | 6299 | AC05 |
| ddx17_var1 | 6300 | A09 |
| ddx17_var1 | 6301 | E09 |
| ddx17_var1 | 6302 | I09 |
| ddx17_var1 | 6303 | M09 |
| ddx17_var1 | 6304 | Q09 |
| ddx17_var1 | 6305 | U09 |
| ddx17_var1 | 6306 | Y09 |
| ddx17_var1 | 6307 | AC09 |
| ddx17_var1 | 6308 | A13 |
| ddx17_var1 | 6309 | E13 |
| ddx17_var1 | 6310 | I13 |
| ddx17_var1 | 6311 | M13 |
| ddx17_var1 | 6312 | Q13 |
| ddx17_var1 | 6313 | U13 |
| ddx17_var1 | 6314 | Y13 |
| ddx17_var1 | 6315 | AC13 |
| ddx17_var1 | 6316 | A17 |
| ddx17_var1 | 6317 | E17 |
| ddx17_var1 | 6318 | I17 |
| ddx17_var1 | 6319 | M17 |
| ddx17_var1 | 6320 | Q17 |
| ddx17_var1 | 6321 | U17 |
| ddx17_var1 | 6322 | Y17 |
| ddx17_var1 | 6323 | AC17 |
| ddx17_var1 | 6324 | A21 |
| ddx17_var1 | 6325 | E21 |
| ddx17_var1 | 6326 | I21 |
| ddx17_var1 | 6327 | M21 |
| ddx17_var1 | 6328 | Q21 |
| ddx17_var1 | 6329 | U21 |
| ddx17_var1 | 6330 | Y21 |
| ddx17_var1 | 6331 | AC21 |
| ddx17_var1 | 6332 | A25 |
| ddx17_var1 | 6333 | E25 |
| ddx17_var1 | 6334 | I25 |
| ddx17_var1 | 6335 | M25 |
| ddx17_var1 | 6336 | Q25 |
| ddx17_var1 | 6337 | U25 |
| ddx17_var1 | 6338 | Y25 |
| ddx17_var1 | 6339 | AC25 |
| ddx17_var1 | 6340 | A29 |

| | | |
|---|---|---|
| ddx17_var | 6341 | E29 |
| ddx17_var | 6342 | I29 |
| ddx17_var | 6343 | M29 |
| ddx17_var | 6344 | Q29 |
| ddx17_var | 6345 | U29 |
| ddx17_var | 6346 | Y29 |
| ddx17_var | 6347 | AC29 |
| ddx17_var | 6348 | A33 |
| ddx17_var | 6349 | E33 |
| ddx17_var | 6350 | I33 |
| ddx17_var | 6351 | M33 |
| ddx17_var | 6352 | Q33 |
| ddx17_var | 6353 | U33 |
| ddx17_var | 6354 | Y33 |
| ddx17_var | 6355 | AC33 |
| ddx17_var | 6356 | A37 |
| ddx17_var | 6357 | E37 |
| ddx17_var | 6358 | I37 |
| ddx17_var | 6359 | M37 |
| ddx17_var | 6360 | Q37 |
| ddx17_var | 6361 | U37 |
| ddx17_var | 6362 | Y37 |
| ddx17_var | 6363 | AC37 |
| ddx17_var | 6364 | A41 |
| ddx17_var | 6365 | E41 |
| ddx17_var | 6366 | I41 |
| ddx17_var | 6367 | M41 |
| ddx17_var | 6368 | Q41 |
| ddx17_var | 6369 | U41 |
| ddx17_var | 6370 | Y41 |
| ddx17_var | 6371 | AC41 |
| ddx17_var | 6372 | A45 |
| ddx17_var | 6373 | E45 |
| ddx17_var | 6374 | I45 |
| ddx17_var | 6375 | M45 |
| ddx17_var | 6376 | Q45 |
| ddx17_var | 6377 | U45 |
| ddx17_var | 6378 | Y45 |
| ddx17_var | 6379 | AC45 |
| ddx17_var | 6380 | A03 |
| ddx17_var | 6381 | E03 |
| ddx17_var | 6382 | I03 |
| ddx17_var | 6383 | M03 |
| ddx17_var | 6384 | Q03 |
| ddx17_var | 6385 | U03 |
| ddx17_var | 6386 | Y03 |
| ddx17_var | 6387 | AC03 |
| ddx17_var | 6388 | A07 |
| ddx17_var | 6389 | E07 |
| ddx17_var | 6390 | I07 |

| | | |
|---|---|---|
| ddx17_var1 | 6391 | M07 |
| ddx17_var1 | 6392 | Q07 |
| ddx17_var1 | 6393 | U07 |
| ddx17_var1 | 6394 | Y07 |
| ddx17_var1 | 6395 | AC07 |
| ddx17_var1 | 6396 | A11 |
| ddx17_var1 | 6397 | E11 |
| ddx17_var1 | 6398 | I11 |
| ddx17_var1 | 6399 | M11 |
| ddx17_var1 | 6400 | Q11 |
| ddx17_var1 | 6401 | U11 |
| ddx17_var1 | 6402 | Y11 |
| ddx17_var1 | 6403 | AC11 |
| ddx17_var1 | 6404 | A15 |
| ddx17_var1 | 6405 | E15 |
| ddx17_var1 | 6406 | I15 |
| ddx17_var1 | 6407 | M15 |
| ddx17_var1 | 6408 | Q15 |
| ddx17_var1 | 6409 | U15 |
| ddx17_var1 | 6410 | Y15 |
| ddx17_var1 | 6411 | AC15 |
| ddx17_var1 | 6412 | A19 |
| ddx17_var1 | 6413 | E19 |
| ddx17_var1 | 6414 | I19 |
| ddx17_var1 | 6415 | M19 |
| ddx17_var1 | 6416 | Q19 |
| ddx17_var1 | 6417 | U19 |
| ddx17_var1 | 6418 | Y19 |
| ddx17_var1 | 6419 | AC19 |
| ddx17_var1 | 6421 | A23 |
| ddx17_var1 | 6422 | E23 |
| ddx17_var1 | 6423 | I23 |
| ddx17_var1 | 6424 | M23 |
| ddx17_var1 | 6425 | Q23 |
| ddx17_var1 | 6426 | U23 |
| ddx17_var1 | 6427 | Y23 |
| ddx17_var1 | 6428 | AC23 |
| ddx17_var1 | 6429 | A27 |
| ddx17_var1 | 6430 | E27 |
| ddx17_var1 | 6431 | I27 |
| ddx17_var1 | 6432 | M27 |
| ddx17_var1 | 6433 | Q27 |
| ddx17_var1 | 6434 | U27 |
| ddx17_var1 | 6435 | Y27 |
| ddx17_var1 | 6436 | AC27 |
| ddx17_var1 | 6437 | A31 |
| ddx17_var1 | 6438 | E31 |
| ddx17_var1 | 6439 | I31 |
| ddx17_var1 | 6440 | M31 |
| ddx17_var1 | 6441 | Q31 |

| | | |
|---|---|---|
| ddx17_var1 | 6442 | U31 |
| ddx17_var1 | 6443 | Y31 |
| ddx17_var1 | 6444 | AC31 |
| ddx17_var1 | 6445 | A35 |
| ddx17_var1 | 6446 | E35 |
| ddx17_var1 | 6447 | I35 |
| ddx17_var1 | 6448 | M35 |
| ddx17_var1 | 6449 | Q35 |
| ddx17_var1 | 6450 | U35 |
| ddx17_var1 | 6451 | Y35 |
| ddx17_var1 | 6452 | AC35 |
| ddx17_var1 | 6453 | A39 |
| ddx17_var1 | 6454 | E39 |
| ddx17_var1 | 6455 | I39 |
| ddx17_var1 | 6456 | M39 |
| ddx17_var1 | 6457 | Q39 |
| ddx17_var1 | 6458 | U39 |
| ddx17_var1 | 6459 | Y39 |
| ddx17_var1 | 6460 | AC39 |
| ddx17_var1 | 6461 | A43 |
| ddx17_var1 | 6462 | E43 |
| ddx17_var1 | 6463 | I43 |
| ddx17_var1 | 6464 | M43 |
| ddx17_var1 | 6465 | Q43 |
| ddx17_var1 | 6466 | U43 |
| ddx17_var1 | 6467 | Y43 |
| ddx17_var1 | 6468 | AC43 |
| ddx17_var1 | 6469 | A47 |
| ddx17_var1 | 6470 | E47 |
| ddx17_var1 | 6471 | I47 |
| ddx17_var1 | 6472 | M47 |
| ddx17_var1 | 6473 | Q47 |
| ddx17_var1 | 6474 | U47 |
| ddx17_var1 | 6475 | Y47 |
| ddx17_var1 | 6476 | AC47 |
| ddx17_var1 | 6477 | C01 |
| ddx17_var1 | 6478 | G01 |
| ddx17_var1 | 6479 | K01 |
| ddx17_var1 | 6480 | O01 |
| ddx17_var1 | 6481 | S01 |
| ddx17_var1 | 6482 | W01 |
| ddx17_var1 | 6483 | AA01 |
| ddx17_var1 | 6484 | AE01 |
| ddx17_var1 | 6485 | C05 |
| ddx17_var1 | 6486 | G05 |
| ddx17_var1 | 6487 | K05 |
| ddx17_var1 | 6488 | O05 |
| ddx17_var1 | 6489 | S05 |
| ddx17_var1 | 6490 | W05 |
| ddx17_var1 | 6491 | AA05 |

| | | |
|---|---|---|
| ddx17_var1 | 6492 | AE05 |
| ddx17_var1 | 6493 | C09 |
| ddx17_var1 | 6494 | G09 |
| ddx17_var1 | 6495 | K09 |
| ddx17_var1 | 6496 | O09 |
| ddx17_var1 | 6497 | S09 |
| ddx17_var1 | 6498 | W09 |
| ddx17_var1 | 6499 | AA09 |
| ddx17_var1 | 6500 | AE09 |
| ddx17_var1 | 6501 | C13 |
| ddx17_var1 | 6502 | G13 |
| ddx17_var1 | 6503 | K13 |
| ddx17_var1 | 6504 | O13 |
| ddx17_var1 | 6505 | S13 |
| ddx17_var1 | 6506 | W13 |
| ddx17_var1 | 6507 | AA13 |
| ddx17_var1 | 6508 | AE13 |
| ddx17_var1 | 6509 | C17 |
| ddx17_var1 | 6510 | G17 |
| ddx17_var1 | 6511 | K17 |
| ddx17_var1 | 6512 | O17 |
| ddx17_var1 | 6513 | S17 |
| ddx17_var1 | 6514 | W17 |
| ddx17_var1 | 6515 | AA17 |
| ddx17_var1 | 6516 | AE17 |
| ddx17_var1 | 6517 | C21 |
| ddx17_var1 | 6518 | G21 |
| ddx17_var1 | 6519 | K21 |
| ddx17_var1 | 6520 | O21 |
| ddx17_var1 | 6521 | S21 |
| ddx17_var1 | 6522 | W21 |
| ddx17_var1 | 6523 | AA21 |
| ddx17_var1 | 6524 | AE21 |
| ddx17_var1 | 6525 | C25 |
| ddx17_var1 | 6526 | G25 |
| ddx17_var1 | 6527 | K25 |
| ddx17_var1 | 6528 | O25 |
| ddx17_var1 | 6529 | S25 |
| ddx17_var1 | 6530 | W25 |
| ddx17_var1 | 6531 | AA25 |
| ddx17_var1 | 6532 | AE25 |
| ddx17_var1 | 6533 | C29 |
| ddx17_var1 | 6534 | G29 |
| ddx17_var1 | 6535 | K29 |
| ddx17_var1 | 6536 | O29 |
| ddx17_var1 | 6537 | S29 |
| ddx17_var1 | 6538 | W29 |
| ddx17_var1 | 6539 | AA29 |
| ddx17_var1 | 6540 | AE29 |
| ddx17_var1 | 6541 | C33 |

| | | |
|---|---|---|
| ddx17_var1 | 6542 | G33 |
| ddx17_var1 | 6543 | K33 |
| ddx17_var1 | 6544 | O33 |
| ddx17_var1 | 6545 | S33 |
| ddx17_var1 | 6546 | W33 |
| ddx17_var1 | 6547 | AA33 |
| ddx17_var1 | 6548 | AE33 |
| ddx17_var1 | 6549 | C37 |
| ddx17_var1 | 6550 | G37 |
| ddx17_var1 | 6551 | K37 |
| ddx17_var1 | 6552 | O37 |
| ddx17_var1 | 6553 | S37 |
| ddx17_var1 | 6554 | W37 |
| ddx17_var1 | 6555 | AA37 |
| ddx17_var1 | 6556 | AE37 |
| ddx17_var1 | 6557 | C41 |
| ddx17_var1 | 6558 | G41 |
| ddx17_var1 | 6559 | K41 |
| ddx17_var1 | 6560 | O41 |
| ddx17_var1 | 6561 | S41 |
| ddx17_var1 | 6562 | W41 |
| ddx17_var1 | 6563 | AA41 |
| ddx17_var1 | 6564 | AE41 |
| ddx17_var1 | 6565 | C45 |
| ddx17_var1 | 6566 | G45 |
| ddx17_var1 | 6567 | K45 |
| ddx17_var1 | 6568 | O45 |
| ddx17_var1 | 6569 | S45 |
| ddx17_var1 | 6570 | W45 |
| ddx17_var1 | 6571 | AA45 |
| ddx17_var1 | 6572 | AE45 |
| ddx17_var1 | 6573 | C03 |
| ddx17_var1 | 6574 | G03 |
| ddx17_var1 | 6575 | K03 |
| ddx17_var1 | 6576 | O03 |
| ddx17_var1 | 6577 | S03 |
| ddx17_var1 | 6578 | W03 |
| ddx17_var1 | 6579 | AA03 |
| ddx17_var1 | 6580 | AE03 |
| ddx17_var1 | 6581 | C07 |
| ddx17_var1 | 6582 | G07 |
| ddx17_var1 | 6583 | K07 |
| ddx17_var1 | 6584 | O07 |
| ddx17_var1 | 6585 | S07 |
| ddx17_var1 | 6586 | W07 |
| ddx17_var1 | 6587 | AA07 |
| ddx17_var1 | 6588 | AE07 |
| ddx17_var1 | 6589 | C11 |
| ddx17_var1 | 6590 | G11 |
| ddx17_var1 | 6591 | K11 |

| | | |
|---|---|---|
| ddx17_var1 | 6592 | O11 |
| ddx17_var1 | 6593 | S11 |
| ddx17_var1 | 6594 | W11 |
| ddx17_var1 | 6595 | AA11 |
| ddx17_var1 | 6596 | AE11 |
| ddx17_var1 | 6597 | C15 |
| ddx17_var1 | 6598 | G15 |
| ddx17_var1 | 6599 | K15 |
| ddx17_var1 | 6600 | O15 |
| ddx17_var1 | 6601 | S15 |
| ddx17_var1 | 6602 | W15 |
| ddx17_var1 | 6603 | AA15 |
| ddx17_var1 | 6604 | AE15 |
| ddx17_var1 | 6605 | C19 |
| ddx17_var1 | 6606 | G19 |
| ddx17_var1 | 6607 | K19 |
| ddx17_var1 | 6608 | O19 |
| ddx17_var1 | 6609 | S19 |
| ddx17_var1 | 6610 | W19 |
| ddx17_var1 | 6611 | AA19 |
| ddx17_var1 | 6612 | AE19 |
| ddx17_var1 | 6613 | C23 |
| ddx17_var1 | 6614 | G23 |
| ddx17_var1 | 6615 | K23 |
| ddx17_var1 | 6616 | O23 |
| ddx17_var1 | 6617 | S23 |
| ddx17_var1 | 6618 | W23 |
| ddx17_var1 | 6619 | AA23 |
| ddx17_var1 | 6620 | AE23 |
| ddx17_var1 | 6621 | C27 |
| ddx17_var1 | 6622 | G27 |
| ddx17_var1 | 6623 | K27 |
| ddx17_var1 | 6624 | O27 |
| ddx17_var1 | 6625 | S27 |
| ddx17_var1 | 2815 | W27 |
| ddx17_var1 | 2816 | AA27 |
| ddx17_var1 | 2817 | AE27 |
| ddx17_var1 | 2820 | C31 |
| ddx17_var1 | 2821 | G31 |
| ddx17_var1 | 2822 | K31 |
| ddx17_var1 | 3245 | O31 |
| ddx17_var1 | 3246 | S31 |
| ddx17_var1 | 3247 | W31 |
| ddx17_var1 | 3472 | AA31 |
| ddx17_var1 | 3473 | AE31 |
| ddx17_var1 | 3474 | C35 |
| ddx17_var1 | 3327 | G35 |
| ddx17_var1 | 3583 | K35 |
| ddx17_var1 | 3584 | O35 |
| ddx17_var1 | 3601 | S35 |

| | | |
|---|---|---|
| ddx17_var1 | 3602 | W35 |
| ddx17_var1 | 3603 | AA35 |
| ddx17_var1 | 3775 | AE35 |
| ddx17_var1 | 3776 | C39 |
| ddx17_var1 | 3777 | G39 |
| ddx17_var1 | 1808 | K39 |
| ddx17_var1 | 1809 | O39 |
| ddx17_var1 | 1812 | S39 |
| ddx17_var1 | 1879 | W39 |
| ddx17_var1 | 1880 | AA39 |
| ddx17_var1 | 1881 | AE39 |
| ddx17_var1 | 2360 | C43 |
| ddx17_var1 | 2361 | G43 |
| ddx17_var1 | 2362 | K43 |
| ddx17_var1 | 2492 | O43 |
| ddx17_var1 | 2493 | S43 |
| ddx17_var1 | 2494 | W43 |
| ddx17_var1 | 2579 | AA43 |
| ddx17_var1 | 2580 | AE43 |
| ddx17_var1 | 2581 | C47 |
| ddx17_var1 | 2602 | G47 |
| ddx17_var1 | 2603 | K47 |
| ddx17_var1 | 2604 | O47 |
| ddx17_var1 | 2846 | S47 |
| ddx17_var1 | 2847 | W47 |
| ddx17_var1 | 2848 | AA47 |
| ddx17_var1 | NTC | AE47 |
| dnaj_var1 | 6284 | A02 |
| dnaj_var1 | 6285 | E02 |
| dnaj_var1 | 6286 | I02 |
| dnaj_var1 | 6287 | M02 |
| dnaj_var1 | 6288 | Q02 |
| dnaj_var1 | 6289 | U02 |
| dnaj_var1 | 6290 | Y02 |
| dnaj_var1 | 6291 | AC02 |
| dnaj_var1 | 6292 | A06 |
| dnaj_var1 | 6293 | E06 |
| dnaj_var1 | 6294 | I06 |
| dnaj_var1 | 6295 | M06 |
| dnaj_var1 | 6296 | Q06 |
| dnaj_var1 | 6297 | U06 |
| dnaj_var1 | 6298 | Y06 |
| dnaj_var1 | 6299 | AC06 |
| dnaj_var1 | 6300 | A10 |
| dnaj_var1 | 6301 | E10 |
| dnaj_var1 | 6302 | I10 |
| dnaj_var1 | 6303 | M10 |
| dnaj_var1 | 6304 | Q10 |
| dnaj_var1 | 6305 | U10 |
| dnaj_var1 | 6306 | Y10 |

| | | |
|---|---|---|
| dnaj_var1 | 6307 | AC10 |
| dnaj_var1 | 6308 | A14 |
| dnaj_var1 | 6309 | E14 |
| dnaj_var1 | 6310 | I14 |
| dnaj_var1 | 6311 | M14 |
| dnaj_var1 | 6312 | Q14 |
| dnaj_var1 | 6313 | U14 |
| dnaj_var1 | 6314 | Y14 |
| dnaj_var1 | 6315 | AC14 |
| dnaj_var1 | 6316 | A18 |
| dnaj_var1 | 6317 | E18 |
| dnaj_var1 | 6318 | I18 |
| dnaj_var1 | 6319 | M18 |
| dnaj_var1 | 6320 | Q18 |
| dnaj_var1 | 6321 | U18 |
| dnaj_var1 | 6322 | Y18 |
| dnaj_var1 | 6323 | AC18 |
| dnaj_var1 | 6324 | A22 |
| dnaj_var1 | 6325 | E22 |
| dnaj_var1 | 6326 | I22 |
| dnaj_var1 | 6327 | M22 |
| dnaj_var1 | 6328 | Q22 |
| dnaj_var1 | 6329 | U22 |
| dnaj_var1 | 6330 | Y22 |
| dnaj_var1 | 6331 | AC22 |
| dnaj_var1 | 6332 | A26 |
| dnaj_var1 | 6333 | E26 |
| dnaj_var1 | 6334 | I26 |
| dnaj_var1 | 6335 | M26 |
| dnaj_var1 | 6336 | Q26 |
| dnaj_var1 | 6337 | U26 |
| dnaj_var1 | 6338 | Y26 |
| dnaj_var1 | 6339 | AC26 |
| dnaj_var1 | 6340 | A30 |
| dnaj_var1 | 6341 | E30 |
| dnaj_var1 | 6342 | I30 |
| dnaj_var1 | 6343 | M30 |
| dnaj_var1 | 6344 | Q30 |
| dnaj_var1 | 6345 | U30 |
| dnaj_var1 | 6346 | Y30 |
| dnaj_var1 | 6347 | AC30 |
| dnaj_var1 | 6348 | A34 |
| dnaj_var1 | 6349 | E34 |
| dnaj_var1 | 6350 | I34 |
| dnaj_var1 | 6351 | M34 |
| dnaj_var1 | 6352 | Q34 |
| dnaj_var1 | 6353 | U34 |
| dnaj_var1 | 6354 | Y34 |
| dnaj_var1 | 6355 | AC34 |
| dnaj_var1 | 6356 | A38 |

| | | |
|---|---|---|
| dnaj_var1 | 6357 | E38 |
| dnaj_var1 | 6358 | I38 |
| dnaj_var1 | 6359 | M38 |
| dnaj_var1 | 6360 | Q38 |
| dnaj_var1 | 6361 | U38 |
| dnaj_var1 | 6362 | Y38 |
| dnaj_var1 | 6363 | AC38 |
| dnaj_var1 | 6364 | A42 |
| dnaj_var1 | 6365 | E42 |
| dnaj_var1 | 6366 | I42 |
| dnaj_var1 | 6367 | M42 |
| dnaj_var1 | 6368 | Q42 |
| dnaj_var1 | 6369 | U42 |
| dnaj_var1 | 6370 | Y42 |
| dnaj_var1 | 6371 | AC42 |
| dnaj_var1 | 6372 | A46 |
| dnaj_var1 | 6373 | E46 |
| dnaj_var1 | 6374 | I46 |
| dnaj_var1 | 6375 | M46 |
| dnaj_var1 | 6376 | Q46 |
| dnaj_var1 | 6377 | U46 |
| dnaj_var1 | 6378 | Y46 |
| dnaj_var1 | 6379 | AC46 |
| dnaj_var1 | 6380 | A04 |
| dnaj_var1 | 6381 | E04 |
| dnaj_var1 | 6382 | I04 |
| dnaj_var1 | 6383 | M04 |
| dnaj_var1 | 6384 | Q04 |
| dnaj_var1 | 6385 | U04 |
| dnaj_var1 | 6386 | Y04 |
| dnaj_var1 | 6387 | AC04 |
| dnaj_var1 | 6388 | A08 |
| dnaj_var1 | 6389 | E08 |
| dnaj_var1 | 6390 | I08 |
| dnaj_var1 | 6391 | M08 |
| dnaj_var1 | 6392 | Q08 |
| dnaj_var1 | 6393 | U08 |
| dnaj_var1 | 6394 | Y08 |
| dnaj_var1 | 6395 | AC08 |
| dnaj_var1 | 6396 | A12 |
| dnaj_var1 | 6397 | E12 |
| dnaj_var1 | 6398 | I12 |
| dnaj_var1 | 6399 | M12 |
| dnaj_var1 | 6400 | Q12 |
| dnaj_var1 | 6401 | U12 |
| dnaj_var1 | 6402 | Y12 |
| dnaj_var1 | 6403 | AC12 |
| dnaj_var1 | 6404 | A16 |
| dnaj_var1 | 6405 | E16 |
| dnaj_var1 | 6406 | I16 |

| | | |
|---|---|---|
| dnaj_var1 | 6407 | M16 |
| dnaj_var1 | 6408 | Q16 |
| dnaj_var1 | 6409 | U16 |
| dnaj_var1 | 6410 | Y16 |
| dnaj_var1 | 6411 | AC16 |
| dnaj_var1 | 6412 | A20 |
| dnaj_var1 | 6413 | E20 |
| dnaj_var1 | 6414 | I20 |
| dnaj_var1 | 6415 | M20 |
| dnaj_var1 | 6416 | Q20 |
| dnaj_var1 | 6417 | U20 |
| dnaj_var1 | 6418 | Y20 |
| dnaj_var1 | 6419 | AC20 |
| dnaj_var1 | 6421 | A24 |
| dnaj_var1 | 6422 | E24 |
| dnaj_var1 | 6423 | I24 |
| dnaj_var1 | 6424 | M24 |
| dnaj_var1 | 6425 | Q24 |
| dnaj_var1 | 6426 | U24 |
| dnaj_var1 | 6427 | Y24 |
| dnaj_var1 | 6428 | AC24 |
| dnaj_var1 | 6429 | A28 |
| dnaj_var1 | 6430 | E28 |
| dnaj_var1 | 6431 | I28 |
| dnaj_var1 | 6432 | M28 |
| dnaj_var1 | 6433 | Q28 |
| dnaj_var1 | 6434 | U28 |
| dnaj_var1 | 6435 | Y28 |
| dnaj_var1 | 6436 | AC28 |
| dnaj_var1 | 6437 | A32 |
| dnaj_var1 | 6438 | E32 |
| dnaj_var1 | 6439 | I32 |
| dnaj_var1 | 6440 | M32 |
| dnaj_var1 | 6441 | Q32 |
| dnaj_var1 | 6442 | U32 |
| dnaj_var1 | 6443 | Y32 |
| dnaj_var1 | 6444 | AC32 |
| dnaj_var1 | 6445 | A36 |
| dnaj_var1 | 6446 | E36 |
| dnaj_var1 | 6447 | I36 |
| dnaj_var1 | 6448 | M36 |
| dnaj_var1 | 6449 | Q36 |
| dnaj_var1 | 6450 | U36 |
| dnaj_var1 | 6451 | Y36 |
| dnaj_var1 | 6452 | AC36 |
| dnaj_var1 | 6453 | A40 |
| dnaj_var1 | 6454 | E40 |
| dnaj_var1 | 6455 | I40 |
| dnaj_var1 | 6456 | M40 |
| dnaj_var1 | 6457 | Q40 |

| | | |
|---|---|---|
| dnaj_var1 | 6458 | U40 |
| dnaj_var1 | 6459 | Y40 |
| dnaj_var1 | 6460 | AC40 |
| dnaj_var1 | 6461 | A44 |
| dnaj_var1 | 6462 | E44 |
| dnaj_var1 | 6463 | I44 |
| dnaj_var1 | 6464 | M44 |
| dnaj_var1 | 6465 | Q44 |
| dnaj_var1 | 6466 | U44 |
| dnaj_var1 | 6467 | Y44 |
| dnaj_var1 | 6468 | AC44 |
| dnaj_var1 | 6469 | A48 |
| dnaj_var1 | 6470 | E48 |
| dnaj_var1 | 6471 | I48 |
| dnaj_var1 | 6472 | M48 |
| dnaj_var1 | 6473 | Q48 |
| dnaj_var1 | 6474 | U48 |
| dnaj_var1 | 6475 | Y48 |
| dnaj_var1 | 6476 | AC48 |
| dnaj_var1 | 6477 | C02 |
| dnaj_var1 | 6478 | G02 |
| dnaj_var1 | 6479 | K02 |
| dnaj_var1 | 6480 | O02 |
| dnaj_var1 | 6481 | S02 |
| dnaj_var1 | 6482 | W02 |
| dnaj_var1 | 6483 | AA02 |
| dnaj_var1 | 6484 | AE02 |
| dnaj_var1 | 6485 | C06 |
| dnaj_var1 | 6486 | G06 |
| dnaj_var1 | 6487 | K06 |
| dnaj_var1 | 6488 | O06 |
| dnaj_var1 | 6489 | S06 |
| dnaj_var1 | 6490 | W06 |
| dnaj_var1 | 6491 | AA06 |
| dnaj_var1 | 6492 | AE06 |
| dnaj_var1 | 6493 | C10 |
| dnaj_var1 | 6494 | G10 |
| dnaj_var1 | 6495 | K10 |
| dnaj_var1 | 6496 | O10 |
| dnaj_var1 | 6497 | S10 |
| dnaj_var1 | 6498 | W10 |
| dnaj_var1 | 6499 | AA10 |
| dnaj_var1 | 6500 | AE10 |
| dnaj_var1 | 6501 | C14 |
| dnaj_var1 | 6502 | G14 |
| dnaj_var1 | 6503 | K14 |
| dnaj_var1 | 6504 | O14 |
| dnaj_var1 | 6505 | S14 |
| dnaj_var1 | 6506 | W14 |
| dnaj_var1 | 6507 | AA14 |

| | | |
|---|---|---|
| dnaj_var1 | 6508 | AE14 |
| dnaj_var1 | 6509 | C18 |
| dnaj_var1 | 6510 | G18 |
| dnaj_var1 | 6511 | K18 |
| dnaj_var1 | 6512 | O18 |
| dnaj_var1 | 6513 | S18 |
| dnaj_var1 | 6514 | W18 |
| dnaj_var1 | 6515 | AA18 |
| dnaj_var1 | 6516 | AE18 |
| dnaj_var1 | 6517 | C22 |
| dnaj_var1 | 6518 | G22 |
| dnaj_var1 | 6519 | K22 |
| dnaj_var1 | 6520 | O22 |
| dnaj_var1 | 6521 | S22 |
| dnaj_var1 | 6522 | W22 |
| dnaj_var1 | 6523 | AA22 |
| dnaj_var1 | 6524 | AE22 |
| dnaj_var1 | 6525 | C26 |
| dnaj_var1 | 6526 | G26 |
| dnaj_var1 | 6527 | K26 |
| dnaj_var1 | 6528 | O26 |
| dnaj_var1 | 6529 | S26 |
| dnaj_var1 | 6530 | W26 |
| dnaj_var1 | 6531 | AA26 |
| dnaj_var1 | 6532 | AE26 |
| dnaj_var1 | 6533 | C30 |
| dnaj_var1 | 6534 | G30 |
| dnaj_var1 | 6535 | K30 |
| dnaj_var1 | 6536 | O30 |
| dnaj_var1 | 6537 | S30 |
| dnaj_var1 | 6538 | W30 |
| dnaj_var1 | 6539 | AA30 |
| dnaj_var1 | 6540 | AE30 |
| dnaj_var1 | 6541 | C34 |
| dnaj_var1 | 6542 | G34 |
| dnaj_var1 | 6543 | K34 |
| dnaj_var1 | 6544 | O34 |
| dnaj_var1 | 6545 | S34 |
| dnaj_var1 | 6546 | W34 |
| dnaj_var1 | 6547 | AA34 |
| dnaj_var1 | 6548 | AE34 |
| dnaj_var1 | 6549 | C38 |
| dnaj_var1 | 6550 | G38 |
| dnaj_var1 | 6551 | K38 |
| dnaj_var1 | 6552 | O38 |
| dnaj_var1 | 6553 | S38 |
| dnaj_var1 | 6554 | W38 |
| dnaj_var1 | 6555 | AA38 |
| dnaj_var1 | 6556 | AE38 |
| dnaj_var1 | 6557 | C42 |

| | | |
|---|---|---|
| dnaj_var1 | 6558 | G42 |
| dnaj_var1 | 6559 | K42 |
| dnaj_var1 | 6560 | O42 |
| dnaj_var1 | 6561 | S42 |
| dnaj_var1 | 6562 | W42 |
| dnaj_var1 | 6563 | AA42 |
| dnaj_var1 | 6564 | AE42 |
| dnaj_var1 | 6565 | C46 |
| dnaj_var1 | 6566 | G46 |
| dnaj_var1 | 6567 | K46 |
| dnaj_var1 | 6568 | O46 |
| dnaj_var1 | 6569 | S46 |
| dnaj_var1 | 6570 | W46 |
| dnaj_var1 | 6571 | AA46 |
| dnaj_var1 | 6572 | AE46 |
| dnaj_var1 | 6573 | C04 |
| dnaj_var1 | 6574 | G04 |
| dnaj_var1 | 6575 | K04 |
| dnaj_var1 | 6576 | O04 |
| dnaj_var1 | 6577 | S04 |
| dnaj_var1 | 6578 | W04 |
| dnaj_var1 | 6579 | AA04 |
| dnaj_var1 | 6580 | AE04 |
| dnaj_var1 | 6581 | C08 |
| dnaj_var1 | 6582 | G08 |
| dnaj_var1 | 6583 | K08 |
| dnaj_var1 | 6584 | O08 |
| dnaj_var1 | 6585 | S08 |
| dnaj_var1 | 6586 | W08 |
| dnaj_var1 | 6587 | AA08 |
| dnaj_var1 | 6588 | AE08 |
| dnaj_var1 | 6589 | C12 |
| dnaj_var1 | 6590 | G12 |
| dnaj_var1 | 6591 | K12 |
| dnaj_var1 | 6592 | O12 |
| dnaj_var1 | 6593 | S12 |
| dnaj_var1 | 6594 | W12 |
| dnaj_var1 | 6595 | AA12 |
| dnaj_var1 | 6596 | AE12 |
| dnaj_var1 | 6597 | C16 |
| dnaj_var1 | 6598 | G16 |
| dnaj_var1 | 6599 | K16 |
| dnaj_var1 | 6600 | O16 |
| dnaj_var1 | 6601 | S16 |
| dnaj_var1 | 6602 | W16 |
| dnaj_var1 | 6603 | AA16 |
| dnaj_var1 | 6604 | AE16 |
| dnaj_var1 | 6605 | C20 |
| dnaj_var1 | 6606 | G20 |
| dnaj_var1 | 6607 | K20 |

| | | |
|---|---|---|
| dnaj_var1 | 6608 | O20 |
| dnaj_var1 | 6609 | S20 |
| dnaj_var1 | 6610 | W20 |
| dnaj_var1 | 6611 | AA20 |
| dnaj_var1 | 6612 | AE20 |
| dnaj_var1 | 6613 | C24 |
| dnaj_var1 | 6614 | G24 |
| dnaj_var1 | 6615 | K24 |
| dnaj_var1 | 6616 | O24 |
| dnaj_var1 | 6617 | S24 |
| dnaj_var1 | 6618 | W24 |
| dnaj_var1 | 6619 | AA24 |
| dnaj_var1 | 6620 | AE24 |
| dnaj_var1 | 6621 | C28 |
| dnaj_var1 | 6622 | G28 |
| dnaj_var1 | 6623 | K28 |
| dnaj_var1 | 6624 | O28 |
| dnaj_var1 | 6625 | S28 |
| dnaj_var1 | 2815 | W28 |
| dnaj_var1 | 2816 | AA28 |
| dnaj_var1 | 2817 | AE28 |
| dnaj_var1 | 2820 | C32 |
| dnaj_var1 | 2821 | G32 |
| dnaj_var1 | 2822 | K32 |
| dnaj_var1 | 3245 | O32 |
| dnaj_var1 | 3246 | S32 |
| dnaj_var1 | 3247 | W32 |
| dnaj_var1 | 3472 | AA32 |
| dnaj_var1 | 3473 | AE32 |
| dnaj_var1 | 3474 | C36 |
| dnaj_var1 | 3327 | G36 |
| dnaj_var1 | 3583 | K36 |
| dnaj_var1 | 3584 | O36 |
| dnaj_var1 | 3601 | S36 |
| dnaj_var1 | 3602 | W36 |
| dnaj_var1 | 3603 | AA36 |
| dnaj_var1 | 3775 | AE36 |
| dnaj_var1 | 3776 | C40 |
| dnaj_var1 | 3777 | G40 |
| dnaj_var1 | 1808 | K40 |
| dnaj_var1 | 1809 | O40 |
| dnaj_var1 | 1812 | S40 |
| dnaj_var1 | 1879 | W40 |
| dnaj_var1 | 1880 | AA40 |
| dnaj_var1 | 1881 | AE40 |
| dnaj_var1 | 2360 | C44 |
| dnaj_var1 | 2361 | G44 |
| dnaj_var1 | 2362 | K44 |
| dnaj_var1 | 2492 | O44 |
| dnaj_var1 | 2493 | S44 |

| | | |
|---|---|---|
| dnaj_var1 | 2494 | W44 |
| dnaj_var1 | 2579 | AA44 |
| dnaj_var1 | 2580 | AE44 |
| dnaj_var1 | 2581 | C48 |
| dnaj_var1 | 2602 | G48 |
| dnaj_var1 | 2603 | K48 |
| dnaj_var1 | 2604 | O48 |
| dnaj_var1 | 2846 | S48 |
| dnaj_var1 | 2847 | W48 |
| dnaj_var1 | 2848 | AA48 |
| dnaj_var1 | NTC | AE48 |
| fam178_va | 6284 | B01 |
| fam178_va | 6285 | F01 |
| fam178_va | 6286 | J01 |
| fam178_va | 6287 | N01 |
| fam178_va | 6288 | R01 |
| fam178_va | 6289 | V01 |
| fam178_va | 6290 | Z01 |
| fam178_va | 6291 | AD01 |
| fam178_va | 6292 | B05 |
| fam178_va | 6293 | F05 |
| fam178_va | 6294 | J05 |
| fam178_va | 6295 | N05 |
| fam178_va | 6296 | R05 |
| fam178_va | 6297 | V05 |
| fam178_va | 6298 | Z05 |
| fam178_va | 6299 | AD05 |
| fam178_va | 6300 | B09 |
| fam178_va | 6301 | F09 |
| fam178_va | 6302 | J09 |
| fam178_va | 6303 | N09 |
| fam178_va | 6304 | R09 |
| fam178_va | 6305 | V09 |
| fam178_va | 6306 | Z09 |
| fam178_va | 6307 | AD09 |
| fam178_va | 6308 | B13 |
| fam178_va | 6309 | F13 |
| fam178_va | 6310 | J13 |
| fam178_va | 6311 | N13 |
| fam178_va | 6312 | R13 |
| fam178_va | 6313 | V13 |
| fam178_va | 6314 | Z13 |
| fam178_va | 6315 | AD13 |
| fam178_va | 6316 | B17 |
| fam178_va | 6317 | F17 |
| fam178_va | 6318 | J17 |
| fam178_va | 6319 | N17 |
| fam178_va | 6320 | R17 |
| fam178_va | 6321 | V17 |
| fam178_va | 6322 | Z17 |

| | | |
|---|---|---|
| fam178_va | 6323 | AD17 |
| fam178_va | 6324 | B21 |
| fam178_va | 6325 | F21 |
| fam178_va | 6326 | J21 |
| fam178_va | 6327 | N21 |
| fam178_va | 6328 | R21 |
| fam178_va | 6329 | V21 |
| fam178_va | 6330 | Z21 |
| fam178_va | 6331 | AD21 |
| fam178_va | 6332 | B25 |
| fam178_va | 6333 | F25 |
| fam178_va | 6334 | J25 |
| fam178_va | 6335 | N25 |
| fam178_va | 6336 | R25 |
| fam178_va | 6337 | V25 |
| fam178_va | 6338 | Z25 |
| fam178_va | 6339 | AD25 |
| fam178_va | 6340 | B29 |
| fam178_va | 6341 | F29 |
| fam178_va | 6342 | J29 |
| fam178_va | 6343 | N29 |
| fam178_va | 6344 | R29 |
| fam178_va | 6345 | V29 |
| fam178_va | 6346 | Z29 |
| fam178_va | 6347 | AD29 |
| fam178_va | 6348 | B33 |
| fam178_va | 6349 | F33 |
| fam178_va | 6350 | J33 |
| fam178_va | 6351 | N33 |
| fam178_va | 6352 | R33 |
| fam178_va | 6353 | V33 |
| fam178_va | 6354 | Z33 |
| fam178_va | 6355 | AD33 |
| fam178_va | 6356 | B37 |
| fam178_va | 6357 | F37 |
| fam178_va | 6358 | J37 |
| fam178_va | 6359 | N37 |
| fam178_va | 6360 | R37 |
| fam178_va | 6361 | V37 |
| fam178_va | 6362 | Z37 |
| fam178_va | 6363 | AD37 |
| fam178_va | 6364 | B41 |
| fam178_va | 6365 | F41 |
| fam178_va | 6366 | J41 |
| fam178_va | 6367 | N41 |
| fam178_va | 6368 | R41 |
| fam178_va | 6369 | V41 |
| fam178_va | 6370 | Z41 |
| fam178_va | 6371 | AD41 |
| fam178_va | 6372 | B45 |

| | | |
|---|---|---|
| fam178_va | 6373 | F45 |
| fam178_va | 6374 | J45 |
| fam178_va | 6375 | N45 |
| fam178_va | 6376 | R45 |
| fam178_va | 6377 | V45 |
| fam178_va | 6378 | Z45 |
| fam178_va | 6379 | AD45 |
| fam178_va | 6380 | B03 |
| fam178_va | 6381 | F03 |
| fam178_va | 6382 | J03 |
| fam178_va | 6383 | N03 |
| fam178_va | 6384 | R03 |
| fam178_va | 6385 | V03 |
| fam178_va | 6386 | Z03 |
| fam178_va | 6387 | AD03 |
| fam178_va | 6388 | B07 |
| fam178_va | 6389 | F07 |
| fam178_va | 6390 | J07 |
| fam178_va | 6391 | N07 |
| fam178_va | 6392 | R07 |
| fam178_va | 6393 | V07 |
| fam178_va | 6394 | Z07 |
| fam178_va | 6395 | AD07 |
| fam178_va | 6396 | B11 |
| fam178_va | 6397 | F11 |
| fam178_va | 6398 | J11 |
| fam178_va | 6399 | N11 |
| fam178_va | 6400 | R11 |
| fam178_va | 6401 | V11 |
| fam178_va | 6402 | Z11 |
| fam178_va | 6403 | AD11 |
| fam178_va | 6404 | B15 |
| fam178_va | 6405 | F15 |
| fam178_va | 6406 | J15 |
| fam178_va | 6407 | N15 |
| fam178_va | 6408 | R15 |
| fam178_va | 6409 | V15 |
| fam178_va | 6410 | Z15 |
| fam178_va | 6411 | AD15 |
| fam178_va | 6412 | B19 |
| fam178_va | 6413 | F19 |
| fam178_va | 6414 | J19 |
| fam178_va | 6415 | N19 |
| fam178_va | 6416 | R19 |
| fam178_va | 6417 | V19 |
| fam178_va | 6418 | Z19 |
| fam178_va | 6419 | AD19 |
| fam178_va | 6421 | B23 |
| fam178_va | 6422 | F23 |
| fam178_va | 6423 | J23 |

| | | |
|---|---|---|
| fam178_va | 6424 | N23 |
| fam178_va | 6425 | R23 |
| fam178_va | 6426 | V23 |
| fam178_va | 6427 | Z23 |
| fam178_va | 6428 | AD23 |
| fam178_va | 6429 | B27 |
| fam178_va | 6430 | F27 |
| fam178_va | 6431 | J27 |
| fam178_va | 6432 | N27 |
| fam178_va | 6433 | R27 |
| fam178_va | 6434 | V27 |
| fam178_va | 6435 | Z27 |
| fam178_va | 6436 | AD27 |
| fam178_va | 6437 | B31 |
| fam178_va | 6438 | F31 |
| fam178_va | 6439 | J31 |
| fam178_va | 6440 | N31 |
| fam178_va | 6441 | R31 |
| fam178_va | 6442 | V31 |
| fam178_va | 6443 | Z31 |
| fam178_va | 6444 | AD31 |
| fam178_va | 6445 | B35 |
| fam178_va | 6446 | F35 |
| fam178_va | 6447 | J35 |
| fam178_va | 6448 | N35 |
| fam178_va | 6449 | R35 |
| fam178_va | 6450 | V35 |
| fam178_va | 6451 | Z35 |
| fam178_va | 6452 | AD35 |
| fam178_va | 6453 | B39 |
| fam178_va | 6454 | F39 |
| fam178_va | 6455 | J39 |
| fam178_va | 6456 | N39 |
| fam178_va | 6457 | R39 |
| fam178_va | 6458 | V39 |
| fam178_va | 6459 | Z39 |
| fam178_va | 6460 | AD39 |
| fam178_va | 6461 | B43 |
| fam178_va | 6462 | F43 |
| fam178_va | 6463 | J43 |
| fam178_va | 6464 | N43 |
| fam178_va | 6465 | R43 |
| fam178_va | 6466 | V43 |
| fam178_va | 6467 | Z43 |
| fam178_va | 6468 | AD43 |
| fam178_va | 6469 | B47 |
| fam178_va | 6470 | F47 |
| fam178_va | 6471 | J47 |
| fam178_va | 6472 | N47 |
| fam178_va | 6473 | R47 |

| | | |
|---|---|---|
| fam178_va | 6474 | V47 |
| fam178_va | 6475 | Z47 |
| fam178_va | 6476 | AD47 |
| fam178_va | 6477 | D01 |
| fam178_va | 6478 | H01 |
| fam178_va | 6479 | L01 |
| fam178_va | 6480 | P01 |
| fam178_va | 6481 | T01 |
| fam178_va | 6482 | X01 |
| fam178_va | 6483 | AB01 |
| fam178_va | 6484 | AF01 |
| fam178_va | 6485 | D05 |
| fam178_va | 6486 | H05 |
| fam178_va | 6487 | L05 |
| fam178_va | 6488 | P05 |
| fam178_va | 6489 | T05 |
| fam178_va | 6490 | X05 |
| fam178_va | 6491 | AB05 |
| fam178_va | 6492 | AF05 |
| fam178_va | 6493 | D09 |
| fam178_va | 6494 | H09 |
| fam178_va | 6495 | L09 |
| fam178_va | 6496 | P09 |
| fam178_va | 6497 | T09 |
| fam178_va | 6498 | X09 |
| fam178_va | 6499 | AB09 |
| fam178_va | 6500 | AF09 |
| fam178_va | 6501 | D13 |
| fam178_va | 6502 | H13 |
| fam178_va | 6503 | L13 |
| fam178_va | 6504 | P13 |
| fam178_va | 6505 | T13 |
| fam178_va | 6506 | X13 |
| fam178_va | 6507 | AB13 |
| fam178_va | 6508 | AF13 |
| fam178_va | 6509 | D17 |
| fam178_va | 6510 | H17 |
| fam178_va | 6511 | L17 |
| fam178_va | 6512 | P17 |
| fam178_va | 6513 | T17 |
| fam178_va | 6514 | X17 |
| fam178_va | 6515 | AB17 |
| fam178_va | 6516 | AF17 |
| fam178_va | 6517 | D21 |
| fam178_va | 6518 | H21 |
| fam178_va | 6519 | L21 |
| fam178_va | 6520 | P21 |
| fam178_va | 6521 | T21 |
| fam178_va | 6522 | X21 |
| fam178_va | 6523 | AB21 |

| | | |
|---|---|---|
| fam178_va | 6524 | AF21 |
| fam178_va | 6525 | D25 |
| fam178_va | 6526 | H25 |
| fam178_va | 6527 | L25 |
| fam178_va | 6528 | P25 |
| fam178_va | 6529 | T25 |
| fam178_va | 6530 | X25 |
| fam178_va | 6531 | AB25 |
| fam178_va | 6532 | AF25 |
| fam178_va | 6533 | D29 |
| fam178_va | 6534 | H29 |
| fam178_va | 6535 | L29 |
| fam178_va | 6536 | P29 |
| fam178_va | 6537 | T29 |
| fam178_va | 6538 | X29 |
| fam178_va | 6539 | AB29 |
| fam178_va | 6540 | AF29 |
| fam178_va | 6541 | D33 |
| fam178_va | 6542 | H33 |
| fam178_va | 6543 | L33 |
| fam178_va | 6544 | P33 |
| fam178_va | 6545 | T33 |
| fam178_va | 6546 | X33 |
| fam178_va | 6547 | AB33 |
| fam178_va | 6548 | AF33 |
| fam178_va | 6549 | D37 |
| fam178_va | 6550 | H37 |
| fam178_va | 6551 | L37 |
| fam178_va | 6552 | P37 |
| fam178_va | 6553 | T37 |
| fam178_va | 6554 | X37 |
| fam178_va | 6555 | AB37 |
| fam178_va | 6556 | AF37 |
| fam178_va | 6557 | D41 |
| fam178_va | 6558 | H41 |
| fam178_va | 6559 | L41 |
| fam178_va | 6560 | P41 |
| fam178_va | 6561 | T41 |
| fam178_va | 6562 | X41 |
| fam178_va | 6563 | AB41 |
| fam178_va | 6564 | AF41 |
| fam178_va | 6565 | D45 |
| fam178_va | 6566 | H45 |
| fam178_va | 6567 | L45 |
| fam178_va | 6568 | P45 |
| fam178_va | 6569 | T45 |
| fam178_va | 6570 | X45 |
| fam178_va | 6571 | AB45 |
| fam178_va | 6572 | AF45 |
| fam178_va | 6573 | D03 |

| | | |
|---|---|---|
| fam178_va | 6574 | H03 |
| fam178_va | 6575 | L03 |
| fam178_va | 6576 | P03 |
| fam178_va | 6577 | T03 |
| fam178_va | 6578 | X03 |
| fam178_va | 6579 | AB03 |
| fam178_va | 6580 | AF03 |
| fam178_va | 6581 | D07 |
| fam178_va | 6582 | H07 |
| fam178_va | 6583 | L07 |
| fam178_va | 6584 | P07 |
| fam178_va | 6585 | T07 |
| fam178_va | 6586 | X07 |
| fam178_va | 6587 | AB07 |
| fam178_va | 6588 | AF07 |
| fam178_va | 6589 | D11 |
| fam178_va | 6590 | H11 |
| fam178_va | 6591 | L11 |
| fam178_va | 6592 | P11 |
| fam178_va | 6593 | T11 |
| fam178_va | 6594 | X11 |
| fam178_va | 6595 | AB11 |
| fam178_va | 6596 | AF11 |
| fam178_va | 6597 | D15 |
| fam178_va | 6598 | H15 |
| fam178_va | 6599 | L15 |
| fam178_va | 6600 | P15 |
| fam178_va | 6601 | T15 |
| fam178_va | 6602 | X15 |
| fam178_va | 6603 | AB15 |
| fam178_va | 6604 | AF15 |
| fam178_va | 6605 | D19 |
| fam178_va | 6606 | H19 |
| fam178_va | 6607 | L19 |
| fam178_va | 6608 | P19 |
| fam178_va | 6609 | T19 |
| fam178_va | 6610 | X19 |
| fam178_va | 6611 | AB19 |
| fam178_va | 6612 | AF19 |
| fam178_va | 6613 | D23 |
| fam178_va | 6614 | H23 |
| fam178_va | 6615 | L23 |
| fam178_va | 6616 | P23 |
| fam178_va | 6617 | T23 |
| fam178_va | 6618 | X23 |
| fam178_va | 6619 | AB23 |
| fam178_va | 6620 | AF23 |
| fam178_va | 6621 | D27 |
| fam178_va | 6622 | H27 |
| fam178_va | 6623 | L27 |

| | | |
|---|---|---|
| fam178_va | 6624 | P27 |
| fam178_va | 6625 | T27 |
| fam178_va | 2815 | X27 |
| fam178_va | 2816 | AB27 |
| fam178_va | 2817 | AF27 |
| fam178_va | 2820 | D31 |
| fam178_va | 2821 | H31 |
| fam178_va | 2822 | L31 |
| fam178_va | 3245 | P31 |
| fam178_va | 3246 | T31 |
| fam178_va | 3247 | X31 |
| fam178_va | 3472 | AB31 |
| fam178_va | 3473 | AF31 |
| fam178_va | 3474 | D35 |
| fam178_va | 3327 | H35 |
| fam178_va | 3583 | L35 |
| fam178_va | 3584 | P35 |
| fam178_va | 3601 | T35 |
| fam178_va | 3602 | X35 |
| fam178_va | 3603 | AB35 |
| fam178_va | 3775 | AF35 |
| fam178_va | 3776 | D39 |
| fam178_va | 3777 | H39 |
| fam178_va | 1808 | L39 |
| fam178_va | 1809 | P39 |
| fam178_va | 1812 | T39 |
| fam178_va | 1879 | X39 |
| fam178_va | 1880 | AB39 |
| fam178_va | 1881 | AF39 |
| fam178_va | 2360 | D43 |
| fam178_va | 2361 | H43 |
| fam178_va | 2362 | L43 |
| fam178_va | 2492 | P43 |
| fam178_va | 2493 | T43 |
| fam178_va | 2494 | X43 |
| fam178_va | 2579 | AB43 |
| fam178_va | 2580 | AF43 |
| fam178_va | 2581 | D47 |
| fam178_va | 2602 | H47 |
| fam178_va | 2603 | L47 |
| fam178_va | 2604 | P47 |
| fam178_va | 2846 | T47 |
| fam178_va | 2847 | X47 |
| fam178_va | 2848 | AB47 |
| fam178_va | NTC | AF47 |
| gak_var1 | 6284 | B02 |
| gak_var1 | 6285 | F02 |
| gak_var1 | 6286 | J02 |
| gak_var1 | 6287 | N02 |
| gak_var1 | 6288 | R02 |

| | | |
|---|---|---|
| gak_var1 | 6289 | V02 |
| gak_var1 | 6290 | Z02 |
| gak_var1 | 6291 | AD02 |
| gak_var1 | 6292 | B06 |
| gak_var1 | 6293 | F06 |
| gak_var1 | 6294 | J06 |
| gak_var1 | 6295 | N06 |
| gak_var1 | 6296 | R06 |
| gak_var1 | 6297 | V06 |
| gak_var1 | 6298 | Z06 |
| gak_var1 | 6299 | AD06 |
| gak_var1 | 6300 | B10 |
| gak_var1 | 6301 | F10 |
| gak_var1 | 6302 | J10 |
| gak_var1 | 6303 | N10 |
| gak_var1 | 6304 | R10 |
| gak_var1 | 6305 | V10 |
| gak_var1 | 6306 | Z10 |
| gak_var1 | 6307 | AD10 |
| gak_var1 | 6308 | B14 |
| gak_var1 | 6309 | F14 |
| gak_var1 | 6310 | J14 |
| gak_var1 | 6311 | N14 |
| gak_var1 | 6312 | R14 |
| gak_var1 | 6313 | V14 |
| gak_var1 | 6314 | Z14 |
| gak_var1 | 6315 | AD14 |
| gak_var1 | 6316 | B18 |
| gak_var1 | 6317 | F18 |
| gak_var1 | 6318 | J18 |
| gak_var1 | 6319 | N18 |
| gak_var1 | 6320 | R18 |
| gak_var1 | 6321 | V18 |
| gak_var1 | 6322 | Z18 |
| gak_var1 | 6323 | AD18 |
| gak_var1 | 6324 | B22 |
| gak_var1 | 6325 | F22 |
| gak_var1 | 6326 | J22 |
| gak_var1 | 6327 | N22 |
| gak_var1 | 6328 | R22 |
| gak_var1 | 6329 | V22 |
| gak_var1 | 6330 | Z22 |
| gak_var1 | 6331 | AD22 |
| gak_var1 | 6332 | B26 |
| gak_var1 | 6333 | F26 |
| gak_var1 | 6334 | J26 |
| gak_var1 | 6335 | N26 |
| gak_var1 | 6336 | R26 |
| gak_var1 | 6337 | V26 |
| gak_var1 | 6338 | Z26 |

| | | |
|---|---|---|
| gak_var1 | 6339 | AD26 |
| gak_var1 | 6340 | B30 |
| gak_var1 | 6341 | F30 |
| gak_var1 | 6342 | J30 |
| gak_var1 | 6343 | N30 |
| gak_var1 | 6344 | R30 |
| gak_var1 | 6345 | V30 |
| gak_var1 | 6346 | Z30 |
| gak_var1 | 6347 | AD30 |
| gak_var1 | 6348 | B34 |
| gak_var1 | 6349 | F34 |
| gak_var1 | 6350 | J34 |
| gak_var1 | 6351 | N34 |
| gak_var1 | 6352 | R34 |
| gak_var1 | 6353 | V34 |
| gak_var1 | 6354 | Z34 |
| gak_var1 | 6355 | AD34 |
| gak_var1 | 6356 | B38 |
| gak_var1 | 6357 | F38 |
| gak_var1 | 6358 | J38 |
| gak_var1 | 6359 | N38 |
| gak_var1 | 6360 | R38 |
| gak_var1 | 6361 | V38 |
| gak_var1 | 6362 | Z38 |
| gak_var1 | 6363 | AD38 |
| gak_var1 | 6364 | B42 |
| gak_var1 | 6365 | F42 |
| gak_var1 | 6366 | J42 |
| gak_var1 | 6367 | N42 |
| gak_var1 | 6368 | R42 |
| gak_var1 | 6369 | V42 |
| gak_var1 | 6370 | Z42 |
| gak_var1 | 6371 | AD42 |
| gak_var1 | 6372 | B46 |
| gak_var1 | 6373 | F46 |
| gak_var1 | 6374 | J46 |
| gak_var1 | 6375 | N46 |
| gak_var1 | 6376 | R46 |
| gak_var1 | 6377 | V46 |
| gak_var1 | 6378 | Z46 |
| gak_var1 | 6379 | AD46 |
| gak_var1 | 6380 | B04 |
| gak_var1 | 6381 | F04 |
| gak_var1 | 6382 | J04 |
| gak_var1 | 6383 | N04 |
| gak_var1 | 6384 | R04 |
| gak_var1 | 6385 | V04 |
| gak_var1 | 6386 | Z04 |
| gak_var1 | 6387 | AD04 |
| gak_var1 | 6388 | B08 |

| | | |
|---|---|---|
| gak_var1 | 6389 | F08 |
| gak_var1 | 6390 | J08 |
| gak_var1 | 6391 | N08 |
| gak_var1 | 6392 | R08 |
| gak_var1 | 6393 | V08 |
| gak_var1 | 6394 | Z08 |
| gak_var1 | 6395 | AD08 |
| gak_var1 | 6396 | B12 |
| gak_var1 | 6397 | F12 |
| gak_var1 | 6398 | J12 |
| gak_var1 | 6399 | N12 |
| gak_var1 | 6400 | R12 |
| gak_var1 | 6401 | V12 |
| gak_var1 | 6402 | Z12 |
| gak_var1 | 6403 | AD12 |
| gak_var1 | 6404 | B16 |
| gak_var1 | 6405 | F16 |
| gak_var1 | 6406 | J16 |
| gak_var1 | 6407 | N16 |
| gak_var1 | 6408 | R16 |
| gak_var1 | 6409 | V16 |
| gak_var1 | 6410 | Z16 |
| gak_var1 | 6411 | AD16 |
| gak_var1 | 6412 | B20 |
| gak_var1 | 6413 | F20 |
| gak_var1 | 6414 | J20 |
| gak_var1 | 6415 | N20 |
| gak_var1 | 6416 | R20 |
| gak_var1 | 6417 | V20 |
| gak_var1 | 6418 | Z20 |
| gak_var1 | 6419 | AD20 |
| gak_var1 | 6421 | B24 |
| gak_var1 | 6422 | F24 |
| gak_var1 | 6423 | J24 |
| gak_var1 | 6424 | N24 |
| gak_var1 | 6425 | R24 |
| gak_var1 | 6426 | V24 |
| gak_var1 | 6427 | Z24 |
| gak_var1 | 6428 | AD24 |
| gak_var1 | 6429 | B28 |
| gak_var1 | 6430 | F28 |
| gak_var1 | 6431 | J28 |
| gak_var1 | 6432 | N28 |
| gak_var1 | 6433 | R28 |
| gak_var1 | 6434 | V28 |
| gak_var1 | 6435 | Z28 |
| gak_var1 | 6436 | AD28 |
| gak_var1 | 6437 | B32 |
| gak_var1 | 6438 | F32 |
| gak_var1 | 6439 | J32 |

| | | |
|---|---|---|
| gak_var1 | 6440 | N32 |
| gak_var1 | 6441 | R32 |
| gak_var1 | 6442 | V32 |
| gak_var1 | 6443 | Z32 |
| gak_var1 | 6444 | AD32 |
| gak_var1 | 6445 | B36 |
| gak_var1 | 6446 | F36 |
| gak_var1 | 6447 | J36 |
| gak_var1 | 6448 | N36 |
| gak_var1 | 6449 | R36 |
| gak_var1 | 6450 | V36 |
| gak_var1 | 6451 | Z36 |
| gak_var1 | 6452 | AD36 |
| gak_var1 | 6453 | B40 |
| gak_var1 | 6454 | F40 |
| gak_var1 | 6455 | J40 |
| gak_var1 | 6456 | N40 |
| gak_var1 | 6457 | R40 |
| gak_var1 | 6458 | V40 |
| gak_var1 | 6459 | Z40 |
| gak_var1 | 6460 | AD40 |
| gak_var1 | 6461 | B44 |
| gak_var1 | 6462 | F44 |
| gak_var1 | 6463 | J44 |
| gak_var1 | 6464 | N44 |
| gak_var1 | 6465 | R44 |
| gak_var1 | 6466 | V44 |
| gak_var1 | 6467 | Z44 |
| gak_var1 | 6468 | AD44 |
| gak_var1 | 6469 | B48 |
| gak_var1 | 6470 | F48 |
| gak_var1 | 6471 | J48 |
| gak_var1 | 6472 | N48 |
| gak_var1 | 6473 | R48 |
| gak_var1 | 6474 | V48 |
| gak_var1 | 6475 | Z48 |
| gak_var1 | 6476 | AD48 |
| gak_var1 | 6477 | D02 |
| gak_var1 | 6478 | H02 |
| gak_var1 | 6479 | L02 |
| gak_var1 | 6480 | P02 |
| gak_var1 | 6481 | T02 |
| gak_var1 | 6482 | X02 |
| gak_var1 | 6483 | AB02 |
| gak_var1 | 6484 | AF02 |
| gak_var1 | 6485 | D06 |
| gak_var1 | 6486 | H06 |
| gak_var1 | 6487 | L06 |
| gak_var1 | 6488 | P06 |
| gak_var1 | 6489 | T06 |

| | | |
|---|---|---|
| gak_var1 | 6490 | X06 |
| gak_var1 | 6491 | AB06 |
| gak_var1 | 6492 | AF06 |
| gak_var1 | 6493 | D10 |
| gak_var1 | 6494 | H10 |
| gak_var1 | 6495 | L10 |
| gak_var1 | 6496 | P10 |
| gak_var1 | 6497 | T10 |
| gak_var1 | 6498 | X10 |
| gak_var1 | 6499 | AB10 |
| gak_var1 | 6500 | AF10 |
| gak_var1 | 6501 | D14 |
| gak_var1 | 6502 | H14 |
| gak_var1 | 6503 | L14 |
| gak_var1 | 6504 | P14 |
| gak_var1 | 6505 | T14 |
| gak_var1 | 6506 | X14 |
| gak_var1 | 6507 | AB14 |
| gak_var1 | 6508 | AF14 |
| gak_var1 | 6509 | D18 |
| gak_var1 | 6510 | H18 |
| gak_var1 | 6511 | L18 |
| gak_var1 | 6512 | P18 |
| gak_var1 | 6513 | T18 |
| gak_var1 | 6514 | X18 |
| gak_var1 | 6515 | AB18 |
| gak_var1 | 6516 | AF18 |
| gak_var1 | 6517 | D22 |
| gak_var1 | 6518 | H22 |
| gak_var1 | 6519 | L22 |
| gak_var1 | 6520 | P22 |
| gak_var1 | 6521 | T22 |
| gak_var1 | 6522 | X22 |
| gak_var1 | 6523 | AB22 |
| gak_var1 | 6524 | AF22 |
| gak_var1 | 6525 | D26 |
| gak_var1 | 6526 | H26 |
| gak_var1 | 6527 | L26 |
| gak_var1 | 6528 | P26 |
| gak_var1 | 6529 | T26 |
| gak_var1 | 6530 | X26 |
| gak_var1 | 6531 | AB26 |
| gak_var1 | 6532 | AF26 |
| gak_var1 | 6533 | D30 |
| gak_var1 | 6534 | H30 |
| gak_var1 | 6535 | L30 |
| gak_var1 | 6536 | P30 |
| gak_var1 | 6537 | T30 |
| gak_var1 | 6538 | X30 |
| gak_var1 | 6539 | AB30 |

| | | |
|---|---|---|
| gak_var1 | 6540 | AF30 |
| gak_var1 | 6541 | D34 |
| gak_var1 | 6542 | H34 |
| gak_var1 | 6543 | L34 |
| gak_var1 | 6544 | P34 |
| gak_var1 | 6545 | T34 |
| gak_var1 | 6546 | X34 |
| gak_var1 | 6547 | AB34 |
| gak_var1 | 6548 | AF34 |
| gak_var1 | 6549 | D38 |
| gak_var1 | 6550 | H38 |
| gak_var1 | 6551 | L38 |
| gak_var1 | 6552 | P38 |
| gak_var1 | 6553 | T38 |
| gak_var1 | 6554 | X38 |
| gak_var1 | 6555 | AB38 |
| gak_var1 | 6556 | AF38 |
| gak_var1 | 6557 | D42 |
| gak_var1 | 6558 | H42 |
| gak_var1 | 6559 | L42 |
| gak_var1 | 6560 | P42 |
| gak_var1 | 6561 | T42 |
| gak_var1 | 6562 | X42 |
| gak_var1 | 6563 | AB42 |
| gak_var1 | 6564 | AF42 |
| gak_var1 | 6565 | D46 |
| gak_var1 | 6566 | H46 |
| gak_var1 | 6567 | L46 |
| gak_var1 | 6568 | P46 |
| gak_var1 | 6569 | T46 |
| gak_var1 | 6570 | X46 |
| gak_var1 | 6571 | AB46 |
| gak_var1 | 6572 | AF46 |
| gak_var1 | 6573 | D04 |
| gak_var1 | 6574 | H04 |
| gak_var1 | 6575 | L04 |
| gak_var1 | 6576 | P04 |
| gak_var1 | 6577 | T04 |
| gak_var1 | 6578 | X04 |
| gak_var1 | 6579 | AB04 |
| gak_var1 | 6580 | AF04 |
| gak_var1 | 6581 | D08 |
| gak_var1 | 6582 | H08 |
| gak_var1 | 6583 | L08 |
| gak_var1 | 6584 | P08 |
| gak_var1 | 6585 | T08 |
| gak_var1 | 6586 | X08 |
| gak_var1 | 6587 | AB08 |
| gak_var1 | 6588 | AF08 |
| gak_var1 | 6589 | D12 |

| | | |
|---|---|---|
| gak_var1 | 6590 | H12 |
| gak_var1 | 6591 | L12 |
| gak_var1 | 6592 | P12 |
| gak_var1 | 6593 | T12 |
| gak_var1 | 6594 | X12 |
| gak_var1 | 6595 | AB12 |
| gak_var1 | 6596 | AF12 |
| gak_var1 | 6597 | D16 |
| gak_var1 | 6598 | H16 |
| gak_var1 | 6599 | L16 |
| gak_var1 | 6600 | P16 |
| gak_var1 | 6601 | T16 |
| gak_var1 | 6602 | X16 |
| gak_var1 | 6603 | AB16 |
| gak_var1 | 6604 | AF16 |
| gak_var1 | 6605 | D20 |
| gak_var1 | 6606 | H20 |
| gak_var1 | 6607 | L20 |
| gak_var1 | 6608 | P20 |
| gak_var1 | 6609 | T20 |
| gak_var1 | 6610 | X20 |
| gak_var1 | 6611 | AB20 |
| gak_var1 | 6612 | AF20 |
| gak_var1 | 6613 | D24 |
| gak_var1 | 6614 | H24 |
| gak_var1 | 6615 | L24 |
| gak_var1 | 6616 | P24 |
| gak_var1 | 6617 | T24 |
| gak_var1 | 6618 | X24 |
| gak_var1 | 6619 | AB24 |
| gak_var1 | 6620 | AF24 |
| gak_var1 | 6621 | D28 |
| gak_var1 | 6622 | H28 |
| gak_var1 | 6623 | L28 |
| gak_var1 | 6624 | P28 |
| gak_var1 | 6625 | T28 |
| gak_var1 | 2815 | X28 |
| gak_var1 | 2816 | AB28 |
| gak_var1 | 2817 | AF28 |
| gak_var1 | 2820 | D32 |
| gak_var1 | 2821 | H32 |
| gak_var1 | 2822 | L32 |
| gak_var1 | 3245 | P32 |
| gak_var1 | 3246 | T32 |
| gak_var1 | 3247 | X32 |
| gak_var1 | 3472 | AB32 |
| gak_var1 | 3473 | AF32 |
| gak_var1 | 3474 | D36 |
| gak_var1 | 3327 | H36 |
| gak_var1 | 3583 | L36 |

| | | | |
|---|---|---|---|
| gak_var1 | | 3584 | P36 |
| gak_var1 | | 3601 | T36 |
| gak_var1 | | 3602 | X36 |
| gak_var1 | | 3603 | AB36 |
| gak_var1 | | 3775 | AF36 |
| gak_var1 | | 3776 | D40 |
| gak_var1 | | 3777 | H40 |
| gak_var1 | | 1808 | L40 |
| gak_var1 | | 1809 | P40 |
| gak_var1 | | 1812 | T40 |
| gak_var1 | | 1879 | X40 |
| gak_var1 | | 1880 | AB40 |
| gak_var1 | | 1881 | AF40 |
| gak_var1 | | 2360 | D44 |
| gak_var1 | | 2361 | H44 |
| gak_var1 | | 2362 | L44 |
| gak_var1 | | 2492 | P44 |
| gak_var1 | | 2493 | T44 |
| gak_var1 | | 2494 | X44 |
| gak_var1 | | 2579 | AB44 |
| gak_var1 | | 2580 | AF44 |
| gak_var1 | | 2581 | D48 |
| gak_var1 | | 2602 | H48 |
| gak_var1 | | 2603 | L48 |
| gak_var1 | | 2604 | P48 |
| gak_var1 | | 2846 | T48 |
| gak_var1 | | 2847 | X48 |
| gak_var1 | | 2848 | AB48 |
| gak_var1 | NTC | | AF48 |
| ganab_var1 | | 6284 | A01 |
| ganab_var1 | | 6285 | E01 |
| ganab_var1 | | 6286 | I01 |
| ganab_var1 | | 6287 | M01 |
| ganab_var1 | | 6288 | Q01 |
| ganab_var1 | | 6289 | U01 |
| ganab_var1 | | 6290 | Y01 |
| ganab_var1 | | 6291 | AC01 |
| ganab_var1 | | 6292 | A05 |
| ganab_var1 | | 6293 | E05 |
| ganab_var1 | | 6294 | I05 |
| ganab_var1 | | 6295 | M05 |
| ganab_var1 | | 6296 | Q05 |
| ganab_var1 | | 6297 | U05 |
| ganab_var1 | | 6298 | Y05 |
| ganab_var1 | | 6299 | AC05 |
| ganab_var1 | | 6300 | A09 |
| ganab_var1 | | 6301 | E09 |
| ganab_var1 | | 6302 | I09 |
| ganab_var1 | | 6303 | M09 |
| ganab_var1 | | 6304 | Q09 |

| | | |
|---|---|---|
| ganab_var1 | 6305 | U09 |
| ganab_var1 | 6306 | Y09 |
| ganab_var1 | 6307 | AC09 |
| ganab_var1 | 6308 | A13 |
| ganab_var1 | 6309 | E13 |
| ganab_var1 | 6310 | I13 |
| ganab_var1 | 6311 | M13 |
| ganab_var1 | 6312 | Q13 |
| ganab_var1 | 6313 | U13 |
| ganab_var1 | 6314 | Y13 |
| ganab_var1 | 6315 | AC13 |
| ganab_var1 | 6316 | A17 |
| ganab_var1 | 6317 | E17 |
| ganab_var1 | 6318 | I17 |
| ganab_var1 | 6319 | M17 |
| ganab_var1 | 6320 | Q17 |
| ganab_var1 | 6321 | U17 |
| ganab_var1 | 6322 | Y17 |
| ganab_var1 | 6323 | AC17 |
| ganab_var1 | 6324 | A21 |
| ganab_var1 | 6325 | E21 |
| ganab_var1 | 6326 | I21 |
| ganab_var1 | 6327 | M21 |
| ganab_var1 | 6328 | Q21 |
| ganab_var1 | 6329 | U21 |
| ganab_var1 | 6330 | Y21 |
| ganab_var1 | 6331 | AC21 |
| ganab_var1 | 6332 | A25 |
| ganab_var1 | 6333 | E25 |
| ganab_var1 | 6334 | I25 |
| ganab_var1 | 6335 | M25 |
| ganab_var1 | 6336 | Q25 |
| ganab_var1 | 6337 | U25 |
| ganab_var1 | 6338 | Y25 |
| ganab_var1 | 6339 | AC25 |
| ganab_var1 | 6340 | A29 |
| ganab_var1 | 6341 | E29 |
| ganab_var1 | 6342 | I29 |
| ganab_var1 | 6343 | M29 |
| ganab_var1 | 6344 | Q29 |
| ganab_var1 | 6345 | U29 |
| ganab_var1 | 6346 | Y29 |
| ganab_var1 | 6347 | AC29 |
| ganab_var1 | 6348 | A33 |
| ganab_var1 | 6349 | E33 |
| ganab_var1 | 6350 | I33 |
| ganab_var1 | 6351 | M33 |
| ganab_var1 | 6352 | Q33 |
| ganab_var1 | 6353 | U33 |
| ganab_var1 | 6354 | Y33 |

| | | |
|---|---|---|
| ganab_var1 | 6355 | AC33 |
| ganab_var1 | 6356 | A37 |
| ganab_var1 | 6357 | E37 |
| ganab_var1 | 6358 | I37 |
| ganab_var1 | 6359 | M37 |
| ganab_var1 | 6360 | Q37 |
| ganab_var1 | 6361 | U37 |
| ganab_var1 | 6362 | Y37 |
| ganab_var1 | 6363 | AC37 |
| ganab_var1 | 6364 | A41 |
| ganab_var1 | 6365 | E41 |
| ganab_var1 | 6366 | I41 |
| ganab_var1 | 6367 | M41 |
| ganab_var1 | 6368 | Q41 |
| ganab_var1 | 6369 | U41 |
| ganab_var1 | 6370 | Y41 |
| ganab_var1 | 6371 | AC41 |
| ganab_var1 | 6372 | A45 |
| ganab_var1 | 6373 | E45 |
| ganab_var1 | 6374 | I45 |
| ganab_var1 | 6375 | M45 |
| ganab_var1 | 6376 | Q45 |
| ganab_var1 | 6377 | U45 |
| ganab_var1 | 6378 | Y45 |
| ganab_var1 | 6379 | AC45 |
| ganab_var1 | 6380 | A03 |
| ganab_var1 | 6381 | E03 |
| ganab_var1 | 6382 | I03 |
| ganab_var1 | 6383 | M03 |
| ganab_var1 | 6384 | Q03 |
| ganab_var1 | 6385 | U03 |
| ganab_var1 | 6386 | Y03 |
| ganab_var1 | 6387 | AC03 |
| ganab_var1 | 6388 | A07 |
| ganab_var1 | 6389 | E07 |
| ganab_var1 | 6390 | I07 |
| ganab_var1 | 6391 | M07 |
| ganab_var1 | 6392 | Q07 |
| ganab_var1 | 6393 | U07 |
| ganab_var1 | 6394 | Y07 |
| ganab_var1 | 6395 | AC07 |
| ganab_var1 | 6396 | A11 |
| ganab_var1 | 6397 | E11 |
| ganab_var1 | 6398 | I11 |
| ganab_var1 | 6399 | M11 |
| ganab_var1 | 6400 | Q11 |
| ganab_var1 | 6401 | U11 |
| ganab_var1 | 6402 | Y11 |
| ganab_var1 | 6403 | AC11 |
| ganab_var1 | 6404 | A15 |

| | | |
|---|---|---|
| ganab_var1 | 6405 | E15 |
| ganab_var1 | 6406 | I15 |
| ganab_var1 | 6407 | M15 |
| ganab_var1 | 6408 | Q15 |
| ganab_var1 | 6409 | U15 |
| ganab_var1 | 6410 | Y15 |
| ganab_var1 | 6411 | AC15 |
| ganab_var1 | 6412 | A19 |
| ganab_var1 | 6413 | E19 |
| ganab_var1 | 6414 | I19 |
| ganab_var1 | 6415 | M19 |
| ganab_var1 | 6416 | Q19 |
| ganab_var1 | 6417 | U19 |
| ganab_var1 | 6418 | Y19 |
| ganab_var1 | 6419 | AC19 |
| ganab_var1 | 6421 | A23 |
| ganab_var1 | 6422 | E23 |
| ganab_var1 | 6423 | I23 |
| ganab_var1 | 6424 | M23 |
| ganab_var1 | 6425 | Q23 |
| ganab_var1 | 6426 | U23 |
| ganab_var1 | 6427 | Y23 |
| ganab_var1 | 6428 | AC23 |
| ganab_var1 | 6429 | A27 |
| ganab_var1 | 6430 | E27 |
| ganab_var1 | 6431 | I27 |
| ganab_var1 | 6432 | M27 |
| ganab_var1 | 6433 | Q27 |
| ganab_var1 | 6434 | U27 |
| ganab_var1 | 6435 | Y27 |
| ganab_var1 | 6436 | AC27 |
| ganab_var1 | 6437 | A31 |
| ganab_var1 | 6438 | E31 |
| ganab_var1 | 6439 | I31 |
| ganab_var1 | 6440 | M31 |
| ganab_var1 | 6441 | Q31 |
| ganab_var1 | 6442 | U31 |
| ganab_var1 | 6443 | Y31 |
| ganab_var1 | 6444 | AC31 |
| ganab_var1 | 6445 | A35 |
| ganab_var1 | 6446 | E35 |
| ganab_var1 | 6447 | I35 |
| ganab_var1 | 6448 | M35 |
| ganab_var1 | 6449 | Q35 |
| ganab_var1 | 6450 | U35 |
| ganab_var1 | 6451 | Y35 |
| ganab_var1 | 6452 | AC35 |
| ganab_var1 | 6453 | A39 |
| ganab_var1 | 6454 | E39 |
| ganab_var1 | 6455 | I39 |

| | | |
|---|---|---|
| ganab_var1 | 6456 | M39 |
| ganab_var1 | 6457 | Q39 |
| ganab_var1 | 6458 | U39 |
| ganab_var1 | 6459 | Y39 |
| ganab_var1 | 6460 | AC39 |
| ganab_var1 | 6461 | A43 |
| ganab_var1 | 6462 | E43 |
| ganab_var1 | 6463 | I43 |
| ganab_var1 | 6464 | M43 |
| ganab_var1 | 6465 | Q43 |
| ganab_var1 | 6466 | U43 |
| ganab_var1 | 6467 | Y43 |
| ganab_var1 | 6468 | AC43 |
| ganab_var1 | 6469 | A47 |
| ganab_var1 | 6470 | E47 |
| ganab_var1 | 6471 | I47 |
| ganab_var1 | 6472 | M47 |
| ganab_var1 | 6473 | Q47 |
| ganab_var1 | 6474 | U47 |
| ganab_var1 | 6475 | Y47 |
| ganab_var1 | 6476 | AC47 |
| ganab_var1 | 6477 | C01 |
| ganab_var1 | 6478 | G01 |
| ganab_var1 | 6479 | K01 |
| ganab_var1 | 6480 | O01 |
| ganab_var1 | 6481 | S01 |
| ganab_var1 | 6482 | W01 |
| ganab_var1 | 6483 | AA01 |
| ganab_var1 | 6484 | AE01 |
| ganab_var1 | 6485 | C05 |
| ganab_var1 | 6486 | G05 |
| ganab_var1 | 6487 | K05 |
| ganab_var1 | 6488 | O05 |
| ganab_var1 | 6489 | S05 |
| ganab_var1 | 6490 | W05 |
| ganab_var1 | 6491 | AA05 |
| ganab_var1 | 6492 | AE05 |
| ganab_var1 | 6493 | C09 |
| ganab_var1 | 6494 | G09 |
| ganab_var1 | 6495 | K09 |
| ganab_var1 | 6496 | O09 |
| ganab_var1 | 6497 | S09 |
| ganab_var1 | 6498 | W09 |
| ganab_var1 | 6499 | AA09 |
| ganab_var1 | 6500 | AE09 |
| ganab_var1 | 6501 | C13 |
| ganab_var1 | 6502 | G13 |
| ganab_var1 | 6503 | K13 |
| ganab_var1 | 6504 | O13 |
| ganab_var1 | 6505 | S13 |

| | | |
|---|---|---|
| ganab_var1 | 6506 | W13 |
| ganab_var1 | 6507 | AA13 |
| ganab_var1 | 6508 | AE13 |
| ganab_var1 | 6509 | C17 |
| ganab_var1 | 6510 | G17 |
| ganab_var1 | 6511 | K17 |
| ganab_var1 | 6512 | O17 |
| ganab_var1 | 6513 | S17 |
| ganab_var1 | 6514 | W17 |
| ganab_var1 | 6515 | AA17 |
| ganab_var1 | 6516 | AE17 |
| ganab_var1 | 6517 | C21 |
| ganab_var1 | 6518 | G21 |
| ganab_var1 | 6519 | K21 |
| ganab_var1 | 6520 | O21 |
| ganab_var1 | 6521 | S21 |
| ganab_var1 | 6522 | W21 |
| ganab_var1 | 6523 | AA21 |
| ganab_var1 | 6524 | AE21 |
| ganab_var1 | 6525 | C25 |
| ganab_var1 | 6526 | G25 |
| ganab_var1 | 6527 | K25 |
| ganab_var1 | 6528 | O25 |
| ganab_var1 | 6529 | S25 |
| ganab_var1 | 6530 | W25 |
| ganab_var1 | 6531 | AA25 |
| ganab_var1 | 6532 | AE25 |
| ganab_var1 | 6533 | C29 |
| ganab_var1 | 6534 | G29 |
| ganab_var1 | 6535 | K29 |
| ganab_var1 | 6536 | O29 |
| ganab_var1 | 6537 | S29 |
| ganab_var1 | 6538 | W29 |
| ganab_var1 | 6539 | AA29 |
| ganab_var1 | 6540 | AE29 |
| ganab_var1 | 6541 | C33 |
| ganab_var1 | 6542 | G33 |
| ganab_var1 | 6543 | K33 |
| ganab_var1 | 6544 | O33 |
| ganab_var1 | 6545 | S33 |
| ganab_var1 | 6546 | W33 |
| ganab_var1 | 6547 | AA33 |
| ganab_var1 | 6548 | AE33 |
| ganab_var1 | 6549 | C37 |
| ganab_var1 | 6550 | G37 |
| ganab_var1 | 6551 | K37 |
| ganab_var1 | 6552 | O37 |
| ganab_var1 | 6553 | S37 |
| ganab_var1 | 6554 | W37 |
| ganab_var1 | 6555 | AA37 |

| | | |
|---|---|---|
| ganab_var1 | 6556 | AE37 |
| ganab_var1 | 6557 | C41 |
| ganab_var1 | 6558 | G41 |
| ganab_var1 | 6559 | K41 |
| ganab_var1 | 6560 | O41 |
| ganab_var1 | 6561 | S41 |
| ganab_var1 | 6562 | W41 |
| ganab_var1 | 6563 | AA41 |
| ganab_var1 | 6564 | AE41 |
| ganab_var1 | 6565 | C45 |
| ganab_var1 | 6566 | G45 |
| ganab_var1 | 6567 | K45 |
| ganab_var1 | 6568 | O45 |
| ganab_var1 | 6569 | S45 |
| ganab_var1 | 6570 | W45 |
| ganab_var1 | 6571 | AA45 |
| ganab_var1 | 6572 | AE45 |
| ganab_var1 | 6573 | C03 |
| ganab_var1 | 6574 | G03 |
| ganab_var1 | 6575 | K03 |
| ganab_var1 | 6576 | O03 |
| ganab_var1 | 6577 | S03 |
| ganab_var1 | 6578 | W03 |
| ganab_var1 | 6579 | AA03 |
| ganab_var1 | 6580 | AE03 |
| ganab_var1 | 6581 | C07 |
| ganab_var1 | 6582 | G07 |
| ganab_var1 | 6583 | K07 |
| ganab_var1 | 6584 | O07 |
| ganab_var1 | 6585 | S07 |
| ganab_var1 | 6586 | W07 |
| ganab_var1 | 6587 | AA07 |
| ganab_var1 | 6588 | AE07 |
| ganab_var1 | 6589 | C11 |
| ganab_var1 | 6590 | G11 |
| ganab_var1 | 6591 | K11 |
| ganab_var1 | 6592 | O11 |
| ganab_var1 | 6593 | S11 |
| ganab_var1 | 6594 | W11 |
| ganab_var1 | 6595 | AA11 |
| ganab_var1 | 6596 | AE11 |
| ganab_var1 | 6597 | C15 |
| ganab_var1 | 6598 | G15 |
| ganab_var1 | 6599 | K15 |
| ganab_var1 | 6600 | O15 |
| ganab_var1 | 6601 | S15 |
| ganab_var1 | 6602 | W15 |
| ganab_var1 | 6603 | AA15 |
| ganab_var1 | 6604 | AE15 |
| ganab_var1 | 6605 | C19 |

| | | |
|---|---|---|
| ganab_var1 | 6606 | G19 |
| ganab_var1 | 6607 | K19 |
| ganab_var1 | 6608 | O19 |
| ganab_var1 | 6609 | S19 |
| ganab_var1 | 6610 | W19 |
| ganab_var1 | 6611 | AA19 |
| ganab_var1 | 6612 | AE19 |
| ganab_var1 | 6613 | C23 |
| ganab_var1 | 6614 | G23 |
| ganab_var1 | 6615 | K23 |
| ganab_var1 | 6616 | O23 |
| ganab_var1 | 6617 | S23 |
| ganab_var1 | 6618 | W23 |
| ganab_var1 | 6619 | AA23 |
| ganab_var1 | 6620 | AE23 |
| ganab_var1 | 6621 | C27 |
| ganab_var1 | 6622 | G27 |
| ganab_var1 | 6623 | K27 |
| ganab_var1 | 6624 | O27 |
| ganab_var1 | 6625 | S27 |
| ganab_var1 | 2815 | W27 |
| ganab_var1 | 2816 | AA27 |
| ganab_var1 | 2817 | AE27 |
| ganab_var1 | 2820 | C31 |
| ganab_var1 | 2821 | G31 |
| ganab_var1 | 2822 | K31 |
| ganab_var1 | 3245 | O31 |
| ganab_var1 | 3246 | S31 |
| ganab_var1 | 3247 | W31 |
| ganab_var1 | 3472 | AA31 |
| ganab_var1 | 3473 | AE31 |
| ganab_var1 | 3474 | C35 |
| ganab_var1 | 3327 | G35 |
| ganab_var1 | 3583 | K35 |
| ganab_var1 | 3584 | O35 |
| ganab_var1 | 3601 | S35 |
| ganab_var1 | 3602 | W35 |
| ganab_var1 | 3603 | AA35 |
| ganab_var1 | 3775 | AE35 |
| ganab_var1 | 3776 | C39 |
| ganab_var1 | 3777 | G39 |
| ganab_var1 | 1808 | K39 |
| ganab_var1 | 1809 | O39 |
| ganab_var1 | 1812 | S39 |
| ganab_var1 | 1879 | W39 |
| ganab_var1 | 1880 | AA39 |
| ganab_var1 | 1881 | AE39 |
| ganab_var1 | 2360 | C43 |
| ganab_var1 | 2361 | G43 |
| ganab_var1 | 2362 | K43 |

| | | |
|---|---|---|
| ganab_var1 | 2492 | O43 |
| ganab_var1 | 2493 | S43 |
| ganab_var1 | 2494 | W43 |
| ganab_var1 | 2579 | AA43 |
| ganab_var1 | 2580 | AE43 |
| ganab_var1 | 2581 | C47 |
| ganab_var1 | 2602 | G47 |
| ganab_var1 | 2603 | K47 |
| ganab_var1 | 2604 | O47 |
| ganab_var1 | 2846 | S47 |
| ganab_var1 | 2847 | W47 |
| ganab_var1 | 2848 | AA47 |
| ganab_var1 | NTC | AE47 |
| hmp19_var | 6284 | A02 |
| hmp19_var | 6285 | E02 |
| hmp19_var | 6286 | I02 |
| hmp19_var | 6287 | M02 |
| hmp19_var | 6288 | Q02 |
| hmp19_var | 6289 | U02 |
| hmp19_var | 6290 | Y02 |
| hmp19_var | 6291 | AC02 |
| hmp19_var | 6292 | A06 |
| hmp19_var | 6293 | E06 |
| hmp19_var | 6294 | I06 |
| hmp19_var | 6295 | M06 |
| hmp19_var | 6296 | Q06 |
| hmp19_var | 6297 | U06 |
| hmp19_var | 6298 | Y06 |
| hmp19_var | 6299 | AC06 |
| hmp19_var | 6300 | A10 |
| hmp19_var | 6301 | E10 |
| hmp19_var | 6302 | I10 |
| hmp19_var | 6303 | M10 |
| hmp19_var | 6304 | Q10 |
| hmp19_var | 6305 | U10 |
| hmp19_var | 6306 | Y10 |
| hmp19_var | 6307 | AC10 |
| hmp19_var | 6308 | A14 |
| hmp19_var | 6309 | E14 |
| hmp19_var | 6310 | I14 |
| hmp19_var | 6311 | M14 |
| hmp19_var | 6312 | Q14 |
| hmp19_var | 6313 | U14 |
| hmp19_var | 6314 | Y14 |
| hmp19_var | 6315 | AC14 |
| hmp19_var | 6316 | A18 |
| hmp19_var | 6317 | E18 |
| hmp19_var | 6318 | I18 |
| hmp19_var | 6319 | M18 |
| hmp19_var | 6320 | Q18 |

| | | |
|---|---|---|
| hmp19_vai | 6321 | U18 |
| hmp19_vai | 6322 | Y18 |
| hmp19_vai | 6323 | AC18 |
| hmp19_vai | 6324 | A22 |
| hmp19_vai | 6325 | E22 |
| hmp19_vai | 6326 | I22 |
| hmp19_vai | 6327 | M22 |
| hmp19_vai | 6328 | Q22 |
| hmp19_vai | 6329 | U22 |
| hmp19_vai | 6330 | Y22 |
| hmp19_vai | 6331 | AC22 |
| hmp19_vai | 6332 | A26 |
| hmp19_vai | 6333 | E26 |
| hmp19_vai | 6334 | I26 |
| hmp19_vai | 6335 | M26 |
| hmp19_vai | 6336 | Q26 |
| hmp19_vai | 6337 | U26 |
| hmp19_vai | 6338 | Y26 |
| hmp19_vai | 6339 | AC26 |
| hmp19_vai | 6340 | A30 |
| hmp19_vai | 6341 | E30 |
| hmp19_vai | 6342 | I30 |
| hmp19_vai | 6343 | M30 |
| hmp19_vai | 6344 | Q30 |
| hmp19_vai | 6345 | U30 |
| hmp19_vai | 6346 | Y30 |
| hmp19_vai | 6347 | AC30 |
| hmp19_vai | 6348 | A34 |
| hmp19_vai | 6349 | E34 |
| hmp19_vai | 6350 | I34 |
| hmp19_vai | 6351 | M34 |
| hmp19_vai | 6352 | Q34 |
| hmp19_vai | 6353 | U34 |
| hmp19_vai | 6354 | Y34 |
| hmp19_vai | 6355 | AC34 |
| hmp19_vai | 6356 | A38 |
| hmp19_vai | 6357 | E38 |
| hmp19_vai | 6358 | I38 |
| hmp19_vai | 6359 | M38 |
| hmp19_vai | 6360 | Q38 |
| hmp19_vai | 6361 | U38 |
| hmp19_vai | 6362 | Y38 |
| hmp19_vai | 6363 | AC38 |
| hmp19_vai | 6364 | A42 |
| hmp19_vai | 6365 | E42 |
| hmp19_vai | 6366 | I42 |
| hmp19_vai | 6367 | M42 |
| hmp19_vai | 6368 | Q42 |
| hmp19_vai | 6369 | U42 |
| hmp19_vai | 6370 | Y42 |

| | | |
|---|---|---|
| hmp19_var | 6371 | AC42 |
| hmp19_var | 6372 | A46 |
| hmp19_var | 6373 | E46 |
| hmp19_var | 6374 | I46 |
| hmp19_var | 6375 | M46 |
| hmp19_var | 6376 | Q46 |
| hmp19_var | 6377 | U46 |
| hmp19_var | 6378 | Y46 |
| hmp19_var | 6379 | AC46 |
| hmp19_var | 6380 | A04 |
| hmp19_var | 6381 | E04 |
| hmp19_var | 6382 | I04 |
| hmp19_var | 6383 | M04 |
| hmp19_var | 6384 | Q04 |
| hmp19_var | 6385 | U04 |
| hmp19_var | 6386 | Y04 |
| hmp19_var | 6387 | AC04 |
| hmp19_var | 6388 | A08 |
| hmp19_var | 6389 | E08 |
| hmp19_var | 6390 | I08 |
| hmp19_var | 6391 | M08 |
| hmp19_var | 6392 | Q08 |
| hmp19_var | 6393 | U08 |
| hmp19_var | 6394 | Y08 |
| hmp19_var | 6395 | AC08 |
| hmp19_var | 6396 | A12 |
| hmp19_var | 6397 | E12 |
| hmp19_var | 6398 | I12 |
| hmp19_var | 6399 | M12 |
| hmp19_var | 6400 | Q12 |
| hmp19_var | 6401 | U12 |
| hmp19_var | 6402 | Y12 |
| hmp19_var | 6403 | AC12 |
| hmp19_var | 6404 | A16 |
| hmp19_var | 6405 | E16 |
| hmp19_var | 6406 | I16 |
| hmp19_var | 6407 | M16 |
| hmp19_var | 6408 | Q16 |
| hmp19_var | 6409 | U16 |
| hmp19_var | 6410 | Y16 |
| hmp19_var | 6411 | AC16 |
| hmp19_var | 6412 | A20 |
| hmp19_var | 6413 | E20 |
| hmp19_var | 6414 | I20 |
| hmp19_var | 6415 | M20 |
| hmp19_var | 6416 | Q20 |
| hmp19_var | 6417 | U20 |
| hmp19_var | 6418 | Y20 |
| hmp19_var | 6419 | AC20 |
| hmp19_var | 6421 | A24 |

| | | |
|---|---|---|
| hmp19_vai | 6422 | E24 |
| hmp19_vai | 6423 | I24 |
| hmp19_vai | 6424 | M24 |
| hmp19_vai | 6425 | Q24 |
| hmp19_vai | 6426 | U24 |
| hmp19_vai | 6427 | Y24 |
| hmp19_vai | 6428 | AC24 |
| hmp19_vai | 6429 | A28 |
| hmp19_vai | 6430 | E28 |
| hmp19_vai | 6431 | I28 |
| hmp19_vai | 6432 | M28 |
| hmp19_vai | 6433 | Q28 |
| hmp19_vai | 6434 | U28 |
| hmp19_vai | 6435 | Y28 |
| hmp19_vai | 6436 | AC28 |
| hmp19_vai | 6437 | A32 |
| hmp19_vai | 6438 | E32 |
| hmp19_vai | 6439 | I32 |
| hmp19_vai | 6440 | M32 |
| hmp19_vai | 6441 | Q32 |
| hmp19_vai | 6442 | U32 |
| hmp19_vai | 6443 | Y32 |
| hmp19_vai | 6444 | AC32 |
| hmp19_vai | 6445 | A36 |
| hmp19_vai | 6446 | E36 |
| hmp19_vai | 6447 | I36 |
| hmp19_vai | 6448 | M36 |
| hmp19_vai | 6449 | Q36 |
| hmp19_vai | 6450 | U36 |
| hmp19_vai | 6451 | Y36 |
| hmp19_vai | 6452 | AC36 |
| hmp19_vai | 6453 | A40 |
| hmp19_vai | 6454 | E40 |
| hmp19_vai | 6455 | I40 |
| hmp19_vai | 6456 | M40 |
| hmp19_vai | 6457 | Q40 |
| hmp19_vai | 6458 | U40 |
| hmp19_vai | 6459 | Y40 |
| hmp19_vai | 6460 | AC40 |
| hmp19_vai | 6461 | A44 |
| hmp19_vai | 6462 | E44 |
| hmp19_vai | 6463 | I44 |
| hmp19_vai | 6464 | M44 |
| hmp19_vai | 6465 | Q44 |
| hmp19_vai | 6466 | U44 |
| hmp19_vai | 6467 | Y44 |
| hmp19_vai | 6468 | AC44 |
| hmp19_vai | 6469 | A48 |
| hmp19_vai | 6470 | E48 |
| hmp19_vai | 6471 | I48 |

| | | |
|---|---|---|
| hmp19_var | 6472 | M48 |
| hmp19_var | 6473 | Q48 |
| hmp19_var | 6474 | U48 |
| hmp19_var | 6475 | Y48 |
| hmp19_var | 6476 | AC48 |
| hmp19_var | 6477 | C02 |
| hmp19_var | 6478 | G02 |
| hmp19_var | 6479 | K02 |
| hmp19_var | 6480 | O02 |
| hmp19_var | 6481 | S02 |
| hmp19_var | 6482 | W02 |
| hmp19_var | 6483 | AA02 |
| hmp19_var | 6484 | AE02 |
| hmp19_var | 6485 | C06 |
| hmp19_var | 6486 | G06 |
| hmp19_var | 6487 | K06 |
| hmp19_var | 6488 | O06 |
| hmp19_var | 6489 | S06 |
| hmp19_var | 6490 | W06 |
| hmp19_var | 6491 | AA06 |
| hmp19_var | 6492 | AE06 |
| hmp19_var | 6493 | C10 |
| hmp19_var | 6494 | G10 |
| hmp19_var | 6495 | K10 |
| hmp19_var | 6496 | O10 |
| hmp19_var | 6497 | S10 |
| hmp19_var | 6498 | W10 |
| hmp19_var | 6499 | AA10 |
| hmp19_var | 6500 | AE10 |
| hmp19_var | 6501 | C14 |
| hmp19_var | 6502 | G14 |
| hmp19_var | 6503 | K14 |
| hmp19_var | 6504 | O14 |
| hmp19_var | 6505 | S14 |
| hmp19_var | 6506 | W14 |
| hmp19_var | 6507 | AA14 |
| hmp19_var | 6508 | AE14 |
| hmp19_var | 6509 | C18 |
| hmp19_var | 6510 | G18 |
| hmp19_var | 6511 | K18 |
| hmp19_var | 6512 | O18 |
| hmp19_var | 6513 | S18 |
| hmp19_var | 6514 | W18 |
| hmp19_var | 6515 | AA18 |
| hmp19_var | 6516 | AE18 |
| hmp19_var | 6517 | C22 |
| hmp19_var | 6518 | G22 |
| hmp19_var | 6519 | K22 |
| hmp19_var | 6520 | O22 |
| hmp19_var | 6521 | S22 |

| | | |
|---|---|---|
| hmp19_vai | 6522 | W22 |
| hmp19_vai | 6523 | AA22 |
| hmp19_vai | 6524 | AE22 |
| hmp19_vai | 6525 | C26 |
| hmp19_vai | 6526 | G26 |
| hmp19_vai | 6527 | K26 |
| hmp19_vai | 6528 | O26 |
| hmp19_vai | 6529 | S26 |
| hmp19_vai | 6530 | W26 |
| hmp19_vai | 6531 | AA26 |
| hmp19_vai | 6532 | AE26 |
| hmp19_vai | 6533 | C30 |
| hmp19_vai | 6534 | G30 |
| hmp19_vai | 6535 | K30 |
| hmp19_vai | 6536 | O30 |
| hmp19_vai | 6537 | S30 |
| hmp19_vai | 6538 | W30 |
| hmp19_vai | 6539 | AA30 |
| hmp19_vai | 6540 | AE30 |
| hmp19_vai | 6541 | C34 |
| hmp19_vai | 6542 | G34 |
| hmp19_vai | 6543 | K34 |
| hmp19_vai | 6544 | O34 |
| hmp19_vai | 6545 | S34 |
| hmp19_vai | 6546 | W34 |
| hmp19_vai | 6547 | AA34 |
| hmp19_vai | 6548 | AE34 |
| hmp19_vai | 6549 | C38 |
| hmp19_vai | 6550 | G38 |
| hmp19_vai | 6551 | K38 |
| hmp19_vai | 6552 | O38 |
| hmp19_vai | 6553 | S38 |
| hmp19_vai | 6554 | W38 |
| hmp19_vai | 6555 | AA38 |
| hmp19_vai | 6556 | AE38 |
| hmp19_vai | 6557 | C42 |
| hmp19_vai | 6558 | G42 |
| hmp19_vai | 6559 | K42 |
| hmp19_vai | 6560 | O42 |
| hmp19_vai | 6561 | S42 |
| hmp19_vai | 6562 | W42 |
| hmp19_vai | 6563 | AA42 |
| hmp19_vai | 6564 | AE42 |
| hmp19_vai | 6565 | C46 |
| hmp19_vai | 6566 | G46 |
| hmp19_vai | 6567 | K46 |
| hmp19_vai | 6568 | O46 |
| hmp19_vai | 6569 | S46 |
| hmp19_vai | 6570 | W46 |
| hmp19_vai | 6571 | AA46 |

| | | |
|---|---|---|
| hmp19_vai | 6572 | AE46 |
| hmp19_vai | 6573 | C04 |
| hmp19_vai | 6574 | G04 |
| hmp19_vai | 6575 | K04 |
| hmp19_vai | 6576 | O04 |
| hmp19_vai | 6577 | S04 |
| hmp19_vai | 6578 | W04 |
| hmp19_vai | 6579 | AA04 |
| hmp19_vai | 6580 | AE04 |
| hmp19_vai | 6581 | C08 |
| hmp19_vai | 6582 | G08 |
| hmp19_vai | 6583 | K08 |
| hmp19_vai | 6584 | O08 |
| hmp19_vai | 6585 | S08 |
| hmp19_vai | 6586 | W08 |
| hmp19_vai | 6587 | AA08 |
| hmp19_vai | 6588 | AE08 |
| hmp19_vai | 6589 | C12 |
| hmp19_vai | 6590 | G12 |
| hmp19_vai | 6591 | K12 |
| hmp19_vai | 6592 | O12 |
| hmp19_vai | 6593 | S12 |
| hmp19_vai | 6594 | W12 |
| hmp19_vai | 6595 | AA12 |
| hmp19_vai | 6596 | AE12 |
| hmp19_vai | 6597 | C16 |
| hmp19_vai | 6598 | G16 |
| hmp19_vai | 6599 | K16 |
| hmp19_vai | 6600 | O16 |
| hmp19_vai | 6601 | S16 |
| hmp19_vai | 6602 | W16 |
| hmp19_vai | 6603 | AA16 |
| hmp19_vai | 6604 | AE16 |
| hmp19_vai | 6605 | C20 |
| hmp19_vai | 6606 | G20 |
| hmp19_vai | 6607 | K20 |
| hmp19_vai | 6608 | O20 |
| hmp19_vai | 6609 | S20 |
| hmp19_vai | 6610 | W20 |
| hmp19_vai | 6611 | AA20 |
| hmp19_vai | 6612 | AE20 |
| hmp19_vai | 6613 | C24 |
| hmp19_vai | 6614 | G24 |
| hmp19_vai | 6615 | K24 |
| hmp19_vai | 6616 | O24 |
| hmp19_vai | 6617 | S24 |
| hmp19_vai | 6618 | W24 |
| hmp19_vai | 6619 | AA24 |
| hmp19_vai | 6620 | AE24 |
| hmp19_vai | 6621 | C28 |

| | | |
|---|---|---|
| hmp19_vaı | 6622 | G28 |
| hmp19_vaı | 6623 | K28 |
| hmp19_vaı | 6624 | O28 |
| hmp19_vaı | 6625 | S28 |
| hmp19_vaı | 2815 | W28 |
| hmp19_vaı | 2816 | AA28 |
| hmp19_vaı | 2817 | AE28 |
| hmp19_vaı | 2820 | C32 |
| hmp19_vaı | 2821 | G32 |
| hmp19_vaı | 2822 | K32 |
| hmp19_vaı | 3245 | O32 |
| hmp19_vaı | 3246 | S32 |
| hmp19_vaı | 3247 | W32 |
| hmp19_vaı | 3472 | AA32 |
| hmp19_vaı | 3473 | AE32 |
| hmp19_vaı | 3474 | C36 |
| hmp19_vaı | 3327 | G36 |
| hmp19_vaı | 3583 | K36 |
| hmp19_vaı | 3584 | O36 |
| hmp19_vaı | 3601 | S36 |
| hmp19_vaı | 3602 | W36 |
| hmp19_vaı | 3603 | AA36 |
| hmp19_vaı | 3775 | AE36 |
| hmp19_vaı | 3776 | C40 |
| hmp19_vaı | 3777 | G40 |
| hmp19_vaı | 1808 | K40 |
| hmp19_vaı | 1809 | O40 |
| hmp19_vaı | 1812 | S40 |
| hmp19_vaı | 1879 | W40 |
| hmp19_vaı | 1880 | AA40 |
| hmp19_vaı | 1881 | AE40 |
| hmp19_vaı | 2360 | C44 |
| hmp19_vaı | 2361 | G44 |
| hmp19_vaı | 2362 | K44 |
| hmp19_vaı | 2492 | O44 |
| hmp19_vaı | 2493 | S44 |
| hmp19_vaı | 2494 | W44 |
| hmp19_vaı | 2579 | AA44 |
| hmp19_vaı | 2580 | AE44 |
| hmp19_vaı | 2581 | C48 |
| hmp19_vaı | 2602 | G48 |
| hmp19_vaı | 2603 | K48 |
| hmp19_vaı | 2604 | O48 |
| hmp19_vaı | 2846 | S48 |
| hmp19_vaı | 2847 | W48 |
| hmp19_vaı | 2848 | AA48 |
| hmp19_vaı NTC | | AE48 |
| msantd4_v | 6284 | B01 |
| msantd4_v | 6285 | F01 |
| msantd4_v | 6286 | J01 |

| | | |
|---|---|---|
| msantd4_v | 6287 | N01 |
| msantd4_v | 6288 | R01 |
| msantd4_v | 6289 | V01 |
| msantd4_v | 6290 | Z01 |
| msantd4_v | 6291 | AD01 |
| msantd4_v | 6292 | B05 |
| msantd4_v | 6293 | F05 |
| msantd4_v | 6294 | J05 |
| msantd4_v | 6295 | N05 |
| msantd4_v | 6296 | R05 |
| msantd4_v | 6297 | V05 |
| msantd4_v | 6298 | Z05 |
| msantd4_v | 6299 | AD05 |
| msantd4_v | 6300 | B09 |
| msantd4_v | 6301 | F09 |
| msantd4_v | 6302 | J09 |
| msantd4_v | 6303 | N09 |
| msantd4_v | 6304 | R09 |
| msantd4_v | 6305 | V09 |
| msantd4_v | 6306 | Z09 |
| msantd4_v | 6307 | AD09 |
| msantd4_v | 6308 | B13 |
| msantd4_v | 6309 | F13 |
| msantd4_v | 6310 | J13 |
| msantd4_v | 6311 | N13 |
| msantd4_v | 6312 | R13 |
| msantd4_v | 6313 | V13 |
| msantd4_v | 6314 | Z13 |
| msantd4_v | 6315 | AD13 |
| msantd4_v | 6316 | B17 |
| msantd4_v | 6317 | F17 |
| msantd4_v | 6318 | J17 |
| msantd4_v | 6319 | N17 |
| msantd4_v | 6320 | R17 |
| msantd4_v | 6321 | V17 |
| msantd4_v | 6322 | Z17 |
| msantd4_v | 6323 | AD17 |
| msantd4_v | 6324 | B21 |
| msantd4_v | 6325 | F21 |
| msantd4_v | 6326 | J21 |
| msantd4_v | 6327 | N21 |
| msantd4_v | 6328 | R21 |
| msantd4_v | 6329 | V21 |
| msantd4_v | 6330 | Z21 |
| msantd4_v | 6331 | AD21 |
| msantd4_v | 6332 | B25 |
| msantd4_v | 6333 | F25 |
| msantd4_v | 6334 | J25 |
| msantd4_v | 6335 | N25 |
| msantd4_v | 6336 | R25 |

| | | |
|---|---|---|
| msantd4_v | 6337 | V25 |
| msantd4_v | 6338 | Z25 |
| msantd4_v | 6339 | AD25 |
| msantd4_v | 6340 | B29 |
| msantd4_v | 6341 | F29 |
| msantd4_v | 6342 | J29 |
| msantd4_v | 6343 | N29 |
| msantd4_v | 6344 | R29 |
| msantd4_v | 6345 | V29 |
| msantd4_v | 6346 | Z29 |
| msantd4_v | 6347 | AD29 |
| msantd4_v | 6348 | B33 |
| msantd4_v | 6349 | F33 |
| msantd4_v | 6350 | J33 |
| msantd4_v | 6351 | N33 |
| msantd4_v | 6352 | R33 |
| msantd4_v | 6353 | V33 |
| msantd4_v | 6354 | Z33 |
| msantd4_v | 6355 | AD33 |
| msantd4_v | 6356 | B37 |
| msantd4_v | 6357 | F37 |
| msantd4_v | 6358 | J37 |
| msantd4_v | 6359 | N37 |
| msantd4_v | 6360 | R37 |
| msantd4_v | 6361 | V37 |
| msantd4_v | 6362 | Z37 |
| msantd4_v | 6363 | AD37 |
| msantd4_v | 6364 | B41 |
| msantd4_v | 6365 | F41 |
| msantd4_v | 6366 | J41 |
| msantd4_v | 6367 | N41 |
| msantd4_v | 6368 | R41 |
| msantd4_v | 6369 | V41 |
| msantd4_v | 6370 | Z41 |
| msantd4_v | 6371 | AD41 |
| msantd4_v | 6372 | B45 |
| msantd4_v | 6373 | F45 |
| msantd4_v | 6374 | J45 |
| msantd4_v | 6375 | N45 |
| msantd4_v | 6376 | R45 |
| msantd4_v | 6377 | V45 |
| msantd4_v | 6378 | Z45 |
| msantd4_v | 6379 | AD45 |
| msantd4_v | 6380 | B03 |
| msantd4_v | 6381 | F03 |
| msantd4_v | 6382 | J03 |
| msantd4_v | 6383 | N03 |
| msantd4_v | 6384 | R03 |
| msantd4_v | 6385 | V03 |
| msantd4_v | 6386 | Z03 |

| | | |
|---|---|---|
| msantd4_v | 6387 | AD03 |
| msantd4_v | 6388 | B07 |
| msantd4_v | 6389 | F07 |
| msantd4_v | 6390 | J07 |
| msantd4_v | 6391 | N07 |
| msantd4_v | 6392 | R07 |
| msantd4_v | 6393 | V07 |
| msantd4_v | 6394 | Z07 |
| msantd4_v | 6395 | AD07 |
| msantd4_v | 6396 | B11 |
| msantd4_v | 6397 | F11 |
| msantd4_v | 6398 | J11 |
| msantd4_v | 6399 | N11 |
| msantd4_v | 6400 | R11 |
| msantd4_v | 6401 | V11 |
| msantd4_v | 6402 | Z11 |
| msantd4_v | 6403 | AD11 |
| msantd4_v | 6404 | B15 |
| msantd4_v | 6405 | F15 |
| msantd4_v | 6406 | J15 |
| msantd4_v | 6407 | N15 |
| msantd4_v | 6408 | R15 |
| msantd4_v | 6409 | V15 |
| msantd4_v | 6410 | Z15 |
| msantd4_v | 6411 | AD15 |
| msantd4_v | 6412 | B19 |
| msantd4_v | 6413 | F19 |
| msantd4_v | 6414 | J19 |
| msantd4_v | 6415 | N19 |
| msantd4_v | 6416 | R19 |
| msantd4_v | 6417 | V19 |
| msantd4_v | 6418 | Z19 |
| msantd4_v | 6419 | AD19 |
| msantd4_v | 6421 | B23 |
| msantd4_v | 6422 | F23 |
| msantd4_v | 6423 | J23 |
| msantd4_v | 6424 | N23 |
| msantd4_v | 6425 | R23 |
| msantd4_v | 6426 | V23 |
| msantd4_v | 6427 | Z23 |
| msantd4_v | 6428 | AD23 |
| msantd4_v | 6429 | B27 |
| msantd4_v | 6430 | F27 |
| msantd4_v | 6431 | J27 |
| msantd4_v | 6432 | N27 |
| msantd4_v | 6433 | R27 |
| msantd4_v | 6434 | V27 |
| msantd4_v | 6435 | Z27 |
| msantd4_v | 6436 | AD27 |
| msantd4_v | 6437 | B31 |

| | | |
|---|---|---|
| msantd4_v | 6438 | F31 |
| msantd4_v | 6439 | J31 |
| msantd4_v | 6440 | N31 |
| msantd4_v | 6441 | R31 |
| msantd4_v | 6442 | V31 |
| msantd4_v | 6443 | Z31 |
| msantd4_v | 6444 | AD31 |
| msantd4_v | 6445 | B35 |
| msantd4_v | 6446 | F35 |
| msantd4_v | 6447 | J35 |
| msantd4_v | 6448 | N35 |
| msantd4_v | 6449 | R35 |
| msantd4_v | 6450 | V35 |
| msantd4_v | 6451 | Z35 |
| msantd4_v | 6452 | AD35 |
| msantd4_v | 6453 | B39 |
| msantd4_v | 6454 | F39 |
| msantd4_v | 6455 | J39 |
| msantd4_v | 6456 | N39 |
| msantd4_v | 6457 | R39 |
| msantd4_v | 6458 | V39 |
| msantd4_v | 6459 | Z39 |
| msantd4_v | 6460 | AD39 |
| msantd4_v | 6461 | B43 |
| msantd4_v | 6462 | F43 |
| msantd4_v | 6463 | J43 |
| msantd4_v | 6464 | N43 |
| msantd4_v | 6465 | R43 |
| msantd4_v | 6466 | V43 |
| msantd4_v | 6467 | Z43 |
| msantd4_v | 6468 | AD43 |
| msantd4_v | 6469 | B47 |
| msantd4_v | 6470 | F47 |
| msantd4_v | 6471 | J47 |
| msantd4_v | 6472 | N47 |
| msantd4_v | 6473 | R47 |
| msantd4_v | 6474 | V47 |
| msantd4_v | 6475 | Z47 |
| msantd4_v | 6476 | AD47 |
| msantd4_v | 6477 | D01 |
| msantd4_v | 6478 | H01 |
| msantd4_v | 6479 | L01 |
| msantd4_v | 6480 | P01 |
| msantd4_v | 6481 | T01 |
| msantd4_v | 6482 | X01 |
| msantd4_v | 6483 | AB01 |
| msantd4_v | 6484 | AF01 |
| msantd4_v | 6485 | D05 |
| msantd4_v | 6486 | H05 |
| msantd4_v | 6487 | L05 |

| | | |
|---|---|---|
| msantd4_v | 6488 | P05 |
| msantd4_v | 6489 | T05 |
| msantd4_v | 6490 | X05 |
| msantd4_v | 6491 | AB05 |
| msantd4_v | 6492 | AF05 |
| msantd4_v | 6493 | D09 |
| msantd4_v | 6494 | H09 |
| msantd4_v | 6495 | L09 |
| msantd4_v | 6496 | P09 |
| msantd4_v | 6497 | T09 |
| msantd4_v | 6498 | X09 |
| msantd4_v | 6499 | AB09 |
| msantd4_v | 6500 | AF09 |
| msantd4_v | 6501 | D13 |
| msantd4_v | 6502 | H13 |
| msantd4_v | 6503 | L13 |
| msantd4_v | 6504 | P13 |
| msantd4_v | 6505 | T13 |
| msantd4_v | 6506 | X13 |
| msantd4_v | 6507 | AB13 |
| msantd4_v | 6508 | AF13 |
| msantd4_v | 6509 | D17 |
| msantd4_v | 6510 | H17 |
| msantd4_v | 6511 | L17 |
| msantd4_v | 6512 | P17 |
| msantd4_v | 6513 | T17 |
| msantd4_v | 6514 | X17 |
| msantd4_v | 6515 | AB17 |
| msantd4_v | 6516 | AF17 |
| msantd4_v | 6517 | D21 |
| msantd4_v | 6518 | H21 |
| msantd4_v | 6519 | L21 |
| msantd4_v | 6520 | P21 |
| msantd4_v | 6521 | T21 |
| msantd4_v | 6522 | X21 |
| msantd4_v | 6523 | AB21 |
| msantd4_v | 6524 | AF21 |
| msantd4_v | 6525 | D25 |
| msantd4_v | 6526 | H25 |
| msantd4_v | 6527 | L25 |
| msantd4_v | 6528 | P25 |
| msantd4_v | 6529 | T25 |
| msantd4_v | 6530 | X25 |
| msantd4_v | 6531 | AB25 |
| msantd4_v | 6532 | AF25 |
| msantd4_v | 6533 | D29 |
| msantd4_v | 6534 | H29 |
| msantd4_v | 6535 | L29 |
| msantd4_v | 6536 | P29 |
| msantd4_v | 6537 | T29 |

| | | |
|---|---|---|
| msantd4_v | 6538 | X29 |
| msantd4_v | 6539 | AB29 |
| msantd4_v | 6540 | AF29 |
| msantd4_v | 6541 | D33 |
| msantd4_v | 6542 | H33 |
| msantd4_v | 6543 | L33 |
| msantd4_v | 6544 | P33 |
| msantd4_v | 6545 | T33 |
| msantd4_v | 6546 | X33 |
| msantd4_v | 6547 | AB33 |
| msantd4_v | 6548 | AF33 |
| msantd4_v | 6549 | D37 |
| msantd4_v | 6550 | H37 |
| msantd4_v | 6551 | L37 |
| msantd4_v | 6552 | P37 |
| msantd4_v | 6553 | T37 |
| msantd4_v | 6554 | X37 |
| msantd4_v | 6555 | AB37 |
| msantd4_v | 6556 | AF37 |
| msantd4_v | 6557 | D41 |
| msantd4_v | 6558 | H41 |
| msantd4_v | 6559 | L41 |
| msantd4_v | 6560 | P41 |
| msantd4_v | 6561 | T41 |
| msantd4_v | 6562 | X41 |
| msantd4_v | 6563 | AB41 |
| msantd4_v | 6564 | AF41 |
| msantd4_v | 6565 | D45 |
| msantd4_v | 6566 | H45 |
| msantd4_v | 6567 | L45 |
| msantd4_v | 6568 | P45 |
| msantd4_v | 6569 | T45 |
| msantd4_v | 6570 | X45 |
| msantd4_v | 6571 | AB45 |
| msantd4_v | 6572 | AF45 |
| msantd4_v | 6573 | D03 |
| msantd4_v | 6574 | H03 |
| msantd4_v | 6575 | L03 |
| msantd4_v | 6576 | P03 |
| msantd4_v | 6577 | T03 |
| msantd4_v | 6578 | X03 |
| msantd4_v | 6579 | AB03 |
| msantd4_v | 6580 | AF03 |
| msantd4_v | 6581 | D07 |
| msantd4_v | 6582 | H07 |
| msantd4_v | 6583 | L07 |
| msantd4_v | 6584 | P07 |
| msantd4_v | 6585 | T07 |
| msantd4_v | 6586 | X07 |
| msantd4_v | 6587 | AB07 |

| | | |
|---|---|---|
| msantd4_v | 6588 | AF07 |
| msantd4_v | 6589 | D11 |
| msantd4_v | 6590 | H11 |
| msantd4_v | 6591 | L11 |
| msantd4_v | 6592 | P11 |
| msantd4_v | 6593 | T11 |
| msantd4_v | 6594 | X11 |
| msantd4_v | 6595 | AB11 |
| msantd4_v | 6596 | AF11 |
| msantd4_v | 6597 | D15 |
| msantd4_v | 6598 | H15 |
| msantd4_v | 6599 | L15 |
| msantd4_v | 6600 | P15 |
| msantd4_v | 6601 | T15 |
| msantd4_v | 6602 | X15 |
| msantd4_v | 6603 | AB15 |
| msantd4_v | 6604 | AF15 |
| msantd4_v | 6605 | D19 |
| msantd4_v | 6606 | H19 |
| msantd4_v | 6607 | L19 |
| msantd4_v | 6608 | P19 |
| msantd4_v | 6609 | T19 |
| msantd4_v | 6610 | X19 |
| msantd4_v | 6611 | AB19 |
| msantd4_v | 6612 | AF19 |
| msantd4_v | 6613 | D23 |
| msantd4_v | 6614 | H23 |
| msantd4_v | 6615 | L23 |
| msantd4_v | 6616 | P23 |
| msantd4_v | 6617 | T23 |
| msantd4_v | 6618 | X23 |
| msantd4_v | 6619 | AB23 |
| msantd4_v | 6620 | AF23 |
| msantd4_v | 6621 | D27 |
| msantd4_v | 6622 | H27 |
| msantd4_v | 6623 | L27 |
| msantd4_v | 6624 | P27 |
| msantd4_v | 6625 | T27 |
| msantd4_v | 2815 | X27 |
| msantd4_v | 2816 | AB27 |
| msantd4_v | 2817 | AF27 |
| msantd4_v | 2820 | D31 |
| msantd4_v | 2821 | H31 |
| msantd4_v | 2822 | L31 |
| msantd4_v | 3245 | P31 |
| msantd4_v | 3246 | T31 |
| msantd4_v | 3247 | X31 |
| msantd4_v | 3472 | AB31 |
| msantd4_v | 3473 | AF31 |
| msantd4_v | 3474 | D35 |

| | | |
|---|---|---|
| msantd4_v | 3327 | H35 |
| msantd4_v | 3583 | L35 |
| msantd4_v | 3584 | P35 |
| msantd4_v | 3601 | T35 |
| msantd4_v | 3602 | X35 |
| msantd4_v | 3603 | AB35 |
| msantd4_v | 3775 | AF35 |
| msantd4_v | 3776 | D39 |
| msantd4_v | 3777 | H39 |
| msantd4_v | 1808 | L39 |
| msantd4_v | 1809 | P39 |
| msantd4_v | 1812 | T39 |
| msantd4_v | 1879 | X39 |
| msantd4_v | 1880 | AB39 |
| msantd4_v | 1881 | AF39 |
| msantd4_v | 2360 | D43 |
| msantd4_v | 2361 | H43 |
| msantd4_v | 2362 | L43 |
| msantd4_v | 2492 | P43 |
| msantd4_v | 2493 | T43 |
| msantd4_v | 2494 | X43 |
| msantd4_v | 2579 | AB43 |
| msantd4_v | 2580 | AF43 |
| msantd4_v | 2581 | D47 |
| msantd4_v | 2602 | H47 |
| msantd4_v | 2603 | L47 |
| msantd4_v | 2604 | P47 |
| msantd4_v | 2846 | T47 |
| msantd4_v | 2847 | X47 |
| msantd4_v | 2848 | AB47 |
| msantd4_v | NTC | AF47 |
| opa_var1 | 6284 | B02 |
| opa_var1 | 6285 | F02 |
| opa_var1 | 6286 | J02 |
| opa_var1 | 6287 | N02 |
| opa_var1 | 6288 | R02 |
| opa_var1 | 6289 | V02 |
| opa_var1 | 6290 | Z02 |
| opa_var1 | 6291 | AD02 |
| opa_var1 | 6292 | B06 |
| opa_var1 | 6293 | F06 |
| opa_var1 | 6294 | J06 |
| opa_var1 | 6295 | N06 |
| opa_var1 | 6296 | R06 |
| opa_var1 | 6297 | V06 |
| opa_var1 | 6298 | Z06 |
| opa_var1 | 6299 | AD06 |
| opa_var1 | 6300 | B10 |
| opa_var1 | 6301 | F10 |
| opa_var1 | 6302 | J10 |

| | | |
|---|---|---|
| opa_var1 | 6303 | N10 |
| opa_var1 | 6304 | R10 |
| opa_var1 | 6305 | V10 |
| opa_var1 | 6306 | Z10 |
| opa_var1 | 6307 | AD10 |
| opa_var1 | 6308 | B14 |
| opa_var1 | 6309 | F14 |
| opa_var1 | 6310 | J14 |
| opa_var1 | 6311 | N14 |
| opa_var1 | 6312 | R14 |
| opa_var1 | 6313 | V14 |
| opa_var1 | 6314 | Z14 |
| opa_var1 | 6315 | AD14 |
| opa_var1 | 6316 | B18 |
| opa_var1 | 6317 | F18 |
| opa_var1 | 6318 | J18 |
| opa_var1 | 6319 | N18 |
| opa_var1 | 6320 | R18 |
| opa_var1 | 6321 | V18 |
| opa_var1 | 6322 | Z18 |
| opa_var1 | 6323 | AD18 |
| opa_var1 | 6324 | B22 |
| opa_var1 | 6325 | F22 |
| opa_var1 | 6326 | J22 |
| opa_var1 | 6327 | N22 |
| opa_var1 | 6328 | R22 |
| opa_var1 | 6329 | V22 |
| opa_var1 | 6330 | Z22 |
| opa_var1 | 6331 | AD22 |
| opa_var1 | 6332 | B26 |
| opa_var1 | 6333 | F26 |
| opa_var1 | 6334 | J26 |
| opa_var1 | 6335 | N26 |
| opa_var1 | 6336 | R26 |
| opa_var1 | 6337 | V26 |
| opa_var1 | 6338 | Z26 |
| opa_var1 | 6339 | AD26 |
| opa_var1 | 6340 | B30 |
| opa_var1 | 6341 | F30 |
| opa_var1 | 6342 | J30 |
| opa_var1 | 6343 | N30 |
| opa_var1 | 6344 | R30 |
| opa_var1 | 6345 | V30 |
| opa_var1 | 6346 | Z30 |
| opa_var1 | 6347 | AD30 |
| opa_var1 | 6348 | B34 |
| opa_var1 | 6349 | F34 |
| opa_var1 | 6350 | J34 |
| opa_var1 | 6351 | N34 |
| opa_var1 | 6352 | R34 |

| | | |
|---|---|---|
| opa_var1 | 6353 | V34 |
| opa_var1 | 6354 | Z34 |
| opa_var1 | 6355 | AD34 |
| opa_var1 | 6356 | B38 |
| opa_var1 | 6357 | F38 |
| opa_var1 | 6358 | J38 |
| opa_var1 | 6359 | N38 |
| opa_var1 | 6360 | R38 |
| opa_var1 | 6361 | V38 |
| opa_var1 | 6362 | Z38 |
| opa_var1 | 6363 | AD38 |
| opa_var1 | 6364 | B42 |
| opa_var1 | 6365 | F42 |
| opa_var1 | 6366 | J42 |
| opa_var1 | 6367 | N42 |
| opa_var1 | 6368 | R42 |
| opa_var1 | 6369 | V42 |
| opa_var1 | 6370 | Z42 |
| opa_var1 | 6371 | AD42 |
| opa_var1 | 6372 | B46 |
| opa_var1 | 6373 | F46 |
| opa_var1 | 6374 | J46 |
| opa_var1 | 6375 | N46 |
| opa_var1 | 6376 | R46 |
| opa_var1 | 6377 | V46 |
| opa_var1 | 6378 | Z46 |
| opa_var1 | 6379 | AD46 |
| opa_var1 | 6380 | B04 |
| opa_var1 | 6381 | F04 |
| opa_var1 | 6382 | J04 |
| opa_var1 | 6383 | N04 |
| opa_var1 | 6384 | R04 |
| opa_var1 | 6385 | V04 |
| opa_var1 | 6386 | Z04 |
| opa_var1 | 6387 | AD04 |
| opa_var1 | 6388 | B08 |
| opa_var1 | 6389 | F08 |
| opa_var1 | 6390 | J08 |
| opa_var1 | 6391 | N08 |
| opa_var1 | 6392 | R08 |
| opa_var1 | 6393 | V08 |
| opa_var1 | 6394 | Z08 |
| opa_var1 | 6395 | AD08 |
| opa_var1 | 6396 | B12 |
| opa_var1 | 6397 | F12 |
| opa_var1 | 6398 | J12 |
| opa_var1 | 6399 | N12 |
| opa_var1 | 6400 | R12 |
| opa_var1 | 6401 | V12 |
| opa_var1 | 6402 | Z12 |

| | | |
|---|---|---|
| opa_var1 | 6403 | AD12 |
| opa_var1 | 6404 | B16 |
| opa_var1 | 6405 | F16 |
| opa_var1 | 6406 | J16 |
| opa_var1 | 6407 | N16 |
| opa_var1 | 6408 | R16 |
| opa_var1 | 6409 | V16 |
| opa_var1 | 6410 | Z16 |
| opa_var1 | 6411 | AD16 |
| opa_var1 | 6412 | B20 |
| opa_var1 | 6413 | F20 |
| opa_var1 | 6414 | J20 |
| opa_var1 | 6415 | N20 |
| opa_var1 | 6416 | R20 |
| opa_var1 | 6417 | V20 |
| opa_var1 | 6418 | Z20 |
| opa_var1 | 6419 | AD20 |
| opa_var1 | 6421 | B24 |
| opa_var1 | 6422 | F24 |
| opa_var1 | 6423 | J24 |
| opa_var1 | 6424 | N24 |
| opa_var1 | 6425 | R24 |
| opa_var1 | 6426 | V24 |
| opa_var1 | 6427 | Z24 |
| opa_var1 | 6428 | AD24 |
| opa_var1 | 6429 | B28 |
| opa_var1 | 6430 | F28 |
| opa_var1 | 6431 | J28 |
| opa_var1 | 6432 | N28 |
| opa_var1 | 6433 | R28 |
| opa_var1 | 6434 | V28 |
| opa_var1 | 6435 | Z28 |
| opa_var1 | 6436 | AD28 |
| opa_var1 | 6437 | B32 |
| opa_var1 | 6438 | F32 |
| opa_var1 | 6439 | J32 |
| opa_var1 | 6440 | N32 |
| opa_var1 | 6441 | R32 |
| opa_var1 | 6442 | V32 |
| opa_var1 | 6443 | Z32 |
| opa_var1 | 6444 | AD32 |
| opa_var1 | 6445 | B36 |
| opa_var1 | 6446 | F36 |
| opa_var1 | 6447 | J36 |
| opa_var1 | 6448 | N36 |
| opa_var1 | 6449 | R36 |
| opa_var1 | 6450 | V36 |
| opa_var1 | 6451 | Z36 |
| opa_var1 | 6452 | AD36 |
| opa_var1 | 6453 | B40 |

| | | |
|---|---|---|
| opa_var1 | 6454 | F40 |
| opa_var1 | 6455 | J40 |
| opa_var1 | 6456 | N40 |
| opa_var1 | 6457 | R40 |
| opa_var1 | 6458 | V40 |
| opa_var1 | 6459 | Z40 |
| opa_var1 | 6460 | AD40 |
| opa_var1 | 6461 | B44 |
| opa_var1 | 6462 | F44 |
| opa_var1 | 6463 | J44 |
| opa_var1 | 6464 | N44 |
| opa_var1 | 6465 | R44 |
| opa_var1 | 6466 | V44 |
| opa_var1 | 6467 | Z44 |
| opa_var1 | 6468 | AD44 |
| opa_var1 | 6469 | B48 |
| opa_var1 | 6470 | F48 |
| opa_var1 | 6471 | J48 |
| opa_var1 | 6472 | N48 |
| opa_var1 | 6473 | R48 |
| opa_var1 | 6474 | V48 |
| opa_var1 | 6475 | Z48 |
| opa_var1 | 6476 | AD48 |
| opa_var1 | 6477 | D02 |
| opa_var1 | 6478 | H02 |
| opa_var1 | 6479 | L02 |
| opa_var1 | 6480 | P02 |
| opa_var1 | 6481 | T02 |
| opa_var1 | 6482 | X02 |
| opa_var1 | 6483 | AB02 |
| opa_var1 | 6484 | AF02 |
| opa_var1 | 6485 | D06 |
| opa_var1 | 6486 | H06 |
| opa_var1 | 6487 | L06 |
| opa_var1 | 6488 | P06 |
| opa_var1 | 6489 | T06 |
| opa_var1 | 6490 | X06 |
| opa_var1 | 6491 | AB06 |
| opa_var1 | 6492 | AF06 |
| opa_var1 | 6493 | D10 |
| opa_var1 | 6494 | H10 |
| opa_var1 | 6495 | L10 |
| opa_var1 | 6496 | P10 |
| opa_var1 | 6497 | T10 |
| opa_var1 | 6498 | X10 |
| opa_var1 | 6499 | AB10 |
| opa_var1 | 6500 | AF10 |
| opa_var1 | 6501 | D14 |
| opa_var1 | 6502 | H14 |
| opa_var1 | 6503 | L14 |

| | | |
|---|---|---|
| opa_var1 | 6504 | P14 |
| opa_var1 | 6505 | T14 |
| opa_var1 | 6506 | X14 |
| opa_var1 | 6507 | AB14 |
| opa_var1 | 6508 | AF14 |
| opa_var1 | 6509 | D18 |
| opa_var1 | 6510 | H18 |
| opa_var1 | 6511 | L18 |
| opa_var1 | 6512 | P18 |
| opa_var1 | 6513 | T18 |
| opa_var1 | 6514 | X18 |
| opa_var1 | 6515 | AB18 |
| opa_var1 | 6516 | AF18 |
| opa_var1 | 6517 | D22 |
| opa_var1 | 6518 | H22 |
| opa_var1 | 6519 | L22 |
| opa_var1 | 6520 | P22 |
| opa_var1 | 6521 | T22 |
| opa_var1 | 6522 | X22 |
| opa_var1 | 6523 | AB22 |
| opa_var1 | 6524 | AF22 |
| opa_var1 | 6525 | D26 |
| opa_var1 | 6526 | H26 |
| opa_var1 | 6527 | L26 |
| opa_var1 | 6528 | P26 |
| opa_var1 | 6529 | T26 |
| opa_var1 | 6530 | X26 |
| opa_var1 | 6531 | AB26 |
| opa_var1 | 6532 | AF26 |
| opa_var1 | 6533 | D30 |
| opa_var1 | 6534 | H30 |
| opa_var1 | 6535 | L30 |
| opa_var1 | 6536 | P30 |
| opa_var1 | 6537 | T30 |
| opa_var1 | 6538 | X30 |
| opa_var1 | 6539 | AB30 |
| opa_var1 | 6540 | AF30 |
| opa_var1 | 6541 | D34 |
| opa_var1 | 6542 | H34 |
| opa_var1 | 6543 | L34 |
| opa_var1 | 6544 | P34 |
| opa_var1 | 6545 | T34 |
| opa_var1 | 6546 | X34 |
| opa_var1 | 6547 | AB34 |
| opa_var1 | 6548 | AF34 |
| opa_var1 | 6549 | D38 |
| opa_var1 | 6550 | H38 |
| opa_var1 | 6551 | L38 |
| opa_var1 | 6552 | P38 |
| opa_var1 | 6553 | T38 |

| | | |
|---|---|---|
| opa_var1 | 6554 | X38 |
| opa_var1 | 6555 | AB38 |
| opa_var1 | 6556 | AF38 |
| opa_var1 | 6557 | D42 |
| opa_var1 | 6558 | H42 |
| opa_var1 | 6559 | L42 |
| opa_var1 | 6560 | P42 |
| opa_var1 | 6561 | T42 |
| opa_var1 | 6562 | X42 |
| opa_var1 | 6563 | AB42 |
| opa_var1 | 6564 | AF42 |
| opa_var1 | 6565 | D46 |
| opa_var1 | 6566 | H46 |
| opa_var1 | 6567 | L46 |
| opa_var1 | 6568 | P46 |
| opa_var1 | 6569 | T46 |
| opa_var1 | 6570 | X46 |
| opa_var1 | 6571 | AB46 |
| opa_var1 | 6572 | AF46 |
| opa_var1 | 6573 | D04 |
| opa_var1 | 6574 | H04 |
| opa_var1 | 6575 | L04 |
| opa_var1 | 6576 | P04 |
| opa_var1 | 6577 | T04 |
| opa_var1 | 6578 | X04 |
| opa_var1 | 6579 | AB04 |
| opa_var1 | 6580 | AF04 |
| opa_var1 | 6581 | D08 |
| opa_var1 | 6582 | H08 |
| opa_var1 | 6583 | L08 |
| opa_var1 | 6584 | P08 |
| opa_var1 | 6585 | T08 |
| opa_var1 | 6586 | X08 |
| opa_var1 | 6587 | AB08 |
| opa_var1 | 6588 | AF08 |
| opa_var1 | 6589 | D12 |
| opa_var1 | 6590 | H12 |
| opa_var1 | 6591 | L12 |
| opa_var1 | 6592 | P12 |
| opa_var1 | 6593 | T12 |
| opa_var1 | 6594 | X12 |
| opa_var1 | 6595 | AB12 |
| opa_var1 | 6596 | AF12 |
| opa_var1 | 6597 | D16 |
| opa_var1 | 6598 | H16 |
| opa_var1 | 6599 | L16 |
| opa_var1 | 6600 | P16 |
| opa_var1 | 6601 | T16 |
| opa_var1 | 6602 | X16 |
| opa_var1 | 6603 | AB16 |

| | | |
|---|---|---|
| opa_var1 | 6604 | AF16 |
| opa_var1 | 6605 | D20 |
| opa_var1 | 6606 | H20 |
| opa_var1 | 6607 | L20 |
| opa_var1 | 6608 | P20 |
| opa_var1 | 6609 | T20 |
| opa_var1 | 6610 | X20 |
| opa_var1 | 6611 | AB20 |
| opa_var1 | 6612 | AF20 |
| opa_var1 | 6613 | D24 |
| opa_var1 | 6614 | H24 |
| opa_var1 | 6615 | L24 |
| opa_var1 | 6616 | P24 |
| opa_var1 | 6617 | T24 |
| opa_var1 | 6618 | X24 |
| opa_var1 | 6619 | AB24 |
| opa_var1 | 6620 | AF24 |
| opa_var1 | 6621 | D28 |
| opa_var1 | 6622 | H28 |
| opa_var1 | 6623 | L28 |
| opa_var1 | 6624 | P28 |
| opa_var1 | 6625 | T28 |
| opa_var1 | 2815 | X28 |
| opa_var1 | 2816 | AB28 |
| opa_var1 | 2817 | AF28 |
| opa_var1 | 2820 | D32 |
| opa_var1 | 2821 | H32 |
| opa_var1 | 2822 | L32 |
| opa_var1 | 3245 | P32 |
| opa_var1 | 3246 | T32 |
| opa_var1 | 3247 | X32 |
| opa_var1 | 3472 | AB32 |
| opa_var1 | 3473 | AF32 |
| opa_var1 | 3474 | D36 |
| opa_var1 | 3327 | H36 |
| opa_var1 | 3583 | L36 |
| opa_var1 | 3584 | P36 |
| opa_var1 | 3601 | T36 |
| opa_var1 | 3602 | X36 |
| opa_var1 | 3603 | AB36 |
| opa_var1 | 3775 | AF36 |
| opa_var1 | 3776 | D40 |
| opa_var1 | 3777 | H40 |
| opa_var1 | 1808 | L40 |
| opa_var1 | 1809 | P40 |
| opa_var1 | 1812 | T40 |
| opa_var1 | 1879 | X40 |
| opa_var1 | 1880 | AB40 |
| opa_var1 | 1881 | AF40 |
| opa_var1 | 2360 | D44 |

| | | |
|---|---|---|
| opa_var1 | 2361 | H44 |
| opa_var1 | 2362 | L44 |
| opa_var1 | 2492 | P44 |
| opa_var1 | 2493 | T44 |
| opa_var1 | 2494 | X44 |
| opa_var1 | 2579 | AB44 |
| opa_var1 | 2580 | AF44 |
| opa_var1 | 2581 | D48 |
| opa_var1 | 2602 | H48 |
| opa_var1 | 2603 | L48 |
| opa_var1 | 2604 | P48 |
| opa_var1 | 2846 | T48 |
| opa_var1 | 2847 | X48 |
| opa_var1 | 2848 | AB48 |
| opa_var1 | NTC | AF48 |
| plekhg1_va | 6284 | A01 |
| plekhg1_va | 6285 | E01 |
| plekhg1_va | 6286 | I01 |
| plekhg1_va | 6287 | M01 |
| plekhg1_va | 6288 | Q01 |
| plekhg1_va | 6289 | U01 |
| plekhg1_va | 6290 | Y01 |
| plekhg1_va | 6291 | AC01 |
| plekhg1_va | 6292 | A05 |
| plekhg1_va | 6293 | E05 |
| plekhg1_va | 6294 | I05 |
| plekhg1_va | 6295 | M05 |
| plekhg1_va | 6296 | Q05 |
| plekhg1_va | 6297 | U05 |
| plekhg1_va | 6298 | Y05 |
| plekhg1_va | 6299 | AC05 |
| plekhg1_va | 6300 | A09 |
| plekhg1_va | 6301 | E09 |
| plekhg1_va | 6302 | I09 |
| plekhg1_va | 6303 | M09 |
| plekhg1_va | 6304 | Q09 |
| plekhg1_va | 6305 | U09 |
| plekhg1_va | 6306 | Y09 |
| plekhg1_va | 6307 | AC09 |
| plekhg1_va | 6308 | A13 |
| plekhg1_va | 6309 | E13 |
| plekhg1_va | 6310 | I13 |
| plekhg1_va | 6311 | M13 |
| plekhg1_va | 6312 | Q13 |
| plekhg1_va | 6313 | U13 |
| plekhg1_va | 6314 | Y13 |
| plekhg1_va | 6315 | AC13 |
| plekhg1_va | 6316 | A17 |
| plekhg1_va | 6317 | E17 |
| plekhg1_va | 6318 | I17 |

| | | |
|---|---|---|
| plekhg1_va | 6319 | M17 |
| plekhg1_va | 6320 | Q17 |
| plekhg1_va | 6321 | U17 |
| plekhg1_va | 6322 | Y17 |
| plekhg1_va | 6323 | AC17 |
| plekhg1_va | 6324 | A21 |
| plekhg1_va | 6325 | E21 |
| plekhg1_va | 6326 | I21 |
| plekhg1_va | 6327 | M21 |
| plekhg1_va | 6328 | Q21 |
| plekhg1_va | 6329 | U21 |
| plekhg1_va | 6330 | Y21 |
| plekhg1_va | 6331 | AC21 |
| plekhg1_va | 6332 | A25 |
| plekhg1_va | 6333 | E25 |
| plekhg1_va | 6334 | I25 |
| plekhg1_va | 6335 | M25 |
| plekhg1_va | 6336 | Q25 |
| plekhg1_va | 6337 | U25 |
| plekhg1_va | 6338 | Y25 |
| plekhg1_va | 6339 | AC25 |
| plekhg1_va | 6340 | A29 |
| plekhg1_va | 6341 | E29 |
| plekhg1_va | 6342 | I29 |
| plekhg1_va | 6343 | M29 |
| plekhg1_va | 6344 | Q29 |
| plekhg1_va | 6345 | U29 |
| plekhg1_va | 6346 | Y29 |
| plekhg1_va | 6347 | AC29 |
| plekhg1_va | 6348 | A33 |
| plekhg1_va | 6349 | E33 |
| plekhg1_va | 6350 | I33 |
| plekhg1_va | 6351 | M33 |
| plekhg1_va | 6352 | Q33 |
| plekhg1_va | 6353 | U33 |
| plekhg1_va | 6354 | Y33 |
| plekhg1_va | 6355 | AC33 |
| plekhg1_va | 6356 | A37 |
| plekhg1_va | 6357 | E37 |
| plekhg1_va | 6358 | I37 |
| plekhg1_va | 6359 | M37 |
| plekhg1_va | 6360 | Q37 |
| plekhg1_va | 6361 | U37 |
| plekhg1_va | 6362 | Y37 |
| plekhg1_va | 6363 | AC37 |
| plekhg1_va | 6364 | A41 |
| plekhg1_va | 6365 | E41 |
| plekhg1_va | 6366 | I41 |
| plekhg1_va | 6367 | M41 |
| plekhg1_va | 6368 | Q41 |

| | | |
|---|---|---|
| plekhg1_va | 6369 | U41 |
| plekhg1_va | 6370 | Y41 |
| plekhg1_va | 6371 | AC41 |
| plekhg1_va | 6372 | A45 |
| plekhg1_va | 6373 | E45 |
| plekhg1_va | 6374 | I45 |
| plekhg1_va | 6375 | M45 |
| plekhg1_va | 6376 | Q45 |
| plekhg1_va | 6377 | U45 |
| plekhg1_va | 6378 | Y45 |
| plekhg1_va | 6379 | AC45 |
| plekhg1_va | 6380 | A03 |
| plekhg1_va | 6381 | E03 |
| plekhg1_va | 6382 | I03 |
| plekhg1_va | 6383 | M03 |
| plekhg1_va | 6384 | Q03 |
| plekhg1_va | 6385 | U03 |
| plekhg1_va | 6386 | Y03 |
| plekhg1_va | 6387 | AC03 |
| plekhg1_va | 6388 | A07 |
| plekhg1_va | 6389 | E07 |
| plekhg1_va | 6390 | I07 |
| plekhg1_va | 6391 | M07 |
| plekhg1_va | 6392 | Q07 |
| plekhg1_va | 6393 | U07 |
| plekhg1_va | 6394 | Y07 |
| plekhg1_va | 6395 | AC07 |
| plekhg1_va | 6396 | A11 |
| plekhg1_va | 6397 | E11 |
| plekhg1_va | 6398 | I11 |
| plekhg1_va | 6399 | M11 |
| plekhg1_va | 6400 | Q11 |
| plekhg1_va | 6401 | U11 |
| plekhg1_va | 6402 | Y11 |
| plekhg1_va | 6403 | AC11 |
| plekhg1_va | 6404 | A15 |
| plekhg1_va | 6405 | E15 |
| plekhg1_va | 6406 | I15 |
| plekhg1_va | 6407 | M15 |
| plekhg1_va | 6408 | Q15 |
| plekhg1_va | 6409 | U15 |
| plekhg1_va | 6410 | Y15 |
| plekhg1_va | 6411 | AC15 |
| plekhg1_va | 6412 | A19 |
| plekhg1_va | 6413 | E19 |
| plekhg1_va | 6414 | I19 |
| plekhg1_va | 6415 | M19 |
| plekhg1_va | 6416 | Q19 |
| plekhg1_va | 6417 | U19 |
| plekhg1_va | 6418 | Y19 |

| | | |
|---|---|---|
| plekhg1_va | 6419 | AC19 |
| plekhg1_va | 6421 | A23 |
| plekhg1_va | 6422 | E23 |
| plekhg1_va | 6423 | I23 |
| plekhg1_va | 6424 | M23 |
| plekhg1_va | 6425 | Q23 |
| plekhg1_va | 6426 | U23 |
| plekhg1_va | 6427 | Y23 |
| plekhg1_va | 6428 | AC23 |
| plekhg1_va | 6429 | A27 |
| plekhg1_va | 6430 | E27 |
| plekhg1_va | 6431 | I27 |
| plekhg1_va | 6432 | M27 |
| plekhg1_va | 6433 | Q27 |
| plekhg1_va | 6434 | U27 |
| plekhg1_va | 6435 | Y27 |
| plekhg1_va | 6436 | AC27 |
| plekhg1_va | 6437 | A31 |
| plekhg1_va | 6438 | E31 |
| plekhg1_va | 6439 | I31 |
| plekhg1_va | 6440 | M31 |
| plekhg1_va | 6441 | Q31 |
| plekhg1_va | 6442 | U31 |
| plekhg1_va | 6443 | Y31 |
| plekhg1_va | 6444 | AC31 |
| plekhg1_va | 6445 | A35 |
| plekhg1_va | 6446 | E35 |
| plekhg1_va | 6447 | I35 |
| plekhg1_va | 6448 | M35 |
| plekhg1_va | 6449 | Q35 |
| plekhg1_va | 6450 | U35 |
| plekhg1_va | 6451 | Y35 |
| plekhg1_va | 6452 | AC35 |
| plekhg1_va | 6453 | A39 |
| plekhg1_va | 6454 | E39 |
| plekhg1_va | 6455 | I39 |
| plekhg1_va | 6456 | M39 |
| plekhg1_va | 6457 | Q39 |
| plekhg1_va | 6458 | U39 |
| plekhg1_va | 6459 | Y39 |
| plekhg1_va | 6460 | AC39 |
| plekhg1_va | 6461 | A43 |
| plekhg1_va | 6462 | E43 |
| plekhg1_va | 6463 | I43 |
| plekhg1_va | 6464 | M43 |
| plekhg1_va | 6465 | Q43 |
| plekhg1_va | 6466 | U43 |
| plekhg1_va | 6467 | Y43 |
| plekhg1_va | 6468 | AC43 |
| plekhg1_va | 6469 | A47 |

| | | |
|---|---|---|
| plekhg1_va | 6470 | E47 |
| plekhg1_va | 6471 | I47 |
| plekhg1_va | 6472 | M47 |
| plekhg1_va | 6473 | Q47 |
| plekhg1_va | 6474 | U47 |
| plekhg1_va | 6475 | Y47 |
| plekhg1_va | 6476 | AC47 |
| plekhg1_va | 6477 | C01 |
| plekhg1_va | 6478 | G01 |
| plekhg1_va | 6479 | K01 |
| plekhg1_va | 6480 | O01 |
| plekhg1_va | 6481 | S01 |
| plekhg1_va | 6482 | W01 |
| plekhg1_va | 6483 | AA01 |
| plekhg1_va | 6484 | AE01 |
| plekhg1_va | 6485 | C05 |
| plekhg1_va | 6486 | G05 |
| plekhg1_va | 6487 | K05 |
| plekhg1_va | 6488 | O05 |
| plekhg1_va | 6489 | S05 |
| plekhg1_va | 6490 | W05 |
| plekhg1_va | 6491 | AA05 |
| plekhg1_va | 6492 | AE05 |
| plekhg1_va | 6493 | C09 |
| plekhg1_va | 6494 | G09 |
| plekhg1_va | 6495 | K09 |
| plekhg1_va | 6496 | O09 |
| plekhg1_va | 6497 | S09 |
| plekhg1_va | 6498 | W09 |
| plekhg1_va | 6499 | AA09 |
| plekhg1_va | 6500 | AE09 |
| plekhg1_va | 6501 | C13 |
| plekhg1_va | 6502 | G13 |
| plekhg1_va | 6503 | K13 |
| plekhg1_va | 6504 | O13 |
| plekhg1_va | 6505 | S13 |
| plekhg1_va | 6506 | W13 |
| plekhg1_va | 6507 | AA13 |
| plekhg1_va | 6508 | AE13 |
| plekhg1_va | 6509 | C17 |
| plekhg1_va | 6510 | G17 |
| plekhg1_va | 6511 | K17 |
| plekhg1_va | 6512 | O17 |
| plekhg1_va | 6513 | S17 |
| plekhg1_va | 6514 | W17 |
| plekhg1_va | 6515 | AA17 |
| plekhg1_va | 6516 | AE17 |
| plekhg1_va | 6517 | C21 |
| plekhg1_va | 6518 | G21 |
| plekhg1_va | 6519 | K21 |

| | | |
|---|---|---|
| plekhg1_va | 6520 | O21 |
| plekhg1_va | 6521 | S21 |
| plekhg1_va | 6522 | W21 |
| plekhg1_va | 6523 | AA21 |
| plekhg1_va | 6524 | AE21 |
| plekhg1_va | 6525 | C25 |
| plekhg1_va | 6526 | G25 |
| plekhg1_va | 6527 | K25 |
| plekhg1_va | 6528 | O25 |
| plekhg1_va | 6529 | S25 |
| plekhg1_va | 6530 | W25 |
| plekhg1_va | 6531 | AA25 |
| plekhg1_va | 6532 | AE25 |
| plekhg1_va | 6533 | C29 |
| plekhg1_va | 6534 | G29 |
| plekhg1_va | 6535 | K29 |
| plekhg1_va | 6536 | O29 |
| plekhg1_va | 6537 | S29 |
| plekhg1_va | 6538 | W29 |
| plekhg1_va | 6539 | AA29 |
| plekhg1_va | 6540 | AE29 |
| plekhg1_va | 6541 | C33 |
| plekhg1_va | 6542 | G33 |
| plekhg1_va | 6543 | K33 |
| plekhg1_va | 6544 | O33 |
| plekhg1_va | 6545 | S33 |
| plekhg1_va | 6546 | W33 |
| plekhg1_va | 6547 | AA33 |
| plekhg1_va | 6548 | AE33 |
| plekhg1_va | 6549 | C37 |
| plekhg1_va | 6550 | G37 |
| plekhg1_va | 6551 | K37 |
| plekhg1_va | 6552 | O37 |
| plekhg1_va | 6553 | S37 |
| plekhg1_va | 6554 | W37 |
| plekhg1_va | 6555 | AA37 |
| plekhg1_va | 6556 | AE37 |
| plekhg1_va | 6557 | C41 |
| plekhg1_va | 6558 | G41 |
| plekhg1_va | 6559 | K41 |
| plekhg1_va | 6560 | O41 |
| plekhg1_va | 6561 | S41 |
| plekhg1_va | 6562 | W41 |
| plekhg1_va | 6563 | AA41 |
| plekhg1_va | 6564 | AE41 |
| plekhg1_va | 6565 | C45 |
| plekhg1_va | 6566 | G45 |
| plekhg1_va | 6567 | K45 |
| plekhg1_va | 6568 | O45 |
| plekhg1_va | 6569 | S45 |

| | | |
|---|---|---|
| plekhg1_va | 6570 | W45 |
| plekhg1_va | 6571 | AA45 |
| plekhg1_va | 6572 | AE45 |
| plekhg1_va | 6573 | C03 |
| plekhg1_va | 6574 | G03 |
| plekhg1_va | 6575 | K03 |
| plekhg1_va | 6576 | O03 |
| plekhg1_va | 6577 | S03 |
| plekhg1_va | 6578 | W03 |
| plekhg1_va | 6579 | AA03 |
| plekhg1_va | 6580 | AE03 |
| plekhg1_va | 6581 | C07 |
| plekhg1_va | 6582 | G07 |
| plekhg1_va | 6583 | K07 |
| plekhg1_va | 6584 | O07 |
| plekhg1_va | 6585 | S07 |
| plekhg1_va | 6586 | W07 |
| plekhg1_va | 6587 | AA07 |
| plekhg1_va | 6588 | AE07 |
| plekhg1_va | 6589 | C11 |
| plekhg1_va | 6590 | G11 |
| plekhg1_va | 6591 | K11 |
| plekhg1_va | 6592 | O11 |
| plekhg1_va | 6593 | S11 |
| plekhg1_va | 6594 | W11 |
| plekhg1_va | 6595 | AA11 |
| plekhg1_va | 6596 | AE11 |
| plekhg1_va | 6597 | C15 |
| plekhg1_va | 6598 | G15 |
| plekhg1_va | 6599 | K15 |
| plekhg1_va | 6600 | O15 |
| plekhg1_va | 6601 | S15 |
| plekhg1_va | 6602 | W15 |
| plekhg1_va | 6603 | AA15 |
| plekhg1_va | 6604 | AE15 |
| plekhg1_va | 6605 | C19 |
| plekhg1_va | 6606 | G19 |
| plekhg1_va | 6607 | K19 |
| plekhg1_va | 6608 | O19 |
| plekhg1_va | 6609 | S19 |
| plekhg1_va | 6610 | W19 |
| plekhg1_va | 6611 | AA19 |
| plekhg1_va | 6612 | AE19 |
| plekhg1_va | 6613 | C23 |
| plekhg1_va | 6614 | G23 |
| plekhg1_va | 6615 | K23 |
| plekhg1_va | 6616 | O23 |
| plekhg1_va | 6617 | S23 |
| plekhg1_va | 6618 | W23 |
| plekhg1_va | 6619 | AA23 |

| | | |
|---|---|---|
| plekhg1_va | 6620 | AE23 |
| plekhg1_va | 6621 | C27 |
| plekhg1_va | 6622 | G27 |
| plekhg1_va | 6623 | K27 |
| plekhg1_va | 6624 | O27 |
| plekhg1_va | 6625 | S27 |
| plekhg1_va | 2815 | W27 |
| plekhg1_va | 2816 | AA27 |
| plekhg1_va | 2817 | AE27 |
| plekhg1_va | 2820 | C31 |
| plekhg1_va | 2821 | G31 |
| plekhg1_va | 2822 | K31 |
| plekhg1_va | 3245 | O31 |
| plekhg1_va | 3246 | S31 |
| plekhg1_va | 3247 | W31 |
| plekhg1_va | 3472 | AA31 |
| plekhg1_va | 3473 | AE31 |
| plekhg1_va | 3474 | C35 |
| plekhg1_va | 3327 | G35 |
| plekhg1_va | 3583 | K35 |
| plekhg1_va | 3584 | O35 |
| plekhg1_va | 3601 | S35 |
| plekhg1_va | 3602 | W35 |
| plekhg1_va | 3603 | AA35 |
| plekhg1_va | 3775 | AE35 |
| plekhg1_va | 3776 | C39 |
| plekhg1_va | 3777 | G39 |
| plekhg1_va | 1808 | K39 |
| plekhg1_va | 1809 | O39 |
| plekhg1_va | 1812 | S39 |
| plekhg1_va | 1879 | W39 |
| plekhg1_va | 1880 | AA39 |
| plekhg1_va | 1881 | AE39 |
| plekhg1_va | 2360 | C43 |
| plekhg1_va | 2361 | G43 |
| plekhg1_va | 2362 | K43 |
| plekhg1_va | 2492 | O43 |
| plekhg1_va | 2493 | S43 |
| plekhg1_va | 2494 | W43 |
| plekhg1_va | 2579 | AA43 |
| plekhg1_va | 2580 | AE43 |
| plekhg1_va | 2581 | C47 |
| plekhg1_va | 2602 | G47 |
| plekhg1_va | 2603 | K47 |
| plekhg1_va | 2604 | O47 |
| plekhg1_va | 2846 | S47 |
| plekhg1_va | 2847 | W47 |
| plekhg1_va | 2848 | AA47 |
| plekhg1_va | NTC | AE47 |
| slc25_var1 | 6284 | A02 |

| | | |
|---|---|---|
| slc25_var1 | 6285 | E02 |
| slc25_var1 | 6286 | I02 |
| slc25_var1 | 6287 | M02 |
| slc25_var1 | 6288 | Q02 |
| slc25_var1 | 6289 | U02 |
| slc25_var1 | 6290 | Y02 |
| slc25_var1 | 6291 | AC02 |
| slc25_var1 | 6292 | A06 |
| slc25_var1 | 6293 | E06 |
| slc25_var1 | 6294 | I06 |
| slc25_var1 | 6295 | M06 |
| slc25_var1 | 6296 | Q06 |
| slc25_var1 | 6297 | U06 |
| slc25_var1 | 6298 | Y06 |
| slc25_var1 | 6299 | AC06 |
| slc25_var1 | 6300 | A10 |
| slc25_var1 | 6301 | E10 |
| slc25_var1 | 6302 | I10 |
| slc25_var1 | 6303 | M10 |
| slc25_var1 | 6304 | Q10 |
| slc25_var1 | 6305 | U10 |
| slc25_var1 | 6306 | Y10 |
| slc25_var1 | 6307 | AC10 |
| slc25_var1 | 6308 | A14 |
| slc25_var1 | 6309 | E14 |
| slc25_var1 | 6310 | I14 |
| slc25_var1 | 6311 | M14 |
| slc25_var1 | 6312 | Q14 |
| slc25_var1 | 6313 | U14 |
| slc25_var1 | 6314 | Y14 |
| slc25_var1 | 6315 | AC14 |
| slc25_var1 | 6316 | A18 |
| slc25_var1 | 6317 | E18 |
| slc25_var1 | 6318 | I18 |
| slc25_var1 | 6319 | M18 |
| slc25_var1 | 6320 | Q18 |
| slc25_var1 | 6321 | U18 |
| slc25_var1 | 6322 | Y18 |
| slc25_var1 | 6323 | AC18 |
| slc25_var1 | 6324 | A22 |
| slc25_var1 | 6325 | E22 |
| slc25_var1 | 6326 | I22 |
| slc25_var1 | 6327 | M22 |
| slc25_var1 | 6328 | Q22 |
| slc25_var1 | 6329 | U22 |
| slc25_var1 | 6330 | Y22 |
| slc25_var1 | 6331 | AC22 |
| slc25_var1 | 6332 | A26 |
| slc25_var1 | 6333 | E26 |
| slc25_var1 | 6334 | I26 |

| | | |
|---|---|---|
| slc25_var1 | 6335 | M26 |
| slc25_var1 | 6336 | Q26 |
| slc25_var1 | 6337 | U26 |
| slc25_var1 | 6338 | Y26 |
| slc25_var1 | 6339 | AC26 |
| slc25_var1 | 6340 | A30 |
| slc25_var1 | 6341 | E30 |
| slc25_var1 | 6342 | I30 |
| slc25_var1 | 6343 | M30 |
| slc25_var1 | 6344 | Q30 |
| slc25_var1 | 6345 | U30 |
| slc25_var1 | 6346 | Y30 |
| slc25_var1 | 6347 | AC30 |
| slc25_var1 | 6348 | A34 |
| slc25_var1 | 6349 | E34 |
| slc25_var1 | 6350 | I34 |
| slc25_var1 | 6351 | M34 |
| slc25_var1 | 6352 | Q34 |
| slc25_var1 | 6353 | U34 |
| slc25_var1 | 6354 | Y34 |
| slc25_var1 | 6355 | AC34 |
| slc25_var1 | 6356 | A38 |
| slc25_var1 | 6357 | E38 |
| slc25_var1 | 6358 | I38 |
| slc25_var1 | 6359 | M38 |
| slc25_var1 | 6360 | Q38 |
| slc25_var1 | 6361 | U38 |
| slc25_var1 | 6362 | Y38 |
| slc25_var1 | 6363 | AC38 |
| slc25_var1 | 6364 | A42 |
| slc25_var1 | 6365 | E42 |
| slc25_var1 | 6366 | I42 |
| slc25_var1 | 6367 | M42 |
| slc25_var1 | 6368 | Q42 |
| slc25_var1 | 6369 | U42 |
| slc25_var1 | 6370 | Y42 |
| slc25_var1 | 6371 | AC42 |
| slc25_var1 | 6372 | A46 |
| slc25_var1 | 6373 | E46 |
| slc25_var1 | 6374 | I46 |
| slc25_var1 | 6375 | M46 |
| slc25_var1 | 6376 | Q46 |
| slc25_var1 | 6377 | U46 |
| slc25_var1 | 6378 | Y46 |
| slc25_var1 | 6379 | AC46 |
| slc25_var1 | 6380 | A04 |
| slc25_var1 | 6381 | E04 |
| slc25_var1 | 6382 | I04 |
| slc25_var1 | 6383 | M04 |
| slc25_var1 | 6384 | Q04 |

| | | |
|---|---|---|
| slc25_var1 | 6385 | U04 |
| slc25_var1 | 6386 | Y04 |
| slc25_var1 | 6387 | AC04 |
| slc25_var1 | 6388 | A08 |
| slc25_var1 | 6389 | E08 |
| slc25_var1 | 6390 | I08 |
| slc25_var1 | 6391 | M08 |
| slc25_var1 | 6392 | Q08 |
| slc25_var1 | 6393 | U08 |
| slc25_var1 | 6394 | Y08 |
| slc25_var1 | 6395 | AC08 |
| slc25_var1 | 6396 | A12 |
| slc25_var1 | 6397 | E12 |
| slc25_var1 | 6398 | I12 |
| slc25_var1 | 6399 | M12 |
| slc25_var1 | 6400 | Q12 |
| slc25_var1 | 6401 | U12 |
| slc25_var1 | 6402 | Y12 |
| slc25_var1 | 6403 | AC12 |
| slc25_var1 | 6404 | A16 |
| slc25_var1 | 6405 | E16 |
| slc25_var1 | 6406 | I16 |
| slc25_var1 | 6407 | M16 |
| slc25_var1 | 6408 | Q16 |
| slc25_var1 | 6409 | U16 |
| slc25_var1 | 6410 | Y16 |
| slc25_var1 | 6411 | AC16 |
| slc25_var1 | 6412 | A20 |
| slc25_var1 | 6413 | E20 |
| slc25_var1 | 6414 | I20 |
| slc25_var1 | 6415 | M20 |
| slc25_var1 | 6416 | Q20 |
| slc25_var1 | 6417 | U20 |
| slc25_var1 | 6418 | Y20 |
| slc25_var1 | 6419 | AC20 |
| slc25_var1 | 6421 | A24 |
| slc25_var1 | 6422 | E24 |
| slc25_var1 | 6423 | I24 |
| slc25_var1 | 6424 | M24 |
| slc25_var1 | 6425 | Q24 |
| slc25_var1 | 6426 | U24 |
| slc25_var1 | 6427 | Y24 |
| slc25_var1 | 6428 | AC24 |
| slc25_var1 | 6429 | A28 |
| slc25_var1 | 6430 | E28 |
| slc25_var1 | 6431 | I28 |
| slc25_var1 | 6432 | M28 |
| slc25_var1 | 6433 | Q28 |
| slc25_var1 | 6434 | U28 |
| slc25_var1 | 6435 | Y28 |

| | | |
|---|---|---|
| slc25_var1 | 6436 | AC28 |
| slc25_var1 | 6437 | A32 |
| slc25_var1 | 6438 | E32 |
| slc25_var1 | 6439 | I32 |
| slc25_var1 | 6440 | M32 |
| slc25_var1 | 6441 | Q32 |
| slc25_var1 | 6442 | U32 |
| slc25_var1 | 6443 | Y32 |
| slc25_var1 | 6444 | AC32 |
| slc25_var1 | 6445 | A36 |
| slc25_var1 | 6446 | E36 |
| slc25_var1 | 6447 | I36 |
| slc25_var1 | 6448 | M36 |
| slc25_var1 | 6449 | Q36 |
| slc25_var1 | 6450 | U36 |
| slc25_var1 | 6451 | Y36 |
| slc25_var1 | 6452 | AC36 |
| slc25_var1 | 6453 | A40 |
| slc25_var1 | 6454 | E40 |
| slc25_var1 | 6455 | I40 |
| slc25_var1 | 6456 | M40 |
| slc25_var1 | 6457 | Q40 |
| slc25_var1 | 6458 | U40 |
| slc25_var1 | 6459 | Y40 |
| slc25_var1 | 6460 | AC40 |
| slc25_var1 | 6461 | A44 |
| slc25_var1 | 6462 | E44 |
| slc25_var1 | 6463 | I44 |
| slc25_var1 | 6464 | M44 |
| slc25_var1 | 6465 | Q44 |
| slc25_var1 | 6466 | U44 |
| slc25_var1 | 6467 | Y44 |
| slc25_var1 | 6468 | AC44 |
| slc25_var1 | 6469 | A48 |
| slc25_var1 | 6470 | E48 |
| slc25_var1 | 6471 | I48 |
| slc25_var1 | 6472 | M48 |
| slc25_var1 | 6473 | Q48 |
| slc25_var1 | 6474 | U48 |
| slc25_var1 | 6475 | Y48 |
| slc25_var1 | 6476 | AC48 |
| slc25_var1 | 6477 | C02 |
| slc25_var1 | 6478 | G02 |
| slc25_var1 | 6479 | K02 |
| slc25_var1 | 6480 | O02 |
| slc25_var1 | 6481 | S02 |
| slc25_var1 | 6482 | W02 |
| slc25_var1 | 6483 | AA02 |
| slc25_var1 | 6484 | AE02 |
| slc25_var1 | 6485 | C06 |

| | | |
|---|---|---|
| slc25_var1 | 6486 | G06 |
| slc25_var1 | 6487 | K06 |
| slc25_var1 | 6488 | O06 |
| slc25_var1 | 6489 | S06 |
| slc25_var1 | 6490 | W06 |
| slc25_var1 | 6491 | AA06 |
| slc25_var1 | 6492 | AE06 |
| slc25_var1 | 6493 | C10 |
| slc25_var1 | 6494 | G10 |
| slc25_var1 | 6495 | K10 |
| slc25_var1 | 6496 | O10 |
| slc25_var1 | 6497 | S10 |
| slc25_var1 | 6498 | W10 |
| slc25_var1 | 6499 | AA10 |
| slc25_var1 | 6500 | AE10 |
| slc25_var1 | 6501 | C14 |
| slc25_var1 | 6502 | G14 |
| slc25_var1 | 6503 | K14 |
| slc25_var1 | 6504 | O14 |
| slc25_var1 | 6505 | S14 |
| slc25_var1 | 6506 | W14 |
| slc25_var1 | 6507 | AA14 |
| slc25_var1 | 6508 | AE14 |
| slc25_var1 | 6509 | C18 |
| slc25_var1 | 6510 | G18 |
| slc25_var1 | 6511 | K18 |
| slc25_var1 | 6512 | O18 |
| slc25_var1 | 6513 | S18 |
| slc25_var1 | 6514 | W18 |
| slc25_var1 | 6515 | AA18 |
| slc25_var1 | 6516 | AE18 |
| slc25_var1 | 6517 | C22 |
| slc25_var1 | 6518 | G22 |
| slc25_var1 | 6519 | K22 |
| slc25_var1 | 6520 | O22 |
| slc25_var1 | 6521 | S22 |
| slc25_var1 | 6522 | W22 |
| slc25_var1 | 6523 | AA22 |
| slc25_var1 | 6524 | AE22 |
| slc25_var1 | 6525 | C26 |
| slc25_var1 | 6526 | G26 |
| slc25_var1 | 6527 | K26 |
| slc25_var1 | 6528 | O26 |
| slc25_var1 | 6529 | S26 |
| slc25_var1 | 6530 | W26 |
| slc25_var1 | 6531 | AA26 |
| slc25_var1 | 6532 | AE26 |
| slc25_var1 | 6533 | C30 |
| slc25_var1 | 6534 | G30 |
| slc25_var1 | 6535 | K30 |

| | | |
|---|---|---|
| slc25_var1 | 6536 | O30 |
| slc25_var1 | 6537 | S30 |
| slc25_var1 | 6538 | W30 |
| slc25_var1 | 6539 | AA30 |
| slc25_var1 | 6540 | AE30 |
| slc25_var1 | 6541 | C34 |
| slc25_var1 | 6542 | G34 |
| slc25_var1 | 6543 | K34 |
| slc25_var1 | 6544 | O34 |
| slc25_var1 | 6545 | S34 |
| slc25_var1 | 6546 | W34 |
| slc25_var1 | 6547 | AA34 |
| slc25_var1 | 6548 | AE34 |
| slc25_var1 | 6549 | C38 |
| slc25_var1 | 6550 | G38 |
| slc25_var1 | 6551 | K38 |
| slc25_var1 | 6552 | O38 |
| slc25_var1 | 6553 | S38 |
| slc25_var1 | 6554 | W38 |
| slc25_var1 | 6555 | AA38 |
| slc25_var1 | 6556 | AE38 |
| slc25_var1 | 6557 | C42 |
| slc25_var1 | 6558 | G42 |
| slc25_var1 | 6559 | K42 |
| slc25_var1 | 6560 | O42 |
| slc25_var1 | 6561 | S42 |
| slc25_var1 | 6562 | W42 |
| slc25_var1 | 6563 | AA42 |
| slc25_var1 | 6564 | AE42 |
| slc25_var1 | 6565 | C46 |
| slc25_var1 | 6566 | G46 |
| slc25_var1 | 6567 | K46 |
| slc25_var1 | 6568 | O46 |
| slc25_var1 | 6569 | S46 |
| slc25_var1 | 6570 | W46 |
| slc25_var1 | 6571 | AA46 |
| slc25_var1 | 6572 | AE46 |
| slc25_var1 | 6573 | C04 |
| slc25_var1 | 6574 | G04 |
| slc25_var1 | 6575 | K04 |
| slc25_var1 | 6576 | O04 |
| slc25_var1 | 6577 | S04 |
| slc25_var1 | 6578 | W04 |
| slc25_var1 | 6579 | AA04 |
| slc25_var1 | 6580 | AE04 |
| slc25_var1 | 6581 | C08 |
| slc25_var1 | 6582 | G08 |
| slc25_var1 | 6583 | K08 |
| slc25_var1 | 6584 | O08 |
| slc25_var1 | 6585 | S08 |

| | | |
|---|---|---|
| slc25_var1 | 6586 | W08 |
| slc25_var1 | 6587 | AA08 |
| slc25_var1 | 6588 | AE08 |
| slc25_var1 | 6589 | C12 |
| slc25_var1 | 6590 | G12 |
| slc25_var1 | 6591 | K12 |
| slc25_var1 | 6592 | O12 |
| slc25_var1 | 6593 | S12 |
| slc25_var1 | 6594 | W12 |
| slc25_var1 | 6595 | AA12 |
| slc25_var1 | 6596 | AE12 |
| slc25_var1 | 6597 | C16 |
| slc25_var1 | 6598 | G16 |
| slc25_var1 | 6599 | K16 |
| slc25_var1 | 6600 | O16 |
| slc25_var1 | 6601 | S16 |
| slc25_var1 | 6602 | W16 |
| slc25_var1 | 6603 | AA16 |
| slc25_var1 | 6604 | AE16 |
| slc25_var1 | 6605 | C20 |
| slc25_var1 | 6606 | G20 |
| slc25_var1 | 6607 | K20 |
| slc25_var1 | 6608 | O20 |
| slc25_var1 | 6609 | S20 |
| slc25_var1 | 6610 | W20 |
| slc25_var1 | 6611 | AA20 |
| slc25_var1 | 6612 | AE20 |
| slc25_var1 | 6613 | C24 |
| slc25_var1 | 6614 | G24 |
| slc25_var1 | 6615 | K24 |
| slc25_var1 | 6616 | O24 |
| slc25_var1 | 6617 | S24 |
| slc25_var1 | 6618 | W24 |
| slc25_var1 | 6619 | AA24 |
| slc25_var1 | 6620 | AE24 |
| slc25_var1 | 6621 | C28 |
| slc25_var1 | 6622 | G28 |
| slc25_var1 | 6623 | K28 |
| slc25_var1 | 6624 | O28 |
| slc25_var1 | 6625 | S28 |
| slc25_var1 | 2815 | W28 |
| slc25_var1 | 2816 | AA28 |
| slc25_var1 | 2817 | AE28 |
| slc25_var1 | 2820 | C32 |
| slc25_var1 | 2821 | G32 |
| slc25_var1 | 2822 | K32 |
| slc25_var1 | 3245 | O32 |
| slc25_var1 | 3246 | S32 |
| slc25_var1 | 3247 | W32 |
| slc25_var1 | 3472 | AA32 |

| | | |
|---|---|---|
| slc25_var1 | 3473 | AE32 |
| slc25_var1 | 3474 | C36 |
| slc25_var1 | 3327 | G36 |
| slc25_var1 | 3583 | K36 |
| slc25_var1 | 3584 | O36 |
| slc25_var1 | 3601 | S36 |
| slc25_var1 | 3602 | W36 |
| slc25_var1 | 3603 | AA36 |
| slc25_var1 | 3775 | AE36 |
| slc25_var1 | 3776 | C40 |
| slc25_var1 | 3777 | G40 |
| slc25_var1 | 1808 | K40 |
| slc25_var1 | 1809 | O40 |
| slc25_var1 | 1812 | S40 |
| slc25_var1 | 1879 | W40 |
| slc25_var1 | 1880 | AA40 |
| slc25_var1 | 1881 | AE40 |
| slc25_var1 | 2360 | C44 |
| slc25_var1 | 2361 | G44 |
| slc25_var1 | 2362 | K44 |
| slc25_var1 | 2492 | O44 |
| slc25_var1 | 2493 | S44 |
| slc25_var1 | 2494 | W44 |
| slc25_var1 | 2579 | AA44 |
| slc25_var1 | 2580 | AE44 |
| slc25_var1 | 2581 | C48 |
| slc25_var1 | 2602 | G48 |
| slc25_var1 | 2603 | K48 |
| slc25_var1 | 2604 | O48 |
| slc25_var1 | 2846 | S48 |
| slc25_var1 | 2847 | W48 |
| slc25_var1 | 2848 | AA48 |
| slc25_var1 NTC | | AE48 |
| sre_var1 | 6284 | B01 |
| sre_var1 | 6285 | F01 |
| sre_var1 | 6286 | J01 |
| sre_var1 | 6287 | N01 |
| sre_var1 | 6288 | R01 |
| sre_var1 | 6289 | V01 |
| sre_var1 | 6290 | Z01 |
| sre_var1 | 6291 | AD01 |
| sre_var1 | 6292 | B05 |
| sre_var1 | 6293 | F05 |
| sre_var1 | 6294 | J05 |
| sre_var1 | 6295 | N05 |
| sre_var1 | 6296 | R05 |
| sre_var1 | 6297 | V05 |
| sre_var1 | 6298 | Z05 |
| sre_var1 | 6299 | AD05 |
| sre_var1 | 6300 | B09 |

| | | |
|---|---|---|
| sre_var1 | 6301 | F09 |
| sre_var1 | 6302 | J09 |
| sre_var1 | 6303 | N09 |
| sre_var1 | 6304 | R09 |
| sre_var1 | 6305 | V09 |
| sre_var1 | 6306 | Z09 |
| sre_var1 | 6307 | AD09 |
| sre_var1 | 6308 | B13 |
| sre_var1 | 6309 | F13 |
| sre_var1 | 6310 | J13 |
| sre_var1 | 6311 | N13 |
| sre_var1 | 6312 | R13 |
| sre_var1 | 6313 | V13 |
| sre_var1 | 6314 | Z13 |
| sre_var1 | 6315 | AD13 |
| sre_var1 | 6316 | B17 |
| sre_var1 | 6317 | F17 |
| sre_var1 | 6318 | J17 |
| sre_var1 | 6319 | N17 |
| sre_var1 | 6320 | R17 |
| sre_var1 | 6321 | V17 |
| sre_var1 | 6322 | Z17 |
| sre_var1 | 6323 | AD17 |
| sre_var1 | 6324 | B21 |
| sre_var1 | 6325 | F21 |
| sre_var1 | 6326 | J21 |
| sre_var1 | 6327 | N21 |
| sre_var1 | 6328 | R21 |
| sre_var1 | 6329 | V21 |
| sre_var1 | 6330 | Z21 |
| sre_var1 | 6331 | AD21 |
| sre_var1 | 6332 | B25 |
| sre_var1 | 6333 | F25 |
| sre_var1 | 6334 | J25 |
| sre_var1 | 6335 | N25 |
| sre_var1 | 6336 | R25 |
| sre_var1 | 6337 | V25 |
| sre_var1 | 6338 | Z25 |
| sre_var1 | 6339 | AD25 |
| sre_var1 | 6340 | B29 |
| sre_var1 | 6341 | F29 |
| sre_var1 | 6342 | J29 |
| sre_var1 | 6343 | N29 |
| sre_var1 | 6344 | R29 |
| sre_var1 | 6345 | V29 |
| sre_var1 | 6346 | Z29 |
| sre_var1 | 6347 | AD29 |
| sre_var1 | 6348 | B33 |
| sre_var1 | 6349 | F33 |
| sre_var1 | 6350 | J33 |

| | | |
|---|---|---|
| sre_var1 | 6351 | N33 |
| sre_var1 | 6352 | R33 |
| sre_var1 | 6353 | V33 |
| sre_var1 | 6354 | Z33 |
| sre_var1 | 6355 | AD33 |
| sre_var1 | 6356 | B37 |
| sre_var1 | 6357 | F37 |
| sre_var1 | 6358 | J37 |
| sre_var1 | 6359 | N37 |
| sre_var1 | 6360 | R37 |
| sre_var1 | 6361 | V37 |
| sre_var1 | 6362 | Z37 |
| sre_var1 | 6363 | AD37 |
| sre_var1 | 6364 | B41 |
| sre_var1 | 6365 | F41 |
| sre_var1 | 6366 | J41 |
| sre_var1 | 6367 | N41 |
| sre_var1 | 6368 | R41 |
| sre_var1 | 6369 | V41 |
| sre_var1 | 6370 | Z41 |
| sre_var1 | 6371 | AD41 |
| sre_var1 | 6372 | B45 |
| sre_var1 | 6373 | F45 |
| sre_var1 | 6374 | J45 |
| sre_var1 | 6375 | N45 |
| sre_var1 | 6376 | R45 |
| sre_var1 | 6377 | V45 |
| sre_var1 | 6378 | Z45 |
| sre_var1 | 6379 | AD45 |
| sre_var1 | 6380 | B03 |
| sre_var1 | 6381 | F03 |
| sre_var1 | 6382 | J03 |
| sre_var1 | 6383 | N03 |
| sre_var1 | 6384 | R03 |
| sre_var1 | 6385 | V03 |
| sre_var1 | 6386 | Z03 |
| sre_var1 | 6387 | AD03 |
| sre_var1 | 6388 | B07 |
| sre_var1 | 6389 | F07 |
| sre_var1 | 6390 | J07 |
| sre_var1 | 6391 | N07 |
| sre_var1 | 6392 | R07 |
| sre_var1 | 6393 | V07 |
| sre_var1 | 6394 | Z07 |
| sre_var1 | 6395 | AD07 |
| sre_var1 | 6396 | B11 |
| sre_var1 | 6397 | F11 |
| sre_var1 | 6398 | J11 |
| sre_var1 | 6399 | N11 |
| sre_var1 | 6400 | R11 |

| | | |
|---|---|---|
| sre_var1 | 6401 | V11 |
| sre_var1 | 6402 | Z11 |
| sre_var1 | 6403 | AD11 |
| sre_var1 | 6404 | B15 |
| sre_var1 | 6405 | F15 |
| sre_var1 | 6406 | J15 |
| sre_var1 | 6407 | N15 |
| sre_var1 | 6408 | R15 |
| sre_var1 | 6409 | V15 |
| sre_var1 | 6410 | Z15 |
| sre_var1 | 6411 | AD15 |
| sre_var1 | 6412 | B19 |
| sre_var1 | 6413 | F19 |
| sre_var1 | 6414 | J19 |
| sre_var1 | 6415 | N19 |
| sre_var1 | 6416 | R19 |
| sre_var1 | 6417 | V19 |
| sre_var1 | 6418 | Z19 |
| sre_var1 | 6419 | AD19 |
| sre_var1 | 6421 | B23 |
| sre_var1 | 6422 | F23 |
| sre_var1 | 6423 | J23 |
| sre_var1 | 6424 | N23 |
| sre_var1 | 6425 | R23 |
| sre_var1 | 6426 | V23 |
| sre_var1 | 6427 | Z23 |
| sre_var1 | 6428 | AD23 |
| sre_var1 | 6429 | B27 |
| sre_var1 | 6430 | F27 |
| sre_var1 | 6431 | J27 |
| sre_var1 | 6432 | N27 |
| sre_var1 | 6433 | R27 |
| sre_var1 | 6434 | V27 |
| sre_var1 | 6435 | Z27 |
| sre_var1 | 6436 | AD27 |
| sre_var1 | 6437 | B31 |
| sre_var1 | 6438 | F31 |
| sre_var1 | 6439 | J31 |
| sre_var1 | 6440 | N31 |
| sre_var1 | 6441 | R31 |
| sre_var1 | 6442 | V31 |
| sre_var1 | 6443 | Z31 |
| sre_var1 | 6444 | AD31 |
| sre_var1 | 6445 | B35 |
| sre_var1 | 6446 | F35 |
| sre_var1 | 6447 | J35 |
| sre_var1 | 6448 | N35 |
| sre_var1 | 6449 | R35 |
| sre_var1 | 6450 | V35 |
| sre_var1 | 6451 | Z35 |

| | | |
|---|---|---|
| sre_var1 | 6452 | AD35 |
| sre_var1 | 6453 | B39 |
| sre_var1 | 6454 | F39 |
| sre_var1 | 6455 | J39 |
| sre_var1 | 6456 | N39 |
| sre_var1 | 6457 | R39 |
| sre_var1 | 6458 | V39 |
| sre_var1 | 6459 | Z39 |
| sre_var1 | 6460 | AD39 |
| sre_var1 | 6461 | B43 |
| sre_var1 | 6462 | F43 |
| sre_var1 | 6463 | J43 |
| sre_var1 | 6464 | N43 |
| sre_var1 | 6465 | R43 |
| sre_var1 | 6466 | V43 |
| sre_var1 | 6467 | Z43 |
| sre_var1 | 6468 | AD43 |
| sre_var1 | 6469 | B47 |
| sre_var1 | 6470 | F47 |
| sre_var1 | 6471 | J47 |
| sre_var1 | 6472 | N47 |
| sre_var1 | 6473 | R47 |
| sre_var1 | 6474 | V47 |
| sre_var1 | 6475 | Z47 |
| sre_var1 | 6476 | AD47 |
| sre_var1 | 6477 | D01 |
| sre_var1 | 6478 | H01 |
| sre_var1 | 6479 | L01 |
| sre_var1 | 6480 | P01 |
| sre_var1 | 6481 | T01 |
| sre_var1 | 6482 | X01 |
| sre_var1 | 6483 | AB01 |
| sre_var1 | 6484 | AF01 |
| sre_var1 | 6485 | D05 |
| sre_var1 | 6486 | H05 |
| sre_var1 | 6487 | L05 |
| sre_var1 | 6488 | P05 |
| sre_var1 | 6489 | T05 |
| sre_var1 | 6490 | X05 |
| sre_var1 | 6491 | AB05 |
| sre_var1 | 6492 | AF05 |
| sre_var1 | 6493 | D09 |
| sre_var1 | 6494 | H09 |
| sre_var1 | 6495 | L09 |
| sre_var1 | 6496 | P09 |
| sre_var1 | 6497 | T09 |
| sre_var1 | 6498 | X09 |
| sre_var1 | 6499 | AB09 |
| sre_var1 | 6500 | AF09 |
| sre_var1 | 6501 | D13 |

| | | |
|---|---|---|
| sre_var1 | 6502 | H13 |
| sre_var1 | 6503 | L13 |
| sre_var1 | 6504 | P13 |
| sre_var1 | 6505 | T13 |
| sre_var1 | 6506 | X13 |
| sre_var1 | 6507 | AB13 |
| sre_var1 | 6508 | AF13 |
| sre_var1 | 6509 | D17 |
| sre_var1 | 6510 | H17 |
| sre_var1 | 6511 | L17 |
| sre_var1 | 6512 | P17 |
| sre_var1 | 6513 | T17 |
| sre_var1 | 6514 | X17 |
| sre_var1 | 6515 | AB17 |
| sre_var1 | 6516 | AF17 |
| sre_var1 | 6517 | D21 |
| sre_var1 | 6518 | H21 |
| sre_var1 | 6519 | L21 |
| sre_var1 | 6520 | P21 |
| sre_var1 | 6521 | T21 |
| sre_var1 | 6522 | X21 |
| sre_var1 | 6523 | AB21 |
| sre_var1 | 6524 | AF21 |
| sre_var1 | 6525 | D25 |
| sre_var1 | 6526 | H25 |
| sre_var1 | 6527 | L25 |
| sre_var1 | 6528 | P25 |
| sre_var1 | 6529 | T25 |
| sre_var1 | 6530 | X25 |
| sre_var1 | 6531 | AB25 |
| sre_var1 | 6532 | AF25 |
| sre_var1 | 6533 | D29 |
| sre_var1 | 6534 | H29 |
| sre_var1 | 6535 | L29 |
| sre_var1 | 6536 | P29 |
| sre_var1 | 6537 | T29 |
| sre_var1 | 6538 | X29 |
| sre_var1 | 6539 | AB29 |
| sre_var1 | 6540 | AF29 |
| sre_var1 | 6541 | D33 |
| sre_var1 | 6542 | H33 |
| sre_var1 | 6543 | L33 |
| sre_var1 | 6544 | P33 |
| sre_var1 | 6545 | T33 |
| sre_var1 | 6546 | X33 |
| sre_var1 | 6547 | AB33 |
| sre_var1 | 6548 | AF33 |
| sre_var1 | 6549 | D37 |
| sre_var1 | 6550 | H37 |
| sre_var1 | 6551 | L37 |

| | | |
|---|---|---|
| sre_var1 | 6552 | P37 |
| sre_var1 | 6553 | T37 |
| sre_var1 | 6554 | X37 |
| sre_var1 | 6555 | AB37 |
| sre_var1 | 6556 | AF37 |
| sre_var1 | 6557 | D41 |
| sre_var1 | 6558 | H41 |
| sre_var1 | 6559 | L41 |
| sre_var1 | 6560 | P41 |
| sre_var1 | 6561 | T41 |
| sre_var1 | 6562 | X41 |
| sre_var1 | 6563 | AB41 |
| sre_var1 | 6564 | AF41 |
| sre_var1 | 6565 | D45 |
| sre_var1 | 6566 | H45 |
| sre_var1 | 6567 | L45 |
| sre_var1 | 6568 | P45 |
| sre_var1 | 6569 | T45 |
| sre_var1 | 6570 | X45 |
| sre_var1 | 6571 | AB45 |
| sre_var1 | 6572 | AF45 |
| sre_var1 | 6573 | D03 |
| sre_var1 | 6574 | H03 |
| sre_var1 | 6575 | L03 |
| sre_var1 | 6576 | P03 |
| sre_var1 | 6577 | T03 |
| sre_var1 | 6578 | X03 |
| sre_var1 | 6579 | AB03 |
| sre_var1 | 6580 | AF03 |
| sre_var1 | 6581 | D07 |
| sre_var1 | 6582 | H07 |
| sre_var1 | 6583 | L07 |
| sre_var1 | 6584 | P07 |
| sre_var1 | 6585 | T07 |
| sre_var1 | 6586 | X07 |
| sre_var1 | 6587 | AB07 |
| sre_var1 | 6588 | AF07 |
| sre_var1 | 6589 | D11 |
| sre_var1 | 6590 | H11 |
| sre_var1 | 6591 | L11 |
| sre_var1 | 6592 | P11 |
| sre_var1 | 6593 | T11 |
| sre_var1 | 6594 | X11 |
| sre_var1 | 6595 | AB11 |
| sre_var1 | 6596 | AF11 |
| sre_var1 | 6597 | D15 |
| sre_var1 | 6598 | H15 |
| sre_var1 | 6599 | L15 |
| sre_var1 | 6600 | P15 |
| sre_var1 | 6601 | T15 |

| | | |
|---|---|---|
| sre_var1 | 6602 | X15 |
| sre_var1 | 6603 | AB15 |
| sre_var1 | 6604 | AF15 |
| sre_var1 | 6605 | D19 |
| sre_var1 | 6606 | H19 |
| sre_var1 | 6607 | L19 |
| sre_var1 | 6608 | P19 |
| sre_var1 | 6609 | T19 |
| sre_var1 | 6610 | X19 |
| sre_var1 | 6611 | AB19 |
| sre_var1 | 6612 | AF19 |
| sre_var1 | 6613 | D23 |
| sre_var1 | 6614 | H23 |
| sre_var1 | 6615 | L23 |
| sre_var1 | 6616 | P23 |
| sre_var1 | 6617 | T23 |
| sre_var1 | 6618 | X23 |
| sre_var1 | 6619 | AB23 |
| sre_var1 | 6620 | AF23 |
| sre_var1 | 6621 | D27 |
| sre_var1 | 6622 | H27 |
| sre_var1 | 6623 | L27 |
| sre_var1 | 6624 | P27 |
| sre_var1 | 6625 | T27 |
| sre_var1 | 2815 | X27 |
| sre_var1 | 2816 | AB27 |
| sre_var1 | 2817 | AF27 |
| sre_var1 | 2820 | D31 |
| sre_var1 | 2821 | H31 |
| sre_var1 | 2822 | L31 |
| sre_var1 | 3245 | P31 |
| sre_var1 | 3246 | T31 |
| sre_var1 | 3247 | X31 |
| sre_var1 | 3472 | AB31 |
| sre_var1 | 3473 | AF31 |
| sre_var1 | 3474 | D35 |
| sre_var1 | 3327 | H35 |
| sre_var1 | 3583 | L35 |
| sre_var1 | 3584 | P35 |
| sre_var1 | 3601 | T35 |
| sre_var1 | 3602 | X35 |
| sre_var1 | 3603 | AB35 |
| sre_var1 | 3775 | AF35 |
| sre_var1 | 3776 | D39 |
| sre_var1 | 3777 | H39 |
| sre_var1 | 1808 | L39 |
| sre_var1 | 1809 | P39 |
| sre_var1 | 1812 | T39 |
| sre_var1 | 1879 | X39 |
| sre_var1 | 1880 | AB39 |

| | | |
|---|---|---|
| sre_var1 | 1881 | AF39 |
| sre_var1 | 2360 | D43 |
| sre_var1 | 2361 | H43 |
| sre_var1 | 2362 | L43 |
| sre_var1 | 2492 | P43 |
| sre_var1 | 2493 | T43 |
| sre_var1 | 2494 | X43 |
| sre_var1 | 2579 | AB43 |
| sre_var1 | 2580 | AF43 |
| sre_var1 | 2581 | D47 |
| sre_var1 | 2602 | H47 |
| sre_var1 | 2603 | L47 |
| sre_var1 | 2604 | P47 |
| sre_var1 | 2846 | T47 |
| sre_var1 | 2847 | X47 |
| sre_var1 | 2848 | AB47 |
| sre_var1 | NTC | AF47 |
| supt6h_var | 6284 | B02 |
| supt6h_var | 6285 | F02 |
| supt6h_var | 6286 | J02 |
| supt6h_var | 6287 | N02 |
| supt6h_var | 6288 | R02 |
| supt6h_var | 6289 | V02 |
| supt6h_var | 6290 | Z02 |
| supt6h_var | 6291 | AD02 |
| supt6h_var | 6292 | B06 |
| supt6h_var | 6293 | F06 |
| supt6h_var | 6294 | J06 |
| supt6h_var | 6295 | N06 |
| supt6h_var | 6296 | R06 |
| supt6h_var | 6297 | V06 |
| supt6h_var | 6298 | Z06 |
| supt6h_var | 6299 | AD06 |
| supt6h_var | 6300 | B10 |
| supt6h_var | 6301 | F10 |
| supt6h_var | 6302 | J10 |
| supt6h_var | 6303 | N10 |
| supt6h_var | 6304 | R10 |
| supt6h_var | 6305 | V10 |
| supt6h_var | 6306 | Z10 |
| supt6h_var | 6307 | AD10 |
| supt6h_var | 6308 | B14 |
| supt6h_var | 6309 | F14 |
| supt6h_var | 6310 | J14 |
| supt6h_var | 6311 | N14 |
| supt6h_var | 6312 | R14 |
| supt6h_var | 6313 | V14 |
| supt6h_var | 6314 | Z14 |
| supt6h_var | 6315 | AD14 |
| supt6h_var | 6316 | B18 |

| | | |
|---|---|---|
| supt6h_var | 6317 | F18 |
| supt6h_var | 6318 | J18 |
| supt6h_var | 6319 | N18 |
| supt6h_var | 6320 | R18 |
| supt6h_var | 6321 | V18 |
| supt6h_var | 6322 | Z18 |
| supt6h_var | 6323 | AD18 |
| supt6h_var | 6324 | B22 |
| supt6h_var | 6325 | F22 |
| supt6h_var | 6326 | J22 |
| supt6h_var | 6327 | N22 |
| supt6h_var | 6328 | R22 |
| supt6h_var | 6329 | V22 |
| supt6h_var | 6330 | Z22 |
| supt6h_var | 6331 | AD22 |
| supt6h_var | 6332 | B26 |
| supt6h_var | 6333 | F26 |
| supt6h_var | 6334 | J26 |
| supt6h_var | 6335 | N26 |
| supt6h_var | 6336 | R26 |
| supt6h_var | 6337 | V26 |
| supt6h_var | 6338 | Z26 |
| supt6h_var | 6339 | AD26 |
| supt6h_var | 6340 | B30 |
| supt6h_var | 6341 | F30 |
| supt6h_var | 6342 | J30 |
| supt6h_var | 6343 | N30 |
| supt6h_var | 6344 | R30 |
| supt6h_var | 6345 | V30 |
| supt6h_var | 6346 | Z30 |
| supt6h_var | 6347 | AD30 |
| supt6h_var | 6348 | B34 |
| supt6h_var | 6349 | F34 |
| supt6h_var | 6350 | J34 |
| supt6h_var | 6351 | N34 |
| supt6h_var | 6352 | R34 |
| supt6h_var | 6353 | V34 |
| supt6h_var | 6354 | Z34 |
| supt6h_var | 6355 | AD34 |
| supt6h_var | 6356 | B38 |
| supt6h_var | 6357 | F38 |
| supt6h_var | 6358 | J38 |
| supt6h_var | 6359 | N38 |
| supt6h_var | 6360 | R38 |
| supt6h_var | 6361 | V38 |
| supt6h_var | 6362 | Z38 |
| supt6h_var | 6363 | AD38 |
| supt6h_var | 6364 | B42 |
| supt6h_var | 6365 | F42 |
| supt6h_var | 6366 | J42 |

| | | |
|---|---|---|
| supt6h_var | 6367 | N42 |
| supt6h_var | 6368 | R42 |
| supt6h_var | 6369 | V42 |
| supt6h_var | 6370 | Z42 |
| supt6h_var | 6371 | AD42 |
| supt6h_var | 6372 | B46 |
| supt6h_var | 6373 | F46 |
| supt6h_var | 6374 | J46 |
| supt6h_var | 6375 | N46 |
| supt6h_var | 6376 | R46 |
| supt6h_var | 6377 | V46 |
| supt6h_var | 6378 | Z46 |
| supt6h_var | 6379 | AD46 |
| supt6h_var | 6380 | B04 |
| supt6h_var | 6381 | F04 |
| supt6h_var | 6382 | J04 |
| supt6h_var | 6383 | N04 |
| supt6h_var | 6384 | R04 |
| supt6h_var | 6385 | V04 |
| supt6h_var | 6386 | Z04 |
| supt6h_var | 6387 | AD04 |
| supt6h_var | 6388 | B08 |
| supt6h_var | 6389 | F08 |
| supt6h_var | 6390 | J08 |
| supt6h_var | 6391 | N08 |
| supt6h_var | 6392 | R08 |
| supt6h_var | 6393 | V08 |
| supt6h_var | 6394 | Z08 |
| supt6h_var | 6395 | AD08 |
| supt6h_var | 6396 | B12 |
| supt6h_var | 6397 | F12 |
| supt6h_var | 6398 | J12 |
| supt6h_var | 6399 | N12 |
| supt6h_var | 6400 | R12 |
| supt6h_var | 6401 | V12 |
| supt6h_var | 6402 | Z12 |
| supt6h_var | 6403 | AD12 |
| supt6h_var | 6404 | B16 |
| supt6h_var | 6405 | F16 |
| supt6h_var | 6406 | J16 |
| supt6h_var | 6407 | N16 |
| supt6h_var | 6408 | R16 |
| supt6h_var | 6409 | V16 |
| supt6h_var | 6410 | Z16 |
| supt6h_var | 6411 | AD16 |
| supt6h_var | 6412 | B20 |
| supt6h_var | 6413 | F20 |
| supt6h_var | 6414 | J20 |
| supt6h_var | 6415 | N20 |
| supt6h_var | 6416 | R20 |

| | | |
|---|---|---|
| supt6h_var | 6417 | V20 |
| supt6h_var | 6418 | Z20 |
| supt6h_var | 6419 | AD20 |
| supt6h_var | 6421 | B24 |
| supt6h_var | 6422 | F24 |
| supt6h_var | 6423 | J24 |
| supt6h_var | 6424 | N24 |
| supt6h_var | 6425 | R24 |
| supt6h_var | 6426 | V24 |
| supt6h_var | 6427 | Z24 |
| supt6h_var | 6428 | AD24 |
| supt6h_var | 6429 | B28 |
| supt6h_var | 6430 | F28 |
| supt6h_var | 6431 | J28 |
| supt6h_var | 6432 | N28 |
| supt6h_var | 6433 | R28 |
| supt6h_var | 6434 | V28 |
| supt6h_var | 6435 | Z28 |
| supt6h_var | 6436 | AD28 |
| supt6h_var | 6437 | B32 |
| supt6h_var | 6438 | F32 |
| supt6h_var | 6439 | J32 |
| supt6h_var | 6440 | N32 |
| supt6h_var | 6441 | R32 |
| supt6h_var | 6442 | V32 |
| supt6h_var | 6443 | Z32 |
| supt6h_var | 6444 | AD32 |
| supt6h_var | 6445 | B36 |
| supt6h_var | 6446 | F36 |
| supt6h_var | 6447 | J36 |
| supt6h_var | 6448 | N36 |
| supt6h_var | 6449 | R36 |
| supt6h_var | 6450 | V36 |
| supt6h_var | 6451 | Z36 |
| supt6h_var | 6452 | AD36 |
| supt6h_var | 6453 | B40 |
| supt6h_var | 6454 | F40 |
| supt6h_var | 6455 | J40 |
| supt6h_var | 6456 | N40 |
| supt6h_var | 6457 | R40 |
| supt6h_var | 6458 | V40 |
| supt6h_var | 6459 | Z40 |
| supt6h_var | 6460 | AD40 |
| supt6h_var | 6461 | B44 |
| supt6h_var | 6462 | F44 |
| supt6h_var | 6463 | J44 |
| supt6h_var | 6464 | N44 |
| supt6h_var | 6465 | R44 |
| supt6h_var | 6466 | V44 |
| supt6h_var | 6467 | Z44 |

| | | |
|---|---|---|
| supt6h_var | 6468 | AD44 |
| supt6h_var | 6469 | B48 |
| supt6h_var | 6470 | F48 |
| supt6h_var | 6471 | J48 |
| supt6h_var | 6472 | N48 |
| supt6h_var | 6473 | R48 |
| supt6h_var | 6474 | V48 |
| supt6h_var | 6475 | Z48 |
| supt6h_var | 6476 | AD48 |
| supt6h_var | 6477 | D02 |
| supt6h_var | 6478 | H02 |
| supt6h_var | 6479 | L02 |
| supt6h_var | 6480 | P02 |
| supt6h_var | 6481 | T02 |
| supt6h_var | 6482 | X02 |
| supt6h_var | 6483 | AB02 |
| supt6h_var | 6484 | AF02 |
| supt6h_var | 6485 | D06 |
| supt6h_var | 6486 | H06 |
| supt6h_var | 6487 | L06 |
| supt6h_var | 6488 | P06 |
| supt6h_var | 6489 | T06 |
| supt6h_var | 6490 | X06 |
| supt6h_var | 6491 | AB06 |
| supt6h_var | 6492 | AF06 |
| supt6h_var | 6493 | D10 |
| supt6h_var | 6494 | H10 |
| supt6h_var | 6495 | L10 |
| supt6h_var | 6496 | P10 |
| supt6h_var | 6497 | T10 |
| supt6h_var | 6498 | X10 |
| supt6h_var | 6499 | AB10 |
| supt6h_var | 6500 | AF10 |
| supt6h_var | 6501 | D14 |
| supt6h_var | 6502 | H14 |
| supt6h_var | 6503 | L14 |
| supt6h_var | 6504 | P14 |
| supt6h_var | 6505 | T14 |
| supt6h_var | 6506 | X14 |
| supt6h_var | 6507 | AB14 |
| supt6h_var | 6508 | AF14 |
| supt6h_var | 6509 | D18 |
| supt6h_var | 6510 | H18 |
| supt6h_var | 6511 | L18 |
| supt6h_var | 6512 | P18 |
| supt6h_var | 6513 | T18 |
| supt6h_var | 6514 | X18 |
| supt6h_var | 6515 | AB18 |
| supt6h_var | 6516 | AF18 |
| supt6h_var | 6517 | D22 |

| | | |
|---|---|---|
| supt6h_var | 6518 | H22 |
| supt6h_var | 6519 | L22 |
| supt6h_var | 6520 | P22 |
| supt6h_var | 6521 | T22 |
| supt6h_var | 6522 | X22 |
| supt6h_var | 6523 | AB22 |
| supt6h_var | 6524 | AF22 |
| supt6h_var | 6525 | D26 |
| supt6h_var | 6526 | H26 |
| supt6h_var | 6527 | L26 |
| supt6h_var | 6528 | P26 |
| supt6h_var | 6529 | T26 |
| supt6h_var | 6530 | X26 |
| supt6h_var | 6531 | AB26 |
| supt6h_var | 6532 | AF26 |
| supt6h_var | 6533 | D30 |
| supt6h_var | 6534 | H30 |
| supt6h_var | 6535 | L30 |
| supt6h_var | 6536 | P30 |
| supt6h_var | 6537 | T30 |
| supt6h_var | 6538 | X30 |
| supt6h_var | 6539 | AB30 |
| supt6h_var | 6540 | AF30 |
| supt6h_var | 6541 | D34 |
| supt6h_var | 6542 | H34 |
| supt6h_var | 6543 | L34 |
| supt6h_var | 6544 | P34 |
| supt6h_var | 6545 | T34 |
| supt6h_var | 6546 | X34 |
| supt6h_var | 6547 | AB34 |
| supt6h_var | 6548 | AF34 |
| supt6h_var | 6549 | D38 |
| supt6h_var | 6550 | H38 |
| supt6h_var | 6551 | L38 |
| supt6h_var | 6552 | P38 |
| supt6h_var | 6553 | T38 |
| supt6h_var | 6554 | X38 |
| supt6h_var | 6555 | AB38 |
| supt6h_var | 6556 | AF38 |
| supt6h_var | 6557 | D42 |
| supt6h_var | 6558 | H42 |
| supt6h_var | 6559 | L42 |
| supt6h_var | 6560 | P42 |
| supt6h_var | 6561 | T42 |
| supt6h_var | 6562 | X42 |
| supt6h_var | 6563 | AB42 |
| supt6h_var | 6564 | AF42 |
| supt6h_var | 6565 | D46 |
| supt6h_var | 6566 | H46 |
| supt6h_var | 6567 | L46 |

| | | |
|---|---|---|
| supt6h_var | 6568 | P46 |
| supt6h_var | 6569 | T46 |
| supt6h_var | 6570 | X46 |
| supt6h_var | 6571 | AB46 |
| supt6h_var | 6572 | AF46 |
| supt6h_var | 6573 | D04 |
| supt6h_var | 6574 | H04 |
| supt6h_var | 6575 | L04 |
| supt6h_var | 6576 | P04 |
| supt6h_var | 6577 | T04 |
| supt6h_var | 6578 | X04 |
| supt6h_var | 6579 | AB04 |
| supt6h_var | 6580 | AF04 |
| supt6h_var | 6581 | D08 |
| supt6h_var | 6582 | H08 |
| supt6h_var | 6583 | L08 |
| supt6h_var | 6584 | P08 |
| supt6h_var | 6585 | T08 |
| supt6h_var | 6586 | X08 |
| supt6h_var | 6587 | AB08 |
| supt6h_var | 6588 | AF08 |
| supt6h_var | 6589 | D12 |
| supt6h_var | 6590 | H12 |
| supt6h_var | 6591 | L12 |
| supt6h_var | 6592 | P12 |
| supt6h_var | 6593 | T12 |
| supt6h_var | 6594 | X12 |
| supt6h_var | 6595 | AB12 |
| supt6h_var | 6596 | AF12 |
| supt6h_var | 6597 | D16 |
| supt6h_var | 6598 | H16 |
| supt6h_var | 6599 | L16 |
| supt6h_var | 6600 | P16 |
| supt6h_var | 6601 | T16 |
| supt6h_var | 6602 | X16 |
| supt6h_var | 6603 | AB16 |
| supt6h_var | 6604 | AF16 |
| supt6h_var | 6605 | D20 |
| supt6h_var | 6606 | H20 |
| supt6h_var | 6607 | L20 |
| supt6h_var | 6608 | P20 |
| supt6h_var | 6609 | T20 |
| supt6h_var | 6610 | X20 |
| supt6h_var | 6611 | AB20 |
| supt6h_var | 6612 | AF20 |
| supt6h_var | 6613 | D24 |
| supt6h_var | 6614 | H24 |
| supt6h_var | 6615 | L24 |
| supt6h_var | 6616 | P24 |
| supt6h_var | 6617 | T24 |

| | | |
|---|---|---|
| supt6h_var | 6618 | X24 |
| supt6h_var | 6619 | AB24 |
| supt6h_var | 6620 | AF24 |
| supt6h_var | 6621 | D28 |
| supt6h_var | 6622 | H28 |
| supt6h_var | 6623 | L28 |
| supt6h_var | 6624 | P28 |
| supt6h_var | 6625 | T28 |
| supt6h_var | 2815 | X28 |
| supt6h_var | 2816 | AB28 |
| supt6h_var | 2817 | AF28 |
| supt6h_var | 2820 | D32 |
| supt6h_var | 2821 | H32 |
| supt6h_var | 2822 | L32 |
| supt6h_var | 3245 | P32 |
| supt6h_var | 3246 | T32 |
| supt6h_var | 3247 | X32 |
| supt6h_var | 3472 | AB32 |
| supt6h_var | 3473 | AF32 |
| supt6h_var | 3474 | D36 |
| supt6h_var | 3327 | H36 |
| supt6h_var | 3583 | L36 |
| supt6h_var | 3584 | P36 |
| supt6h_var | 3601 | T36 |
| supt6h_var | 3602 | X36 |
| supt6h_var | 3603 | AB36 |
| supt6h_var | 3775 | AF36 |
| supt6h_var | 3776 | D40 |
| supt6h_var | 3777 | H40 |
| supt6h_var | 1808 | L40 |
| supt6h_var | 1809 | P40 |
| supt6h_var | 1812 | T40 |
| supt6h_var | 1879 | X40 |
| supt6h_var | 1880 | AB40 |
| supt6h_var | 1881 | AF40 |
| supt6h_var | 2360 | D44 |
| supt6h_var | 2361 | H44 |
| supt6h_var | 2362 | L44 |
| supt6h_var | 2492 | P44 |
| supt6h_var | 2493 | T44 |
| supt6h_var | 2494 | X44 |
| supt6h_var | 2579 | AB44 |
| supt6h_var | 2580 | AF44 |
| supt6h_var | 2581 | D48 |
| supt6h_var | 2602 | H48 |
| supt6h_var | 2603 | L48 |
| supt6h_var | 2604 | P48 |
| supt6h_var | 2846 | T48 |
| supt6h_var | 2847 | X48 |
| supt6h_var | 2848 | AB48 |

| | | |
|---|---|---|
| supt6h_var | NTC | AF48 |
| usp_var1 | 6284 | A02 |
| usp_var1 | 6285 | E02 |
| usp_var1 | 6286 | I02 |
| usp_var1 | 6287 | M02 |
| usp_var1 | 6288 | Q02 |
| usp_var1 | 6289 | U02 |
| usp_var1 | 6290 | Y02 |
| usp_var1 | 6291 | AC02 |
| usp_var1 | 6292 | A06 |
| usp_var1 | 6293 | E06 |
| usp_var1 | 6294 | I06 |
| usp_var1 | 6295 | M06 |
| usp_var1 | 6296 | Q06 |
| usp_var1 | 6297 | U06 |
| usp_var1 | 6298 | Y06 |
| usp_var1 | 6299 | AC06 |
| usp_var1 | 6300 | A10 |
| usp_var1 | 6301 | E10 |
| usp_var1 | 6302 | I10 |
| usp_var1 | 6303 | M10 |
| usp_var1 | 6304 | Q10 |
| usp_var1 | 6305 | U10 |
| usp_var1 | 6306 | Y10 |
| usp_var1 | 6307 | AC10 |
| usp_var1 | 6308 | A14 |
| usp_var1 | 6309 | E14 |
| usp_var1 | 6310 | I14 |
| usp_var1 | 6311 | M14 |
| usp_var1 | 6312 | Q14 |
| usp_var1 | 6313 | U14 |
| usp_var1 | 6314 | Y14 |
| usp_var1 | 6315 | AC14 |
| usp_var1 | 6316 | A18 |
| usp_var1 | 6317 | E18 |
| usp_var1 | 6318 | I18 |
| usp_var1 | 6319 | M18 |
| usp_var1 | 6320 | Q18 |
| usp_var1 | 6321 | U18 |
| usp_var1 | 6322 | Y18 |
| usp_var1 | 6323 | AC18 |
| usp_var1 | 6324 | A22 |
| usp_var1 | 6325 | E22 |
| usp_var1 | 6326 | I22 |
| usp_var1 | 6327 | M22 |
| usp_var1 | 6328 | Q22 |
| usp_var1 | 6329 | U22 |
| usp_var1 | 6330 | Y22 |
| usp_var1 | 6331 | AC22 |
| usp_var1 | 6332 | A26 |

| | | |
|---|---|---|
| usp_var1 | 6333 | E26 |
| usp_var1 | 6334 | I26 |
| usp_var1 | 6335 | M26 |
| usp_var1 | 6336 | Q26 |
| usp_var1 | 6337 | U26 |
| usp_var1 | 6338 | Y26 |
| usp_var1 | 6339 | AC26 |
| usp_var1 | 6340 | A30 |
| usp_var1 | 6341 | E30 |
| usp_var1 | 6342 | I30 |
| usp_var1 | 6343 | M30 |
| usp_var1 | 6344 | Q30 |
| usp_var1 | 6345 | U30 |
| usp_var1 | 6346 | Y30 |
| usp_var1 | 6347 | AC30 |
| usp_var1 | 6348 | A34 |
| usp_var1 | 6349 | E34 |
| usp_var1 | 6350 | I34 |
| usp_var1 | 6351 | M34 |
| usp_var1 | 6352 | Q34 |
| usp_var1 | 6353 | U34 |
| usp_var1 | 6354 | Y34 |
| usp_var1 | 6355 | AC34 |
| usp_var1 | 6356 | A38 |
| usp_var1 | 6357 | E38 |
| usp_var1 | 6358 | I38 |
| usp_var1 | 6359 | M38 |
| usp_var1 | 6360 | Q38 |
| usp_var1 | 6361 | U38 |
| usp_var1 | 6362 | Y38 |
| usp_var1 | 6363 | AC38 |
| usp_var1 | 6364 | A42 |
| usp_var1 | 6365 | E42 |
| usp_var1 | 6366 | I42 |
| usp_var1 | 6367 | M42 |
| usp_var1 | 6368 | Q42 |
| usp_var1 | 6369 | U42 |
| usp_var1 | 6370 | Y42 |
| usp_var1 | 6371 | AC42 |
| usp_var1 | 6372 | A46 |
| usp_var1 | 6373 | E46 |
| usp_var1 | 6374 | I46 |
| usp_var1 | 6375 | M46 |
| usp_var1 | 6376 | Q46 |
| usp_var1 | 6377 | U46 |
| usp_var1 | 6378 | Y46 |
| usp_var1 | 6379 | AC46 |
| usp_var1 | 6380 | A04 |
| usp_var1 | 6381 | E04 |
| usp_var1 | 6382 | I04 |

| | | |
|---|---|---|
| usp_var1 | 6383 | M04 |
| usp_var1 | 6384 | Q04 |
| usp_var1 | 6385 | U04 |
| usp_var1 | 6386 | Y04 |
| usp_var1 | 6387 | AC04 |
| usp_var1 | 6388 | A08 |
| usp_var1 | 6389 | E08 |
| usp_var1 | 6390 | I08 |
| usp_var1 | 6391 | M08 |
| usp_var1 | 6392 | Q08 |
| usp_var1 | 6393 | U08 |
| usp_var1 | 6394 | Y08 |
| usp_var1 | 6395 | AC08 |
| usp_var1 | 6396 | A12 |
| usp_var1 | 6397 | E12 |
| usp_var1 | 6398 | I12 |
| usp_var1 | 6399 | M12 |
| usp_var1 | 6400 | Q12 |
| usp_var1 | 6401 | U12 |
| usp_var1 | 6402 | Y12 |
| usp_var1 | 6403 | AC12 |
| usp_var1 | 6404 | A16 |
| usp_var1 | 6405 | E16 |
| usp_var1 | 6406 | I16 |
| usp_var1 | 6407 | M16 |
| usp_var1 | 6408 | Q16 |
| usp_var1 | 6409 | U16 |
| usp_var1 | 6410 | Y16 |
| usp_var1 | 6411 | AC16 |
| usp_var1 | 6412 | A20 |
| usp_var1 | 6413 | E20 |
| usp_var1 | 6414 | I20 |
| usp_var1 | 6415 | M20 |
| usp_var1 | 6416 | Q20 |
| usp_var1 | 6417 | U20 |
| usp_var1 | 6418 | Y20 |
| usp_var1 | 6419 | AC20 |
| usp_var1 | 6421 | A24 |
| usp_var1 | 6422 | E24 |
| usp_var1 | 6423 | I24 |
| usp_var1 | 6424 | M24 |
| usp_var1 | 6425 | Q24 |
| usp_var1 | 6426 | U24 |
| usp_var1 | 6427 | Y24 |
| usp_var1 | 6428 | AC24 |
| usp_var1 | 6429 | A28 |
| usp_var1 | 6430 | E28 |
| usp_var1 | 6431 | I28 |
| usp_var1 | 6432 | M28 |
| usp_var1 | 6433 | Q28 |

| | | |
|---|---|---|
| usp_var1 | 6434 | U28 |
| usp_var1 | 6435 | Y28 |
| usp_var1 | 6436 | AC28 |
| usp_var1 | 6437 | A32 |
| usp_var1 | 6438 | E32 |
| usp_var1 | 6439 | I32 |
| usp_var1 | 6440 | M32 |
| usp_var1 | 6441 | Q32 |
| usp_var1 | 6442 | U32 |
| usp_var1 | 6443 | Y32 |
| usp_var1 | 6444 | AC32 |
| usp_var1 | 6445 | A36 |
| usp_var1 | 6446 | E36 |
| usp_var1 | 6447 | I36 |
| usp_var1 | 6448 | M36 |
| usp_var1 | 6449 | Q36 |
| usp_var1 | 6450 | U36 |
| usp_var1 | 6451 | Y36 |
| usp_var1 | 6452 | AC36 |
| usp_var1 | 6453 | A40 |
| usp_var1 | 6454 | E40 |
| usp_var1 | 6455 | I40 |
| usp_var1 | 6456 | M40 |
| usp_var1 | 6457 | Q40 |
| usp_var1 | 6458 | U40 |
| usp_var1 | 6459 | Y40 |
| usp_var1 | 6460 | AC40 |
| usp_var1 | 6461 | A44 |
| usp_var1 | 6462 | E44 |
| usp_var1 | 6463 | I44 |
| usp_var1 | 6464 | M44 |
| usp_var1 | 6465 | Q44 |
| usp_var1 | 6466 | U44 |
| usp_var1 | 6467 | Y44 |
| usp_var1 | 6468 | AC44 |
| usp_var1 | 6469 | A48 |
| usp_var1 | 6470 | E48 |
| usp_var1 | 6471 | I48 |
| usp_var1 | 6472 | M48 |
| usp_var1 | 6473 | Q48 |
| usp_var1 | 6474 | U48 |
| usp_var1 | 6475 | Y48 |
| usp_var1 | 6476 | AC48 |
| usp_var1 | 6477 | C02 |
| usp_var1 | 6478 | G02 |
| usp_var1 | 6479 | K02 |
| usp_var1 | 6480 | O02 |
| usp_var1 | 6481 | S02 |
| usp_var1 | 6482 | W02 |
| usp_var1 | 6483 | AA02 |

| | | |
|---|---|---|
| usp_var1 | 6484 | AE02 |
| usp_var1 | 6485 | C06 |
| usp_var1 | 6486 | G06 |
| usp_var1 | 6487 | K06 |
| usp_var1 | 6488 | O06 |
| usp_var1 | 6489 | S06 |
| usp_var1 | 6490 | W06 |
| usp_var1 | 6491 | AA06 |
| usp_var1 | 6492 | AE06 |
| usp_var1 | 6493 | C10 |
| usp_var1 | 6494 | G10 |
| usp_var1 | 6495 | K10 |
| usp_var1 | 6496 | O10 |
| usp_var1 | 6497 | S10 |
| usp_var1 | 6498 | W10 |
| usp_var1 | 6499 | AA10 |
| usp_var1 | 6500 | AE10 |
| usp_var1 | 6501 | C14 |
| usp_var1 | 6502 | G14 |
| usp_var1 | 6503 | K14 |
| usp_var1 | 6504 | O14 |
| usp_var1 | 6505 | S14 |
| usp_var1 | 6506 | W14 |
| usp_var1 | 6507 | AA14 |
| usp_var1 | 6508 | AE14 |
| usp_var1 | 6509 | C18 |
| usp_var1 | 6510 | G18 |
| usp_var1 | 6511 | K18 |
| usp_var1 | 6512 | O18 |
| usp_var1 | 6513 | S18 |
| usp_var1 | 6514 | W18 |
| usp_var1 | 6515 | AA18 |
| usp_var1 | 6516 | AE18 |
| usp_var1 | 6517 | C22 |
| usp_var1 | 6518 | G22 |
| usp_var1 | 6519 | K22 |
| usp_var1 | 6520 | O22 |
| usp_var1 | 6521 | S22 |
| usp_var1 | 6522 | W22 |
| usp_var1 | 6523 | AA22 |
| usp_var1 | 6524 | AE22 |
| usp_var1 | 6525 | C26 |
| usp_var1 | 6526 | G26 |
| usp_var1 | 6527 | K26 |
| usp_var1 | 6528 | O26 |
| usp_var1 | 6529 | S26 |
| usp_var1 | 6530 | W26 |
| usp_var1 | 6531 | AA26 |
| usp_var1 | 6532 | AE26 |
| usp_var1 | 6533 | C30 |

| | | |
|---|---|---|
| usp_var1 | 6534 | G30 |
| usp_var1 | 6535 | K30 |
| usp_var1 | 6536 | O30 |
| usp_var1 | 6537 | S30 |
| usp_var1 | 6538 | W30 |
| usp_var1 | 6539 | AA30 |
| usp_var1 | 6540 | AE30 |
| usp_var1 | 6541 | C34 |
| usp_var1 | 6542 | G34 |
| usp_var1 | 6543 | K34 |
| usp_var1 | 6544 | O34 |
| usp_var1 | 6545 | S34 |
| usp_var1 | 6546 | W34 |
| usp_var1 | 6547 | AA34 |
| usp_var1 | 6548 | AE34 |
| usp_var1 | 6549 | C38 |
| usp_var1 | 6550 | G38 |
| usp_var1 | 6551 | K38 |
| usp_var1 | 6552 | O38 |
| usp_var1 | 6553 | S38 |
| usp_var1 | 6554 | W38 |
| usp_var1 | 6555 | AA38 |
| usp_var1 | 6556 | AE38 |
| usp_var1 | 6557 | C42 |
| usp_var1 | 6558 | G42 |
| usp_var1 | 6559 | K42 |
| usp_var1 | 6560 | O42 |
| usp_var1 | 6561 | S42 |
| usp_var1 | 6562 | W42 |
| usp_var1 | 6563 | AA42 |
| usp_var1 | 6564 | AE42 |
| usp_var1 | 6565 | C46 |
| usp_var1 | 6566 | G46 |
| usp_var1 | 6567 | K46 |
| usp_var1 | 6568 | O46 |
| usp_var1 | 6569 | S46 |
| usp_var1 | 6570 | W46 |
| usp_var1 | 6571 | AA46 |
| usp_var1 | 6572 | AE46 |
| usp_var1 | 6573 | C04 |
| usp_var1 | 6574 | G04 |
| usp_var1 | 6575 | K04 |
| usp_var1 | 6576 | O04 |
| usp_var1 | 6577 | S04 |
| usp_var1 | 6578 | W04 |
| usp_var1 | 6579 | AA04 |
| usp_var1 | 6580 | AE04 |
| usp_var1 | 6581 | C08 |
| usp_var1 | 6582 | G08 |
| usp_var1 | 6583 | K08 |

| | | |
|---|---|---|
| usp_var1 | 6584 | O08 |
| usp_var1 | 6585 | S08 |
| usp_var1 | 6586 | W08 |
| usp_var1 | 6587 | AA08 |
| usp_var1 | 6588 | AE08 |
| usp_var1 | 6589 | C12 |
| usp_var1 | 6590 | G12 |
| usp_var1 | 6591 | K12 |
| usp_var1 | 6592 | O12 |
| usp_var1 | 6593 | S12 |
| usp_var1 | 6594 | W12 |
| usp_var1 | 6595 | AA12 |
| usp_var1 | 6596 | AE12 |
| usp_var1 | 6597 | C16 |
| usp_var1 | 6598 | G16 |
| usp_var1 | 6599 | K16 |
| usp_var1 | 6600 | O16 |
| usp_var1 | 6601 | S16 |
| usp_var1 | 6602 | W16 |
| usp_var1 | 6603 | AA16 |
| usp_var1 | 6604 | AE16 |
| usp_var1 | 6605 | C20 |
| usp_var1 | 6606 | G20 |
| usp_var1 | 6607 | K20 |
| usp_var1 | 6608 | O20 |
| usp_var1 | 6609 | S20 |
| usp_var1 | 6610 | W20 |
| usp_var1 | 6611 | AA20 |
| usp_var1 | 6612 | AE20 |
| usp_var1 | 6613 | C24 |
| usp_var1 | 6614 | G24 |
| usp_var1 | 6615 | K24 |
| usp_var1 | 6616 | O24 |
| usp_var1 | 6617 | S24 |
| usp_var1 | 6618 | W24 |
| usp_var1 | 6619 | AA24 |
| usp_var1 | 6620 | AE24 |
| usp_var1 | 6621 | C28 |
| usp_var1 | 6622 | G28 |
| usp_var1 | 6623 | K28 |
| usp_var1 | 6624 | O28 |
| usp_var1 | 6625 | S28 |
| usp_var1 | 2815 | W28 |
| usp_var1 | 2816 | AA28 |
| usp_var1 | 2817 | AE28 |
| usp_var1 | 2820 | C32 |
| usp_var1 | 2821 | G32 |
| usp_var1 | 2822 | K32 |
| usp_var1 | 3245 | O32 |
| usp_var1 | 3246 | S32 |

| | | |
|---|---|---|
| usp_var1 | 3247 | W32 |
| usp_var1 | 3472 | AA32 |
| usp_var1 | 3473 | AE32 |
| usp_var1 | 3474 | C36 |
| usp_var1 | 3327 | G36 |
| usp_var1 | 3583 | K36 |
| usp_var1 | 3584 | O36 |
| usp_var1 | 3601 | S36 |
| usp_var1 | 3602 | W36 |
| usp_var1 | 3603 | AA36 |
| usp_var1 | 3775 | AE36 |
| usp_var1 | 3776 | C40 |
| usp_var1 | 3777 | G40 |
| usp_var1 | 1808 | K40 |
| usp_var1 | 1809 | O40 |
| usp_var1 | 1812 | S40 |
| usp_var1 | 1879 | W40 |
| usp_var1 | 1880 | AA40 |
| usp_var1 | 1881 | AE40 |
| usp_var1 | 2360 | C44 |
| usp_var1 | 2361 | G44 |
| usp_var1 | 2362 | K44 |
| usp_var1 | 2492 | O44 |
| usp_var1 | 2493 | S44 |
| usp_var1 | 2494 | W44 |
| usp_var1 | 2579 | AA44 |
| usp_var1 | 2580 | AE44 |
| usp_var1 | 2581 | C48 |
| usp_var1 | 2602 | G48 |
| usp_var1 | 2603 | K48 |
| usp_var1 | 2604 | O48 |
| usp_var1 | 2846 | S48 |
| usp_var1 | 2847 | W48 |
| usp_var1 | 2848 | AA48 |
| usp_var1 | NTC | AE48 |
| wiz_var1 | 6284 | B01 |
| wiz_var1 | 6285 | F01 |
| wiz_var1 | 6286 | J01 |
| wiz_var1 | 6287 | N01 |
| wiz_var1 | 6288 | R01 |
| wiz_var1 | 6289 | V01 |
| wiz_var1 | 6290 | Z01 |
| wiz_var1 | 6291 | AD01 |
| wiz_var1 | 6292 | B05 |
| wiz_var1 | 6293 | F05 |
| wiz_var1 | 6294 | J05 |
| wiz_var1 | 6295 | N05 |
| wiz_var1 | 6296 | R05 |
| wiz_var1 | 6297 | V05 |
| wiz_var1 | 6298 | Z05 |

| | | |
|---|---|---|
| wiz_var1 | 6299 | AD05 |
| wiz_var1 | 6300 | B09 |
| wiz_var1 | 6301 | F09 |
| wiz_var1 | 6302 | J09 |
| wiz_var1 | 6303 | N09 |
| wiz_var1 | 6304 | R09 |
| wiz_var1 | 6305 | V09 |
| wiz_var1 | 6306 | Z09 |
| wiz_var1 | 6307 | AD09 |
| wiz_var1 | 6308 | B13 |
| wiz_var1 | 6309 | F13 |
| wiz_var1 | 6310 | J13 |
| wiz_var1 | 6311 | N13 |
| wiz_var1 | 6312 | R13 |
| wiz_var1 | 6313 | V13 |
| wiz_var1 | 6314 | Z13 |
| wiz_var1 | 6315 | AD13 |
| wiz_var1 | 6316 | B17 |
| wiz_var1 | 6317 | F17 |
| wiz_var1 | 6318 | J17 |
| wiz_var1 | 6319 | N17 |
| wiz_var1 | 6320 | R17 |
| wiz_var1 | 6321 | V17 |
| wiz_var1 | 6322 | Z17 |
| wiz_var1 | 6323 | AD17 |
| wiz_var1 | 6324 | B21 |
| wiz_var1 | 6325 | F21 |
| wiz_var1 | 6326 | J21 |
| wiz_var1 | 6327 | N21 |
| wiz_var1 | 6328 | R21 |
| wiz_var1 | 6329 | V21 |
| wiz_var1 | 6330 | Z21 |
| wiz_var1 | 6331 | AD21 |
| wiz_var1 | 6332 | B25 |
| wiz_var1 | 6333 | F25 |
| wiz_var1 | 6334 | J25 |
| wiz_var1 | 6335 | N25 |
| wiz_var1 | 6336 | R25 |
| wiz_var1 | 6337 | V25 |
| wiz_var1 | 6338 | Z25 |
| wiz_var1 | 6339 | AD25 |
| wiz_var1 | 6340 | B29 |
| wiz_var1 | 6341 | F29 |
| wiz_var1 | 6342 | J29 |
| wiz_var1 | 6343 | N29 |
| wiz_var1 | 6344 | R29 |
| wiz_var1 | 6345 | V29 |
| wiz_var1 | 6346 | Z29 |
| wiz_var1 | 6347 | AD29 |
| wiz_var1 | 6348 | B33 |

| | | |
|---|---|---|
| wiz_var1 | 6349 | F33 |
| wiz_var1 | 6350 | J33 |
| wiz_var1 | 6351 | N33 |
| wiz_var1 | 6352 | R33 |
| wiz_var1 | 6353 | V33 |
| wiz_var1 | 6354 | Z33 |
| wiz_var1 | 6355 | AD33 |
| wiz_var1 | 6356 | B37 |
| wiz_var1 | 6357 | F37 |
| wiz_var1 | 6358 | J37 |
| wiz_var1 | 6359 | N37 |
| wiz_var1 | 6360 | R37 |
| wiz_var1 | 6361 | V37 |
| wiz_var1 | 6362 | Z37 |
| wiz_var1 | 6363 | AD37 |
| wiz_var1 | 6364 | B41 |
| wiz_var1 | 6365 | F41 |
| wiz_var1 | 6366 | J41 |
| wiz_var1 | 6367 | N41 |
| wiz_var1 | 6368 | R41 |
| wiz_var1 | 6369 | V41 |
| wiz_var1 | 6370 | Z41 |
| wiz_var1 | 6371 | AD41 |
| wiz_var1 | 6372 | B45 |
| wiz_var1 | 6373 | F45 |
| wiz_var1 | 6374 | J45 |
| wiz_var1 | 6375 | N45 |
| wiz_var1 | 6376 | R45 |
| wiz_var1 | 6377 | V45 |
| wiz_var1 | 6378 | Z45 |
| wiz_var1 | 6379 | AD45 |
| wiz_var1 | 6380 | B03 |
| wiz_var1 | 6381 | F03 |
| wiz_var1 | 6382 | J03 |
| wiz_var1 | 6383 | N03 |
| wiz_var1 | 6384 | R03 |
| wiz_var1 | 6385 | V03 |
| wiz_var1 | 6386 | Z03 |
| wiz_var1 | 6387 | AD03 |
| wiz_var1 | 6388 | B07 |
| wiz_var1 | 6389 | F07 |
| wiz_var1 | 6390 | J07 |
| wiz_var1 | 6391 | N07 |
| wiz_var1 | 6392 | R07 |
| wiz_var1 | 6393 | V07 |
| wiz_var1 | 6394 | Z07 |
| wiz_var1 | 6395 | AD07 |
| wiz_var1 | 6396 | B11 |
| wiz_var1 | 6397 | F11 |
| wiz_var1 | 6398 | J11 |

| | | |
|---|---|---|
| wiz_var1 | 6399 | N11 |
| wiz_var1 | 6400 | R11 |
| wiz_var1 | 6401 | V11 |
| wiz_var1 | 6402 | Z11 |
| wiz_var1 | 6403 | AD11 |
| wiz_var1 | 6404 | B15 |
| wiz_var1 | 6405 | F15 |
| wiz_var1 | 6406 | J15 |
| wiz_var1 | 6407 | N15 |
| wiz_var1 | 6408 | R15 |
| wiz_var1 | 6409 | V15 |
| wiz_var1 | 6410 | Z15 |
| wiz_var1 | 6411 | AD15 |
| wiz_var1 | 6412 | B19 |
| wiz_var1 | 6413 | F19 |
| wiz_var1 | 6414 | J19 |
| wiz_var1 | 6415 | N19 |
| wiz_var1 | 6416 | R19 |
| wiz_var1 | 6417 | V19 |
| wiz_var1 | 6418 | Z19 |
| wiz_var1 | 6419 | AD19 |
| wiz_var1 | 6421 | B23 |
| wiz_var1 | 6422 | F23 |
| wiz_var1 | 6423 | J23 |
| wiz_var1 | 6424 | N23 |
| wiz_var1 | 6425 | R23 |
| wiz_var1 | 6426 | V23 |
| wiz_var1 | 6427 | Z23 |
| wiz_var1 | 6428 | AD23 |
| wiz_var1 | 6429 | B27 |
| wiz_var1 | 6430 | F27 |
| wiz_var1 | 6431 | J27 |
| wiz_var1 | 6432 | N27 |
| wiz_var1 | 6433 | R27 |
| wiz_var1 | 6434 | V27 |
| wiz_var1 | 6435 | Z27 |
| wiz_var1 | 6436 | AD27 |
| wiz_var1 | 6437 | B31 |
| wiz_var1 | 6438 | F31 |
| wiz_var1 | 6439 | J31 |
| wiz_var1 | 6440 | N31 |
| wiz_var1 | 6441 | R31 |
| wiz_var1 | 6442 | V31 |
| wiz_var1 | 6443 | Z31 |
| wiz_var1 | 6444 | AD31 |
| wiz_var1 | 6445 | B35 |
| wiz_var1 | 6446 | F35 |
| wiz_var1 | 6447 | J35 |
| wiz_var1 | 6448 | N35 |
| wiz_var1 | 6449 | R35 |

| | | |
|---|---|---|
| wiz_var1 | 6450 | V35 |
| wiz_var1 | 6451 | Z35 |
| wiz_var1 | 6452 | AD35 |
| wiz_var1 | 6453 | B39 |
| wiz_var1 | 6454 | F39 |
| wiz_var1 | 6455 | J39 |
| wiz_var1 | 6456 | N39 |
| wiz_var1 | 6457 | R39 |
| wiz_var1 | 6458 | V39 |
| wiz_var1 | 6459 | Z39 |
| wiz_var1 | 6460 | AD39 |
| wiz_var1 | 6461 | B43 |
| wiz_var1 | 6462 | F43 |
| wiz_var1 | 6463 | J43 |
| wiz_var1 | 6464 | N43 |
| wiz_var1 | 6465 | R43 |
| wiz_var1 | 6466 | V43 |
| wiz_var1 | 6467 | Z43 |
| wiz_var1 | 6468 | AD43 |
| wiz_var1 | 6469 | B47 |
| wiz_var1 | 6470 | F47 |
| wiz_var1 | 6471 | J47 |
| wiz_var1 | 6472 | N47 |
| wiz_var1 | 6473 | R47 |
| wiz_var1 | 6474 | V47 |
| wiz_var1 | 6475 | Z47 |
| wiz_var1 | 6476 | AD47 |
| wiz_var1 | 6477 | D01 |
| wiz_var1 | 6478 | H01 |
| wiz_var1 | 6479 | L01 |
| wiz_var1 | 6480 | P01 |
| wiz_var1 | 6481 | T01 |
| wiz_var1 | 6482 | X01 |
| wiz_var1 | 6483 | AB01 |
| wiz_var1 | 6484 | AF01 |
| wiz_var1 | 6485 | D05 |
| wiz_var1 | 6486 | H05 |
| wiz_var1 | 6487 | L05 |
| wiz_var1 | 6488 | P05 |
| wiz_var1 | 6489 | T05 |
| wiz_var1 | 6490 | X05 |
| wiz_var1 | 6491 | AB05 |
| wiz_var1 | 6492 | AF05 |
| wiz_var1 | 6493 | D09 |
| wiz_var1 | 6494 | H09 |
| wiz_var1 | 6495 | L09 |
| wiz_var1 | 6496 | P09 |
| wiz_var1 | 6497 | T09 |
| wiz_var1 | 6498 | X09 |
| wiz_var1 | 6499 | AB09 |

| | | |
|---|---|---|
| wiz_var1 | 6500 | AF09 |
| wiz_var1 | 6501 | D13 |
| wiz_var1 | 6502 | H13 |
| wiz_var1 | 6503 | L13 |
| wiz_var1 | 6504 | P13 |
| wiz_var1 | 6505 | T13 |
| wiz_var1 | 6506 | X13 |
| wiz_var1 | 6507 | AB13 |
| wiz_var1 | 6508 | AF13 |
| wiz_var1 | 6509 | D17 |
| wiz_var1 | 6510 | H17 |
| wiz_var1 | 6511 | L17 |
| wiz_var1 | 6512 | P17 |
| wiz_var1 | 6513 | T17 |
| wiz_var1 | 6514 | X17 |
| wiz_var1 | 6515 | AB17 |
| wiz_var1 | 6516 | AF17 |
| wiz_var1 | 6517 | D21 |
| wiz_var1 | 6518 | H21 |
| wiz_var1 | 6519 | L21 |
| wiz_var1 | 6520 | P21 |
| wiz_var1 | 6521 | T21 |
| wiz_var1 | 6522 | X21 |
| wiz_var1 | 6523 | AB21 |
| wiz_var1 | 6524 | AF21 |
| wiz_var1 | 6525 | D25 |
| wiz_var1 | 6526 | H25 |
| wiz_var1 | 6527 | L25 |
| wiz_var1 | 6528 | P25 |
| wiz_var1 | 6529 | T25 |
| wiz_var1 | 6530 | X25 |
| wiz_var1 | 6531 | AB25 |
| wiz_var1 | 6532 | AF25 |
| wiz_var1 | 6533 | D29 |
| wiz_var1 | 6534 | H29 |
| wiz_var1 | 6535 | L29 |
| wiz_var1 | 6536 | P29 |
| wiz_var1 | 6537 | T29 |
| wiz_var1 | 6538 | X29 |
| wiz_var1 | 6539 | AB29 |
| wiz_var1 | 6540 | AF29 |
| wiz_var1 | 6541 | D33 |
| wiz_var1 | 6542 | H33 |
| wiz_var1 | 6543 | L33 |
| wiz_var1 | 6544 | P33 |
| wiz_var1 | 6545 | T33 |
| wiz_var1 | 6546 | X33 |
| wiz_var1 | 6547 | AB33 |
| wiz_var1 | 6548 | AF33 |
| wiz_var1 | 6549 | D37 |

| | | |
|---|---|---|
| wiz_var1 | 6550 | H37 |
| wiz_var1 | 6551 | L37 |
| wiz_var1 | 6552 | P37 |
| wiz_var1 | 6553 | T37 |
| wiz_var1 | 6554 | X37 |
| wiz_var1 | 6555 | AB37 |
| wiz_var1 | 6556 | AF37 |
| wiz_var1 | 6557 | D41 |
| wiz_var1 | 6558 | H41 |
| wiz_var1 | 6559 | L41 |
| wiz_var1 | 6560 | P41 |
| wiz_var1 | 6561 | T41 |
| wiz_var1 | 6562 | X41 |
| wiz_var1 | 6563 | AB41 |
| wiz_var1 | 6564 | AF41 |
| wiz_var1 | 6565 | D45 |
| wiz_var1 | 6566 | H45 |
| wiz_var1 | 6567 | L45 |
| wiz_var1 | 6568 | P45 |
| wiz_var1 | 6569 | T45 |
| wiz_var1 | 6570 | X45 |
| wiz_var1 | 6571 | AB45 |
| wiz_var1 | 6572 | AF45 |
| wiz_var1 | 6573 | D03 |
| wiz_var1 | 6574 | H03 |
| wiz_var1 | 6575 | L03 |
| wiz_var1 | 6576 | P03 |
| wiz_var1 | 6577 | T03 |
| wiz_var1 | 6578 | X03 |
| wiz_var1 | 6579 | AB03 |
| wiz_var1 | 6580 | AF03 |
| wiz_var1 | 6581 | D07 |
| wiz_var1 | 6582 | H07 |
| wiz_var1 | 6583 | L07 |
| wiz_var1 | 6584 | P07 |
| wiz_var1 | 6585 | T07 |
| wiz_var1 | 6586 | X07 |
| wiz_var1 | 6587 | AB07 |
| wiz_var1 | 6588 | AF07 |
| wiz_var1 | 6589 | D11 |
| wiz_var1 | 6590 | H11 |
| wiz_var1 | 6591 | L11 |
| wiz_var1 | 6592 | P11 |
| wiz_var1 | 6593 | T11 |
| wiz_var1 | 6594 | X11 |
| wiz_var1 | 6595 | AB11 |
| wiz_var1 | 6596 | AF11 |
| wiz_var1 | 6597 | D15 |
| wiz_var1 | 6598 | H15 |
| wiz_var1 | 6599 | L15 |

| | | |
|---|---|---|
| wiz_var1 | 6600 | P15 |
| wiz_var1 | 6601 | T15 |
| wiz_var1 | 6602 | X15 |
| wiz_var1 | 6603 | AB15 |
| wiz_var1 | 6604 | AF15 |
| wiz_var1 | 6605 | D19 |
| wiz_var1 | 6606 | H19 |
| wiz_var1 | 6607 | L19 |
| wiz_var1 | 6608 | P19 |
| wiz_var1 | 6609 | T19 |
| wiz_var1 | 6610 | X19 |
| wiz_var1 | 6611 | AB19 |
| wiz_var1 | 6612 | AF19 |
| wiz_var1 | 6613 | D23 |
| wiz_var1 | 6614 | H23 |
| wiz_var1 | 6615 | L23 |
| wiz_var1 | 6616 | P23 |
| wiz_var1 | 6617 | T23 |
| wiz_var1 | 6618 | X23 |
| wiz_var1 | 6619 | AB23 |
| wiz_var1 | 6620 | AF23 |
| wiz_var1 | 6621 | D27 |
| wiz_var1 | 6622 | H27 |
| wiz_var1 | 6623 | L27 |
| wiz_var1 | 6624 | P27 |
| wiz_var1 | 6625 | T27 |
| wiz_var1 | 2815 | X27 |
| wiz_var1 | 2816 | AB27 |
| wiz_var1 | 2817 | AF27 |
| wiz_var1 | 2820 | D31 |
| wiz_var1 | 2821 | H31 |
| wiz_var1 | 2822 | L31 |
| wiz_var1 | 3245 | P31 |
| wiz_var1 | 3246 | T31 |
| wiz_var1 | 3247 | X31 |
| wiz_var1 | 3472 | AB31 |
| wiz_var1 | 3473 | AF31 |
| wiz_var1 | 3474 | D35 |
| wiz_var1 | 3327 | H35 |
| wiz_var1 | 3583 | L35 |
| wiz_var1 | 3584 | P35 |
| wiz_var1 | 3601 | T35 |
| wiz_var1 | 3602 | X35 |
| wiz_var1 | 3603 | AB35 |
| wiz_var1 | 3775 | AF35 |
| wiz_var1 | 3776 | D39 |
| wiz_var1 | 3777 | H39 |
| wiz_var1 | 1808 | L39 |
| wiz_var1 | 1809 | P39 |
| wiz_var1 | 1812 | T39 |

| | | |
|---|---|---|
| wiz_var1 | 1879 | X39 |
| wiz_var1 | 1880 | AB39 |
| wiz_var1 | 1881 | AF39 |
| wiz_var1 | 2360 | D43 |
| wiz_var1 | 2361 | H43 |
| wiz_var1 | 2362 | L43 |
| wiz_var1 | 2492 | P43 |
| wiz_var1 | 2493 | T43 |
| wiz_var1 | 2494 | X43 |
| wiz_var1 | 2579 | AB43 |
| wiz_var1 | 2580 | AF43 |
| wiz_var1 | 2581 | D47 |
| wiz_var1 | 2602 | H47 |
| wiz_var1 | 2603 | L47 |
| wiz_var1 | 2604 | P47 |
| wiz_var1 | 2846 | T47 |
| wiz_var1 | 2847 | X47 |
| wiz_var1 | 2848 | AB47 |
| wiz_var1 | NTC | AF47 |
| ND4_var12 | 6284 | B02 |
| ND4_var12 | 6285 | F02 |
| ND4_var12 | 6286 | J02 |
| ND4_var12 | 6287 | N02 |
| ND4_var12 | 6288 | R02 |
| ND4_var12 | 6289 | V02 |
| ND4_var12 | 6290 | Z02 |
| ND4_var12 | 6291 | AD02 |
| ND4_var12 | 6292 | B06 |
| ND4_var12 | 6293 | F06 |
| ND4_var12 | 6294 | J06 |
| ND4_var12 | 6295 | N06 |
| ND4_var12 | 6296 | R06 |
| ND4_var12 | 6297 | V06 |
| ND4_var12 | 6298 | Z06 |
| ND4_var12 | 6299 | AD06 |
| ND4_var12 | 6300 | B10 |
| ND4_var12 | 6301 | F10 |
| ND4_var12 | 6302 | J10 |
| ND4_var12 | 6303 | N10 |
| ND4_var12 | 6304 | R10 |
| ND4_var12 | 6305 | V10 |
| ND4_var12 | 6306 | Z10 |
| ND4_var12 | 6307 | AD10 |
| ND4_var12 | 6308 | B14 |
| ND4_var12 | 6309 | F14 |
| ND4_var12 | 6310 | J14 |
| ND4_var12 | 6311 | N14 |
| ND4_var12 | 6312 | R14 |
| ND4_var12 | 6313 | V14 |
| ND4_var12 | 6314 | Z14 |

| | | |
|---|---|---|
| ND4_var12 | 6315 | AD14 |
| ND4_var12 | 6316 | B18 |
| ND4_var12 | 6317 | F18 |
| ND4_var12 | 6318 | J18 |
| ND4_var12 | 6319 | N18 |
| ND4_var12 | 6320 | R18 |
| ND4_var12 | 6321 | V18 |
| ND4_var12 | 6322 | Z18 |
| ND4_var12 | 6323 | AD18 |
| ND4_var12 | 6324 | B22 |
| ND4_var12 | 6325 | F22 |
| ND4_var12 | 6326 | J22 |
| ND4_var12 | 6327 | N22 |
| ND4_var12 | 6328 | R22 |
| ND4_var12 | 6329 | V22 |
| ND4_var12 | 6330 | Z22 |
| ND4_var12 | 6331 | AD22 |
| ND4_var12 | 6332 | B26 |
| ND4_var12 | 6333 | F26 |
| ND4_var12 | 6334 | J26 |
| ND4_var12 | 6335 | N26 |
| ND4_var12 | 6336 | R26 |
| ND4_var12 | 6337 | V26 |
| ND4_var12 | 6338 | Z26 |
| ND4_var12 | 6339 | AD26 |
| ND4_var12 | 6340 | B30 |
| ND4_var12 | 6341 | F30 |
| ND4_var12 | 6342 | J30 |
| ND4_var12 | 6343 | N30 |
| ND4_var12 | 6344 | R30 |
| ND4_var12 | 6345 | V30 |
| ND4_var12 | 6346 | Z30 |
| ND4_var12 | 6347 | AD30 |
| ND4_var12 | 6348 | B34 |
| ND4_var12 | 6349 | F34 |
| ND4_var12 | 6350 | J34 |
| ND4_var12 | 6351 | N34 |
| ND4_var12 | 6352 | R34 |
| ND4_var12 | 6353 | V34 |
| ND4_var12 | 6354 | Z34 |
| ND4_var12 | 6355 | AD34 |
| ND4_var12 | 6356 | B38 |
| ND4_var12 | 6357 | F38 |
| ND4_var12 | 6358 | J38 |
| ND4_var12 | 6359 | N38 |
| ND4_var12 | 6360 | R38 |
| ND4_var12 | 6361 | V38 |
| ND4_var12 | 6362 | Z38 |
| ND4_var12 | 6363 | AD38 |
| ND4_var12 | 6364 | B42 |

| | | |
|---|---|---|
| ND4_var12 | 6365 | F42 |
| ND4_var12 | 6366 | J42 |
| ND4_var12 | 6367 | N42 |
| ND4_var12 | 6368 | R42 |
| ND4_var12 | 6369 | V42 |
| ND4_var12 | 6370 | Z42 |
| ND4_var12 | 6371 | AD42 |
| ND4_var12 | 6372 | B46 |
| ND4_var12 | 6373 | F46 |
| ND4_var12 | 6374 | J46 |
| ND4_var12 | 6375 | N46 |
| ND4_var12 | 6376 | R46 |
| ND4_var12 | 6377 | V46 |
| ND4_var12 | 6378 | Z46 |
| ND4_var12 | 6379 | AD46 |
| ND4_var12 | 6380 | B04 |
| ND4_var12 | 6381 | F04 |
| ND4_var12 | 6382 | J04 |
| ND4_var12 | 6383 | N04 |
| ND4_var12 | 6384 | R04 |
| ND4_var12 | 6385 | V04 |
| ND4_var12 | 6386 | Z04 |
| ND4_var12 | 6387 | AD04 |
| ND4_var12 | 6388 | B08 |
| ND4_var12 | 6389 | F08 |
| ND4_var12 | 6390 | J08 |
| ND4_var12 | 6391 | N08 |
| ND4_var12 | 6392 | R08 |
| ND4_var12 | 6393 | V08 |
| ND4_var12 | 6394 | Z08 |
| ND4_var12 | 6395 | AD08 |
| ND4_var12 | 6396 | B12 |
| ND4_var12 | 6397 | F12 |
| ND4_var12 | 6398 | J12 |
| ND4_var12 | 6399 | N12 |
| ND4_var12 | 6400 | R12 |
| ND4_var12 | 6401 | V12 |
| ND4_var12 | 6402 | Z12 |
| ND4_var12 | 6403 | AD12 |
| ND4_var12 | 6404 | B16 |
| ND4_var12 | 6405 | F16 |
| ND4_var12 | 6406 | J16 |
| ND4_var12 | 6407 | N16 |
| ND4_var12 | 6408 | R16 |
| ND4_var12 | 6409 | V16 |
| ND4_var12 | 6410 | Z16 |
| ND4_var12 | 6411 | AD16 |
| ND4_var12 | 6412 | B20 |
| ND4_var12 | 6413 | F20 |
| ND4_var12 | 6414 | J20 |

| | | |
|---|---|---|
| ND4_var12 | 6415 | N20 |
| ND4_var12 | 6416 | R20 |
| ND4_var12 | 6417 | V20 |
| ND4_var12 | 6418 | Z20 |
| ND4_var12 | 6419 | AD20 |
| ND4_var12 | 6421 | B24 |
| ND4_var12 | 6422 | F24 |
| ND4_var12 | 6423 | J24 |
| ND4_var12 | 6424 | N24 |
| ND4_var12 | 6425 | R24 |
| ND4_var12 | 6426 | V24 |
| ND4_var12 | 6427 | Z24 |
| ND4_var12 | 6428 | AD24 |
| ND4_var12 | 6429 | B28 |
| ND4_var12 | 6430 | F28 |
| ND4_var12 | 6431 | J28 |
| ND4_var12 | 6432 | N28 |
| ND4_var12 | 6433 | R28 |
| ND4_var12 | 6434 | V28 |
| ND4_var12 | 6435 | Z28 |
| ND4_var12 | 6436 | AD28 |
| ND4_var12 | 6437 | B32 |
| ND4_var12 | 6438 | F32 |
| ND4_var12 | 6439 | J32 |
| ND4_var12 | 6440 | N32 |
| ND4_var12 | 6441 | R32 |
| ND4_var12 | 6442 | V32 |
| ND4_var12 | 6443 | Z32 |
| ND4_var12 | 6444 | AD32 |
| ND4_var12 | 6445 | B36 |
| ND4_var12 | 6446 | F36 |
| ND4_var12 | 6447 | J36 |
| ND4_var12 | 6448 | N36 |
| ND4_var12 | 6449 | R36 |
| ND4_var12 | 6450 | V36 |
| ND4_var12 | 6451 | Z36 |
| ND4_var12 | 6452 | AD36 |
| ND4_var12 | 6453 | B40 |
| ND4_var12 | 6454 | F40 |
| ND4_var12 | 6455 | J40 |
| ND4_var12 | 6456 | N40 |
| ND4_var12 | 6457 | R40 |
| ND4_var12 | 6458 | V40 |
| ND4_var12 | 6459 | Z40 |
| ND4_var12 | 6460 | AD40 |
| ND4_var12 | 6461 | B44 |
| ND4_var12 | 6462 | F44 |
| ND4_var12 | 6463 | J44 |
| ND4_var12 | 6464 | N44 |
| ND4_var12 | 6465 | R44 |

| | | |
|---|---|---|
| ND4_var12 | 6466 | V44 |
| ND4_var12 | 6467 | Z44 |
| ND4_var12 | 6468 | AD44 |
| ND4_var12 | 6469 | B48 |
| ND4_var12 | 6470 | F48 |
| ND4_var12 | 6471 | J48 |
| ND4_var12 | 6472 | N48 |
| ND4_var12 | 6473 | R48 |
| ND4_var12 | 6474 | V48 |
| ND4_var12 | 6475 | Z48 |
| ND4_var12 | 6476 | AD48 |
| ND4_var12 | 6477 | D02 |
| ND4_var12 | 6478 | H02 |
| ND4_var12 | 6479 | L02 |
| ND4_var12 | 6480 | P02 |
| ND4_var12 | 6481 | T02 |
| ND4_var12 | 6482 | X02 |
| ND4_var12 | 6483 | AB02 |
| ND4_var12 | 6484 | AF02 |
| ND4_var12 | 6485 | D06 |
| ND4_var12 | 6486 | H06 |
| ND4_var12 | 6487 | L06 |
| ND4_var12 | 6488 | P06 |
| ND4_var12 | 6489 | T06 |
| ND4_var12 | 6490 | X06 |
| ND4_var12 | 6491 | AB06 |
| ND4_var12 | 6492 | AF06 |
| ND4_var12 | 6493 | D10 |
| ND4_var12 | 6494 | H10 |
| ND4_var12 | 6495 | L10 |
| ND4_var12 | 6496 | P10 |
| ND4_var12 | 6497 | T10 |
| ND4_var12 | 6498 | X10 |
| ND4_var12 | 6499 | AB10 |
| ND4_var12 | 6500 | AF10 |
| ND4_var12 | 6501 | D14 |
| ND4_var12 | 6502 | H14 |
| ND4_var12 | 6503 | L14 |
| ND4_var12 | 6504 | P14 |
| ND4_var12 | 6505 | T14 |
| ND4_var12 | 6506 | X14 |
| ND4_var12 | 6507 | AB14 |
| ND4_var12 | 6508 | AF14 |
| ND4_var12 | 6509 | D18 |
| ND4_var12 | 6510 | H18 |
| ND4_var12 | 6511 | L18 |
| ND4_var12 | 6512 | P18 |
| ND4_var12 | 6513 | T18 |
| ND4_var12 | 6514 | X18 |
| ND4_var12 | 6515 | AB18 |

| | | |
|---|---|---|
| ND4_var12 | 6516 | AF18 |
| ND4_var12 | 6517 | D22 |
| ND4_var12 | 6518 | H22 |
| ND4_var12 | 6519 | L22 |
| ND4_var12 | 6520 | P22 |
| ND4_var12 | 6521 | T22 |
| ND4_var12 | 6522 | X22 |
| ND4_var12 | 6523 | AB22 |
| ND4_var12 | 6524 | AF22 |
| ND4_var12 | 6525 | D26 |
| ND4_var12 | 6526 | H26 |
| ND4_var12 | 6527 | L26 |
| ND4_var12 | 6528 | P26 |
| ND4_var12 | 6529 | T26 |
| ND4_var12 | 6530 | X26 |
| ND4_var12 | 6531 | AB26 |
| ND4_var12 | 6532 | AF26 |
| ND4_var12 | 6533 | D30 |
| ND4_var12 | 6534 | H30 |
| ND4_var12 | 6535 | L30 |
| ND4_var12 | 6536 | P30 |
| ND4_var12 | 6537 | T30 |
| ND4_var12 | 6538 | X30 |
| ND4_var12 | 6539 | AB30 |
| ND4_var12 | 6540 | AF30 |
| ND4_var12 | 6541 | D34 |
| ND4_var12 | 6542 | H34 |
| ND4_var12 | 6543 | L34 |
| ND4_var12 | 6544 | P34 |
| ND4_var12 | 6545 | T34 |
| ND4_var12 | 6546 | X34 |
| ND4_var12 | 6547 | AB34 |
| ND4_var12 | 6548 | AF34 |
| ND4_var12 | 6549 | D38 |
| ND4_var12 | 6550 | H38 |
| ND4_var12 | 6551 | L38 |
| ND4_var12 | 6552 | P38 |
| ND4_var12 | 6553 | T38 |
| ND4_var12 | 6554 | X38 |
| ND4_var12 | 6555 | AB38 |
| ND4_var12 | 6556 | AF38 |
| ND4_var12 | 6557 | D42 |
| ND4_var12 | 6558 | H42 |
| ND4_var12 | 6559 | L42 |
| ND4_var12 | 6560 | P42 |
| ND4_var12 | 6561 | T42 |
| ND4_var12 | 6562 | X42 |
| ND4_var12 | 6563 | AB42 |
| ND4_var12 | 6564 | AF42 |
| ND4_var12 | 6565 | D46 |

| | | |
|---|---|---|
| ND4_var12 | 6566 | H46 |
| ND4_var12 | 6567 | L46 |
| ND4_var12 | 6568 | P46 |
| ND4_var12 | 6569 | T46 |
| ND4_var12 | 6570 | X46 |
| ND4_var12 | 6571 | AB46 |
| ND4_var12 | 6572 | AF46 |
| ND4_var12 | 6573 | D04 |
| ND4_var12 | 6574 | H04 |
| ND4_var12 | 6575 | L04 |
| ND4_var12 | 6576 | P04 |
| ND4_var12 | 6577 | T04 |
| ND4_var12 | 6578 | X04 |
| ND4_var12 | 6579 | AB04 |
| ND4_var12 | 6580 | AF04 |
| ND4_var12 | 6581 | D08 |
| ND4_var12 | 6582 | H08 |
| ND4_var12 | 6583 | L08 |
| ND4_var12 | 6584 | P08 |
| ND4_var12 | 6585 | T08 |
| ND4_var12 | 6586 | X08 |
| ND4_var12 | 6587 | AB08 |
| ND4_var12 | 6588 | AF08 |
| ND4_var12 | 6589 | D12 |
| ND4_var12 | 6590 | H12 |
| ND4_var12 | 6591 | L12 |
| ND4_var12 | 6592 | P12 |
| ND4_var12 | 6593 | T12 |
| ND4_var12 | 6594 | X12 |
| ND4_var12 | 6595 | AB12 |
| ND4_var12 | 6596 | AF12 |
| ND4_var12 | 6597 | D16 |
| ND4_var12 | 6598 | H16 |
| ND4_var12 | 6599 | L16 |
| ND4_var12 | 6600 | P16 |
| ND4_var12 | 6601 | T16 |
| ND4_var12 | 6602 | X16 |
| ND4_var12 | 6603 | AB16 |
| ND4_var12 | 6604 | AF16 |
| ND4_var12 | 6605 | D20 |
| ND4_var12 | 6606 | H20 |
| ND4_var12 | 6607 | L20 |
| ND4_var12 | 6608 | P20 |
| ND4_var12 | 6609 | T20 |
| ND4_var12 | 6610 | X20 |
| ND4_var12 | 6611 | AB20 |
| ND4_var12 | 6612 | AF20 |
| ND4_var12 | 6613 | D24 |
| ND4_var12 | 6614 | H24 |
| ND4_var12 | 6615 | L24 |

| | | |
|---|---|---|
| ND4_var12 | 6616 | P24 |
| ND4_var12 | 6617 | T24 |
| ND4_var12 | 6618 | X24 |
| ND4_var12 | 6619 | AB24 |
| ND4_var12 | 6620 | AF24 |
| ND4_var12 | 6621 | D28 |
| ND4_var12 | 6622 | H28 |
| ND4_var12 | 6623 | L28 |
| ND4_var12 | 6624 | P28 |
| ND4_var12 | 6625 | T28 |
| ND4_var12 | 2815 | X28 |
| ND4_var12 | 2816 | AB28 |
| ND4_var12 | 2817 | AF28 |
| ND4_var12 | 2820 | D32 |
| ND4_var12 | 2821 | H32 |
| ND4_var12 | 2822 | L32 |
| ND4_var12 | 3245 | P32 |
| ND4_var12 | 3246 | T32 |
| ND4_var12 | 3247 | X32 |
| ND4_var12 | 3472 | AB32 |
| ND4_var12 | 3473 | AF32 |
| ND4_var12 | 3474 | D36 |
| ND4_var12 | 3327 | H36 |
| ND4_var12 | 3583 | L36 |
| ND4_var12 | 3584 | P36 |
| ND4_var12 | 3601 | T36 |
| ND4_var12 | 3602 | X36 |
| ND4_var12 | 3603 | AB36 |
| ND4_var12 | 3775 | AF36 |
| ND4_var12 | 3776 | D40 |
| ND4_var12 | 3777 | H40 |
| ND4_var12 | 1808 | L40 |
| ND4_var12 | 1809 | P40 |
| ND4_var12 | 1812 | T40 |
| ND4_var12 | 1879 | X40 |
| ND4_var12 | 1880 | AB40 |
| ND4_var12 | 1881 | AF40 |
| ND4_var12 | 2360 | D44 |
| ND4_var12 | 2361 | H44 |
| ND4_var12 | 2362 | L44 |
| ND4_var12 | 2492 | P44 |
| ND4_var12 | 2493 | T44 |
| ND4_var12 | 2494 | X44 |
| ND4_var12 | 2579 | AB44 |
| ND4_var12 | 2580 | AF44 |
| ND4_var12 | 2581 | D48 |
| ND4_var12 | 2602 | H48 |
| ND4_var12 | 2603 | L48 |
| ND4_var12 | 2604 | P48 |
| ND4_var12 | 2846 | T48 |

| | | |
|---|---|---|
| ND4_var12 | 2847 | X48 |
| ND4_var12 | 2848 | AB48 |
| ND4_var12 | NTC | AF48 |

| | |
|---|---|
| C | G |
| C | G |
| C | G |
| C | G |
| - | C |
| - | C |
| - | C |
| - | C |
| C | G |
| C | G |
| C | G |
| C | G |
| C | T |
| C | T |
| C | T |
| C | T |
| G | A |
| G | A |
| G | A |
| G | A |
| A | C |
| A | C |
| A | C |
| A | C |
| C | G |
| C | G |
| C | G |
| C | G |
| C | A |
| C | A |
| C | A |
| C | A |
| G | A |
| G | A |
| G | A |
| G | A |

| | |
|---|---|
| T | G |
| T | G |
| T | G |
| T | G |
| T | C |
| T | C |
| T | C |
| T | C |
| T | C |
| T | C |
| T | C |
| T | C |
| A | G |
| A | G |
| A | G |
| A | G |
| T | A |
| T | A |
| T | A |
| T | A |
| C | A |
| C | A |
| C | A |
| C | A |
| G | A |
| G | A |
| G | A |
| G | A |
| C | T |
| C | T |
| C | T |
| C | T |
| T | C |
| T | C |
| T | C |
| T | C |

Assay:arh_Sheffield_var1 DNA:Vlasi-1+Vlasi-2+Vlasi-3+Vlasi-4

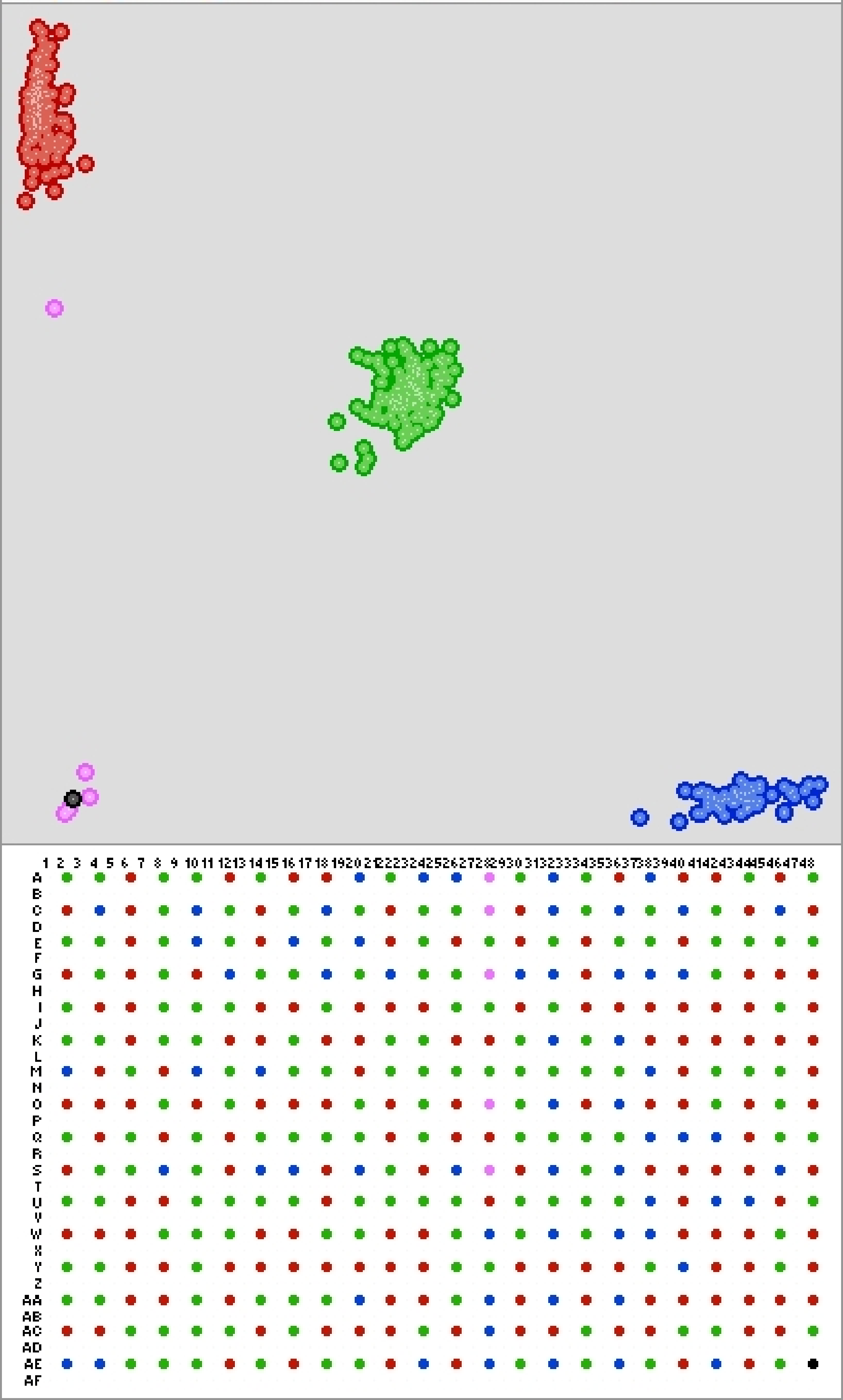

Assay:clasp2_Sheffield_var1 DNA:Vlasi-1+Vlasi-2+Vlasi-3+Vlasi-4

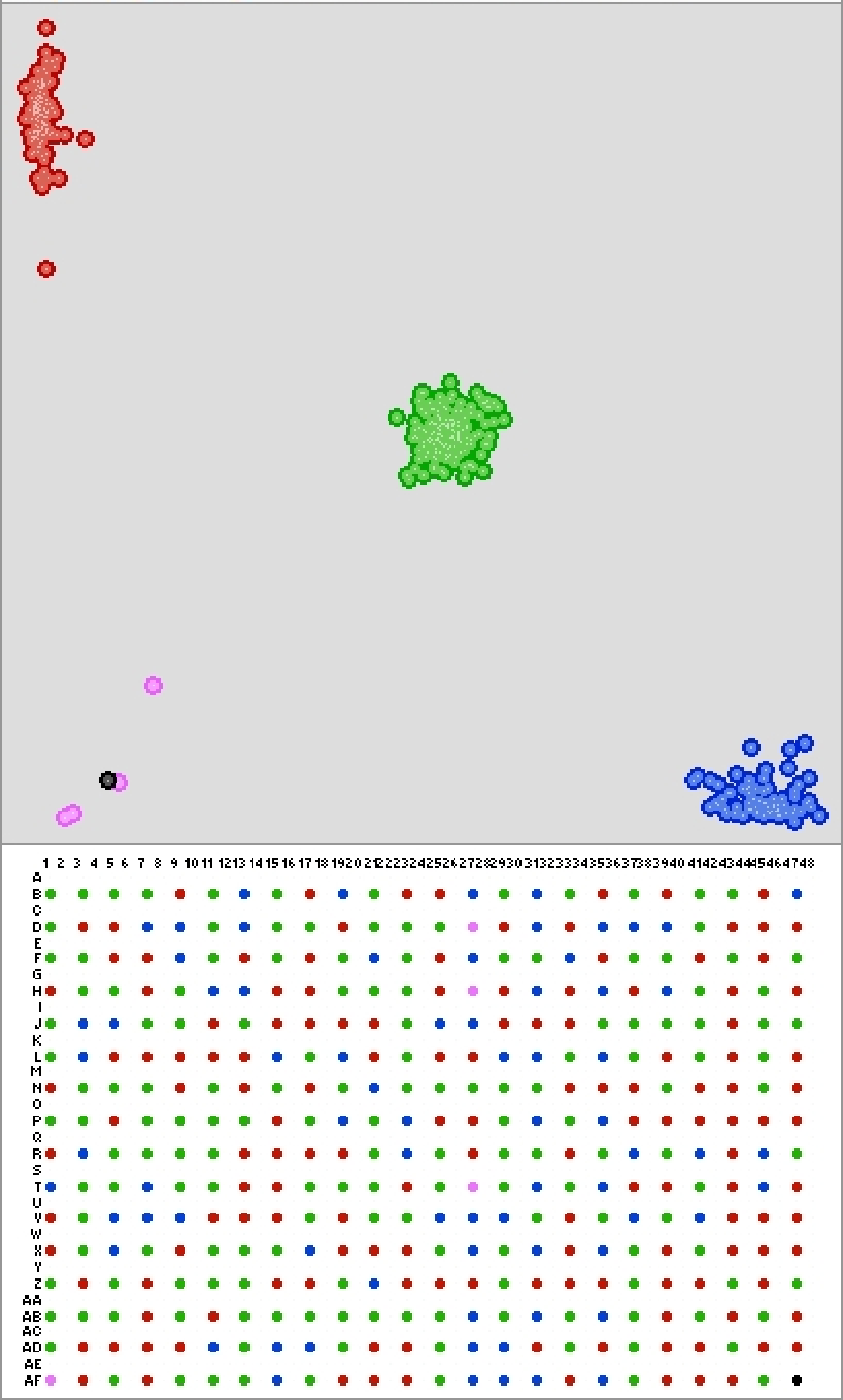

Assay:col18_Sheffield_var1 DNA:Vlasi-1+Vlasi-2+Vlasi-3+Vlasi-4

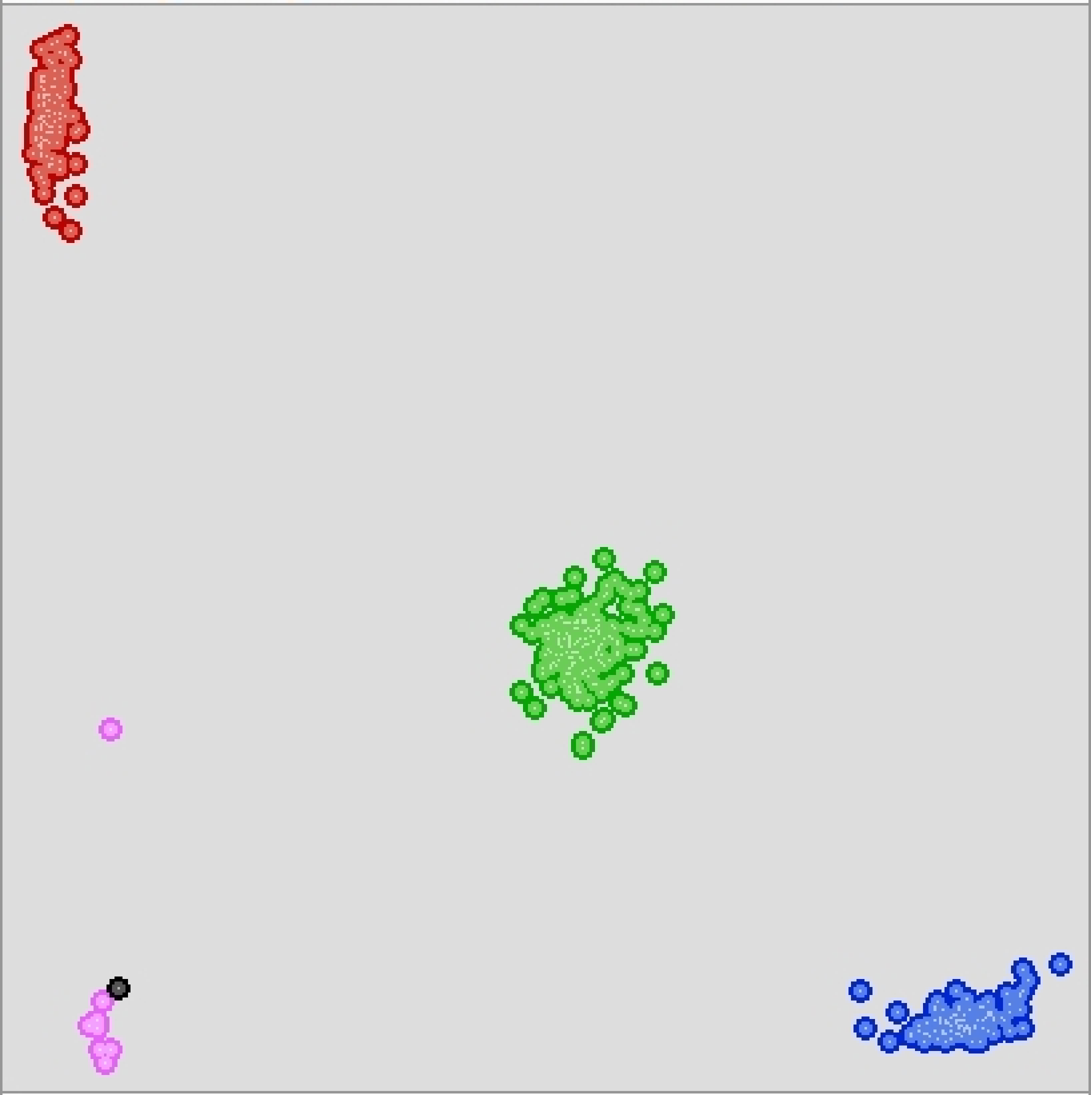

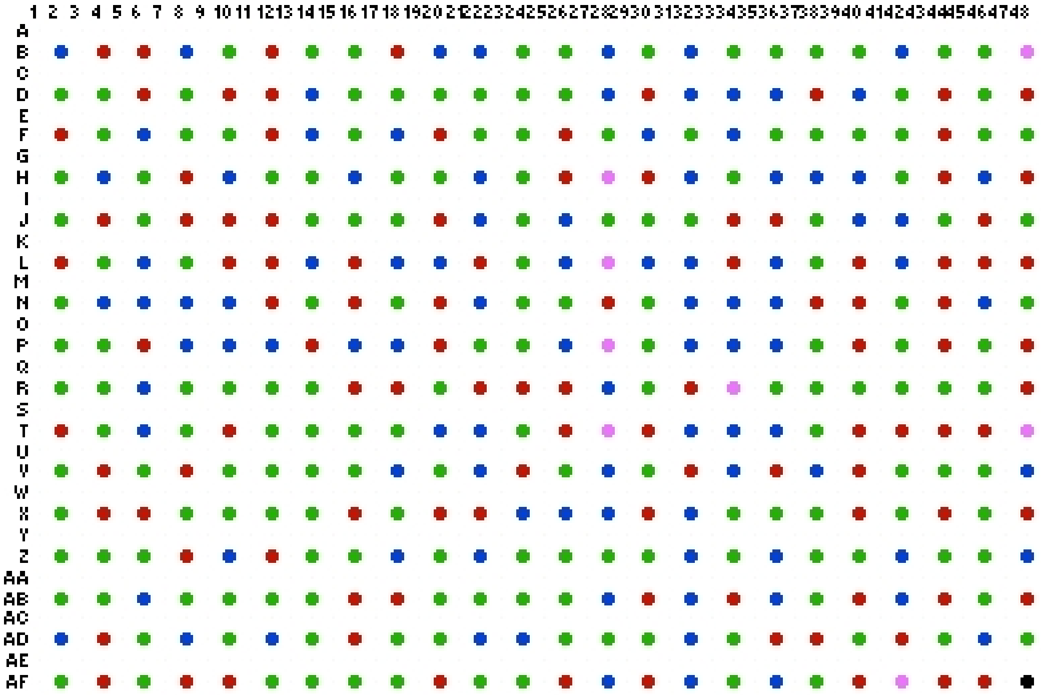

Assay:ddx17_Sheffield_var1 DNA:Vlasi-1+Vlasi-2+Vlasi-3+Vlasi-4

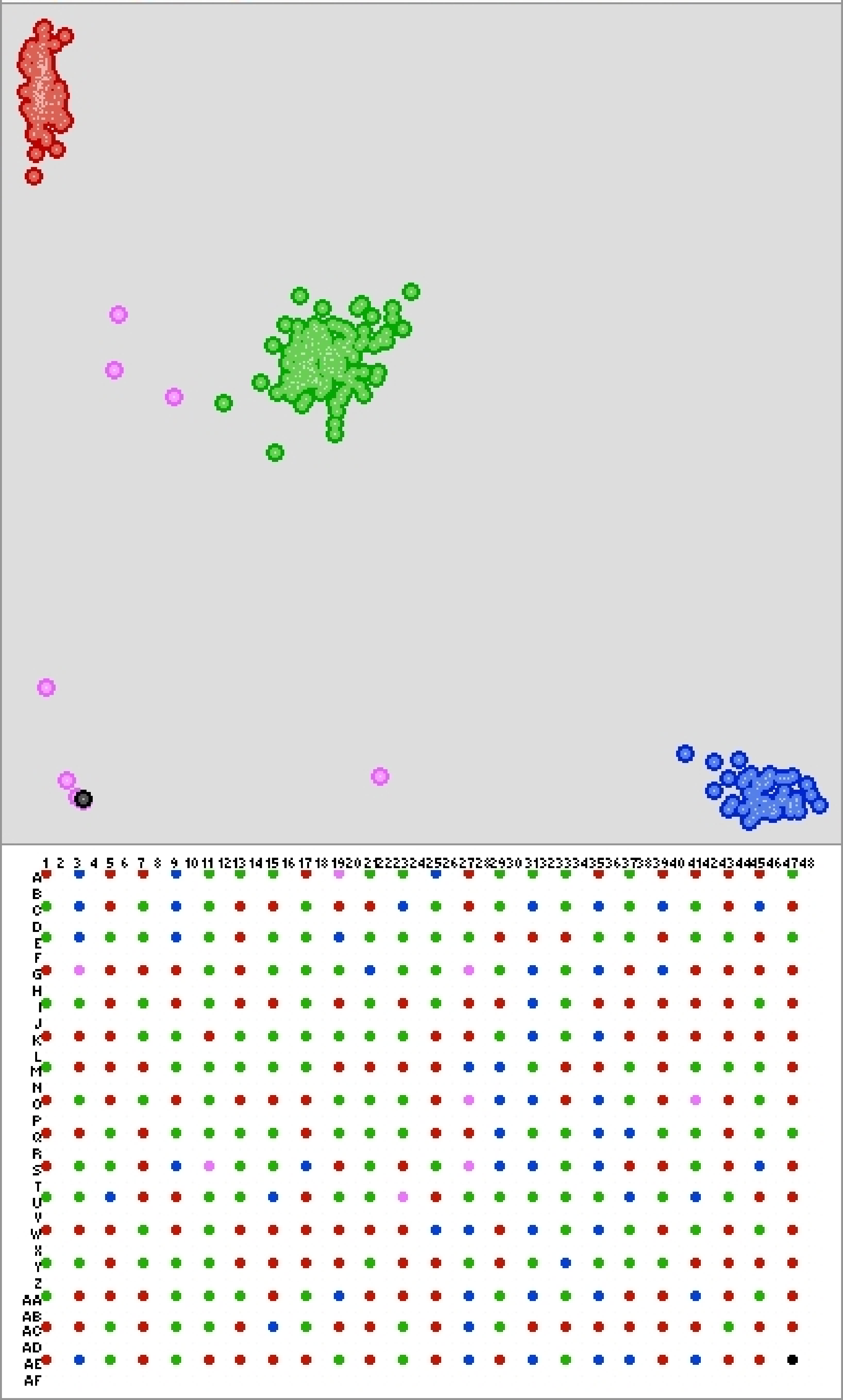

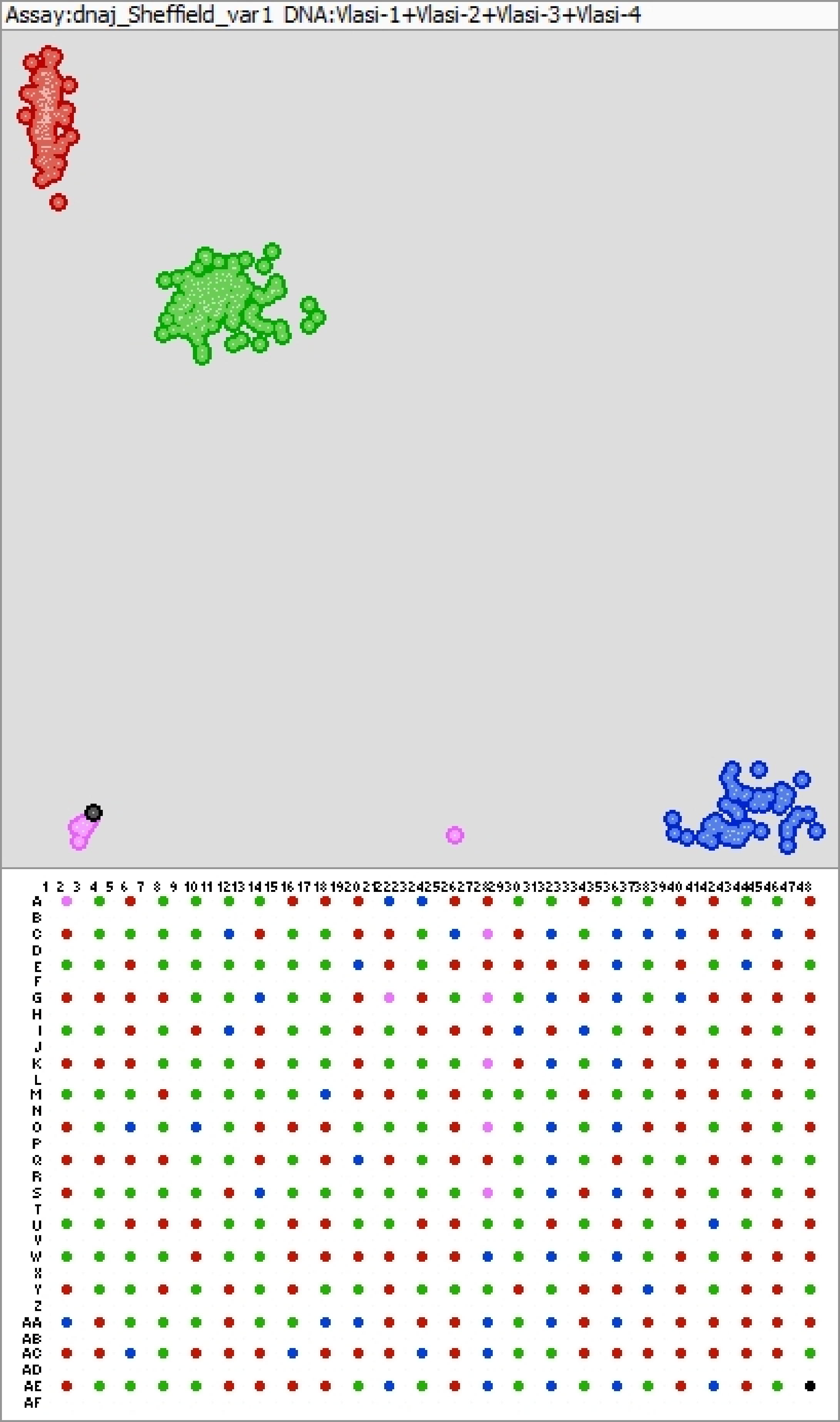
Assay:dnaj_Sheffield_var1 DNA:Vlasi-1+Vlasi-2+Vlasi-3+Vlasi-4
1 2 3 4 5 6 7 8 9 10 11 12 13 14 15 16 17 18 19 20 21 22 23 24 25 26 27 28 29 30 31 32 33 34 35 36 37 38 39 40 41 42 43 44 45 46 47 48
A B C D E F G H I J K L M N O P Q R S T U V W X Y Z AA AB AC AD AE AF

Assay:fam178_Sheffield_var1 DNA:Vlasi-1+Vlasi-2+Vlasi-3+Vlasi-4

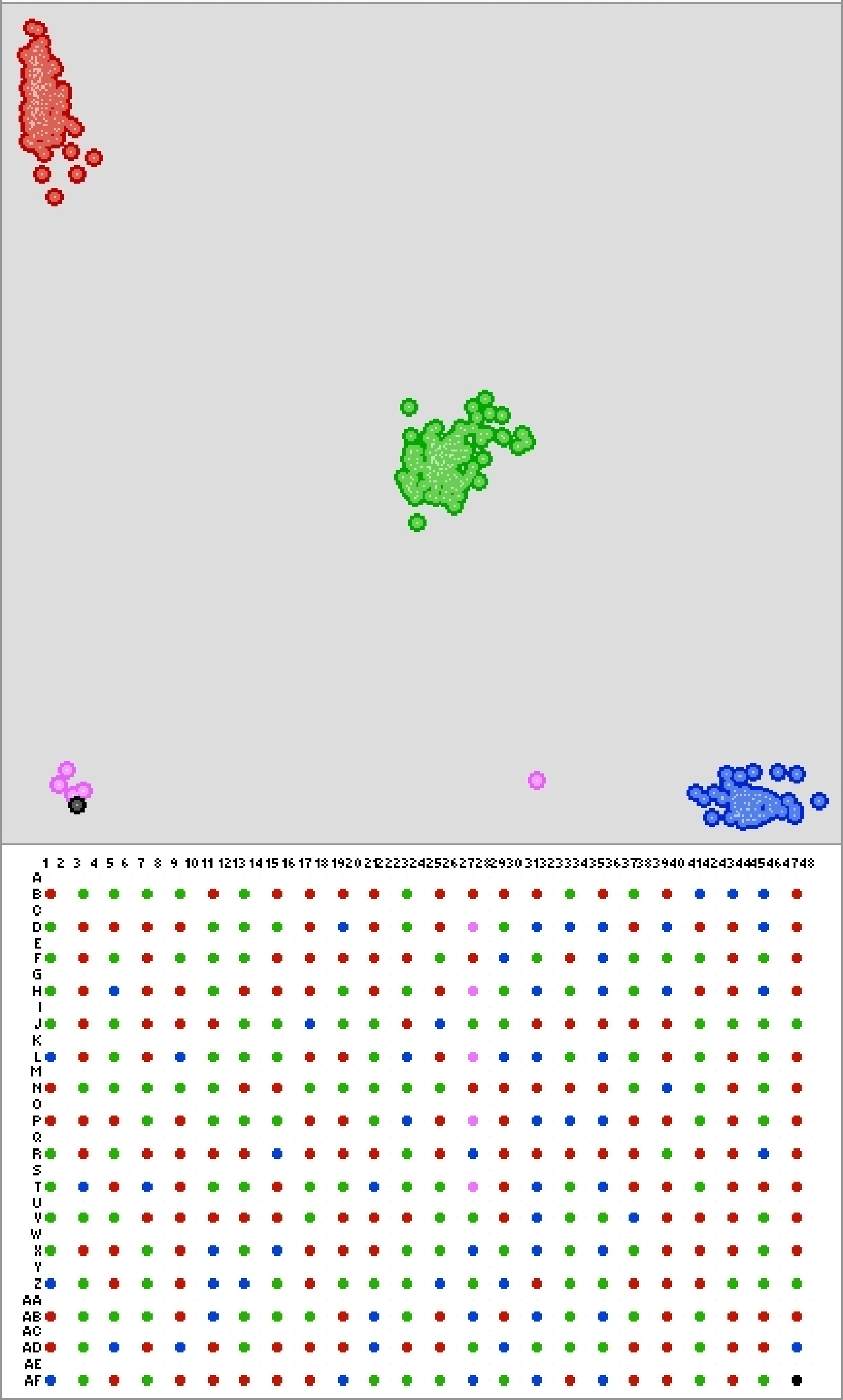

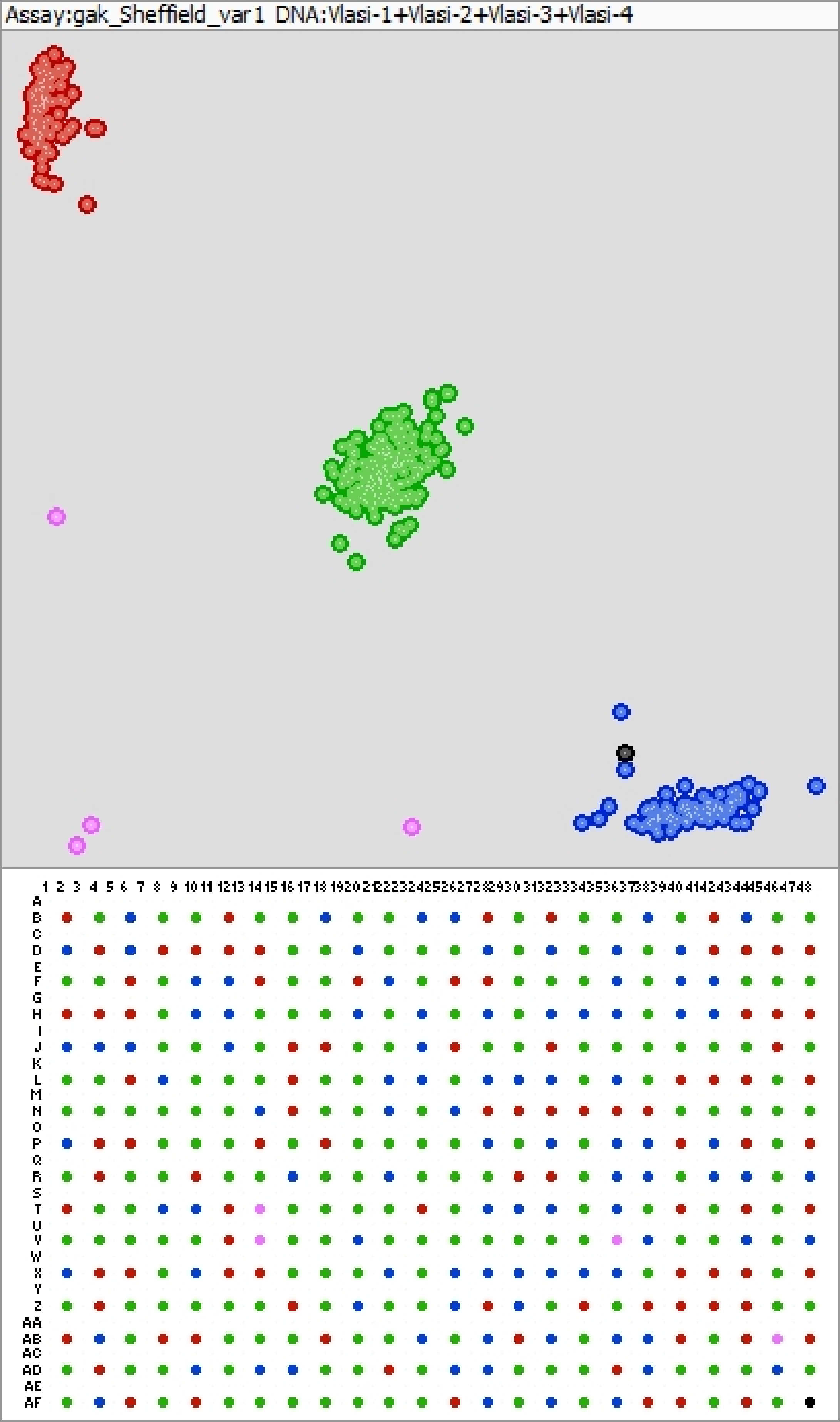
Assay:gak_Sheffield_var1 DNA:Vlasi-1+Vlasi-2+Vlasi-3+Vlasi-4

Assay:ganab_Sheffield_var1 DNA:Vlasi-1+Vlasi-2+Vlasi-3+Vlasi-4

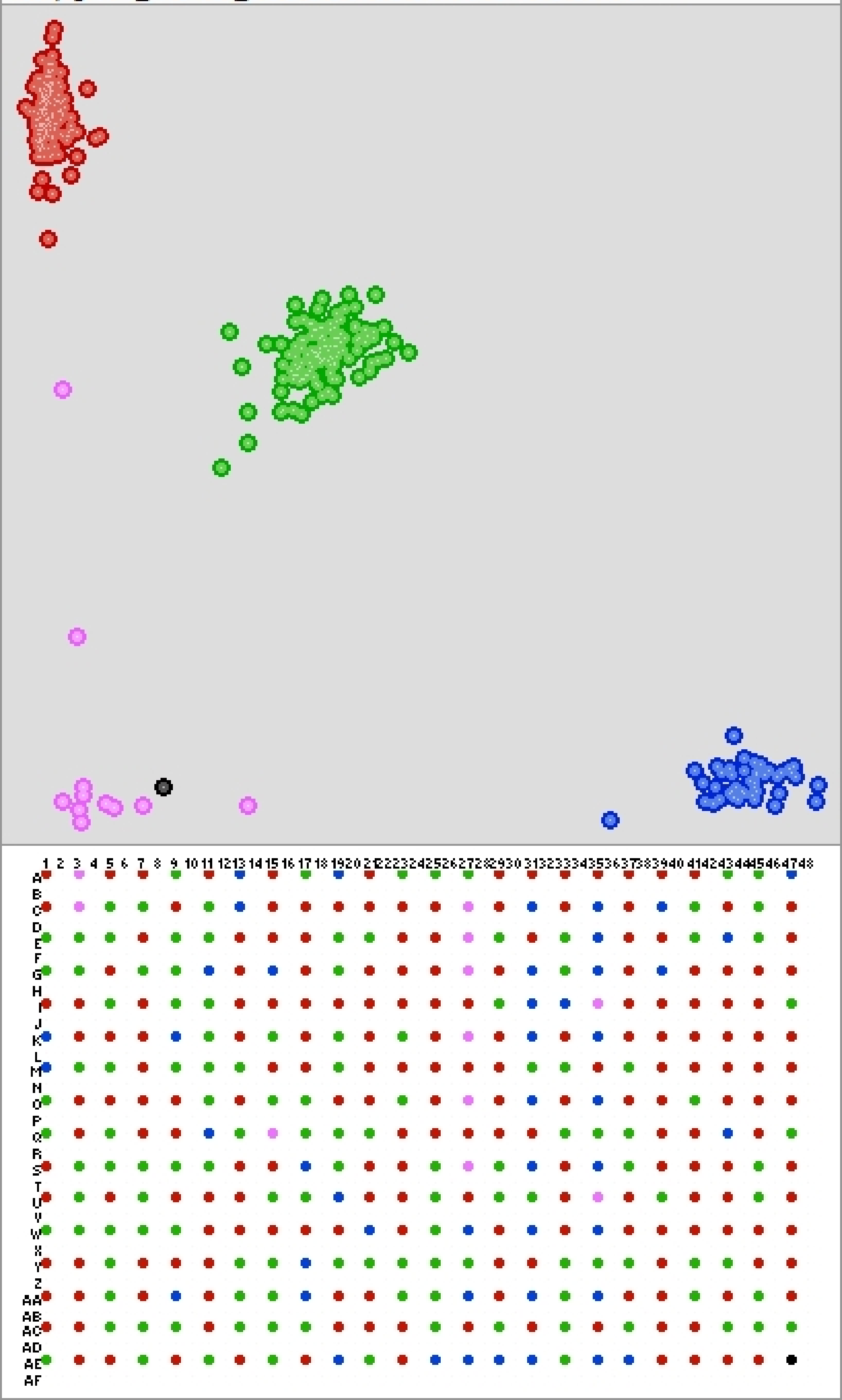

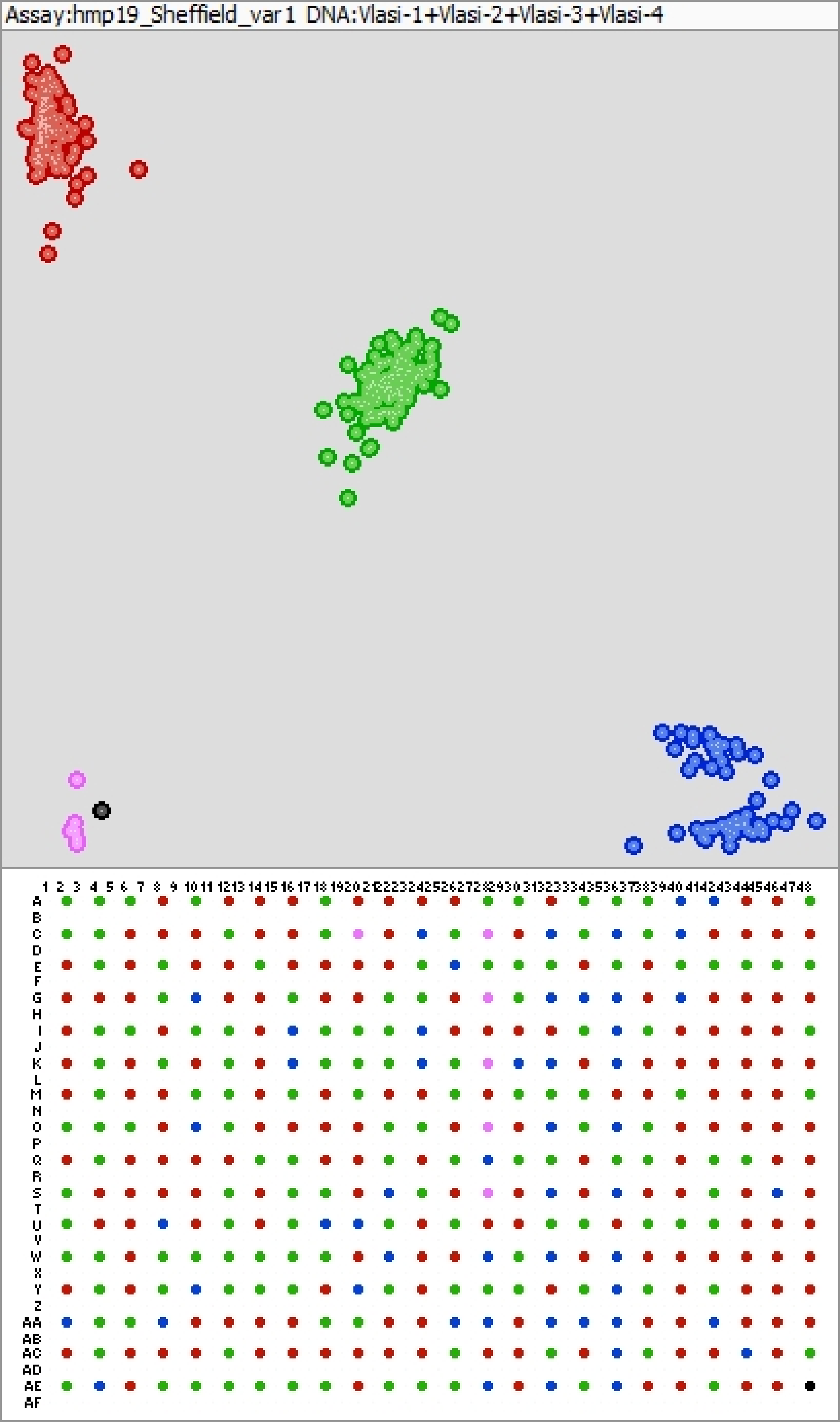
Assay:hmp19_Sheffield_var1 DNA:Vlasi-1+Vlasi-2+Vlasi-3+Vlasi-4

Assay:msantd4_Sheffield_var1 DNA:Vlasi-1+Vlasi-2+Vlasi-3+Vlasi-4

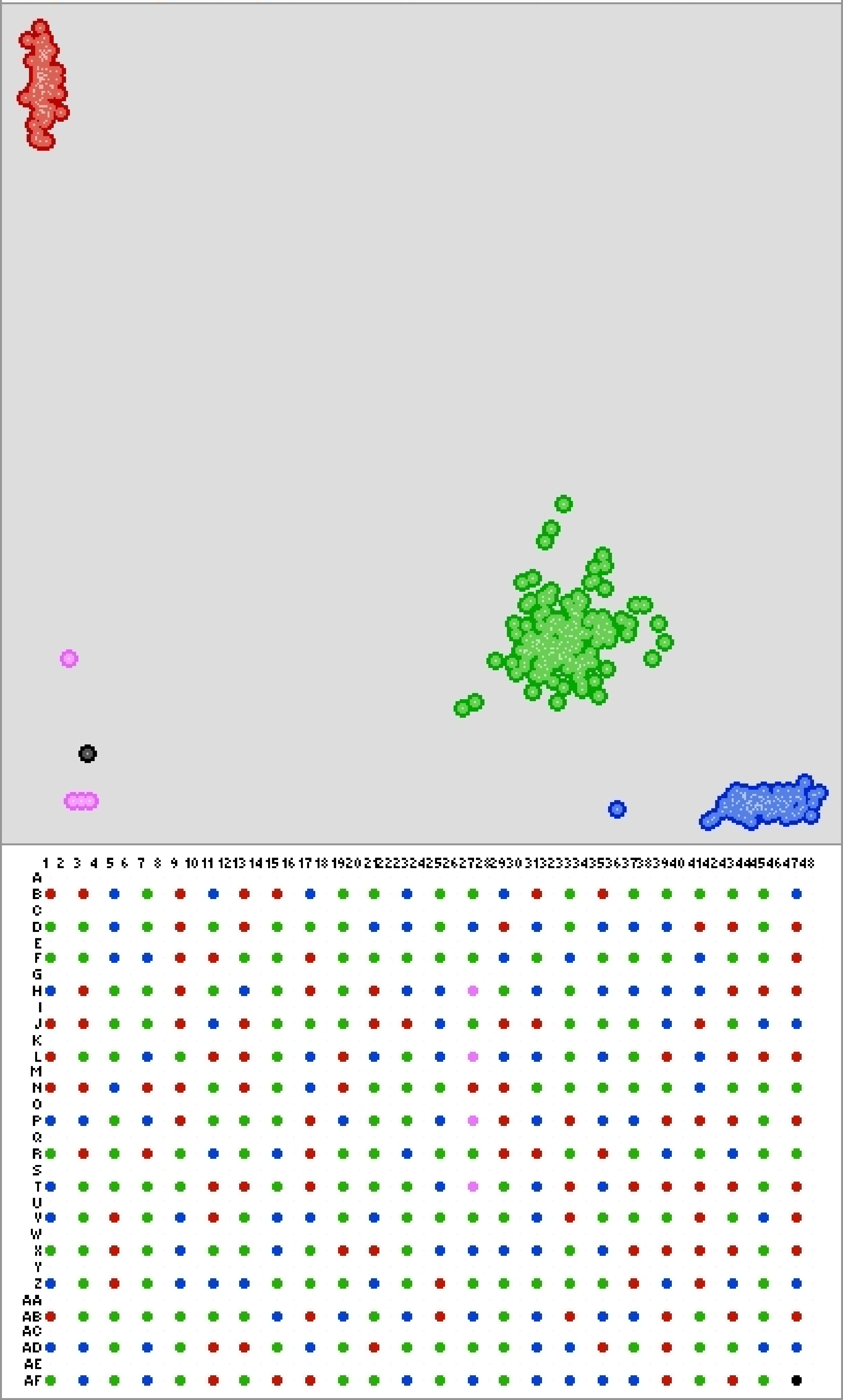

Assay:ND4_Sheffield_var12 DNA:Vlasi-1+Vlasi-2+Vlasi-3+Vlasi-4

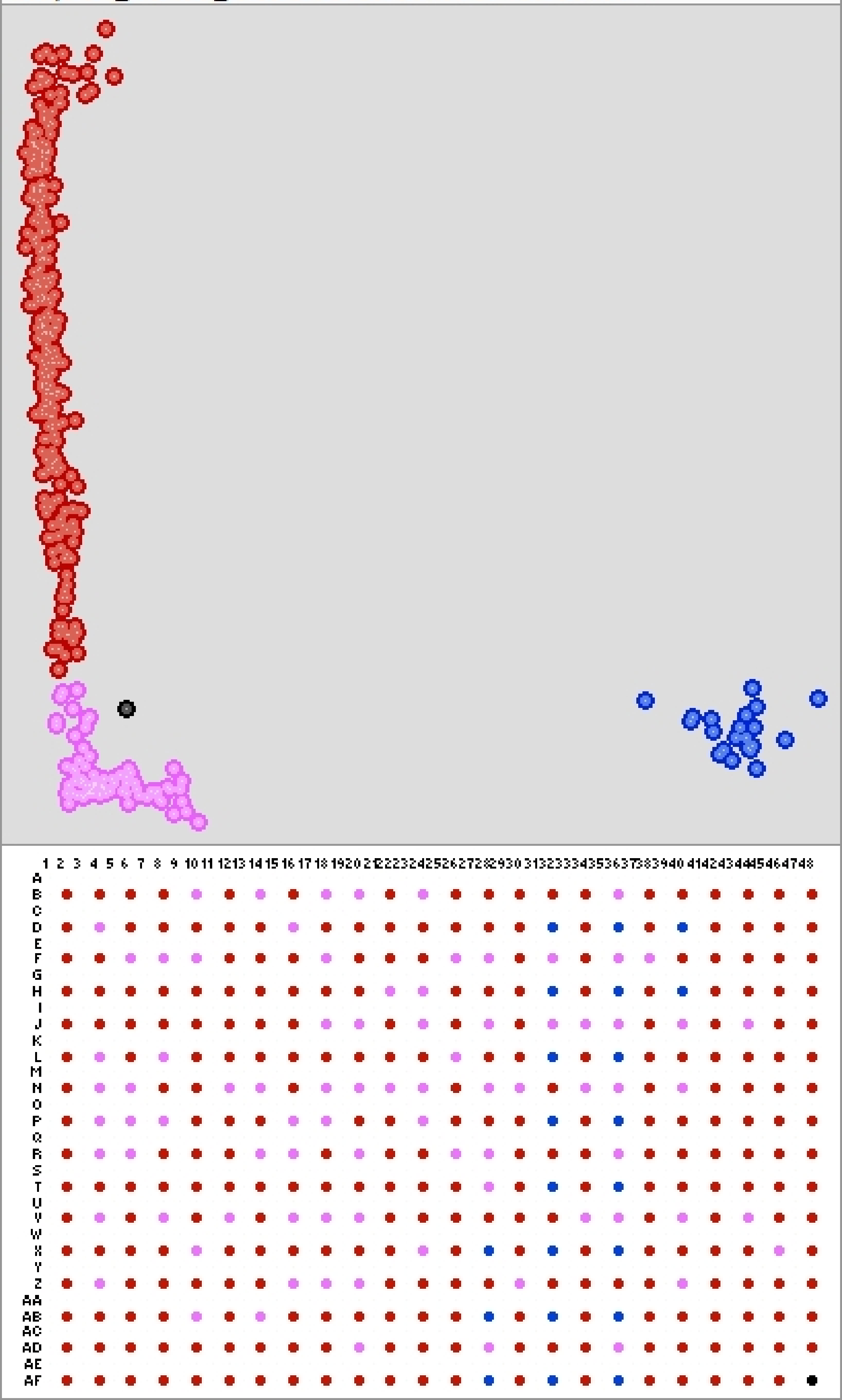

Assay:opa_Sheffield_var1 DNA:Vlasi-1+Vlasi-2+Vlasi-3+Vlasi-4

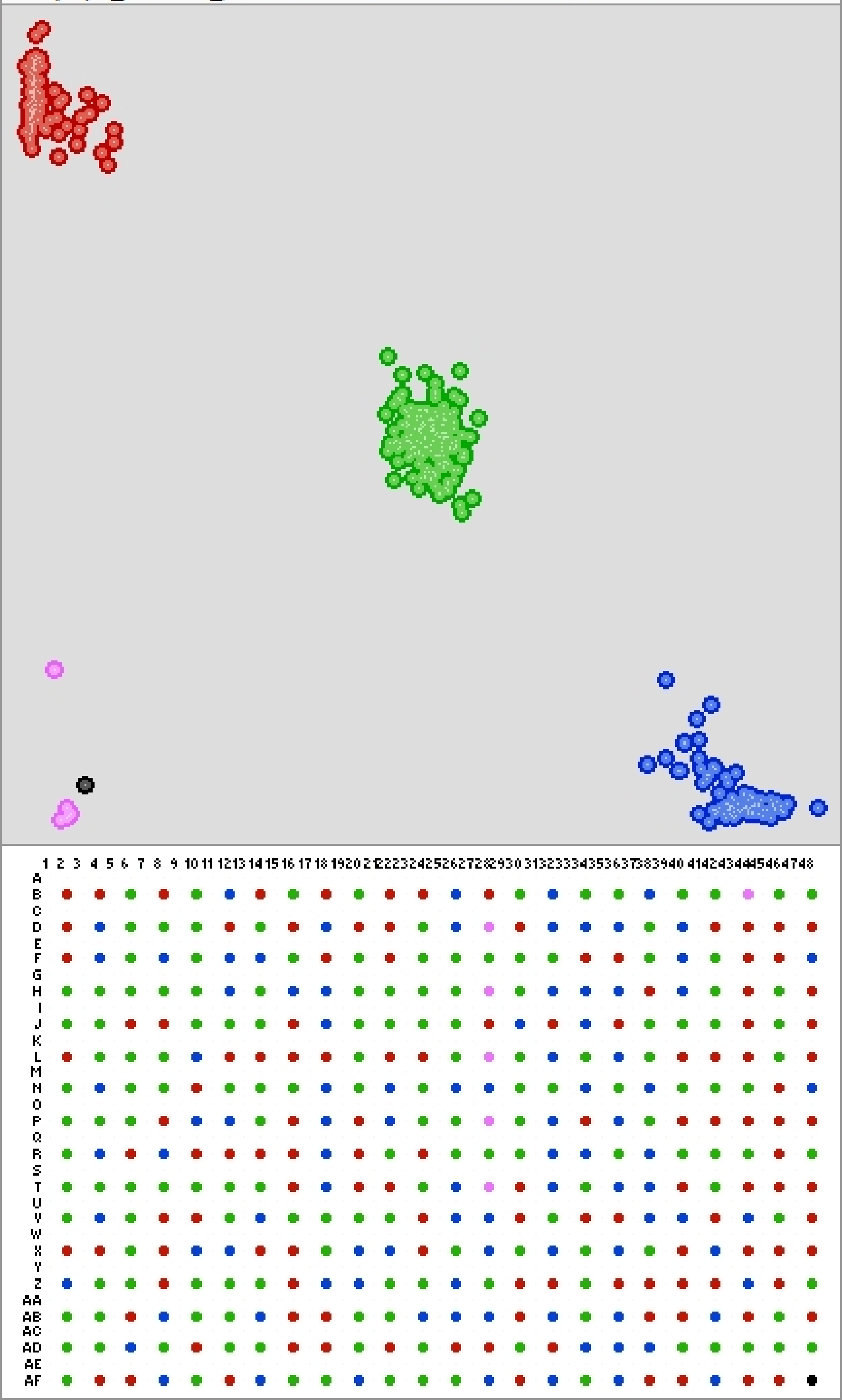

Assay:plekhg1_Sheffield_var1 DNA:Vlasi-1+Vlasi-2+Vlasi-3+Vlasi-4

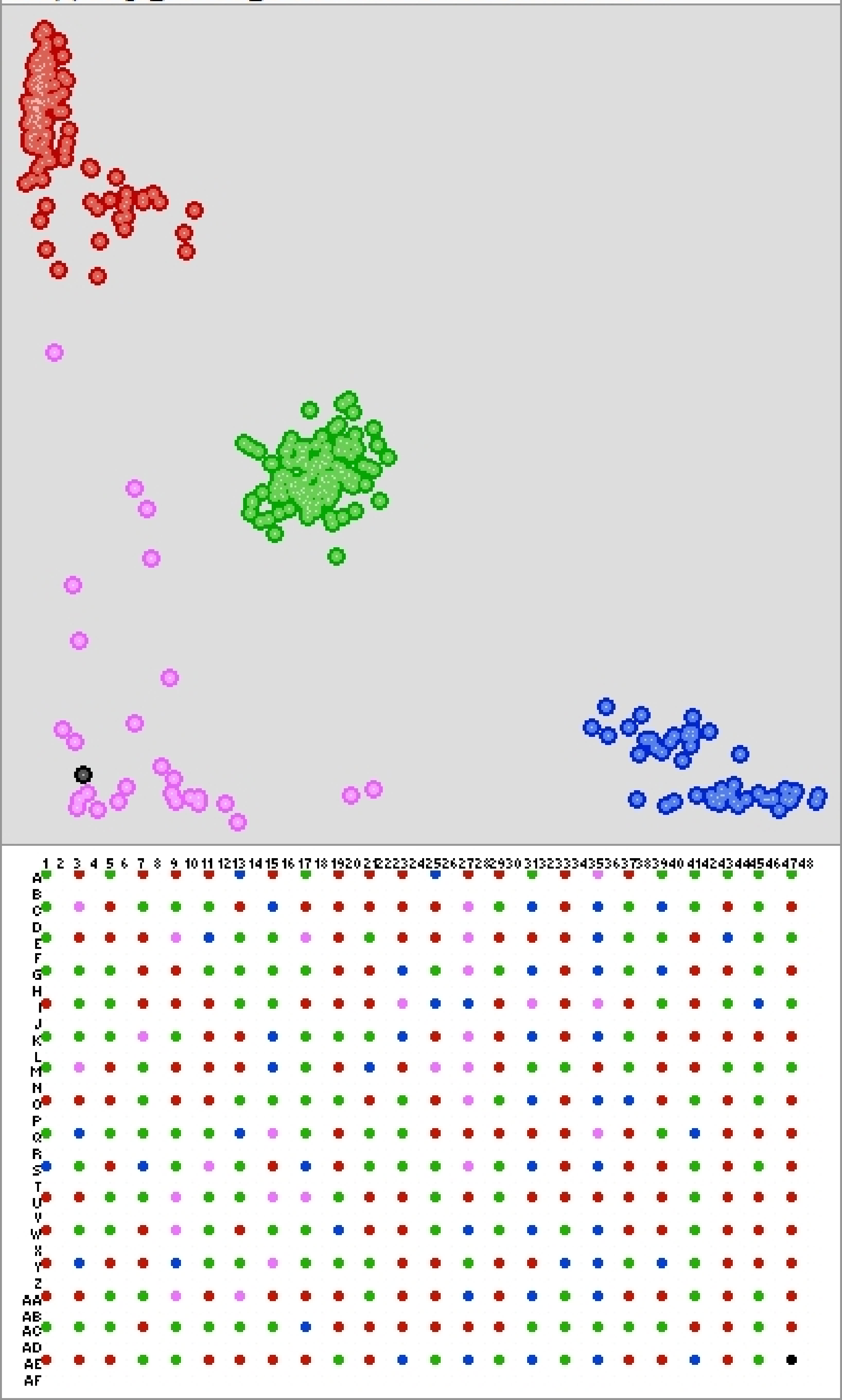

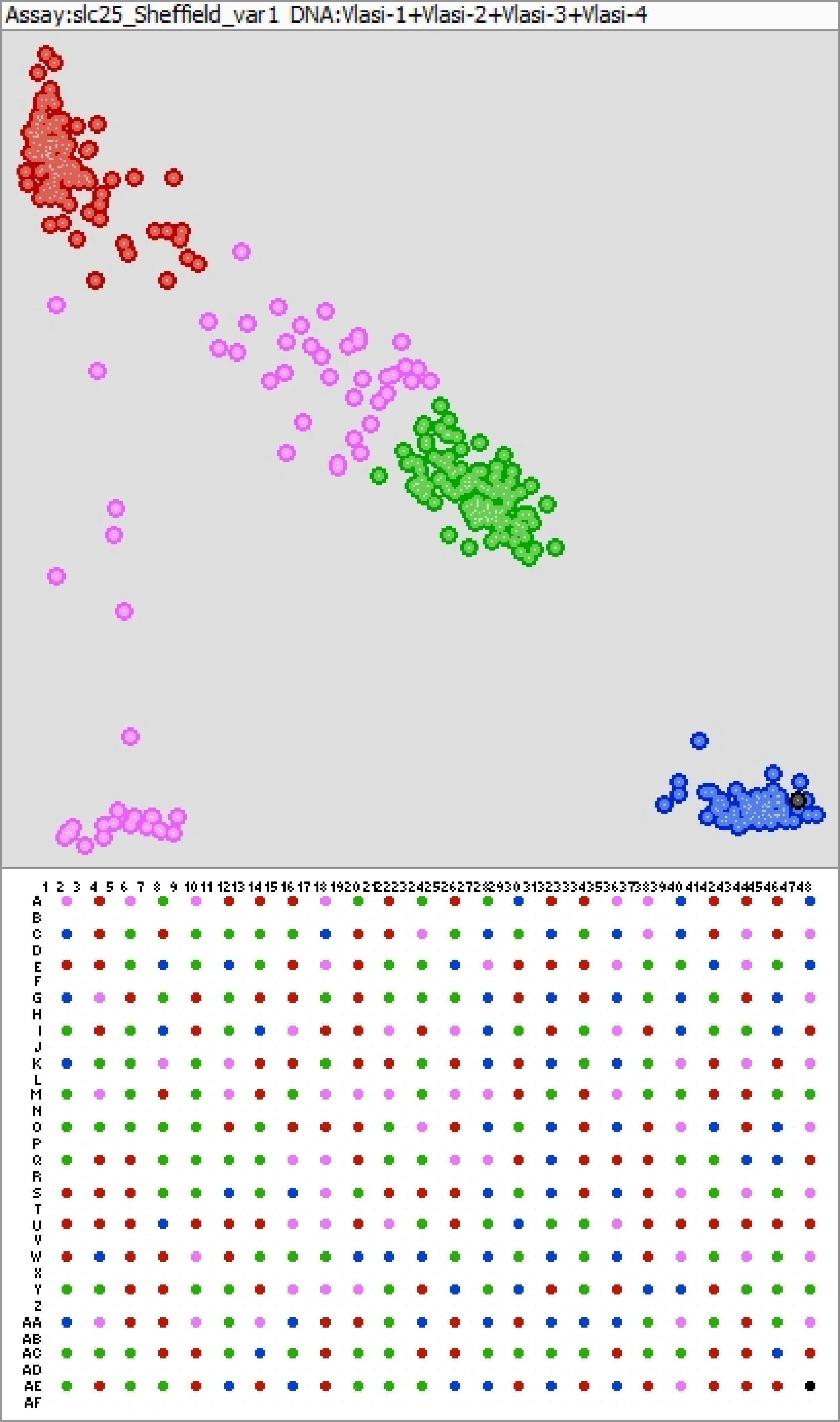
Assay:slc25_Sheffield_var1 DNA:Vlasi-1+Vlasi-2+Vlasi-3+Vlasi-4

Assay:sre_Sheffield_var1 DNA:Vlasi-1+Vlasi-2+Vlasi-3+Vlasi-4

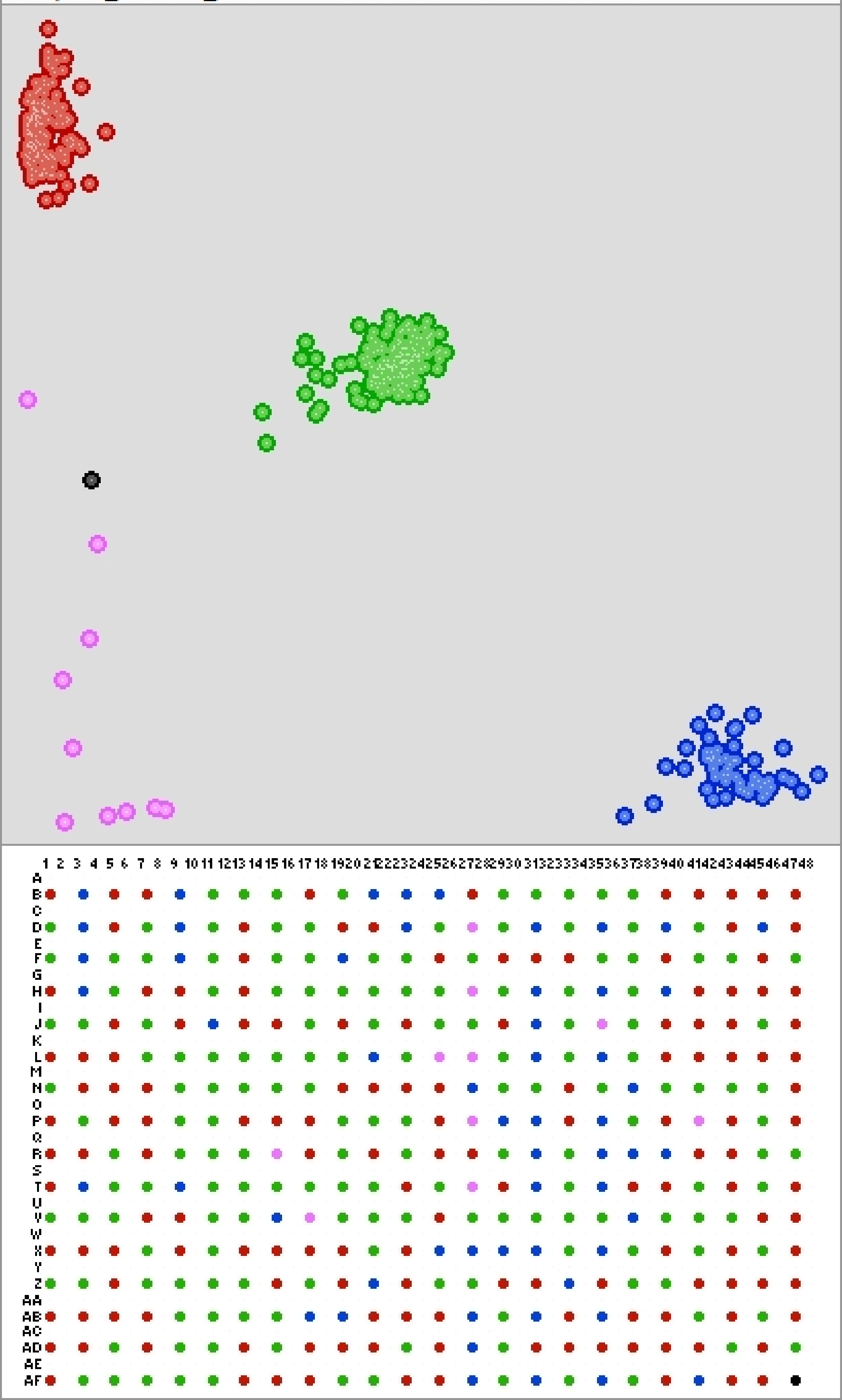

Assay:supt6h_Sheffield_var1 DNA:Vlasi-1+Vlasi-2+Vlasi-3+Vlasi-4

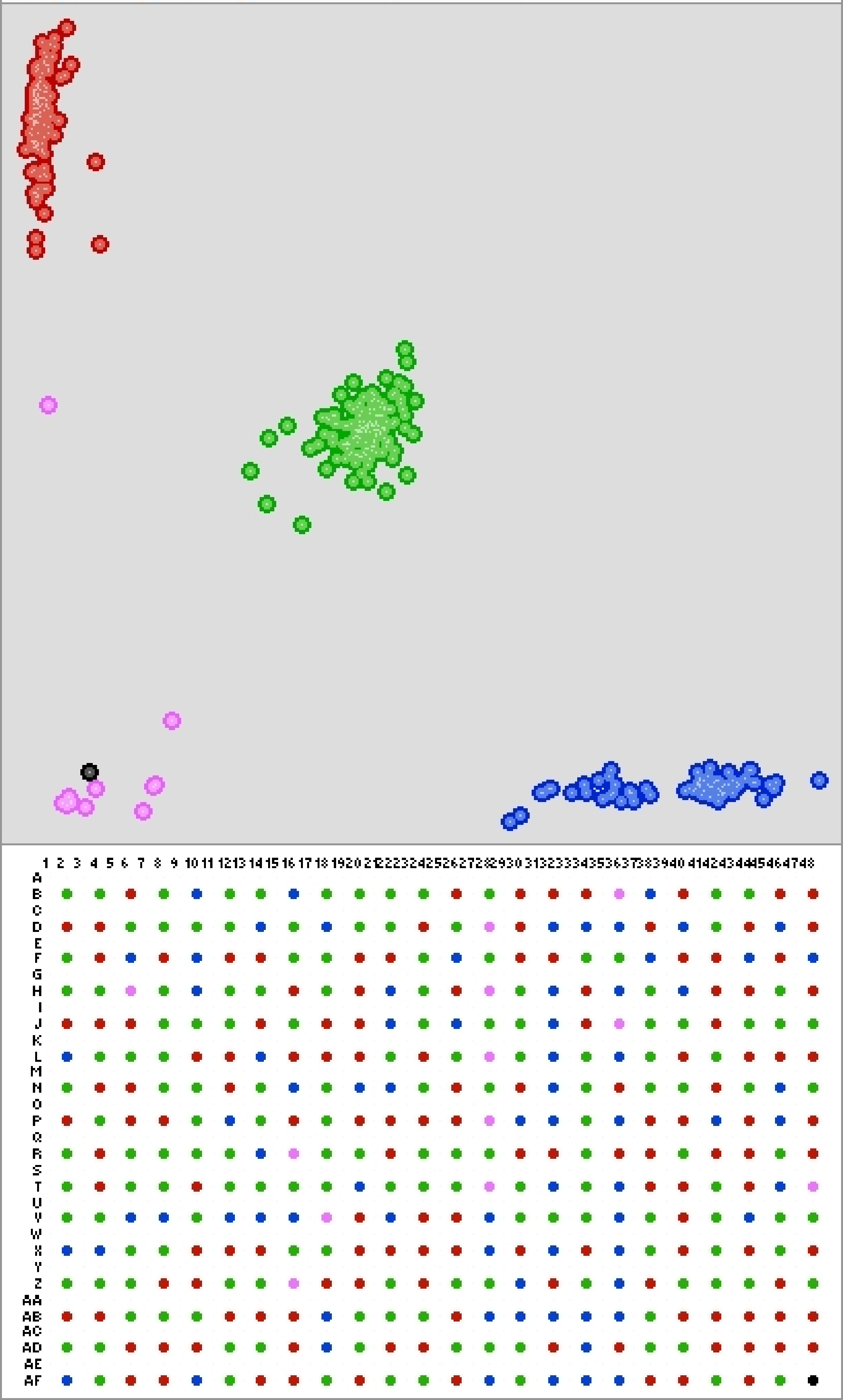

Assay:usp_Sheffield_var1 DNA:Vlasi-1+Vlasi-2+Vlasi-3+Vlasi-4

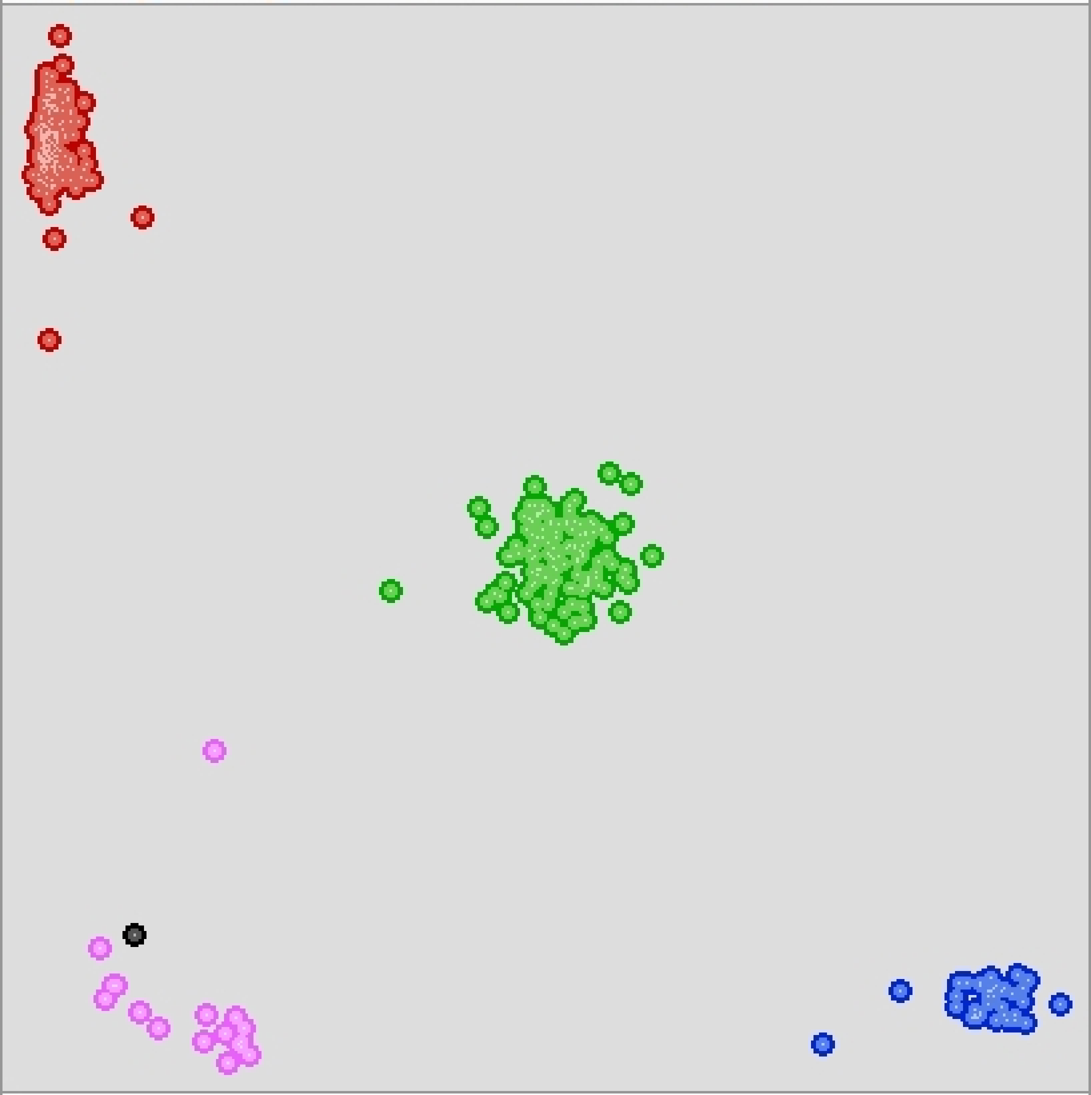

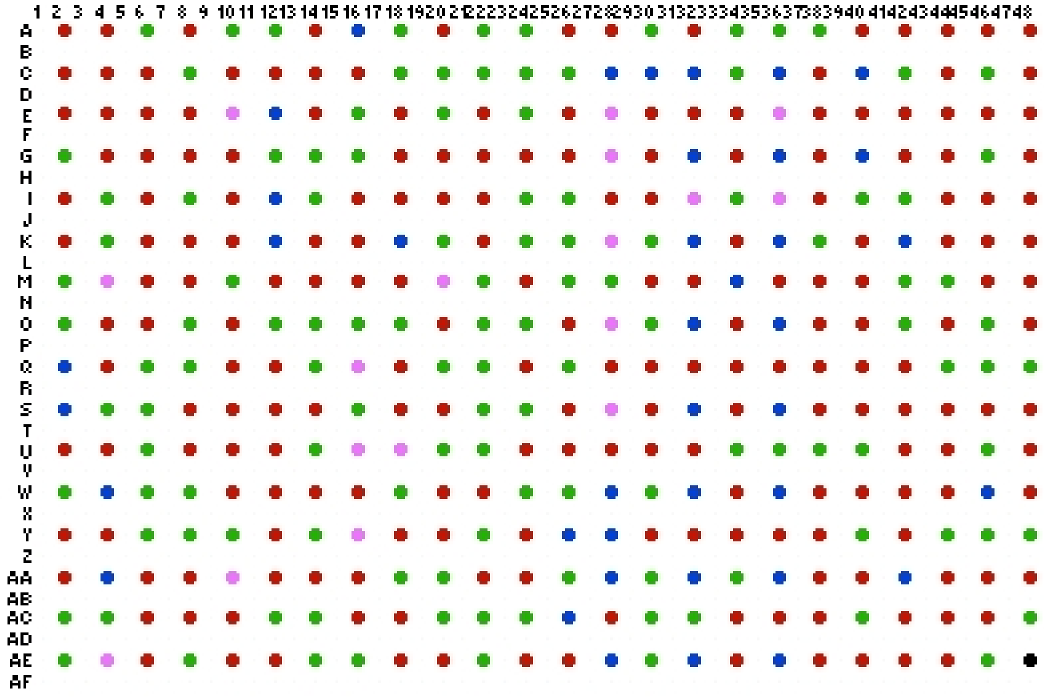

Assay:wiz_Sheffield_var1 DNA:Vlasi-1+Vlasi-2+Vlasi-3+Vlasi-4

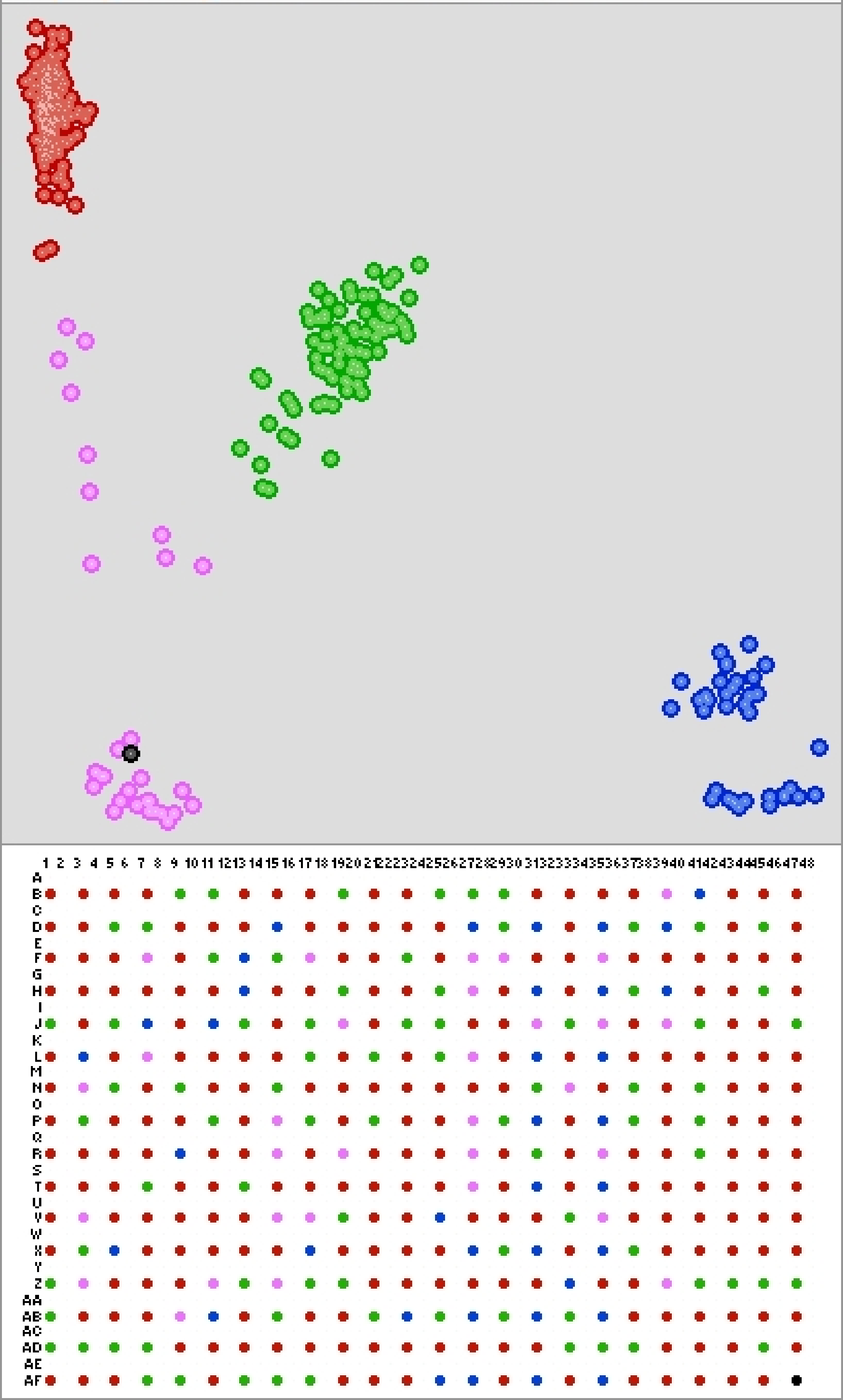

Supplement: Supplemental Information 2 [file peerj-06-5317-s002.pdf]
